# Development of an aerosol intervention for COVID-19 disease: Tolerability of soluble ACE2 (APN01) administered via nebulizer

**DOI:** 10.1371/journal.pone.0271066

**Published:** 2022-07-11

**Authors:** Robert H. Shoemaker, Reynold A. Panettieri, Steven K. Libutti, Howard S. Hochster, Norman R. Watts, Paul T. Wingfield, Philipp Starkl, Lisabeth Pimenov, Riem Gawish, Anastasiya Hladik, Sylvia Knapp, Daniel Boring, Jonathan M. White, Quentin Lawrence, Jeremy Boone, Jason D. Marshall, Rebecca L. Matthews, Brian D. Cholewa, Jeffrey W. Richig, Ben T. Chen, David L. McCormick, Romana Gugensberger, Sonja Höller, Josef M. Penninger, Gerald Wirnsberger

**Affiliations:** 1 Chemopreventive Agent Development Research Group, Division of Cancer Prevention, National Cancer Institute, National Institutes of Health, Bethesda, Maryland, United States of America; 2 Rutgers Institute for Translational Medicine and Science, New Brunswick, New Jersey, United States of America; 3 Rutgers Cancer Institute, New Brunswick, New Jersey, United States of America; 4 Protein Expression Laboratory, National Institute of Arthritis and Musculoskeletal and Skin Diseases, National Institutes of Health, Bethesda, Maryland, United States of America; 5 Department of Medicine I, Laboratory of Infection Biology, Medical University of Vienna, Vienna, Austria; 6 MRIGlobal, Kansas City, Missouri, United States of America; 7 Cancer ImmunoPrevention Laboratory, Frederick National Laboratory for Cancer Research, Frederick, Maryland, United States of America; 8 IIT Research Institute, Chicago, Illinois, United States of America; 9 Apeiron Biologics AG, Campus-Vienna-Biocenter 5, Vienna, Austria; 10 Institute of Molecular Biotechnology of the Austrian Academy of Sciences, Vienna, Austria; 11 Department of Medical Genetics, Life Sciences Institute, University of British Columbia, Vancouver, British Columbia, Canada; Escola Paulista de Medicina, BRAZIL

## Abstract

As ACE2 is the critical SARS-CoV-2 receptor, we hypothesized that aerosol administration of clinical grade soluble human recombinant ACE2 (APN01) will neutralize SARS-CoV-2 in the airways, limit spread of infection in the lung, and mitigate lung damage caused by deregulated signaling in the renin-angiotensin (RAS) and Kinin pathways. Here, after demonstrating *in vitro* neutralization of SARS-CoV-2 by APN01, and after obtaining preliminary evidence of its tolerability and preventive efficacy in a mouse model, we pursued development of an aerosol formulation. As a prerequisite to a clinical trial, we evaluated both virus binding activity and enzymatic activity for cleavage of Ang II following aerosolization. We report successful aerosolization for APN01, retaining viral binding as well as catalytic RAS activity. Dose range-finding and IND-enabling repeat-dose aerosol toxicology testing were conducted in dogs. Twice daily aerosol administration for two weeks at the maximum feasible concentration revealed no notable toxicities. Based on these results, a Phase I clinical trial in healthy volunteers has now been initiated (NCT05065645), with subsequent Phase II testing planned for individuals with SARS-CoV-2 infection.

## Introduction

Early in the COVID-19 pandemic, sequencing of SARS-CoV-2 enabled recognition of the high degree of homology with SARS-CoV and the identification of ACE2 as a candidate receptor for both viruses [1, 2]. A series of publications in early 2020 defined the molecular details regarding structural interactions between the receptor binding domain of SARS-CoV-2 and the ACE2 receptor [3–6]. Blocking the Spike-ACE2 interaction provided a potential anti-SARS-CoV-2 therapeutic strategy and is the basis for virtually all successful vaccine designs [7]. Proteins or peptides interacting with either of the binding partners could have therapeutic potential and some very high affinity binders have been reported [8]. Likewise, engineered antibodies can inhibit the Spike-ACE2 interaction, and several have received Emergency Use Authorization (EUA) as single agents or combinations from the FDA and EMA as systemic therapeutics [9–11]. These may be useful in combination with i.v. therapeutics such as Remdesivir or the recently authorized oral antivirals. Soluble recombinant human ACE2 (APN01) in combination with Remdesivir showed strong additive activity against SARS-CoV-2 in both Vero E6 cells and kidney organoids [12]. As a single agent or in combinations, APN01 may prove to be a robust therapeutic intervention, since all emerging SARS-CoV-2 variants tested retain sensitivity to APN01 [13], including the Omicron variant [14].

After the outbreak of the first SARS virus in 2003, APN01 was developed for systemic treatment of acute respiratory distress syndrome (ARDS) [15, 16]. In this indication, the catalytic activity of ACE2 in cleaving Ang II was exploited to reduce damage to the lung as observed in virus induced ARDS. Phase I and Phase II clinical trials demonstrated that APN01 had an acceptable safety profile and strongly reduced pathogenic Ang II levels [17]. Another group recently reported a gene therapy approach using an evolved ACE2 construct in which the enzymatic activity was inactivated [18]. Our group first reported *in vitro* SARS-CoV-2 neutralizing activity of APN01 using a Swedish isolate in cells and human organoids [19]. Importantly, interactions between Spike proteins of multiple variants of concern and APN01 have been demonstrated to be of considerably higher affinity [13]. Moreover, APN01 can neutralize all tested SARS-CoV-2 variants of concern and variants of interest [13] including the Omicron variant [14] that is driving the current COVID-19 surge.

APN01 has recently undergone a randomized Phase 2 clinical trial for treatment of severe COVID-19 using intravenous administration (NCT04335136, manuscript in preparation). We reasoned that direct introduction of APN01 into the airways could locally neutralize the virus, limiting the spread of infection, and thereby limit damage to the lung. Potent *in vitro* SARS-CoV-2 neutralizing activity of the clinical grade APN01 was confirmed using Vero E6 cells. As a probe for the potential neutralizing activity of APN01 activity following introduction into the airways, we tested intranasal administration in a mouse model of SARS-CoV-2 infection [20]. Results demonstrated strong protective activity, providing experimental support for direct APN01 administration into the airways. The critical path for this intervention includes the development of an aerosol formulation of APN01 that retains both virus-binding activity and enzymatic activity. We report the successful development of inhalable APN01 and results of preclinical toxicology studies that support the safety of this intervention when administered by aerosol. These data pave the way for clinical testing of this intervention strategy in COVID-19 disease.

## Materials and methods

### *In vitro* anti-SARS-CoV-2 neutralizing activity of APN01

Serial dilutions of APN01 were prepared in assay medium (MEM supplemented with 2% fetal bovine serum and 50 μg/ml gentamicin) and a suspension of SARS-CoV-2, USA-WA1/2020 was added to assess neutralization. For assessment of APN01 effects on viability, assay medium without virus was added. After one-hour incubation at 37°C, the dilutions were transferred to 96-well round-bottom plates containing 30,000 Vero E6 target cells per well to achieve a multiplicity of infection (MOI) of 0.001 (one infectious virus particle for each 1000 Vero E6 target cells). Triplicate wells of each APN01 dilution with virus were included and duplicate uninfected wells were included as toxicity controls. Six replicate wells were used to determine control values without APN01 treatment. Incubation was continued for four days (time of maximal cytopathic effect) and cell numbers were assessed with a neutral red endpoint. Cytopathic Effect (CPE) was calculated as the average optical density (OD) for replicate infected and treated wells divided by average control OD X 100 (expressed as a percentage). Viability was calculated as the average optical density for replicate uninfected and treated wells divided by average control OD X 100 (expressed as a percentage). Testing was performed under contract at the Institute for Antiviral Research, Utah State University, Logan, UT. All of the experiments reported here used the same lot of clinical grade APN01 (lot ACE20620-B).

### Activity of APN01 in a mouse model of SARS-CoV-2 infection

Ten-week-old male BALB/c mice (Charles River) received daily intranasal treatments with 100 μg APN01 (five mice) or the respective dilution of vehicle (five mice) in endotoxin-free PBS (Gibco). The first dose was given as a mix with 1 x 10^5^ TCID_50_ of *maVie16*, a mouse-adapted SARS-CoV-2 virus [20], in 50 μl, the remaining doses were administered in 40 μl of endotoxin-free PBS to isoflurane-anesthetized animals. All mice were monitored for health at least daily. Mice either succumbed to the lethal effects of infection or were sacrificed for complete necropsy. Animals surviving for five days following infection were sacrificed by cervical dislocation. All experiments involving SARS-CoV-2 or its derivatives were performed in Biosafety Level 3 (BSL-3) facilities at the Medical University of Vienna and performed after approval by the institutional review board of the Austrian Ministry of Sciences (BMBWF-2020-0.253.770) and in accordance with the directives of the EU.

### Recovery of aerosolized APN01

An adaptor was connected to the mouthpiece port of a PARI LC PLUS nebulizer, secured to a ring stand, to route the aerosol into a custom fabricated condenser consisting of a 4-L polypropylene graduated cylinder lined with downward-spiraling C-Flex tubing (Cole Palmer). The cylinder was filled with an ice water bath and the exit tubing was then fed through a hole into a 50 mL conical collection tube. The nebulizer was connected to a PARI Vios PRO Compressor according to the manufacturer’s instructions. For Trials 1–2, APN01 was diluted to a volume of 4 ml at 2.5 mg/ml, transferred to the nebulizer cup, and nebulized for 24 min. For Trials 3–4, APN01 was diluted to a volume of 4 ml at 0.1 mg/ml and nebulized for 24 min. This resulted in a range of recovery from all trials of 0.570–1.420 ml APN01 volume in the collection tube, corresponding to 15.4–38.4% of the starting volume. Three samples were tested: diluted APN01 sample before addition to the nebulizer cup (Control), unnebulized sample remainder in the nebulizer cup (Pre), and sample obtained from the collection tube after condensation (Post).

### ELISA to assess binding of APN01 to the SARS-CoV-2 receptor binding domain (RBD)

A sandwich ELISA was developed with SARS-CoV-2 Spike RBD-Fc protein bound to the well-bottom surface, then APN01 applied in a liquid phase, and bound APN01 detected with biotinylated goat anti-human ACE2 and streptavidin-horseradish peroxidase. Specifically, 96-well ELISA plates (Thermo-Fisher Scientific, Cat # 439454) were coated with 2 μg/ml SARS-CoV-2 Spike RBD-Fc protein (Sino Biological, Cat # 40592-V02H) in coating buffer (0.1 M carbonate-bicarbonate pH 9.4, Thermo-Fisher Scientific, Cat # 28382). Plates were sealed with adhesive plate seals and incubated overnight at 4°C, then washed 3X with wash buffer (1X PBS + 0.05% Tween 20, Sigma, Cat # P3563-10PAK). Plates were blocked with blocking buffer (1X PBS + 1% BSA), sealed, and incubated for 1 h at r.t. APN01 samples recovered from the nebulization runs were diluted in blocking buffer to 27 μg/ml, as quantified with hACE2 ELISA measurements. Further 1:3 dilutions were conducted using blocking buffer to achieve a seven-point dilution curve. All 7 sample dilutions and a blank were added to the ELISA plate in triplicate. Plates were covered and incubated for 1 h at r.t., then washed 5X with wash buffer. Biotinylated goat anti-human ACE2 (R&D Systems, Cat # BAF933) diluted in blocking buffer was added to plates at 0.4 μg/ml followed by a 1 h incubation at r.t. Plates were washed 5X with wash buffer. Streptavidin-HRP (Thermo-Fisher Scientific, Cat # 21126) diluted in blocking buffer was added to plates at 0.1 μg/mL followed by a 30 min incubation at r.t., then washed 3X with wash buffer. TMB substrate solution (SeraCare, Cat # 5120–0050) was added followed by a 6 min incubation in the dark at r.t. Finally, a stop solution of 1 M H_2_SO_4_ (J.T. Baker, Inc., Cat # 4700–01) was added and plates were gently shaken for 5 s to mix and then absorbance (O.D.) was measured at 450/620 nm on a SpectraMax M5 (Molecular Devices) instrument and data were processed by SoftMax Pro 6.4 software (Molecular Devices). Blank well readings were subtracted from sample dilution readings, and nonlinear regression to derive EC_50_ values was performed through a 4-parameter logistic equation by GraphPad Prism v.8 plotting OD450/620 versus APN01 (ng/ml).

### Angiotensin II cleavage assay

Substrate was prepared by reconstituting MAPL-DNP (Anaspec) in DMSO to make 1 mM stock with gentle mixing by inversion to ensure dissolution. This solution was diluted in assay buffer (10 μM ZnCl_2_, 50 mM MES, 300 mM NaCl, 0.01% Brij L23, pH 6.5) to 1 mM and then further diluted 1:5 in assay buffer to prepare a 0.2 mM working solution of MAPL-DNP. The substrate was prewarmed in an oven at 37°C prior to addition to the sample. APN01 samples recovered from the nebulization runs were diluted in assay buffer to 1.0 μg/ml, as quantified with hACE2 ELISA measurements. Further dilution in assay buffer was conducted to yield 100 ng/ml, 50 ng/ml, and 25 ng/ml solutions at volumes of 1 ml each. The three APN01 dilutions of each sample were loaded into the assay plate (Greiner) in quadruplicate in the center of the plate, avoiding the outer edges, while assay buffer blanks were loaded in columns 2 and 11. Prewarmed 0.2 mM MAPL-DNP substrate was added to all wells in the plate starting with row A and contents in all rows were mixed by pipetting. Plates were read immediately for fluorescence (320/420 nm) in kinetic mode on a SpectraMax M5 (Molecular Devices) instrument at 37°C for 1 h taking readings at 1 min intervals. Background-subtracted and averaged sample values (RFU) were plotted in Excel versus time, and slopes were determined (ΔRFU/min) for each sample. Data were converted to (ΔRFU/min)/ng APN01, where ΔRFU/min is the slope and ng APN01 was determined by multiplying the ng/mL concentration by 0.05 for the 50 μl of sample used per well.

### ELISA to assess APN01 in dog plasma

Serum samples were analyzed for levels of APN01 using a commercial kit (Human ACE2 ELISA Kit PicoKine™; SKU EK0997; Boster Bio, Pleasanton, CA).

### HPLC quantitation of APN01 on filters

The aerosol mass concentration in each oronasal inhalation exposure system was determined by collecting the aerosol on glass-fiber filters. Samples were collected at a constant flow rate equal to the port flow of the delivery tube, and the total volume of air samples was measured by a dry-gas meter. One aerosol sample per dose level was collected during each exposure. Filter samples were extracted with phosphate buffered saline (PBS) and stored refrigerated (approximately 4°C). Filter samples were analyzed for levels of APN01 using high performance liquid chromatography (HPLC) with UV wavelength detection according to a validated method. During method validation, quality control (QC) samples prepared in PBS at target APN01 concentrations of 49 and 245 μg/mL were demonstrated to be stable for at least 22 days when stored refrigerated (111% and 104% of the original value, respectively) and for at least 2 days when stored at room temperature (111% and 103% of the original value, respectively). For instrument calibration, primary standard solutions of APN01 with target concentrations of 490 μg/mL were prepared by dilution of 0.5 mL of the test article formulation (4.9 mg/mL ACE2 content per the Certificate of Analysis) in PBS in a 5 mL volumetric flask and filling to volume. Standard curve calibrators were prepared at target concentrations of 29.4, 44.1, 58.8, 117.6, 176.4, 264.6 and 352.8 μg/mL by diluting the 490 μg/mL primary standards with PBS. QC samples of APN01 in PBS were prepared at low (50 μg/mL) and high (250 μg/mL) target concentrations and were analyzed along with the filter samples. Calibrator, QC and processed filter samples were analyzed by HPLC using the following equipment and conditions: HPLC System: Waters Alliance 2695; HPLC Detector: Waters 2487 Dual λ Absorbance Detector; Data System: Waters Empower3; HPLC Column: Agilent PLRP-S (reversed phase); 300 Å; 250 × 4.6 mm; 8 μm; Column Temperature: 25°C; Sample Temperature: 4°C; Injection Volume: 5 μL; Flow Rate: 1.0 mL/minute; Mobile Phase (MP): A: 0.1% trifluoroacetic acid in ASTM type I water, B: 0.1% trifluoroacetic acid in acetonitrile. The retention time of APN01 was approximately 5 to 6 minutes. The calibration curve was calculated from the linear regression of the calibrators’ peak areas versus their respective concentrations. The concentrations of test article in the processed filter samples were determined from each sample’s peak area using the linear regression parameters derived from the calibration curves and correcting the resulting concentration by multiplying by the appropriate dilution factor, as applicable. A representative chromatogram is shown in S1 Fig.

### Aerosol particle size distribution

Aerosol particle size distribution was determined twice per group during the study by collecting size-segregated aerosol samples using a 10-stage quartz crystal microbalance (QCM) cascade impactor (California Measurements Inc.; Sierra Madre, CA). The aerosol output from one port of the exposure system was connected to the QCM and was sampled at least once per inhalation exposure level. The mass median aerodynamic diameter (MMAD) and geometric standard deviation (GSD) of the test aerosol were calculated from the mass accumulated on each collection stage of the QCM by using a validated computer program (QCMSIZE) that was developed at IITRI.

### Toxicology studies

Toxicology studies were conducted according to Good Laboratory Practices. Animal studies were performed in full compliance with the Animal Welfare Act and in accordance to the NIH Guide for the Care and Use of Laboratory Animals. To minimize stress on the animals during the aerosol exposure, they were conditioned to placement and restraint in the oronasal inhalation exposure system for periods of 10 minutes, 20 minutes and 40 minutes per day prior to initiation of APN01 exposures. On Study Day 15 (one day after the end of the 14-day dosing period), all dogs in the study were anesthetized with sodium pentobarbital and euthanized by exsanguination for the scheduled necropsy. A complete necropsy with tissue collection was performed, and all tissues from all study animals were evaluated microscopically. The full toxicology report with detailed decriptions of study design, methods, and results is provided in the S1 File.

## Results and discussion

### *In vitro* anti-SARS-CoV-2 activity of APN01

**Fig 1:**
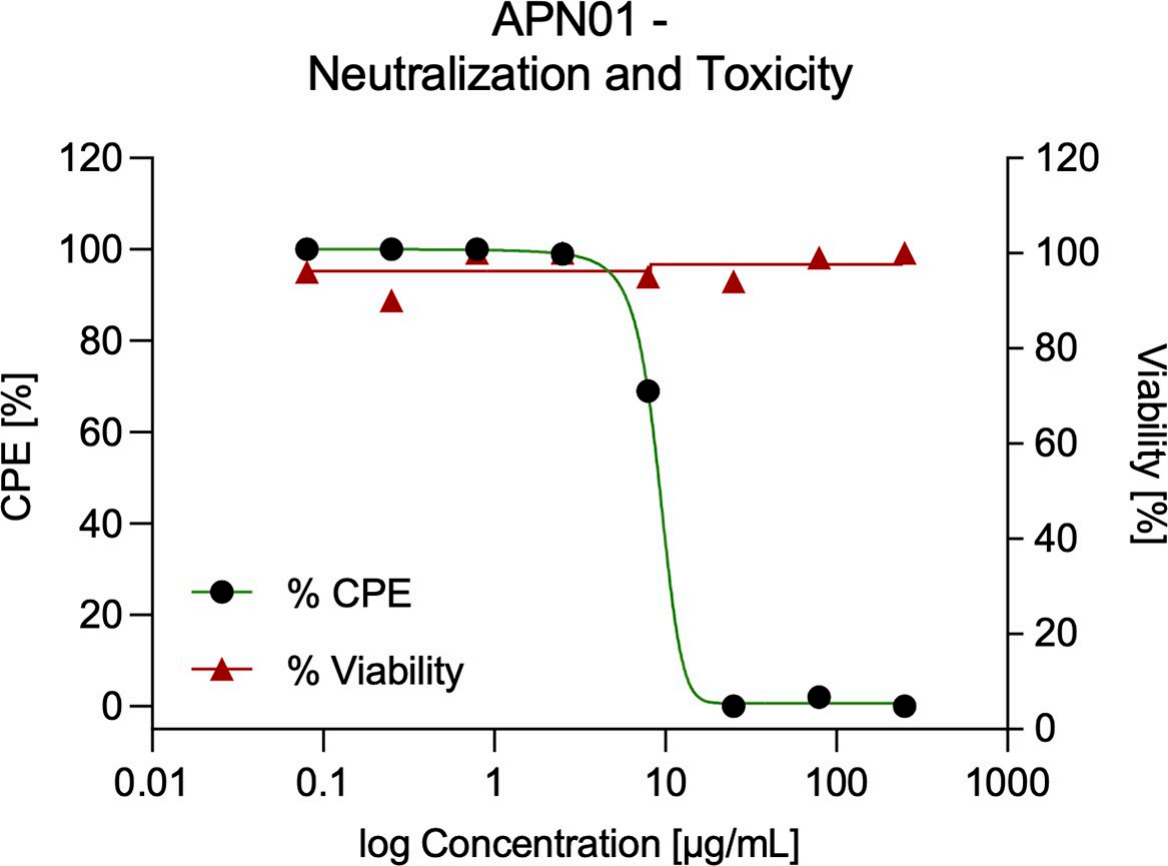
APN01 neutralization of SARS-CoV-2 in the absence of cytotoxicity. Serial dilutions of APN01 were prepared in assay medium (MEM supplemented with 2% fetal bovine serum and 50 μg/mL gentamicin) and a suspension of SARS-CoV-2 (USA-WA1/2020) was added to assess neutralization. For assessment of APN01 on viability, assay medium without virus was added. After one-hour incubation at 37°C, the dilutions were transferred to wells containing Vero E6 target cells (Multiplicity Of Infection, 0.001). Incubation was continued for four days and cell numbers were assessed with a neutral red endpoint. Cytopathic Effect (CPE) was calculated as the average optical density (OD) for replicate infected and treated wells divided by average control OD X 100 (expressed as a percentage). Viability was calculated as the average optical density for replicate uninfected and treated wells divided by average control OD X 100 (expressed as a percentage).

While anti-SARS-CoV-2 activity of APN01 has been reported previously [19], we first evaluated the *in vitro* neutralizing activity of the lot of cGMP produced, i.v. injectable APN01 used for aerosol formulation and toxicology studies. As shown in Fig 1, one-hour exposure to concentrations of APN01 as low as 25 μg/ml completely neutralized SARS-CoV-2 as assessed in a four-day cytopathic effect (CPE) assay on Vero E6 cells. Thus, clinical grade ACE2 exhibits antiviral activity without any toxic effects.

### APN01 protects from respiratory SARS-CoV-2 infections

**Fig 2:**
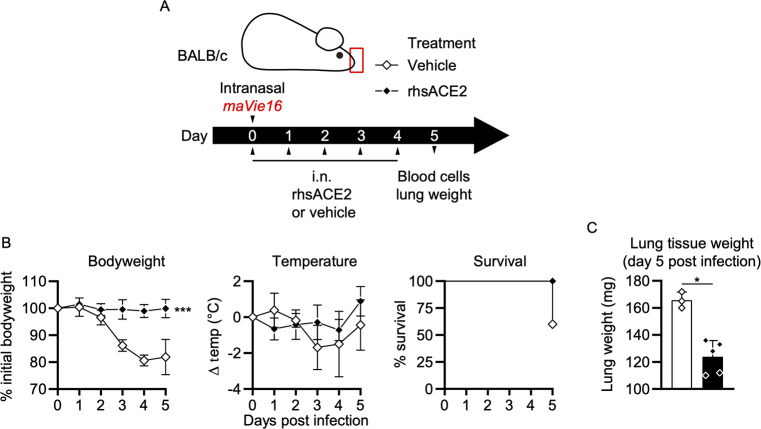
Intranasal APN01 protects from disease in a mouse-adapted SARS-CoV-2 respiratory infection model. (A) Experimental outline for infection of male BALB/c mice (n = 5 for both groups) with SARS-CoV-2 (strain *maVie16*) and daily intranasal treatment with APN01 for five days. (B) Body weight, temperature and survival curves for infected BALB/c mice treated with vehicle control or APN01. Body weights and temperature were compared using mixed-effect analyses. Survival differences were analyzed using a Mantel-Cox test. (C) Lung tissue weight as assessed 5 days after infection of mice; data were analyzed with the Mann-Whitney test. Statistical significances are indicated by asterisks (p-value < 0.05: *; p-value < 0.001: ***).

We have recently developed an animal model that recapitulates SARS-CoV-2 infections and results in severe lung pathologies, weight loss, and, dependent on the mouse strain, death of the infected mice. This model is based on a mouse-adapted SARS-CoV-2 virus (termed *maVie16*) and expression of ACE2 was found to be essential for infection and disease [20]. We used this model to test whether application of clinical grade APN01 into the upper respiratory tract with SARS-CoV-2 *maVie16* would neutralize the virus as indicated by reduced evidence of disease (Fig 2A). Of note, SARS-CoV-2 *maVie16* can still effectively infect human cells, i.e. the *maVie16* Spike can bind to human ACE2. Whereas *maVie16* infection of Balb/c mice resulted in rapid weight loss, lung pathologies, and eventual death, co-administration of APN01 protected from lung damage and weight loss (Fig 2B). Treatment-related changes in body temperature did not reach statistical significance. Importantly, APN01 administration resulted in 100% survival of the SARS-CoV-2 infected mice whereas all controls succumbed to the infection (infected mice surviving to day 5 were moribund when sacrificed). Increase in lung tissue weight, a correlate of severe infection [21], was also significantly reduced by intranasal APN01 treatment (Fig 2C). Thus, respiratory delivery of APN01 can protect from SARS-CoV-2 infections.

### APN01 aerosolization

**Fig 3:**
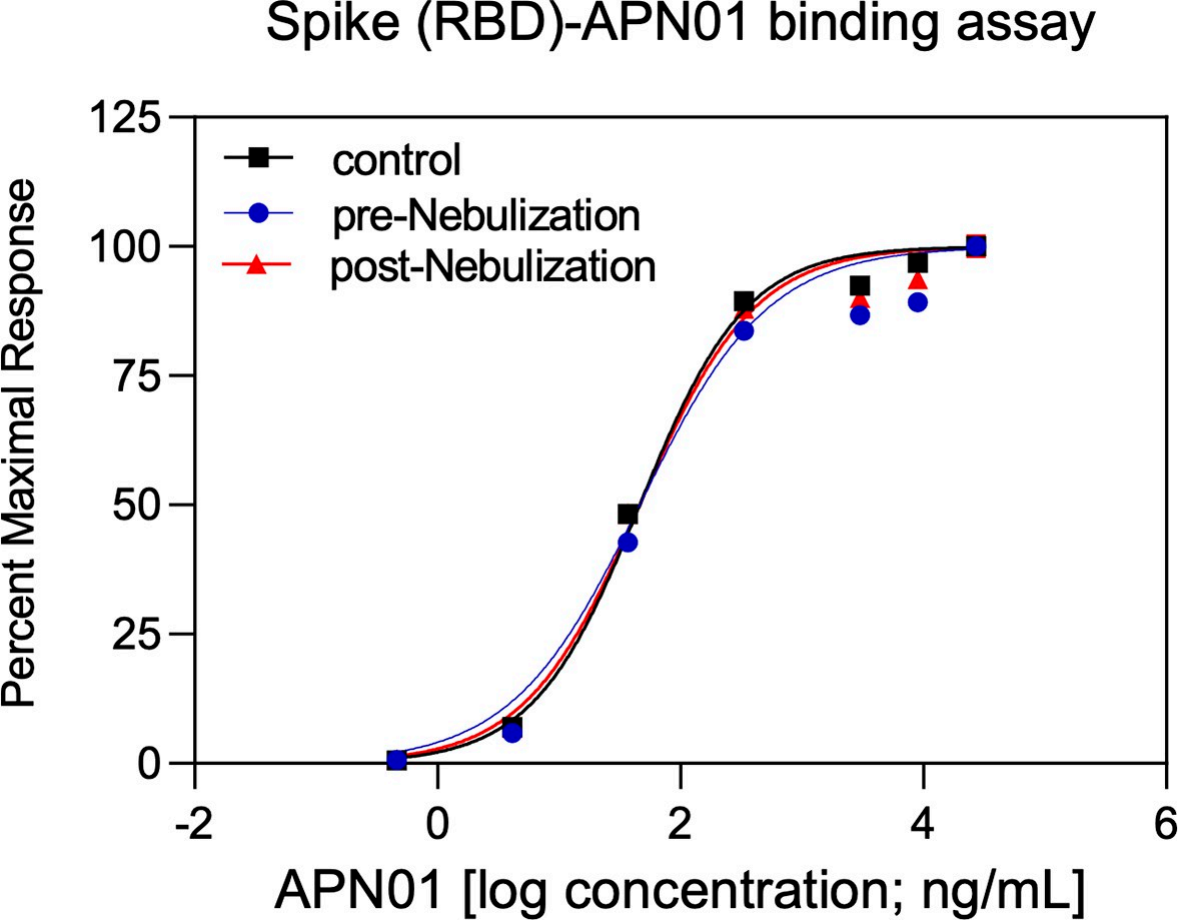
APN01 binding to the SARS-CoV-2 RBD is not altered by nebulization. Binding of increasing doses of APN01 to plate-immobilized RBD domain was assessed by ELISA. Curves depict pre-nebulization samples of APN01 (material remaining as liquid in the nebulizer cup), APN01 collected after nebulization (post-nebulization), and non-treated control APN01. For this experiment, APN01 was aerosolized at 2.5 mg/ml.

To aerosolize APN01 for preclinical studies in a scalable manner to facilitate widespread clinical use, we selected PARI LC PLUS nebulizers. These are effective and standardized devices used in hospital and at-home applications and have remained widely available during the pandemic. Aerosolized APN01 was collected using a custom fabricated condenser and analyzed for virus-binding activity and enzymatic activity for cleaving a fluorogenic substrate. Clinical grade APN01 is formulated for i.v. use at 5 mg/ml. Recognizing that *in vitro* anti-SARS-CoV-2 activity was observed at concentrations as low as 25 μg/ml for the reference USA-WA1/2020 virus (Fig 1) we aerosolized a range of concentrations from 100 μg/ml to 2.5 mg/ml. The concentration of recovered APN01 was assessed by enzyme-linked immunosorbent assay (ELISA) measurements. Binding assays were conducted by coating plates with a SARS-CoV-2 Spike RBD-Fc fusion protein and assessing APN01 binding (Fig 3). APN01 binding to the SARS-CoV-2 Spike protein was almost identical after nebulization, indicating that the integrity of the protein structure was maintained. Similar results were obtained in replicate assays for APN01 nebulized at 2.5 mg/ml and 0.1 mg/ml (S1 Table).

**Fig 4:**
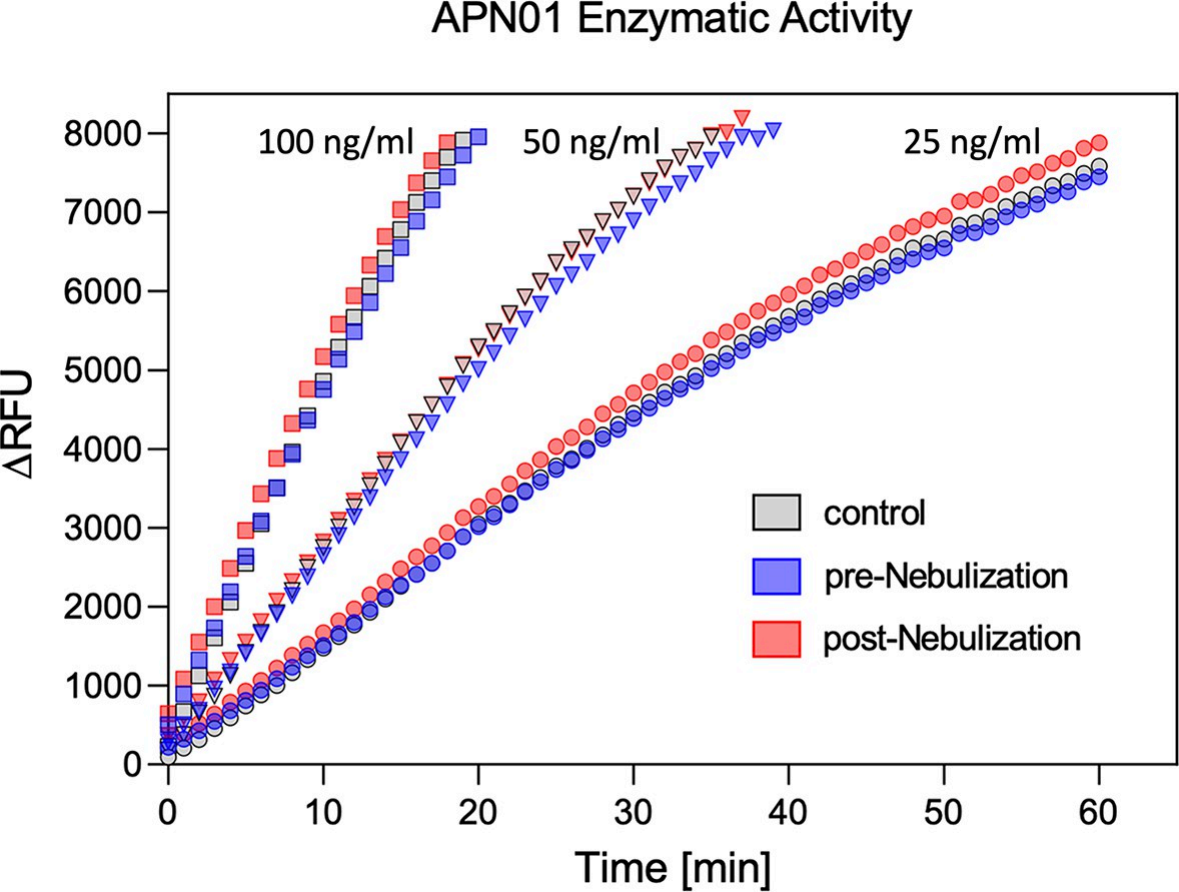
APN01 enzymatic activity is not altered by nebulization. Three samples were tested as defined in the legend to Fig 3. Samples were diluted to APN01 concentrations of 25 ng/ml, 50 ng/ml, and 100 ng/ml and assayed for enzymatic function by cleavage of quenched fluorogenic MAPL-DNP substrate and subsequent measurement of fluorescence activity. Data are plotted as ΔRFU (change in relative fluorescence units) vs. time (min). One representative experiment out of 4 biological replicates is shown.

To test for a potential effect of nebulization on the enzymatic activity of APN01, cleavage of a fluorogenic peptide substrate was assessed. Kinetic analysis was performed at multiple concentrations of APN01 and expressed as the change in Relative Fluorescence Units per minute per ng [(ΔRFU/min)/ng]. Results for a representative assay are illustrated in Fig 4. Enzymatic activity for cleaving the fluorescently labeled peptide substate by APN01 aerosolized at lower concentration was also not affected. S2 Table presents results from replicate experiments with APN01 nebulized at 2.5 and 0.1 mg/ml. In summary, aerosolization of APN01 did not affect the structural integrity of APN01 in terms of its ability to bind SARS-CoV-2 Spike RBD or its enzymatic activity.

### Toxicologic assessment of aerosolized APN01

We next performed a comprehensive evaluation of the potential toxicity of twice daily inhalation administration of APN01 aerosols to beagle dogs for 14 consecutive days. The goals of this study, which was designed to support regulatory filings, included: (1) characterization of toxicities associated with repeat-dose inhalation exposure to APN01 aerosols; (2) identification of sensitive target tissues of inhaled APN01; (3) characterization of serum levels and toxicokinetics (TK) of inhaled APN01; and (4) identification of a No Observed Adverse Effect Level [NO(A)EL]. The study design and different test cohorts are summarized in Table 1.

**Table 1:** Toxicology design. Nebulized APN01 was administrated twice a day for 14 consecutive days at a low APN01 concentration (0.019 mg/L), a mid concentration (0.038 mg/L), and a high APN01 concentration (0.075 mg/L) for each single administration. The high APN01 dose was demonstrated to be the maximum feasible concentration (MFC), obtained by aerosolizing the i.v. formulation in a dose range-finding study.

Group	Number of Dogs (M + F)	Agent	Number and Duration of Daily Exposures	Number of Exposure Days	Target APN01 Concentration in Test Atmosphere (mg/L)
1	3 + 3	Saline (control)	2 x 60 minutes	14	0 (Control)
2	3 + 3	Vehicle (control)	2 x 60 minutes	14	0 (Control)
3	3 + 3	APN01–Low	2 x 60 minutes	14	0.019
4	3 + 3	APN01–Mid	2 x 60 minutes	14	0.038
5	3 + 3	APN01–High	2 x 60 minutes	14	0.075

For treatment of experimental animals, multiple PARI LC PLUS nebulizers were multiplexed through a distribution plenum with hoses connected to oronasal masks. Analytical data (obtained by HPLC analysis of filters) demonstrated that test aerosols consistently achieved target exposure concentrations. Characteristics of the generated APN01 aerosols are summarized in Table 2. Particle size distribution data showed that the generated particles were within the respirable size range for dogs [22] and met targets for both median mass aerodynamic diameter (MMAD) and geometric standard deviation (GSD).

**Table 2:** Characterization of APN01 aerosols. Samples were collected at morning (AM) and afternoon (PM) exposures and characterized for APN01 concentrations to enable dose calculation and assess particle sizes to confirm respirability. GSD, geometric standard deviation; MMAD, median mass aerodynamic diameter.

Group (Treatment and Target APN01 Concentration)	Sample Collection Time Point	Measured APN01 Concentration (mg/L) ^a^	Particle Size Distribution	
			MMAD ^a^ (μm)	GSD Range
1 (Saline Control)	AM	0.000 ± 0.0000	1.06 ± 0.113	2.25–2.36
	PM	0.000 ± 0.0000		
2 (Vehicle Control)	AM	0.000 ± 0.0000	1.36 ± 0.078	2.20–2.56
	PM	0.000 ± 0.0000		
3 (APN01–0.019 mg/L)	AM	0.017 ± 0.0014	1.57 ± 0.566	1.53–2.28
	PM	0.017 ± 0.0028		
4 (APN01–0.038 mg/L)	AM	0.034 ± 0.0034	1.92 ± 0.205	1.80–2.41
	PM	0.035 ± 0.0024		
5 (APN01–0.075 mg/L)	AM	0.078 ± 0.0062	2.00 ± 0.290	1.87–1.95
	PM	0.082 ± 0.0058		

^a^ Mean ± standard deviation

The inhaled dose of APN01 following a one hour exposure was calculated from the aerosol concentration, average volume inhaled (Minute Volume in L/min), exposure time and animal body weights. Calculated inhaled APN01 levels for each group after a single exposure (Study Day 1) and after repeat-dose exposure (after the first exposure on Study Day 11) are shown in Table 3. Inhaled doses were concentration dependent and consistent over the treatment interval. Even dosed at the lowest concentration, the inhaled dose was >0.3 mg/kg/exposure, far exceeding the minimum virus neutralizing activity of APN01.

**Table 3:** Calculated inhaled dose levels. At all concentrations, the inhaled APN01 dose exceeded SARS-CoV-2 neutralizing concentrations. This was the case for both the initial administration and after repeated exposures.

Group (Treatment)	Sex	Test Atmosphere APN01 Concentration ^a^ (mg/L)		Minute Volume (L/min)		Mean Body Weight (kg)		Calculated Inhaled APN01 Dose (mg/kg/Exposure) ^b^	
		Post Single Exposure^c^	Post Repeated Exposures^d^	Post Single Exposure^c^	Post Repeated Exposures^d^	Post Single Exposure^c^	Post Repeated Exposures^d^	Post Single Exposure^c^	Post Repeated Exposures^d^
1 (Saline Control)	M	0	0	3.0153	2.9915	9.24	9.15	0.0	0.0
	F	0	0	2.5442	2.5470	7.49	7.50	0.0	0.0
2 (Vehicle Control)	M	0	0	2.8906	2.9650	8.77	9.05	0.0	0.0
	F	0	0	2.4337	2.4864	7.09	7.28	0.0	0.0
3 (APN01; 0.019 mg/L)	M	0.019	0.017	2.8773	2.9332	8.72	8.93	0.38	0.34
	F	0.016	0.013	2.3585	2.3529	6.82	6.80	0.33	0.27
4 (APN01; 0.038 mg/L)	M	0.027	0.035	2.9199	2.8986	8.88	8.80	0.53	0.69
	F	0.026	0.035	2.3753	2.3725	6.88	6.87	0.54	0.73
5 (APN01; 0.075 mg/L)	M	0.068	0.078	2.9066	2.9119	8.83	8.85	1.34	1.54
	F	0.073	0.080	2.4947	2.5470	7.31	7.50	1.49	1.63

^a^ Determined by HPLC

^b^ Inhaled dose calculated as: Dose (mg/kg/day) = (C × MV × T) / BW,

where C is the test atmosphere concentration (mg/L) per group (first of two daily exposures)

MV is the group mean minute volume for the appropriate sex, calculated as (L/min) = 0.499 × BW^0.809^ *

T is the duration of exposure (60 minutes)

BW is the group mean body weight for the appropriate sex.

* Bide, RW, et al. *J*. *Appl*. *Toxicol*. **20**, 273–290 (2000)

^c^ Study Day 1 after the first of two daily exposures.

^d^ Study Day 11 after the first of two daily exposures (21 total exposures); data from this time point were used since this was the last available non-fasted body weight (twice weekly weighing schedule).

We also tested whether inhalation of APN01 might result in dispersion of APN01 outside the respiratory system. Systemic exposure [defined as serum levels of APN01 above the limit of quantitation (LOQ)] was very low in dogs in the low-dose and mid-dose groups as assessed on Days 1 and 14; in both groups, serum levels of APN01 were below the LOQ (0.5 ng/mL) in most animals at most time points (Table 4). In the high-dose group, serum levels of APN01 on Days 1 and 14 were above the LOQ in 5 out of 6 dogs at all time points tested after 0.5 hr following inhalation. Although interanimal variability was substantial, mean C_max_ (pooled across both sexes) in the high-dose group was approximately 8 ng/mL on both Day 1 and Day 14. The systemic exposure to APN01 following aerosol administration was far below the levels attained following i.v. administration in the clinic where plasma concentrations have been reported in excess of 1000 ng/mL [23].

**Table 4:** Serum levels of inhaled APN01 and toxicokinetic parameters. Dog serum samples were analyzed for levels of APN01 using ELISA. At scheduled blood sampling times (Days 1 and 14), serum was sampled from individual animals in Groups 3–5 and subsequently used to model toxicokinetic (TK) parameters. Reported TK parameters are: time at which maximum concentration was observed (T_max_), maximum concentration observed (C_max_), and area under the concentration curve at six hours (AUC_6hr_). Terminal phase parameters [e.g., T_1/2_] could not be calculated reliably for any of the profiles due to a lack of amenable data in the terminal phase of the profiles. Data for males and females are shown.

Group	Target Test Atmosphere APN01 Concentration (mg/L)	Estimated Mean Inhaled APN01 Dose (mg/kg)	Mean / *Median* Group TK Parameter			
			n^a^	T_max_ (hr)	C_max_ (ng/mL)	AUC_6hr_ (hr*ng/mL)
**MALES**						
**Day 1**						
3	0.19	0.38	2	*1*	1.26	--
4	0.038	0.53	3	*2*	1.92	16.3
5	0.075	1.34	3	*1*	4.38	21.1
**Day 14**						
3	0.19	0.34	1	*1*	1.04	--
4	0.038	0.69	3	*0*.*5*	1.44	--
5	0.075	1.54	3	*1*	7.96	38.0
**FEMALES**						
**Day 1**						
3	0.19	0.33	3	*6*	3.63	20.1
4	0.038	0.54	2	*1*	1.64	--
5	0.075	1.49	3	*6*	11.5	54.0
**Day 14**						
3	0.19	0.27	2	*0*.*25*	3.73	13.5
4	0.038	0.73	2	*0*.*38*	2.26	9.87
5	0.075	1.63	3	*0*.*5*	7.50	28.9

^**a**^ Number of animals with measurable serum concentration at one or more time points.

Toxicology endpoints also included mortality/moribundity observations; clinical observations for signs of toxicity; physical examinations; heart rate and blood pressure measurements; body weight measurements; food consumption measurements; ophthalmic examinations; electrocardiographic evaluations; respiratory function evaluations; measurements of blood oxygen saturation and pH; neurotoxicity evaluations (functional observational battery [FOB]); clinical pathology assessments (clinical chemistry, hematology, coagulation, and urinalysis); quantitation of serum drug levels; limited modeling of serum toxicokinetics (TK); gross pathology at necropsy; organ weights; and microscopic evaluation of tissues. For a full description see the S1 File.

No early deaths occurred during the study and no gross clinical signs of toxicity were seen in any study animal. Inhalation administration of APN01 aerosols had no effects on body weight, food consumption, clinical pathology parameters, heart rate, blood pressure, electrocardiography, blood oxygen saturation, blood pH, FOB parameters, or ophthalmology. Respiratory function evaluations (respiratory rate, tidal volume and minute volume) were inconclusive due to excitement and/or panting exhibited by study animals during measurement periods. Organ weights were comparable in all study groups. No gross or microscopic pathology was linked to APN01 administration. Moreover, no evidence of systemic or organ-specific toxicity was identified in any dog receiving twice daily 60-minute exposures to APN01 aerosols at all target concentrations of 0.019, 0.038, or 0.075 mg/L for 14 consecutive days. On this basis, the NO(A)EL was determined to be the highest dose investigated, i.e. 0.075 mg/L. Thus, in agreement with toxicologic assessments of systemic exposure of APN01 in rodent and non-rodent species, APN01 exhibited an excellent tolerability and safety profile upon administration as an aerosol.

## Discussion

The results presented here support the feasibility of aerosol administration of APN01 for treatment of SARS-CoV-2 infection. APN01 retains virus binding and enzymatic activities following aerosolization. The aerosol generated using a widely available commercial nebulizer has a particle size distribution consistent with delivery throughout the respiratory tract and could be delivered repeatedly at high doses to dogs without evidence of toxicities. The observation of *in vitro* SARS-CoV-2 neutralization at concentrations as low as 25 μg/ml suggests that aerosol administration should deliver effective antiviral therapy to the airways. While the intranasal studies reported here were conducted in a preventive mode, similar studies using soluble recombinant mouse ACE2 and the same SARS-COV-2 derivative demonstrated that a therapeutic effect could be demonstrated when treatment was initiated up to 48 hours following infection [20].

Recent characterization of the activity of a modified ACE2 gene therapy construct designed for intranasal administration [18] is consistent with our findings [13, 14] of increased affinity of multiple SARS-CoV-2 variants for ACE2 and increased susceptibility to neutralization. It is notable that the gene therapy construct could be delivered without apparent toxicity in mice and non-human primates. This construct was engineered to eliminate ACE2 enzymatic activity and minimize off-target effects in the gene therapy setting. As shown here, aerosolized APN01 retains enzymaic activity, which can compensate for viral down-regulation of ACE2 expression in cells infected with virus, and was the basis for its original use in acute respiratory distress syndrome.

In the toxicology study only a single species of experimental animals is included. Additional confidence in the results could be generated by including non-human primates in the analysis. Of note, mice also did not show any signs of pathologies when they received APN01 into their respiratory system for 5 day preventive efficacy studies. Moreover, the prior experience in clinical administration of APN01 in severe COVID-19 patients *via* the i.v. route, without serious adverse events (manuscript in preparation), has supported moving ahead with Phase I testing using a conservative dose-escalation strategy. A starting clinical dose of ¼ the maximum feasible concentration for 15 minutes provides an estimated 100-fold safety margin assuming 100% aerosol deposition when compared to the NO(A)EL observed in dogs. Escalation to twice per day and then increasing concentrations of ½ to the maximum feasible concentration is the design. A Phase I trial for safety and tolerability of aerosolized APN01 in healthy volunteers is currently underway (NCT05065645) which will be followed by Phase II trials in individuals infected with SARS-CoV-2. The latter trials are expected to use viral clearance as the primary endpoint with severe disease and hospitalization as secondary endpoints.

## Conclusions

Our study demonstrates the feasibility and excellent safety profile of APN01 delivered as an aerosol in a toxicologic assessment in dogs. These data, along with the observation of activity against all variants of concern as of this date [13,14], highlight the potential of aerosolized APN01 to serve as an SARS-CoV-2 therapeutic.

## Supporting information

S1 Fig HPLC chromatogram illustrating quantitation of APN01 extracted from filters placed in the nebulized atmosphere at 0.075 mg/L APN01. Absorbance Units (AU) monitored at 220 nm are plotted as a function of time. Chromatography conditions are described in Materials and Methods. APN01 was resolved as a single peak eluting between five and six minutes. The concentrations of test article in the processed filter samples were determined from each sample’s peak area using the linear regression parameters derived from the calibration curves and correcting the resulting concentration by multiplying by the appropriate dilution factor, as applicable. In addition to supporting quantitation of APN01 in the nebulized atmosphere, this result supports maintenance of the physical integrity of APN01 during the process of aerosolization.
(TIF)

S1 Table ACE2 binding to SARS-CoV-2 Spike protein is not significantly altered after nebulization. All samples from trials 1–4 were normalized to 27 μg/ml according to R&D Systems ELISA measurements and assayed for binding to the SARS-CoV-2 Spike RBD. Data are recorded as EC_50_ values in ng/ml with 95% confidence intervals. The differences between Pre-Nebulization and Post-Nebulization binding activity are indicated as a % change for each APN01 dilution. In this Table, Pre-Nebulization corresponds to the Control samples in Figs 3 and 4 of the main text.
(XLSX)

S2 Table APN01 enzymatic function remains unaltered after nebulization. Data from trials 1–4 were recorded as (ΔRFU/min)/ng for the three starting dilutions of APN01 at 25 ng/ml, 50 ng/ml, 100 ng/ml. The difference between Pre-Nebulization and Post-Nebulization enzymatic activity is indicated as a % change for each APN01 dilution and the mean change and standard deviation across all three dilutions. In this Table, Pre-Nebulization corresponds to the Control samples in Figs 3 and 4 of the main text.
(XLSX)

S1 File Detailed aerosol toxicology report. (ZIP)

## References

[1] Yan RH, Zhang YY, Li YN, Xia L, Guo YY, Zhou Q. Structural basis for the recognition of SARS-CoV-2 by full-length human ACE2. Science. 2020;367(6485):1444–1448. doi: 10.1126/science.abb2762 32132184 PMC7164635

[2] Hoffmann M, Kleine-Weber H, Schroeder S, Kruger N, Herrler T, Erichsen S, et al. SARS-CoV-2 Cell Entry Depends on ACE2 and TMPRSS2 and Is Blocked by a Clinically Proven Protease Inhibitor. Cell. 2020;181(2):271–280. doi: 10.1016/j.cell.2020.02.052 32142651 PMC7102627

[3] Walls AC, Park YJ, Tortorici MA, Wall A, McGuire AT, Veesler D. Structure, Function, and Antigenicity of the SARS-CoV-2 Spike Glycoprotein. Cell. 2020;181(2):281–292. doi: 10.1016/j.cell.2020.02.058 32155444 PMC7102599

[4] Shang J, Ye G, Shi K, Wan YS, Luo CM, Aihara H, et al. Structural basis of receptor recognition by SARS-CoV-2. Nature. 2020;581(7807):221–224. doi: 10.1038/s41586-020-2179-y 32225175 PMC7328981

[5] Wang QH, Zhang YF, Wu LL, Niu S, Song CL, Zhang ZY, et al. Structural and Functional Basis of SARS-CoV-2 Entry by Using Human ACE2. Cell. 2020;181(4):894–904. doi: 10.1016/j.cell.2020.03.045 32275855 PMC7144619

[6] Wrapp D, Wang NS, Corbett KS, Goldsmith JA, Hsieh CL, Abiona O, et al. Cryo-EM structure of the 2019-nCoV spike in the prefusion conformation. Science. 2020;367(6483):1260–1263. doi: 10.1126/science.abb2507 32075877 PMC7164637

[7] Creech CB, Walker SC, Samuels RJ. SARS-CoV-2 Vaccines. Jama-J Am Med Assoc. 2021;325(13):1318–1320. doi: 10.1001/jama.2021.3199 33635317

[8] Linsky TW, Vergara R, Codina N, Nelson JW, Walker MJ, Su W, et al. De novo design of potent and resilient hACE2 decoys to neutralize SARS-CoV-2. Science. 2020;370(6521):1208–1214. doi: 10.1126/science.abe0075 33154107 PMC7920261

[9] Jiang SB, Hillyer C, Du LY. Neutralizing Antibodies against SARS-CoV-2 and Other Human Coronaviruses. Trends Immunol. 2020;41(5):355–359. doi: 10.1016/j.it.2020.03.007 32249063 PMC7129017

[10] Wang YQ, Zhang L, Sang L, Ye F, Ruan SC, Zhong B, et al. Kinetics of viral load and antibody response in relation to COVID-19 severity. J Clin Invest. 2020;130(10):5235–5244. doi: 10.1172/JCI138759 32634129 PMC7524490

[11] Weinreich DM, Sivapalasingam S, Norton T, Ali S, Gao H, Bhore R, et al. REGN-COV2, a Neutralizing Antibody Cocktail, in Outpatients with Covid-19. New Engl J Med. 2021;384(3):238–251. doi: 10.1056/NEJMoa2035002 33332778 PMC7781102

[12] Monteil V, Dyczynski M, Lauschke VM, Kwon H, Wirnsberger G, Youhanna S, et al. Human soluble ACE2 improves the effect of remdesivir in SARS-CoV-2 infection. Embo Mol Med. 2021;13(1). doi: 10.15252/emmm.202013426 33179852 PMC7799356

[13] Wirnsberger G, Monteil V, Eaton B, Postnikova E, Murphy M, Braunsfeld B, et al. Clinical grade ACE2 as a universal agent to block SARS-CoV-2 variants. bioRxiv. 2021:2021.09.10.459744. doi: 10.1101/2021.09.10.459744 35781796 PMC9350269

[14] Monteil V, Stephanie D, Klingström J, Thålin C, Kellner MJ, Christ W, et al. Clinical grade ACE2 effectively inhibits SARS-CoV-2 Omicron infections. bioRxiv. 2021:2021.12.25.474113.

[15] Kuba K, Imai Y, Rao SA, Gao H, Guo F, Guan B, et al. A crucial role of angiotensin converting enzyme 2 (ACE2) in SARS coronavirus-induced lung injury. Nat Med. 2005;11(8):875–879. doi: 10.1038/nm1267 16007097 PMC7095783

[16] Imai Y, Kuba K, Rao S, Huan Y, Guo F, Guan B, et al. Angiotensin-converting enzyme 2 protects from severe acute lung failure. Nature. 2005;436(7047):112–116. doi: 10.1038/nature03712 16001071 PMC7094998

[17] Khan A, Benthin C, Zeno B, Albertson TE, Boyd J, Christie JD, et al. A pilot clinical trial of recombinant human angiotensin-converting enzyme 2 in acute respiratory distress syndrome. Crit Care. 2017;21. 28877748 10.1186/s13054-017-1823-x PMC5588692

[18] Sims JJ, Greig JA, Michalson KT, Lian SR, Martino RA, Meggersee R, et al. Intranasal gene therapy to prevent infection by SARS-CoV-2 variants. Plos Pathog. 2021;17(7). doi: 10.1371/journal.ppat.1009544 34265018 PMC8282039

[19] Monteil V, Kwon H, Prado P, Hagelkruys A, Wimmer RA, Stahl M, et al. Inhibition of SARS-CoV-2 Infections in Engineered Human Tissues Using Clinical-Grade Soluble Human ACE2. Cell. 2020;181(4):905–913. doi: 10.1016/j.cell.2020.04.004 32333836 PMC7181998

[20] Gawish R, Starkl P, Pimenov L, Hladik A, Lakovits K, Oberndorfer F, et al. ACE2 is the critical in vivo receptor for SARS-CoV-2 in a novel COVID-19 mouse model with TNF- and IFN gamma-driven immunopathology. Elife. 2022;11. 10.7554/eLife.74623 PMC8776253 35023830

[21] Baazim H, Antonio-Herrera L, Bergthaler A. The interplay of immunology and cachexia in infection and cancer. Nat Rev Immunol. 2021:309–321. doi: 10.1038/s41577-021-00624-w 34608281 PMC8489366

[22] DeGeorge JJ, Ahn CH, Andrews PA, Brower ME, Choi YS, Chun MY, et al. Considerations for toxicology studies of respiratory drug products. Regul Toxicol Pharm. 1997;25(2):189–193. doi: 10.1006/rtph.1997.1099 9185894

[23] Zoufaly A, Poglitsch M, Aberle JH. Human recombinant soluble ACE2 in severe COVID-19 (vol 8, pg 1154, 2020). Lancet Resp Med. 2020;8(11):E78-E. 10.1016/S2213-2600(20)30418-5 PMC7515587 33131609

