# Supplementary figures and images for: Development of an aerosol intervention for COVID-19 disease: Tolerability of soluble ACE2 (APN01) administered via nebulizer
Source: PLoS One. 2022 Jul 11;17(7):e0271066. doi: 10.1371/journal.pone.0271066 (PMC9273060; doi:10.1371/journal.pone.0271066)

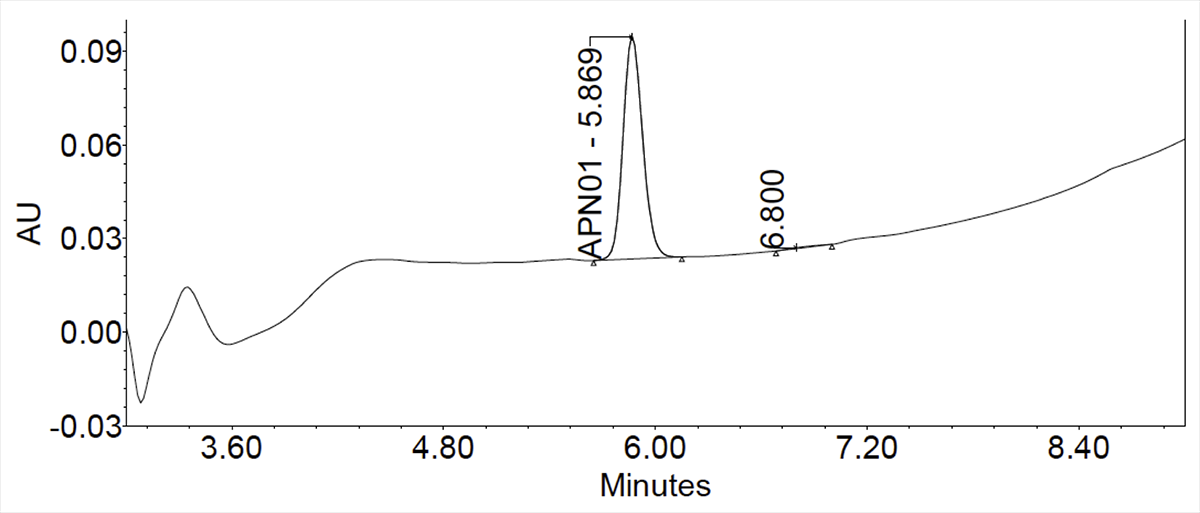

Supplement: S1 Fig — Absorbance Units (AU) monitored at 220 nm are plotted as a function of time. Chromatography conditions are described in Materials and Methods. APN01 was resolved as a single peak eluting between five and six minutes. The concentrations of test article in the processed filter samples were determined from each sample’s peak area using the linear regression parameters derived from the calibration curves and correcting the resulting concentration by multiplying by the appropriate dilution factor, as applicable. In addition to supporting quantitation of APN01 in the nebulized atmosphere, this result supports maintenance of the physical integrity of APN01 during the process of aerosolization. (TIF) [file pone.0271066.s001.tif]
