# Supplementary material for: Development of an aerosol intervention for COVID-19 disease: Tolerability of soluble ACE2 (APN01) administered via nebulizer
Source: PLoS One. 2022 Jul 11;17(7):e0271066. doi: 10.1371/journal.pone.0271066 (PMC9273060; doi:10.1371/journal.pone.0271066)
Supplement: S1 File — (ZIP) [file pone.0271066.s004.zip › S1 File.pdf]

Two-Week Aerosol Toxicity Study of APN01 in Dogs

Final Report

IITRI Project Number: **285700300102**

Contract Number: **75N91019D00013**
Task Order Number: **75N91020F00002**

Testing Facility:

IIT Research Institute (IITRI)
10 West 35th Street
Chicago, IL 60616

Study Director:

Jeffrey W. Richig, D.V.M.

Sponsor:

Chemopreventive Agent Development Research Group
Division of Cancer Prevention
National Cancer Institute (NCI)
9609 Medical Center Drive
Rockville, MD 20850

Contracting Officer's Representative (COR):

Robert Shoemaker, Ph.D.

Study Initiation Date: October 28, 2020

Study Completion Date: April 26, 2021

FOREWORD

Study Initiation:	October 28, 2020
Experimental Initiation:	November 2, 2020
In-Life Study Completion:	November 17, 2020

This report, entitled "Two-Week Aerosol Toxicity Study of APN01 in Dogs" (IITRI Project No. 285700300102), describes a study carried out by IIT Research Institute (IITRI) for the National Cancer Institute (Chemopreventive Agent Development Research Group; Division of Cancer Prevention). The Contracting Officer's Representative (COR) was Robert Shoemaker, Ph.D. Supervisory IITRI personnel were as follows:

Study Director:	Jeffrey W. Richig, D.V.M.
Principal Investigator/<br>President and Director:	David L. McCormick, Ph.D., D.A.B.T.
Veterinarian:	Kelly Garcia, D.V.M., Ph.D., D.A.C.L.A.M.
Analytical Chemistry Division Manager:<br>(*Dose Formulation, Serum Drug Level and Toxicokinetic Analyses*)	Miguel Muzzio, Ph.D., M.B.A.
Quality Assurance Unit Manager:	Glenn B. Miller, M.S., R.Q.A.P.-G.L.P., R.A.C.

Additional supervisory personnel were as follows:

Toxicologic Pathology:	Carol J. Detrisac, D.V.M., Ph.D., D.A.C.V.P.<br>Charles River Laboratories, Inc.<br>8025 Lamon Avenue, Suite 447<br>Skokie, IL 60077

Report Approval:

Jeffrey W. Richig — 2021-04-26

Jeffrey W. Richig, D.V.M.
Study Director

Date

David L. McCormick — 2021-04-26

David L. McCormick, Ph.D., D.A.B.T.
Principal Investigator
President and Director

Date

GLP COMPLIANCE STATEMENT

This study was conducted in accordance with U.S. Food and Drug Administration (FDA) Good Laboratory Practice (GLP) Regulations (Title 21, Part 58 of the *Code of Federal Regulations*) with the exception that the protocol was not approved by the Sponsor at the start of the study. The data have been reviewed, and the information contained in this report accurately reflects and is supported by the study raw data, representing an appropriate and accurate conclusion within the context of the study design and evaluation criteria.

Jeffrey W. Richig (signature)
Jeffrey W. Richig, D.V.M.
Study Director

2021-04-26
Date

QUALITY ASSURANCE STATEMENT

Study Title: Two-Week Aerosol Toxicity Study of APN01 in Dogs

Project Number: 285700300102

Study Director: Jeffrey W. Richig, D.V.M.

This study has been inspected and the report has been audited by the IITRI Quality Assurance Unit in accordance with U.S. Food and Drug Administration (FDA) Good Laboratory Practice Regulations as set forth in the *Code of Federal Regulations*, Title 21 Section 58.35. The report describes the methods and procedures used in the study and the reported results accurately reflect the raw data of the study. The following are the inspection dates, phases inspected, and the dates the inspection findings were reported:

		Findings Reported to:	
Date of Inspection	Phase	Study Director	Management
10/28/2020	Protocol review	10/28/2020	10/28/2020
11/2/2020	1st Exposure (Day 1), Exposure Atmosphere Sample Filter Collection and Respiration Measurements inspection	11/3/2020	11/3/2020
11/3/2020	Protocol Amendment #1 Review and TK Blood Collection, Blood Pressure & Pulse Oximetry Measurements inspection	11/4/2020	11/4/2020
11/10/2020	Inspect dose formulation (test atmosphere) analysis	11/10/2020	11/10/2020
11/12/2020	Inspect Functional Observational Battery	11/13/2020	11/13/2020
11/17/2020	Inspect Organ Weights and Necropsy	11/17/2020	11/17/2020
11/30/2020	Inspect serum analysis	11/30/2020	11/30/2020
12/18/2020	Review protocol amendment 2	12/18/2020	12/18/2020
12/30/2020 & 1/4/2021	Audit test atmosphere draft report (Appendix D) and data	1/4/2021	1/4/2021
1/14-15/2021	Audit serum analysis draft report (Appendix G) and data	1/15/2021	1/15/2021
1/20/2021	Audit toxicokinetic draft report (appendix H) and data	1/20/2021	1/20/2021
01/20-22, 25-29 & 02/1-3,26 & 03/1-4, 8-9/2021	Protocol Deviation #1, Data and Draft Report (excluding Appendices B-J)	3/10/2021	3/10/2021
01/21-22/2021	Audit clinical pathology report (Appendix F) and raw data	1/25/2021	1/25/2021
3/4 & 5/2021	Audit test atmosphere and exposure report tables	3/5/2021	3/5/2021
03/04-05/2021	Audit clin obs, physical exams, BW, OW report tables (Appendix C) and data	3/8/2021	3/8/2021

(*Continued on next page*)

QUALITY ASSURANCE STATEMENT

		Findings Reported to:	
Date of Inspection	Phase	Study Director	Management
03/04/2021	Audit ophthalmic examination, ECG evaluation reports, FOB, and associated raw data and report text (Appendices D, E, and I)	3/4/2021	3/4/2021
3/8/2021	Audit heart rate and oximetry report tables	3/8/2021	3/8/2021
3/16-18, 23-26/2021	Respiratory Function Data Audit	3/29/2021	3/29/2021
4/8/2021	Protocol deviation #2	4/8/2021	4/8/2021
04/23 & 26/2021	Check Data Corrections and Final Report Audit	4/26/2021	4/26/2021

Glenn B. Miller, M.S., R.Q.A.P.-G.L.P., R.A.C.
Assistant Vice President and Manager
Quality Assurance Unit

2021-04-26
Date

SUMMARY

APN01 is a recombinant soluble human angiotensin converting enzyme 2 (ACE2) being developed as an inhibitor of SARS-CoV-2 infection and its associated disease, COVID-19. This study was performed to provide a comprehensive evaluation of the toxicity of twice daily inhalation administration of APN01 aerosols to dogs for 14 consecutive days. Goals of this study included:

- Characterization of the toxicity of repeat-dose inhalation exposure to APN01 aerosols in dogs for fourteen days
- Identification of sensitive target tissues for the toxicity of inhaled APN01 in dogs
- Characterization of serum levels and toxicokinetics (TK) of inhaled APN01 in dogs
- Identification of a No Observed Adverse Effect Level [NO(A)EL] for twice daily inhalation administration of APN01 to dogs for fourteen consecutive days

The study design is summarized below:

Group	Number of Dogs (M + F)	Agent	Number and Duration of Daily Exposures	Number of Exposure Days	Target APN01 Concentration in Test Atmosphere (mg/L)
1	3 + 3	Saline (Control)	2 x 60 minutes	14	0
2	3 + 3	Vehicle (Control)	2 x 60 minutes	14	0
3	3 + 3	APN01 – Low	2 x 60 minutes	14	0.019
4	3 + 3	APN01 – Mid	2 x 60 minutes	14	0.038
5	3 + 3	APN01 - High	2 x 60 minutes	14	0.075

The target APN01 concentration of 0.075 mg/L used for the high dose group was demonstrated to be the maximum feasible concentration (MFC) in a preliminary range-finding study.

Toxicology endpoints included mortality/moribundity observations; clinical observations for signs of toxicity; physical examinations; heart rate and blood pressure measurements; body weight measurements; food consumption measurements; ophthalmic examinations; electrocardiographic evaluations; respiratory function evaluations; measurements of blood oxygen saturation and pH; neurotoxicity evaluations (functional observational battery [FOB]); clinical pathology assessments (clinical chemistry, hematology, coagulation, and urinalysis); quantitation of serum drug levels; limited modeling of serum toxicokinetics (TK); gross pathology at necropsy; organ weights; and microscopic evaluation of tissues.

Mean APN01 concentrations and particle size distributions in test atmospheres are provided in the table below. Particle size distributions in all groups met OECD requirements for repeat dose studies (MMAD < 2.0 µm, GSD of 1 to 3 µm) and were within the respirable size range for dogs. Test aerosol concentrations were consistent with concentration targets in all groups.

SUMMARY

Group	Treatment	APN01 Target Concentration	Sample Collection Time Point	Analytical Concentration (mg/L) [a]	Particle Size Distribution	
					MMAD [a] (µm)	GSD Range
1	Saline Control	0 (control)	AM	0.000 ± 0.0000	1.06 ± 0.113	2.25-2.36
			PM	0.000 ± 0.0000		
2	Vehicle Control	0 (control)	AM	0.000 ± 0.0000	1.36 ± 0.078	2.20-2.56
			PM	0.000 ± 0.0000		
3	APN01 – Low	0.019 mg/L	AM	0.017 ± 0.0014	1.57 ± 0.566	1.53-2.28
			PM	0.017 ± 0.0028		
4	APN01 – Mid	0.038 mg/L	AM	0.034 ± 0.0034	1.92 ± 0.205	1.80-2.41
			PM	0.035 ± 0.0024		
5	APN01 - High	0.075 mg/L	AM	0.078 ± 0.0062	2.00 ± 0.290	1.87-1.95
			PM	0.082 ± 0.0058		

[a] Mean ± Standard Deviation

No early deaths occurred during the study, and no gross clinical signs of toxicity were seen in any study animal. Inhalation administration of APN01 aerosols had no effects on body weight, food consumption, clinical pathology parameters, heart rate, blood pressure, electrocardiography, blood oxygen saturation, blood pH, FOB parameters, or ophthalmology. Respiratory function evaluations (respiratory rate, tidal volume and minute volume) were inconclusive due to excitement and/or panting exhibited by study animals during measurement periods. Organ weights were comparable in all study groups. No gross or microscopic pathology was linked to APN01 administration.

Systemic exposure [defined as serum levels of APN01 above the limit of quantitation (LOQ)] was very low in dogs in the low dose and mid dose groups on both Days 1 and 14; in both groups, serum levels of APN01 were below the LOQ (0.5 ng/mL) in most animals at most time points. In the high dose group, serum levels of APN01 on Days 1 and 14 were above the LOQ in 5 of 6 dogs at all time points after 0.5 hr. Although interanimal variability was substantial, mean C_{max} (pooled across both sexes) in the high dose group was approximately 8 ng/mL on both Day 1 and Day 14.

No evidence of systemic or organ-specific toxicity was identified in any dog receiving twice daily 60 minute exposures to APN01 aerosols at target concentrations of 0.019, 0.038, or 0.075 mg/L (approximately 0.6, 1.4, and 3.0 mg/kg/day, respectively) for fourteen consecutive days. On this basis, the No-Observed-Adverse-Effect Level [NO(A)EL] for twice daily one hour exposures to aerosolized APN01 for fourteen days is 0.075 mg/L (approximately 3.0 mg/kg/day).

TABLE OF CONTENTS

Page

FOREWORD 2

GLP COMPLIANCE STATEMENT 3

QUALITY ASSURANCE STATEMENT 4

SUMMARY 6

I. INTRODUCTION 11

II. MATERIALS AND METHODS 11

- A. Data Management 12
- B. Test and Control Articles and Vehicle 12
- C. Animals and Animal Maintenance 13
- D. Experimental Design 15
- E. Inhalation Exposure Methods 16
- F. Toxicology Methods 18
- G. Postmortem Procedures 21
- H. Statistical Procedures 22
- I. Archives 23

III. RESULTS 23

- A. Test Atmospheres 23
- B. Inhaled Dose 24
- C. Mortality and Clinical Signs 25
- D. Body Weights and Body Weight Changes 26
- E. Food Consumption 26
- F. Clinical Pathology 26
- G. Heart Rate and Blood Pressure 26
- H. Ophthalmic Examination 27
- I. Electrocardiography 27
- J. Respiratory Function 27
- K. Peripheral and Venous Blood Oxygen Saturation and Blood pH 28
- L. Functional Observational Battery 29
- M. Serum Drug Levels and Toxicokinetics 29
- N. Organ Weights 30
- O. Gross Pathology and Histopathology 30

IV. DISCUSSION AND CONCLUSION 30

(*Continued on next page*)

TABLE OF CONTENTS

Page

V. SUMMARY TABLES

Table 1. Summary of Test Atmosphere Concentration and Particle Size Data Tab-1
Table 2. Summary of Test Atmosphere Environmental Data Tab-2
Table 3. Summary of Fates Tab-3
Table 4a. Summary of Clinical Observation Frequency (vs. Group 1) Tab-4
Table 4b. Summary of Clinical Observation Frequency (vs. Group 2) Tab-5
Table 5a. Summary of Physical Examination Observation Frequency (vs. Group 1) ..Tab-6
Table 5b. Summary of Physical Examination Observation Frequency (vs. Group 2) ..Tab-7
Table 6a. Summary of Heart Rate and Blood Pressure Data (vs. Group 1)..................Tab-8
Table 6b. Summary of Heart Rate and Blood Pressure Data (vs. Group 2)................Tab-23
Table 7a. Summary of Body Weight Data (vs. Group 1)...Tab-38
Table 7b. Summary of Body Weight Data (vs. Group 2)...Tab-39
Table 8a. Summary of Body Weight Change Data (vs. Group 1)..............................Tab-40
Table 8b. Summary of Body Weight Change Data (vs. Group 2)..............................Tab-41
Table 9a. Summary of Respiratory Rate Data (vs. Group 1) Tab-42
Table 9b. Summary of Respiratory Rate Data (vs. Group 2) Tab-44
Table 10a. Summary of Tidal Volume Data (vs. Group 1)..Tab-46
Table 10b. Summary of Tidal Volume Data (vs. Group 2)..Tab-48
Table 11a. Summary of Minute Volume Data (vs. Group 1).......................................Tab-50
Table 11b. Summary of Minute Volume Data (vs. Group 2).......................................Tab-52
Table 12a. Summary of Peripheral Oxygen Saturation Data (vs. Group 1).................Tab-54
Table 12b. Summary of Peripheral Oxygen Saturation Data (vs. Group 2).................Tab-56
Table 13a. Summary of Venous Blood Oxygen Saturation Data (vs. Group 1) Tab-58
Table 13b. Summary of Venous Blood Oxygen Saturation Data (vs. Group 2) Tab-60
Table 14a. Summary of Blood pH Data (vs. Group 1) Tab-62
Table 14b. Summary of Blood pH Data (vs. Group 2) Tab-64
Table 15a. Summary of Absolute Organ Weight Data (vs. Group 1)...........................Tab-66
Table 15b. Summary of Absolute Organ Weight Data (vs. Group 2)...........................Tab-68
Table 16a. Summary of Relative Organ Weight (Organ-to-Body Weight Ratio) Data (vs. Group 1) Tab-70
Table 16b. Summary of Relative Organ Weight (Organ-to-Body Weight Ratio) Data (vs. Group 2) Tab-72

(*Continued on next page*)

TABLE OF CONTENTS

VI. APPENDICES *

Appendix A. Protocol, Protocol Amendments and Protocol Deviations

Appendix B. Test Atmosphere Analysis Report

Appendix C. Daily Exposure and Individual Animal Data

Appendix D. Ophthalmic Examination Report

Appendix E. Electrocardiographic Examination Report

Appendix F. Clinical Pathology Report

Appendix G. Serum Analysis and Toxicokinetic Report

Appendix H. Pathology Report

Appendix I. Functional Observational Battery Data

Appendix J. Abbreviations and Units of Measure; Urinalysis Key

Appendix K. Test Material Documentation

* *Detailed Tables of Contents for Appendices B, C and F-I are provided within the appendix.*

I. INTRODUCTION

A critical early step in cellular entry for SARS-CoV-2, the coronavirus responsible for COVID-19, is binding to angiotensin converting enzyme 2 (ACE2) as a cell surface receptor (Shang *et al*., PNAS 117, 11727-11734, 2020). Administration of exogenous ACE2 has been suggested as a pharmacologic approach to block SARS-CoV-2 infection. This idea is based on the hypothesis that exogenous soluble ACE2 provides an additional target for SARS-CoV-2 binding, and will thereby reduce viral binding to ACE2 on the cell surface and subsequent entry into the host cell.

APN01 is a recombinant soluble human ACE2 developed by Apeiron Biologics AG (Vienna, Austria). APN01 was developed to treat acute lung injury and pulmonary hypertension; the rationale for this approach is based on the protection conferred by administration of exogenous ACE2 in acute lung failure (Imai *et al*., Nature 436, 112-116, 2005). Phase 2 clinical trials of APN01 as a COVID-19 therapeutic are in progress in Europe, and are planned for expansion into the United States (Apeiron, https://www.apeiron-biologics.com/our-work-on-a-potential-drug-candidate-for-covid-19/, 2020).

Clinical data demonstrate that intravenous administration of recombinant human ACE2 is well-tolerated. The present Task Order is part of a preclinical development effort for inhaled APN01, with the goal of increasing APN01 delivery to the lung and thereby increasing its efficacy in reducing SARS-CoV-2 infection. The objective of the study was to provide data to support a comprehensive evaluation of the toxicity of twice daily aerosol administration of APN01 to dogs for 14 consecutive days. The goals of this study included:

- Characterization of the toxicity of inhaled APN01 aerosols in a suitable non-rodent species (dogs)
- Characterization of serum drug levels and toxicokinetics (TK) of inhaled APN01 in dogs
- Identification of sensitive target tissues for the toxicity of inhaled APN01 aerosols in dogs
- Identification of a No-Observed-Adverse-Effect Level [NO(A)EL] for twice daily inhalation administration of APN01 to dogs for fourteen days.

II. MATERIALS AND METHODS

The study protocol, protocol amendments and protocol deviations are included in Appendix A. A list of the abbreviations and units of measure used in this report and their definitions is provided in Appendix J.

A. Data Management: The ToxData® system for data acquisition, management, and reporting (version 3.0; PDS Pathology Data Systems; Basel, Switzerland) was used to collect and analyze in-life toxicology data. Excel® (Microsoft; Redmond, WA) was used to tabulate and calculate descriptive statistics for inhalation exposures, respiratory function evaluations, and quantitative data from functional observational battery (FOB) evaluations. Data for test atmosphere characterization (Appendix B) as well as data for serum drug level and toxicokinetic analyses (Appendix G) were collected and analyzed as described in Contributing Scientist Reports. The data management system used by the histopathology test site for necropsy and histopathology data is listed in Appendix H (Pathology Report).

   Standard of Exchange of Nonclinical Data (SEND) data sets were created using TranSEND (PDS Pathology Data Systems; Basel, Switzerland; version 1.2.2 or later).

B. Test and Control Articles and Vehicle: The test and control articles used in this study and the suppliers are listed below in Text Table 1. The test article was identified by the Sponsor as APN01. Test aerosols used in the study were based on a supplied formulation of APN01 (a clear liquid) from Apeiron Biologics. However, the formulation made by Polymun Scientific (the contract manufacturer for Apeiron Biologics) was not the true clinical formulation. Per the Sponsor, the supplied APN01 formulation contained exactly 1/6th of the intended PS80 concentration (due to an error during 6-fold dilution of DS→DP). The intended concentration was 0.02% (w/v) PS80. Therefore, as stipulated in the protocol, prior to use of the formulation to generate test aerosols for the study, 0.5 mL of PS80 (APN01 PS80 Concentrate, prepared by MRIGlobal) was added at IITRI using sterile technique to each vial (containing 4 mL) of APN01 (ACE2 Drug Product) supplied by Apeiron.

   Two control articles were used to generate test aerosols – sterile saline and APN01 Diluent (composition provided below).

   All quantities of test and control articles that were dispensed were documented. The lot/batch numbers, receipt dates, and storage conditions are provided in Text Table 1.

Text Table 1: Composition of Test and Control Articles

Material	Vendor/Manufacturer	Lot/Batch Number	Date Received at IITRI	Storage Conditions at IITRI
**TEST ARTICLE**				
APN01 (ACE2 Drug Product) [a]	Polymun Scientific; Klosterneuburg, Austria; supplied by Apeiron Biologics (Vienna, Austria)	ACE20620-B	October 8 and November 3, 2020	Approximately 2-8°C in original vials
APN01 PS80 Concentrate [b,c]	MRIGlobal (Kansas City, MO)	15145-29-2	October 9, 2020	Approximately 2-8°C
		15241-4-3	November 3, 2020	
**CONTROL ARTICLES**				
Saline (0.9% Sodium Chloride) [a]	Hospira (Rocky Mount, NC)	87-116-4B	May 9, 2018	Room Temperature
	Laboratorios Biogalenic (San Salvador, El Salvador)	1902033	May 10, 2019	
APN01 Diluent [b,d]	MRIGlobal (Kansas City, MO)	15145-29-1	October 9, 2020	Approximately 2-8°C
		15241-4-2	November 3, 2020	

[a] Certificate(s) of Analysis provided in Appendix K
[b] Statement of Product Composition provided in Appendix K
[c] Formulation: 10 mM sodium phosphate, 210 mM sucrose, 25 mM arginine HCl, 10 mM sodium chloride, 50 µM zinc chloride, 0.154% (w/w) polysorbate 80, in water
[d] Formulation: 10 mM sodium phosphate, 210 mM sucrose, 25 mM arginine HCl, 10 mM sodium chloride, 50 µM zinc chloride, 0.02% (w/w) polysorbate 80, in water

The vehicle was filtered, compressed air. Compressed air was generated using an oil-less compressor; prior to introduction into a test atmosphere, compressed air was filtered and purified using a disposable compressed air-activated carbon air filter (to remove aerosols) and a carbon absorber (to remove organic compounds).

C. Animals and Animal Maintenance

1. Receipt and Quarantine: Thirty naïve, purebred beagle dogs (15 per sex) were purchased from Ridglan Farms (Mt. Horeb, WI) for use in this study. Prior to shipment by the supplier, all dogs were immunized against distemper, type 2 adenovirus, parainfluenza, bordetella, rabies, papilloma virus and parvovirus. All dogs also received toltrazuril as a prophylactic treatment for coccidia. The dogs were received at IITRI on September 23, 2020.

   During the approximately five-week quarantine period, dogs were observed twice daily for mortality and/or evidence of moribundity. Prior to release from quarantine, each dog received a handheld physical examination to ensure its general health and

suitability for use as a test subject. Dogs were released from quarantine on October 29, 2020.

On the first day of APN01 administration, dogs were at least five months old. Male dogs weighed 8.04-10.02 kg, and female dogs weighed 6.12-9.12 kg.

2. Group Assignment and Identification: Except as noted in Protocol Deviation No. 1, study animals were assorted into five groups (identified in Section II.D) based on body weights. Group assignments were made using a computerized randomization procedure (ToxData®). Animals were assigned to groups on October 29, 2020.

   Each dog was identified by a USDA tattoo in the right or left ear and was also assigned a unique number within the study. All pens were identified by a cage card indicating IITRI project number, animal number, and sex.

3. Housing and Environment: Except during inhalation exposures, dogs were single-housed or double-housed by sex in floor level pens equipped with automatic watering systems. Pens were cleaned daily.

   The temperature and relative humidity (RH) in the room where the dogs were housed were recorded each day. Animal room environmental conditions were consistent with temperature (20-26°C) and relative humidity (30-70%) ranges identified in IITRI Standard Operating Procedures. A 12-hour light/dark cycle was maintained using an automatic timer.

4. Food and Water: Approximately 400 g of certified Teklad Global 21% Protein Dog Diet [#2021C; obtained from the Madison, WI, facility of Teklad Laboratory Animal Diets, Envigo (Indianapolis, IN)], was provided to each dog for a minimum of one hour daily. Each certified lot of diet was analyzed for contaminants to ensure that none are present at concentrations that would be expected to interfere with the conduct or purpose of this study. Analytical data from lots of diet used in this study are maintained with IITRI vivarium records.

   Coarse-filtered City of Chicago water was supplied *ad libitum* to all dogs using an automatic watering system. Water is analyzed periodically for evidence of bacterial contamination and chemical composition (e.g., electrolytes, metals, etc.). No contaminants are known to be present in the water at levels that would be expected

to interfere with the conduct or outcome of the study. Reports for the water analyses are maintained with IITRI vivarium records.

5. Restraint and Acclimation to Restraint: During inhalation exposures, each dog was restrained in an exposure (holding) sling. Once secured in the sling, an oronasal mask was positioned over the muzzle of the dog. Each mask was connected by a hose to the test article exposure manifold/plenum. Sling restraint and mask placement were performed to minimize animal stress. When restrained in slings, dogs were observed frequently and, if necessary, were repositioned in the sling to ensure exposure to test or control article and to prevent injury. Oronasal masks were cleaned daily.

   To condition study animals to placement and restraint in the oronasal exposure system and to reduce stress during inhalation exposures, dogs were acclimated to the sling and oronasal mask during a pre-treatment acclimation period. Each dog was acclimated for 10 minutes on Acclimation Day 1, 20 minutes on Acclimation Day 2, and 40 minutes on Acclimation Day 3.

6. Animal Welfare: This study was performed in full compliance with the Animal Welfare Act (AWA; Title 9, *Code of Federal Regulations*), the *Public Health Service Policy on Humane Care and Use of Laboratory Animals* (National Institutes of Health Office of Laboratory Animal Welfare, 2002/2015), and the *Guide for the Care and Use of Laboratory Animals* (8th Edition, National Research Council, 2011). To the greatest extent possible, procedures used in this study were designed to avoid or minimize discomfort, stress, and pain to animals.

D. Experimental Design: Dogs were exposed to aerosolized APN01 (at one of three dose levels) or a control article (saline or diluent) twice daily for 60 minutes per exposure for 14 consecutive days. The design is summarized in Text Table 2.

Text Table 2: Summary of Experimental Design

Group	Number of Dogs (M + F)	Agent	Number and Duration of Daily Exposures	Number of Exposure Days	Target APN01 Concentration in Test Atmosphere (mg/L)
1	3 + 3	Saline (Control)	2 x 60 minutes	14	0
2	3 + 3	Vehicle (Control)	2 x 60 minutes	14	0
3	3 + 3	APN01 – Low	2 x 60 minutes	14	0.019
4	3 + 3	APN01 – Mid	2 x 60 minutes	14	0.038
5	3 + 3	APN01 - High	2 x 60 minutes	14	0.075

The target concentration of APN01 in test atmospheres for the high dose group (0.075 mg/L) was determined as the maximum feasible concentration (MFC) in a range-finding toxicity study of APN01 (IITRI Project Number 285700300101). Inhalation administration of APN01 aerosols was initiated on November 2, 2020 (males) and November 3, 2020 (females).

E. Inhalation Exposure Methods

1. Inhalation Exposure System: Inhalation exposures were performed using custom 8-port exposure systems that are designed to supply uniform test atmospheres to all ports. During inhalation exposures, each dog was held in a sling and fitted with an oronasal mask connected to the exposure supply and exhaust manifolds.

2. Test Atmosphere Generation: Air for test atmospheres was generated using an oil-less compressor; prior to entry into a test atmosphere, compressed air was filtered using a compressed air filter (to remove aerosols) and a carbon absorber (to remove organic compounds). Test atmosphere inlet and exhaust configurations provided a uniform and continuous stream of fresh (non-recycled) test atmosphere to animals.

   Test atmospheres were generated by aerosolization of control or test substances using four Pari LC Plus nebulizers (Pari Respirator Equipment; Midlothian, VA). Test article formulations were delivered via syringe to the nebulizers. For each dose group, nebulizers were filled as follows:

   - Group 1 (Saline Control): All four nebulizers were filled with Saline
   - Group 2 (Vehicle Control): All four nebulizers were filled with APN01 Diluent
   - Group 3 (Low Dose): One nebulizer was filled with APN01 and the other three nebulizers were run dry, i.e., without APN01

- Group 4 (Mid Dose): Two nebulizers were filled with APN01 and the other two nebulizers were run dry
- Group 5 (High Dose): All four nebulizers were filled with APN01

Prior to the study, nebulizer airflow was calculated to be approximately 5.27 liters per minute (LPM; per nebulizer) when the operating pressure is set to 25 PSI.

The output of the nebulizers entered a four liter mixing plenum before being delivered to ports through respiratory tubing. Four ports in the eight-port system were connected to oronasal masks; the remaining ports were used for test atmosphere monitoring. Exhaust from each port was filtered using a high efficiency particulate air (HEPA) filter and then exhausted outside the building.

3. Test Atmosphere Monitoring

a. Aerosol Mass Concentration: For each exposure group, filter samples were collected from one port of the exposure system to determine aerosol mass concentration in test atmosphere. The filter train consisted of a pre-weighed, 47 mm, glass fiber filter in series with a dry-gas meter (to measure sample volume) connected to a constant-flow vacuum pump.

Aerosol mass concentration was monitored gravimetrically for each group at least once per exposure period. Filter samples were collected at a constant flow rate that was approximately equal to the port flow of the delivery tube, and the total volume of air sampled was measured using the dry-gas meter. The weight-to-volume ratio was determined to obtain aerosol mass concentration.

In addition, aerosol concentrations in the exposure system were monitored using an aerosol sensor. The sensor served as a real-time indicator of any short-term changes in aerosol concentration.

b. HPLC Analysis of Filter Samples: After collection, filter samples were transferred to the IITRI Analytical Chemistry Division for quantitation of APN01 by HPLC. The analytical method used is described in Appendix B.

c. Aerosol Particle Size Distribution: Aerosol particle size distribution was determined twice per group during the study by collecting size-segregated aerosol samples using a 10-stage quartz crystal microbalance (QCM) cascade impactor (California Measurements Inc.; Sierra Madre, CA). The aerosol output

from one port of the exposure system was connected to the QCM and was sampled at least once per inhalation exposure level. The mass median aerodynamic diameter (MMAD) and geometric standard deviation (GSD) of the test aerosol were calculated from the mass accumulated on each collection stage of the QCM by using a validated computer program (QCMSIZE) that was developed at IITRI.

d. Temperature, Relative Humidity and Nebulizer Pressure: Temperature and relative humidity in the inhalation exposure system were measured during each exposure using a handheld thermohygrometer [model 8721; Control Company/ Traceable® Products (distributed by Fisher Scientific; Waltham, MA)]. Nebulizer pressure was recorded once during each exposure.

F. Toxicology Methods

1. Mortality/Moribundity Observations: Except for one day during the study (Protocol Deviation No. 1), dogs were observed at least twice daily throughout the quarantine and exposure periods for mortality or evidence of moribundity and to assess their general health. Mortality/moribundity checks were separated by a minimum of four hours.

2. Physical Examinations and Clinical Observations: Detailed physical examinations were performed on all dogs pre-test and once per exposure day, following the first exposure of the day. Cage-side clinical observations were performed on all dogs daily during the exposure period (approximately one hour after the end of the first exposure of each day).

3. Body Weights: Dogs were weighed once pre-test and twice weekly during the exposure period. Body weight changes were calculated on the basis of body weight differences at each interval.

4. Food Consumption: Food consumption (per pen) was measured once pre-test and daily during the exposure period.

5. Ophthalmology: Indirect funduscopic ophthalmic examinations were performed on all dogs pretest and during Study Week 2. Examinations were performed by an IITRI veterinarian.

6. Electrocardiography (ECG): ECG tracings were obtained from all dogs pre-test and during Study Week 2 (approximately two hours after the first of the two daily exposures). ECG tracings were evaluated by an IITRI veterinary cardiologist. ECG evaluations included heart rate, rhythm, duration of the PR interval, QRS complex and QT interval.

7. Heart Rate and Blood Pressure: Heart rate and blood pressure (systolic, diastolic and mean arterial) measurements were collected from each dog pre-test and daily following the first exposure of each day. Measurements were collected using a SunTech® Vet20 blood pressure monitor (SunTech Medical, Morrisville, NC) connected to a tail cuff.

8. Respiratory Function: Respiratory rate and tidal volume were measured for each dog pre-test and once daily during the exposure period, except as noted in Protocol Deviation No. 2. Minute volume was calculated from the daily respiratory rate and tidal volume measurements.

9. Peripheral and Venous Blood Oxygen Saturation and Blood pH: Peripheral oxygen saturation (SpO_2) data were collected using a RAD-5® handheld pulse oximeter (Masimo; Irvine, CA). Measurements were collected from between the digits of the front right paw from each dog pre-test and immediately after the first daily exposure on Days 1, 4, 7, 10 and 14 (as noted in Protocol Deviation No. 1, no data were collected for female dogs on Day 14 due to instrument failure).

   Blood samples for measurement of venous blood oxygen saturation (SvO_2) and blood pH were collected from the jugular vein of each dog pre-test and after the first daily exposure on Days 1, 4, 7, 10 and 14. Blood samples were collected into tubes containing lithium heparin as an anticoagulant, and were analyzed using a Stat Profile Prime Plus® Analyzer (Nova Biomedical, Waltham, MA).

10. Functional Observational Battery: A functional observational battery (FOB) was performed on all dogs pre-test and during Study Week 2. The FOB included evaluations of body temperature, mental status, gait and posture, forelimb and hindlimb responses, neuromuscular function, and reflex responses. Details and the results of the FOB evaluations are provided in Appendix I (Functional Observational Battery Data).

11. Serum Drug Level Analysis and Toxicokinetics: On Days 1 and 14, timed blood samples (approximately 3 mL) for serum drug level analysis were collected from each dog exposed to an APN01 aerosol. Blood samples were collected from the cephalic or jugular vein of each dog at the following time points (see Protocol Deviation No. 1):

    - Prior to the first daily exposure (pretest)
    - 15 and 30 minutes and 1, 2, 4 and 6 hours (Day 1) or 15 and 30 minutes and 1, 2 and 4-5 hours (Day 14) after the first daily exposure
    - 24 hours after the first daily exposure (immediately prior to the first exposure on Day 2 or prior to necropsy on Day 15)

    Samples were collected into CAT Serum Sep Clot Activator tubes (Greiner Bio-One North America, Monroe, NC). Following collection, blood was centrifuged and serum was collected and stored frozen at approximately -70°C until analyzed.

    Serum samples were analyzed using a commercial ELISA kit for quantitative detection of human ACE2. For the saline control and vehicle control groups (Groups 1 and 2, respectively), ELISA assays were performed only on serum samples collected at 1 hour post-exposure (see Protocol Deviation No. 1).

    Serum drug concentration data from each animal exposed to an APN01 aerosol (Groups 3-5) were used to model TK parameters. Noncompartmental modeling was performed using Phoenix WinNonlin software (Version 8.1; Certara, Princeton, NJ). Estimated inhaled dose levels for each group were calculated based on APN01 test atmosphere concentration, exposure time, and group mean animal weight (by sex by study day), as shown in Section III.B.

    Detailed methods for serum drug level analysis and toxicokinetic analysis are provided in Appendix G (Serum Analysis and Toxicokinetic Report).

12. Clinical Pathology: Blood samples for clinical chemistry, hematology and coagulation determinations were collected from the jugular or cephalic vein of each dog during pre-test and prior to terminal necropsy on Study Day 15. Dogs were fasted overnight prior to blood collections for clinical pathology.

    Urine samples for urinalysis were collected from all dogs prior to exposure on Study Day 1 (pretest) and on Study Day 15. For pre-test collections, dogs were placed in cages overnight, and urine was collected from pans below the cages. For the Study

Day 15 urine collections, urine samples were collected directly from the urinary bladder at necropsy.

Clinical pathology parameters evaluated are listed in Appendix F (Clinical Pathology Report), along with the methods and instrumentation used for the analyses.

G. Postmortem Procedures

1. Necropsy and Tissues Preserved: After an overnight fast, all dogs were anesthetized with sodium pentobarbital on Study Day 15 and were then euthanized by exsanguination. All dogs received a complete necropsy with tissue collection. Complete necropsy consisted of examination of the external surface of the body; all orifices; and the cranial, thoracic, and peritoneal cavities and their contents.

   Tissues listed in Text Table 3 were collected and fixed in 10% neutral buffered formalin, with the exception of the eyes with optic nerves (fixed in Davidson's solution) and the testes and epididymides (fixed in modified Davidson's solution). Tissues marked with an asterisk were weighed at necropsy; paired organs were weighed together. Organ-to-body weight ratios were calculated using the fasted body weight for each dog.

Text Table 3: Tissues Collected at Scheduled Necropsy

Animal Identification [1]	Lymph node, mesenteric
Artery, aorta	Muscle, skeletal
Bone, femur (with epiphyseal plate of head)	Nerve, optic (paired)
Bone marrow (sternum)	Nerve, sciatic
Brain*	Ovary (paired) *
Cervix	Pancreas
Epididymis (paired) *	Skin, ventral abdomen
Esophagus	Small intestine, duodenum
Eye (paired)	Small intestine, ileum
Gallbladder	Small intestine, jejunum
Gland, adrenal (paired)*	Spinal cord, cervical
Gland, mammary	Spinal cord, lumbar
Gland, parathyroid (paired)	Spinal cord, thoracic
Gland, pituitary	Spleen *
Gland, prostate	Stomach
Gland, salivary	Testis (paired) *
Gland, thyroid	Thymus *
Heart *	Tongue
Kidney (paired) *	Tonsil (paired)
Large intestine, cecum	Trachea
Large intestine, colon	Ureter
Large intestine, rectum	Urinary bladder
Liver *	Uterus *
Lung	Vagina
Lymph node, bronchial	Gross lesions(if any)
Lymph node, mandibular	

[1] Ear with tattoo; collected, but not processed

2. Histopathology: The tissues and organs listed above (except ear with identification) from all study dogs were evaluated microscopically by a board-certified veterinary pathologist. Tissues were trimmed, processed by routine histologic methods, embedded in paraffin, cut at 5 µm, and stained with hematoxylin and eosin. Detailed histopathology methods are presented in Appendix H (Pathology Report).

H. Statistical Procedures: Descriptive statistics for data collected during the exposure period were calculated and analyzed using statistical tests embedded in the ToxData® system or SigmaPlot (version 13.0; Systat Software, Inc.; San Jose, CA). Incidence data (e.g., clinical signs, physical examinations) were evaluated using Chi-square analysis and/or Fisher's Exact test. For all other data that was normally distributed and of equal variance, statistical comparisons were performed by one-way analysis of variance (ANOVA), with post-hoc comparisons made versus Group 1 (Saline Control) and/or Group 2 (Vehicle Control) using Dunnett's test. If normality and/or equal variance failed for any specific data set, statistical comparisons were performed nonparametrically using Kruskal-Wallis ANOVA, with separate post-hoc comparisons made versus Group 1 and

Group 2 using Dunn's test. A minimum significance level of $p < 0.05$ was used for all statistical comparisons. Quantitative data analyzed included:

- Heart rate and blood pressure (systolic, diastolic and mean arterial blood pressure) data
- Body weight and body weight change data
- Blood oxygen saturation (SpO_2 and SvO_2) and pH data
- Clinical pathology [clinical chemistry, hematology (excluding erythrocyte morphology), coagulation and select urinalysis (refractive index, specific gravity, pH and volume) parameters]
- Respiratory function (respiratory rate, tidal volume and minute volume) data
- Body temperature data
- Absolute and relative organ weight data

I. Archives: All original data generated at IITRI, all study specimens, and a copy of the final report will be retained in the IITRI Archives for at least five years after submission of the signed final report. After that time, the Sponsor will be responsible for all costs associated with their continued storage in the IITRI Archives or for the shipment of these materials to a new storage facility. The IITRI Quality Assurance Unit will maintain a complete record of the disposition of all archival materials.

III. RESULTS

A. Test Atmospheres: Exposure data are summarized in Table 1 (test atmosphere concentration and particle size data) and Table 2 (test atmosphere environmental data). Daily test atmosphere concentration data are presented in Appendix C, Table C-1. Weekly test atmosphere particle size data is presented in Appendix C, Table C-2. Daily test atmosphere environmental (temperature, humidity and nebulizer pressure) data are presented in Appendix C, Table C-3.

1. Test Atmosphere APN01 Concentration and Particle Size: Test atmosphere concentration data and particle size distribution data are summarized in Text Table 4. Analytical data (obtained by HPLC analysis of filters) demonstrated that test aerosols consistently achieved target exposure concentrations. Particle size distribution data showed that the generated particles were within the respirable size range for dogs and met targets for both MMAD and GSD.

Text Table 4: Summary of Aerosol Concentration and Particle Size Distribution

Group (Treatment)	Sample Collection Time Point	Analytical Concentration (mg/L) [a,b,c]	Particle Size Distribution	
			MMAD [a,d] (µm)	GSD Range[d]
1 (Saline Control)	AM	0.000 ± 0.0000	1.06 ± 0.113	2.25-2.36
	PM	0.000 ± 0.0000		
2 (Vehicle Control)	AM	0.000 ± 0.0000	1.36 ± 0.078	2.20-2.56
	PM	0.000 ± 0.0000		
3 (APN01; 0.019 mg/L)	AM	0.017 ± 0.0014	1.57 ± 0.566	1.53-2.28
	PM	0.017 ± 0.0028		
4 (APN01; 0.038 mg/L)	AM	0.034 ± 0.0034	1.92 ± 0.205	1.80-2.41
	PM	0.035 ± 0.0024		
5 (APN01; 0.075 mg/L)	AM	0.078 ± 0.0062	2.00 ± 0.290	1.87-1.95
	PM	0.082 ± 0.0058		

[a] Mean ± standard deviation
[b] N = 15 (each study group was exposed for 14 days; however, due to staggered dosing for the two sexes, aerosol samples were collected over 15 days)
[c] Analytical concentration calculated based on HPLC analysis results (see Appendix B): mg/filter HPLC result/sample volume (L)
[d] N = 2 (one measurement per study week)

2. Temperature, Humidity and Nebulizer Pressure: Mean temperature of test atmospheres generated during the study were within the target range (20-26°C). Mean relative humidity values were at times above the target range (30-70% RH); however, higher mean relative humidity values for the test atmospheres were not unexpected, and resulted from the excess moisture that is inherent in administering test article using an aqueous solvent. Nebulizer pressures were consistently 25 PSI, indicating that sufficient airflow was achieved.

B. Inhaled Dose: Calculated inhaled APN01 dose levels for each group after a single exposure (Study Day 1) and after repeat-dose exposure (after the first exposure on Study Day 11) are provided in Text Table 5.

Text Table 5: Calculation of Inhaled APN01 Dose

Group (Treatment)	Sex	Test Atmosphere APN01 Concentration [a] (mg/L)		Minute Volume (L/min)		Mean Body Weight (kg)		Calculated Inhaled APN01 Dose (mg/kg/Exposure) [b]	
		Post Single Exposure[c]	Post Repeated Exposures[d]	Post Single Exposure[c]	Post Repeated Exposures[d]	Post Single Exposure[c]	Post Repeated Exposures[d]	Post Single Exposure[c]	Post Repeated Exposures[d]
1 (Saline Control)	M	0	0	3.0153	2.9915	9.24	9.15	0.0	0.0
	F	0	0	2.5442	2.5470	7.49	7.50	0.0	0.0
2 (Vehicle Control)	M	0	0	2.8906	2.9650	8.77	9.05	0.0	0.0
	F	0	0	2.4337	2.4864	7.09	7.28	0.0	0.0
3 (APN01; 0.019 mg/L)	M	0.019	0.017	2.8773	2.9332	8.72	8.93	0.38	0.34
	F	0.016	0.013	2.3585	2.3529	6.82	6.80	0.33	0.27
4 (APN01; 0.038 mg/L)	M	0.027	0.035	2.9199	2.8986	8.88	8.80	0.53	0.69
	F	0.026	0.035	2.3753	2.3725	6.88	6.87	0.54	0.73
5 (APN01; 0.075 mg/L)	M	0.068	0.078	2.9066	2.9119	8.83	8.85	1.34	1.54
	F	0.073	0.080	2.4947	2.5470	7.31	7.50	1.49	1.63

[a] Determined by HPLC analysis

[b] Inhaled dose calculated as: **Dose (mg/kg/day) = (C × MV × T) / BW**,
where C is the test atmosphere concentration (mg/L) per group (first of two daily exposures);
MV is the group mean minute volume for the appropriate sex;
calculated as (L/min) = $0.499 \times BW^{0.809}$ *;
T is the duration of exposure (60 minutes);
BW is the group mean body weight for the appropriate sex.
* Bide, RW, et al. *J. Appl. Toxicol.* **20**, 273–290 (2000)

[c] Study Day 1 after the first of two daily exposures.

[d] Study Day 11 after the first of two daily exposures (21 total exposures); data from this time point were used since this was the last available non-fasted body weight (twice weekly weighing schedule).

C. Mortality and Clinical Signs: Animal fates are summarized in Table 3. Clinical observation frequency is summarized Table 4a/b, and physical examination observation frequency is summarized in Table 5a/b [“a” table data were analyzed for statistical significance against Group 1 (Saline Control) and “b” table data were analyzed for statistical significance against Group 2 (Vehicle Control)]. Individual animal clinical observations and fates are presented in Appendix C, Table C-4, and individual animal detailed physical examination observations are presented in Appendix C, Table C-5.

No deaths occurred during this study. Clinical observations and physical examinations did not identify any evidence of test article toxicity; in test article-treated groups, clinical signs were limited to observations of a sore in two male dogs [one dog in Group 4 (APN01; 0.038 mg/L) and one dog in Group 5 (APN01; 0.075 mg/L)] and scab in two

male dogs [both in Group 5 (APN01; 0.075 mg/L)]. Sores resulted from the oronasal mask rubbing against the muzzle; scabs were from unrelated minor injuries.

D. Body Weights and Body Weight Changes: Body weight and body weight change data are summarized in Table 7a/b and Table 8a/b, respectively ["a" table data were analyzed for statistical significance against Group 1 (Saline Control) and "b" table data were analyzed for statistical significance against Group 2 (Vehicle Control)]. Individual animal data are presented in Appendix C, Table C-7 and Table C-8.

When compared to Group 1 (Saline Control) or Group 2 (Vehicle Control), no significant differences in group mean body weight or body weight gain were seen in either male or female dogs in any group exposed to APN01 (Groups 3-5).

E. Food Consumption: Food consumption data are presented in Appendix C, Table C-9.

No significant differences in food consumption were seen in males or females in the test article-treated groups (Groups 3-5) in comparison to dogs of the same sex in Group 1 (Saline Control) or Group 2 (Vehicle Control).

F. Clinical Pathology: Results of the clinical pathology analyses are detailed in Appendix F (Clinical Pathology Report).

No test article-related differences in clinical chemistry, hematology, coagulation or urinalysis parameters versus either Group 1 (Saline Control) or Group 2 (Vehicle Control) were seen in males or females in test article-treated groups (Groups 3-5).

G. Heart Rate and Blood Pressure: Heart rate and blood pressure (systolic, diastolic and mean arterial blood pressure) data are summarized in Table 6a/b ["a" table data were analyzed for statistical significance against Group 1 (Saline Control); "b" table data were analyzed for statistical significance against Group 2 (Vehicle Control)]. Individual animal heart rate and blood pressure data are presented in Appendix C, Table C-6.

Although occasional statistically significant differences in heart rate or blood pressure versus one control group were seen in individual groups exposed to APN01, no consistent patterns of APN01 effects on heart rate or blood pressure were seen in any group. Any significant differences were seen in an APN01-treated group versus one control group only, and not in the APN01-treated group in comparison to both control groups. Furthermore, the small number of statistically significant differences were not consistent between sexes, were not temporally consistent, and did not demonstrate dose-relatedness.

On this basis, the occasional differences identified in Table C-6 are interpreted as reflecting the large number of statistical comparisons being made, and do not suggest any effect of APN01 on heart rate or blood pressure.

A statistically significantly increased mean diastolic blood pressure in comparison to Group 2 (Vehicle Control) was seen in the Group 3 (APN01; 0.019 mg/L) males at Day 14.

H. Ophthalmic Examination: Results of ophthalmic examinations are presented in Appendix D (Ophthalmic Examination Report).

No test article-related ocular abnormalities were seen in any dog at the Study Week 2 examinations.

I. Electrocardiography: Results of the electrocardiographic examinations are presented in Appendix E (Electrocardiographic Evaluation Report).

No test article-related electrocardiographic findings were noted in any dog at the Study Week 2 examinations.

J. Respiratory Function: Respiratory function data are summarized in Table 9a/b (respiratory rate), Table 10a/b (tidal volume) and Table 11a/b (minute volume) [“a” table data were analyzed for statistical significance against Group 1 (Saline Control) and “b” table data were analyzed for statistical significance against Group 2 (Vehicle Control)]. Individual animal respiratory function data are presented in Appendix C, Table C-10.

Statistically significantly increased respiratory rates in comparison to Group 1 (Saline Control) were seen in Group 4 (APN01; 0.038 mg/L) males at Days 6, 7, 8, 9, and 14. Statistically significantly increased respiratory rates in comparison to Group 2 (Vehicle Control) were seen in Group 4 males at Days 7, 8, 9 and 14.

A statistically significantly decreased tidal volume in comparison to Group 1 (Saline Control) was seen in Group 4 males at Study Day 13. A statistically significantly increased tidal volume in comparison to Group 2 (Vehicle Control) was seen in Group 3 (APN01; 0.019 mg/L) males at Day 1.

Statistically significantly increased minute volumes in comparison to Group 1 (Saline Control) were seen in Group 3 (APN01; 0.019 mg/L) males at Day 5 and in Group 5 (APN01; 0.075 mg/L) females at Day 4. A statistically significantly increased minute volume in comparison to Group 2 (Vehicle Control) was seen in Group 3 males at Day 5.

The sporadic statistically significant differences in respiratory function seen on several days during the study are considered to be due to the excitement and/or panting that the dogs exhibited, rather than to any effects of APN01 on respiratory function. This interpretation is supported by the facts that no dose-response relationships for effects on any parameter were seen across APN01-treated groups, and/or the changes on specific study days were generally seen in only one sex.

K. Peripheral and Venous Blood Oxygen Saturation and Blood pH: Peripheral oxygen saturation (SpO_2), venous blood oxygen saturation (SvO_2), and blood pH data are summarized in Text Table 6 and in Table 12a/b, Table 13a/b and Table 14a/b, respectively ["a" table data were analyzed for statistical significance against Group 1 (Saline Control); "b" table data were analyzed for statistical significance against Group 2 (Vehicle Control)]. The corresponding individual animal data are presented in Appendix C, Table C-11 (SpO_2), Table C-12 (SvO_2) and Table C-13 (blood pH).

Text Table 6: Blood Oxygen Saturation and pH

Group (Treatment)	Sex	Group Mean Blood Oxygen Saturation [a]											
		Pre-test [b]		Day 1		Day 4		Day 7		Day 10		Day 14	
		SpO_2	SvO_2	SpO_2	SvO_2	SpO_2	SvO_2	SpO_2	SvO_2	SpO_2	SvO_2	SpO_2	SvO_2
Group 1 (Saline Control)	M	89	44.9	87	47.2	86	62.5	95	71.1	88	71.1	85	53.3
	F	84	49.6	93	56.0	81	98.2	80	107.9	76	61.8	—	84.6
Group 2 (Vehicle Control)	M	88	56.3	80	54.2	81	64.6	91	64.7	82	52.5	90	59.5
	F	89	49.8	80	81.1	86	68.2	90	77.9	92	59.6	—	117.9
Group 3 (APN01; 0.019 mg/L)	M	92	48.6	92	80.0*	97	55.6	89	48.0	96	59.1	92	53.2
	F	92	50.9	98	67.6	80	86.6	87	89.1	86	58.7	—	78.3
Group 4 (APN01; 0.38 mg/L)	M	88	47.3	86	44.2	88	49.2	93	67.6	86	50.8	83	131.9
	F	82	60.8	94	43.4	82	54.1	90	62.1	90	45.7	—	136.5
Group 5 (APN01; 0.75 mg/L)	M	89	55.1	83	47.2	84	55.9	87	52.6	75	64.6	84	56.3
	F	83	43.2	88	56.0	90	66.4	99	79.4	83	77.3	—	69.9

[a] Blood oxygen saturation determined using peripheral oxygen saturation (SpO_2; %) and venous blood oxygen saturation (SvO_2; mmHg)
[b] Pre-test data not analyzed for statistical significance

NOTES
[1] "—" = No data available due to instrument failure on Day 14 (females only)
[2] Results marked with an asterisk are (*) statistically significant ($p < 0.05$) in comparison to Group 1 (Saline) dogs of the same sex, see Table 12a (SpO_2) and Table 13a (SvO_2) for details.
[3] No results are statistically significant in comparison to Group 2 (vs. Group 2), see Table 12b (SpO_2) and Table 13b (SvO_2) for details.

The only statistically significant difference in peripheral or venous blood oxygen saturation was an increase in venous blood oxygen saturation for Group 3 (APN01;

0.019 mg/L) males at Day 1 in comparison to Group 1 (Saline Control). This difference was not considered test article-related due to its sporadic nature, presence in only a single sex, and lack of a dose-response relationship.

A statistically significant increase in blood pH in comparison to Group 2 (Vehicle Control) was seen in Group 5 (APN01; 0.075 mg/L) females at Day 14. This change was not considered test article-related due to only being seen in one sex. No other statistically significant differences in comparison to Group 1 (Saline Control) or Group 2 were seen in the males or females in any test article-treated group.

L. Functional Observational Battery: Functional Observational Battery (FOB) data are presented in Appendix I (Functional Observational Battery Data).

In FOBs performed during Study Week 2, no statistically significant differences in body temperature in APN01-treated groups were seen in comparison to either Group 1 (Saline Control) or Group 2 (Vehicle Control) dogs. Similarly, no significant differences in any other FOB parameters (mental status, gait and posture, hopping tests, placing response, patellar reflex, perineal reflex, flexor reflexes, menace reflex, pupillary reflex or righting reflex) were identified.

M. Serum Drug Levels and Toxicokinetics: Results of serum drug level analysis and associated toxicokinetics (TK) data are presented in Appendix G (Serum Analysis and Toxicokinetic Report).

Systemic exposure [defined as serum levels of APN01 above the limit of quantitation (LOQ)] was very low in dogs in the low dose and mid dose groups after both single dose exposure (after the first dose on Study Day 1) and after repeat-dose exposure (after the first dose on Study Day 14). In both groups on both days, serum levels of APN01 were below the LOQ (0.5 ng/mL) in most animals at most time points.

In the high dose group, serum levels of APN01 on Days 1 and 14 were above the LOQ in 5 of 6 dogs at all time points after 0.5 hr. Although interanimal variability was substantial, mean C_{max} (pooled across both sexes) in the high dose group was approximately 8 ng/mL on both Day 1 and Day 14.

Based on C_{max} and AUC, systemic exposure to APN01 was low after exposure to aerosols containing either the low or mid dose of APN01, but was higher after inhalation exposure to the high dose of APN01. Neither serum drug levels nor TK parameters in the high

dose group demonstrated any consistent pattern of differences between sexes or after repeat exposures versus a single exposure.

N. Organ Weights: Absolute and relative (organ-to-body weight ratio) organ weight data are summarized in Table 15a/b and Table 16a/b, respectively [“a” table data were analyzed for statistical significance against Group 1 (Saline Control) and “b” table data were analyzed for statistical significance against Group 2 (Vehicle Control)]. Individual animal absolute and relative organ weights are presented in Appendix C, Table C-14 and Table C-15, respectively.

No statistically significant differences in absolute or relative organ weights in comparison to Group 1 (Saline Control) or Group 2 (Vehicle Control) were seen in the males or females in any test article-treated group (Groups 3-5) at Study Day 15.

O. Gross Pathology and Histopathology: Gross pathology and histopathologic findings are detailed in Appendix H (Pathology Report).

No gross or microscopic changes were identified in any animal receiving twice daily inhalation exposure to APN01.

IV. DISCUSSION AND CONCLUSION

Twice daily inhalation administration (60 minutes per exposure) of APN01 at test atmosphere concentrations of 0.019, 0.038 or 0.075 mg/L to beagle dogs for 14 consecutive days induced no mortality, clinical signs of toxicity, effects on body weight or body weight change, effects on food consumption, or effects on clinical pathology (hematology, clinical chemistry, coagulation, urinalysis) parameters. Similarly, twice daily administration of APN01 by inhalation had no effects on heart rate, blood pressure, blood oxygen saturation, FOB parameters, ophthalmology, or electrocardiography. Respiratory function evaluations were inconclusive. No gross pathology attributable to APN01 exposure was identified at the terminal necropsy on Day 15, and organ weights were comparable in all study groups. No microscopic findings associated with APN01 administration were identified in any study animal.

No evidence of systemic or organ-specific toxicity was identified in any dog receiving twice daily 60 minute exposures to APN01 aerosols at target concentrations of 0.019, 0.038, or 0.075 mg/L (approximately 0.6, 1.4, and 3.0 mg/kg/day, respectively) for fourteen consecutive days. On this basis, the No-Observed-Adverse-Effect Level [NO(A)EL] for

twice daily one hour exposures to aerosolized APN01 for fourteen days is 0.075 mg/L (approximately 3.0 mg/kg/day).

V. SUMMARY TABLES

[NOTE: *In "a" tables, post hoc statistical comparisons were made against Group 1 (Saline Control). In "b" tables, post hoc statistical comparisons were made against Group 2 (Vehicle Control).*]

Table 1 – Summary of Test Atmosphere Concentration and Particle Size Data

Group (Treatment)	Sample Collection Time Point	Analytical Concentration (mg/L) [a,b,c]	Particle Size Distribution	
			MMAD [a,d] (µm)	GSD Range[d]
1 (Saline Control)	AM	0.000 ± 0.0000	1.06 ± 0.113	2.25-2.36
	PM	0.000 ± 0.0000		
2 (Vehicle Control)	AM	0.000 ± 0.0000	1.36 ± 0.078	2.20-2.56
	PM	0.000 ± 0.0000		
3 (APN01; 0.019 mg/L)	AM	0.017 ± 0.0014	1.57 ± 0.566	1.53-2.28
	PM	0.017 ± 0.0028		
4 (APN01; 0.038 mg/L)	AM	0.034 ± 0.0034	1.92 ± 0.205	1.80-2.41
	PM	0.035 ± 0.0024		
5 (APN01; 0.075 mg/L)	AM	0.078 ± 0.0062	2.00 ± 0.290	1.87-1.95
	PM	0.082 ± 0.0058		

[a] Mean ± standard deviation

[b] N = 15 (each study group was exposed for 14 days; however, due to staggered dosing for the two sexes, aerosol samples were collected over 15 days)

[c] Analytical concentration calculated based on HPLC analysis results (see Appendix B):
mg/filter HPLC result/sample volume (L)

[d] N = 2 (one measurement per study week)

Table 2 – Summary of Test Atmosphere Environmental Data
(Temperature, Humidity and Nebulizer Pressure)

AM

Group (Treatment)	Sample Collection Time Point	Mean Test Atmosphere Environmental Data [a]								
		Temperature Reading (°C) [a,b]			Relative Humidity Reading (%) [a,b]			Nebulizer Pressure Reading (PSI) [a, b, c]		
1 (Saline Control)	AM	21.0	±	0.30	69.1	±	8.79	25.0	±	0.00
2 (Vehicle Control)	AM	21.2	±	0.37	68.8	±	7.60	25.0	±	0.00
3 (APN01; 0.019 mg/L)	AM	21.0	±	0.54	47.1	±	12.29	25.0	±	0.00
4 (APN01; 0.038 mg/L)	AM	20.8	±	0.67	54.3	±	9.38	25.0	±	0.00
5 (APN01; 0.075 mg/L)	AM	20.4	±	0.84	86.4	±	4.45	25.0	±	0.00

[a] Mean ± standard deviation

[b] N = 30 (each study group was exposed for 14 days; however, due to staggered dosing for the two sexes, aerosol samples were collected over 15 days, with two readings per day)

[c] Corresponding nebulizer airflow was calculated to be approximately 5.27 LPM (per nebulizer)

PM

Group (Treatment)	Sample Collection Time Point	Mean Test Atmosphere Environmental Data [a]								
		Temperature Reading (°C) [a,b]			Relative Humidity Reading (%) [a,b]			Nebulizer Pressure Reading (PSI) [a, b, c]		
1 (Saline Control)	PM	21.1	±	0.41	72.6	±	8.38	25.0	±	0.00
2 (Vehicle Control)	PM	21.4	±	0.40	71.1	±	6.08	25.0	±	0.00
3 (APN01; 0.019 mg/L)	PM	21.2	±	0.87	45.1	±	7.24	25.0	±	0.00
4 (APN01; 0.038 mg/L)	PM	21.1	±	0.48	55.1	±	5.22	25.0	±	0.00
5 (APN01; 0.075 mg/L)	PM	20.7	±	0.84	82.9	±	12.11	25.0	±	0.00

[a] Mean ± standard deviation

[b] N = 30 (each study group was exposed for 14 days; however, due to staggered dosing for the two sexes, aerosol samples were collected over 15 days, with two readings per day)

[c] Corresponding nebulizer airflow was calculated to be approximately 5.27 LPM (per nebulizer)

Table 3 – Summary of Fates

MALES

			G 1 / M<br>Saline	G 2 / M<br>Vehicle	G 3 / M<br>Low<br>0.019 mg/L	G 4 / M<br>Mid<br>0.038 mg/L	G 5 / M<br>High<br>0.075 mg/L
day 1 to 15	Animals examined	N	3	3	3	3	3
	terminal sacrifice	N	3	3	3	3	3

FEMALES

			G 1 / F<br>Saline	G 2 / F<br>Vehicle	G 3 / F<br>Low<br>0.019 mg/L	G 4 / F<br>Mid<br>0.038 mg/L	G 5 / F<br>High<br>0.075 mg/L
day 1 to 15	Animals examined	N	3	3	3	3	3
	terminal sacrifice	N	3	3	3	3	3

Table 4a – Summary of Clinical Observation Frequency (vs. Group 1)

MALES

			G 1 / M Saline		G 2 / M Vehicle	G 3 / M Low 0.019 mg/L	G 4 / M Mid 0.038 mg/L	G 5 / M High 0.075 mg/L
day 1 to 14	Animals examined	N	3		3	3	3	3
	Animals with signs	N	0	cx	1	0	1	1
	normal *normal*	N	3	cx	3	3	3	3
	general	N	0	cx	1	0	1	1
	canine cherry eye	N	0	cx	1	0	0	0
	sore	N	0	cx	0	0	1	1

cx=CHI-SQUARE-EXACT

FEMALES

			G 1 / F Saline		G 2 / F Vehicle	G 3 / F Low 0.019 mg/L	G 4 / F Mid 0.038 mg/L	G 5 / F High 0.075 mg/L
day 1 to 14	Animals examined	N	3		3	3	3	3
	Animals with signs	N	0	cx	1	0	0	0
	normal *normal*	N	3	cx	3	3	3	3
	general *alopecia*	N	0	cx	1	0	0	0

cx=CHI-SQUARE-EXACT

NOTE: "Frequency" (table title) = number of animals for which the sign was observed at any point during the given study period.

Table 4b – Summary Clinical Observation Frequency (vs. Group 2)

MALES

			G 2 / M Vehicle		G 1 / M Saline	G 3 / M Low 0.019 mg/L	G 4 / M Mid 0.038 mg/L	G 5 / M High 0.075 mg/L
day 1 to 14	Animals examined	N	3		3	3	3	3
	Animals with signs	N	1	cx	0	0	1	1
	normal<br>*normal*	N	3	cx	3	3	3	3
	general	N	1	cx	0	0	1	1
	canine cherry eye	N	1	cx	0	0	0	0
	sore	N	0	cx	0	0	1	1

cx=CHI-SQUARE-EXACT

FEMALES

			G 2 / F Vehicle		G 1 / F Saline	G 3 / F Low 0.019 mg/L	G 4 / F Mid 0.038 mg/L	G 5 / F High 0.075 mg/L
day 1 to 14	Animals examined	N	3		3	3	3	3
	Animals with signs	N	1	cx	0	0	0	0
	normal<br>*normal*	N	3	cx	3	3	3	3
	general<br>*alopecia*	N	1	cx	0	0	0	0

cx=CHI-SQUARE-EXACT

NOTE: "Frequency" (table title) = number of animals for which the sign was observed at any point during the given study period.

Table 5a – Summary of Physical Examination Observation Frequency (vs. Group 1)

MALES

			G 1 / M Saline		G 2 / M Vehicle	G 3 / M Low 0.019 mg/L	G 4 / M Mid 0.038 mg/L	G 5 / M High 0.075 mg/L
day 1 to 15	Animals examined	N	3		3	3	3	3
	Animals with signs	N	0	cx	1	0	1	2
	general	N	0	cx	1	0	1	2
	sore	N	0	cx	0	0	1	1
	canine cherry eye	N	0	cx	1	0	0	0
	scab	N	0	cx	0	0	0	2
	normal *normal*	N	3	cx	2	3	3	3

cx=CHI-SQUARE-EXACT

FEMALES

			G 1 / F Saline		G 2 / F Vehicle	G 3 / F Low 0.019 mg/L	G 4 / F Mid 0.038 mg/L	G 5 / F High 0.075 mg/L
day 1 to 15	Animals examined	N	3		3	3	3	3
	Animals with signs	N	1	cx	1	0	0	0
	general	N	1	cx	1	0	0	0
	sore	N	1	cx	0	0	0	0
	alopecia	N	0	cx	1	0	0	0
	normal *normal*	N	3	cx	2	3	3	3

cx=CHI-SQUARE-EXACT

NOTE: “Frequency” (table title) = number of animals for which the sign was observed at any point during the given study period.

Table 5b – Summary of Physical Examination Observation Frequency (vs. Group 2)

MALES

			G 2 / M Vehicle		G 1 / M Saline	G 3 / M Low 0.019 mg/L	G 4 / M Mid 0.038 mg/L	G 5 / M High 0.075 mg/L
day 1 to 15	Animals examined	N	3		3	3	3	3
	Animals with signs	N	1	cx	0	0	1	2
	general	N	1	cx	0	0	1	2
	sore	N	0	cx	0	0	1	1
	canine cherry eye	N	1	cx	0	0	0	0
	scab	N	0	cx	0	0	0	2
	normal *normal*	N	2	cx	3	3	3	3

cx=CHI-SQUARE-EXACT

FEMALES

			G 2 / F Vehicle		G 1 / F Saline	G 3 / F Low 0.019 mg/L	G 4 / F Mid 0.038 mg/L	G 5 / F High 0.075 mg/L
day 1 to 15	Animals examined	N	3		3	3	3	3
	Animals with signs	N	1	cx	1	0	0	0
	general	N	1	cx	1	0	0	0
	sore	N	0	cx	1	0	0	0
	alopecia	N	1	cx	0	0	0	0
	normal *normal*	N	2	cx	3	3	3	3

cx=CHI-SQUARE-EXACT

Table 6a – Summary of Heart Rate and Blood Pressure Data (vs. Group 1)

PRE-TEST (MALES)

		G 1 / M Saline	G 2 / M Vehicle	G 3 / M Low 0.019 mg/L	G 4 / M Mid 0.038 mg/L	G 5 / M High 0.075 mg/L
Heart Rate	Mean	129 a	138	128	106	128
[Beats/Minute]	S.D.	12.1	17.5	21.6	22.0	23.5
day -4	N	3	3	3	3	3
	P-Value	@0.4304				
Diastolic Blood Pressure	Mean	86 k	99	81	73	81
[mmHg]	S.D.	5.5	17.4	32.6	14.4	24.5
day -4	N	3	3	3	3	3
	P-Value	@0.6647				
Mean Arterial Pressure	Mean	101 a	121	99	96	97
[mmHg]	S.D.	6.0	9.7	29.1	12.6	21.8
day -4	N	3	3	3	3	3
	P-Value	@0.4679				
Systolic Blood Pressure	Mean	143 k	174	152	163	160
[mmHg]	S.D.	7.0	1.7	25.7	9.2	24.2
day -4	N	3	3	3	3	3
	P-Value	@0.3251				

a=ANOVA; k=KRUSKAL-WALLIS

PRE-TEST (FEMALES)

		G 1 / F Saline	G 2 / F Vehicle	G 3 / F Low 0.019 mg/L	G 4 / F Mid 0.038 mg/L	G 5 / F High 0.075 mg/L
Heart Rate	Mean	147 a	128	109	162	138
[Beats/Minute]	S.D.	7.6	24.9	30.4	3.8	41.0
day -5	N	3	3	3	3	3
	P-Value	@0.2047				
Diastolic Blood Pressure	Mean	97 a	69	98	113	103
[mmHg]	S.D.	3.5	15.9	18.2	26.5	25.2
day -5	N	3	3	3	3	3
	P-Value	@0.1532				
Mean Arterial Pressure	Mean	112 a	78	111	123	115
[mmHg]	S.D.	4.0	10.7	18.5	27.7	24.4
day -5	N	3	3	3	3	3
	P-Value	@0.1162				
Systolic Blood Pressure	Mean	168 a	125	157	168	157
[mmHg]	S.D.	26.9	30.0	12.4	22.4	26.0
day -5	N	3	3	3	3	3
	P-Value	@0.2479				

a=ANOVA

Table 6a – Summary of Heart Rate and Blood Pressure Data (vs. Group 1)

DAY 1 (MALES)

		G 1 / M Saline	G 2 / M Vehicle	G 3 / M Low 0.019 mg/L	G 4 / M Mid 0.038 mg/L	G 5 / M High 0.075 mg/L
Heart Rate	Mean	123 k	146	133	118	122
[Beats/Minute]	S.D.	27.9	18.6	12.9	13.7	24.0
day 1	N	3	3	3	3	3
	P-Value	@0.4105				
Diastolic Blood Pressure	Mean	81 k	105	79	74	104
[mmHg]	S.D.	27.8	14.2	14.6	17.3	26.1
day 1	N	3	3	3	3	3
	P-Value	@0.2259				
Systolic Blood Pressure	Mean	144 a	161	132	138	156
[mmHg]	S.D.	43.7	17.0	38.8	43.9	58.6
day 1	N	3	3	3	3	3
	P-Value	@0.9060				
Mean Arterial Pressure	Mean	95 k	118	92	87	114
[mmHg]	S.D.	25.6	13.1	20.8	23.1	33.2
day 1	N	3	3	3	3	3
	P-Value	@0.2612				

k=KRUSKAL-WALLIS; a=ANOVA

DAY 1 (FEMALES)

		G 1 / F Saline	G 2 / F Vehicle	G 3 / F Low 0.019 mg/L	G 4 / F Mid 0.038 mg/L	G 5 / F High 0.075 mg/L
Heart Rate	Mean	139 k	136	105	148	133
[Beats/Minute]	S.D.	5.7	13.3	21.7	6.8	30.0
day 1	N	3	3	3	3	3
	P-Value	@0.1810				
Diastolic Blood Pressure	Mean	93 k	82	77	99	80
[mmHg]	S.D.	10.4	19.9	21.4	14.6	10.7
day 1	N	3	3	3	3	3
	P-Value	@0.4811				
Systolic Blood Pressure	Mean	151 d	145	113	164	136
[mmHg]	S.D.	6.1	4.0	28.9	18.2	14.1
day 1	N	3	3	3	3	3
	P-Value	@0.0396	0.9760	0.0619	0.7685	0.6462
Mean Arterial Pressure	Mean	106 a	98	85	116	92
[mmHg]	S.D.	9.8	14.4	22.5	16.4	10.8
day 1	N	3	3	3	3	3
	P-Value	@0.2006				

k=KRUSKAL-WALLIS; d=ANOVA-DUNNETT; a=ANOVA

Table 6a – Summary of Heart Rate and Blood Pressure Data (vs. Group 1)

DAY 2 (MALES)

		G 1 / M Saline	G 2 / M Vehicle	G 3 / M Low 0.019 mg/L	G 4 / M Mid 0.038 mg/L	G 5 / M High 0.075 mg/L
Heart Rate	Mean	102 a	134	129	116	121
[Beats/Minute]	S.D.	38.2	15.0	27.1	11.1	26.5
day 2	N	3	3	3	3	3
	P-Value	@0.5991				
Diastolic Blood Pressure	Mean	87 k	48	81	65	102
[mmHg]	S.D.	32.1	5.5	21.4	21.9	25.9
day 2	N	3	3	3	3	3
	P-Value	@0.1141				
Systolic Blood Pressure	Mean	152 k	95	132	142	156
[mmHg]	S.D.	11.7	47.8	50.1	11.0	21.4
day 2	N	3	3	3	3	3
	P-Value	@0.3702				
Mean Arterial Pressure	Mean	97 k	60	92	85	112
[mmHg]	S.D.	24.8	16.8	25.4	14.5	25.0
day 2	N	3	3	3	3	3
	P-Value	@0.1861				

a=ANOVA; k=KRUSKAL-WALLIS

DAY 2 (FEMALES)

		G 1 / F Saline	G 2 / F Vehicle	G 3 / F Low 0.019 mg/L	G 4 / F Mid 0.038 mg/L	G 5 / F High 0.075 mg/L
Heart Rate	Mean	158 a	137	138	153	155
[Beats/Minute]	S.D.	6.2	12.5	25.5	7.5	5.0
day 2	N	3	3	3	3	3
	P-Value	@0.2283				
Diastolic Blood Pressure	Mean	80 a	78	80	64	89
[mmHg]	S.D.	2.1	5.5	37.6	14.7	41.0
day 2	N	3	3	3	3	3
	P-Value	@0.8337				
Systolic Blood Pressure	Mean	134 k	132	124	134	150
[mmHg]	S.D.	4.0	10.6	41.2	22.2	33.7
day 2	N	3	3	3	3	3
	P-Value	@0.9195				
Mean Arterial Pressure	Mean	93 a	91	90	84	102
[mmHg]	S.D.	2.5	9.0	35.5	17.8	41.9
day 2	N	3	3	3	3	3
	P-Value	@0.9391				

a=ANOVA; k=KRUSKAL-WALLIS

Table 6a – Summary of Heart Rate and Blood Pressure Data (vs. Group 1)

DAY 3 (MALES)

		G 1 / M Saline	G 2 / M Vehicle	G 3 / M Low 0.019 mg/L	G 4 / M Mid 0.038 mg/L	G 5 / M High 0.075 mg/L
Heart Rate	Mean	129 a	138	129	117	146
[Beats/Minute]	S.D.	24.1	12.7	30.9	19.0	21.8
day 3	N	3	3	3	3	3
	P-Value	@0.6083				
Diastolic Blood Pressure	Mean	80 k	68	71	70	107
[mmHg]	S.D.	1.7	2.1	20.6	13.1	48.8
day 3	N	3	3	3	3	3
	P-Value	@0.7145				
Systolic Blood Pressure	Mean	126 a	132	97	155	141
[mmHg]	S.D.	26.9	35.3	24.9	9.9	63.6
day 3	N	3	3	3	3	3
	P-Value	@0.4350				
Mean Arterial Pressure	Mean	91 a	85	77	88	113
[mmHg]	S.D.	5.3	11.0	20.9	8.5	51.5
day 3	N	3	3	3	3	3
	P-Value	@0.5284				

a=ANOVA; k=KRUSKAL-WALLIS

DAY 3 (FEMALES)

		G 1 / F Saline	G 2 / F Vehicle	G 3 / F Low 0.019 mg/L	G 4 / F Mid 0.038 mg/L	G 5 / F High 0.075 mg/L
Heart Rate	Mean	148 a	127	129	154	142
[Beats/Minute]	S.D.	13.7	27.0	28.3	8.3	13.2
day 3	N	3	3	3	3	3
	P-Value	@0.4346				
Diastolic Blood Pressure	Mean	99 k	50	98	101	93
[mmHg]	S.D.	30.3	1.2	24.2	14.7	26.9
day 3	N	3	3	3	3	3
	P-Value	@0.1234				
Systolic Blood Pressure	Mean	143 k	123	142	150	151
[mmHg]	S.D.	3.5	49.6	23.3	12.6	23.5
day 3	N	3	3	3	3	3
	P-Value	@0.9552				
Mean Arterial Pressure	Mean	106 a	70	106	111	107
[mmHg]	S.D.	27.1	13.3	23.9	15.4	24.3
day 3	N	3	3	3	3	3
	P-Value	@0.1876				

a=ANOVA; k=KRUSKAL-WALLIS

Table 6a – Summary of Heart Rate and Blood Pressure Data (vs. Group 1)

DAY 4 (MALES)

		G 1 / M Saline	G 2 / M Vehicle	G 3 / M Low 0.019 mg/L	G 4 / M Mid 0.038 mg/L	G 5 / M High 0.075 mg/L
Heart Rate	Mean	127 a	122	155	102	134
[Beats/Minute]	S.D.	6.8	17.7	30.4	22.3	26.6
day 4	N	3	3	3	3	3
	P-Value	@0.1330				
Diastolic Blood Pressure	Mean	69 k	53	66	88	90
[mmHg]	S.D.	13.7	7.0	48.2	21.9	38.0
day 4	N	3	3	3	3	3
	P-Value	@0.3039				
Systolic Blood Pressure	Mean	127 a	102	149	124	141
[mmHg]	S.D.	25.7	36.3	11.2	35.0	63.7
day 4	N	3	3	3	3	3
	P-Value	@0.6310				
Mean Arterial Pressure	Mean	80 a	66	109	96	98
[mmHg]	S.D.	16.7	7.8	16.2	25.4	42.6
day 4	N	3	3	3	3	3
	P-Value	@0.3192				

a=ANOVA; k=KRUSKAL-WALLIS

DAY 4 (FEMALES)

		G 1 / F Saline	G 2 / F Vehicle	G 3 / F Low 0.019 mg/L	G 4 / F Mid 0.038 mg/L	G 5 / F High 0.075 mg/L
Heart Rate	Mean	149 a	144	139	153	140
[Beats/Minute]	S.D.	19.7	27.4	21.2	3.8	39.9
day 4	N	3	3	3	3	3
	P-Value	@0.9454				
Diastolic Blood Pressure	Mean	126 k	82	76	96	76
[mmHg]	S.D.	18.9	0.6	18.6	22.9	12.5
day 4	N	3	3	3	3	3
	P-Value	@0.1350				
Systolic Blood Pressure	Mean	178 k	150	147	181	126
[mmHg]	S.D.	13.4	15.6	26.5	33.2	38.4
day 4	N	3	3	3	3	3
	P-Value	@0.2066				
Mean Arterial Pressure	Mean	136 a	96	89	115	90
[mmHg]	S.D.	15.7	9.6	22.2	21.9	22.3
day 4	N	3	3	3	3	3
	P-Value	@0.0551				

a=ANOVA; k=KRUSKAL-WALLIS

Table 6a – Summary of Heart Rate and Blood Pressure Data (vs. Group 1)

DAY 5 (MALES)

		G 1 / M Saline	G 2 / M Vehicle	G 3 / M Low 0.019 mg/L	G 4 / M Mid 0.038 mg/L	G 5 / M High 0.075 mg/L
Heart Rate	Mean	138 k	161	144	138	133
[Beats/Minute]	S.D.	20.6	20.4	20.5	13.3	41.2
day 5	N	3	3	3	3	3
	P-Value	@0.7820				
Diastolic Blood Pressure	Mean	63 k	97	130	85	93
[mmHg]	S.D.	19.1	28.0	53.5	21.5	19.5
day 5	N	3	3	3	3	3
	P-Value	@0.3862				
Systolic Blood Pressure	Mean	112 a	157	170	119	150
[mmHg]	S.D.	23.6	22.1	45.6	32.7	1.5
day 5	N	3	3	3	3	3
	P-Value	@0.1406				
Mean Arterial Pressure	Mean	72 a	109	140	90	107
[mmHg]	S.D.	22.5	24.9	54.0	21.0	14.0
day 5	N	3	3	3	3	3
	P-Value	@0.1648				

k=KRUSKAL-WALLIS; a=ANOVA

DAY 5 (FEMALES)

		G 1 / F Saline	G 2 / F Vehicle	G 3 / F Low 0.019 mg/L	G 4 / F Mid 0.038 mg/L	G 5 / F High 0.075 mg/L
Heart Rate	Mean	150 a	149	144	133	140
[Beats/Minute]	S.D.	8.1	9.1	4.7	9.5	8.4
day 5	N	3	3	3	3	3
	P-Value	@0.1459				
Diastolic Blood Pressure	Mean	86 a	77	94	65	67
[mmHg]	S.D.	9.1	22.4	12.5	33.5	17.3
day 5	N	3	3	3	3	3
	P-Value	@0.4441				
Systolic Blood Pressure	Mean	145 a	123	151	119	136
[mmHg]	S.D.	18.3	45.0	9.5	46.7	23.5
day 5	N	3	3	3	3	3
	P-Value	@0.7020				
Mean Arterial Pressure	Mean	99 k	82	111	77	78
[mmHg]	S.D.	16.2	23.4	11.7	35.9	18.1
day 5	N	3	3	3	3	3
	P-Value	@0.3309				

a=ANOVA; k=KRUSKAL-WALLIS

Table 6a – Summary of Heart Rate and Blood Pressure Data (vs. Group 1)

DAY 6 (MALES)

		G 1 / M Saline	G 2 / M Vehicle	G 3 / M Low 0.019 mg/L	G 4 / M Mid 0.038 mg/L	G 5 / M High 0.075 mg/L
Heart Rate	Mean	142 a	134	132	138	143
[Beats/Minute]	S.D.	18.2	46.0	30.8	9.0	12.5
day 6	N	3	3	3	3	3
	P-Value	@0.9827				
Diastolic Blood Pressure	Mean	71 a	120	88	66	95
[mmHg]	S.D.	24.5	17.9	20.1	30.0	1.5
day 6	N	3	3	3	3	3
	P-Value	@0.0673				
Systolic Blood Pressure	Mean	129 d	162 *	147	162 *	143
[mmHg]	S.D.	11.3	10.6	7.2	18.3	9.0
day 6	N	3	3	3	3	3
	P-Value	@0.0321	0.0231	0.2556	0.0207	0.4280
Mean Arterial Pressure	Mean	80 d	131 *	101	92	106
[mmHg]	S.D.	21.5	14.7	17.4	17.8	4.2
day 6	N	3	3	3	3	3
	P-Value	@0.0334	0.0115	0.3969	0.8070	0.2177

a=ANOVA; d=ANOVA-DUNNETT; * = p < 0.05

DAY 6 (FEMALES)

		G 1 / F Saline	G 2 / F Vehicle	G 3 / F Low 0.019 mg/L	G 4 / F Mid 0.038 mg/L	G 5 / F High 0.075 mg/L
Heart Rate	Mean	159 k	123	139	151	166
[Beats/Minute]	S.D.	15.0	39.6	17.7	4.0	16.8
day 6	N	3	3	3	3	3
	P-Value	@0.3075				
Diastolic Blood Pressure	Mean	77 a	70	105	120	111
[mmHg]	S.D.	20.3	23.4	32.3	28.3	32.6
day 6	N	3	3	3	3	3
	P-Value	@0.1660				
Systolic Blood Pressure	Mean	165 a	118	148	171	173
[mmHg]	S.D.	10.0	44.9	29.5	31.5	36.7
day 6	N	3	3	3	3	3
	P-Value	@0.2761				
Mean Arterial Pressure	Mean	93 k	82	114	129	121
[mmHg]	S.D.	21.5	31.2	31.9	27.9	28.6
day 6	N	3	3	3	3	3
	P-Value	@0.3270				

k=KRUSKAL-WALLIS; a=ANOVA

Table 6a – Summary of Heart Rate and Blood Pressure Data (vs. Group 1)

DAY 7 (MALES)

		G 1 / M Saline	G 2 / M Vehicle	G 3 / M Low 0.019 mg/L	G 4 / M Mid 0.038 mg/L	G 5 / M High 0.075 mg/L
Heart Rate	Mean	127 k	157	148	115	123
[Beats/Minute]	S.D.	15.8	5.0	9.3	27.6	13.3
day 7	N	3	3	3	3	3
	P-Value	@0.0760				
Diastolic Blood Pressure	Mean	67 a	69	82	62	94
[mmHg]	S.D.	12.5	30.8	7.5	31.7	0.0
day 7	N	3	3	3	3	3
	P-Value	@0.3836				
Systolic Blood Pressure	Mean	135 k	140	179	114	150
[mmHg]	S.D.	18.5	20.4	4.6	60.5	3.5
day 7	N	3	3	3	3	3
	P-Value	@0.1234				
Mean Arterial Pressure	Mean	82 k	83	114	74	107
[mmHg]	S.D.	12.0	23.7	5.7	39.4	0.6
day 7	N	3	3	3	3	3
	P-Value	@0.0620				

k=KRUSKAL-WALLIS; a=ANOVA

DAY 7 (FEMALES)

		G 1 / F Saline	G 2 / F Vehicle	G 3 / F Low 0.019 mg/L	G 4 / F Mid 0.038 mg/L	G 5 / F High 0.075 mg/L
Heart Rate	Mean	155 a	148	129	156	147
[Beats/Minute]	S.D.	7.0	5.9	13.0	3.8	21.1
day 7	N	3	3	3	3	3
	P-Value	@0.1146				
Diastolic Blood Pressure	Mean	89 k	75	102	101	96
[mmHg]	S.D.	23.9	17.4	28.9	1.7	4.9
day 7	N	3	3	3	3	3
	P-Value	@0.3232				
Systolic Blood Pressure	Mean	155 k	121	158	141	157
[mmHg]	S.D.	19.9	7.5	23.4	7.5	4.4
day 7	N	3	3	3	3	3
	P-Value	@0.0790				
Mean Arterial Pressure	Mean	105 a	85	113	116	110
[mmHg]	S.D.	19.2	16.5	28.8	9.0	9.6
day 7	N	3	3	3	3	3
	P-Value	@0.2981				

a=ANOVA; k=KRUSKAL-WALLIS

Table 6a – Summary of Heart Rate and Blood Pressure Data (vs. Group 1)

DAY 8 (MALES)

		G 1 / M Saline	G 2 / M Vehicle	G 3 / M Low 0.019 mg/L	G 4 / M Mid 0.038 mg/L	G 5 / M High 0.075 mg/L
Heart Rate	Mean	124 a	150	142	132	142
[Beats/Minute]	S.D.	8.1	6.7	18.7	11.2	33.9
day 8	N	3	3	3	3	3
	P-Value	@0.5325				
Diastolic Blood Pressure	Mean	88 a	72	101	81	84
[mmHg]	S.D.	14.6	39.4	7.8	26.9	25.9
day 8	N	3	3	3	3	3
	P-Value	@0.7142				
Systolic Blood Pressure	Mean	148 a	118	153	150	129
[mmHg]	S.D.	20.1	53.6	10.7	11.2	48.6
day 8	N	3	3	3	3	3
	P-Value	@0.6626				
Mean Arterial Pressure	Mean	98 a	78	113	93	92
[mmHg]	S.D.	11.2	39.1	9.6	24.1	28.5
day 8	N	3	3	3	3	3
	P-Value	@0.5708				

a=ANOVA

DAY 8 (FEMALES)

		G 1 / F Saline	G 2 / F Vehicle	G 3 / F Low 0.019 mg/L	G 4 / F Mid 0.038 mg/L	G 5 / F High 0.075 mg/L
Heart Rate	Mean	165 d	146	117 *	149	149
[Beats/Minute]	S.D.	3.8	9.1	20.5	16.5	21.0
day 8	N	3	3	3	3	3
	P-Value	@0.0417	0.4524	0.0124	0.5768	0.5768
Diastolic Blood Pressure	Mean	85 k	78	84	92	74
[mmHg]	S.D.	13.3	3.2	2.9	41.3	20.0
day 8	N	3	3	3	3	3
	P-Value	@0.7780				
Systolic Blood Pressure	Mean	150 k	135	157	142	149
[mmHg]	S.D.	6.5	8.9	3.5	28.6	5.2
day 8	N	3	3	3	3	3
	P-Value	@0.1959				
Mean Arterial Pressure	Mean	103 a	93	94	100	91
[mmHg]	S.D.	6.7	4.7	6.0	41.6	14.9
day 8	N	3	3	3	3	3
	P-Value	@0.9404				

d=ANOVA-DUNNETT; * = $p < 0.05$; k=KRUSKAL-WALLIS; a=ANOVA

Table 6a – Summary of Heart Rate and Blood Pressure Data (vs. Group 1)

DAY 9 (MALES)

		G 1 / M Saline	G 2 / M Vehicle	G 3 / M Low 0.019 mg/L	G 4 / M Mid 0.038 mg/L	G 5 / M High 0.075 mg/L
Heart Rate	Mean	139 k	155	140	139	123
[Beats/Minute]	S.D.	10.4	14.2	19.6	23.1	8.5
day 9	N	3	3	3	3	3
	P-Value	@0.2879				
Diastolic Blood Pressure	Mean	95 k	87	94	94	66
[mmHg]	S.D.	30.3	22.7	19.1	2.1	19.0
day 9	N	3	3	3	3	3
	P-Value	@0.4189				
Systolic Blood Pressure	Mean	127 a	138	155	147	137
[mmHg]	S.D.	2.5	23.4	16.9	9.3	25.3
day 9	N	3	3	3	3	3
	P-Value	@0.4294				
Mean Arterial Pressure	Mean	87 a	98	103	104	86
[mmHg]	S.D.	5.1	23.5	19.1	2.5	20.6
day 9	N	3	3	3	3	3
	P-Value	@0.5139				

k=KRUSKAL-WALLIS; a=ANOVA

DAY 9 (FEMALES)

		G 1 / F Saline	G 2 / F Vehicle	G 3 / F Low 0.019 mg/L	G 4 / F Mid 0.038 mg/L	G 5 / F High 0.075 mg/L
Heart Rate	Mean	152 a	138	140	144	140
[Beats/Minute]	S.D.	5.1	15.1	37.1	15.5	9.8
day 9	N	3	3	3	3	3
	P-Value	@0.9152				
Diastolic Blood Pressure	Mean	81 k	93	89	82	96
[mmHg]	S.D.	11.1	0.6	19.2	23.9	16.2
day 9	N	3	3	3	3	3
	P-Value	@0.6535				
Systolic Blood Pressure	Mean	138 a	133	159	155	158
[mmHg]	S.D.	5.9	1.0	29.5	11.2	10.0
day 9	N	3	3	3	3	3
	P-Value	@0.1741				
Mean Arterial Pressure	Mean	97 k	101	105	102	108
[mmHg]	S.D.	15.0	1.2	25.2	19.3	15.5
day 9	N	3	3	3	3	3
	P-Value	@0.8183				

a=ANOVA; k=KRUSKAL-WALLIS

Table 6a – Summary of Heart Rate and Blood Pressure Data (vs. Group 1)

DAY 10 (MALES)

		G 1 / M Saline	G 2 / M Vehicle	G 3 / M Low 0.019 mg/L	G 4 / M Mid 0.038 mg/L	G 5 / M High 0.075 mg/L
Heart Rate [Beats/Minute] day 10	Mean	124 d	148	166 *	128	137
	S.D.	13.5	13.5	19.1	7.2	18.1
	N	3	3	3	3	3
	P-Value	@0.0380	0.2233	0.0210	0.9956	0.6754
Diastolic Blood Pressure [mmHg] day 10	Mean	100 k	98	109	98	82
	S.D.	38.4	11.0	16.2	40.7	29.1
	N	3	3	3	3	3
	P-Value	@0.7476				
Systolic Blood Pressure [mmHg] day 10	Mean	148 a	132	164	144	136
	S.D.	26.1	5.5	15.7	53.4	58.4
	N	3	3	3	3	3
	P-Value	@0.8505				
Mean Arterial Pressure [mmHg] day 10	Mean	110 a	105	122	107	92
	S.D.	38.0	10.1	13.7	43.0	32.9
	N	3	3	3	3	3
	P-Value	@0.8171				

d=ANOVA-DUNNETT; * = p < 0.05; k=KRUSKAL-WALLIS; a=ANOVA

DAY 10 (FEMALES)

		G 1 / F Saline	G 2 / F Vehicle	G 3 / F Low 0.019 mg/L	G 4 / F Mid 0.038 mg/L	G 5 / F High 0.075 mg/L
Heart Rate [Beats/Minute] day 10	Mean	135 a	153	140	156	172
	S.D.	31.5	25.1	16.8	20.6	18.7
	N	3	3	3	3	3
	P-Value	@0.3754				
Diastolic Blood Pressure [mmHg] day 10	Mean	87 a	94	105	101	101
	S.D.	13.0	17.9	15.5	9.9	24.1
	N	3	3	3	3	3
	P-Value	@0.7044				
Systolic Blood Pressure [mmHg] day 10	Mean	134 a	160	149	168	158
	S.D.	27.8	17.3	9.0	14.6	20.5
	N	3	3	3	3	3
	P-Value	@0.3072				
Mean Arterial Pressure [mmHg] day 10	Mean	97 a	107	114	118	113
	S.D.	15.4	14.0	14.4	3.1	24.6
	N	3	3	3	3	3
	P-Value	@0.5404				

a=ANOVA

Table 6a – Summary of Heart Rate and Blood Pressure Data (vs. Group 1)

DAY 11 (MALES)

		G 1 / M Saline	G 2 / M Vehicle	G 3 / M Low 0.019 mg/L	G 4 / M Mid 0.038 mg/L	G 5 / M High 0.075 mg/L
Heart Rate	Mean	113 a	161	125	125	125
[Beats/Minute]	S.D.	29.8	18.0	32.5	11.8	15.3
day 11	N	3	3	3	3	3
	P-Value	@0.1881				
Diastolic Blood Pressure	Mean	67 a	80	108	111	94
[mmHg]	S.D.	25.1	11.0	39.1	10.8	4.6
day 11	N	3	3	3	3	3
	P-Value	@0.1481				
Systolic Blood Pressure	Mean	116 a	132	157	169	162
[mmHg]	S.D.	10.3	22.1	36.4	14.0	5.6
day 11	N	3	3	3	3	3
	P-Value	@0.0508				
Mean Arterial Pressure	Mean	79 a	89	116	124	108
[mmHg]	S.D.	17.6	10.7	35.9	3.2	9.0
day 11	N	3	3	3	3	3
	P-Value	@0.0752				

a=ANOVA

DAY 11 (FEMALES)

		G 1 / F Saline	G 2 / F Vehicle	G 3 / F Low 0.019 mg/L	G 4 / F Mid 0.038 mg/L	G 5 / F High 0.075 mg/L
Heart Rate	Mean	129 a	165	152	143	141
[Beats/Minute]	S.D.	41.7	14.6	20.1	12.5	12.2
day 11	N	3	3	3	3	3
	P-Value	@0.4365				
Diastolic Blood Pressure	Mean	100 k	87	79	85	96
[mmHg]	S.D.	32.7	19.1	40.4	30.6	8.0
day 11	N	3	3	3	3	3
	P-Value	@0.9255				
Systolic Blood Pressure	Mean	153 a	150	161	173	155
[mmHg]	S.D.	15.9	5.1	17.9	2.1	9.5
day 11	N	3	3	3	3	3
	P-Value	@0.2014				
Mean Arterial Pressure	Mean	113 k	100	100	104	104
[mmHg]	S.D.	30.1	14.4	31.2	21.0	11.1
day 11	N	3	3	3	3	3
	P-Value	@0.9850				

a=ANOVA; k=KRUSKAL-WALLIS

Table 6a – Summary of Heart Rate and Blood Pressure Data (vs. Group 1)

DAY 12 (MALES)

		G 1 / M Saline	G 2 / M Vehicle	G 3 / M Low 0.019 mg/L	G 4 / M Mid 0.038 mg/L	G 5 / M High 0.075 mg/L
Heart Rate	Mean	133 k	157	150	144	135
[Beats/Minute]	S.D.	16.5	4.0	23.7	17.2	14.4
day 12	N	3	3	3	3	3
	P-Value	@0.3780				
Diastolic Blood Pressure	Mean	80 a	76	92	93	78
[mmHg]	S.D.	3.6	2.1	1.5	30.6	14.2
day 12	N	3	3	3	3	3
	P-Value	@0.5376				
Systolic Blood Pressure	Mean	130 d	141	158 **	150 *	162 **
[mmHg]	S.D.	11.6	2.1	2.0	13.1	5.6
day 12	N	3	3	3	3	3
	P-Value	@0.0048	0.3076	0.0062	0.0384	0.0025
Mean Arterial Pressure	Mean	90 a	91	107	104	100
[mmHg]	S.D.	6.6	3.6	4.2	35.3	11.4
day 12	N	3	3	3	3	3
	P-Value	@0.6523				

k=KRUSKAL-WALLIS; a=ANOVA; d=ANOVA-DUNNETT; ** = $p < 0.01$; * = $p < 0.05$

DAY 12 (FEMALES)

		G 1 / F Saline	G 2 / F Vehicle	G 3 / F Low 0.019 mg/L	G 4 / F Mid 0.038 mg/L	G 5 / F High 0.075 mg/L
Heart Rate	Mean	147 a	146	138	161	153
[Beats/Minute]	S.D.	13.6	28.6	15.0	29.0	12.9
day 12	N	3	3	3	3	3
	P-Value	@0.7391				
Diastolic Blood Pressure	Mean	83 a	73	90	78	97
[mmHg]	S.D.	2.1	20.3	6.8	30.7	10.0
day 12	N	3	3	3	3	3
	P-Value	@0.4774				
Systolic Blood Pressure	Mean	137 a	133	144	164	149
[mmHg]	S.D.	11.0	9.5	12.2	42.1	3.0
day 12	N	3	3	3	3	3
	P-Value	@0.4301				
Mean Arterial Pressure	Mean	90 a	88	100	100	109
[mmHg]	S.D.	7.0	16.8	7.4	34.1	8.5
day 12	N	3	3	3	3	3
	P-Value	@0.6288				

a=ANOVA

Table 6a – Summary of Heart Rate and Blood Pressure Data (vs. Group 1)

DAY 13 (MALES)

		G 1 / M Saline	G 2 / M Vehicle	G 3 / M Low 0.019 mg/L	G 4 / M Mid 0.038 mg/L	G 5 / M High 0.075 mg/L
Heart Rate	Mean	117 a	156	150	133	130
[Beats/Minute]	S.D.	5.7	6.4	14.0	37.3	17.2
day 13	N	3	3	3	3	3
	P-Value	@0.1861				
Diastolic Blood Pressure	Mean	72 k	84	89	84	74
[mmHg]	S.D.	2.6	20.8	0.6	46.5	19.4
day 13	N	3	3	3	3	3
	P-Value	@0.4569				
Systolic Blood Pressure	Mean	116 k	130	165	125	143
[mmHg]	S.D.	3.8	11.8	12.1	70.2	6.6
day 13	N	3	3	3	3	3
	P-Value	@0.0843				
Mean Arterial Pressure	Mean	81 k	96	105	88	86
[mmHg]	S.D.	1.5	18.9	8.4	47.9	20.6
day 13	N	3	3	3	3	3
	P-Value	@0.5523				

a=ANOVA; k=KRUSKAL-WALLIS

DAY 13 (FEMALES)

		G 1 / F Saline	G 2 / F Vehicle	G 3 / F Low 0.019 mg/L	G 4 / F Mid 0.038 mg/L	G 5 / F High 0.075 mg/L
Heart Rate	Mean	148 k	139	131	144	150
[Beats/Minute]	S.D.	19.1	23.8	29.7	11.2	6.9
day 13	N	3	3	3	3	3
	P-Value	@0.8301				
Diastolic Blood Pressure	Mean	89 k	94	84	75	90
[mmHg]	S.D.	5.0	0.6	21.4	21.2	11.6
day 13	N	3	3	3	3	3
	P-Value	@0.4387				
Systolic Blood Pressure	Mean	141 a	141	138	135	146
[mmHg]	S.D.	34.6	3.8	8.6	23.6	2.0
day 13	N	3	3	3	3	3
	P-Value	@0.9645				
Mean Arterial Pressure	Mean	102 a	103	94	85	101
[mmHg]	S.D.	13.6	2.1	17.6	19.2	7.2
day 13	N	3	3	3	3	3
	P-Value	@0.4598				

k=KRUSKAL-WALLIS; a=ANOVA

Table 6a – Summary of Heart Rate and Blood Pressure Data (vs. Group 1)

DAY 14 (MALES)

		G 1 / M Saline	G 2 / M Vehicle	G 3 / M Low 0.019 mg/L	G 4 / M Mid 0.038 mg/L	G 5 / M High 0.075 mg/L
Heart Rate	Mean	111 a	144	140	149	122
[Beats/Minute]	S.D.	31.7	12.8	8.5	15.3	15.8
day 14	N	3	3	3	3	3
	P-Value	@0.1337				
Diastolic Blood Pressure	Mean	69 u	63	133	101	91
[mmHg]	S.D.	26.4	15.6	32.0	6.4	6.2
day 14	N	3	3	3	3	3
	P-Value	@0.0320	1.0000	0.0701	0.7409	1.0000
Systolic Blood Pressure	Mean	114 k	128	175	148	157
[mmHg]	S.D.	39.5	56.6	21.7	9.3	7.0
day 14	N	3	3	3	3	3
	P-Value	@0.0947				
Mean Arterial Pressure	Mean	81 d	79	141 *	113	110
[mmHg]	S.D.	22.0	26.5	33.9	3.5	6.1
day 14	N	3	3	3	3	3
	P-Value	@0.0324	0.9999	0.0237	0.2929	0.3691

a=ANOVA; u=KRUSKAL-WALLIS-DUNN; k=KRUSKAL-WALLIS; d=ANOVA-DUNNETT; * = p < 0.05

DAY 14 (FEMALES)

		G 1 / F Saline	G 2 / F Vehicle	G 3 / F Low 0.019 mg/L	G 4 / F Mid 0.038 mg/L	G 5 / F High 0.075 mg/L
Heart Rate	Mean	154 a	140	135	147	147
[Beats/Minute]	S.D.	20.0	14.7	17.6	17.9	18.7
day 14	N	3	3	3	3	3
	P-Value	@0.7537				
Diastolic Blood Pressure	Mean	96 k	88	90	83	81
[mmHg]	S.D.	20.2	4.0	34.5	18.7	5.5
day 14	N	3	3	3	3	3
	P-Value	@0.8759				
Systolic Blood Pressure	Mean	141 a	132	146	151	147
[mmHg]	S.D.	9.0	6.2	15.7	9.9	2.5
day 14	N	3	3	3	3	3
	P-Value	@0.2358				
Mean Arterial Pressure	Mean	107 a	100	105	101	97
[mmHg]	S.D.	18.2	7.2	30.0	11.8	5.5
day 14	N	3	3	3	3	3
	P-Value	@0.9570				

a=ANOVA; k=KRUSKAL-WALLIS

Table 6b – Summary of Heart Rate and Blood Pressure Data (vs. Group 2)

PRE-TEST (MALES)

		G 2 / M Vehicle	G 1 / M Saline	G 3 / M Low 0.019 mg/L	G 4 / M Mid 0.038 mg/L	G 5 / M High 0.075 mg/L
Heart Rate	Mean	138 a	129	128	106	128
[Beats/Minute]	S.D.	17.5	12.1	21.6	22.0	23.5
day -4	N	3	3	3	3	3
	P-Value	@0.4304				
Diastolic Blood Pressure	Mean	99 k	86	81	73	81
[mmHg]	S.D.	17.4	5.5	32.6	14.4	24.5
day -4	N	3	3	3	3	3
	P-Value	@0.6647				
Mean Arterial Pressure	Mean	121 a	101	99	96	97
[mmHg]	S.D.	9.7	6.0	29.1	12.6	21.8
day -4	N	3	3	3	3	3
	P-Value	@0.4679				
Systolic Blood Pressure	Mean	174 k	143	152	163	160
[mmHg]	S.D.	1.7	7.0	25.7	9.2	24.2
day -4	N	3	3	3	3	3
	P-Value	@0.3251				

a=ANOVA; k=KRUSKAL-WALLIS

PRE-TEST (FEMALES)

		G 2 / F Vehicle	G 1 / F Saline	G 3 / F Low 0.019 mg/L	G 4 / F Mid 0.038 mg/L	G 5 / F High 0.075 mg/L
Heart Rate	Mean	128 a	147	109	162	138
[Beats/Minute]	S.D.	24.9	7.6	30.4	3.8	41.0
day -5	N	3	3	3	3	3
	P-Value	@0.2047				
Diastolic Blood Pressure	Mean	69 a	97	98	113	103
[mmHg]	S.D.	15.9	3.5	18.2	26.5	25.2
day -5	N	3	3	3	3	3
	P-Value	@0.1532				
Mean Arterial Pressure	Mean	78 a	112	111	123	115
[mmHg]	S.D.	10.7	4.0	18.5	27.7	24.4
day -5	N	3	3	3	3	3
	P-Value	@0.1162				
Systolic Blood Pressure	Mean	125 a	168	157	168	157
[mmHg]	S.D.	30.0	26.9	12.4	22.4	26.0
day -5	N	3	3	3	3	3
	P-Value	@0.2479				

a=ANOVA

Table 6b – Summary of Heart Rate and Blood Pressure Data (vs. Group 2)

DAY 1 (MALES)

		G 2 / M Vehicle	G 1 / M Saline	G 3 / M Low 0.019 mg/L	G 4 / M Mid 0.038 mg/L	G 5 / M High 0.075 mg/L
Heart Rate	Mean	146 k	123	133	118	122
[Beats/Minute]	S.D.	18.6	27.9	12.9	13.7	24.0
day 1	N	3	3	3	3	3
	P-Value	@0.4105				
Diastolic Blood Pressure	Mean	105 k	81	79	74	104
[mmHg]	S.D.	14.2	27.8	14.6	17.3	26.1
day 1	N	3	3	3	3	3
	P-Value	@0.2259				
Systolic Blood Pressure	Mean	161 a	144	132	138	156
[mmHg]	S.D.	17.0	43.7	38.8	43.9	58.6
day 1	N	3	3	3	3	3
	P-Value	@0.9060				
Mean Arterial Pressure	Mean	118 k	95	92	87	114
[mmHg]	S.D.	13.1	25.6	20.8	23.1	33.2
day 1	N	3	3	3	3	3
	P-Value	@0.2612				

k=KRUSKAL-WALLIS; a=ANOVA

DAY 1 (FEMALES)

		G 2 / F Vehicle	G 1 / F Saline	G 3 / F Low 0.019 mg/L	G 4 / F Mid 0.038 mg/L	G 5 / F High 0.075 mg/L
Heart Rate	Mean	136 k	139	105	148	133
[Beats/Minute]	S.D.	13.3	5.7	21.7	6.8	30.0
day 1	N	3	3	3	3	3
	P-Value	@0.1810				
Diastolic Blood Pressure	Mean	82 k	93	77	99	80
[mmHg]	S.D.	19.9	10.4	21.4	14.6	10.7
day 1	N	3	3	3	3	3
	P-Value	@0.4811				
Systolic Blood Pressure	Mean	145 d	151	113	164	136
[mmHg]	S.D.	4.0	6.1	28.9	18.2	14.1
day 1	N	3	3	3	3	3
	P-Value	@0.0396	0.9760	0.1247	0.4958	0.8986
Mean Arterial Pressure	Mean	98 a	106	85	116	92
[mmHg]	S.D.	14.4	9.8	22.5	16.4	10.8
day 1	N	3	3	3	3	3
	P-Value	@0.2006				

k=KRUSKAL-WALLIS; d=ANOVA-DUNNETT; a=ANOVA

Table 6b – Summary of Heart Rate and Blood Pressure Data (vs. Group 2)

DAY 2 (MALES)

		G 2 / M Vehicle	G 1 / M Saline	G 3 / M Low 0.019 mg/L	G 4 / M Mid 0.038 mg/L	G 5 / M High 0.075 mg/L
Heart Rate	Mean	134 a	102	129	116	121
[Beats/Minute]	S.D.	15.0	38.2	27.1	11.1	26.5
day 2	N	3	3	3	3	3
	P-Value	@0.5991				
Diastolic Blood Pressure	Mean	48 k	87	81	65	102
[mmHg]	S.D.	5.5	32.1	21.4	21.9	25.9
day 2	N	3	3	3	3	3
	P-Value	@0.1141				
Systolic Blood Pressure	Mean	95 k	152	132	142	156
[mmHg]	S.D.	47.8	11.7	50.1	11.0	21.4
day 2	N	3	3	3	3	3
	P-Value	@0.3702				
Mean Arterial Pressure	Mean	60 k	97	92	85	112
[mmHg]	S.D.	16.8	24.8	25.4	14.5	25.0
day 2	N	3	3	3	3	3
	P-Value	@0.1861				

a=ANOVA; k=KRUSKAL-WALLIS

DAY 2 (FEMALES)

		G 2 / F Vehicle	G 1 / F Saline	G 3 / F Low 0.019 mg/L	G 4 / F Mid 0.038 mg/L	G 5 / F High 0.075 mg/L
Heart Rate	Mean	137 a	158	138	153	155
[Beats/Minute]	S.D.	12.5	6.2	25.5	7.5	5.0
day 2	N	3	3	3	3	3
	P-Value	@0.2283				
Diastolic Blood Pressure	Mean	78 a	80	80	64	89
[mmHg]	S.D.	5.5	2.1	37.6	14.7	41.0
day 2	N	3	3	3	3	3
	P-Value	@0.8337				
Systolic Blood Pressure	Mean	132 k	134	124	134	150
[mmHg]	S.D.	10.6	4.0	41.2	22.2	33.7
day 2	N	3	3	3	3	3
	P-Value	@0.9195				
Mean Arterial Pressure	Mean	91 a	93	90	84	102
[mmHg]	S.D.	9.0	2.5	35.5	17.8	41.9
day 2	N	3	3	3	3	3
	P-Value	@0.9391				

a=ANOVA; k=KRUSKAL-WALLIS

Table 6b – Summary of Heart Rate and Blood Pressure Data (vs. Group 2)

DAY 3 (MALES)

		G 2 / M Vehicle		G 1 / M Saline	G 3 / M Low 0.019 mg/L	G 4 / M Mid 0.038 mg/L	G 5 / M High 0.075 mg/L
Heart Rate	Mean	138	a	129	129	117	146
[Beats/Minute]	S.D.	12.7		24.1	30.9	19.0	21.8
day 3	N	3		3	3	3	3
	P-Value	@0.6083					
Diastolic Blood Pressure	Mean	68	k	80	71	70	107
[mmHg]	S.D.	2.1		1.7	20.6	13.1	48.8
day 3	N	3		3	3	3	3
	P-Value	@0.7145					
Systolic Blood Pressure	Mean	132	a	126	97	155	141
[mmHg]	S.D.	35.3		26.9	24.9	9.9	63.6
day 3	N	3		3	3	3	3
	P-Value	@0.4350					
Mean Arterial Pressure	Mean	85	a	91	77	88	113
[mmHg]	S.D.	11.0		5.3	20.9	8.5	51.5
day 3	N	3		3	3	3	3
	P-Value	@0.5284					

a=ANOVA; k=KRUSKAL-WALLIS

DAY 3 (FEMALES)

		G 2 / F Vehicle		G 1 / F Saline	G 3 / F Low 0.019 mg/L	G 4 / F Mid 0.038 mg/L	G 5 / F High 0.075 mg/L
Heart Rate	Mean	127	a	148	129	154	142
[Beats/Minute]	S.D.	27.0		13.7	28.3	8.3	13.2
day 3	N	3		3	3	3	3
	P-Value	@0.4346					
Diastolic Blood Pressure	Mean	50	k	99	98	101	93
[mmHg]	S.D.	1.2		30.3	24.2	14.7	26.9
day 3	N	3		3	3	3	3
	P-Value	@0.1234					
Systolic Blood Pressure	Mean	123	k	143	142	150	151
[mmHg]	S.D.	49.6		3.5	23.3	12.6	23.5
day 3	N	3		3	3	3	3
	P-Value	@0.9552					
Mean Arterial Pressure	Mean	70	a	106	106	111	107
[mmHg]	S.D.	13.3		27.1	23.9	15.4	24.3
day 3	N	3		3	3	3	3
	P-Value	@0.1876					

a=ANOVA; k=KRUSKAL-WALLIS

Table 6b – Summary of Heart Rate and Blood Pressure Data (vs. Group 2)

DAY 4 (MALES)

		G 2 / M Vehicle	G 1 / M Saline	G 3 / M Low 0.019 mg/L	G 4 / M Mid 0.038 mg/L	G 5 / M High 0.075 mg/L
Heart Rate	Mean	122 a	127	155	102	134
[Beats/Minute]	S.D.	17.7	6.8	30.4	22.3	26.6
day 4	N	3	3	3	3	3
	P-Value	@0.1330				
Diastolic Blood Pressure	Mean	53 k	69	66	88	90
[mmHg]	S.D.	7.0	13.7	48.2	21.9	38.0
day 4	N	3	3	3	3	3
	P-Value	@0.3039				
Systolic Blood Pressure	Mean	102 a	127	149	124	141
[mmHg]	S.D.	36.3	25.7	11.2	35.0	63.7
day 4	N	3	3	3	3	3
	P-Value	@0.6310				
Mean Arterial Pressure	Mean	66 a	80	109	96	98
[mmHg]	S.D.	7.8	16.7	16.2	25.4	42.6
day 4	N	3	3	3	3	3
	P-Value	@0.3192				

a=ANOVA; k=KRUSKAL-WALLIS

DAY 4 (FEMALES)

		G 2 / F Vehicle	G 1 / F Saline	G 3 / F Low 0.019 mg/L	G 4 / F Mid 0.038 mg/L	G 5 / F High 0.075 mg/L
Heart Rate	Mean	144 a	149	139	153	140
[Beats/Minute]	S.D.	27.4	19.7	21.2	3.8	39.9
day 4	N	3	3	3	3	3
	P-Value	@0.9454				
Diastolic Blood Pressure	Mean	82 k	126	76	96	76
[mmHg]	S.D.	0.6	18.9	18.6	22.9	12.5
day 4	N	3	3	3	3	3
	P-Value	@0.1350				
Systolic Blood Pressure	Mean	150 k	178	147	181	126
[mmHg]	S.D.	15.6	13.4	26.5	33.2	38.4
day 4	N	3	3	3	3	3
	P-Value	@0.2066				
Mean Arterial Pressure	Mean	96 a	136	89	115	90
[mmHg]	S.D.	9.6	15.7	22.2	21.9	22.3
day 4	N	3	3	3	3	3
	P-Value	@0.0551				

a=ANOVA; k=KRUSKAL-WALLIS

Table 6b – Summary of Heart Rate and Blood Pressure Data (vs. Group 2)

DAY 5 (MALES)

		G 2 / M Vehicle	G 1 / M Saline	G 3 / M Low 0.019 mg/L	G 4 / M Mid 0.038 mg/L	G 5 / M High 0.075 mg/L
Heart Rate	Mean	161 k	138	144	138	133
[Beats/Minute]	S.D.	20.4	20.6	20.5	13.3	41.2
day 5	N	3	3	3	3	3
	P-Value	@0.7820				
Diastolic Blood Pressure	Mean	97 k	63	130	85	93
[mmHg]	S.D.	28.0	19.1	53.5	21.5	19.5
day 5	N	3	3	3	3	3
	P-Value	@0.3862				
Systolic Blood Pressure	Mean	157 a	112	170	119	150
[mmHg]	S.D.	22.1	23.6	45.6	32.7	1.5
day 5	N	3	3	3	3	3
	P-Value	@0.1406				
Mean Arterial Pressure	Mean	109 a	72	140	90	107
[mmHg]	S.D.	24.9	22.5	54.0	21.0	14.0
day 5	N	3	3	3	3	3
	P-Value	@0.1648				

k=KRUSKAL-WALLIS; a=ANOVA

DAY 5 (FEMALES)

		G 2 / F Vehicle	G 1 / F Saline	G 3 / F Low 0.019 mg/L	G 4 / F Mid 0.038 mg/L	G 5 / F High 0.075 mg/L
Heart Rate	Mean	149 a	150	144	133	140
[Beats/Minute]	S.D.	9.1	8.1	4.7	9.5	8.4
day 5	N	3	3	3	3	3
	P-Value	@0.1459				
Diastolic Blood Pressure	Mean	77 a	86	94	65	67
[mmHg]	S.D.	22.4	9.1	12.5	33.5	17.3
day 5	N	3	3	3	3	3
	P-Value	@0.4441				
Systolic Blood Pressure	Mean	123 a	145	151	119	136
[mmHg]	S.D.	45.0	18.3	9.5	46.7	23.5
day 5	N	3	3	3	3	3
	P-Value	@0.7020				
Mean Arterial Pressure	Mean	82 k	99	111	77	78
[mmHg]	S.D.	23.4	16.2	11.7	35.9	18.1
day 5	N	3	3	3	3	3
	P-Value	@0.3309				

a=ANOVA; k=KRUSKAL-WALLIS

Table 6b – Summary of Heart Rate and Blood Pressure Data (vs. Group 2)

DAY 6 (MALES)

		G 2 / M Vehicle	G 1 / M Saline	G 3 / M Low 0.019 mg/L	G 4 / M Mid 0.038 mg/L	G 5 / M High 0.075 mg/L
Heart Rate	Mean	134 a	142	132	138	143
[Beats/Minute]	S.D.	46.0	18.2	30.8	9.0	12.5
day 6	N	3	3	3	3	3
	P-Value	@0.9827				
Diastolic Blood Pressure	Mean	120 a	71	88	66	95
[mmHg]	S.D.	17.9	24.5	20.1	30.0	1.5
day 6	N	3	3	3	3	3
	P-Value	@0.0673				
Systolic Blood Pressure	Mean	162 d	129 *	147	162	143
[mmHg]	S.D.	10.6	11.3	7.2	18.3	9.0
day 6	N	3	3	3	3	3
	P-Value	@0.0321	0.0231	0.4097	1.0000	0.2431
Mean Arterial Pressure	Mean	131 d	80 *	101	92 *	106
[mmHg]	S.D.	14.7	21.5	17.4	17.8	4.2
day 6	N	3	3	3	3	3
	P-Value	@0.0334	0.0115	0.1372	0.0459	0.2619

a=ANOVA; d=ANOVA-DUNNETT; * = p < 0.05

DAY 6 (FEMALES)

		G 2 / F Vehicle	G 1 / F Saline	G 3 / F Low 0.019 mg/L	G 4 / F Mid 0.038 mg/L	G 5 / F High 0.075 mg/L
Heart Rate	Mean	123 k	159	139	151	166
[Beats/Minute]	S.D.	39.6	15.0	17.7	4.0	16.8
day 6	N	3	3	3	3	3
	P-Value	@0.3075				
Diastolic Blood Pressure	Mean	70 a	77	105	120	111
[mmHg]	S.D.	23.4	20.3	32.3	28.3	32.6
day 6	N	3	3	3	3	3
	P-Value	@0.1660				
Systolic Blood Pressure	Mean	118 a	165	148	171	173
[mmHg]	S.D.	44.9	10.0	29.5	31.5	36.7
day 6	N	3	3	3	3	3
	P-Value	@0.2761				
Mean Arterial Pressure	Mean	82 k	93	114	129	121
[mmHg]	S.D.	31.2	21.5	31.9	27.9	28.6
day 6	N	3	3	3	3	3
	P-Value	@0.3270				

k=KRUSKAL-WALLIS; a=ANOVA

Table 6b – Summary of Heart Rate and Blood Pressure Data (vs. Group 2)

DAY 7 (MALES)

		G 2 / M Vehicle	G 1 / M Saline	G 3 / M Low 0.019 mg/L	G 4 / M Mid 0.038 mg/L	G 5 / M High 0.075 mg/L
Heart Rate	Mean	157 k	127	148	115	123
[Beats/Minute]	S.D.	5.0	15.8	9.3	27.6	13.3
day 7	N	3	3	3	3	3
	P-Value	@0.0760				
Diastolic Blood Pressure	Mean	69 a	67	82	62	94
[mmHg]	S.D.	30.8	12.5	7.5	31.7	0.0
day 7	N	3	3	3	3	3
	P-Value	@0.3836				
Systolic Blood Pressure	Mean	140 k	135	179	114	150
[mmHg]	S.D.	20.4	18.5	4.6	60.5	3.5
day 7	N	3	3	3	3	3
	P-Value	@0.1234				
Mean Arterial Pressure	Mean	83 k	82	114	74	107
[mmHg]	S.D.	23.7	12.0	5.7	39.4	0.6
day 7	N	3	3	3	3	3
	P-Value	@0.0620				

k=KRUSKAL-WALLIS; a=ANOVA

DAY 7 (FEMALES)

		G 2 / F Vehicle	G 1 / F Saline	G 3 / F Low 0.019 mg/L	G 4 / F Mid 0.038 mg/L	G 5 / F High 0.075 mg/L
Heart Rate	Mean	148 a	155	129	156	147
[Beats/Minute]	S.D.	5.9	7.0	13.0	3.8	21.1
day 7	N	3	3	3	3	3
	P-Value	@0.1146				
Diastolic Blood Pressure	Mean	75 k	89	102	101	96
[mmHg]	S.D.	17.4	23.9	28.9	1.7	4.9
day 7	N	3	3	3	3	3
	P-Value	@0.3232				
Systolic Blood Pressure	Mean	121 k	155	158	141	157
[mmHg]	S.D.	7.5	19.9	23.4	7.5	4.4
day 7	N	3	3	3	3	3
	P-Value	@0.0790				
Mean Arterial Pressure	Mean	85 a	105	113	116	110
[mmHg]	S.D.	16.5	19.2	28.8	9.0	9.6
day 7	N	3	3	3	3	3
	P-Value	@0.2981				

a=ANOVA; k=KRUSKAL-WALLIS

Table 6b – Summary of Heart Rate and Blood Pressure Data (vs. Group 2)

DAY 8 (MALES)

		G 2 / M Vehicle	G 1 / M Saline	G 3 / M Low 0.019 mg/L	G 4 / M Mid 0.038 mg/L	G 5 / M High 0.075 mg/L
Heart Rate	Mean	150 a	124	142	132	142
[Beats/Minute]	S.D.	6.7	8.1	18.7	11.2	33.9
day 8	N	3	3	3	3	3
	P-Value	@0.5325				
Diastolic Blood Pressure	Mean	72 a	88	101	81	84
[mmHg]	S.D.	39.4	14.6	7.8	26.9	25.9
day 8	N	3	3	3	3	3
	P-Value	@0.7142				
Systolic Blood Pressure	Mean	118 a	148	153	150	129
[mmHg]	S.D.	53.6	20.1	10.7	11.2	48.6
day 8	N	3	3	3	3	3
	P-Value	@0.6626				
Mean Arterial Pressure	Mean	78 a	98	113	93	92
[mmHg]	S.D.	39.1	11.2	9.6	24.1	28.5
day 8	N	3	3	3	3	3
	P-Value	@0.5708				

a=ANOVA

DAY 8 (FEMALES)

		G 2 / F Vehicle	G 1 / F Saline	G 3 / F Low 0.019 mg/L	G 4 / F Mid 0.038 mg/L	G 5 / F High 0.075 mg/L
Heart Rate	Mean	146 d	165	117	149	149
[Beats/Minute]	S.D.	9.1	3.8	20.5	16.5	21.0
day 8	N	3	3	3	3	3
	P-Value	@0.0417	0.4524	0.1263	0.9985	0.9985
Diastolic Blood Pressure	Mean	78 k	85	84	92	74
[mmHg]	S.D.	3.2	13.3	2.9	41.3	20.0
day 8	N	3	3	3	3	3
	P-Value	@0.7780				
Systolic Blood Pressure	Mean	135 k	150	157	142	149
[mmHg]	S.D.	8.9	6.5	3.5	28.6	5.2
day 8	N	3	3	3	3	3
	P-Value	@0.1959				
Mean Arterial Pressure	Mean	93 a	103	94	100	91
[mmHg]	S.D.	4.7	6.7	6.0	41.6	14.9
day 8	N	3	3	3	3	3
	P-Value	@0.9404				

d=ANOVA-DUNNETT; k=KRUSKAL-WALLIS; a=ANOVA

Table 6b – Summary of Heart Rate and Blood Pressure Data (vs. Group 2)

DAY 9 (MALES)

		G 2 / M Vehicle	G 1 / M Saline	G 3 / M Low 0.019 mg/L	G 4 / M Mid 0.038 mg/L	G 5 / M High 0.075 mg/L
Heart Rate	Mean	155 k	139	140	139	123
[Beats/Minute]	S.D.	14.2	10.4	19.6	23.1	8.5
day 9	N	3	3	3	3	3
	P-Value	@0.2879				
Diastolic Blood Pressure	Mean	87 k	95	94	94	66
[mmHg]	S.D.	22.7	30.3	19.1	2.1	19.0
day 9	N	3	3	3	3	3
	P-Value	@0.4189				
Systolic Blood Pressure	Mean	138 a	127	155	147	137
[mmHg]	S.D.	23.4	2.5	16.9	9.3	25.3
day 9	N	3	3	3	3	3
	P-Value	@0.4294				
Mean Arterial Pressure	Mean	98 a	87	103	104	86
[mmHg]	S.D.	23.5	5.1	19.1	2.5	20.6
day 9	N	3	3	3	3	3
	P-Value	@0.5139				

k=KRUSKAL-WALLIS; a=ANOVA

DAY 9 (FEMALES)

		G 2 / F Vehicle	G 1 / F Saline	G 3 / F Low 0.019 mg/L	G 4 / F Mid 0.038 mg/L	G 5 / F High 0.075 mg/L
Heart Rate	Mean	138 a	152	140	144	140
[Beats/Minute]	S.D.	15.1	5.1	37.1	15.5	9.8
day 9	N	3	3	3	3	3
	P-Value	@0.9152				
Diastolic Blood Pressure	Mean	93 k	81	89	82	96
[mmHg]	S.D.	0.6	11.1	19.2	23.9	16.2
day 9	N	3	3	3	3	3
	P-Value	@0.6535				
Systolic Blood Pressure	Mean	133 a	138	159	155	158
[mmHg]	S.D.	1.0	5.9	29.5	11.2	10.0
day 9	N	3	3	3	3	3
	P-Value	@0.1741				
Mean Arterial Pressure	Mean	101 k	97	105	102	108
[mmHg]	S.D.	1.2	15.0	25.2	19.3	15.5
day 9	N	3	3	3	3	3
	P-Value	@0.8183				

a=ANOVA; k=KRUSKAL-WALLIS

Table 6b – Summary of Heart Rate and Blood Pressure Data (vs. Group 2)

DAY 10 (MALES)

		G 2 / M Vehicle	G 1 / M Saline	G 3 / M Low 0.019 mg/L	G 4 / M Mid 0.038 mg/L	G 5 / M High 0.075 mg/L
Heart Rate	Mean	148 d	124	166	128	137
[Beats/Minute]	S.D.	13.5	13.5	19.1	7.2	18.1
day 10	N	3	3	3	3	3
	P-Value	@0.0380	0.2233	0.4260	0.3305	0.7946
Diastolic Blood Pressure	Mean	98 k	100	109	98	82
[mmHg]	S.D.	11.0	38.4	16.2	40.7	29.1
day 10	N	3	3	3	3	3
	P-Value	@0.7476				
Systolic Blood Pressure	Mean	132 a	148	164	144	136
[mmHg]	S.D.	5.5	26.1	15.7	53.4	58.4
day 10	N	3	3	3	3	3
	P-Value	@0.8505				
Mean Arterial Pressure	Mean	105 a	110	122	107	92
[mmHg]	S.D.	10.1	38.0	13.7	43.0	32.9
day 10	N	3	3	3	3	3
	P-Value	@0.8171				

d=ANOVA-DUNNETT; k=KRUSKAL-WALLIS; a=ANOVA

DAY 10 (FEMALES)

		G 2 / F Vehicle	G 1 / F Saline	G 3 / F Low 0.019 mg/L	G 4 / F Mid 0.038 mg/L	G 5 / F High 0.075 mg/L
Heart Rate	Mean	153 a	135	140	156	172
[Beats/Minute]	S.D.	25.1	31.5	16.8	20.6	18.7
day 10	N	3	3	3	3	3
	P-Value	@0.3754				
Diastolic Blood Pressure	Mean	94 a	87	105	101	101
[mmHg]	S.D.	17.9	13.0	15.5	9.9	24.1
day 10	N	3	3	3	3	3
	P-Value	@0.7044				
Systolic Blood Pressure	Mean	160 a	134	149	168	158
[mmHg]	S.D.	17.3	27.8	9.0	14.6	20.5
day 10	N	3	3	3	3	3
	P-Value	@0.3072				
Mean Arterial Pressure	Mean	107 a	97	114	118	113
[mmHg]	S.D.	14.0	15.4	14.4	3.1	24.6
day 10	N	3	3	3	3	3
	P-Value	@0.5404				

a=ANOVA

Table 6b – Summary of Heart Rate and Blood Pressure Data (vs. Group 2)

DAY 11 (MALES)

		G 2 / M Vehicle	G 1 / M Saline	G 3 / M Low 0.019 mg/L	G 4 / M Mid 0.038 mg/L	G 5 / M High 0.075 mg/L
Heart Rate	Mean	161 a	113	125	125	125
[Beats/Minute]	S.D.	18.0	29.8	32.5	11.8	15.3
day 11	N	3	3	3	3	3
	P-Value	@0.1881				
Diastolic Blood Pressure	Mean	80 a	67	108	111	94
[mmHg]	S.D.	11.0	25.1	39.1	10.8	4.6
day 11	N	3	3	3	3	3
	P-Value	@0.1481				
Systolic Blood Pressure	Mean	132 a	116	157	169	162
[mmHg]	S.D.	22.1	10.3	36.4	14.0	5.6
day 11	N	3	3	3	3	3
	P-Value	@0.0508				
Mean Arterial Pressure	Mean	89 a	79	116	124	108
[mmHg]	S.D.	10.7	17.6	35.9	3.2	9.0
day 11	N	3	3	3	3	3
	P-Value	@0.0752				

a=ANOVA

DAY 11 (FEMALES)

		G 2 / F Vehicle	G 1 / F Saline	G 3 / F Low 0.019 mg/L	G 4 / F Mid 0.038 mg/L	G 5 / F High 0.075 mg/L
Heart Rate	Mean	165 a	129	152	143	141
[Beats/Minute]	S.D.	14.6	41.7	20.1	12.5	12.2
day 11	N	3	3	3	3	3
	P-Value	@0.4365				
Diastolic Blood Pressure	Mean	87 k	100	79	85	96
[mmHg]	S.D.	19.1	32.7	40.4	30.6	8.0
day 11	N	3	3	3	3	3
	P-Value	@0.9255				
Systolic Blood Pressure	Mean	150 a	153	161	173	155
[mmHg]	S.D.	5.1	15.9	17.9	2.1	9.5
day 11	N	3	3	3	3	3
	P-Value	@0.2014				
Mean Arterial Pressure	Mean	100 k	113	100	104	104
[mmHg]	S.D.	14.4	30.1	31.2	21.0	11.1
day 11	N	3	3	3	3	3
	P-Value	@0.9850				

a=ANOVA; k=KRUSKAL-WALLIS

Table 6b – Summary of Heart Rate and Blood Pressure Data (vs. Group 2)

DAY 12 (MALES)

		G 2 / M Vehicle	G 1 / M Saline	G 3 / M Low 0.019 mg/L	G 4 / M Mid 0.038 mg/L	G 5 / M High 0.075 mg/L
Heart Rate	Mean	157 k	133	150	144	135
[Beats/Minute]	S.D.	4.0	16.5	23.7	17.2	14.4
day 12	N	3	3	3	3	3
	P-Value	@0.3780				
Diastolic Blood Pressure	Mean	76 a	80	92	93	78
[mmHg]	S.D.	2.1	3.6	1.5	30.6	14.2
day 12	N	3	3	3	3	3
	P-Value	@0.5376				
Systolic Blood Pressure	Mean	141 d	130	158	150	162 *
[mmHg]	S.D.	2.1	11.6	2.0	13.1	5.6
day 12	N	3	3	3	3	3
	P-Value	@0.0048	0.3076	0.1003	0.5117	0.0384
Mean Arterial Pressure	Mean	91 a	90	107	104	100
[mmHg]	S.D.	3.6	6.6	4.2	35.3	11.4
day 12	N	3	3	3	3	3
	P-Value	@0.6523				

k=KRUSKAL-WALLIS; a=ANOVA; d=ANOVA-DUNNETT; * = p < 0.05

DAY 12 (FEMALES)

		G 2 / F Vehicle	G 1 / F Saline	G 3 / F Low 0.019 mg/L	G 4 / F Mid 0.038 mg/L	G 5 / F High 0.075 mg/L
Heart Rate	Mean	146 a	147	138	161	153
[Beats/Minute]	S.D.	28.6	13.6	15.0	29.0	12.9
day 12	N	3	3	3	3	3
	P-Value	@0.7391				
Diastolic Blood Pressure	Mean	73 a	83	90	78	97
[mmHg]	S.D.	20.3	2.1	6.8	30.7	10.0
day 12	N	3	3	3	3	3
	P-Value	@0.4774				
Systolic Blood Pressure	Mean	133 a	137	144	164	149
[mmHg]	S.D.	9.5	11.0	12.2	42.1	3.0
day 12	N	3	3	3	3	3
	P-Value	@0.4301				
Mean Arterial Pressure	Mean	88 a	90	100	100	109
[mmHg]	S.D.	16.8	7.0	7.4	34.1	8.5
day 12	N	3	3	3	3	3
	P-Value	@0.6288				

a=ANOVA

Table 6b – Summary of Heart Rate and Blood Pressure Data (vs. Group 2)

DAY 13 (MALES)

		G 2 / M Vehicle	G 1 / M Saline	G 3 / M Low 0.019 mg/L	G 4 / M Mid 0.038 mg/L	G 5 / M High 0.075 mg/L
Heart Rate	Mean	156 a	117	150	133	130
[Beats/Minute]	S.D.	6.4	5.7	14.0	37.3	17.2
day 13	N	3	3	3	3	3
	P-Value	@0.1861				
Diastolic Blood Pressure	Mean	84 k	72	89	84	74
[mmHg]	S.D.	20.8	2.6	0.6	46.5	19.4
day 13	N	3	3	3	3	3
	P-Value	@0.4569				
Systolic Blood Pressure	Mean	130 k	116	165	125	143
[mmHg]	S.D.	11.8	3.8	12.1	70.2	6.6
day 13	N	3	3	3	3	3
	P-Value	@0.0843				
Mean Arterial Pressure	Mean	96 k	81	105	88	86
[mmHg]	S.D.	18.9	1.5	8.4	47.9	20.6
day 13	N	3	3	3	3	3
	P-Value	@0.5523				

a=ANOVA; k=KRUSKAL-WALLIS

DAY 13 (FEMALES)

		G 2 / F Vehicle	G 1 / F Saline	G 3 / F Low 0.019 mg/L	G 4 / F Mid 0.038 mg/L	G 5 / F High 0.075 mg/L
Heart Rate	Mean	139 k	148	131	144	150
[Beats/Minute]	S.D.	23.8	19.1	29.7	11.2	6.9
day 13	N	3	3	3	3	3
	P-Value	@0.8301				
Diastolic Blood Pressure	Mean	94 k	89	84	75	90
[mmHg]	S.D.	0.6	5.0	21.4	21.2	11.6
day 13	N	3	3	3	3	3
	P-Value	@0.4387				
Systolic Blood Pressure	Mean	141 a	141	138	135	146
[mmHg]	S.D.	3.8	34.6	8.6	23.6	2.0
day 13	N	3	3	3	3	3
	P-Value	@0.9645				
Mean Arterial Pressure	Mean	103 a	102	94	85	101
[mmHg]	S.D.	2.1	13.6	17.6	19.2	7.2
day 13	N	3	3	3	3	3
	P-Value	@0.4598				

k=KRUSKAL-WALLIS; a=ANOVA

Table 6b – Summary of Heart Rate and Blood Pressure Data (vs. Group 2)

DAY 14 (MALES)

		G 2 / M Vehicle	G 1 / M Saline	G 3 / M Low 0.019 mg/L	G 4 / M Mid 0.038 mg/L	G 5 / M High 0.075 mg/L
Heart Rate	Mean	144 a	111	140	149	122
[Beats/Minute]	S.D.	12.8	31.7	8.5	15.3	15.8
day 14	N	3	3	3	3	3
	P-Value	@0.1337				
Diastolic Blood Pressure	Mean	63 u	69	133 *	101	91
[mmHg]	S.D.	15.6	26.4	32.0	6.4	6.2
day 14	N	3	3	3	3	3
	P-Value	@0.0320	1.0000	0.0138	0.2442	1.0000
Systolic Blood Pressure	Mean	128 k	114	175	148	157
[mmHg]	S.D.	56.6	39.5	21.7	9.3	7.0
day 14	N	3	3	3	3	3
	P-Value	@0.0947				
Mean Arterial Pressure	Mean	79 d	81	141 *	113	110
[mmHg]	S.D.	26.5	22.0	33.9	3.5	6.1
day 14	N	3	3	3	3	3
	P-Value	@0.0324	0.9999	0.0198	0.2494	0.3169

a=ANOVA; u=KRUSKAL-WALLIS-DUNN; * = p < 0.05; k=KRUSKAL-WALLIS; d=ANOVA-DUNNETT

DAY 14 (FEMALES)

		G 2 / F Vehicle	G 1 / F Saline	G 3 / F Low 0.019 mg/L	G 4 / F Mid 0.038 mg/L	G 5 / F High 0.075 mg/L
Heart Rate	Mean	140 a	154	135	147	147
[Beats/Minute]	S.D.	14.7	20.0	17.6	17.9	18.7
day 14	N	3	3	3	3	3
	P-Value	@0.7537				
Diastolic Blood Pressure	Mean	88 k	96	90	83	81
[mmHg]	S.D.	4.0	20.2	34.5	18.7	5.5
day 14	N	3	3	3	3	3
	P-Value	@0.8759				
Systolic Blood Pressure	Mean	132 a	141	146	151	147
[mmHg]	S.D.	6.2	9.0	15.7	9.9	2.5
day 14	N	3	3	3	3	3
	P-Value	@0.2358				
Mean Arterial Pressure	Mean	100 a	107	105	101	97
[mmHg]	S.D.	7.2	18.2	30.0	11.8	5.5
day 14	N	3	3	3	3	3
	P-Value	@0.9570				

a=ANOVA; k=KRUSKAL-WALLIS

Table 7a – Summary of Body Weight Data (vs. Group 1)
(kg)

MALES

		G 1 / M Saline	G 2 / M Vehicle	G 3 / M Low 0.019 mg/L	G 4 / M Mid 0.038 mg/L	G 5 / M High 0.075 mg/L
day 1	Mean	9.24 a	8.77	8.72	8.88	8.83
	S.D.	0.761	0.205	0.349	0.732	0.114
	N	3	3	3	3	3
	P-Value	@0.7427				
day 4	Mean	9.01 a	8.82	8.77	8.85	8.85
	S.D.	0.780	0.334	0.427	0.391	0.293
	N	3	3	3	3	3
	P-Value	@0.9755				
day 8	Mean	8.97 a	8.83	8.67	8.89	8.95
	S.D.	0.855	0.513	0.241	0.391	0.306
	N	3	3	3	3	3
	P-Value	@0.9505				
day 11	Mean	9.15 k	9.05	8.93	8.80	8.85
	S.D.	0.888	0.421	0.304	0.461	0.185
	N	3	3	3	3	3
	P-Value	@0.8722				

a=ANOVA; k=KRUSKAL-WALLIS

NOTE: Body weights collected on Days 1 and 11 were collected prior to the first exposure.

FEMALES

		G 1 / F Saline	G 2 / F Vehicle	G 3 / F Low 0.019 mg/L	G 4 / F Mid 0.038 mg/L	G 5 / F High 0.075 mg/L
day 1	Mean	7.49 a	7.09	6.82	6.88	7.31
	S.D.	1.454	0.830	0.732	0.465	0.862
	N	3	3	3	3	3
	P-Value	@0.8834				
day 4	Mean	7.55 a	7.27	6.73	6.81	7.45
	S.D.	1.323	0.682	0.762	0.534	0.801
	N	3	3	3	3	3
	P-Value	@0.6974				
day 8	Mean	7.47 k	7.23	6.76	6.85	7.33
	S.D.	1.396	0.687	0.692	0.618	0.781
	N	3	3	3	3	3
	P-Value	@0.8873				
day 11	Mean	7.50 a	7.28	6.80	6.87	7.50
	S.D.	1.374	0.538	0.694	0.628	0.786
	N	3	3	3	3	3
	P-Value	@0.7590				

a=ANOVA; k=KRUSKAL-WALLIS

NOTE: Body weights collected on Days 1 and 11 were collected prior to the first exposure.

Table 7b – Summary of Body Weight Data (vs. Group 2)
(kg)

MALES

		G 2 / M Vehicle		G 1 / M Saline	G 3 / M Low 0.019 mg/L	G 4 / M Mid 0.038 mg/L	G 5 / M High 0.075 mg/L
day 1	Mean	8.77	a	9.24	8.72	8.88	8.83
	S.D.	0.205		0.761	0.349	0.732	0.114
	N	3		3	3	3	3
	P-Value	@0.7427					
day 4	Mean	8.82	a	9.01	8.77	8.85	8.85
	S.D.	0.334		0.780	0.427	0.391	0.293
	N	3		3	3	3	3
	P-Value	@0.9755					
day 8	Mean	8.83	a	8.97	8.67	8.89	8.95
	S.D.	0.513		0.855	0.241	0.391	0.306
	N	3		3	3	3	3
	P-Value	@0.9505					
day 11	Mean	9.05	k	9.15	8.93	8.80	8.85
	S.D.	0.421		0.888	0.304	0.461	0.185
	N	3		3	3	3	3
	P-Value	@0.8722					

a=ANOVA; k=KRUSKAL-WALLIS

NOTE: Body weights collected on Days 1 and 11 were collected prior to the first exposure.

FEMALES

		G 2 / F Vehicle		G 1 / F Saline	G 3 / F Low 0.019 mg/L	G 4 / F Mid 0.038 mg/L	G 5 / F High 0.075 mg/L
day 1	Mean	7.09	a	7.49	6.82	6.88	7.31
	S.D.	0.830		1.454	0.732	0.465	0.862
	N	3		3	3	3	3
	P-Value	@0.8834					
day 4	Mean	7.27	a	7.55	6.73	6.81	7.45
	S.D.	0.682		1.323	0.762	0.534	0.801
	N	3		3	3	3	3
	P-Value	@0.6974					
day 8	Mean	7.23	k	7.47	6.76	6.85	7.33
	S.D.	0.687		1.396	0.692	0.618	0.781
	N	3		3	3	3	3
	P-Value	@0.8873					
day 11	Mean	7.28	a	7.50	6.80	6.87	7.50
	S.D.	0.538		1.374	0.694	0.628	0.786
	N	3		3	3	3	3
	P-Value	@0.7590					

a=ANOVA; k=KRUSKAL-WALLIS

NOTE: Body weights collected on Days 1 and 11 were collected prior to the first exposure.

Table 8a – Summary of Body Weight Change Data (vs. Group 1)
(kg)

MALES

		G 1 / M Saline	G 2 / M Vehicle	G 3 / M Low 0.019 mg/L	G 4 / M Mid 0.038 mg/L	G 5 / M High 0.075 mg/L
d 1 to 4	Mean	-0.23 a	0.05	0.05	-0.03	0.03
	S.D.	0.031	0.130	0.081	0.386	0.180
	N	3	3	3	3	3
	P-Value	@0.4622				
d 4 to 8	Mean	-0.04 a	0.01	-0.09	0.04	0.10
	S.D.	0.183	0.181	0.204	0.000	0.020
	N	3	3	3	3	3
	P-Value	@0.5747				
d 8 to 11	Mean	0.18 u	0.22	0.25	-0.09	-0.10
	S.D.	0.035	0.160	0.064	0.095	0.144
	N	3	3	3	3	3
	P-Value	@0.0315	1.0000	1.0000	0.3627	0.3627
d 1 to 11	Mean	-0.09 a	0.28	0.21	-0.08	0.03
	S.D.	0.240	0.231	0.095	0.390	0.081
	N	3	3	3	3	3
	P-Value	@0.2735				

d = day; a=ANOVA; u=KRUSKAL-WALLIS-DUNN

NOTE: Body weights collected on Days 1 and 11 were collected prior to the first exposure.

FEMALES

		G 1 / F Saline	G 2 / F Vehicle	G 3 / F Low 0.019 mg/L	G 4 / F Mid 0.038 mg/L	G 5 / F High 0.075 mg/L
d 1 to 4	Mean	0.05 a	0.18	-0.09	-0.07	0.15
	S.D.	0.133	0.260	0.083	0.070	0.061
	N	3	3	3	3	3
	P-Value	@0.1387				
d 4 to 8	Mean	-0.07 k	-0.05	0.03	0.04	-0.12
	S.D.	0.083	0.012	0.101	0.111	0.020
	N	3	3	3	3	3
	P-Value	@0.0986				
d 8 to 11	Mean	0.03 k	0.05	0.04	0.02	0.17
	S.D.	0.023	0.151	0.087	0.035	0.023
	N	3	3	3	3	3
	P-Value	@0.1390				
d 1 to 11	Mean	0.01 a	0.19	-0.02	-0.01	0.19
	S.D.	0.081	0.358	0.100	0.181	0.081
	N	3	3	3	3	3
	P-Value	@0.4631				

d = day; a=ANOVA; k=KRUSKAL-WALLIS

NOTE: Body weights collected on Days 1 and 11 were collected prior to the first exposure.

Table 8b – Summary of Body Weight Change Data (vs. Group 2)
(kg)

MALES

		G 2 / M Vehicle	G 1 / M Saline	G 3 / M Low 0.019 mg/L	G 4 / M Mid 0.038 mg/L	G 5 / M High 0.075 mg/L
d 1 to 4	Mean	0.05 a	-0.23	0.05	-0.03	0.03
	S.D.	0.130	0.031	0.081	0.386	0.180
	N	3	3	3	3	3
	P-Value	@0.4622				
d 4 to 8	Mean	0.01 a	-0.04	-0.09	0.04	0.10
	S.D.	0.181	0.183	0.204	0.000	0.020
	N	3	3	3	3	3
	P-Value	@0.5747				
d 8 to 11	Mean	0.22 u	0.18	0.25	-0.09	-0.10
	S.D.	0.160	0.035	0.064	0.095	0.144
	N	3	3	3	3	3
	P-Value	@0.0315	1.0000	1.0000	0.1265	0.1265
d 1 to 11	Mean	0.28 a	-0.09	0.21	-0.08	0.03
	S.D.	0.231	0.240	0.095	0.390	0.081
	N	3	3	3	3	3
	P-Value	@0.2735				

d = day; a=ANOVA; u=KRUSKAL-WALLIS-DUNN

NOTE: Body weights collected on Days 1 and 11 were collected prior to the first exposure.

FEMALES

		G 2 / F Vehicle	G 1 / F Saline	G 3 / F Low 0.019 mg/L	G 4 / F Mid 0.038 mg/L	G 5 / F High 0.075 mg/L
d 1 to 4	Mean	0.18 a	0.05	-0.09	-0.07	0.15
	S.D.	0.260	0.133	0.083	0.070	0.061
	N	3	3	3	3	3
	P-Value	@0.1387				
d 4 to 8	Mean	-0.05 k	-0.07	0.03	0.04	-0.12
	S.D.	0.012	0.083	0.101	0.111	0.020
	N	3	3	3	3	3
	P-Value	@0.0986				
d 8 to 11	Mean	0.05 k	0.03	0.04	0.02	0.17
	S.D.	0.151	0.023	0.087	0.035	0.023
	N	3	3	3	3	3
	P-Value	@0.1390				
d 1 to 11	Mean	0.19 a	0.01	-0.02	-0.01	0.19
	S.D.	0.358	0.081	0.100	0.181	0.081
	N	3	3	3	3	3
	P-Value	@0.4631				

d = day; a=ANOVA; k=KRUSKAL-WALLIS

NOTE: Body weights collected on Days 1 and 11 were collected prior to the first exposure.

Table 9a – Summary of Respiratory Rate Data (vs. Group 1)
(Breaths/Minute)

MALES

Study Day	Group 1 (Saline Control)			Group 2 (Vehicle Control)			Group 3 (APN01; 0.019 mg/L)			Group 4 (APN01; 0.038 mg/L)			Group 5 (APN01; 0.075 mg/L)		
	**Mean**	S.D.	*p*-value	**Mean**	S.D.	*p*-value	**Mean**	S.D.	*p*-value	**Mean**	S.D.	*p*-value	**Mean**	S.D.	*p*-value
1[a]	**37.0**	21.0	0.395	**49.8**	10.8	—	**50.8**	8.0	—	**49.5**	19.9	—	**67.0**	22.2	—
2[b]	**33.0**	3.1	0.093	**62.2**	11.3	—	**55.4**	8.5	—	**92.8**	57.4	—	**63.4**	9.3	—
3[b]	**37.5**	5.5	0.058	**79.0**	25.5	—	**43.4**	21.0	—	**164.1**	101.7	—	**58.5**	15.8	—
4[a]	**36.3**	15.4	0.103	**66.2**	39.1	—	**67.7**	22.0	—	**113.0**	44.2	—	**57.4**	19.8	—
5[a]	**42.4**	8.1	0.091	**43.8**	12.6	—	**87.6**	53.6	—	**111.4**	47.5	—	**47.2**	7.2	—
6[a]	**40.1**	21.0	0.047	**57.6**	21.5	0.749	**67.5**	18.6	0.424	**104.6***	36.1	0.019	**51.6**	2.0	0.921
7[a]	**40.0**	9.5	<0.001	**85.8**	55.4	0.371	**65.4**	9.7	0.791	**232.8***	51.3	<0.001	**53.3**	19.0	0.971
8[a]	**84.1**	47.6	0.036	**118.3**	120.3	0.918	**119.1**	68.1	0.912	**256.7***	23.6	0.031	**61.7**	7.9	0.980
9[a]	**78.7**	60.6	0.002	**144.6**	49.1	0.298	**95.6**	53.9	0.974	**261.9***	37.3	0.002	**65.6**	16.8	0.989
10[a]	**47.4**	19.0	0.356	**70.1**	50.8	—	**71.9**	54.4	—	**123.5**	66.6	—	**56.8**	14.4	—
11[b]	**34.9**	5.1	0.097	**45.4**	25.6	—	**50.0**	12.6	—	**171.5**	156.7	—	**74.9**	17.5	—
12[b]	**41.3**	8.1	0.264	**81.2**	51.2	—	**102.4**	56.0	—	**100.5**	57.1	—	**89.9**	67.7	—
13[b]	**32.7**	5.7	0.149	**54.8**	14.5	—	**101.6**	78.4	—	**185.1**	129.8	—	**46.0**	19.1	—
14[a]	**30.6**	7.6	<0.001	**49.0**	30.5	0.691	**73.8**	31.6	0.102	**188.7***	16.1	<0.001	**59.0**	9.3	0.358

[a] Parameter analyzed for statistical significance using one-way ANOVA (*p*-value listed in Group 1 column). When one-way ANOVA results were statistically significant ($p < 0.05$), Dunnett's test was used *post hoc* to determine statistical significance compared to Group 1. For tested time points, *p*-values for Dunnett's test are listed in Groups 2-5 columns.

[b] Parameter statistically analyzed using Kruskal-Wallis one-way ANOVA on ranks (*p*-value listed in Group 1 column). No statistically significant differences were seen between groups; therefore, no *post hoc* tests were run on group means for indicated time points.

* Result statistically significant ($p < 0.05$) compared to Group 1 (Dunnett's test).

NOTES:

[1] N = 3

[2] "—" = No *p*-value calculated/a *post hoc* test was not performed.

Table 9a – Summary of Respiratory Rate Data (vs. Group 1)
(Breaths/Minute)

FEMALES

Study Day	Group 1 (Saline Control)			Group 2 (Vehicle Control)			Group 3 (APN01; 0.019 mg/L)			Group 4 (APN01; 0.038 mg/L)			Group 5 (APN01; 0.075 mg/L)		
	**Mean**	S.D.	*p*-value	**Mean**	S.D.	*p*-value	**Mean**	S.D.	*p*-value	**Mean**	S.D.	*p*-value	**Mean**	S.D.	*p*-value
1[a]	**58.5**	29.6	0.379	**44.4**	6.8	—	**54.7**	23.6	—	**95.6**	55.7	—	**55.4**	5.9	—
2[a]	**49.2**	22.5	0.203	**89.7**	14.6	—	**93.1**	90.0	—	**199.6**	131.4	—	**86.0**	19.5	—
3[a]	**110.8**	100.1	0.813	**55.9**	17.8	—	**125.5**	149.6	—	**118.4**	51.2	—	**71.6**	37.6	—
4[a]	**100.3**	95.7	0.239	**65.9**	14.6	—	**72.8**	39.5	—	**197.4**	76.2	—	**109.3**	92.4	—
5[a]	**64.9**	41.1	0.109	**65.3**	19.0	—	**65.4**	25.1	—	**127.3**	27.2	—	**66.6**	34.0	—
6[a]	**110.0**	75.6	0.053	**64.8**	34.8	—	**57.7**	10.4	—	**181.1**	61.2	—	**69.2**	30.0	—
7[a]	**51.3**	1.4	0.437	**83.7**	3.5	—	**144.6**	138.0	—	**164.5**	93.1	—	**96.2**	55.3	—
8[a]	**138.3**	106.8	0.422	**74.4**	44.3	—	**121.7**	109.2	—	**194.6**	76.4	—	**86.2**	27.8	—
9[b]	**78.5**	35.3	0.894	**143.4**	162.0	—	**136.4**	131.0	—	**154.4**	136.2	—	**92.0**	39.3	—
10[b]	**83.6**	61.2	0.486	**101.9**	86.9	—	**121.6**	118.3	—	**170.1**	62.7	—	**57.2**	9.1	—
11[a]	**87.5**	57.1	0.808	**167.1**	63.6	—	**127.5**	128.6	—	**119.0**	67.1	—	**105.0**	73.3	—
12[a]	**52.3**	7.9	0.645	**152.6**	166.8	—	**129.3**	129.6	—	**166.7**	71.4	—	**91.2**	34.9	—
13[a]	**78.0**	31.1	0.258	**55.8**	7.4	—	**58.8**	8.3	—	**182.1**	186.0	—	**70.3**	15.0	—
14[b]	**65.6**	55.7	0.244	**50.7**	17.3	—	**40.1**	19.7	—	**184.3**	126.5	—	**57.5**	15.0	—

[a] Parameter statistically analyzed using one-way ANOVA (*p*-value listed in Group 1 column). No statistically significant differences were seen between groups; therefore, no *post hoc* tests were run on group means for indicated time points.

[b] Parameter statistically analyzed using Kruskal-Wallis one-way ANOVA on ranks (*p*-value listed in Group 1 column). No statistically significant differences were seen between groups; therefore, no *post hoc* tests were run on group means for indicated time points.

NOTES:

[1] N = 3, except for Group 1 at Day 14 and Group 3 at Day 1, where N=2

[2] "—" = No *p*-value calculated/a *post hoc* test was not performed.

Table 9b – Summary of Respiratory Rate Data (vs. Group 2)
(Breaths/Minute)

MALES

Study Day	Group 1 (Saline Control)			Group 2 (Vehicle Control)			Group 3 (APN01; 0.019 mg/L)			Group 4 (APN01; 0.038 mg/L)			Group 5 (APN01; 0.075 mg/L)		
	**Mean**	S.D.	*p*-value	**Mean**	S.D.	*p*-value	**Mean**	S.D.	*p*-value	**Mean**	S.D.	*p*-value	**Mean**	S.D.	*p*-value
1[a]	**37.0**	21.0	—	**49.8**	10.8	0.395	**50.8**	8.0	—	**49.5**	19.9	—	**67.0**	22.2	—
2[b]	**33.0**	3.1	—	**62.2**	11.3	0.093	**55.4**	8.5	—	**92.8**	57.4	—	**63.4**	9.3	—
3[b]	**37.5**	5.5	—	**79.0**	25.5	0.058	**43.4**	21.0	—	**164.1**	101.7	—	**58.5**	15.8	—
4[a]	**36.3**	15.4	—	**66.2**	39.1	0.103	**67.7**	22.0	—	**113.0**	44.2	—	**57.4**	19.8	—
5[a]	**42.4**	8.1	—	**43.8**	12.6	0.091	**87.6**	53.6	—	**111.4**	47.5	—	**47.2**	7.2	—
6[a]	**40.1**	21.0	0.749	**57.6**	21.5	0.047	**67.5**	18.6	0.953	**104.6**	36.1	0.088	**51.6**	2.0	0.992
7[a]	**40.0**	9.5	0.371	**85.8**	55.4	<0.001	**65.4**	9.7	0.887	**232.8***	51.3	0.002	**53.3**	19.0	0.638
8[a]	**84.1**	47.6	0.918	**118.3**	120.3	0.036	**119.1**	68.1	1.000	**256.7**	23.6	0.086	**61.7**	7.9	0.0690
9[a]	**78.7**	60.6	0.298	**144.6**	49.1	0.002	**95.6**	53.9	0.530	**261.9***	37.3	0.035	**65.6**	16.8	0.178
10[a]	**47.4**	19.0	—	**70.1**	50.8	0.356	**71.9**	54.4	—	**123.5**	66.6	—	**56.8**	14.4	—
11[b]	**34.9**	5.1	—	**45.4**	25.6	0.097	**50.0**	12.6	—	**171.5**	156.7	—	**74.9**	17.5	—
12[a]	**41.3**	8.1	—	**81.2**	51.2	0.0356	**102.4**	56.0	—	**100.5**	57.1	—	**89.9**	67.7	—
13[b]	**32.7**	5.7	—	**54.8**	14.5	0.149	**101.6**	78.4	—	**185.1**	129.8	—	**46.0**	19.1	—
14[a]	**30.6**	7.6	0.691	**49.0**	30.5	<0.001	**73.8**	31.6	0.468	**188.7***	16.1	<0.001	**59.0**	9.3	0.942

[a] Parameter analyzed for statistical significance using one-way ANOVA (*p*-value listed in Group 2 column). When one-way ANOVA results were statistically significant ($p < 0.05$), Dunnett's test was used *post hoc* to determine statistical significance compared to Group 2. For tested time points, *p*-values for Dunnett's test are listed in Groups 1, 3, 4 and 5 columns.

[b] Parameter statistically analyzed using Kruskal-Wallis one-way ANOVA on ranks (*p*-value listed in Group 2 column). No statistically significant differences were seen between groups; therefore, a *post hoc* test was not run on group means for indicated time points.

* Result statistically significant ($p < 0.05$) compared to Group 2 (Dunnett's test).

NOTES:
[1] N = 3
[2] "—" = No *p*-value calculated/a *post hoc* test was not performed.

Table 9b – Summary of Respiratory Rate Data (vs. Group 2)
(Breaths/Minute)

FEMALES

Study Day	Group 1 (Saline Control)			Group 2 (Vehicle Control)			Group 3 (APN01; 0.019 mg/L)			Group 4 (APN01; 0.038 mg/L)			Group 5 (APN01; 0.075 mg/L)		
	**Mean**	S.D.	*p*-value	**Mean**	S.D.	*p*-value	**Mean**	S.D.	*p*-value	**Mean**	S.D.	*p*-value	**Mean**	S.D.	*p*-value
1[a]	**58.5**	29.6	—	**44.4**	6.8	0.379	**54.7**	23.6	—	**95.6**	55.7	—	**55.4**	5.9	—
2[a]	**49.2**	22.5	—	**89.7**	14.6	0.203	**93.1**	90.0	—	**199.6**	131.4	—	**86.0**	19.5	—
3[a]	**110.8**	100.1	—	**55.9**	17.8	0.813	**125.5**	149.6	—	**118.4**	51.2	—	**71.6**	37.6	—
4[a]	**100.3**	95.7	—	**65.9**	14.6	0.239	**72.8**	39.5	—	**197.4**	76.2	—	**109.3**	92.4	—
5[a]	**64.9**	41.1	—	**65.3**	19.0	0.109	**65.4**	25.1	—	**127.3**	27.2	—	**66.6**	34.0	—
6[a]	**110.0**	75.6	—	**64.8**	34.8	0.053	**57.7**	10.4	—	**181.1**	61.2	—	**69.2**	30.0	—
7[a]	**51.3**	1.4	—	**83.7**	3.5	0.437	**144.6**	138.0	—	**164.5**	93.1	—	**96.2**	55.3	—
8[a]	**138.3**	106.8	—	**74.4**	44.3	0.422	**121.7**	109.2	—	**194.6**	76.4	—	**86.2**	27.8	—
9[b]	**78.5**	35.3	—	**143.4**	162.0	0.894	**136.4**	131.0	—	**154.4**	136.2	—	**92.0**	39.3	—
10[a]	**83.6**	61.2	—	**101.9**	86.9	0.486	**121.6**	118.3	—	**170.1**	62.7	—	**57.2**	9.1	—
11[a]	**87.5**	57.1	—	**167.1**	63.6	0.808	**127.5**	128.6	—	**119.0**	67.1	—	**105.0**	73.3	—
12[a]	**52.3**	7.9	—	**152.6**	166.8	0.645	**129.3**	129.6	—	**166.7**	71.4	—	**91.2**	34.9	—
13[b]	**78.0**	31.1	—	**55.8**	7.4	0.258	**58.8**	8.3	—	**182.1**	186.0	—	**70.3**	15.0	—
14[b]	**65.6**	55.7	—	**50.7**	17.3	0.244	**40.1**	19.7	—	**184.3**	126.5	—	**57.5**	15.0	—

[a] Parameter statistically analyzed using one-way ANOVA (*p*-value listed in Group 2 column). No statistically significant differences were seen between groups; therefore, a *post hoc* test was not run on group means for indicated time points.

[b] Parameter statistically analyzed using Kruskal-Wallis one-way ANOVA on ranks (*p*-value listed in Group 2 column). No statistically significant differences were seen between groups; therefore, a *post hoc* test was not run on group means for indicated time points.

NOTES:

[1] N = 3, except for Group 1 at Day 14 and Group 3 at Day 1, where N=2

[2] "—" = No *p*-value calculated/a *post hoc* test was not performed.

Table 10a – Summary of Tidal Volume Data (vs. Group 1)
(mL/breath)

MALES

Study Day	Group 1 (Saline Control)			Group 2 (Vehicle Control)			Group 3 (APN01; 0.019 mg/L)			Group 4 (APN01; 0.038 mg/L)			Group 5 (APN01; 0.075 mg/L)		
	**Mean**	S.D.	*p*-value	**Mean**	S.D.	*p*-value	**Mean**	S.D.	*p*-value	**Mean**	S.D.	*p*-value	**Mean**	S.D.	*p*-value
1[a]	**188**	37	0.039	**131**	51	0.703	**294**	53	0.231	**277**	67	0.363	**138**	108	0.779
2[a]	**248**	96	0.133	**174**	78	—	**353**	27	—	**196**	123	—	**122**	139	—
3[a]	**172**	38	0.225	**176**	42	—	**255**	58	—	**112**	100	—	**121**	109	—
4[a]	**209**	35	0.183	**144**	20	—	**150**	37	—	**117**	40	—	**181**	72	—
5[a]	**205**	64	0.490	**203**	48	—	**264**	146	—	**145**	74	—	**255**	62	—
6[a]	**263**	20	0.893	**202**	60	—	**256**	72	—	**204**	177	—	**226**	72	—
7[b]	**229**	39	0.083	**251**	214	—	**204**	46	—	**46**	21	—	**151**	50	—
8[a]	**173**	91	0.272	**140**	79	—	**152**	122	—	**85**	90	—	**259**	48	—
9[a]	**113**	73	0.217	**65**	71	—	**155**	93	—	**45**	4	—	**201**	127	—
10[b]	**200**	146	0.277	**186**	153	—	**299**	89	—	**99**	112	—	**207**	103	—
11[a]	**186**	82	0.071	**136**	20	—	**236**	63	—	**96**	52	—	**123**	39	—
12[a]	**197**	114	0.645	**109**	27	—	**166**	51	—	**123**	90	—	**197**	121	—
13[a]	**204**	59	0.010	**151**	97	0.576	**104**	31	0.132	**38***	19	0.011	**222**	6	0.977
14[a]	**222**	62	0.051	**161**	41	—	**225**	79	—	**34**	18	—	**189**	121	—

[a] Parameter analyzed for statistical significance using one-way ANOVA (*p*-value listed in Group 1 column). When one-way ANOVA results were statistically significant ($p < 0.05$), Dunnett's test was used *post hoc* to determine statistical significance compared to Group 1. For tested time points, *p*-values for Dunnett's test are listed in Groups 2-5 columns.

[b] Parameter statistically analyzed using Kruskal-Wallis one-way ANOVA on ranks (*p*-value listed in Group 1 column). No statistically significant differences were seen between groups; therefore, a *post hoc* test was not run on group means for indicated time points.

* Result statistically significant ($p < 0.05$) compared to Group 1 (Dunnett's test).

NOTES:

[1] N = 3

[2] "—" = No *p*-value calculated/a *post hoc* test was not performed.

Table 10a – Summary of Tidal Volume Data (vs. Group 1)
(mL/breath)

FEMALES

Study Day	Group 1 (Saline Control)			Group 2 (Vehicle Control)			Group 3 (APN01; 0.019 mg/L)			Group 4 (APN01; 0.038 mg/L)			Group 5 (APN01; 0.075 mg/L)		
	**Mean**	S.D.	*p*-value	**Mean**	S.D.	*p*-value	**Mean**	S.D.	*p*-value	**Mean**	S.D.	*p*-value	**Mean**	S.D.	*p*-value
1[a]	**171**	141	0.793	**251**	23	—	**133**	93	—	**163**	148	—	**206**	110	—
2[a]	**114**	61	0.641	**100**	41	—	**196**	151	—	**93**	94	—	**108**	60	—
3[a]	**140**	113	0.566	**192**	56	—	**147**	113	—	**152**	95	—	**263**	113	—
4[a]	**97**	29	0.316	**138**	49	—	**170**	67	—	**103**	70	—	**240**	159	—
5[a]	**143**	60	0.644	**182**	105	—	**200**	97	—	**113**	33	—	**166**	46	—
6[a]	**123**	61	0.236	**114**	29	—	**170**	15	—	**73**	47	—	**134**	63	—
7[a]	**109**	22	0.703	**74**	29	—	**188**	155	—	**121**	129	—	**169**	126	—
8[a]	**133**	152	0.510	**223**	131	—	**87**	89	—	**67**	61	—	**156**	110	—
9[a]	**138**	57	0.823	**162**	157	—	**218**	128	—	**139**	77	—	**201**	64	—
10[a]	**152**	69	0.254	**107**	27	—	**113**	79	—	**66**	41	—	**179**	68	—
11[a]	**147**	112	0.234	**46**	24	—	**92**	57	—	**111**	101	—	**212**	93	—
12[a]	**234**	77	0.274	**144**	119	—	**131**	67	—	**129**	80	—	**245**	49	—
13[a]	**121**	24	0.342	**226**	93	—	**138**	97	—	**116**	50	—	**129**	54	—
14[a]	**178**	99	0.820	**221**	85	—	**183**	137	—	**114**	114	—	**166**	95	—

[a] Parameter statistically analyzed using one-way ANOVA (*p*-value listed in Group 1 column). No statistically significant differences were seen between groups; therefore, no *post hoc* tests were run on group means for indicated time points.

NOTES:

[1] N = 3, except for Group 1 at Day 14 and Group 3 at Day 1, where N=2

[2] "—" = No *p*-value calculated/a *post hoc* test was not performed.

Table 10b – Summary of Tidal Volume Data (vs. Group 2)
(mL/breath)

MALES

Study Day	Group 1 (Saline Control)			Group 2 (Vehicle Control)			Group 3 (APN01; 0.019 mg/L)			Group 4 (APN01; 0.038 mg/L)			Group 5 (APN01; 0.075 mg/L)		
	**Mean**	S.D.	*p*-value	**Mean**	S.D.	*p*-value	**Mean**	S.D.	*p*-value	**Mean**	S.D.	*p*-value	**Mean**	S.D.	*p*-value
1[a]	**188**	37	0.703	**131**	51	0.039	**294***	53	0.046	**277**	67	0.077	**138**	108	1.000
2[a]	**248**	96	—	**174**	78	0.133	**353**	27	—	**196**	123	—	**122**	139	—
3[a]	**172**	38	—	**176**	42	0.225	**255**	58	—	**112**	100	—	**121**	109	—
4[a]	**209**	35	—	**144**	20	0.183	**150**	37	—	**117**	40	—	**181**	72	—
5[a]	**205**	64	—	**203**	48	0.490	**264**	146	—	**145**	74	—	**255**	62	—
6[a]	**263**	20	—	**202**	60	0.893	**256**	72	—	**204**	177	—	**226**	72	—
7[b]	**229**	39	—	**251**	214	0.083	**204**	46	—	**46**	21	—	**151**	50	—
8[a]	**173**	91	—	**140**	79	0.272	**152**	122	—	**85**	90	—	**259**	48	—
9[a]	**113**	73	—	**65**	71	0.217	**155**	93	—	**45**	4	—	**201**	127	—
10[b]	**200**	146	—	**186**	153	0.277	**299**	89	—	**99**	112	—	**207**	103	—
11[a]	**186**	82	—	**136**	20	0.071	**236**	63	—	**96**	52	—	**123**	39	—
12[a]	**197**	114	—	**109**	27	0.645	**166**	51	—	**123**	90	—	**197**	121	—
13[a]	**204**	59	0.576	**151**	97	0.010	**104**	31	0.679	**38**	19	0.081	**222**	6	0.337
14[a]	**222**	62	—	**161**	41	0.051	**225**	79	—	**34**	18	—	**189**	121	—

[a] Parameter analyzed for statistical significance using one-way ANOVA (*p*-value listed in Group 2 column). When one-way ANOVA results were statistically significant ($p < 0.05$), Dunnett's test was used *post hoc* to determine statistical significance compared to Group 2. For tested time points, *p*-values for Dunnett's test are listed in Groups 1, 3, 4 and 5 columns.

[b] Parameter statistically analyzed using Kruskal-Wallis one-way ANOVA on ranks (*p*-value listed in Group 2 column). No statistically significant differences were seen between groups; therefore, a *post hoc* test was not run on group means for indicated time points.

* Result statistically significant ($p < 0.05$) compared to Group 2 (Dunnett's test).

NOTES:

[1] N = 3

[2] "—" = No *p*-value calculated/a *post hoc* test was not performed.

Table 10b – Summary of Tidal Volume Data (vs. Group 2)
(mL/breath)

FEMALES

Study Day	Group 1 (Saline Control)			Group 2 (Vehicle Control)			Group 3 (APN01; 0.019 mg/L)			Group 4 (APN01; 0.038 mg/L)			Group 5 (APN01; 0.075 mg/L)		
	**Mean**	S.D.	*p*-value	**Mean**	S.D.	*p*-value	**Mean**	S.D.	*p*-value	**Mean**	S.D.	*p*-value	**Mean**	S.D.	*p*-value
1[a]	**171**	141	—	**251**	23	0.793	**133**	93	—	**163**	148	—	**206**	110	—
2[a]	**114**	61	—	**100**	41	0.641	**196**	151	—	**93**	94	—	**108**	60	—
3[a]	**140**	113	—	**192**	56	0.566	**147**	113	—	**152**	95	—	**263**	113	—
4[a]	**97**	29	—	**138**	49	0.316	**170**	67	—	**103**	70	—	**240**	159	—
5[a]	**143**	60	—	**182**	105	0.644	**200**	97	—	**113**	33	—	**166**	46	—
6[a]	**123**	61	—	**114**	29	0.236	**170**	15	—	**73**	47	—	**134**	63	—
7[a]	**109**	22	—	**74**	29	0.703	**188**	155	—	**121**	129	—	**169**	126	—
8[a]	**133**	152	—	**223**	131	0.510	**87**	89	—	**67**	61	—	**156**	110	—
9[a]	**138**	57	—	**162**	157	0.823	**218**	128	—	**139**	77	—	**201**	64	—
10[a]	**152**	69	—	**107**	27	0.254	**113**	79	—	**66**	41	—	**179**	68	—
11[a]	**147**	112	—	**46**	24	0.234	**92**	57	—	**111**	101	—	**212**	93	—
12[a]	**234**	77	—	**144**	119	0.274	**131**	67	—	**129**	80	—	**245**	49	—
13[a]	**121**	24	—	**226**	93	0.342	**138**	97	—	**116**	50	—	**129**	54	—
14[a]	**178**	99	—	**221**	85	0.820	**183**	137	—	**114**	114	—	**166**	95	—

[a] Parameter statistically analyzed using one-way ANOVA (*p*-value listed in Group 2 column). No statistically significant differences were seen between groups; therefore, no *post hoc* tests were run on group means for indicated time points.

NOTES:

[1] N = 3, except for Group 1 at Day 14 and Group 3 at Day 1, where N=2

[2] "—" = No *p*-value calculated/a *post hoc* test was not performed.

Table 11a – Summary of Minute Volume Data (vs. Group 1)
(mL/minute)

MALES

Study Day	Group 1 (Saline Control)			Group 2 (Vehicle Control)			Group 3 (APN01; 0.019 mg/L)			Group 4 (APN01; 0.038 mg/L)			Group 5 (APN01; 0.075 mg/L)		
	**Mean**	S.D.	*p*-value	**Mean**	S.D.	*p*-value	**Mean**	S.D.	*p*-value	**Mean**	S.D.	*p*-value	**Mean**	S.D.	*p*-value
1[a]	**7412**	5554	0.078	**6390**	2655	—	**14720**	1448	—	**12852**	3259	—	**8063**	4329	—
2[a]	**8150**	3407	0.123	**10969**	5976	—	**19491**	3167	—	**13482**	3857	—	**7601**	8717	—
3[a]	**6517**	1969	0.228	**12771**	4682	—	**11534**	6545	—	**12066**	3004	—	**5930**	4125	—
4[a]	**7240**	1872	0.646	**9595**	5934	—	**9906**	3402	—	**12293**	3400	—	**9933**	3654	—
5[a]	**8745**	3108	0.014	**9210**	4427	0.999	**17925***	2632	0.008	**13864**	985	0.142	**11713**	1375	0.529
6[a]	**10765**	6370	0.425	**10667**	1000	—	**16380**	1079	—	**17508**	9676	—	**11715**	3959	—
7[a]	**9384**	3495	0.474	**16806**	11151	—	**13160**	2428	—	**10533**	5064	—	**8204**	4187	—
8[b]	**12781**	8447	0.541	**10223**	3814	—	**13203**	3578	—	**21343**	22579	—	**15764**	1852	—
9[a]	**5809**	1501	0.319	**7235**	5228	—	**12256**	5846	—	**11918**	2555	—	**11948**	5868	—
10[a]	**8205**	4045	0.292	**10173**	6625	—	**19005**	10627	—	**7916**	4493	—	**11243**	4448	—
11[a]	**6729**	3927	0.194	**6327**	3979	—	**11312**	1048	—	**11027**	3295	—	**8659**	718	—
12[a]	**8080**	4863	0.253	**8116**	3800	—	**15012**	3191	—	**10430**	5499	—	**12266**	1900	—
13[a]	**6593**	2021	0.947	**8997**	7955	—	**9269**	5692	—	**8239**	6433	—	**10213**	4174	—
14[a]	**6509**	969	0.176	**8374**	6690	—	**14980**	3258	—	**6552**	3783	—	**10425**	4903	—

[a] Parameter analyzed for statistical significance using one-way ANOVA (*p*-value listed in Group 1 column). When one-way ANOVA results were statistically significant ($p < 0.05$), Dunnett's test was used *post hoc* to determine statistical significance compared to Group 1. For tested time points, *p*-values for Dunnett's test are listed in Groups 2-5 columns.

[b] Parameter statistically analyzed using Kruskal-Wallis one-way ANOVA on ranks (*p*-value listed in Group 1 column). No statistically significant differences were seen between groups; therefore, a *post hoc* test was not run on group means for indicated time points.

* Result statistically significant ($p < 0.05$) compared to Group 1 (Dunnett's test).

NOTES:

[1] N = 3

[2] "—" = No *p*-value calculated/a *post hoc* test was not performed.

Table 11a – Summary of Minute Volume Data (vs. Group 1)
(mL/minute)

FEMALES

Study Day	Group 1 (Saline Control)			Group 2 (Vehicle Control)			Group 3 (APN01; 0.019 mg/L)			Group 4 (APN01; 0.038 mg/L)			Group 5 (APN01; 0.075 mg/L)		
	**Mean**	S.D.	*p*-value	**Mean**	S.D.	*p*-value	**Mean**	S.D.	*p*-value	**Mean**	S.D.	*p*-value	**Mean**	S.D.	*p*-value
1[a]	**7954**	3405	0.662	**11252**	2680	—	**6163**	1930	—	**10333**	5465	—	**10884**	5864	—
2[a]	**5001**	2932	0.500	**9056**	5097	—	**9937**	3047	—	**10859**	1693	—	**8813**	6056	—
3[a]	**10081**	3596	0.075	**10982**	4949	—	**7224**	1989	—	**15227**	3727	—	**16003**	3609	—
4[a]	**7969**	5291	0.029	**8820**	2623	0.996	**11019**	3273	0.738	**16816**	3065	0.057	**17895***	4392	0.032
5[a]	**8767**	4677	0.655	**10785**	4637	—	**11487**	2265	—	**13777**	903	—	**10672**	5377	—
6[a]	**10385**	1285	0.621	**6690**	1627	—	**9783**	2291	—	**11347**	5482	—	**9279**	5263	—
7[a]	**5607**	1243	0.075	**6053**	2450	—	**13461**	6703	—	**11996**	3626	—	**11576**	1332	—
8[a]	**10341**	5063	0.599	**12774**	4404	—	**6365**	2969	—	**11086**	6695	—	**12045**	5672	—
9[a]	**10794**	5230	0.994	**10269**	7894	—	**8980**	4320	—	**9603**	4632	—	**10209**	3732	—
10[a]	**10168**	4336	0.997	**10269**	7894	—	**8980**	4320	—	**9603**	4632	—	**10209**	3732	—
11[b]	**9007**	821	0.147	**6504**	1612	—	**9284**	6188	—	**8885**	4689	—	**19290**	8181	—
12[a]	**11787**	1947	0.092	**13092**	6754	—	**11569**	6073	—	**17698**	1479	—	**21222**	3318	—
13[a]	**9006**	2418	0.420	**12222**	4020	—	**7738**	4701	—	**17501**	13293	—	**8556**	2060	—
14[a]	**8921**	3435	0.762	**12123**	7194	—	**6299**	5323	—	**11625**	7667	—	**8790**	4281	—

[a] Parameter analyzed for statistical significance using one-way ANOVA (*p*-value listed in Group 1 column). When one-way ANOVA results were statistically significant ($p < 0.05$), Dunnett's test was used *post hoc* to determine statistical significance compared to Group 1. For tested time points, *p*-values for Dunnett's test are listed in Groups 2-5 columns.

[b] Parameter statistically analyzed using Kruskal-Wallis one-way ANOVA on ranks (*p*-value listed in Group 1 column). No statistically significant differences were seen between groups; therefore, a *post hoc* test was not run on group means for indicated time points.

* Result statistically significant ($p < 0.05$) compared to Group 1 (Dunnett's test).

NOTES:

[1] N = 3, except for Group 1 at Day 14 and Group 3 at Day 1, where N=2

[2] "—" = No *p*-value calculated/a *post hoc* test was not performed.

Table 11b – Summary of Minute Volume Data (vs. Group 2)
(mL/minute)

MALES

Study Day	Group 1 (Saline Control)			Group 2 (Vehicle Control)			Group 3 (APN01; 0.019 mg/L)			Group 4 (APN01; 0.038 mg/L)			Group 5 (APN01; 0.075 mg/L)		
	**Mean**	S.D.	*p*-value	**Mean**	S.D.	*p*-value	**Mean**	S.D.	*p*-value	**Mean**	S.D.	*p*-value	**Mean**	S.D.	*p*-value
1[a]	**7412**	5554	—	**6390**	2655	0.078	**14720**	1448	—	**12852**	3259	—	**8063**	4329	—
2[a]	**8150**	3407	—	**10969**	5976	0.123	**19491**	3167	—	**13482**	3857	—	**7601**	8717	—
3[a]	**6517**	1969	—	**12771**	4682	0.228	**11534**	6545	—	**12066**	3004	—	**5930**	4125	—
4[a]	**7240**	1872	—	**9595**	5934	0.646	**9906**	3402	—	**12293**	3400	—	**9933**	3654	—
5[a]	**8745**	3108	0.999	**9210**	4427	0.014	**17925***	2632	0.011	**13864**	985	0.194	**11713**	1375	0.657
6[a]	**10765**	6370	—	**10667**	1000	0.425	**16380**	1079	—	**17508**	9676	—	**11715**	3959	—
7[a]	**9384**	3495	—	**16806**	11151	0.474	**13160**	2428	—	**10533**	5064	—	**8204**	4187	—
8[b]	**12781**	8447	—	**10223**	3814	0.541	**13203**	3578	—	**21343**	22579	—	**15764**	1852	—
9[a]	**5809**	1501	—	**7235**	5228	0.319	**12256**	5846	—	**11918**	2555	—	**11948**	5868	—
10[a]	**8205**	4045	—	**10173**	6625	0.292	**19005**	10627	—	**7916**	4493	—	**11243**	4448	—
11[a]	**6729**	3927	—	**6327**	3979	0.194	**11312**	1048	—	**11027**	3295	—	**8659**	718	—
12[a]	**8080**	4863	—	**8116**	3800	0.253	**15012**	3191	—	**10430**	5499	—	**12266**	1900	—
13[a]	**6593**	2021	—	**8997**	7955	0.947	**9269**	5692	—	**8239**	6433	—	**10213**	4174	—
14[a]	**6509**	969	—	**8374**	6690	0.176	**14980**	3258	—	**6552**	3783	—	**10425**	4903	—

[a] Parameter analyzed for statistical significance using one-way ANOVA (*p*-value listed in Group 2 column). When one-way ANOVA results were statistically significant ($p < 0.05$), Dunnett's test was used *post hoc* to determine statistical significance compared to Group 2. For tested time points, *p*-values for Dunnett's test are listed in Groups 1, 3, 4 and 5 columns.

[b] Parameter statistically analyzed using Kruskal-Wallis one-way ANOVA on ranks (*p*-value listed in Group 2 column). No statistically significant differences were seen between groups; therefore, a *post hoc* test was not run on group means for indicated time points.

* Result statistically significant ($p < 0.05$) compared to Group 2 (Dunnett's test).

NOTES:
[1] N = 3
[2] "—" = No *p*-value calculated/a *post hoc* test was not performed.

Table 11b – Summary of Minute Volume Data (vs. Group 2)
(mL/minute)

FEMALES

Study Day	Group 1 (Saline Control)			Group 2 (Vehicle Control)			Group 3 (APN01; 0.019 mg/L)			Group 4 (APN01; 0.038 mg/L)			Group 5 (APN01; 0.075 mg/L)		
	**Mean**	S.D.	*p*-value	**Mean**	S.D.	*p*-value	**Mean**	S.D.	*p*-value	**Mean**	S.D.	*p*-value	**Mean**	S.D.	*p*-value
1[a]	**7954**	3405	—	**11252**	2680	0.662	**6163**	1930	—	**10333**	5465	—	**10884**	5864	—
2[a]	**5001**	2932	—	**9056**	5097	0.500	**9937**	3047	—	**10859**	1693	—	**8813**	6056	—
3[a]	**10081**	3596	—	**10982**	4949	0.075	**7224**	1989	—	**15227**	3727	—	**16003**	3609	—
4[a]	**7969**	5291	0.996	**8820**	2623	0.029	**11019**	3273	0.889	**16816**	3065	0.088	**17895**	4392	0.051
5[a]	**8767**	4677	—	**10785**	4637	0.655	**11487**	2265	—	**13777**	903	—	**10672**	5377	—
6[a]	**10385**	1285	—	**6690**	1627	0.621	**9783**	2291	—	**11347**	5482	—	**9279**	5263	—
7[a]	**5607**	1243	—	**6053**	2450	0.075	**13461**	6703	—	**11996**	3626	—	**11576**	1332	—
8[a]	**10341**	5063	—	**12774**	4404	0.599	**6365**	2969	—	**11086**	6695	—	**12045**	5672	—
9[a]	**10794**	5230	—	**10269**	7894	0.994	**8980**	4320	—	**9603**	4632	—	**10209**	3732	—
10[a]	**10168**	4336	—	**10269**	7894	0.997	**8980**	4320	—	**9603**	4632	—	**10209**	3732	—
11[b]	**9007**	821	—	**6504**	1612	0.147	**9284**	6188	—	**8885**	4689	—	**19290**	8181	—
12[a]	**11787**	1947	—	**13092**	6754	0.092	**11569**	6073	—	**17698**	1479	—	**21222**	3318	—
13[a]	**9006**	2418	—	**12222**	4020	0.420	**7738**	4701	—	**17501**	13293	—	**8556**	2060	—
14[a]	**8921**	3435	—	**12123**	7194	0.762	**6299**	5323	—	**11625**	7667	—	**8790**	4281	—

[a] Parameter statistically analyzed using one-way ANOVA (*p*-value listed in Group 2 column). No statistically significant differences were seen between groups; therefore, a *post hoc* test was not run on group means for indicated time points.

[b] Parameter statistically analyzed using Kruskal-Wallis one-way ANOVA on ranks (*p*-value listed in Group 2 column). No statistically significant differences were seen between groups; therefore, a *post hoc* test was not run on group means for indicated time points.

NOTES:

[1] N = 3, except for Group 1 at Day 14 and Group 3 at Day 1, where N=2

[2] "—" = No *p*-value calculated/a *post hoc* test was not performed.

Table 12a – Summary of Peripheral Oxygen Saturation Data (vs. Group 1)
(SpO_2; %)

PRE-TEST (MALES)

		G 1 / M Saline	G 2 / M Vehicle	G 3 / M Low 0.019 mg/L	G 4 / M Mid 0.038 mg/L	G 5 / M High 0.075 mg/L
day -4	Mean	89k	88	92	88	89
	S.D.	7.2	7.8	5.7	1.2	12.1
	N	3	3	3	3	3
	P-Value	@0.8478				

k=KRUSKAL-WALLIS

PRE-TEST (FEMALES)

		G 1 / F Saline	G 2 / F Vehicle	G 3 / F Low 0.019 mg/L	G 4 / F Mid 0.038 mg/L	G 5 / F High 0.075 mg/L
day -5	Mean	84k	89	92	82	83
	S.D.	11.0	8.1	9.3	2.6	14.2
	N	3	3	3	3	3
	P-Value	@0.6474				

k=KRUSKAL-WALLIS

Table 12a – Summary of Peripheral Oxygen Saturation Data (vs. Group 1)
(SpO_2; %)

TREATMENT PERIOD (MALES)

		G 1 / M Saline	G 2 / M Vehicle	G 3 / M Low 0.019 mg/L	G 4 / M Mid 0.038 mg/L	G 5 / M High 0.075 mg/L
day 1	Mean	87 k	80	92	86	83
	S.D.	9.2	5.8	8.3	10.2	9.0
	N	3	3	3	3	3
	P-Value	@0.4042				
day 4	Mean	86 k	81	97	88	84
	S.D.	6.1	12.7	1.7	11.8	8.3
	N	3	3	3	3	3
	P-Value	@0.1180				
day 7	Mean	95 k	91	89	93	87
	S.D.	3.0	12.1	13.2	2.6	9.2
	N	3	3	3	3	3
	P-Value	@0.6030				
day 10	Mean	88 u	82	96	86	75
	S.D.	1.5	2.5	2.9	9.8	2.0
	N	3	3	3	3	3
	P-Value	@0.0244	1.0000	1.0000	1.0000	0.1110
day 14	Mean	85 a	90	92	83	84
	S.D.	11.6	9.1	10.4	9.1	11.2
	N	3	3	3	3	3
	P-Value	@0.7863				

k=KRUSKAL-WALLIS; u=KRUSKAL-WALLIS-DUNN; a=ANOVA

TREATMENT PERIOD (FEMALES)

		G 1 / F Saline	G 2 / F Vehicle	G 3 / F Low 0.019 mg/L	G 4 / F Mid 0.038 mg/L	G 5 / F High 0.075 mg/L
day 1	Mean	93 k	80	98	94	88
	S.D.	4.0	8.5	1.7	2.6	10.8
	N	3	3	3	3	3
	P-Value	@0.0628				
day 4	Mean	81 k	86	80	82	90
	S.D.	11.0	6.4	10.5	12.5	11.9
	N	3	3	3	3	3
	P-Value	@0.6014				
day 7	Mean	80 a	90	87	90	99
	S.D.	4.4	2.5	7.8	13.3	1.0
	N	3	3	3	3	3
	P-Value	@0.0929				
day 10	Mean	76 k	92	86	90	83
	S.D.	1.7	6.8	9.2	13.9	9.9
	N	3	3	3	3	3
	P-Value	@0.3794				

k=KRUSKAL-WALLIS; a=ANOVA

NOTE: Peripheral oxygen saturation measurements not collected from females on Day 14, see Protocol Deviation No. 1.

Table 12b – Summary of Peripheral Oxygen Saturation Data (vs. Group 2)
(SpO_2; %)

PRE-TEST (MALES)

		G 2 / M Vehicle	G 1 / M Saline	G 3 / M Low 0.019 mg/L	G 4 / M Mid 0.038 mg/L	G 5 / M High 0.075 mg/L
day -4	Mean	88k	89	92	88	89
	S.D.	7.8	7.2	5.7	1.2	12.1
	N	3	3	3	3	3
	P-Value	@0.8478				

k=KRUSKAL-WALLIS

PRE-TEST (FEMALES)

		G 2 / F Vehicle	G 1 / F Saline	G 3 / F Low 0.019 mg/L	G 4 / F Mid 0.038 mg/L	G 5 / F High 0.075 mg/L
day -5	Mean	89k	84	92	82	83
	S.D.	8.1	11.0	9.3	2.6	14.2
	N	3	3	3	3	3
	P-Value	@0.6474				

k=KRUSKAL-WALLIS

Table 12b – Summary of Peripheral Oxygen Saturation Data (vs. Group 2)
(SpO_2; %)

TREATMENT PERIOD (MALES)

		G 2 / M Vehicle		G 1 / M Saline	G 3 / M Low 0.019 mg/L	G 4 / M Mid 0.038 mg/L	G 5 / M High 0.075 mg/L
day 1	Mean	80	k	87	92	86	83
	S.D.	5.8		9.2	8.3	10.2	9.0
	N	3		3	3	3	3
	P-Value	@0.4042					
day 4	Mean	81	k	86	97	88	84
	S.D.	12.7		6.1	1.7	11.8	8.3
	N	3		3	3	3	3
	P-Value	@0.1180					
day 7	Mean	91	k	95	89	93	87
	S.D.	12.1		3.0	13.2	2.6	9.2
	N	3		3	3	3	3
	P-Value	@0.6030					
day 10	Mean	82	u	88	96	86	75
	S.D.	2.5		1.5	2.9	9.8	2.0
	N	3		3	3	3	3
	P-Value	@0.0244		1.0000	0.2166	1.0000	0.7969
day 14	Mean	90	a	85	92	83	84
	S.D.	9.1		11.6	10.4	9.1	11.2
	N	3		3	3	3	3
	P-Value	@0.7863					

k=KRUSKAL-WALLIS; u=KRUSKAL-WALLIS-DUNN; a=ANOVA

TREATMENT PERIOD (FEMALES)

		G 2 / F Vehicle		G 1 / F Saline	G 3 / F Low 0.019 mg/L	G 4 / F Mid 0.038 mg/L	G 5 / F High 0.075 mg/L
day 1	Mean	80	k	93	98	94	88
	S.D.	8.5		4.0	1.7	2.6	10.8
	N	3		3	3	3	3
	P-Value	@0.0628					
day 4	Mean	86	k	81	80	82	90
	S.D.	6.4		11.0	10.5	12.5	11.9
	N	3		3	3	3	3
	P-Value	@0.6014					
day 7	Mean	90	a	80	87	90	99
	S.D.	2.5		4.4	7.8	13.3	1.0
	N	3		3	3	3	3
	P-Value	@0.0929					
day 10	Mean	92	k	76	86	90	83
	S.D.	6.8		1.7	9.2	13.9	9.9
	N	3		3	3	3	3
	P-Value	@0.3794					

k=KRUSKAL-WALLIS; a=ANOVA

NOTE: Peripheral oxygen saturation measurements not collected from females on Day 14, see Protocol Deviation No. 1.

Table 13a – Summary of Venous Blood Oxygen Saturation Data (vs. Group 1)
(SvO_2; mmHg)

PRE-TEST (MALES)

		G 1 / M Saline	G 2 / M Vehicle	G 3 / M Low 0.019 mg/L	G 4 / M Mid 0.038 mg/L	G 5 / M High 0.075 mg/L
day -4	Mean	44.9 a	56.3	48.6	47.3	55.1
	S.D.	2.62	19.33	9.51	3.32	2.32
	N	3	3	3	3	3
	P-Value	@0.5672				

a=ANOVA

PRE-TEST (FEMALES)

		G 1 / F Saline	G 2 / F Vehicle	G 3 / F Low 0.019 mg/L	G 4 / F Mid 0.038 mg/L	G 5 / F High 0.075 mg/L
day -5	Mean	49.6 a	49.8	50.9	60.8	43.2
	S.D.	2.85	2.91	4.94	14.15	1.15
	N	3	3	3	3	3
	P-Value	@0.1125				

a=ANOVA

Table 13a – Summary of Venous Blood Oxygen Saturation Data (vs. Group 1)
(SvO_2; mmHg)

TREATMENT PERIOD (MALES)

		G 1 / M Saline	G 2 / M Vehicle	G 3 / M Low 0.019 mg/L	G 4 / M Mid 0.038 mg/L	G 5 / M High 0.075 mg/L
day 1	Mean	47.2 d	54.2	80.0 *	44.2	47.2
	S.D.	6.79	14.94	15.68	9.36	4.61
	N	3	3	3	3	3
	P-Value	@0.0158	0.8553	0.0157	0.9916	1.0000
day 4	Mean	62.5 k	64.6	55.6	49.2	55.9
	S.D.	12.98	1.13	16.42	8.20	1.93
	N	3	3	3	3	3
	P-Value	@0.2977				
day 7	Mean	71.1 a	64.7	48.0	67.6	52.6
	S.D.	17.28	5.20	5.87	7.70	5.25
	N	3	3	3	3	3
	P-Value	@0.0547				
day 10	Mean	71.1 a	52.5	59.1	50.8	64.6
	S.D.	9.62	3.94	13.77	16.20	6.16
	N	3	3	3	3	3
	P-Value	@0.2063				
day 14	Mean	53.3 k	59.5	53.2	131.9	56.3
	S.D.	10.95	5.97	5.66	17.69	10.28
	N	3	3	3	3	3
	P-Value	@0.0951				

d=ANOVA-DUNNETT; * = $p < 0.05$; k=KRUSKAL-WALLIS; a=ANOVA

TREATMENT PERIOD (FEMALES)

		G 1 / F Saline	G 2 / F Vehicle	G 3 / F Low 0.019 mg/L	G 4 / F Mid 0.038 mg/L	G 5 / F High 0.075 mg/L
day 1	Mean	56.0 a	81.1	67.6	43.4	56.0
	S.D.	5.15	49.50	8.84	9.49	8.39
	N	3	3	3	3	3
	P-Value	@0.4020				
day 4	Mean	98.2 a	68.2	86.6	54.1	66.4
	S.D.	24.46	14.96	22.95	11.00	10.83
	N	3	3	3	3	3
	P-Value	@0.0548				
day 7	Mean	107.9 k	77.9	89.1	62.1	79.4
	S.D.	47.60	24.48	15.78	20.44	17.09
	N	3	3	3	3	3
	P-Value	@0.6566				
day 10	Mean	61.8 k	59.6	58.7	45.7	77.3
	S.D.	10.11	9.27	10.92	5.52	20.20
	N	3	3	3	3	3
	P-Value	@0.1756				
day 14	Mean	84.6 k	117.9	78.3	136.5	69.9
	S.D.	5.28	38.74	22.02	36.32	10.21
	N	3	3	3	3	3
	P-Value	@0.0603				

a=ANOVA; k=KRUSKAL-WALLIS

Table 13b – Summary of Venous Blood Oxygen Saturation Data (vs. Group 2)
(SvO_2; mmHg)

PRE-TEST (MALES)

		G 2 / M Vehicle	G 1 / M Saline	G 3 / M Low 0.019 mg/L	G 4 / M Mid 0.038 mg/L	G 5 / M High 0.075 mg/L
day -4	Mean	56.3 a	44.9	48.6	47.3	55.1
	S.D.	19.33	2.62	9.51	3.32	2.32
	N	3	3	3	3	3
	P-Value	@0.5672				

a=ANOVA

PRE-TEST (FEMALES)

		G 2 / F Vehicle	G 1 / F Saline	G 3 / F Low 0.019 mg/L	G 4 / F Mid 0.038 mg/L	G 5 / F High 0.075 mg/L
day -5	Mean	49.8 a	49.6	50.9	60.8	43.2
	S.D.	2.91	2.85	4.94	14.15	1.15
	N	3	3	3	3	3
	P-Value	@0.1125				

a=ANOVA

Table 13b – Summary of Venous Blood Oxygen Saturation Data (vs. Group 2)
(SvO_2; mmHg)

TREATMENT PERIOD (MALES)

		G 2 / M Vehicle	G 1 / M Saline	G 3 / M Low 0.019 mg/L	G 4 / M Mid 0.038 mg/L	G 5 / M High 0.075 mg/L
day 1	Mean	54.2 d	47.2	80.0	44.2	47.2
	S.D.	14.94	6.79	15.68	9.36	4.61
	N	3	3	3	3	3
	P-Value	@0.0158	0.8553	0.0547	0.6598	0.8572
day 4	Mean	64.6 k	62.5	55.6	49.2	55.9
	S.D.	1.13	12.98	16.42	8.20	1.93
	N	3	3	3	3	3
	P-Value	@0.2977				
day 7	Mean	64.7 a	71.1	48.0	67.6	52.6
	S.D.	5.20	17.28	5.87	7.70	5.25
	N	3	3	3	3	3
	P-Value	@0.0547				
day 10	Mean	52.5 a	71.1	59.1	50.8	64.6
	S.D.	3.94	9.62	13.77	16.20	6.16
	N	3	3	3	3	3
	P-Value	@0.2063				
day 14	Mean	59.5 k	53.3	53.2	131.9	56.3
	S.D.	5.97	10.95	5.66	17.69	10.28
	N	3	3	3	3	3
	P-Value	@0.0951				

d=ANOVA-DUNNETT; k=KRUSKAL-WALLIS; a=ANOVA

TREATMENT PERIOD (FEMALES)

		G 2 / F Vehicle	G 1 / F Saline	G 3 / F Low 0.019 mg/L	G 4 / F Mid 0.038 mg/L	G 5 / F High 0.075 mg/L
day 1	Mean	81.1 a	56.0	67.6	43.4	56.0
	S.D.	49.50	5.15	8.84	9.49	8.39
	N	3	3	3	3	3
	P-Value	@0.4020				
day 4	Mean	68.2 a	98.2	86.6	54.1	66.4
	S.D.	14.96	24.46	22.95	11.00	10.83
	N	3	3	3	3	3
	P-Value	@0.0548				
day 7	Mean	77.9 k	107.9	89.1	62.1	79.4
	S.D.	24.48	47.60	15.78	20.44	17.09
	N	3	3	3	3	3
	P-Value	@0.6566				
day 10	Mean	59.6 k	61.8	58.7	45.7	77.3
	S.D.	9.27	10.11	10.92	5.52	20.20
	N	3	3	3	3	3
	P-Value	@0.1756				
day 14	Mean	117.9 k	84.6	78.3	136.5	69.9
	S.D.	38.74	5.28	22.02	36.32	10.21
	N	3	3	3	3	3
	P-Value	@0.0603				

a=ANOVA; k=KRUSKAL-WALLIS

Table 14a – Summary of Blood pH Data (vs. Group 1)

PRE-TEST (MALES)

		G 1 / M Saline	G 2 / M Vehicle	G 3 / M Low 0.019 mg/L	G 4 / M Mid 0.038 mg/L	G 5 / M High 0.075 mg/L
day -4	Mean	7.393 k	7.366	7.412	7.399	7.430
	S.D.	0.0277	0.0097	0.0371	0.0618	0.0178
	N	3	3	3	3	3
	P-Value	@0.2191				

k=KRUSKAL-WALLIS

PRE-TEST (FEMALES)

		G 1 / F Saline	G 2 / F Vehicle	G 3 / F Low 0.019 mg/L	G 4 / F Mid 0.038 mg/L	G 5 / F High 0.075 mg/L
day -5	Mean	7.393 a	7.368	7.336	7.393	7.392
	S.D.	0.0503	0.0248	0.0250	0.0154	0.0252
	N	3	3	3	3	3
	P-Value	@0.1641				

a=ANOVA

Table 14a – Summary of Blood pH Data (vs. Group 1)

TREATMENT PERIOD (MALES)

		G 1 / M Saline	G 2 / M Vehicle	G 3 / M Low 0.019 mg/L	G 4 / M Mid 0.038 mg/L	G 5 / M High 0.075 mg/L
day 1	Mean	7.337 k	7.365	7.318	7.334	7.351
	S.D.	0.0350	0.0320	0.0172	0.0384	0.0101
	N	3	3	3	3	3
	P-Value	@0.2706				
day 4	Mean	7.371 a	7.374	7.368	7.390	7.416
	S.D.	0.0185	0.0556	0.0419	0.0130	0.0244
	N	3	3	3	3	3
	P-Value	@0.4576				
day 7	Mean	7.357 a	7.397	7.352	7.410	7.346
	S.D.	0.0183	0.0191	0.0142	0.0083	0.0570
	N	3	3	3	3	3
	P-Value	@0.0710				
day 10	Mean	7.365 k	7.402	7.346	7.383	7.380
	S.D.	0.0230	0.0115	0.0473	0.0110	0.0335
	N	3	3	3	3	3
	P-Value	@0.1324				
day 14	Mean	7.357 k	7.431	7.349	7.376	7.366
	S.D.	0.0205	0.0285	0.0216	0.0318	0.0282
	N	3	3	3	3	3
	P-Value	@0.0691				

k=KRUSKAL-WALLIS; a=ANOVA

TREATMENT PERIOD (FEMALES)

		G 1 / F Saline	G 2 / F Vehicle	G 3 / F Low 0.019 mg/L	G 4 / F Mid 0.038 mg/L	G 5 / F High 0.075 mg/L
day 1	Mean	7.372 k	7.396	7.406	7.404	7.373
	S.D.	0.0195	0.0229	0.0131	0.0337	0.0156
	N	3	3	3	3	3
	P-Value	@0.2239				
day 4	Mean	7.399 a	7.380	7.453	7.411	7.411
	S.D.	0.0527	0.0316	0.0449	0.0036	0.0501
	N	3	3	3	3	3
	P-Value	@0.3346				
day 7	Mean	7.338 a	7.379	7.340	7.431	7.381
	S.D.	0.0750	0.0316	0.0116	0.0286	0.0345
	N	3	3	3	3	3
	P-Value	@0.1087				
day 10	Mean	7.328 a	7.383	7.348	7.375	7.379
	S.D.	0.0343	0.0161	0.0234	0.0240	0.0113
	N	3	3	3	3	3
	P-Value	@0.0624				
day 14	Mean	7.297 d	7.275	7.285	7.285	7.386
	S.D.	0.0636	0.0376	0.0193	0.0173	0.0420
	N	3	3	3	3	3
	P-Value	@0.0349	0.9030	0.9891	0.9879	0.0615

k=KRUSKAL-WALLIS; a=ANOVA; d=ANOVA-DUNNETT

Table 14b – Summary of Blood pH Data (vs. Group 2)

PRE-TEST (MALES)

		G 2 / M Vehicle	G 1 / M Saline	G 3 / M Low 0.019 mg/L	G 4 / M Mid 0.038 mg/L	G 5 / M High 0.075 mg/L
day -4	Mean	7.366 k	7.393	7.412	7.399	7.430
	S.D.	0.0097	0.0277	0.0371	0.0618	0.0178
	N	3	3	3	3	3
	P-Value	@0.2191				

k=KRUSKAL-WALLIS

PRE-TEST (FEMALES)

		G 2 / M Vehicle	G 1 / M Saline	G 3 / M Low 0.019 mg/L	G 4 / M Mid 0.038 mg/L	G 5 / M High 0.075 mg/L
day -4	Mean	7.366 k	7.393	7.412	7.399	7.430
	S.D.	0.0097	0.0277	0.0371	0.0618	0.0178
	N	3	3	3	3	3
	P-Value	@0.2191				

k=KRUSKAL-WALLIS

Table 14b – Summary of Blood pH Data (vs. Group 2)

TREATMENT PERIOD (MALES)

		G 2 / M Vehicle	G 1 / M Saline	G 3 / M Low 0.019 mg/L	G 4 / M Mid 0.038 mg/L	G 5 / M High 0.075 mg/L
day 1	Mean	7.365 k	7.337	7.318	7.334	7.351
	S.D.	0.0320	0.0350	0.0172	0.0384	0.0101
	N	3	3	3	3	3
	P-Value	@0.2706				
day 4	Mean	7.374 a	7.371	7.368	7.390	7.416
	S.D.	0.0556	0.0185	0.0419	0.0130	0.0244
	N	3	3	3	3	3
	P-Value	@0.4576				
day 7	Mean	7.397 a	7.357	7.352	7.410	7.346
	S.D.	0.0191	0.0183	0.0142	0.0083	0.0570
	N	3	3	3	3	3
	P-Value	@0.0710				
day 10	Mean	7.402 k	7.365	7.346	7.383	7.380
	S.D.	0.0115	0.0230	0.0473	0.0110	0.0335
	N	3	3	3	3	3
	P-Value	@0.1324				
day 14	Mean	7.431 k	7.357	7.349	7.376	7.366
	S.D.	0.0285	0.0205	0.0216	0.0318	0.0282
	N	3	3	3	3	3
	P-Value	@0.0691				

k=KRUSKAL-WALLIS; a=ANOVA

TREATMENT PERIOD (FEMALES)

		G 2 / F Vehicle	G 1 / F Saline	G 3 / F Low 0.019 mg/L	G 4 / F Mid 0.038 mg/L	G 5 / F High 0.075 mg/L
day 1	Mean	7.396 k	7.372	7.406	7.404	7.373
	S.D.	0.0229	0.0195	0.0131	0.0337	0.0156
	N	3	3	3	3	3
	P-Value	@0.2239				
day 4	Mean	7.380 a	7.399	7.453	7.411	7.411
	S.D.	0.0316	0.0527	0.0449	0.0036	0.0501
	N	3	3	3	3	3
	P-Value	@0.3346				
day 7	Mean	7.379 a	7.338	7.340	7.431	7.381
	S.D.	0.0316	0.0750	0.0116	0.0286	0.0345
	N	3	3	3	3	3
	P-Value	@0.1087				
day 10	Mean	7.383 a	7.328	7.348	7.375	7.379
	S.D.	0.0161	0.0343	0.0234	0.0240	0.0113
	N	3	3	3	3	3
	P-Value	@0.0624				
day 14	Mean	7.275 d	7.297	7.285	7.285	7.386 *
	S.D.	0.0376	0.0636	0.0193	0.0173	0.0420
	N	3	3	3	3	3
	P-Value	@0.0349	0.9030	0.9923	0.9932	0.0207

k=KRUSKAL-WALLIS; a=ANOVA; d=ANOVA-DUNNETT; * = p < 0.05

Table 15a – Summary of Absolute Organ Weight Data (vs. Group 1)

MALES

		G 1 / M Saline		G 2 / M Vehicle	G 3 / M Low 0.019 mg/L	G 4 / M Mid 0.038 mg/L	G 5 / M High 0.075 mg/L
Adrenals Wt	Mean	1.099	k	1.027	1.045	0.971	0.871
[g]	S.D.	0.0642		0.2391	0.1975	0.0858	0.1429
day 15	N	3		3	3	3	3
	P-Value	@0.3425					
Brain Wt	Mean	78.07	k	75.74	74.72	79.77	77.36
[g]	S.D.	5.525		9.058	3.912	3.065	21.362
day 15	N	3		3	3	3	3
	P-Value	@0.7174					
Heart Wt	Mean	69.54	k	71.58	101.66	71.47	77.85
[g]	S.D.	9.798		4.543	52.548	8.894	13.290
day 15	N	3		3	3	3	3
	P-Value	@0.9246					
Kidneys Wt	Mean	43.67	a	46.81	47.94	42.81	42.13
[g]	S.D.	5.126		6.570	4.423	1.145	1.213
day 15	N	3		3	3	3	3
	P-Value	@0.4206					
Liver Wt	Mean	269.27	k	240.31	272.97	263.39	238.08
[g]	S.D.	33.265		48.111	11.885	15.363	19.603
day 15	N	3		3	3	3	3
	P-Value	@0.4628					
Spleen Wt	Mean	42.733	a	59.597	52.617	49.427	50.557
[g]	S.D.	22.9754		19.6015	8.6758	13.2023	13.3839
day 15	N	3		3	3	3	3
	P-Value	@0.7959					
Testes Wt	Mean	13.61	a	12.39	16.17	10.43	11.08
[g]	S.D.	0.858		0.866	4.121	2.967	2.212
day 15	N	3		3	3	3	3
	P-Value	@0.1173					
Thymus Wt	Mean	7.425	a	5.012	4.370	7.159	7.561
[g]	S.D.	2.1135		0.9222	1.1916	3.2629	1.8849
day 15	N	3		3	3	3	3
	P-Value	@0.2469					
Epididymides Wt	Mean	2.565	k	2.785	3.059	1.902	2.507
[g]	S.D.	0.1651		0.2988	0.8903	0.4679	0.1139
day 15	N	3		3	3	3	3
	P-Value	@0.1193					

k=KRUSKAL-WALLIS; a=ANOVA

Table 15a – Summary of Absolute Organ Weight Data (vs. Group 1)

FEMALES

		G 1 / F Saline		G 2 / F Vehicle	G 3 / F Low 0.019 mg/L	G 4 / F Mid 0.038 mg/L	G 5 / F High 0.075 mg/L
Adrenals Wt	Mean	1.088	a	1.033	0.913	1.029	0.952
[g]	S.D.	0.2083		0.1829	0.0366	0.0700	0.1155
day 15	N	3		3	3	3	3
	P-Value	@0.5739					
Brain Wt	Mean	76.05	a	67.28	71.50	70.68	73.32
[g]	S.D.	1.953		7.182	5.939	3.056	2.321
day 15	N	3		3	3	3	3
	P-Value	@0.2743					
Heart Wt	Mean	69.98	a	64.58	60.55	65.94	64.22
[g]	S.D.	16.262		2.596	5.097	4.065	6.725
day 15	N	3		3	3	3	3
	P-Value	@0.7480					
Kidneys Wt	Mean	37.77	a	31.36	35.75	35.56	35.49
[g]	S.D.	5.291		1.901	6.148	2.910	7.614
day 15	N	3		3	3	3	3
	P-Value	@0.6690					
Liver Wt	Mean	222.54	a	201.12	205.04	202.94	201.59
[g]	S.D.	41.132		16.556	19.882	24.658	43.221
day 15	N	3		3	3	3	3
	P-Value	@0.9019					
Ovaries Wt	Mean	0.770	a	1.027	0.720	0.689	0.682
[g]	S.D.	0.2689		0.5880	0.1070	0.0333	0.0532
day 15	N	3		3	3	3	3
	P-Value	@0.5999					
Spleen Wt	Mean	50.912	k	45.243	43.222	36.521	47.023
[g]	S.D.	29.3744		15.4708	12.7812	9.4080	20.3171
day 15	N	3		3	3	3	3
	P-Value	@0.8726					
Thymus Wt	Mean	7.604	k	5.638	4.476	6.349	4.683
[g]	S.D.	3.1291		1.0701	1.0856	2.3384	1.1174
day 15	N	3		3	3	3	3
	P-Value	@0.3753					
Uterus Wt	Mean	1.73	a	5.55	2.78	2.49	2.34
[g]	S.D.	1.266		6.227	2.203	0.558	0.790
day 15	N	3		3	3	3	3
	P-Value	@0.6701					

a=ANOVA; k=KRUSKAL-WALLIS

Table 15b – Summary of Absolute Organ Weight Data (vs. Group 2)

MALES

		G 2 / M Vehicle	G 1 / M Saline	G 3 / M Low 0.019 mg/L	G 4 / M Mid 0.038 mg/L	G 5 / M High 0.075 mg/L
Adrenals Wt	Mean	1.027 k	1.099	1.045	0.971	0.871
[g]	S.D.	0.2391	0.0642	0.1975	0.0858	0.1429
day 15	N	3	3	3	3	3
	P-Value	@0.3425				
Brain Wt	Mean	75.74 k	78.07	74.72	79.77	77.36
[g]	S.D.	9.058	5.525	3.912	3.065	21.362
day 15	N	3	3	3	3	3
	P-Value	@0.7174				
Heart Wt	Mean	71.58 k	69.54	101.66	71.47	77.85
[g]	S.D.	4.543	9.798	52.548	8.894	13.290
day 15	N	3	3	3	3	3
	P-Value	@0.9246				
Kidneys Wt	Mean	46.81 a	43.67	47.94	42.81	42.13
[g]	S.D.	6.570	5.126	4.423	1.145	1.213
day 15	N	3	3	3	3	3
	P-Value	@0.4206				
Liver Wt	Mean	240.31 k	269.27	272.97	263.39	238.08
[g]	S.D.	48.111	33.265	11.885	15.363	19.603
day 15	N	3	3	3	3	3
	P-Value	@0.4628				
Spleen Wt	Mean	59.597 a	42.733	52.617	49.427	50.557
[g]	S.D.	19.6015	22.9754	8.6758	13.2023	13.3839
day 15	N	3	3	3	3	3
	P-Value	@0.7959				
Testes Wt	Mean	12.39 a	13.61	16.17	10.43	11.08
[g]	S.D.	0.866	0.858	4.121	2.967	2.212
day 15	N	3	3	3	3	3
	P-Value	@0.1173				
Thymus Wt	Mean	5.012 a	7.425	4.370	7.159	7.561
[g]	S.D.	0.9222	2.1135	1.1916	3.2629	1.8849
day 15	N	3	3	3	3	3
	P-Value	@0.2469				
Epididymides Wt	Mean	2.785 k	2.565	3.059	1.902	2.507
[g]	S.D.	0.2988	0.1651	0.8903	0.4679	0.1139
day 15	N	3	3	3	3	3
	P-Value	@0.1193				

k=KRUSKAL-WALLIS; a=ANOVA

Table 15b – Summary of Absolute Organ Weight Data (vs. Group 2)

FEMALES

		G 2 / F Vehicle	G 1 / F Saline	G 3 / F Low 0.019 mg/L	G 4 / F Mid 0.038 mg/L	G 5 / F High 0.075 mg/L
Adrenals Wt	Mean	1.033 a	1.088	0.913	1.029	0.952
[g]	S.D.	0.1829	0.2083	0.0366	0.0700	0.1155
day 15	N	3	3	3	3	3
	P-Value	@0.5739				
Brain Wt	Mean	67.28 a	76.05	71.50	70.68	73.32
[g]	S.D.	7.182	1.953	5.939	3.056	2.321
day 15	N	3	3	3	3	3
	P-Value	@0.2743				
Heart Wt	Mean	64.58 a	69.98	60.55	65.94	64.22
[g]	S.D.	2.596	16.262	5.097	4.065	6.725
day 15	N	3	3	3	3	3
	P-Value	@0.7480				
Kidneys Wt	Mean	31.36 a	37.77	35.75	35.56	35.49
[g]	S.D.	1.901	5.291	6.148	2.910	7.614
day 15	N	3	3	3	3	3
	P-Value	@0.6690				
Liver Wt	Mean	201.12 a	222.54	205.04	202.94	201.59
[g]	S.D.	16.556	41.132	19.882	24.658	43.221
day 15	N	3	3	3	3	3
	P-Value	@0.9019				
Ovaries Wt	Mean	1.027 a	0.770	0.720	0.689	0.682
[g]	S.D.	0.5880	0.2689	0.1070	0.0333	0.0532
day 15	N	3	3	3	3	3
	P-Value	@0.5999				
Spleen Wt	Mean	45.243 k	50.912	43.222	36.521	47.023
[g]	S.D.	15.4708	29.3744	12.7812	9.4080	20.3171
day 15	N	3	3	3	3	3
	P-Value	@0.8726				
Thymus Wt	Mean	5.638 k	7.604	4.476	6.349	4.683
[g]	S.D.	1.0701	3.1291	1.0856	2.3384	1.1174
day 15	N	3	3	3	3	3
	P-Value	@0.3753				
Uterus Wt	Mean	5.55 a	1.73	2.78	2.49	2.34
[g]	S.D.	6.227	1.266	2.203	0.558	0.790
day 15	N	3	3	3	3	3
	P-Value	@0.6701				

a=ANOVA; k=KRUSKAL-WALLIS

Table 16a – Summary of Relative Organ Weight (Organ-to-Body Weight Ratio) Data (vs. Group 1)

MALES

		G 1 / M Saline	G 2 / M Vehicle	G 3 / M Low 0.019 mg/L	G 4 / M Mid 0.038 mg/L	G 5 / M High 0.075 mg/L
Fasted Body Weight	Mean	9.03 a	8.81	8.71	8.67	8.69
[kg]	S.D.	0.970	0.582	0.202	0.491	0.353
day 15	N	3	3	3	3	3
	P-Value	@0.9317				
Adrenals	Mean	0.012 a	0.012	0.012	0.011	0.010
[%]	S.D.	0.0016	0.0020	0.0024	0.0004	0.0015
day 15	N	3	3	3	3	3
	P-Value	@0.5500				
Brain	Mean	0.87 a	0.86	0.86	0.92	0.89
[%]	S.D.	0.150	0.146	0.025	0.087	0.229
day 15	N	3	3	3	3	3
	P-Value	@0.9815				
Heart	Mean	0.77 a	0.81	1.16	0.83	0.90
[%]	S.D.	0.031	0.014	0.569	0.117	0.139
day 15	N	3	3	3	3	3
	P-Value	@0.4431				
Kidneys	Mean	0.49 a	0.53	0.55	0.50	0.49
[%]	S.D.	0.059	0.089	0.052	0.041	0.018
day 15	N	3	3	3	3	3
	P-Value	@0.5246				
Liver	Mean	2.98 a	2.74	3.13	3.05	2.74
[%]	S.D.	0.127	0.579	0.107	0.343	0.122
day 15	N	3	3	3	3	3
	P-Value	@0.4481				
Spleen	Mean	0.485 a	0.678	0.605	0.566	0.586
[%]	S.D.	0.2669	0.2265	0.1087	0.1285	0.1720
day 15	N	3	3	3	3	3
	P-Value	@0.8006				
Testes	Mean	0.15 k	0.14	0.19	0.12	0.13
[%]	S.D.	0.016	0.017	0.048	0.029	0.022
day 15	N	3	3	3	3	3
	P-Value	@0.1507				
Thymus	Mean	0.085 a	0.057	0.050	0.082	0.088
[%]	S.D.	0.0327	0.0086	0.0141	0.0369	0.0246
day 15	N	3	3	3	3	3
	P-Value	@0.3108				
Epididymides	Mean	0.029 a	0.032	0.035	0.022	0.029
[%]	S.D.	0.0047	0.0055	0.0108	0.0045	0.0025
day 15	N	3	3	3	3	3
	P-Value	@0.1901				

a=ANOVA; k=KRUSKAL-WALLIS

NOTE: Organ-to-body weight ratio = [absolute organ weight (g) ÷ fasted body weight (g)] × 100

Table 16a – Summary of Relative Organ Weight (Organ-to-Body Weight Ratio) Data (vs. Group 1)

FEMALES

		G 1 / F Saline	G 2 / F Vehicle	G 3 / F Low 0.019 mg/L	G 4 / F Mid 0.038 mg/L	G 5 / F High 0.075 mg/L
Fasted Body Weight	Mean	7.36 a	6.99	6.69	6.69	7.26
[kg]	S.D.	1.356	0.498	0.745	0.644	0.943
day 15	N	3	3	3	3	3
	P-Value	@0.8247				
Adrenals	Mean	0.015 k	0.015	0.014	0.016	0.013
[%]	S.D.	0.0010	0.0035	0.0018	0.0020	0.0008
day 15	N	3	3	3	3	3
	P-Value	@0.4678				
Brain	Mean	1.06 k	0.97	1.07	1.06	1.02
[%]	S.D.	0.186	0.163	0.091	0.074	0.145
day 15	N	3	3	3	3	3
	P-Value	@0.8384				
Heart	Mean	0.95 a	0.93	0.91	0.99	0.89
[%]	S.D.	0.147	0.044	0.042	0.126	0.052
day 15	N	3	3	3	3	3
	P-Value	@0.6806				
Kidneys	Mean	0.52 k	0.45	0.53	0.53	0.49
[%]	S.D.	0.038	0.051	0.032	0.032	0.043
day 15	N	3	3	3	3	3
	P-Value	@0.1377				
Liver	Mean	3.02 k	2.89	3.07	3.03	2.76
[%]	S.D.	0.025	0.300	0.145	0.116	0.318
day 15	N	3	3	3	3	3
	P-Value	@0.5804				
Ovaries	Mean	0.011 k	0.014	0.011	0.010	0.009
[%]	S.D.	0.0040	0.0071	0.0007	0.0010	0.0007
day 15	N	3	3	3	3	3
	P-Value	@0.4508				
Spleen	Mean	0.675 a	0.646	0.642	0.541	0.640
[%]	S.D.	0.3095	0.2140	0.1690	0.0908	0.2606
day 15	N	3	3	3	3	3
	P-Value	@0.9542				
Thymus	Mean	0.105 a	0.081	0.067	0.094	0.066
[%]	S.D.	0.0467	0.0146	0.0135	0.0307	0.0227
day 15	N	3	3	3	3	3
	P-Value	@0.4199				
Uterus	Mean	0.02 a	0.08	0.04	0.04	0.03
[%]	S.D.	0.012	0.080	0.028	0.007	0.011
day 15	N	3	3	3	3	3
	P-Value	@0.5374				

a=ANOVA; k=KRUSKAL-WALLIS

NOTE: Organ-to-body weight ratio = [absolute organ weight (g) ÷ fasted body weight (g)] × 100

Table 16b – Summary of Relative Organ Weight (Organ-to-Body Weight Ratio) Data (vs. Group 2)

MALES

		G 2 / M Vehicle	G 1 / M Saline	G 3 / M Low 0.019 mg/L	G 4 / M Mid 0.038 mg/L	G 5 / M High 0.075 mg/L
Fasted Body Weight	Mean	8.81 a	9.03	8.71	8.67	8.69
[kg]	S.D.	0.582	0.970	0.202	0.491	0.353
day 15	N	3	3	3	3	3
	P-Value	@0.9317				
Adrenals	Mean	0.012 a	0.012	0.012	0.011	0.010
[%]	S.D.	0.0020	0.0016	0.0024	0.0004	0.0015
day 15	N	3	3	3	3	3
	P-Value	@0.5500				
Brain	Mean	0.86 a	0.87	0.86	0.92	0.89
[%]	S.D.	0.146	0.150	0.025	0.087	0.229
day 15	N	3	3	3	3	3
	P-Value	@0.9815				
Heart	Mean	0.81 a	0.77	1.16	0.83	0.90
[%]	S.D.	0.014	0.031	0.569	0.117	0.139
day 15	N	3	3	3	3	3
	P-Value	@0.4431				
Kidneys	Mean	0.53 a	0.49	0.55	0.50	0.49
[%]	S.D.	0.089	0.059	0.052	0.041	0.018
day 15	N	3	3	3	3	3
	P-Value	@0.5246				
Liver	Mean	2.74 a	2.98	3.13	3.05	2.74
[%]	S.D.	0.579	0.127	0.107	0.343	0.122
day 15	N	3	3	3	3	3
	P-Value	@0.4481				
Spleen	Mean	0.678 a	0.485	0.605	0.566	0.586
[%]	S.D.	0.2265	0.2669	0.1087	0.1285	0.1720
day 15	N	3	3	3	3	3
	P-Value	@0.8006				
Testes	Mean	0.14 k	0.15	0.19	0.12	0.13
[%]	S.D.	0.017	0.016	0.048	0.029	0.022
day 15	N	3	3	3	3	3
	P-Value	@0.1507				
Thymus	Mean	0.057 a	0.085	0.050	0.082	0.088
[%]	S.D.	0.0086	0.0327	0.0141	0.0369	0.0246
day 15	N	3	3	3	3	3
	P-Value	@0.3108				
Epididymides	Mean	0.032 a	0.029	0.035	0.022	0.029
[%]	S.D.	0.0055	0.0047	0.0108	0.0045	0.0025
day 15	N	3	3	3	3	3
	P-Value	@0.1901				

a=ANOVA; k=KRUSKAL-WALLIS

NOTE: Organ-to-body weight ratio = [absolute organ weight (g) ÷ fasted body weight (g)] × 100

Table 16b – Summary of Relative Organ Weight (Organ-to-Body Weight Ratio) Data (vs. Group 2)

FEMALES

		G 2 / F Vehicle	G 1 / F Saline	G 3 / F Low 0.019 mg/L	G 4 / F Mid 0.038 mg/L	G 5 / F High 0.075 mg/L
Fasted Body Weight	Mean	6.99 a	7.36	6.69	6.69	7.26
[kg]	S.D.	0.498	1.356	0.745	0.644	0.943
day 15	N	3	3	3	3	3
	P-Value	@0.8247				
Adrenals	Mean	0.015 k	0.015	0.014	0.016	0.013
[%]	S.D.	0.0035	0.0010	0.0018	0.0020	0.0008
day 15	N	3	3	3	3	3
	P-Value	@0.4678				
Brain	Mean	0.97 k	1.06	1.07	1.06	1.02
[%]	S.D.	0.163	0.186	0.091	0.074	0.145
day 15	N	3	3	3	3	3
	P-Value	@0.8384				
Heart	Mean	0.93 a	0.95	0.91	0.99	0.89
[%]	S.D.	0.044	0.147	0.042	0.126	0.052
day 15	N	3	3	3	3	3
	P-Value	@0.6806				
Kidneys	Mean	0.45 k	0.52	0.53	0.53	0.49
[%]	S.D.	0.051	0.038	0.032	0.032	0.043
day 15	N	3	3	3	3	3
	P-Value	@0.1377				
Liver	Mean	2.89 k	3.02	3.07	3.03	2.76
[%]	S.D.	0.300	0.025	0.145	0.116	0.318
day 15	N	3	3	3	3	3
	P-Value	@0.5804				
Ovaries	Mean	0.014 k	0.011	0.011	0.010	0.009
[%]	S.D.	0.0071	0.0040	0.0007	0.0010	0.0007
day 15	N	3	3	3	3	3
	P-Value	@0.4508				
Spleen	Mean	0.646 a	0.675	0.642	0.541	0.640
[%]	S.D.	0.2140	0.3095	0.1690	0.0908	0.2606
day 15	N	3	3	3	3	3
	P-Value	@0.9542				
Thymus	Mean	0.081 a	0.105	0.067	0.094	0.066
[%]	S.D.	0.0146	0.0467	0.0135	0.0307	0.0227
day 15	N	3	3	3	3	3
	P-Value	@0.4199				
Uterus	Mean	0.08 a	0.02	0.04	0.04	0.03
[%]	S.D.	0.080	0.012	0.028	0.007	0.011
day 15	N	3	3	3	3	3
	P-Value	@0.5374				

a=ANOVA; k=KRUSKAL-WALLIS

NOTE: Organ-to-body weight ratio = [absolute organ weight (g) ÷ fasted body weight (g)] × 100

VI. APPENDICES

Appendix A – Protocol, Protocol Amendments and Protocol Deviations

PROTOCOL

1. **STUDY TITLE:** **Two-Week Aerosol Toxicity Study of APN01 in Dogs**

2. **IDENTIFICATION:**
IITRI Project No.: 285700300102
Contract No. 75N91019D00013
Task Order No. 75N91020F00002

3. **SPONSOR:**
Chemopreventive Agent Development Research Group
Division of Cancer Prevention
National Cancer Institute
9609 Medical Center Drive
Rockville, MD 20850

CONTRACTING OFFICER'S REPRESENTATIVE (COR):
Robert Shoemaker, Ph.D.
Phone: 240-276-7077
Email: robert.shoemaker@nih.gov

4. **TESTING FACILITY:**
IIT Research Institute (IITRI)
10 West 35th Street
Chicago, Illinois 60616

5. **PROPOSED TEST SITES:**

a. **TOXICOLOGIC PATHOLOGY:**
Charles River Laboratories, Inc.
8025 Lamon Avenue, Suite 447
Skokie, IL 60077

PROPOSED CONTRIBUTING SCIENTIST:
Carol Detrisac, D.V.M., Ph.D., D.A.C.V.P.
Tel: 312.567.4876
Email: carol.detrisac@crl.com

b. **TOXICOKINETIC ANALYSIS:**
R&D Services, LLC
10519 Reeder Street
Overland Park, KS 66214

PROPOSED CONTRIBUTING SCIENTIST:
Thomas N. Thompson, Ph.D.
Tel: (913) 481-4309
Email: tnt@rdservkc.com

6. **OBJECTIVE:**
The objective of this study is to provide data to support a comprehensive evaluation of the toxicity of twice daily aerosol administration of APN01 to dogs for fourteen consecutive days. The goals of this study include: (a) characterization of the toxicity of inhaled APN01 aerosols in a suitable non-rodent species (dogs); (b) characterization of plasma drug levels and toxicokinetics (TK) of inhaled APN01 in dogs; (c) identification of sensitive target tissues for the toxicity of inhaled APN01 aerosols in dogs; and (d) identification of a No Observed Adverse Effect Level [NO(A)EL] for repeat-dose inhalation administration of APN01 aerosols to dogs. The results of this study will provide a comprehensive preclinical toxicology and TK database in a suitable non-rodent species to support FDA submissions.

7. **IITRI SUPERVISORY STUDY PERSONNEL:**

a. **STUDY DIRECTOR:** Jeffrey W. Richig, D.V.M.
Phone: (312) 567-4904
Email: jrichig@iitri.org

b. **CONTRIBUTING INHALATION TOXICOLOGIST:** Sridhar Jaligama, Ph.D., D.A.B.T.
Phone: (312) 567-4285
Email: sjaligama@iitri.org

c. **CONTRIBUTING ANALYTICAL CHEMIST:** Miguel Muzzio, Ph.D., M.B.A.
Phone: (312) 567-4909
Email: mmuzzio@iitri.org

d. **PRINCIPAL INVESTIGATOR AND PRESIDENT:** David L. McCormick, Ph.D., D.A.B.T.
Phone: (312) 567-4972
Email: dmccormick@iitri.org

8. **PROPOSED SCHEDULE:**

a. **FIRST DAY OF EXPOSURE:** November 2, 2020 (males)
November 3, 2020 (females)

b. **COMPLETION OF IN-LIFE STUDY:** November 17, 2020

c. **DRAFT REPORT SUBMISSION:** January 25, 2021

9. **DURATION OF IN-LIFE STUDY:** Approximately 14 Days (+ quarantine)

10. **TEST AND CONTROL ARTICLES:**

a. **IDENTIFICATION:**

The test article is identified by the Sponsor as APN01, and will be supplied by Apeiron Biologics, Vienna, Austria. Prior to using the test article to generate test aerosols, 0.5 mL of PS80 concentrate (MRIGlobal, Kansas City, MO) will be added using sterile technique to each vial containing 4 mL APN01 solution. Two control articles will used in the study; Group 1 will be exposed to an aerosol containing sterile saline only, and Group 2 will be exposed to an aerosol containing the diluent used for APN01 (MRIGlobal, Kansas City, MO).

b. **HAZARDS TO PERSONNEL:**

Routine safety procedures used for handling of hazardous or potentially hazardous chemicals will be followed to ensure the health and safety of personnel handling the test article.

c. **TEST AND CONTROL ARTICLE CHARACTERIZATION:**

A Certificate of Analysis that includes the identity and concentration of vialed APN01 will be provided by or on behalf of the Sponsor. All characterization data (including method of synthesis, fabrication, or derivation of the test article) are maintained by the Sponsor or Sponsor's designee.

In addition, Certificates of Analysis or other documentation that include the identity and concentration of the PS80 concentrate and the diluent used for APN01 will be provided by on or behalf of the Sponsor. Sterile saline is a commercial product, and is characterized by its labeling.

d. **STORAGE:**

The bulk test article will be stored refrigerated (approximately 2-8 °C) in its original vials.

e. **SAMPLE DISPOSITION:**

All quantities of the test article that are dispensed will be documented.

f. **BASIS FOR SELECTION OF TEST ARTICLE EXPOSURE LEVELS:**

Test and control articles will be administered to dogs by oronasal inhalation. Inhalation is an intended clinical route of APN01 administration in humans.

The Maximum Feasible Concentration (MFC) was selected as the high dose for this study based on the results of a range-finding study of APN01 aerosols in dogs. Based on animal welfare considerations, the twice daily exposure period (60 minutes per exposure) is the maximum permissible exposure period for subchronic inhalation studies in dogs.

g. **DISPOSITION OF TEST ARTICLE:**

Upon completion of the study, the Sponsor will be contacted to determine the disposition of any remaining test article.

11. **EXPERIMENTAL DESIGN:**

Group	Number of Dogs (M + F)	Agent	Route of Administration	Number and Duration of Daily Exposures	Number of Exposure Days	Target APN01 Concentration
1	3 + 3	Saline	Inhalation	2 x 60 Minutes	14	0 (Control)
2	3 + 3	Diluent	Inhalation	2 x 60 Minutes	14	0 (Control)
3	3 + 3	APN01	Inhalation	2 x 60 Minutes	14	0.25 MFC*
4	3 + 3	APN01	Inhalation	2 x 60 Minutes	14	0.5 MFC*
5	3 + 3	APN01	Inhalation	2 x 60 Minutes	14	MFC*

* MFC to be documented in study records

12. **TEST SYSTEM:**

a. **TEST ANIMALS:**

Fifteen male and fifteen female beagle dogs (Ridglan Farms, Mt. Horeb, WI) were purchased for use in this study. All dogs were immunized (at a minimum) against distemper, type 2 adenovirus, parainfluenza, Bordetella, rabies, papilloma virus, and parvovirus by the supplier and were prophylactically administered broad-spectrum toltrazuril for coccidia. Dogs are at least five months old and will weigh approximately 6 to 10 kg on the first day of APN01 administration.

b. **JUSTIFICATION OF SPECIES SELECTED:**

The beagle dog is a standard non-rodent species that is accepted by the U.S. Food and Drug Administration (FDA) for studies of compounds used or intended for use in humans.

c. **JUSTIFICATION FOR THE NUMBER OF ANIMALS:**

The number of animals used is the minimum necessary to satisfy scientific principles, and was specified by the Sponsor. To the knowledge of the Sponsor and the Study Director, conduct of this study will result in no unnecessary duplication of existing data with regard to species, test article, exposure(s), routes, and duration of administration.

d. **HOUSING:**

Except for periods of inhalation exposure, dogs will be housed in pairs (by sex) in pens equipped with automatic watering systems. Pens will be cleaned daily. Dogs will be housed in accordance with U.S. Department of Agriculture Animal Welfare Standards (Title 9, *Code of Federal Regulations*, Part 3, 1991 Revision) and standards set forth in the *Guide for the Care and Use of Laboratory Animals* (National Research Council, 2011).

e. FOOD:

Approximately 400 g of Teklad Certified Canine Diet #2021C (Envigo, Madison WI) will be made available to each dog daily for a minimum of 1 hour. Each lot of diet is analyzed for contaminants to ensure that none are present at levels that would be expected to interfere with the purpose or conduct of this study. Analytical data from the lots of diet used in the study will be retained on file at IITRI.

f. WATER:

Except for periods of inhalation exposure, coarse-filtered City of Chicago water will be provided *ad libitum* to all animals using an automatic watering system. Water is analyzed periodically for bacterial contamination and chemical composition (*e.g.*, chemical contaminants, electrolytes, and metals). Water analysis records are retained on file at IITRI. No contaminants expected to interfere with the study are known to be present in the water.

g. ANIMAL IDENTIFICATION:

Each dog will be identified by a USDA tattoo number in the right or left ear. Each dog will also be assigned a unique number within the study. All pens will be identified by IITRI Project Number, Animal Number, and Sex.

h. ENVIRONMENTAL CONTROL:

Temperature and relative humidity in the animal room will be recorded manually each day. A 12-hour light/dark cycle (maintained with an automatic timer) will be used. Animal rooms will be generally held within temperature and relative humidity ranges as recommended by IITRI Standard Operating Procedures (SOPs).

13. METHOD:

a. QUARANTINE:

Each dog will be quarantined for a minimum of two weeks prior to test article exposure. Prior to study start, each dog will receive a detailed physical examination to ensure its suitability for use as a test animal. Animals will be released for use in the study by an IITRI Veterinarian.

b. ACCLIMATIZATION:

To condition animals to placement and restraint in the oronasal inhalation exposure systems, and to reduce stress during the exposure phase, each dog will be acclimated to the restraining sling and oronasal mask during pretreatment. Each dog will be acclimated sequentially for periods of 10 minutes, 20 minutes, and 40 minutes per day, and then returned to its run.

c. ADMINISTRATION:

At each exposure level, aerosolized APN01, saline, or diluent will be administered twice daily by oronasal inhalation using a mask-based delivery system that was custom-fabricated at IITRI. Twice daily exposures will be separated by a minimum of four hours.

1) **Test Atmosphere Generation:** APN01 will be administered as a liquid aerosol that is generated by nebulization of the formulation using Pari nebulizers that are integrated into the canine mask oronasal aerosol delivery system.

2) **Test Atmosphere Monitoring:** The aerosol mass concentration in the oronasal inhalation exposure system will be determined by collecting the aerosol on 47 mm diameter glass-fiber filters placed in closed-face filter holders at one exposure system port. Samples will be collected at a constant flow rate equal to the port flow of the delivery tube, and the total volume of air sampled will be measured by a dry gas meter. At least one aerosol sample will be collected during each exposure. Filter-collected samples will be weighed and selected filters will be extracted and analyzed by HPLC to quantitate APN01. In addition, the aerosol concentration in the oronasal inhalation exposure system will be monitored using an aerosol sensor. The aerosol

sensor will serve as real-time indicator of short-term concentration changes and will be used in guiding laboratory personnel if excursions from target concentrations are encountered. Aerosol particle size distribution will be monitored twice per each group during the study using a Quartz Crystal Microbalance (QCM) based cascade impactor (California Measurements, Sierra Madre, CA).

Temperature, relative humidity, and airflow in the inhalation exposure system will be recorded at least once during each exposure. To the maximum extent possible, the oronasal inhalation exposure chamber atmosphere temperature and relative humidity will be maintained in the ranges of 20 to 26°C and 30 to 70% RH. Airflow may be adjusted as a means of controlling test atmosphere concentration.

d. MORTALITY/MORIBUNDITY OBSERVATIONS:

Throughout the quarantine and exposure periods, all surviving animals will be observed at least twice daily for mortality or evidence of moribundity and to assess their general health. Any abnormal clinical signs will be recorded. Moribundity/mortality checks will be separated by a minimum of four hours.

e. MORIBUND ANIMALS:

During regularly scheduled mortality/moribundity observations, any animal judged not likely to survive until the next scheduled observation will, upon consent of the Attending Veterinarian and/or Study Director, be removed from the study, euthanized, and necropsied. These animals will be recorded in the study records as being euthanized *in extremis* and will be counted as a death for that day. Dead animals will be immediately removed for necropsy and the death will be recorded in the study records; the COR will be notified within 48 hours of any deaths or moribund sacrifices. If any death occurs overnight, the carcass will be stored refrigerated until the necropsy procedure can be performed.

f. INJURED OR DISEASED ANIMALS:

Animals on test will be treated for disease or injury using procedures that are consistent with normal veterinary medical practice. The circumstances and the disposition of any affected animals will be included in the study records. Any animals that pose a potential infectious threat to other study animals will be isolated.

g. PHYSICAL EXAMINATIONS AND CLINICAL OBSERVATIONS:

Detailed physical examinations and blood pressure measurements will be performed on all surviving dogs at least once during the pretest period and once on each day of exposure (after the first exposure to test or control article). Cage-side clinical observations will be performed daily during the exposure period at approximately one hour after the end of the first exposure.

h. BODY WEIGHT MEASUREMENTS:

All surviving dogs will be weighed at least once during pretest and twice weekly during the exposure period.

i. FOOD CONSUMPTION:

Food consumption will be measured for each pen at least once during pretest and daily during the treatment period.

j. OPHTHALMOLOGY:

Indirect funduscopic examinations will be performed on all surviving dogs during pretest and during the second week of exposure. Examinations will be performed at IITRI by a veterinarian.

k. ELECTROCARDIOGRAPHY (ECG):

ECG tracings will be obtained from all surviving dogs during pretest and during the second week of exposure. ECGs will be collected within approximately 2 hours after the first daily exposure.

ECG tracings will be evaluated by a veterinary cardiologist; ECG analyses will include heart rate and rhythm, and duration of the PR interval, QRS complex, and QT interval.

l. RESPIRATORY FUNCTION, TIDAL VOLUME, AND MINUTE VOLUME:

Respiration rate and tidal volume will be measured for each study animal once daily during the exposure period; minute volume will be calculated on the basis of these measurements. Oxygen saturation of the blood will be measured by pulse oximetry, blood pO_2 (using a pO_2 electrode), and blood pH will be measured pretest and on Days 1, 4, 7, 10 and 14 at the end of each exposure.

m. BLOOD OXYGEN SATURATION AND PH:

At least once during pretest and near the end of the first exposure period on exposure Days 1, 4, 7, 10, and 14, oxygen saturation of the blood will be measured by pulse oximetry; blood pO_2 will be determined using a pO_2 electrode; and blood pH will be determined.

n. FUNCTIONAL OBSERVATIONAL BATTERY:

A functional observation battery (FOB) will be performed on all surviving animals during pretest and during the second week of exposure. The FOB will include evaluations of body temperature; mental status; gait and posture; forelimb and hindlimb responses; neuromuscular function; and reflex responses.

o. SERUM DRUG LEVEL ANALYSIS AND TOXICOKINETICS

Blood samples (approximately 3 mL collected from the jugular or cephalic vein) for determination of serum levels of APN01 will be obtained from all surviving animals on Day 1 at pre-test (prior to the first exposure), 15 minutes, 30 minutes, 1, 2, and 4 hours after the first exposure and immediately prior to the second exposure. Blood samples will be collected into CAT Serum Sep Clot Activator tubes. Blood will be centrifuged; serum will be collected, frozen, and held at approximately -70°C until analyzed at IITRI using a validated bioanalytical method.

p. CLINICAL PATHOLOGY:

Blood for clinical pathology will be drawn from each surviving dog during pretest and prior to the terminal necropsy. Approximately 5 ml of blood will be collected from the jugular or cephalic vein at each time point. The following clinical pathology tests will be performed:

1) CLINICAL CHEMISTRY:

Blood urea nitrogen	Triglycerides	Total bilirubin
Creatinine	Cholesterol	Sodium
Urea nitrogen/creatinine ratio (calculated)	Creatine kinase	Potassium
Alkaline phosphatase	Total protein	Chloride
Alanine aminotransferase	Albumin (A)	Calcium
Aspartate aminotransferase	Globulin (G)	Phosphorus (inorganic)
Glucose	A/G ratio (calculated)	

2) HEMATOLOGY:

Erythrocyte count	Mean corpuscular hemoglobin
Erythrocyte morphology	Mean corpuscular hemoglobin concentration
Platelet count	Reticulocyte count (absolute and relative)
Hematocrit	Total white blood cell count
Hemoglobin	Differential white blood cell count (absolute and relative)
Mean corpuscular volume	

3) COAGULATION:

Fibrinogen	Prothrombin time	Activated partial thromboplastin time

q. NECROPSY AND TISSUES PRESERVED:

All study animals, whether found dead, euthanized moribund, or euthanized at the termination of the study, will receive a complete necropsy with tissue collection. On Study Day 15, surviving dogs will be anesthetized with sodium pentobarbital and euthanized by exsanguination for necropsy. Necropsy will include examination of the external surface of the body, all orifices, the cranial, thoracic and peritoneal cavities, and their contents. Tissues to be collected at necropsy are listed below and will be fixed in 10% neutral buffered formalin; at scheduled necropsies, eyes (with optic nerves) will be fixed in Davidson's solution; testes and epididymides will be fixed in modified Davidson's solution; and bone marrow smears will be fixed in methanol. Tissues identified with an asterisk will be weighed at scheduled necropsies.

Animal identification (ear with tattoo)	* Heart	Small intestine, duodenum
Artery, aorta	* Kidneys (paired)	Small intestine, ileum
Bone, femur (with epiphyseal plate of head)	Large intestine, cecum	Small intestine, jejunum
	Large intestine, colon	Spinal cord, cervical
Bone marrow, sternum	Large intestine, rectum	Spinal cord, lumbar
* Brain	* Liver	Spinal cord, thoracic
Cervix	Lung	* Spleen
* Epididymis (paired)	Lymph node, bronchial	Stomach
Esophagus	Lymph node, mandibular	* Testis (paired)
Eye (paired)	Lymph node, mesenteric	* Thymus
Gall bladder	Muscle, skeletal	Tongue
* Gland, adrenal (paired)	Nerve, optic (paired)	Tonsil (paired)
Gland, mammary gland	Nerve, sciatic	Trachea
Gland, parathyroid (paired)	* Ovary (paired)	Urinary bladder
Gland, pituitary	Oviduct (paired)	Ureter
Gland, prostate	Pancreas	* Uterus
Gland, salivary gland	Skin, ventral abdomen	Vagina
Gland, thyroid		Gross Lesions (if any)

r. HISTOPATHOLOGIC EVALUATION:

The tissues and organs listed above (except ear with identification) from all animals will be embedded in paraffin, processed by routine histologic methods, stained with hematoxylin and eosin, and evaluated microscopically by a board-certified veterinary pathologist.

14. PROPOSED STATISTICAL ANALYSIS:

Descriptive statistics (mean and standard deviation) will be calculated. If a data set (*e.g.*, body weights, body weight changes, food consumption, clinical pathology, organ weights, etc.) is normally distributed and of equal variance, statistical comparisons will be conducted using one-way analysis of variance (ANOVA), with the *post-hoc* comparisons made using Dunnett's test. If the data set is not normally distributed and/or not of equal variance, statistical comparisons will be conducted using the nonparametric Kruskal-Wallis ANOVA and Dunn's test. Incidence data (*e.g.*, clinical signs, physical examinations) will be evaluated using Chi-square analysis and/or Fisher's Exact test. A minimum significance level of $p < 0.05$ will be used for all comparisons.

15. STUDY RECORDS:

Data captured electronically using ToxData® (*e.g.*, daily mortality/moribundity data, body weights, clinical observations, physical examinations, food consumption, clinical pathology data, organ weights) will be maintained within the computer system database. Electronic copies of ToxData® .htm files will be backed-up onto CD-ROM(s) and the disc(s) will be maintained with the raw data. A copy of the SEND (Standard for Exchange of Nonclinical Data) data set(s) will also be backed-up onto CD-ROM(s) and the disc(s) will be maintained with the raw data.

All original paper data generated at IITRI will be maintained in loose-leaf notebooks. Paper data to be maintained in loose-leaf notebooks will include, but not necessarily be limited to, the following:

- the original study protocol and any amendments and/or deviations;
- animal care records;
- test and control article data;
- blood collection data;
- dose formulation analysis data;
- necropsy data.

16. ALTERATION OF DESIGN:

Alterations in the Protocol may be made as the study progresses. No changes in the Protocol will be made without the specific written consent of the Sponsor.

17. REGULATORY STANDARDS AND COMPLIANCE:

This study will be conducted in compliance with current U.S. FDA Good Laboratory Practices for Nonclinical Laboratory Studies (21 CFR Part 58), with the exception that a retention sample of the control article will not be retained.

18. QUALITY ASSURANCE UNIT (QAU):

a. IITRI (TESTING FACILITY) QAU RESPONSIBILITIES:

Portions of the study performed at IITRI will be audited by the IITRI Quality Assurance Unit (QAU) to assure adherence with GLP regulations, adherence to the study protocol, and compliance with IITRI SOPs. The IITRI QAU will conduct periodic phase audits and draft final report audits to ensure data integrity. The IITRI QAU will be the Lead QAU over the entire study and will communicate with the Test Site QAU to ensure overall GLP compliance. The Study Director will assure the distribution of the signed study protocol (and protocol amendments, if any) to the designated responsible person at the Test Site.

b. TEST SITE QAU RESPONSIBILITIES:

The Toxicologic Pathology Test Site will be responsible for quality assurance of their portion of the protocol, and for adherence to GLP regulations and Test Site SOPs. The Test Site QAU will periodically submit copies of their inspection reports to their Contributing Scientist, Test Site Management, the Study Director, Testing Facility Management, and the Lead (IITRI) QAU. Any deviations from the protocol or Test Site SOPs will be documented at the Test Site, acknowledged by the Test Site Contributing Scientist, and reported to and acknowledged by the Study Director. Additionally, a copy of the protocol and amendments will be maintained and the signed study phase report will be submitted by the Test Site for inclusion in the IITRI final study report.

19. ANIMAL WELFARE COMPLIANCE STATEMENT:

This study will be performed in compliance with the Animal Welfare Act (Title 9, *Code of Federal Regulations*), U.S. Public Health Service Policy on Humane Care and Use of Laboratory Animals (NIH Office of Laboratory Animal Welfare, 2015), and the *Guide for the Care and Use of Laboratory Animals* (National Research Council, 2011). The Sponsor's signature on the study protocol documents, for the Study Director, the Sponsor's assurance that the study described in this protocol does not unnecessarily duplicate previous experiments, and that no known acceptable non-animal alternatives were available. Wherever possible, procedures used in this study have been designed to avoid or minimize discomfort, distress, and pain to animals. All methods to be used are described in this study protocol or in written laboratory standard operating procedures.

The Institutional Animal Care and Use Committee (IACUC) at IITRI has reviewed this protocol and deems its study design appropriate to meet the objectives of the study, while minimizing both pain and distress to the test animals (IITRI IACUC Approval No. 20-053).

20. REPORTS:

An audited draft version of the report will be prepared and submitted to the Sponsor for review. Information in the report will be included, but not necessarily be limited to, the following:

- Copy of the approved protocol, including any amendments and/or deviations
- Species and strain of animal used
- Toxic response data by sex and dose
- Date of death during the study or whether animals survived to termination
- The period of observation of each abnormal sign and its subsequent course
- Body weight and food consumption data
- Clinical pathology tests employed with results (IITRI Contributing Scientist report)
- Serum drug level data (Contributing Scientist report)
- Toxicokinetic data (Contributing Scientist report)
- Dose formulation analyses (Contributing Scientist report)
- Necropsy and histopathology findings (Test Site report)
- Detailed description of results, where appropriate

Following Sponsor review of the audited Draft Report, a Final Report including a SEND data set will be submitted to the Sponsor. The Final Report will contain the signatures of the IITRI Study Director and Division Manager and a statement prepared and signed by the IITRI Quality Assurance Unit. SEND (Standard for Exchange of Nonclinical Data) data set(s) will also be submitted to the Sponsor upon report finalization.

21. PROPOSED MAJOR COMPUTER SYSTEMS:

Major computer systems to be used by IITRI personnel will include, but may not be limited to:

- ToxData® data collection system (PDS Pathology Data Systems, Inc., Basel, Switzerland; version 3.0 or latest version) will be used to randomize animals into groups and collect/calculate in-life toxicology data (dose administration, mortality/moribundity observations, clinical observations, body weights, body weight changes, food consumption, animal room environmental data) and clinical pathology data.
- Waters Empower 3 software (Milford, MA) will be used for HPLC data acquisition, data processing and instrument control, and Microsoft Excel (Redmond, WA; version 2010) will be for data processing and statistical computations.
- Standard of Exchange of Nonclinical Data (SEND) data set(s) will be created using TranSEND (PDS Pathology Data Systems, Inc., Basel, Switzerland; version 1.2.2 or later).

All other computer systems used at IITRI will be documented in the study records and final report. All major computer systems to be used by each Test Site will be included in each Test Site's Final Report.

22. DATA RETENTION:

All raw data generated as a result of this study, specimens and a copy of the final report from the study will be archived at IITRI for a period of five years from the date of completion of the study. The Sponsor will be responsible for all costs associated with continued storage of the archival materials in the IITRI archives or for the shipment of these materials to another storage facility. The IITRI QAU will maintain a complete record of the disposition of all archival materials.

23. PERSONNEL:

Curricula vitae for all personnel involved in the execution of the study are on file at IITRI.

24. PROTOCOL APPROVAL:

This protocol complies with the specific requirements of the Sponsor.

Study Director:	Jeffrey W. Richig, D.V.M.	2020-10-28<br>Date
Principal Investigator and Director:	David L. McCormick, Ph.D., D.A.B.T.	2020-10-28<br>Date
Contracting Officer's Technical Representative:	Robert Shoemaker, Ph.D.	1/25/2021<br>Date

25. PROTOCOL REVIEW:

Quality Assurance Manager:	Glenn B. Miller, M.S., RQAP-GLP, RAC	2020-10-28<br>Date

PROTOCOL AMENDMENT

**Study Title:**	**Two-Week Aerosol Toxicity Study of APN01 in Dogs**
**IITRI Project No.:**	285700300102
**Protocol Amendment No.:**	1

13. METHOD:

l. RESPIRATORY FUNCTION, TIDAL VOLUME, AND MINUTE VOLUME:

Protocol states:

Respiration rate and tidal volume will be measured for each study animal once daily during the exposure period; minute volume will be calculated on the basis of these measurements. Oxygen saturation of the blood will be measured by pulse oximetry, blood pO_2 (using a pO_2 electrode), and blood pH will be measured pretest and on Days 1, 4, 7, 10 and 14 at the end of each exposure.

Delete:

Oxygen saturation of the blood will be measured by pulse oximetry, blood pO_2 (using a pO_2 electrode), and blood pH will be measured pretest and on Days 1, 4, 7, 10 and 14 at the end of each exposure.

o. SERUM DRUG LEVEL ANALYSIS AND TOXICOKINETICS

Protocol states:

Blood samples (approximately 3 mL collected from the jugular or cephalic vein) for determination of serum levels of APN01 will be obtained from all surviving animals on Day 1 at pre-test (prior to the first exposure), 15 minutes, 30 minutes, 1, 2, and 4 hours after the first exposure and immediately prior to the second exposure. Blood samples will be collected into CAT Serum Sep Clot Activator tubes. Blood will be centrifuged; serum will be collected, frozen, and held at approximately -70°C until analyzed at IITRI using a validated bioanalytical method.

Amend protocol to state:

Blood samples (approximately 3 mL collected from the jugular or cephalic vein) for determination of serum levels of APN01 will be obtained from all surviving animals at least once during pretest. On Days 1 and 14, blood samples will be collected from all surviving animals prior to the first daily exposure; at 15 minutes, 30 minutes, 1, 2, and 4 hours after the first daily exposure; and at 24 hours after the first daily exposure (immediately prior to the first exposure on Day 2 or prior to necropsy on Day 15). Blood samples will be collected into CAT Serum Sep Clot Activator tubes. Blood will be centrifuged, and serum will be collected, frozen, and held at approximately -70°C until analyzed at IITRI using a validated bioanalytical method.

p. CLINICAL PATHOLOGY:

Add:

4) URINALYSIS:

Appearance (clarity)	pH	Ketones
Color	Leukocytes	Urobilinogen
Volume	Nitrite	Bilirubin
Refractive index	Protein	Occult blood
Specific gravity	Glucose	Microscopic examination of sediment

Urine samples will be collected pre-test and directly from the urinary bladder at necropsy.

q. **NECROPSY AND TISSUES PRESERVED:**

Protocol states:

Tissues to be collected at necropsy are listed below and will be fixed in 10% neutral buffered formalin; at scheduled necropsies, eyes (with optic nerves) will be fixed in Davidson's solution; testes and epididymides will be fixed in modified Davidson's solution; and bone marrow smears will be fixed in methanol.

Amend protocol to state:

Tissues to be collected at necropsy are listed below and will be fixed in 10% neutral buffered formalin; at scheduled necropsies, eyes (with optic nerves) will be fixed in Davidson's solution; testes and epididymides will be fixed in modified Davidson's solution.

Add:

s. **ADDITIONAL ANALYSES:** In addition to blood samples collected for drug level analyses, blood samples (approximately 3 mL per sample) for possible additional analyses will be collected from all surviving dogs during pre-test and Study Week 2. Blood samples will be collected into CAT Serum Sep Clot Activator tubes. Blood will be centrifuged, and serum will be collected, frozen, and held at approximately -70°C for possible additional analyses.

Reasons for Amendment:

13.l. Delete a redundancy in oxygen saturation evaluation (covered in Section 13.m.)

13.o. Add Day 14 serum sample collection time points for toxicokinetic analysis.

13.p. Add urinalysis and time points.

13.q. Delete procedure for fixation of bone marrow smears; bone marrow smears will not be collected.

13.s. Add blood sample collection for possible additional analyses.

Protocol Amendment Approval:

Study Director: Jeffrey W. Richig (signature)
Jeffrey W. Richig, D.V.M. Date: 2020-11-13

Principal Investigator and President: (signature)
David L. McCormick, Ph.D., D.A.B.T. Date: 2020-11-13

Contracting Officer's Representative (COR): Robert U. Shoemaker (signature)
Robert Shoemaker, Ph.D. Date: 11/13/2020

Protocol Amendment Review:

Quality Assurance Manager: (signature)
Glenn B. Miller, M.S., R.Q.A.P., R.A.C. Date: 2020-11-13

IIT RESEARCH INSTITUTE

IITRI Project No. 285700300102
Protocol Amendment No. 2
Page 1 of 1

PROTOCOL AMENDMENT

Study Title: **Two-Week Aerosol Toxicity Study of APN01 in Dogs**
IITRI Project No.: 285700300102
Protocol Amendment No.: 2

5. PROPOSED TEST SITES:

Protocol states:

**b. TOXICOKINETIC ANALYSIS:**	R&D Services, LLC<br>10519 Reeder Street<br>Overland Park, KS 66214
**PROPOSED CONTRIBUTING SCIENTIST:**	Thomas N. Thompson, Ph.D.<br>Tel: (913) 481-4309<br>Email: tnt@rdservkc.com

Amend protocol to state:

**b. TOXICOKINETIC ANALYSIS:**	IIT Research Institute (IITRI)<br>10 West 35th Street<br>Chicago, IL 60616
**PROPOSED CONTRIBUTING SCIENTIST:**	Miguel Muzzio, Ph.D., M.B.A.<br>Tel: (312) 567-4909<br>Email: mmuzzio@iitri.org

Reason for Amendment:

Change in test site and contributing scientist for the toxicokinetic analysis.

Protocol Amendment Approval:

Study Director:	Jeffrey W. Richig, D.V.M.	Date: 2021-01-07
Principal Investigator and President:	David L. McCormick, Ph.D., D.A.B.T.	Date: 2021-01-07
Contracting Officer's Representative (COR):	Robert Shoemaker, Ph.D.	Date: 1/19/2021

Protocol Amendment Review:

Quality Assurance Manager:	Glenn B. Miller, M.S., R.Q.A.P., R.A.C.	Date: 2021-01-07

IIT RESEARCH INSTITUTE

PROTOCOL DEVIATION NO. 1

Study Title: **Two-Week Aerosol Toxicity Study of APN01 in Dogs**

Date/Period of Deviation: November 2, 2020

Section: **13.d. MORTALITY/MORIBUNDITY OBSERVATIONS:**

Nature of Deviation: The protocol states: "Throughout the quarantine and exposure periods, all surviving animals will be observed at least twice daily for mortality or evidence of moribundity and to assess their general health. Any abnormal clinical signs will be recorded. Moribundity/mortality checks will be separated by a minimum of four hours."

Mortality/moribundity observations were inadvertently not performed in the afternoon on November 2, 2020.

Effect on Study: This deviation is not expected to affect the integrity of the study since the animals were observed in the mornings of November 2 and 3, 2020, with no adverse observations of general health/survival.

Date/Period of Deviation: November 16, 2020

Section: **13.m. BLOOD OXYGEN SATURATION AND pH:**

Nature of Deviation: The protocol states: "At least once during pretest and near the end of the first exposure period on exposure Days 1, 4, 7, 10, and 14, oxygen saturation of the blood will be measured by pulse oximetry; blood pO_2 will be determined using a pO_2 electrode; and blood pH will be determined."

The pulse oximeter had a damaged cable between the sensor and device. The device read "SEN OFF" at all times, even when placed on the animal. Therefore, no pulse oximetry data was collected for female animals on Day 14 (November 16, 2020).

Effect on Study: This deviation is not expected to affect the integrity of the study since sufficient pulse oximetry data was collected during the study.

**Date/Period of Deviation:**	November 3, 2020
**Section:**	**21. PROPOSED MAJOR COMPUTER SYSTEMS:**
**Nature of Deviation:**	The protocol states: "ToxData® data collection system (PDS Pathology Data Systems, Inc., Basel, Switzerland; version 3.0 or latest version) will be used to randomize animals into groups and collect/calculate in-life toxicology data (dose administration, mortality/moribundity observations, clinical observations, body weights, body weight changes, food consumption, animal room environmental data) and clinical pathology data."<br>On the first day of female exposures, Group 2 animal 2ZRE inadvertently underwent exposure with the Group 3 animals. It received exposure in place of Group 3 animal 3FLE which had not yet undergone exposure. Therefore, those two animals were switched from their original groups to being identified as Group 3 animal 3ZRE and Group 2 animal 2FLE for the study, thereby preserving the exposure regimen for each animal. Animal 3ZRE was exposed exclusively in Group 3 and animal 2FLE was exposed exclusively in Group 2.
**Effect on Study:**	This deviation is not expected to affect the integrity of the study since the two animals received the proper exposures for the groups they were switched into.

**Date/Period of Deviation:**	October 28, 2020
**Section:**	**6. OBJECTIVE:**
**Nature of Deviation:**	The protocol states: "(b) characterization of plasma drug levels and toxicokinetics (TK) of inhaled APN01 in dogs;"<br>Serum rather than plasma drug levels were measured, as stated in protocol section 13.o.
**Effect on Study:**	This deviation is due to an error in the protocol and is not expected to affect the integrity of the study.

Date/Period of Deviation: November 2-4, 2020 and November 15-17, 2020

Section: **13.o. SERUM DRUG LEVEL ANALYSIS AND TOXICOKINETICS**

Nature of Deviation: The protocol (Protocol Amendment No. 1) states: "Blood samples (approximately 3 mL collected from the jugular or cephalic vein) for determination of serum levels of APN01 will be obtained from all surviving animals at least once during pretest. On Days 1 and 14, blood samples will be collected from all surviving animals prior to the first daily exposure; at 15 minutes, 30 minutes, 1, 2, and 4 hours after the first daily exposure; and at 24 hours after the first daily exposure (immediately prior to the first exposure on Day 2 or prior to necropsy on Day 15). Blood samples will be collected into CAT Serum Sep Clot Activator tubes. Blood will be centrifuged, and serum will be collected, frozen, and held at approximately -70°C until analyzed at IITRI using a validated bioanalytical method."

Only the 1-hour post-exposure serum samples were evaluated for Groups 1 and 2. In addition, samples were collected at 6-hours post-exposure on Day 1 and at 4-5 hours post-exposure on Day 14.

The Day 1 Group 2 (males) and Group 5 (males) 15 and 30 minute post-exposure samples were collected slightly outside ± 10% of the target time point (Group 2 = -3 minutes; Group 3 = +4 minutes).

Effect on Study: This deviation is not expected to affect the integrity of the study because the 1-hour samples were deemed sufficient for the control groups, the Day 1, 6-hour and Day 14, 4-5 hour time points were additional time points that were deemed beneficial to the study, and the noted Day 1 samples were collected only slightly outside of the target time points.

Date/Period of Deviation: Entire Study

Section: **12.d. HOUSING:**

Nature of Deviation: The protocol states: "Except for periods of inhalation exposure, dogs will be housed in pairs (by sex) in pens equipped with automatic watering systems."

Some dogs were single housed during the study due to the odd number of animals per sex per group (3/sex/group).

Effect on Study: This deviation is not expected to affect the integrity of the study since the dividers between cages are bars and the single-housed animals have visual contact with the neighboring dogs.

Study Director:

Jeffrey W. Richig (signature) — 2021-03-12

Jeffrey W. Richig, D.V.M. — Date

IITRI Project No. 285700300102
Protocol Deviation No. 2
Page 1 of 1

PROTOCOL DEVIATION NO. 2

Study Title: **Two-Week Aerosol Toxicity Study of APN01 in Dogs**

Date/Period of Deviation: November 3, 2020 and November 16, 2020

Section: **13.l. RESPIRATORY FUNCTION, TIDAL VOLUME, AND MINUTE VOLUME:**

Nature of Deviation: The protocol states: "Respiration rate and tidal volume will be measured for each study animal once daily during the exposure period; minute volume will be calculated on the basis of these measurements."

There was no reportable respiratory data for animal number 3FKE (Group 3, female) on Day 1 (November 3, 2020) and 1JSG (Group 1, female) on Day 14 (November 16, 2020) due to an unstable flow signal during the measurement period.

Effect on Study: This deviation is not expected to affect the integrity of the study due to the fact that the data that was not obtained, due to the unstable signal, only involved one animal at each time point. There was ample data available to report for this study.

Study Director:

Jeffrey W. Richig (signature) — 2021-04-09

Jeffrey W. Richig, D.V.M. — Date

IIT RESEARCH INSTITUTE

***Phase*: Quantitating APN01 in Test Atmosphere**	
***Phase Test Site*:**	***Contributing Scientist*:**
IIT Research Institute (IITRI)<br>10 West 35th Street<br>Chicago, IL 60616	Miguel Muzzio, Ph.D., M.B.A.<br>Manager, Analytical Chemistry Division

Appendix B – Test Atmosphere Analysis Report

TABLE OF CONTENTS

Page

CONTRIBUTING SCIENTIST REPORT SIGNATURE PAGE B-2

I. INTRODUCTION B-3

II. MATERIALS AND METHODS B-4

III. RESULTS B-5

IV. TABLES.. B-6

Table 1 – APN01 Concentration in Filter Extract Samples Collected from Aerosol Test Atmospheres B-7

Table 2a – Representative Standard Curve Concentrations B-8

Table 2b – Representative Quality Control Sample Concentrations................................ B-8

V. FIGURES B-9

Figure 1 – Representative Calibration Curve B-10

Figure 2 – Representative Chromatogram: APN01 Calibrator (117.6 µg/mL)............... B-11

Figure 3 – Representative Chromatogram: Group 1 (Saline Control) B-11

Figure 4 – Representative Chromatogram: Group 2 (Vehicle Control) B-12

Figure 5 – Representative Chromatogram: Group 3 (APN01; 0.019 mg/L)................... B-12

Figure 6 – Representative Chromatogram: Group 4 (APN01; 0.038 mg/L)................... B-13

Figure 7 – Representative Chromatogram: Group 5 (APN01; 0.075 mg/L)................... B-13

VI. REFERENCE STANDARD CERTIFICATE OF ANALYSIS B-14

CONTRIBUTING SCIENTIST REPORT SIGNATURE PAGE

Report Approval:

Miguel Muzzio, PhD, MBA
Contributing Scientist
Manager, Analytical Chemistry Division
IIT Research Institute

2021-04-20
Date

I. INTRODUCTION

APN01 is a recombinant soluble human angiotensin converting enzyme 2 (ACE2) being developed as an inhibitor of SARS-CoV-2 infection and its associated disease, COVID-19. This study (IITRI Project Number 285700300102) was performed to provide a comprehensive evaluation of the toxicity of twice daily inhalation administration of APN01 aerosols to dogs for 14 consecutive days. This contributing scientist report describes the test atmosphere analysis portion of the study. The study design is summarized below:

Group	Number of Dogs (M + F)	Agent	Number and Duration of Daily Exposures	Number of Exposure Days	Target APN01 Concentration in Test Atmosphere (mg/L)
1	3 + 3	Saline (Control)	2 x 60 minutes	14	0
2	3 + 3	Vehicle (Control)	2 x 60 minutes	14	0
3	3 + 3	APN01 – Low	2 x 60 minutes	14	0.019
4	3 + 3	APN01 – Mid	2 x 60 minutes	14	0.038
5	3 + 3	APN01 - High	2 x 60 minutes	14	0.075

The aerosol mass concentration in each oronasal inhalation exposure system was determined by collecting the aerosol on glass-fiber filters. Samples were collected at a constant flow rate equal to the port flow of the delivery tube, and the total volume of air samples was measured by a dry-gas meter. One aerosol sample per dose level was collected during each exposure. Filter samples transferred to Analytical Chemistry Department personnel from Inhalation Toxicology Department personnel were extracted with phosphate buffered saline (PBS) and stored refrigerated (approximately 4°C). Filter sample extracts were analyzed at IITRI within the established refrigerated stability period (see following section).

II. MATERIALS AND METHODS

Filter samples were analyzed for levels of APN01 using high performance liquid chromatography (HPLC) with UV wavelength detection according to a method validated at IITRI. Validation work was performed under IITRI Project Number 2857003DFAMV1, Method No. 201001 (“Determination of APN01 in Test Atmospheres Filter Samples by HPLC”). During method validation, quality control (QC) samples prepared in PBS at target APN01 concentrations of 49 and 245 µg/mL were demonstrated to be stable for at least 22 days when stored refrigerated (111% and 104% of the original value, respectively) and for at least 2 days when stored at room temperature (111% and 103% of the original value, respectively).

A. Reference Standard: The APN01 reference standard (ACE20620-B; Polymun Scientific, Klosterneuburg, Austria) was received at IITRI on October 8 and November 3, 2020, from Apeiron Biologics (Vienna, Austria), and was stored at 2-8°C. The APN01 reference standard was used as received for the preparation of calibration standards and quality control (QC) samples for the determination of APN01 in test atmosphere samples. A Certificate of Analysis for the reference standard is presented in Section VI.

B. Sample, Calibrator and QC Preparation: Filters were placed in tubes containing 10 mL of phosphate buffered saline (PBS). The tubes were capped and shaken for 30 minutes for extraction. Following centrifugation, an aliquot (0.25 to 1 mL) from each sample was transferred into an HPLC vial for instrumental analysis.

   For instrument calibration, primary standard solutions of APN01 with target concentrations of 490 µg/mL were prepared by dilution of 0.5 mL of the test article formulation (4.9 mg/mL ACE2 content per the Certificate of Analysis) in PBS in a 5 mL volumetric flask and filling to volume. Standard curve calibrators were prepared at target concentrations of 29.4, 44.1, 58.8, 117.6, 176.4, 264.6 and 352.8 µg/mL by diluting the 490 µg/mL primary standards with PBS. QC samples of APN01 in PBS were prepared at low (50 µg/mL) and high (250 µg/mL) target concentrations and were analyzed along with the filter samples.

C. Analytical Equipment and Conditions: Calibrator, QC and processed filter samples were analyzed by HPLC using the following equipment and conditions:

HPLC System:	Waters Alliance 2695
HPLC Detector:	Waters 2487 Dual λ Absorbance Detector
Data System:	Waters Empower3
HPLC Column:	Agilent PLRP-S (reversed phase); 300 Å; 250 × 4.6 mm; 8 µm
Column Temperature:	25° C
Sample Temperature:	4° C
Injection Volume:	5 µL
Flow Rate:	1.0 mL/minute
Mobile Phase (MP):	A: 0.1% trifluoroacetic acid in ASTM type I water<br>B: 0.1% trifluoroacetic acid in acetonitrile

Gradient:

Time (min.)	% MP A	% MP B
0	75	25
6	10	90
6.01	75	25
12	75	25

Run Time: 12 min
Wavelength: 220 nm

The retention time of APN01 was approximately 5 to 6 minutes. The calibration curve was calculated from the linear regression of the calibrators' peak areas versus their respective concentrations. The concentrations of test article in the processed filter samples were determined from each sample's peak area using the linear regression parameters derived from the calibration curves and correcting the resulting concentration by multiplying by the appropriate dilution factor, as applicable.

III. RESULTS

A. Test Atmosphere Analysis: Samples were analyzed in three analytical batches (on November 3, 10 and 17, 2020). Test atmosphere analysis results (expressed in mg of APN01 in the collection filter) are presented in Table 1. These results were used to determine the mg/L concentrations (reported separately; see Summary Table 1 in the main report and Appendix C, Table C-1).

Representative chromatograms for filter samples are presented in Figures 3, 4, 5, 6 and 7 (for Groups 1, 2, 3, 4 and 5, respectively).

B. Calibration and QC: The standard curves were linear; a representative calibration curve is shown in Figure 1. Calibrator and QC sample results were within acceptable limits (90-110% of the target values); representative results are shown in Table 2a and Table 2b, respectively.

A representative calibrator (117.6 µg/mL) chromatogram is presented in Figure 2.

IV. TABLES

Table 1 – APN01 Concentration in Filter Extract Samples Collected from Aerosol Test Atmospheres

Exposure Day	Date	Group (Treatment)				
		1	2	3	4	5
		Saline Control	Vehicle Control	APN01; 0.019 mg/L	APN01; 0.038 mg/L	APN01; 0.075 mg/L
		APN01 Amount (mg)				
1	2020-11-02 AM	BQL	BQL	0.37	0.52	1.46
	2020-11-02 PM	BQL	BQL	0.39	0.64	1.65
2	2020-11-03 AM	BQL	BQL	0.31	0.59	1.55
	2020-11-03 PM	BQL	BQL	0.51	0.56	1.56
3	2020-11-04 AM	BQL	BQL	0.37	0.71	1.54
	2020-11-04 PM	BQL	BQL	0.64	0.90	1.89
4	2020-11-05 AM	BQL	BQL	0.63	0.82	1.65
	2020-11-05 PM	BQL	BQL	0.43	0.82	1.62
5	2020-11-06 AM	BQL	BQL	0.60	0.84	1.72
	2020-11-06 PM	BQL	BQL	0.72	0.88	1.90
6	2020-11-07 AM	BQL	BQL	0.66	0.80	1.57
	2020-11-07 PM	BQL	BQL	0.45	0.93	1.81
7	2020-11-08 AM	BQL	BQL	0.77	0.97	1.76
	2020-11-08 PM	BQL	BQL	0.84	0.99	1.76
8	2020-11-09 AM	BQL	BQL	0.70	0.92	1.69
	2020-11-09 PM	BQL	BQL	1.05	0.97	1.56
9	2020-11-10 AM	BQL	BQL	0.70	0.96	1.53
	2020-11-10 PM	BQL	BQL	0.56	0.78	1.70
10	2020-11-11 AM	BQL	BQL	0.74	0.90	1.91
	2020-11-11 PM	BQL	BQL	0.62	0.60	1.83
11	2020-11-12 AM	BQL	BQL	0.67	0.78	1.74
	2020-11-12 PM	BQL	BQL	0.62	1.01	1.86
12	2020-11-13 AM	BQL	BQL	0.52	1.01	1.78
	2020-11-13 PM	BQL	BQL	0.76	0.97	1.87
13	2020-11-14 AM	BQL	BQL	0.66	0.80	1.64
	2020-11-14 PM	BQL	BQL	0.99	0.98	1.84
14	2020-11-15 AM	BQL	BQL	0.71	0.99	1.63
	2020-11-15 PM	BQL	BQL	0.83	1.02	1.97
15	2020-11-16 AM	BQL	BQL	0.75	1.15	1.87
	2020-11-16 PM	BQL	BQL	0.69	0.94	1.99

NOTES:

[1] BQL = below quantitation limit (0.29 mg/filter)

[2] Results (in mg) were used to determine the mg/L concentrations reported in Summary Table 1 of the main report and in Appendix C, Table C-1.

[3] Each group was exposed for 14 days; however, due to staggered dosing, aerosol samples were collected over 15 days.

Table 2a – Representative Standard Curve Concentrations

Date of Analysis: 2020-11-03		
**Standard Concentration (µg/mL)**	**Experimental Concentration (µg/mL)**	**Accuracy (%)**
29.40	30.7	**105**
44.10	44.5	**101**
58.80	58.3	**99.2**
117.6	116	**98.8**
176.4	176	**99.9**
264.6	264	**99.8**
352.8	354	**100**
y-intercept:	-4.35E+04	
slope:	4.07E+03	
r^2:	0.9999	

Table 2b – Representative Quality Control Sample Concentrations

Date of Analysis: 2020-11-03		
**QC Sample Concentration (µg/mL)**	**Experimental Concentration (µg/mL)**	**Accuracy (%)**
49.0	46.2	**94.3**
	46.2	**94.3**
	46.7	**95.3**
	46.9	**95.8**
245	231	**94.4**
	232	**94.8**
	233	**95.1**
	231	**94.3**

V. FIGURES

Figure 1 – Representative Calibration Curve

(2020-11-03)

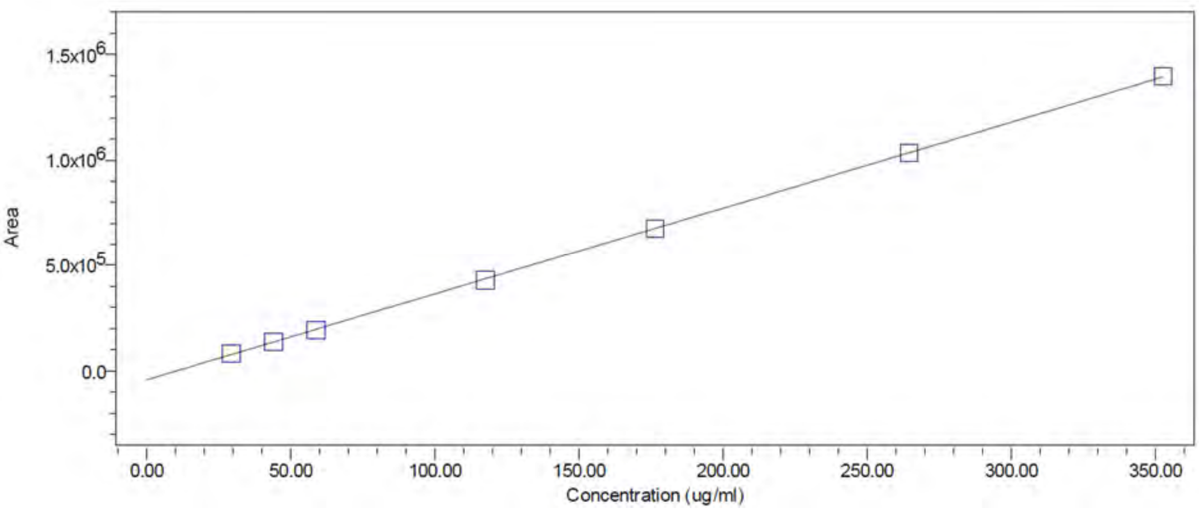

y-intercept:	-4.35E+04
Slope:	4.07E+03
r^{2}:	0.9999

Figure 2 – Representative Chromatogram: APN01 Calibrator (117.6 µg/mL)

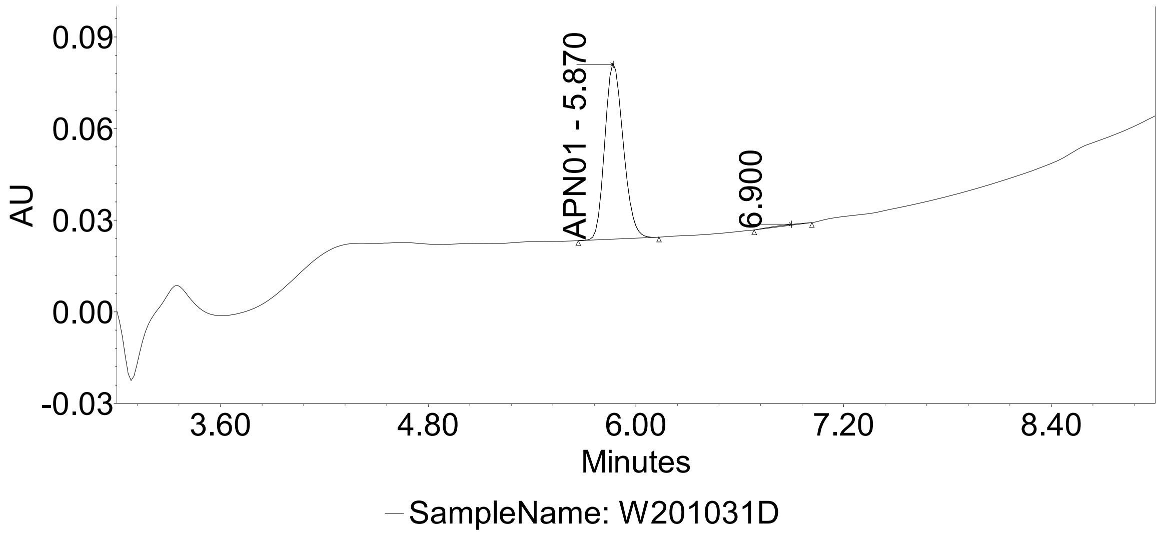

Figure 3 – Representative Chromatogram: Group 1 (Saline Control)

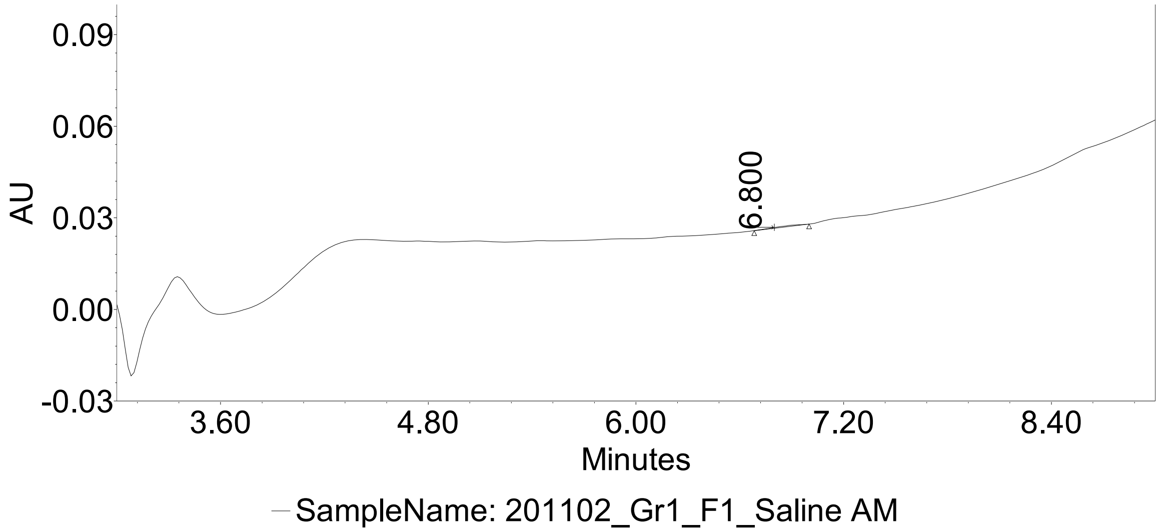

Figure 4 – Representative Chromatogram: Group 2 (Vehicle Control)

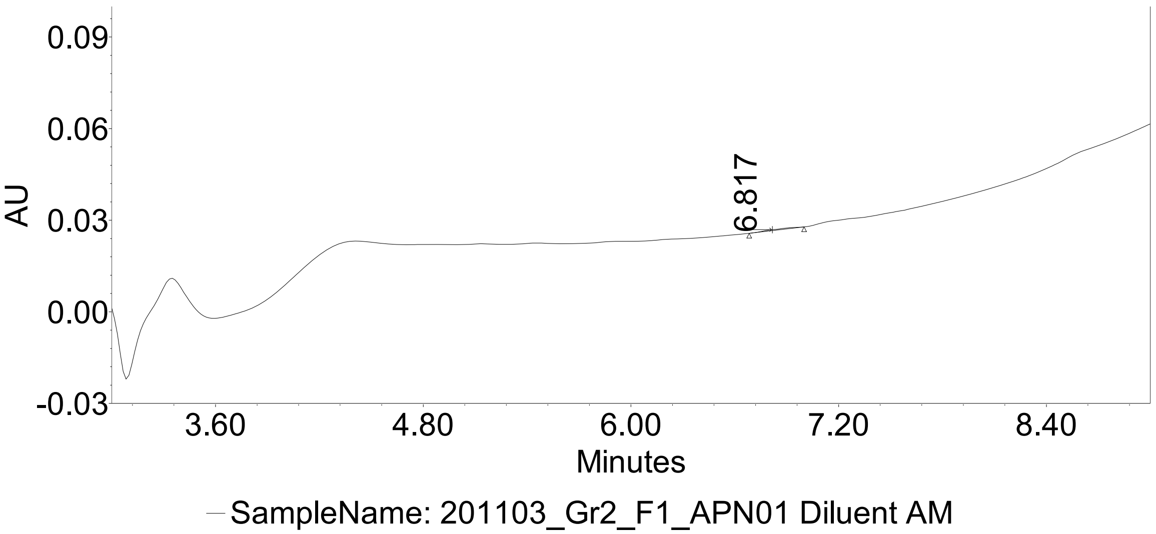

Figure 5 – Representative Chromatogram: Group 3 (APN01; 0.019 mg/L)

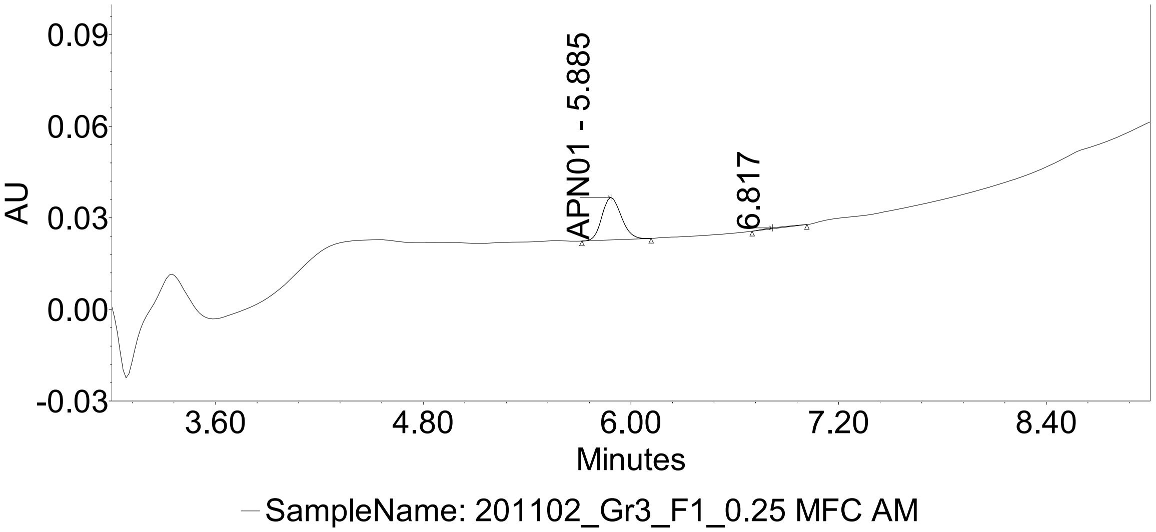

Figure 6 – Representative Chromatogram: Group 4 (APN01; 0.038 mg/L)

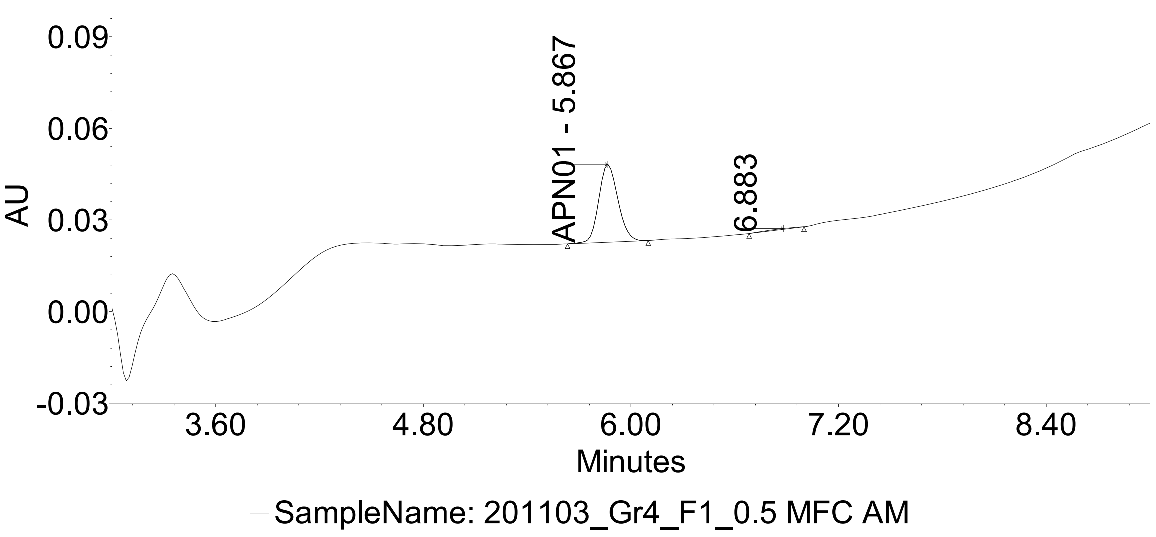

Figure 7 – Representative Chromatogram: Group 5 (APN01; 0.075 mg/L)

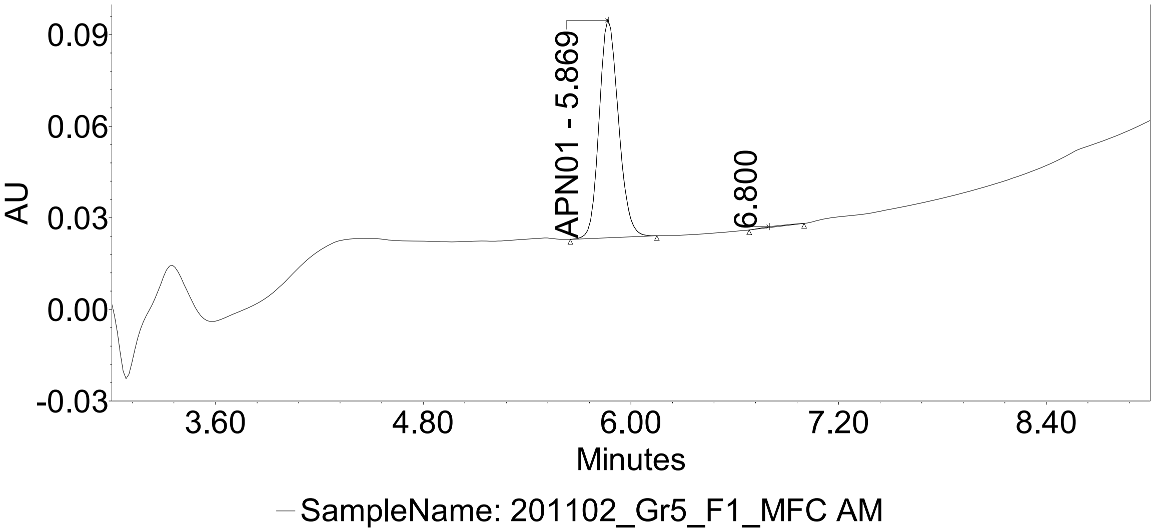

VI. REFERENCE STANDARD CERTIFICATE OF ANALYSIS

Reference Standard (APN01; ACE2 Drug Product) Certificate of Analysis (page 1 of 2)

POLYMUN SCIENTIFIC
Immunbiologische Forschung GmbH

A-3400 Klosterneuburg, Austria
Donaustraße 99
Tel.: +43-2243-25060-300
Fax: +43-2243-25060-399
E.Mail: office@polymun.com
http://www.polymun.com

CERTIFICATE OF ANALYSIS

**Product:** ACE2 Drug Product (5.0 mg/ml)	**Lot Number:** ACE20620-B
**Specification:** ACE2/SPEC/009-01	**Storage Conditions:** 5 ± 3 °C
**Date of Manufacture:** 09.06.2020	**Retest date:** 12/2020
**Site of Manufacture:** Polymun Scientific, Donaustraße 99, A-3400 Klosterneuburg, Austria	

Test Method Reference[1)]	Acceptance criteria	Result	Pass or Fail
**Appearance and Description**			
Visual appearance 224/SOP/011	clear and colourless	Clear, colourless	pass
Visible particles 224/SOP/011	practically free from visible particles	No visible particles	pass
**Identity**			
SDS-PAGE 223/SOP/002, 223/SOP/004	Protein band between 75 kD and 150 kD marker bands banding profile comparable to control standard	conforms	pass
Western Blot 223/SOP/002, 223/SOP/006	Protein band between 75 kD and 150 kD marker bands positive reaction of protein band with specific antibody banding profile comparable to control standard	conforms	pass
Native-PAGE 223/SOP/009, 223/SOP/004	Main band between 242 kD and 480 kD marker bands banding profile comparable to control standard	conforms	pass
Isoelectric Focussing 223/SOP/010, 223/SOP/004	Banding profile comparable to control standard bands between pI 4.2 and 5.3	conforms	pass
**Purity**			
Contents of dimers, di-dimers and polymers, monomers and fragments (SEC-HPLC) ACE2/SOP/007 [2)]	dimers ≥ 95.0 %<br>di-dimers + polymers ≤ 2.0 %<br>monomers ≤ 5.0 %<br>fragments ≤ 2.0 %<br>retention time of main peak comparable to that of control standard	dimers: 99.2 %<br>di-dimers: 0.1 %<br>monomers: 0.5 %<br>fragments: 0.2 %<br>retention time comparable to that of control standard	pass
QSDS-PAGE ACE2/SOP/001	Purity ≥ 95.0 %	98.9 %	pass
RP-HPLC ACE2/SOP/004	Main peak ≥ 90.0 %<br>Retention time of main peak comparable to that of control standard	99.8 %<br>retention time comparable to that of control standard	pass
**Quantity**			
ACE2 content (OD_{280}) 221/SOP/008	5.0 ± 0.5 mg/ml	4.9 mg/ml	pass
**Potency**			
Specific catalytic activity (HPLC) ACE2/SOP/003	0.025 – 0.075 μkat/mg	0.043 μkat/mg	pass

Version 01

Reference Standard (APN01; ACE2 Drug Product) Certificate of Analysis (page 2 of 2)

POLYMUN SCIENTIFIC
Immunbiologische Forschung GmbH

A-3400 Klosterneuburg, Austria
Donaustraße 99
Tel.: +43-2243-25060-300
Fax: +43-2243-25060-399
E.Mail: office@polymun.com
http://www.polymun.com

Test Method Reference[1]	Acceptance criteria	Result	Pass or Fail
**Contaminants**			
Endotoxin (LAL-assay) 221/SOP/001	≤ 10.42 EU/ml	< 0.15 EU/ml	pass
Sterility [3] USP <71>, Ph.Eur2.6.1	sterile	sterile	pass
**General characteristics**			
pH 224/SOP/016	7.0 ± 0.5	7.0	pass
Osmolality 224/SOP/009	330 ± 50 mOsmol/kg	327 mOsmol/kg	pass
Sialic acid (SIA) content (bound) ACE2/SOP/005	6.0 – 17.0 mol SIA/mol ACE2	10.3 mol SIA/mol ACE2	pass
Particulate Matter 224/SOP/018	Particles ≥ 10 µm: ≤6000 particles / container Particles ≥ 25 µm: ≤600 particles / container	Particles ≥ 10 µm: 26 particles / container Particles ≥ 25 µm: 3 particles / container	pass
Extractable Volume 224/SOP/014	Extractable volume (ml) is not less than the stated nominal volume	4.2 ml	pass

1. Unless specified otherwise, tests were performed at Polymun Scientific, Donaustraße 99, A-3400 Klosterneuburg, Austria.
2. Tested according to version 02 of the referenced SOP.
3. Test was performed at AGES, Institut für Med. Mikrobiologie und Hygiene, Beethovenstraße 6, A-8010 Graz, Austria.

The material was tested in compliance with GMP and conforms to the specification ACE2/SPEC/009-01 and.

Date: 12.10.2020

Quality Assurance / Qualified Person

Version 01 Page 2 of 2

Appendix C – Daily Exposure and Individual Animal Data

TABLE OF CONTENTS

Page

Table C-1. Daily Test Atmosphere Concentration Data C-2

Table C-2. Weekly Test Atmosphere Particle Size Distribution Data C-7

Table C-3. Daily Test Atmosphere Environmental Data C-8

Table C-4. Individual Animal Clinical Observations and Fates C-13

Table C-5. Individual Animal Detailed Physical Examination Observations C-15

Table C-6. Individual Animal Heart Rate and Blood Pressure Data C-17

Table C-7. Individual Animal Body Weight Data C-47

Table C-8. Individual Animal Body Weight Change Data C-49

Table C-9. Individual Animal Food Consumption Data C-51

Table C-10. Individual Animal Respiratory Function Data C-55

Table C-11. Individual Animal Peripheral Oxygen Saturation Data C-65

Table C-12. Individual Animal Venous Blood Oxygen Saturation Data C-69

Table C-13. Individual Animal Blood pH Data C-73

Table C-14. Individual Animal Absolute Organ Weight Data C-77

Table C-15. Individual Animal Relative Organ Weight (Organ-to-Body Weight Ratio) Data C-79

Table C-1 – Daily Test Atmosphere Concentration Data

Group (Treatment)	Exposure Day	Exposure Date	Volume (L)	Gravimetric Weight (mg)	Gravimetric Concentration (mg/L)	Analytical Concentration (mg/L)
1 (Saline Control)	**AM SAMPLE COLLECTION**					
	1	11/2/2020	23.619	197.92	8.38	0.000
	2	11/3/2020	12.687	89.62	7.06	0.000
	3	11/4/2020	18.606	112.73	6.06	0.000
	4	11/5/2020	16.709	90.78	5.43	0.000
	5	11/6/2020	15.774	80.88	5.13	0.000
	6	11/7/2020	16.652	102.84	6.18	0.000
	7	11/8/2020	15.661	79.10	5.05	0.000
	8	11/9/2020	17.445	91.07	5.22	0.000
	9	11/10/2020	17.587	81.28	4.62	0.000
	10	11/11/2020	17.757	91.33	5.14	0.000
	11	11/12/2020	17.615	86.20	4.89	0.000
	12	11/13/2020	17.530	91.63	5.23	0.000
	13	11/14/2020	17.162	82.51	4.81	0.000
	14	11/15/2020	16.822	79.07	4.70	0.000
	15	11/16/2020	16.284	82.03	5.04	0.000
					**Mean:**	**0.000**
					**SD:**	**0.0000**
					**N:**	**15**
	**PM SAMPLE COLLECTION**					
	1	11/2/2020	13.990	106.56	7.62	0.000
	2	11/3/2020	13.282	78.77	5.93	0.000
	3	11/4/2020	14.387	74.91	5.21	0.000
	4	11/5/2020	15.236	79.83	5.24	0.000
	5	11/6/2020	15.151	96.14	6.35	0.000
	6	11/7/2020	15.576	85.15	5.47	0.000
	7	11/8/2020	15.972	83.05	5.20	0.000
	8	11/9/2020	17.190	89.05	5.18	0.000
	9	11/10/2020	17.728	83.83	4.73	0.000
	10	11/11/2020	17.728	84.12	4.75	0.000
	11	11/12/2020	17.615	93.01	5.28	0.000
	12	11/13/2020	17.672	91.34	5.17	0.000
	13	11/14/2020	17.134	94.09	5.49	0.000
	14	11/15/2020	17.275	83.23	4.82	0.000
	15	11/16/2020	14.896	91.05	6.11	0.000
					**Mean:**	**0.000**
					**SD:**	**0.0000**
					**N:**	**15**

NOTES:

[1] Each study group was exposed for 14 days; however, due to staggered dosing for the two sexes, aerosol samples were collected over 15 days.

[2] Analytical concentration calculated based on HPLC analysis results (see Appendix B): mg/filter HPLC result/sample volume (L)

Table C-1 – Daily Test Atmosphere Concentration Data

Group (Treatment)	Exposure Day	Exposure Date	Volume (L)	Gravimetric Weight (mg)	Gravimetric Concentration (mg/L)	Analytical Concentration (mg/L)
2 (Vehicle Control)	AM SAMPLE COLLECTION					
	1	11/2/2020	23.760	187.11	7.88	0.000
	2	11/3/2020	13.650	55.52	4.07	0.000
	3	11/4/2020	14.415	77.91	5.40	0.000
	4	11/5/2020	14.925	81.65	5.47	0.000
	5	11/6/2020	11.696	73.35	6.27	0.000
	6	11/7/2020	15.095	85.13	5.64	0.000
	7	11/8/2020	16.256	80.05	4.92	0.000
	8	11/9/2020	17.247	117.59	6.82	0.000
	9	11/10/2020	17.757	101.90	5.74	0.000
	10	11/11/2020	17.728	87.19	4.92	0.000
	11	11/12/2020	17.530	156.49	8.93	0.000
	12	11/13/2020	17.615	134.32	7.63	0.000
	13	11/14/2020	17.077	115.41	6.76	0.000
	14	11/15/2020	16.624	120.32	7.24	0.000
	15	11/16/2020	21.098	103.48	4.90	0.000
					**Mean:**	**0.000**
					**SD:**	**0.0000**
					**N:**	**15**
	PM SAMPLE COLLECTION					
	1	11/2/2020	12.291	84.06	6.84	0.000
	2	11/3/2020	14.273	79.22	5.55	0.000
	3	11/4/2020	13.084	80.45	6.15	0.000
	4	11/5/2020	14.953	80.48	5.38	0.000
	5	11/6/2020	15.859	91.39	5.76	0.000
	6	11/7/2020	15.944	97.35	6.11	0.000
	7	11/8/2020	15.859	111.36	7.02	0.000
	8	11/9/2020	18.465	88.89	4.81	0.000
	9	11/10/2020	18.153	98.71	5.44	0.000
	10	11/11/2020	17.700	133.03	7.52	0.000
	11	11/12/2020	17.445	131.80	7.56	0.000
	12	11/13/2020	17.700	88.51	5.00	0.000
	13	11/14/2020	16.907	90.08	5.33	0.000
	14	11/15/2020	16.511	82.41	4.99	0.000
	15	11/16/2020	16.596	96.21	5.80	0.000
					**Mean:**	**0.000**
					**SD:**	**0.0000**
					**N:**	**15**

NOTES:

[1] Each study group was exposed for 14 days; however, due to staggered dosing for the two sexes, aerosol samples were collected over 15 days.

[2] Analytical concentration calculated based on HPLC analysis results (see Appendix B): mg/filter HPLC result/sample volume (L)

Table C-1 – Daily Test Atmosphere Concentration Data

Group (Treatment)	Exposure Day	Exposure Date	Volume (L)	Gravimetric Weight (mg)	Gravimetric Concentration (mg/L)	Analytical Concentration (mg/L)
3 (APN01; 0.019 mg/L)	AM SAMPLE COLLECTION					
	1	11/2/2020	19.937	6.72	0.34	0.019
	2	11/3/2020	18.804	5.36	0.29	0.016
	3	11/4/2020	19.569	6.91	0.35	0.019
	4	11/5/2020	39.223	12.26	0.31	0.016
	5	11/6/2020	37.241	12.14	0.33	0.016
	6	11/7/2020	39.053	13.14	0.34	0.017
	7	11/8/2020	48.456	15.87	0.33	0.016
	8	11/9/2020	42.055	14.17	0.34	0.017
	9	11/10/2020	38.090	14.21	0.37	0.018
	10	11/11/2020	44.349	15.23	0.34	0.017
	11	11/12/2020	38.657	13.63	0.35	0.017
	12	11/13/2020	40.809	10.55	0.26	0.013
	13	11/14/2020	39.846	13.29	0.33	0.017
	14	11/15/2020	42.537	14.46	0.34	0.017
	15	11/16/2020	43.103	15.50	0.36	0.017
					**Mean:**	**0.017**
					**SD:**	**0.0014**
					**N:**	**15**
	PM SAMPLE COLLECTION					
	1	11/2/2020	19.711	7.08	0.36	0.020
	2	11/3/2020	23.166	8.02	0.35	0.022
	3	11/4/2020	35.570	13.52	0.38	0.018
	4	11/5/2020	37.467	8.20	0.22	0.011
	5	11/6/2020	48.144	15.88	0.33	0.015
	6	11/7/2020	34.805	9.20	0.26	0.013
	7	11/8/2020	51.089	17.37	0.34	0.016
	8	11/9/2020	53.553	17.96	0.34	0.020
	9	11/10/2020	38.685	12.07	0.31	0.014
	10	11/11/2020	35.088	13.22	0.38	0.018
	11	11/12/2020	39.082	12.98	0.33	0.016
	12	11/13/2020	42.423	15.72	0.37	0.018
	13	11/14/2020	56.159	20.99	0.37	0.018
	14	11/15/2020	41.942	16.08	0.38	0.020
	15	11/16/2020	39.903	13.45	0.34	0.017
					**Mean:**	**0.017**
					**SD:**	**0.0028**
					**N:**	**15**

NOTES:

[1] Each study group was exposed for 14 days; however, due to staggered dosing for the two sexes, aerosol samples were collected over 15 days.

[2] Analytical concentration calculated based on HPLC analysis results (see Appendix B): mg/filter HPLC result/sample volume (L)

Table C-1 – Daily Test Atmosphere Concentration Data

Group (Treatment)	Exposure Day	Exposure Date	Volume (L)	Gravimetric Weight (mg)	Gravimetric Concentration (mg/L)	Analytical Concentration (mg/L)
4 (APN01; 0.038 mg/L)	**AM SAMPLE COLLECTION**					
	1	11/2/2020	18.918	11.17	0.59	0.027
	2	11/3/2020	22.373	12.32	0.55	0.026
	3	11/4/2020	18.833	15.97	0.85	0.038
	4	11/5/2020	25.176	18.73	0.74	0.033
	5	11/6/2020	25.800	18.69	0.72	0.033
	6	11/7/2020	24.667	17.94	0.73	0.032
	7	11/8/2020	28.547	22.64	0.79	0.034
	8	11/9/2020	27.216	21.58	0.79	0.034
	9	11/10/2020	27.584	21.24	0.77	0.035
	10	11/11/2020	29.283	21.04	0.72	0.031
	11	11/12/2020	22.486	17.83	0.79	0.035
	12	11/13/2020	28.462	23.72	0.83	0.035
	13	11/14/2020	22.203	18.25	0.82	0.036
	14	11/15/2020	28.150	23.11	0.82	0.035
	15	11/16/2020	29.424	25.40	0.86	0.039
					**Mean:**	**0.034**
					**SD:**	**0.0034**
					**N:**	**15**
	**PM SAMPLE COLLECTION**					
	1	11/2/2020	18.720	14.37	0.77	0.034
	2	11/3/2020	19.428	11.72	0.60	0.029
	3	11/4/2020	24.497	20.06	0.82	0.037
	4	11/5/2020	23.449	17.92	0.76	0.035
	5	11/6/2020	26.989	19.82	0.73	0.033
	6	11/7/2020	27.357	20.62	0.75	0.034
	7	11/8/2020	26.224	21.77	0.83	0.038
	8	11/9/2020	26.706	21.24	0.80	0.036
	9	11/10/2020	24.808	17.27	0.70	0.031
	10	11/11/2020	16.001	12.83	0.80	0.037
	11	11/12/2020	27.385	23.40	0.85	0.037
	12	11/13/2020	27.782	21.65	0.78	0.035
	13	11/14/2020	28.008	22.31	0.80	0.035
	14	11/15/2020	28.348	23.66	0.83	0.036
	15	11/16/2020	25.998	21.67	0.83	0.036
					**Mean:**	**0.035**
					**SD:**	**0.0024**
					**N:**	**15**

NOTES:

[1] Each study group was exposed for 14 days; however, due to staggered dosing for the two sexes, aerosol samples were collected over 15 days.

[2] Analytical concentration calculated based on HPLC analysis results (see Appendix B): mg/filter HPLC result/sample volume (L)

Table C-1 – Daily Test Atmosphere Concentration Data

Group (Treatment)	Exposure Day	Exposure Date	Volume (L)	Gravimetric Weight (mg)	Gravimetric Concentration (mg/L)	Analytical Concentration (mg/L)
**5 (APN01; 0.075 mg/L)**	**AM SAMPLE COLLECTION**					
	1	11/2/2020	21.382	154.08	7.21	0.068
	2	11/3/2020	21.268	266.99	12.55	0.073
	3	11/4/2020	21.325	283.73	13.31	0.072
	4	11/5/2020	21.098	248.66	11.79	0.078
	5	11/6/2020	19.626	197.10	10.04	0.088
	6	11/7/2020	19.512	259.65	13.31	0.080
	7	11/8/2020	20.532	206.68	10.07	0.086
	8	11/9/2020	19.852	270.01	13.60	0.085
	9	11/10/2020	21.268	288.77	13.58	0.072
	10	11/11/2020	23.137	336.34	14.54	0.083
	11	11/12/2020	22.429	315.64	14.07	0.078
	12	11/13/2020	22.231	318.95	14.35	0.080
	13	11/14/2020	22.571	302.04	13.38	0.073
	14	11/15/2020	22.769	315.73	13.87	0.072
	15	11/16/2020	22.231	292.10	13.14	0.084
					**Mean:**	**0.078**
					**SD:**	**0.0062**
					**N:**	**15**
	**PM SAMPLE COLLECTION**					
	1	11/2/2020	23.137	194.60	8.41	0.071
	2	11/3/2020	20.957	218.88	10.44	0.074
	3	11/4/2020	22.288	257.75	11.56	0.085
	4	11/5/2020	21.438	198.89	9.28	0.076
	5	11/6/2020	20.645	243.63	11.80	0.092
	6	11/7/2020	20.390	271.63	13.32	0.089
	7	11/8/2020	20.136	256.14	12.72	0.087
	8	11/9/2020	20.022	253.93	12.68	0.078
	9	11/10/2020	20.985	286.57	13.66	0.081
	10	11/11/2020	22.429	318.76	14.21	0.082
	11	11/12/2020	22.883	321.82	14.06	0.081
	12	11/13/2020	22.769	277.73	12.20	0.082
	13	11/14/2020	21.608	282.40	13.07	0.085
	14	11/15/2020	23.194	309.61	13.35	0.085
	15	11/16/2020	22.628	297.41	13.14	0.088
					**Mean:**	**0.082**
					**SD:**	**0.0058**
					**N:**	**15**

NOTES:
[1] Each study group was exposed for 14 days; however, due to staggered dosing for the two sexes, aerosol samples were collected over 15 days.
[2] Analytical concentration calculated based on HPLC analysis results (see Appendix B): mg/filter HPLC result/sample volume (L)

Table C-2 – Weekly Test Atmosphere Particle Size Distribution Data

Group (Treatment)	Exposure Date	Week/ Sample No.	Particle Size Distribution			
			MMAD (µm)	GSD	MMAD (µm) Average ± SD	GSD Range
**1 (Saline Control)**	11/7/2020	1	1.14	2.36	**1.06 ± 0.113**	**2.25 – 2.36**
	11/12/2020	2	0.98	2.25		
**2 (Vehicle Control)**	11/7/2020	1	1.30	2.20	**1.36 ± 0.078**	**2.20 – 2.56**
	11/12/2020	2	1.41	2.56		
**3 (APN01; 0.019 mg/L)**	11/7/2020	1	1.97	1.53	**1.57 ± 0.566**	**1.53 – 2.28**
	11/14/2020	2	1.17	2.28		
**4 (APN01; 0.038 mg/L)**	11/7/2020	1	2.06	1.80	**1.92 ± 0.205**	**1.80 – 2.41**
	11/14/2020	2	1.77	2.41		
**5 (APN01; 0.075mg/L)**	11/7/2020	1	2.20	1.95	**2.00 ± 0.290**	**1.87 – 1.95**
	11/14/2020	2	1.79	1.87		

NOTES: MMAD = mass median aerodynamic diameter; GSD = geometric standard deviation

Table C-3 – Daily Test Atmosphere Environmental Data

Group (Treatment)	Exposure Day	Exposure Date	Temperature Reading (°C)		Relative Humidity Reading (%)		Nebulizer Pressure Reading (PSI)	
			1st	2nd	1st	2nd	1st	2nd
	**AM SAMPLE COLLECTION**							
	1	11/2/2020	20.3	20.4	86.9	81.7	25.0	25.0
	2	11/3/2020	21.1	21.3	83.3	81.7	25.0	25.0
	3	11/4/2020	21.4	21.3	68.9	68.3	25.0	25.0
	4	11/5/2020	20.8	20.9	75.4	73.1	25.0	25.0
	5	11/6/2020	20.8	21.3	63.8	77.4	25.0	25.0
	6	11/7/2020	20.9	21.3	76.8	68.7	25.0	25.0
	7	11/8/2020	21.4	21.0	68.3	79.2	25.0	25.0
	8	11/9/2020	20.6	20.9	67.1	70.6	25.0	25.0
	9	11/10/2020	21.2	21.0	73.4	75.2	25.0	25.0
	10	11/11/2020	20.7	20.6	64.4	68.2	25.0	25.0
	11	11/12/2020	20.8	20.9	55.1	52.6	25.0	25.0
	12	11/13/2020	21.1	21.4	59.8	57.0	25.0	25.0
	13	11/14/2020	21.0	20.9	64.7	75.3	25.0	25.0
	14	11/15/2020	20.4	20.3	60.9	63.4	25.0	25.0
	15	11/16/2020	20.7	20.9	54.4	57.1	25.0	25.0
		**Mean:**	**21.0**		**69.1**		**25.0**	
		**SD:**	**0.30**		**8.79**		**0.00**	
**1**		**N:**	**30**		**30**		**30**	
**(Saline Control)**	**PM SAMPLE COLLECTION**							
	1	11/2/2020	20.7	20.3	80.4	79.2	25.0	25.0
	2	11/3/2020	21.0	21.3	80.3	75.7	25.0	25.0
	3	11/4/2020	21.8	21.2	78.3	69.4	25.0	25.0
	4	11/5/2020	21.8	21.6	68.4	71.3	25.0	25.0
	5	11/6/2020	21.7	21.4	80.3	68.7	25.0	25.0
	6	11/7/2020	21.7	21.9	69.4	72.4	25.0	25.0
	7	11/8/2020	21.3	21.0	81.4	78.6	25.0	25.0
	8	11/9/2020	20.8	21.1	71.9	80.3	25.0	25.0
	9	11/10/2020	21.0	21.3	78.9	82.7	25.0	25.0
	10	11/11/2020	20.8	20.9	75.7	84.0	25.0	25.0
	11	11/12/2020	20.8	20.9	68.3	73.7	25.0	25.0
	12	11/13/2020	21.1	20.9	54.2	59.3	25.0	25.0
	13	11/14/2020	21.3	21.4	79.7	80.7	25.0	25.0
	14	11/15/2020	20.9	20.7	57.8	54.6	25.0	25.0
	15	11/16/2020	20.3	20.5	62.8	60.3	25.0	25.0
		**Mean:**	**21.1**		**72.6**		**25.0**	
		**SD:**	**0.41**		**8.38**		**0.00**	
		**N:**	**30**		**30**		**30**	

NOTE: Each study group was exposed for 14 days; however, due to staggered dosing for the two genders, aerosol samples were collected over 15 days.

Table C-3 – Daily Test Atmosphere Environmental Data (cont.)

Group (Treatment)	Exposure Day	Exposure Date	Temperature Reading (°C)		Relative Humidity Reading (%)		Nebulizer Pressure Reading (PSI)	
			1st	2nd	1st	2nd	1st	2nd
	**AM SAMPLE COLLECTION**							
	1	11/2/2020	20.4	20.7	79.3	81.9	25.0	25.0
	2	11/3/2020	21.1	21.0	69.7	65.4	25.0	25.0
	3	11/4/2020	21.8	21.3	68.9	70.7	25.0	25.0
	4	11/5/2020	20.9	21.4	68.7	59.8	25.0	25.0
	5	11/6/2020	20.9	21.6	63.7	79.3	25.0	25.0
	6	11/7/2020	21.8	22.1	83.9	86.7	25.0	25.0
	7	11/8/2020	21.0	21.7	69.3	78.2	25.0	25.0
	8	11/9/2020	21.1	21.3	68.2	71.2	25.0	25.0
	9	11/10/2020	21.3	21.1	72.8	68.3	25.0	25.0
	10	11/11/2020	20.7	20.8	56.5	63.7	25.0	25.0
	11	11/12/2020	20.7	20.9	56.6	54.8	25.0	25.0
	12	11/13/2020	21.4	21.8	59.2	61.4	25.0	25.0
	13	11/14/2020	21.3	21.7	72.7	68.2	25.0	25.0
	14	11/15/2020	20.9	21.1	66.2	63.9	25.0	25.0
	15	11/16/2020	20.8	20.9	69.3	65.3	25.0	25.0
		**Mean:**	**21.2**		**68.8**		**25.0**	
		**SD:**	**0.37**		**7.60**		**0.00**	
**2 (Vehicle Control)**		**N:**	**30**		**30**		**30**	
	**PM SAMPLE COLLECTION**							
	1	11/2/2020	20.8	20.4	84.3	80.1	25.0	25.0
	2	11/3/2020	21.3	21.0	70.1	68.5	25.0	25.0
	3	11/4/2020	21.4	21.0	70.0	68.3	25.0	25.0
	4	11/5/2020	21.6	21.4	71.4	70.2	25.0	25.0
	5	11/6/2020	21.8	21.4	84.2	61.8	25.0	25.0
	6	11/7/2020	22.7	22.0	74.3	81.2	25.0	25.0
	7	11/8/2020	21.8	21.3	69.3	80.0	25.0	25.0
	8	11/9/2020	21.6	21.7	68.3	71.3	25.0	25.0
	9	11/10/2020	21.4	21.5	73.5	68.9	25.0	25.0
	10	11/11/2020	21.4	21.0	79.7	73.3	25.0	25.0
	11	11/12/2020	21.3	21.0	62.8	72.2	25.0	25.0
	12	11/13/2020	21.7	21.5	62.8	58.5	25.0	25.0
	13	11/14/2020	21.3	21.6	75.4	79.1	25.0	25.0
	14	11/15/2020	21.2	21.0	61.4	65.8	25.0	25.0
	15	11/16/2020	21.1	20.9	63.1	61.2	25.0	25.0
		**Mean:**	**21.4**		**71.1**		**25.0**	
		**SD:**	**0.40**		**6.08**		**0.00**	
		**N:**	**30**		**30**		**30**	

NOTE: Each study group was exposed for 14 days; however, due to staggered dosing for the two genders, aerosol samples were collected over 15 days.

Table C-3 – Daily Test Atmosphere Environmental Data (cont.)

Group (Treatment)	Exposure Day	Exposure Date	Temperature Reading (°C)		Relative Humidity Reading (%)		Nebulizer Pressure Reading (PSI)	
			1st	2nd	1st	2nd	1st	2nd
	**AM SAMPLE COLLECTION**							
	1	11/2/2020	20.3	20.6	87.3	86.4	25.0	25.0
	2	11/3/2020	22.0	21.9	31.7	30.3	25.0	25.0
	3	11/4/2020	20.7	20.9	45.4	43.1	25.0	25.0
	4	11/5/2020	21.1	21.6	42.7	39.1	25.0	25.0
	5	11/6/2020	21.3	21.6	41.2	39.7	25.0	25.0
	6	11/7/2020	22.4	22.0	37.0	36.1	25.0	25.0
	7	11/8/2020	21.3	21.5	42.5	44.1	25.0	25.0
	8	11/9/2020	20.7	20.8	47.2	49.1	25.0	25.0
	9	11/10/2020	20.4	20.6	50.2	48.9	25.0	25.0
	10	11/11/2020	20.4	20.6	46.7	48.3	25.0	25.0
	11	11/12/2020	20.6	20.8	48.8	46.2	25.0	25.0
	12	11/13/2020	20.6	20.8	46.2	44.9	25.0	25.0
	13	11/14/2020	20.9	21.1	46.4	39.3	25.0	25.0
	14	11/15/2020	20.7	20.9	52.3	50.6	25.0	25.0
	15	11/16/2020	20.8	20.9	52.9	47.2	25.0	25.0
		**Mean:**	**21.0**		**47.1**		**25.0**	
		**SD:**	**0.54**		**12.29**		**0.00**	
**3 (APN01; 0.019 mg/L)**		**N:**	**30**		**30**		**30**	
	**PM SAMPLE COLLECTION**							
	1	11/2/2020	20.5	20.6	52.6	34.8	25.0	25.0
	2	11/3/2020	22.4	22.3	39.7	37.6	25.0	25.0
	3	11/4/2020	21.8	21.2	57.5	61.2	25.0	25.0
	4	11/5/2020	21.6	21.5	26.1	30.1	25.0	25.0
	5	11/6/2020	21.9	22.0	42.8	41.3	25.0	25.0
	6	11/7/2020	22.7	23.0	45.6	48.9	25.0	25.0
	7	11/8/2020	21.6	21.3	41.6	45.8	25.0	25.0
	8	11/9/2020	20.9	20.7	50.6	53.7	25.0	25.0
	9	11/10/2020	20.8	20.9	42.7	51.1	25.0	25.0
	10	11/11/2020	19.7	19.9	43.0	45.0	25.0	25.0
	11	11/12/2020	20.9	21.2	40.9	41.6	25.0	25.0
	12	11/13/2020	20.5	20.3	51.1	49.2	25.0	25.0
	13	11/14/2020	21.7	21.9	44.6	36.2	25.0	25.0
	14	11/15/2020	20.6	20.9	54.8	51.1	25.0	25.0
	15	11/16/2020	19.8	20.1	45.1	47.3	25.0	25.0
		**Mean:**	**21.2**		**45.1**		**25.0**	
		**SD:**	**0.87**		**7.24**		**0.00**	
		**N:**	**30**		**30**		**30**	

NOTE: Each study group was exposed for 14 days; however, due to staggered dosing for the two genders, aerosol samples were collected over 15 days.

Table C-3 – Daily Test Atmosphere Environmental Data

Group (Treatment)	Exposure Day	Exposure Date	Temperature Reading (°C)		Relative Humidity Reading (%)		Nebulizer Pressure Reading (PSI)	
			1st	2nd	1st	2nd	1st	2nd
	**AM SAMPLE COLLECTION**							
	1	11/2/2020	20.3	20.3	87.8	75.3	25.0	25.0
	2	11/3/2020	21.5	21.3	39.6	37.8	25.0	25.0
	3	11/4/2020	20.4	20.6	50.5	49.4	25.0	25.0
	4	11/5/2020	21.8	21.5	50.6	52.1	25.0	25.0
	5	11/6/2020	21.6	21.8	42.9	44.1	25.0	25.0
	6	11/7/2020	22.1	21.5	55.3	48.0	25.0	25.0
	7	11/8/2020	20.3	20.5	58.1	56.3	25.0	25.0
	8	11/9/2020	20.9	20.7	56.7	52.3	25.0	25.0
	9	11/10/2020	21.3	21.2	61.0	54.8	25.0	25.0
	10	11/11/2020	19.3	19.8	59.5	40.5	25.0	25.0
	11	11/12/2020	20.2	20.4	63.3	59.7	25.0	25.0
	12	11/13/2020	20.4	20.6	52.8	54.1	25.0	25.0
	13	11/14/2020	20.9	20.8	56.7	51.4	25.0	25.0
	14	11/15/2020	20.0	20.2	53.8	55.6	25.0	25.0
	15	11/16/2020	21.3	21.4	56.3	52.7	25.0	25.0
		**Mean:**	**20.8**		**54.3**		**25.0**	
		**SD:**	**0.67**		**9.38**		**0.00**	
**4**		**N:**	**30**		**30**		**30**	
**(APN01; 0.038 mg/L)**	**PM SAMPLE COLLECTION**							
	1	11/2/2020	20.7	20.5	45.8	49.1	25.0	25.0
	2	11/3/2020	21.4	21.0	48.7	59.4	25.0	25.0
	3	11/4/2020	21.2	20.8	62.3	58.3	25.0	25.0
	4	11/5/2020	21.2	21.5	54.1	51.9	25.0	25.0
	5	11/6/2020	22.1	22.3	48.5	49.6	25.0	25.0
	6	11/7/2020	21.5	21.5	50.0	46.1	25.0	25.0
	7	11/8/2020	20.8	21.0	55.1	58.7	25.0	25.0
	8	11/9/2020	20.8	20.8	52.3	57.4	25.0	25.0
	9	11/10/2020	21.6	21.4	51.7	56.3	25.0	25.0
	10	11/11/2020	20.5	20.9	57.5	61.2	25.0	25.0
	11	11/12/2020	20.9	20.3	67.4	68.4	25.0	25.0
	12	11/13/2020	20.9	20.9	51.7	55.3	25.0	25.0
	13	11/14/2020	20.9	21.0	56.7	48.6	25.0	25.0
	14	11/15/2020	20.2	20.4	55.8	57.0	25.0	25.0
	15	11/16/2020	21.7	21.2	61.8	54.4	25.0	25.0
		**Mean:**	**21.1**		**55.1**		**25.0**	
		**SD:**	**0.48**		**5.22**		**0.00**	
		**N:**	**30**		**30**		**30**	

NOTE: Each study group was exposed for 14 days; however, due to staggered dosing for the two genders, aerosol samples were collected over 15 days.

Table C-3 – Daily Test Atmosphere Environmental Data

Group (Treatment)	Exposure Day	Exposure Date	Temperature Reading (°C)		Relative Humidity Reading (%)		Nebulizer Pressure Reading (PSI)	
			1st	2nd	1st	2nd	1st	2nd
	**AM SAMPLE COLLECTION**							
	1	11/2/2020	21.3	20.7	87.6	81.4	25.0	25.0
	2	11/3/2020	19.4	19.6	81.3	83.4	25.0	25.0
	3	11/4/2020	20.0	19.9	84.5	85.7	25.0	25.0
	4	11/5/2020	21.4	21.3	85.9	83.7	25.0	25.0
	5	11/6/2020	20.9	21.1	79.3	83.5	25.0	25.0
	6	11/7/2020	22.5	22.2	77.1	76.4	25.0	25.0
	7	11/8/2020	19.3	19.5	86.5	88.0	25.0	25.0
	8	11/9/2020	19.7	19.6	90.3	88.9	25.0	25.0
	9	11/10/2020	20.5	20.6	90.6	92.0	25.0	25.0
	10	11/11/2020	20.5	20.7	93.1	84.6	25.0	25.0
	11	11/12/2020	20.1	20.3	91.3	92.6	25.0	25.0
	12	11/13/2020	20.2	20.0	91.6	92.5	25.0	25.0
	13	11/14/2020	20.9	21.1	89.6	91.3	25.0	25.0
	14	11/15/2020	19.2	19.5	84.8	88.0	25.0	25.0
	15	11/16/2020	20.1	20.3	83.8	81.6	25.0	25.0
		**Mean:**	**20.4**		**86.4**		**25.0**	
		**SD:**	**0.84**		**4.45**		**0.00**	
**5**		**N:**	**30**		**30**		**30**	
**(APN01; 0.075 mg/L)**	**PM SAMPLE COLLECTION**							
	1	11/2/2020	20.3	20.3	84.3	84.1	25.0	25.0
	2	11/3/2020	20.9	21.1	82.7	84.1	25.0	25.0
	3	11/4/2020	20.8	21.1	37.3	46.4	25.0	25.0
	4	11/5/2020	21.8	21.9	81.0	83.0	25.0	25.0
	5	11/6/2020	20.7	20.9	75.6	80.3	25.0	25.0
	6	11/7/2020	23.6	21.0	78.8	77.9	25.0	25.0
	7	11/8/2020	19.7	19.9	87.6	82.1	25.0	25.0
	8	11/9/2020	19.7	19.9	87.6	84.9	25.0	25.0
	9	11/10/2020	20.7	20.6	89.3	85.7	25.0	25.0
	10	11/11/2020	20.4	20.7	89.4	84.9	25.0	25.0
	11	11/12/2020	20.3	20.8	89.1	90.4	25.0	25.0
	12	11/13/2020	19.4	19.6	90.6	92.2	25.0	25.0
	13	11/14/2020	21.4	21.8	92.6	83.3	25.0	25.0
	14	11/15/2020	21.0	21.2	91.4	92.6	25.0	25.0
	15	11/16/2020	19.4	19.6	88.0	90.3	25.0	25.0
		**Mean:**	**20.7**		**82.9**		**25.0**	
		**SD:**	**0.84**		**12.11**		**0.00**	
		**N:**	**30**		**30**		**30**	

NOTE: Each study group was exposed for 14 days; however, due to staggered dosing for the two genders, aerosol samples were collected over 15 days.

Table C-4 – Individual Animal Clinical Observations and Fates

MALES

Dose Group	Animal Number	Sign Type	Sign	First Day	Last Day	Duration [Days]
G 1 / M Saline	1EZF	normal	normal	1	14	14
		fate	terminal sacrifice	15	15	1
	1FUH	normal	normal	1	14	14
		fate	terminal sacrifice	15	15	1
	1UYF	normal	normal	1	14	14
		fate	terminal sacrifice	15	15	1
G 2 / M Vehicle	2AGF	normal	normal	1	14	14
		fate	terminal sacrifice	15	15	1
	2BSF	normal	normal	1	2	2
		general	canine cherry eye	3	14	12
		fate	terminal sacrifice	15	15	1
	2FRH	normal	normal	1	14	14
		fate	terminal sacrifice	15	15	1
G 3 / M Low 0.019 mg/L	3DWH	normal	normal	1	14	14
		fate	terminal sacrifice	15	15	1
	3THF	normal	normal	1	14	14
		fate	terminal sacrifice	15	15	1
	3TYF	normal	normal	1	14	14
		fate	terminal sacrifice	15	15	1
G 4 / M Mid 0.038 mg/L	4EAH	normal	normal	1	14	14
		fate	terminal sacrifice	15	15	1
	4JLH	normal	normal	1	14	14
		fate	terminal sacrifice	15	15	1
	4YJF	normal	normal	1	10	10
		general	sore	11	14	4
		fate	terminal sacrifice	15	15	1
G 5 / M High 0.075 mg/L	5GPH	normal	normal	1	4	4
		general	sore	5	14	10
		fate	terminal sacrifice	15	15	1
	5ZOF	normal	normal	1	14	14
		fate	terminal sacrifice	15	15	1
	5ZTF	normal	normal	1	14	14
		fate	terminal sacrifice	15	15	1

NOTES:

[1] The canine cherry eye seen in Group 2 male 2BSF on Days 3-14 was noted to be in the right eye.

[2] The sores seen in Group 4 male 4YJF and Group 5 male 5GPH were noted to be on the muzzle.

Table C-4 – Individual Animal Clinical Observations and Fates

FEMALES

Dose Group	Animal Number	Sign Type	Sign	First Day	Last Day	Duration [Days]
G 1 / F Saline	1CBE	normal	normal	1	14	14
		fate	terminal sacrifice	15	15	1
	1JKG	normal	normal	1	14	14
		fate	terminal sacrifice	15	15	1
	1JSG	normal	normal	1	14	14
		fate	terminal sacrifice	15	15	1
G 2 / F Vehicle	2AJE	normal	normal	1	14	14
		fate	terminal sacrifice	15	15	1
	2ZQE	normal	normal	1	1	1
		general	alopecia	2	14	13
		general	alopecia	2	14	13
		fate	terminal sacrifice	15	15	1
	2FLE	normal	normal	1	14	14
		fate	terminal sacrifice	15	15	1
G 3 / F Low 0.019 mg/L	3CVG	normal	normal	1	14	14
		fate	terminal sacrifice	15	15	1
	3FKE	normal	normal	1	14	14
		fate	terminal sacrifice	15	15	1
	3ZRE	normal	normal	1	14	14
		fate	terminal sacrifice	15	15	1
G 4 / F Mid 0.038 mg/L	4ACE	normal	normal	1	14	14
		fate	terminal sacrifice	15	15	1
	4CAE	normal	normal	1	14	14
		fate	terminal sacrifice	15	15	1
	4EVE	normal	normal	1	14	14
		fate	terminal sacrifice	15	15	1
G 5 / F High 0.075 mg/L	5AIE	normal	normal	1	14	14
		fate	terminal sacrifice	15	15	1
	5CGE	normal	normal	1	14	14
		fate	terminal sacrifice	15	15	1
	5CSG	normal	normal	1	14	14
		fate	terminal sacrifice	15	15	1

NOTE: The alopecia seen in Group 2 female 2ZQE on Days 2-14 was located on the forelimbs and hindlimbs (one entry for each location). The severity at both locations was noted to be slight.

Table C-5 – Individual Animal Detailed Physical Examination Observations

MALES

Dose Group	Animal Number	Sign Type	Sign	First Day	Last Day	Duration [Days]
G 1 / M Saline	1EZF	normal	normal	1	15	15
	1FUH	normal	normal	1	15	15
	1UYF	normal	normal	1	15	15
G 2 / M Vehicle	2AGF	normal	normal	1	15	15
	2BSF	general	canine cherry eye	1	15	15
	2FRH	normal	normal	1	15	15
G 3 / M Low 0.019 mg/L	3DWH	normal	normal	1	15	15
	3THF	normal	normal	1	15	15
	3TYF	normal	normal	1	15	15
G 4 / M Mid 0.038 mg/L	4EAH	normal	normal	1	15	15
	4JLH	normal	normal	1	15	15
	4YJF	normal	normal	1	9	9
		general	sore	10	11	2
		normal	normal	12	12	1
		general	sore	13	15	3
G 5 / M High 0.075 mg/L	5GPH	normal	normal	1	4	4
		general	sore	5	14	10
		general	scab	15	15	1
	5ZOF	general	scab	1	1	1
		normal	normal	2	15	14
	5ZTF	normal	normal	1	15	15

NOTES:

[1] The canine cherry eye seen in Group 2 male 2BSF on Days 1-15 was noted to be red and located in the right eye.

[2] The sores seen in Group 4 male 4YJF on Days 10-11 and 13-15 and in Group 5 male 5GPH on Days 5-14 were noted to be on the muzzle; the scab seen in the same Group 5 dog on Day 15 was also noted to be on the muzzle.

[3] The scab seen in Group 5 male 5ZOF on Day 1 was on the left dorsal region of the neck. It was noted to be 7 mm x 3 mm in size.

Table C-5 – Individual Animal Detailed Physical Examination Observations

FEMALES

Dose Group	Animal Number	Sign Type	Sign	First Day	Last Day	Duration [Days]
G 1 / F Saline	1CBE	general	sore	1	1	1
		normal	normal	2	15	14
	1JKG	normal	normal	1	15	15
	1JSG	normal	normal	1	15	15
G 2 / F Vehicle	2AJE	normal	normal	1	15	15
	2ZQE	general	alopecia	1	15	15
		general	alopecia	1	15	15
	2FLE	normal	normal	1	15	15
G 3 / F Low 0.019 mg/L	3CVG	normal	normal	1	15	15
	3FKE	normal	normal	1	15	15
	3ZRE	normal	normal	1	15	15
G 4 / F Mid 0.038 mg/L	4ACE	normal	normal	1	15	15
	4CAE	normal	normal	1	15	15
	4EVE	normal	normal	1	15	15
G 5 / F High 0.075 mg/L	5AIE	normal	normal	1	15	15
	5CGE	normal	normal	1	15	15
	5CSG	normal	normal	1	15	15

NOTES:

[1] The sore seen in Group 1 female 1CBE on Day 1 was on the left inguinal region of the dog.

[2] The alopecia seen in Group 2 female 2ZQE on Days 1-15 was located on the forelimbs and hindlimbs (one entry for each location). The severity at both locations was noted to be slight.

Table C-6 – Individual Animal Heart Rate and Blood Pressure Data

MALES (PRE-TEST)

Dose Group	Animal Number	Heart Rate [Beats/Minute] day -4	Diastolic Blood Pressure [mmHg] day -4	Systolic Blood Pressure [mmHg] day -4	Mean Arterial Pressure [mmHg] day -4
G 1 / M Saline	1EZF	130	92	150	107
	1FUH	116	81	143	95
	1UYF	140	86	136	100
	Mean	129	86	143	101
	S.D.	12.1	5.5	7.0	6.0
	N	3	3	3	3
G 2 / M Vehicle	2AGF	156	112	172	129
	2BSF	121	79	175	110
	2FRH	137	105	175	123
	Mean	138	99	174	121
	S.D.	17.5	17.4	1.7	9.7
	N	3	3	3	3
G 3 / M Low 0.019 mg/L	3DWH	126	84	141	101
	3THF	151	112	181	127
	3TYF	108	47	133	69
	Mean	128	81	152	99
	S.D.	21.6	32.6	25.7	29.1
	N	3	3	3	3
G 4 / M Mid 0.038 mg/L	4EAH	117	81	165	98
	4JLH	121	81	171	108
	4YJF	81	56	153	83
	Mean	106	73	163	96
	S.D.	22.0	14.4	9.2	12.6
	N	3	3	3	3
G 5 / M High 0.075 mg/L	5GPH	102	81	174	107
	5ZOF	133	56	132	72
	5ZTF	148	105	174	112
	Mean	128	81	160	97
	S.D.	23.5	24.5	24.2	21.8
	N	3	3	3	3

Table C-6 – Individual Animal Heart Rate and Blood Pressure Data

MALES (DAY 1)

Dose Group	Animal Number	Heart Rate [Beats/Minute] day 1	Diastolic Blood Pressure [mmHg] day 1	Systolic Blood Pressure [mmHg] day 1	Mean Arterial Pressure [mmHg] day 1
G 1 / M Saline	1EZF	146	71	178	97
	1FUH	131	112	160	119
	1UYF	92	59	95	68
	Mean	123	81	144	95
	S.D.	27.9	27.8	43.7	25.6
	N	3	3	3	3
G 2 / M Vehicle	2AGF	167	89	141	103
	2BSF	133	113	171	124
	2FRH	137	114	170	127
	Mean	146	105	161	118
	S.D.	18.6	14.2	17.0	13.1
	N	3	3	3	3
G 3 / M Low 0.019 mg/L	3DWH	138	77	162	100
	3THF	142	94	145	107
	3TYF	118	65	88	68
	Mean	133	79	132	92
	S.D.	12.9	14.6	38.8	20.8
	N	3	3	3	3
G 4 / M Mid 0.038 mg/L	4EAH	124	84	161	100
	4JLH	127	84	165	100
	4YJF	102	54	87	60
	Mean	118	74	138	87
	S.D.	13.7	17.3	43.9	23.1
	N	3	3	3	3
G 5 / M High 0.075 mg/L	5GPH	135	123	213	145
	5ZOF	94	74	96	79
	5ZTF	136	114	159	119
	Mean	122	104	156	114
	S.D.	24.0	26.1	58.6	33.2
	N	3	3	3	3

Table C-6 – Individual Animal Heart Rate and Blood Pressure Data

MALES (DAY 2)

Dose Group	Animal Number	Heart Rate [Beats/Minute] day 2	Diastolic Blood Pressure [mmHg] day 2	Systolic Blood Pressure [mmHg] day 2	Mean Arterial Pressure [mmHg] day 2
G 1 / M Saline	1EZF	144	104	154	111
	1FUH	69	107	139	111
	1UYF	94	50	162	68
	Mean	102	87	152	97
	S.D.	38.2	32.1	11.7	24.8
	N	3	3	3	3
G 2 / M Vehicle	2AGF	142	54	150	79
	2BSF	144	48	64	51
	2FRH	117	43	71	49
	Mean	134	48	95	60
	S.D.	15.0	5.5	47.8	16.8
	N	3	3	3	3
G 3 / M Low 0.019 mg/L	3DWH	138	100	172	110
	3THF	151	86	149	103
	3TYF	99	58	76	63
	Mean	129	81	132	92
	S.D.	27.1	21.4	50.1	25.4
	N	3	3	3	3
G 4 / M Mid 0.038 mg/L	4EAH	118	78	129	92
	4JLH	104	78	148	94
	4YJF	126	40	148	68
	Mean	116	65	142	85
	S.D.	11.1	21.9	11.0	14.5
	N	3	3	3	3
G 5 / M High 0.075 mg/L	5GPH	101	79	132	90
	5ZOF	111	130	163	139
	5ZTF	151	97	173	106
	Mean	121	102	156	112
	S.D.	26.5	25.9	21.4	25.0
	N	3	3	3	3

Table C-6 – Individual Animal Heart Rate and Blood Pressure Data

MALES (DAY 3)

Dose Group	Animal Number	Heart Rate [Beats/Minute] day 3	Diastolic Blood Pressure [mmHg] day 3	Systolic Blood Pressure [mmHg] day 3	Mean Arterial Pressure [mmHg] day 3
G 1 / M Saline	1EZF	145	79	143	95
	1FUH	140	79	140	93
	1UYF	101	82	95	85
	Mean	129	80	126	91
	S.D.	24.1	1.7	26.9	5.3
	N	3	3	3	3
G 2 / M Vehicle	2AGF	144	70	150	90
	2BSF	146	69	154	92
	2FRH	123	66	91	72
	Mean	138	68	132	85
	S.D.	12.7	2.1	35.3	11.0
	N	3	3	3	3
G 3 / M Low 0.019 mg/L	3DWH	120	47	68	53
	3THF	163	81	113	87
	3TYF	103	84	109	91
	Mean	129	71	97	77
	S.D.	30.9	20.6	24.9	20.9
	N	3	3	3	3
G 4 / M Mid 0.038 mg/L	4EAH	137	68	166	91
	4JLH	99	84	148	94
	4YJF	116	58	150	78
	Mean	117	70	155	88
	S.D.	19.0	13.1	9.9	8.5
	N	3	3	3	3
G 5 / M High 0.075 mg/L	5GPH	153	140	171	147
	5ZOF	122	51	68	54
	5ZTF	164	130	184	139
	Mean	146	107	141	113
	S.D.	21.8	48.8	63.6	51.5
	N	3	3	3	3

Table C-6 – Individual Animal Heart Rate and Blood Pressure Data

MALES (DAY 4)

Dose Group	Animal Number	Heart Rate [Beats/Minute] day 4	Diastolic Blood Pressure [mmHg] day 4	Systolic Blood Pressure [mmHg] day 4	Mean Arterial Pressure [mmHg] day 4
G 1 / M Saline	1EZF	132	85	146	99
	1FUH	129	63	138	75
	1UYF	119	60	98	67
	Mean	127	69	127	80
	S.D.	6.8	13.7	25.7	16.7
	N	3	3	3	3
G 2 / M Vehicle	2AGF	131	45	144	75
	2BSF	134	58	85	64
	2FRH	102	56	78	60
	Mean	122	53	102	66
	S.D.	17.7	7.0	36.3	7.8
	N	3	3	3	3
G 3 / M Low 0.019 mg/L	3DWH	150	15	159	119
	3THF	188	111	137	117
	3TYF	128	71	152	90
	Mean	155	66	149	109
	S.D.	30.4	48.2	11.2	16.2
	N	3	3	3	3
G 4 / M Mid 0.038 mg/L	4EAH	116	113	164	125
	4JLH	113	75	101	80
	4YJF	76	75	106	82
	Mean	102	88	124	96
	S.D.	22.3	21.9	35.0	25.4
	N	3	3	3	3
G 5 / M High 0.075 mg/L	5GPH	103	89	152	95
	5ZOF	148	53	73	57
	5ZTF	150	129	199	142
	Mean	134	90	141	98
	S.D.	26.6	38.0	63.7	42.6
	N	3	3	3	3

Table C-6 – Individual Animal Heart Rate and Blood Pressure Data

MALES (DAY 5)

Dose Group	Animal Number	Heart Rate [Beats/Minute] day 5	Diastolic Blood Pressure [mmHg] day 5	Systolic Blood Pressure [mmHg] day 5	Mean Arterial Pressure [mmHg] day 5
G 1 / M Saline	1EZF	151	85	139	98
	1FUH	148	51	94	58
	1UYF	114	53	104	60
	Mean	138	63	112	72
	S.D.	20.6	19.1	23.6	22.5
	N	3	3	3	3
G 2 / M Vehicle	2AGF	146	81	141	93
	2BSF	152	80	147	97
	2FRH	184	129	182	138
	Mean	161	97	157	109
	S.D.	20.4	28.0	22.1	24.9
	N	3	3	3	3
G 3 / M Low 0.019 mg/L	3DWH	124	184	214	194
	3THF	165	129	174	140
	3TYF	144	77	123	86
	Mean	144	130	170	140
	S.D.	20.5	53.5	45.6	54.0
	N	3	3	3	3
G 4 / M Mid 0.038 mg/L	4EAH	130	107	156	112
	4JLH	153	64	93	70
	4YJF	130	84	109	89
	Mean	138	85	119	90
	S.D.	13.3	21.5	32.7	21.0
	N	3	3	3	3
G 5 / M High 0.075 mg/L	5GPH	86	74	150	93
	5ZOF	161	93	149	107
	5ZTF	153	113	152	121
	Mean	133	93	150	107
	S.D.	41.2	19.5	1.5	14.0
	N	3	3	3	3

Table C-6 – Individual Animal Heart Rate and Blood Pressure Data

MALES (DAY 6)

Dose Group	Animal Number	Heart Rate [Beats/Minute] day 6	Diastolic Blood Pressure [mmHg] day 6	Systolic Blood Pressure [mmHg] day 6	Mean Arterial Pressure [mmHg] day 6
G 1 / M Saline	1EZF	121	95	142	101
	1FUH	151	72	123	82
	1UYF	154	46	122	58
	Mean	142	71	129	80
	S.D.	18.2	24.5	11.3	21.5
	N	3	3	3	3
G 2 / M Vehicle	2AGF	157	129	152	136
	2BSF	81	131	173	142
	2FRH	164	99	160	114
	Mean	134	120	162	131
	S.D.	46.0	17.9	10.6	14.7
	N	3	3	3	3
G 3 / M Low 0.019 mg/L	3DWH	140	105	145	107
	3THF	158	94	155	114
	3TYF	98	66	141	81
	Mean	132	88	147	101
	S.D.	30.8	20.1	7.2	17.4
	N	3	3	3	3
G 4 / M Mid 0.038 mg/L	4EAH	143	41	183	88
	4JLH	144	99	156	111
	4YJF	128	57	148	76
	Mean	138	66	162	92
	S.D.	9.0	30.0	18.3	17.8
	N	3	3	3	3
G 5 / M High 0.075 mg/L	5GPH	134	93	148	105
	5ZOF	157	96	133	103
	5ZTF	137	95	149	111
	Mean	143	95	143	106
	S.D.	12.5	1.5	9.0	4.2
	N	3	3	3	3

Table C-6 – Individual Animal Heart Rate and Blood Pressure Data

MALES (DAY 7)

Dose Group	Animal Number	Heart Rate [Beats/Minute] day 7	Diastolic Blood Pressure [mmHg] day 7	Systolic Blood Pressure [mmHg] day 7	Mean Arterial Pressure [mmHg] day 7
G 1 / M Saline	1EZF	113	63	136	83
	1FUH	123	57	116	70
	1UYF	144	81	153	94
	Mean	127	67	135	82
	S.D.	15.8	12.5	18.5	12.0
	N	3	3	3	3
G 2 / M Vehicle	2AGF	152	77	135	79
	2BSF	162	95	162	108
	2FRH	156	35	122	61
	Mean	157	69	140	83
	S.D.	5.0	30.8	20.4	23.7
	N	3	3	3	3
G 3 / M Low 0.019 mg/L	3DWH	159	74	184	108
	3THF	142	83	178	116
	3TYF	144	89	175	119
	Mean	148	82	179	114
	S.D.	9.3	7.5	4.6	5.7
	N	3	3	3	3
G 4 / M Mid 0.038 mg/L	4EAH	145	77	159	100
	4JLH	108	26	45	29
	4YJF	91	84	137	94
	Mean	115	62	114	74
	S.D.	27.6	31.7	60.5	39.4
	N	3	3	3	3
G 5 / M High 0.075 mg/L	5GPH	108	94	146	106
	5ZOF	131	94	152	107
	5ZTF	131	94	152	107
	Mean	123	94	150	107
	S.D.	13.3	0.0	3.5	0.6
	N	3	3	3	3

Table C-6 – Individual Animal Heart Rate and Blood Pressure Data

MALES (DAY 8)

Dose Group	Animal Number	Heart Rate [Beats/Minute] day 8	Diastolic Blood Pressure [mmHg] day 8	Systolic Blood Pressure [mmHg] day 8	Mean Arterial Pressure [mmHg] day 8
G 1 / M Saline	1EZF	133	94	142	106
	1FUH	123	71	131	85
	1UYF	117	98	170	102
	Mean	124	88	148	98
	S.D.	8.1	14.6	20.1	11.2
	N	3	3	3	3
G 2 / M Vehicle	2AGF	142	62	122	70
	2BSF	153	38	62	43
	2FRH	154	115	169	120
	Mean	150	72	118	78
	S.D.	6.7	39.4	53.6	39.1
	N	3	3	3	3
G 3 / M Low 0.019 mg/L	3DWH	156	105	159	117
	3THF	150	106	160	120
	3TYF	121	92	141	102
	Mean	142	101	153	113
	S.D.	18.7	7.8	10.7	9.6
	N	3	3	3	3
G 4 / M Mid 0.038 mg/L	4EAH	145	110	160	121
	4JLH	128	76	138	82
	4YJF	124	57	153	77
	Mean	132	81	150	93
	S.D.	11.2	26.9	11.2	24.1
	N	3	3	3	3
G 5 / M High 0.075 mg/L	5GPH	105	89	154	103
	5ZOF	172	56	73	60
	5ZTF	148	107	160	114
	Mean	142	84	129	92
	S.D.	33.9	25.9	48.6	28.5
	N	3	3	3	3

Table C-6 – Individual Animal Heart Rate and Blood Pressure Data

MALES (DAY 9)

Dose Group	Animal Number	Heart Rate [Beats/Minute] day 9	Diastolic Blood Pressure [mmHg] day 9	Systolic Blood Pressure [mmHg] day 9	Mean Arterial Pressure [mmHg] day 9
G 1 / M Saline	1EZF	127	130	130	81
	1FUH	145	78	125	91
	1UYF	145	77	127	88
	Mean	139	95	127	87
	S.D.	10.4	30.3	2.5	5.1
	N	3	3	3	3
G 2 / M Vehicle	2AGF	139	71	129	82
	2BSF	160	77	121	87
	2FRH	166	113	165	125
	Mean	155	87	138	98
	S.D.	14.2	22.7	23.4	23.5
	N	3	3	3	3
G 3 / M Low 0.019 mg/L	3DWH	138	74	169	83
	3THF	160	112	159	121
	3TYF	121	97	136	106
	Mean	140	94	155	103
	S.D.	19.6	19.1	16.9	19.1
	N	3	3	3	3
G 4 / M Mid 0.038 mg/L	4EAH	166	93	137	102
	4JLH	127	92	155	107
	4YJF	125	96	150	104
	Mean	139	94	147	104
	S.D.	23.1	2.1	9.3	2.5
	N	3	3	3	3
G 5 / M High 0.075 mg/L	5GPH	113	71	156	95
	5ZOF	129	45	108	62
	5ZTF	126	82	146	100
	Mean	123	66	137	86
	S.D.	8.5	19.0	25.3	20.6
	N	3	3	3	3

Table C-6 – Individual Animal Heart Rate and Blood Pressure Data

MALES (DAY 10)

Dose Group	Animal Number	Heart Rate [Beats/Minute] day 10	Diastolic Blood Pressure [mmHg] day 10	Systolic Blood Pressure [mmHg] day 10	Mean Arterial Pressure [mmHg] day 10
G 1 / M Saline	1EZF	138	78	142	92
	1FUH	124	144	177	154
	1UYF	111	77	126	85
	Mean	124	100	148	110
	S.D.	13.5	38.4	26.1	38.0
	N	3	3	3	3
G 2 / M Vehicle	2AGF	134	92	138	103
	2BSF	161	111	128	116
	2FRH	149	92	129	96
	Mean	148	98	132	105
	S.D.	13.5	11.0	5.5	10.1
	N	3	3	3	3
G 3 / M Low 0.019 mg/L	3DWH	186	100	159	116
	3THF	148	128	182	138
	3TYF	164	100	152	113
	Mean	166	109	164	122
	S.D.	19.1	16.2	15.7	13.7
	N	3	3	3	3
G 4 / M Mid 0.038 mg/L	4EAH	136	126	180	137
	4JLH	123	51	83	58
	4YJF	124	116	170	127
	Mean	128	98	144	107
	S.D.	7.2	40.7	53.4	43.0
	N	3	3	3	3
G 5 / M High 0.075 mg/L	5GPH	124	85	180	103
	5ZOF	158	51	70	55
	5ZTF	130	109	159	118
	Mean	137	82	136	92
	S.D.	18.1	29.1	58.4	32.9
	N	3	3	3	3

Table C-6 – Individual Animal Heart Rate and Blood Pressure Data

MALES (DAY 11)

Dose Group	Animal Number	Heart Rate [Beats/Minute] day 11	Diastolic Blood Pressure [mmHg] day 11	Systolic Blood Pressure [mmHg] day 11	Mean Arterial Pressure [mmHg] day 11
G 1 / M Saline	1EZF	133	88	125	96
	1FUH	79	73	105	81
	1UYF	128	39	119	61
	Mean	113	67	116	79
	S.D.	29.8	25.1	10.3	17.6
	N	3	3	3	3
G 2 / M Vehicle	2AGF	149	69	111	77
	2BSF	153	91	155	98
	2FRH	182	81	129	91
	Mean	161	80	132	89
	S.D.	18.0	11.0	22.1	10.7
	N	3	3	3	3
G 3 / M Low 0.019 mg/L	3DWH	147	150	199	156
	3THF	141	100	141	107
	3TYF	88	73	132	86
	Mean	125	108	157	116
	S.D.	32.5	39.1	36.4	35.9
	N	3	3	3	3
G 4 / M Mid 0.038 mg/L	4EAH	128	120	173	128
	4JLH	135	114	153	123
	4YJF	112	99	180	122
	Mean	125	111	169	124
	S.D.	11.8	10.8	14.0	3.2
	N	3	3	3	3
G 5 / M High 0.075 mg/L	5GPH	107	89	163	107
	5ZOF	133	95	156	99
	5ZTF	134	98	167	117
	Mean	125	94	162	108
	S.D.	15.3	4.6	5.6	9.0
	N	3	3	3	3

Table C-6 – Individual Animal Heart Rate and Blood Pressure Data

MALES (DAY 12)

Dose Group	Animal Number	Heart Rate [Beats/Minute] day 12	Diastolic Blood Pressure [mmHg] day 12	Systolic Blood Pressure [mmHg] day 12	Mean Arterial Pressure [mmHg] day 12
G 1 / M Saline	1EZF	134	79	128	89
	1FUH	149	84	142	97
	1UYF	116	77	119	84
	Mean	133	80	130	90
	S.D.	16.5	3.6	11.6	6.6
	N	3	3	3	3
G 2 / M Vehicle	2AGF	161	77	139	87
	2BSF	156	78	143	94
	2FRH	153	74	142	92
	Mean	157	76	141	91
	S.D.	4.0	2.1	2.1	3.6
	N	3	3	3	3
G 3 / M Low 0.019 mg/L	3DWH	176	92	158	106
	3THF	143	91	156	104
	3TYF	130	94	160	112
	Mean	150	92	158	107
	S.D.	23.7	1.5	2.0	4.2
	N	3	3	3	3
G 4 / M Mid 0.038 mg/L	4EAH	163	76	140	87
	4JLH	130	128	165	145
	4YJF	138	74	146	81
	Mean	144	93	150	104
	S.D.	17.2	30.6	13.1	35.3
	N	3	3	3	3
G 5 / M High 0.075 mg/L	5GPH	118	67	156	91
	5ZOF	143	94	163	113
	5ZTF	143	73	167	97
	Mean	135	78	162	100
	S.D.	14.4	14.2	5.6	11.4
	N	3	3	3	3

Table C-6 – Individual Animal Heart Rate and Blood Pressure Data

MALES (DAY 13)

Dose Group	Animal Number	Heart Rate [Beats/Minute] day 13	Diastolic Blood Pressure [mmHg] day 13	Systolic Blood Pressure [mmHg] day 13	Mean Arterial Pressure [mmHg] day 13
G 1 / M Saline	1EZF	112	71	119	81
	1FUH	123	70	118	79
	1UYF	115	75	112	82
	Mean	117	72	116	81
	S.D.	5.7	2.6	3.8	1.5
	N	3	3	3	3
G 2 / M Vehicle	2AGF	151	60	116	75
	2BSF	153	95	136	103
	2FRH	163	97	137	111
	Mean	156	84	130	96
	S.D.	6.4	20.8	11.8	18.9
	N	3	3	3	3
G 3 / M Low 0.019 mg/L	3DWH	164	89	174	109
	3THF	150	89	169	110
	3TYF	136	90	151	95
	Mean	150	89	165	105
	S.D.	14.0	0.6	12.1	8.4
	N	3	3	3	3
G 4 / M Mid 0.038 mg/L	4EAH	167	112	164	117
	4JLH	138	30	44	33
	4YJF	93	109	167	115
	Mean	133	84	125	88
	S.D.	37.3	46.5	70.2	47.9
	N	3	3	3	3
G 5 / M High 0.075 mg/L	5GPH	111	84	137	96
	5ZOF	136	52	142	62
	5ZTF	144	87	150	99
	Mean	130	74	143	86
	S.D.	17.2	19.4	6.6	20.6
	N	3	3	3	3

Table C-6 – Individual Animal Heart Rate and Blood Pressure Data

MALES (DAY 14)

Dose Group	Animal Number	Heart Rate [Beats/Minute] day 14	Diastolic Blood Pressure [mmHg] day 14	Systolic Blood Pressure [mmHg] day 14	Mean Arterial Pressure [mmHg] day 14
G 1 / M Saline	1EZF	107	51	149	80
	1FUH	144	99	121	104
	1UYF	81	56	71	60
	Mean	111	69	114	81
	S.D.	31.7	26.4	39.5	22.0
	N	3	3	3	3
G 2 / M Vehicle	2AGF	155	73	163	98
	2BSF	147	71	159	91
	2FRH	130	45	63	49
	Mean	144	63	128	79
	S.D.	12.8	15.6	56.6	26.5
	N	3	3	3	3
G 3 / M Low 0.019 mg/L	3DWH	139	170	200	180
	3THF	132	114	163	119
	3TYF	149	115	162	124
	Mean	140	133	175	141
	S.D.	8.5	32.0	21.7	33.9
	N	3	3	3	3
G 4 / M Mid 0.038 mg/L	4EAH	157	96	142	109
	4JLH	131	108	159	116
	4YJF	158	98	144	113
	Mean	149	101	148	113
	S.D.	15.3	6.4	9.3	3.5
	N	3	3	3	3
G 5 / M High 0.075 mg/L	5GPH	108	93	162	111
	5ZOF	139	84	149	103
	5ZTF	118	96	160	115
	Mean	122	91	157	110
	S.D.	15.8	6.2	7.0	6.1
	N	3	3	3	3

Table C-6 – Individual Animal Heart Rate and Blood Pressure Data

FEMALES (PRE-TEST)

Dose Group	Animal Number	Heart Rate [Beats/Minute] day -5	Diastolic Blood Pressure [mmHg] day -5	Systolic Blood Pressure [mmHg] day -5	Mean Arterial Pressure [mmHg] day -5
G 1 / F Saline	1CBE	149	97	199	116
	1JKG	139	101	157	113
	1JSG	154	94	149	108
	Mean	147	97	168	112
	S.D.	7.6	3.5	26.9	4.0
	N	3	3	3	3
G 2 / F Vehicle	2AJE	138	60	125	76
	2ZQE	100	59	95	69
	2FLE	147	87	155	90
	Mean	128	69	125	78
	S.D.	24.9	15.9	30.0	10.7
	N	3	3	3	3
G 3 / F Low 0.019 mg/L	3CVG	133	101	150	112
	3FKE	120	78	149	92
	3ZRE	75	114	171	129
	Mean	109	98	157	111
	S.D.	30.4	18.2	12.4	18.5
	N	3	3	3	3
G 4 / F Mid 0.038 mg/L	4ACE	165	143	188	154
	4CAE	158	103	173	116
	4EVE	164	93	144	100
	Mean	162	113	168	123
	S.D.	3.8	26.5	22.4	27.7
	N	3	3	3	3
G 5 / F High 0.075 mg/L	5AIE	96	116	171	126
	5CGE	140	74	127	87
	5CSG	178	119	173	132
	Mean	138	103	157	115
	S.D.	41.0	25.2	26.0	24.4
	N	3	3	3	3

Table C-6 – Individual Animal Heart Rate and Blood Pressure Data

FEMALES (DAY 1)

Dose Group	Animal Number	Heart Rate [Beats/Minute] day 1	Diastolic Blood Pressure [mmHg] day 1	Systolic Blood Pressure [mmHg] day 1	Mean Arterial Pressure [mmHg] day 1
G 1 / F Saline	1CBE	144	87	146	98
	1JKG	133	87	150	103
	1JSG	141	105	158	117
	Mean	139	93	151	106
	S.D.	5.7	10.4	6.1	9.8
	N	3	3	3	3
G 2 / F Vehicle	2AJE	151	98	146	109
	2ZQE	127	89	149	104
	2FLE	129	60	141	82
	Mean	136	82	145	98
	S.D.	13.3	19.9	4.0	14.4
	N	3	3	3	3
G 3 / F Low 0.019 mg/L	3CVG	117	101	139	111
	3FKE	80	68	82	71
	3ZRE	118	61	119	73
	Mean	105	77	113	85
	S.D.	21.7	21.4	28.9	22.5
	N	3	3	3	3
G 4 / F Mid 0.038 mg/L	4ACE	153	116	185	134
	4CAE	140	93	155	112
	4EVE	150	89	152	102
	Mean	148	99	164	116
	S.D.	6.8	14.6	18.2	16.4
	N	3	3	3	3
G 5 / F High 0.075 mg/L	5AIE	144	68	121	80
	5CGE	156	89	149	101
	5CSG	99	82	138	95
	Mean	133	80	136	92
	S.D.	30.0	10.7	14.1	10.8
	N	3	3	3	3

Table C-6 – Individual Animal Heart Rate and Blood Pressure Data

FEMALES (DAY 2)

Dose Group	Animal Number	Heart Rate [Beats/Minute] day 2	Diastolic Blood Pressure [mmHg] day 2	Systolic Blood Pressure [mmHg] day 2	Mean Arterial Pressure [mmHg] day 2
G 1 / F Saline	1CBE	160	81	132	93
	1JKG	163	78	139	96
	1JSG	151	82	132	91
	Mean	158	80	134	93
	S.D.	6.2	2.1	4.0	2.5
	N	3	3	3	3
G 2 / F Vehicle	2AJE	151	82	142	101
	2ZQE	128	81	121	85
	2FLE	131	72	134	86
	Mean	137	78	132	91
	S.D.	12.5	5.5	10.6	9.0
	N	3	3	3	3
G 3 / F Low 0.019 mg/L	3CVG	125	44	85	55
	3FKE	121	78	119	88
	3ZRE	167	119	167	126
	Mean	138	80	124	90
	S.D.	25.5	37.6	41.2	35.5
	N	3	3	3	3
G 4 / F Mid 0.038 mg/L	4ACE	146	53	128	76
	4CAE	161	59	116	71
	4EVE	153	81	159	104
	Mean	153	64	134	84
	S.D.	7.5	14.7	22.2	17.8
	N	3	3	3	3
G 5 / F High 0.075 mg/L	5AIE	156	45	115	57
	5CGE	160	96	154	108
	5CSG	150	126	182	140
	Mean	155	89	150	102
	S.D.	5.0	41.0	33.7	41.9
	N	3	3	3	3

Table C-6 – Individual Animal Heart Rate and Blood Pressure Data

FEMALES (DAY 3)

Dose Group	Animal Number	Heart Rate [Beats/Minute] day 3	Diastolic Blood Pressure [mmHg] day 3	Systolic Blood Pressure [mmHg] day 3	Mean Arterial Pressure [mmHg] day 3
G 1 / F Saline	1CBE	163	117	145	125
	1JKG	136	64	139	75
	1JSG	146	116	145	118
	Mean	148	99	143	106
	S.D.	13.7	30.3	3.5	27.1
	N	3	3	3	3
G 2 / F Vehicle	2AJE	145	49	156	81
	2ZQE	140	51	147	73
	2FLE	96	51	66	55
	Mean	127	50	123	70
	S.D.	27.0	1.2	49.6	13.3
	N	3	3	3	3
G 3 / F Low 0.019 mg/L	3CVG	137	89	149	99
	3FKE	98	79	116	87
	3ZRE	153	125	161	133
	Mean	129	98	142	106
	S.D.	28.3	24.2	23.3	23.9
	N	3	3	3	3
G 4 / F Mid 0.038 mg/L	4ACE	147	118	163	128
	4CAE	163	93	138	98
	4EVE	151	92	148	107
	Mean	154	101	150	111
	S.D.	8.3	14.7	12.6	15.4
	N	3	3	3	3
G 5 / F High 0.075 mg/L	5AIE	130	78	143	94
	5CGE	156	77	132	92
	5CSG	139	124	177	135
	Mean	142	93	151	107
	S.D.	13.2	26.9	23.5	24.3
	N	3	3	3	3

Table C-6 – Individual Animal Heart Rate and Blood Pressure Data

FEMALES (DAY 4)

Dose Group	Animal Number	Heart Rate [Beats/Minute] day 4	Diastolic Blood Pressure [mmHg] day 4	Systolic Blood Pressure [mmHg] day 4	Mean Arterial Pressure [mmHg] day 4
G 1 / F Saline	1CBE	172	134	193	143
	1JKG	137	104	172	118
	1JSG	139	139	168	147
	Mean	149	126	178	136
	S.D.	19.7	18.9	13.4	15.7
	N	3	3	3	3
G 2 / F Vehicle	2AJE	165	83	168	106
	2ZQE	154	82	142	87
	2FLE	113	82	140	94
	Mean	144	82	150	96
	S.D.	27.4	0.6	15.6	9.6
	N	3	3	3	3
G 3 / F Low 0.019 mg/L	3CVG	131	94	176	114
	3FKE	123	57	124	71
	3ZRE	163	78	141	83
	Mean	139	76	147	89
	S.D.	21.2	18.6	26.5	22.2
	N	3	3	3	3
G 4 / F Mid 0.038 mg/L	4ACE	156	111	196	131
	4CAE	155	108	204	124
	4EVE	149	70	143	90
	Mean	153	96	181	115
	S.D.	3.8	22.9	33.2	21.9
	N	3	3	3	3
G 5 / F High 0.075 mg/L	5AIE	161	82	148	97
	5CGE	165	85	149	108
	5CSG	94	62	82	65
	Mean	140	76	126	90
	S.D.	39.9	12.5	38.4	22.3
	N	3	3	3	3

Table C-6 – Individual Animal Heart Rate and Blood Pressure Data

FEMALES (DAY 5)

Dose Group	Animal Number	Heart Rate [Beats/Minute] day 5	Diastolic Blood Pressure [mmHg] day 5	Systolic Blood Pressure [mmHg] day 5	Mean Arterial Pressure [mmHg] day 5
G 1 / F Saline	1CBE	141	96	166	116
	1JKG	151	79	139	84
	1JSG	157	82	131	96
	Mean	150	86	145	99
	S.D.	8.1	9.1	18.3	16.2
	N	3	3	3	3
G 2 / F Vehicle	2AJE	148	51	72	55
	2ZQE	159	92	157	96
	2FLE	141	87	140	95
	Mean	149	77	123	82
	S.D.	9.1	22.4	45.0	23.4
	N	3	3	3	3
G 3 / F Low 0.019 mg/L	3CVG	149	108	161	121
	3FKE	142	85	142	98
	3ZRE	140	88	150	113
	Mean	144	94	151	111
	S.D.	4.7	12.5	9.5	11.7
	N	3	3	3	3
G 4 / F Mid 0.038 mg/L	4ACE	134	104	168	118
	4CAE	142	44	75	51
	4EVE	123	48	114	62
	Mean	133	65	119	77
	S.D.	9.5	33.5	46.7	35.9
	N	3	3	3	3
G 5 / F High 0.075 mg/L	5AIE	135	87	160	99
	5CGE	150	56	135	71
	5CSG	136	58	113	65
	Mean	140	67	136	78
	S.D.	8.4	17.3	23.5	18.1
	N	3	3	3	3

Table C-6 – Individual Animal Heart Rate and Blood Pressure Data

FEMALES (DAY 6)

Dose Group	Animal Number	Heart Rate [Beats/Minute] day 6	Diastolic Blood Pressure [mmHg] day 6	Systolic Blood Pressure [mmHg] day 6	Mean Arterial Pressure [mmHg] day 6
G 1 / F Saline	1CBE	163	95	166	115
	1JKG	171	55	175	72
	1JSG	142	81	155	91
	Mean	159	77	165	93
	S.D.	15.0	20.3	10.0	21.5
	N	3	3	3	3
G 2 / F Vehicle	2AJE	164	97	166	118
	2ZQE	85	56	77	60
	2FLE	121	57	111	69
	Mean	123	70	118	82
	S.D.	39.6	23.4	44.9	31.2
	N	3	3	3	3
G 3 / F Low 0.019 mg/L	3CVG	136	97	137	105
	3FKE	123	78	125	87
	3ZRE	158	141	181	149
	Mean	139	105	148	114
	S.D.	17.7	32.3	29.5	31.9
	N	3	3	3	3
G 4 / F Mid 0.038 mg/L	4ACE	153	94	140	104
	4CAE	146	115	170	123
	4EVE	153	150	203	159
	Mean	151	120	171	129
	S.D.	4.0	28.3	31.5	27.9
	N	3	3	3	3
G 5 / F High 0.075 mg/L	5AIE	181	134	197	138
	5CGE	170	74	131	88
	5CSG	148	126	192	137
	Mean	166	111	173	121
	S.D.	16.8	32.6	36.7	28.6
	N	3	3	3	3

Table C-6 – Individual Animal Heart Rate and Blood Pressure Data

FEMALES (DAY 7)

Dose Group	Animal Number	Heart Rate [Beats/Minute] day 7	Diastolic Blood Pressure [mmHg] day 7	Systolic Blood Pressure [mmHg] day 7	Mean Arterial Pressure [mmHg] day 7
G 1 / F Saline	1CBE	158	106	167	122
	1JKG	160	62	166	84
	1JSG	147	100	132	108
	Mean	155	89	155	105
	S.D.	7.0	23.9	19.9	19.2
	N	3	3	3	3
G 2 / F Vehicle	2AJE	155	69	129	85
	2ZQE	144	95	114	101
	2FLE	146	62	121	68
	Mean	148	75	121	85
	S.D.	5.9	17.4	7.5	16.5
	N	3	3	3	3
G 3 / F Low 0.019 mg/L	3CVG	137	128	183	139
	3FKE	114	71	137	82
	3ZRE	136	108	153	118
	Mean	129	102	158	113
	S.D.	13.0	28.9	23.4	28.8
	N	3	3	3	3
G 4 / F Mid 0.038 mg/L	4ACE	154	100	141	116
	4CAE	160	103	148	125
	4EVE	153	100	133	107
	Mean	156	101	141	116
	S.D.	3.8	1.7	7.5	9.0
	N	3	3	3	3
G 5 / F High 0.075 mg/L	5AIE	150	94	159	114
	5CGE	167	93	152	99
	5CSG	125	102	160	117
	Mean	147	96	157	110
	S.D.	21.1	4.9	4.4	9.6
	N	3	3	3	3

Table C-6 – Individual Animal Heart Rate and Blood Pressure Data

FEMALES (DAY 8)

Dose Group	Animal Number	Heart Rate [Beats/Minute] day 8	Diastolic Blood Pressure [mmHg] day 8	Systolic Blood Pressure [mmHg] day 8	Mean Arterial Pressure [mmHg] day 8
G 1 / F Saline	1CBE	169	100	143	110
	1JKG	162	76	156	101
	1JSG	163	78	150	97
	Mean	165	85	150	103
	S.D.	3.8	13.3	6.5	6.7
	N	3	3	3	3
G 2 / F Vehicle	2AJE	156	80	145	91
	2ZQE	138	79	132	98
	2FLE	145	74	128	89
	Mean	146	78	135	93
	S.D.	9.1	3.2	8.9	4.7
	N	3	3	3	3
G 3 / F Low 0.019 mg/L	3CVG	137	81	154	88
	3FKE	96	86	161	93
	3ZRE	117	86	157	100
	Mean	117	84	157	94
	S.D.	20.5	2.9	3.5	6.0
	N	3	3	3	3
G 4 / F Mid 0.038 mg/L	4ACE	138	69	135	74
	4CAE	168	140	173	148
	4EVE	141	68	117	78
	Mean	149	92	142	100
	S.D.	16.5	41.3	28.6	41.6
	N	3	3	3	3
G 5 / F High 0.075 mg/L	5AIE	149	87	155	102
	5CGE	170	84	146	97
	5CSG	128	51	146	74
	Mean	149	74	149	91
	S.D.	21.0	20.0	5.2	14.9
	N	3	3	3	3

Table C-6 – Individual Animal Heart Rate and Blood Pressure Data

FEMALES (DAY 9)

Dose Group	Animal Number	Heart Rate [Beats/Minute] day 9	Diastolic Blood Pressure [mmHg] day 9	Systolic Blood Pressure [mmHg] day 9	Mean Arterial Pressure [mmHg] day 9
G 1 / F Saline	1CBE	153	82	142	96
	1JKG	146	69	131	82
	1JSG	156	91	140	112
	Mean	152	81	138	97
	S.D.	5.1	11.1	5.9	15.0
	N	3	3	3	3
G 2 / F Vehicle	2AJE	154	94	134	102
	2ZQE	124	93	133	100
	2FLE	136	93	132	100
	Mean	138	93	133	101
	S.D.	15.1	0.6	1.0	1.2
	N	3	3	3	3
G 3 / F Low 0.019 mg/L	3CVG	137	102	168	118
	3FKE	104	67	126	76
	3ZRE	178	98	183	121
	Mean	140	89	159	105
	S.D.	37.1	19.2	29.5	25.2
	N	3	3	3	3
G 4 / F Mid 0.038 mg/L	4ACE	145	99	158	114
	4CAE	159	93	165	113
	4EVE	128	55	143	80
	Mean	144	82	155	102
	S.D.	15.5	23.9	11.2	19.3
	N	3	3	3	3
G 5 / F High 0.075 mg/L	5AIE	132	86	154	102
	5CGE	151	88	150	97
	5CSG	137	115	169	126
	Mean	140	96	158	108
	S.D.	9.8	16.2	10.0	15.5
	N	3	3	3	3

Table C-6 – Individual Animal Heart Rate and Blood Pressure Data

FEMALES (DAY 10)

Dose Group	Animal Number	Heart Rate [Beats/Minute] day 10	Diastolic Blood Pressure [mmHg] day 10	Systolic Blood Pressure [mmHg] day 10	Mean Arterial Pressure [mmHg] day 10
G 1 / F Saline	1CBE	100	72	102	79
	1JKG	161	94	152	104
	1JSG	144	95	148	107
	Mean	135	87	134	97
	S.D.	31.5	13.0	27.8	15.4
	N	3	3	3	3
G 2 / F Vehicle	2AJE	181	114	175	122
	2ZQE	147	90	164	106
	2FLE	132	79	141	94
	Mean	153	94	160	107
	S.D.	25.1	17.9	17.3	14.0
	N	3	3	3	3
G 3 / F Low 0.019 mg/L	3CVG	134	123	158	130
	3FKE	127	94	140	103
	3ZRE	159	99	149	108
	Mean	140	105	149	114
	S.D.	16.8	15.5	9.0	14.4
	N	3	3	3	3
G 4 / F Mid 0.038 mg/L	4ACE	154	106	170	121
	4CAE	177	90	181	115
	4EVE	136	108	152	119
	Mean	156	101	168	118
	S.D.	20.6	9.9	14.6	3.1
	N	3	3	3	3
G 5 / F High 0.075 mg/L	5AIE	193	98	163	115
	5CGE	166	78	135	87
	5CSG	157	126	175	136
	Mean	172	101	158	113
	S.D.	18.7	24.1	20.5	24.6
	N	3	3	3	3

Table C-6 – Individual Animal Heart Rate and Blood Pressure Data

FEMALES (DAY 11)

Dose Group	Animal Number	Heart Rate [Beats/Minute] day 11	Diastolic Blood Pressure [mmHg] day 11	Systolic Blood Pressure [mmHg] day 11	Mean Arterial Pressure [mmHg] day 11
G 1 / F Saline	1CBE	156	83	144	100
	1JKG	150	80	143	91
	1JSG	81	138	171	147
	Mean	129	100	153	113
	S.D.	41.7	32.7	15.9	30.1
	N	3	3	3	3
G 2 / F Vehicle	2AJE	182	75	149	92
	2ZQE	159	77	146	92
	2FLE	155	109	156	117
	Mean	165	87	150	100
	S.D.	14.6	19.1	5.1	14.4
	N	3	3	3	3
G 3 / F Low 0.019 mg/L	3CVG	171	103	170	118
	3FKE	131	32	140	64
	3ZRE	155	101	172	118
	Mean	152	79	161	100
	S.D.	20.1	40.4	17.9	31.2
	N	3	3	3	3
G 4 / F Mid 0.038 mg/L	4ACE	131	83	171	105
	4CAE	156	117	174	124
	4EVE	142	56	175	82
	Mean	143	85	173	104
	S.D.	12.5	30.6	2.1	21.0
	N	3	3	3	3
G 5 / F High 0.075 mg/L	5AIE	144	104	160	116
	5CGE	152	88	144	94
	5CSG	128	95	161	103
	Mean	141	96	155	104
	S.D.	12.2	8.0	9.5	11.1
	N	3	3	3	3

Table C-6 – Individual Animal Heart Rate and Blood Pressure Data

FEMALES (DAY 12)

Dose Group	Animal Number	Heart Rate [Beats/Minute] day 12	Diastolic Blood Pressure [mmHg] day 12	Systolic Blood Pressure [mmHg] day 12	Mean Arterial Pressure [mmHg] day 12
G 1 / F Saline	1CBE	142	81	126	83
	1JKG	162	85	148	97
	1JSG	136	84	137	91
	Mean	147	83	137	90
	S.D.	13.6	2.1	11.0	7.0
	N	3	3	3	3
G 2 / F Vehicle	2AJE	177	96	144	107
	2ZQE	139	59	128	77
	2FLE	121	63	127	79
	Mean	146	73	133	88
	S.D.	28.6	20.3	9.5	16.8
	N	3	3	3	3
G 3 / F Low 0.019 mg/L	3CVG	146	95	150	106
	3FKE	121	82	130	92
	3ZRE	148	92	152	103
	Mean	138	90	144	100
	S.D.	15.0	6.8	12.2	7.4
	N	3	3	3	3
G 4 / F Mid 0.038 mg/L	4ACE	185	110	205	136
	4CAE	170	74	167	97
	4EVE	129	49	121	68
	Mean	161	78	164	100
	S.D.	29.0	30.7	42.1	34.1
	N	3	3	3	3
G 5 / F High 0.075 mg/L	5AIE	158	101	152	112
	5CGE	162	86	149	99
	5CSG	138	105	146	115
	Mean	153	97	149	109
	S.D.	12.9	10.0	3.0	8.5
	N	3	3	3	3

Table C-6 – Individual Animal Heart Rate and Blood Pressure Data

FEMALES (DAY 13)

Dose Group	Animal Number	Heart Rate [Beats/Minute] day 13	Diastolic Blood Pressure [mmHg] day 13	Systolic Blood Pressure [mmHg] day 13	Mean Arterial Pressure [mmHg] day 13
G 1 / F Saline	1CBE	170	90	153	100
	1JKG	138	94	168	116
	1JSG	136	84	102	89
	Mean	148	89	141	102
	S.D.	19.1	5.0	34.6	13.6
	N	3	3	3	3
G 2 / F Vehicle	2AJE	166	93	145	104
	2ZQE	121	94	138	105
	2FLE	130	94	139	101
	Mean	139	94	141	103
	S.D.	23.8	0.6	3.8	2.1
	N	3	3	3	3
G 3 / F Low 0.019 mg/L	3CVG	156	103	147	110
	3FKE	138	61	130	75
	3ZRE	98	89	136	96
	Mean	131	84	138	94
	S.D.	29.7	21.4	8.6	17.6
	N	3	3	3	3
G 4 / F Mid 0.038 mg/L	4ACE	131	81	113	88
	4CAE	148	92	160	102
	4EVE	152	51	132	64
	Mean	144	75	135	85
	S.D.	11.2	21.2	23.6	19.2
	N	3	3	3	3
G 5 / F High 0.075 mg/L	5AIE	158	82	148	95
	5CGE	146	84	146	99
	5CSG	146	103	144	109
	Mean	150	90	146	101
	S.D.	6.9	11.6	2.0	7.2
	N	3	3	3	3

Table C-6 – Individual Animal Heart Rate and Blood Pressure Data

FEMALES (DAY 14)

Dose Group	Animal Number	Heart Rate [Beats/Minute] day 14	Diastolic Blood Pressure [mmHg] day 14	Systolic Blood Pressure [mmHg] day 14	Mean Arterial Pressure [mmHg] day 14
G 1 / F Saline	1CBE	134	119	151	128
	1JKG	174	84	135	95
	1JSG	153	84	136	98
	Mean	154	96	141	107
	S.D.	20.0	20.2	9.0	18.2
	N	3	3	3	3
G 2 / F Vehicle	2AJE	153	92	137	108
	2ZQE	143	87	134	98
	2FLE	124	84	125	94
	Mean	140	88	132	100
	S.D.	14.7	4.0	6.2	7.2
	N	3	3	3	3
G 3 / F Low 0.019 mg/L	3CVG	145	125	157	134
	3FKE	115	56	128	74
	3ZRE	146	88	153	106
	Mean	135	90	146	105
	S.D.	17.6	34.5	15.7	30.0
	N	3	3	3	3
G 4 / F Mid 0.038 mg/L	4ACE	143	89	162	107
	4CAE	167	98	144	108
	4EVE	132	62	146	87
	Mean	147	83	151	101
	S.D.	17.9	18.7	9.9	11.8
	N	3	3	3	3
G 5 / F High 0.075 mg/L	5AIE	153	85	147	103
	5CGE	162	84	149	97
	5CSG	126	75	144	92
	Mean	147	81	147	97
	S.D.	18.7	5.5	2.5	5.5
	N	3	3	3	3

Table C-7 – Individual Animal Body Weights
(kg)

MALES

Dose Group	Animal Number	day 1	day 4	day 8	day 11
G 1 / M Saline	1EZF	8.50	8.24	8.04	8.18
	1FUH	10.02	9.80	9.72	9.92
	1UYF	9.20	9.00	9.16	9.36
	Mean	9.24	9.01	8.97	9.15
	S.D.	0.761	0.780	0.855	0.888
	N	3	3	3	3
G 2 / M Vehicle	2AGF	8.60	8.52	8.40	8.62
	2BSF	8.72	8.76	8.70	9.08
	2FRH	9.00	9.18	9.40	9.46
	Mean	8.77	8.82	8.83	9.05
	S.D.	0.205	0.334	0.513	0.421
	N	3	3	3	3
G 3 / M Low 0.019 mg/L	3DWH	8.96	9.08	8.90	9.20
	3THF	8.88	8.94	8.70	8.98
	3TYF	8.32	8.28	8.42	8.60
	Mean	8.72	8.77	8.67	8.93
	S.D.	0.349	0.427	0.241	0.304
	N	3	3	3	3
G 4 / M Mid 0.038 mg/L	4EAH	8.04	8.44	8.48	8.36
	4JLH	9.22	8.88	8.92	8.76
	4YJF	9.38	9.22	9.26	9.28
	Mean	8.88	8.85	8.89	8.80
	S.D.	0.732	0.391	0.391	0.461
	N	3	3	3	3
G 5 / M High 0.075 mg/L	5GPH	8.86	8.90	9.02	8.96
	5ZOF	8.92	9.12	9.22	8.96
	5ZTF	8.70	8.54	8.62	8.64
	Mean	8.83	8.85	8.95	8.85
	S.D.	0.114	0.293	0.306	0.185
	N	3	3	3	3

NOTE: Body weights collected on Days 1 and 11 were collected prior to the first exposure.

Table C-7 – Individual Animal Body Weights
(kg)

FEMALES

Dose Group	Animal Number	day 1	day 4	day 8	day 11
G 1 / F Saline	1CBE	6.32	6.46	6.36	6.40
	1JKG	9.12	9.02	9.04	9.04
	1JSG	7.04	7.16	7.02	7.06
	Mean	7.49	7.55	7.47	7.50
	S.D.	1.454	1.323	1.396	1.374
	N	3	3	3	3
G 2 / F Vehicle	2AJE	6.42	6.90	6.84	7.00
	2ZQE	8.02	8.06	8.02	7.90
	2FLE	6.84	6.86	6.82	6.94
	Mean	7.09	7.27	7.23	7.28
	S.D.	0.830	0.682	0.687	0.538
	N	3	3	3	3
G 3 / F Low 0.019 mg/L	3CVG	7.58	7.56	7.48	7.56
	3FKE	6.12	6.06	6.10	6.20
	3ZRE	6.76	6.58	6.70	6.64
	Mean	6.82	6.73	6.76	6.80
	S.D.	0.732	0.762	0.692	0.694
	N	3	3	3	3
G 4 / F Mid 0.038 mg/L	4ACE	7.30	7.30	7.36	7.36
	4CAE	6.38	6.24	6.16	6.16
	4EVE	6.96	6.88	7.02	7.08
	Mean	6.88	6.81	6.85	6.87
	S.D.	0.465	0.534	0.618	0.628
	N	3	3	3	3
G 5 / F High 0.075 mg/L	5AIE	7.14	7.30	7.18	7.32
	5CGE	6.54	6.74	6.64	6.82
	5CSG	8.24	8.32	8.18	8.36
	Mean	7.31	7.45	7.33	7.50
	S.D.	0.862	0.801	0.781	0.786
	N	3	3	3	3

NOTE: Body weights collected on Days 1 and 11 were collected prior to the first exposure.

Table C-8 – Individual Animal Body Weight Change
(kg)

MALES

Dose Group	Animal Number	d 1 to 4	d 4 to 8	d 8 to 11	d 1 to 11
G 1 / M Saline	1EZF	-0.26	-0.20	0.14	-0.32
	1FUH	-0.22	-0.08	0.20	-0.10
	1UYF	-0.20	0.16	0.20	0.16
	Mean	-0.23	-0.04	0.18	-0.09
	S.D.	0.031	0.183	0.035	0.240
	N	3	3	3	3
G 2 / M Vehicle	2AGF	-0.08	-0.12	0.22	0.02
	2BSF	0.04	-0.06	0.38	0.36
	2FRH	0.18	0.22	0.06	0.46
	Mean	0.05	0.01	0.22	0.28
	S.D.	0.130	0.181	0.160	0.231
	N	3	3	3	3
G 3 / M Low 0.019 mg/L	3DWH	0.12	-0.18	0.30	0.24
	3THF	0.06	-0.24	0.28	0.10
	3TYF	-0.04	0.14	0.18	0.28
	Mean	0.05	-0.09	0.25	0.21
	S.D.	0.081	0.204	0.064	0.095
	N	3	3	3	3
G 4 / M Mid 0.038 mg/L	4EAH	0.40	0.04	-0.12	0.32
	4JLH	-0.34	0.04	-0.16	-0.46
	4YJF	-0.16	0.04	0.02	-0.10
	Mean	-0.03	0.04	-0.09	-0.08
	S.D.	0.386	0.000	0.095	0.390
	N	3	3	3	3
G 5 / M High 0.075 mg/L	5GPH	0.04	0.12	-0.06	0.10
	5ZOF	0.20	0.10	-0.26	0.04
	5ZTF	-0.16	0.08	0.02	-0.06
	Mean	0.03	0.10	-0.10	0.03
	S.D.	0.180	0.020	0.144	0.081
	N	3	3	3	3

d = day

NOTE: Body weights collected on Days 1 and 11 were collected prior to the first exposure.

Table C-8 – Individual Animal Body Weight Change
(kg)

FEMALES

Dose Group	Animal Number	d 1 to 4	d 4 to 8	d 8 to 11	d 1 to 11
G 1 / F Saline	1CBE	0.14	-0.10	0.04	0.08
	1JKG	-0.10	0.02	0.00	-0.08
	1JSG	0.12	-0.14	0.04	0.02
	Mean	0.05	-0.07	0.03	0.01
	S.D.	0.133	0.083	0.023	0.081
	N	3	3	3	3
G 2 / F Vehicle	2AJE	0.48	-0.06	0.16	0.58
	2ZQE	0.04	-0.04	-0.12	-0.12
	2FLE	0.02	-0.04	0.12	0.10
	Mean	0.18	-0.05	0.05	0.19
	S.D.	0.260	0.012	0.151	0.358
	N	3	3	3	3
G 3 / F Low 0.019 mg/L	3CVG	-0.02	-0.08	0.08	-0.02
	3FKE	-0.06	0.04	0.10	0.08
	3ZRE	-0.18	0.12	-0.06	-0.12
	Mean	-0.09	0.03	0.04	-0.02
	S.D.	0.083	0.101	0.087	0.100
	N	3	3	3	3
G 4 / F Mid 0.038 mg/L	4ACE	0.00	0.06	0.00	0.06
	4CAE	-0.14	-0.08	0.00	-0.22
	4EVE	-0.08	0.14	0.06	0.12
	Mean	-0.07	0.04	0.02	-0.01
	S.D.	0.070	0.111	0.035	0.181
	N	3	3	3	3
G 5 / F High 0.075 mg/L	5AIE	0.16	-0.12	0.14	0.18
	5CGE	0.20	-0.10	0.18	0.28
	5CSG	0.08	-0.14	0.18	0.12
	Mean	0.15	-0.12	0.17	0.19
	S.D.	0.061	0.020	0.023	0.081
	N	3	3	3	3

d = day

NOTE: Body weights collected on Days 1 and 11 were collected prior to the first exposure.

Table C-9 – Individual Animal Food Consumption Data
(g)

MALES (PRE-TEST)

Group	Animals	d -3 to -2
G 1 / M Saline	1EZF, 1FUH	800
	1UYF	400
G 2 / M Vehicle	2BSF, 2AGF	800
	2FRH	400
G 3 / M Low 0.019 mg/L	3THF, 3DWH	800
	3TYF	400
G 4 / M Mid 0.038 mg/L	4EAH, 4JLH	800
	4YJF	400
G 5 / M High 0.075 mg/L	5GPH, 5ZOF	800
	5ZTF	400

d = day

NOTE: Dogs were single or double-housed throughout the study. In the above table, double-housed dogs are listed as a single entry. The food consumption values listed are the total amounts of food consumed per pen, i.e., out of a total of 400 g for single housed dogs and out of a total of 800 g for double-housed dogs.

Table C-9 – Individual Animal Food Consumption Data
(g)

MALES (DAYS 1-14)

Group	Animals	d 1 to 2	d 2 to 3	d 3 to 4	d 4 to 5	d 5 to 6	d 6 to 7	d 7 to 8
G 1 / M Saline	1EZF, 1FUH	800	800	800	800	800	800	800
	1UYF	400	400	400	400	400	400	400
G 2 / M Vehicle	2BSF, 2AGF	800	591	800	486	468	800	645
	2FRH	400	400	400	400	400	400	400
G 3 / M Low 0.019 mg/L	3THF, 3DWH	800	800	800	800	800	800	631
	3TYF	400	400	400	400	400	400	400
G 4 / M Mid 0.038 mg/L	4EAH, 4JLH	800	800	800	603	800	689	689
	4YJF	400	400	400	400	400	400	400
G 5 / M High 0.075 mg/L	5GPH, 5ZOF	800	800	800	628	800	800	800
	5ZTF	400	400	237	254	280	201	400

Group	Animals	d 8 to 9	d 9 to 10	d 10 to 11	d 11 to 12	d 12 to 13	d 13 to 14	d 14 to 15
G 1 / M Saline	1EZF, 1FUH	800	800	800	800	800	800	800
	1UYF	400	400	400	400	400	269	169
G 2 / M Vehicle	2BSF, 2AGF	671	669	800	800	800	677	80
	2FRH	400	400	400	400	400	400	254
G 3 / M Low 0.019 mg/L	3THF, 3DWH	800	800	800	600	613	800	282
	3TYF	400	400	400	400	400	400	400
G 4 / M Mid 0.038 mg/L	4EAH, 4JLH	699	601	800	800	800	675	276
	4YJF	400	267	400	400	221	400	61
G 5 / M High 0.075 mg/L	5GPH, 5ZOF	578	800	486	800	590	800	254
	5ZTF	256	306	400	400	400	400	77

d = day

NOTE: Dogs were single or double-housed throughout the study. In the above table, double-housed dogs are listed as a single entry. The food consumption values listed are the total amounts of food consumed per pen, i.e., out of a total of 400 g for single housed dogs and out of a total of 800 g for double-housed dogs.

Table C-9 – Individual Animal Food Consumption Data
(g)

FEMALES (PRE-TEST)

Group	Animals	d -4 to -3
G 1 / F Saline	1JKG, 1CBE	800
	1JSG	265
G 2 / F Vehicle	2ZQE, 2AJE	800
	2FLE	400
G 3 / F Low 0.019 mg/L	3ZRE	400
	3CVG, 3FKE	662
G 4 / F Mid 0.038 mg/L	4CAE, 4ACE	800
	4EVE	400
G 5 / F High 0.075 mg/L	5AIE, 5CGE	800
	5CSG	120

d = day

NOTE: Dogs were single or double-housed throughout the study. In the above table, double-housed dogs are listed as a single entry. The food consumption values listed are the total amounts of food consumed per pen, i.e., out of a total of 400 g for single housed dogs and out of a total of 800 g for double-housed dogs.

Table C-9 – Individual Animal Food Consumption Data
(g)

FEMALES (DAYS 1-14)

Group	Animals	d 1 to 2	d 2 to 3	d 3 to 4	d 4 to 5	d 5 to 6	d 6 to 7	d 7 to 8
G 1 / F Saline	1JKG, 1CBE	800	583	550	548	800	498	640
	1JSG	400	233	180	298	231	400	293
G 2 / F Vehicle	2ZQE, 2AJE	575	625	679	610	669	564	519
	2FLE	221	235	125	252	153	400	167
G 3 / F Low 0.019 mg/L	3ZRE	209	308	178	254	232	400	268
	3CVG, 3FKE	800	527	489	662	492	556	550
G 4 / F Mid 0.038 mg/L	4CAE, 4ACE	800	542	520	614	524	569	475
	4EVE	400	293	258	400	400	311	400
G 5 / F High 0.075 mg/L	5AIE, 5CGE	800	800	800	800	800	800	633
	5CSG	400	400	242	262	400	400	300

Group	Animals	d 8 to 9	d 9 to 10	d 10 to 11	d 11 to 12	d 12 to 13	d 13 to 14	d 14 to 15
G 1 / F Saline	1JKG, 1CBE	800	616	512	800	800	615	235
	1JSG	224	400	255	400	243	250	49
G 2 / F Vehicle	2ZQE, 2AJE	586	571	510	800	559	580	31
	2FLE	291	183	400	212	400	220	37
G 3 / F Low 0.019 mg/L	3ZRE	148	250	400	219	228	249	144
	3CVG, 3FKE	537	548	558	566	567	608	588
G 4 / F Mid 0.038 mg/L	4CAE, 4ACE	609	550	471	485	504	409	250
	4EVE	298	400	314	400	400	268	89
G 5 / F High 0.075 mg/L	5AIE, 5CGE	684	800	656	800	800	800	237
	5CSG	400	400	400	270	400	400	240

d = day

NOTE: Dogs were single or double-housed throughout the study. In the above table, double-housed dogs are listed as a single entry. The food consumption values listed are the total amounts of food consumed per pen, i.e., out of a total of 400 g for single housed dogs and out of a total of 800 g for double-housed dogs.

Table C-10 – Individual Animal Respiratory Function Data
(Respiratory Rate, Tidal Volume and Minute Volume)

GROUP 1 (SALINE CONTROL) MALES

Study Day	Individual Animal Data			Daily Summary Data		
	1EZF	1FUH	1UYF	Mean	S.D.	N
Respiratory Rate (Breaths/Minute)						
1	24.4	25.4	61.3	37.0	21.0	3
2	29.5	34.1	35.5	33.0	3.1	3
3	31.8	37.9	42.8	37.5	5.5	3
4	22.7	33.3	53.0	36.3	15.4	3
5	34.9	41.3	51.0	42.4	8.1	3
6	27.2	28.8	64.3	40.1	21.0	3
7	29.0	46.4	44.5	40.0	9.5	3
8	30.5	121.6	100.3	84.1	47.6	3
9	43.3	44.3	148.7	78.7	60.6	3
10	34.7	38.3	69.3	47.4	19.0	3
11	31.1	33.0	40.7	34.9	5.1	3
12	49.2	41.7	32.9	41.3	8.1	3
13	27.0	32.8	38.3	32.7	5.7	3
14	22.2	36.9	32.8	30.6	7.6	3
Tidal Volume (mL/Breath)						
1	188	150	225	188	37	3
2	221	354	169	248	96	3
3	135	211	170	172	38	3
4	231	227	168	209	35	3
5	153	276	186	205	64	3
6	241	266	281	263	20	3
7	184	242	260	229	39	3
8	262	177	80	173	91	3
9	161	149	29	113	73	3
10	368	135	98	200	146	3
11	173	111	274	186	82	3
12	119	328	144	197	114	3
13	250	138	222	204	59	3
14	279	156	232	222	62	3
Minute Volume (mL/Minute)						
1	4608	3819	13810	7412	5554	3
2	6371	12078	6001	8150	3407	3
3	4279	7983	7289	6517	1969	3
4	5228	7562	8931	7240	1872	3
5	5333	11414	9488	8745	3108	3
6	6543	7661	18092	10765	6370	3
7	5352	11253	11548	9384	3495	3
8	7851	22535	7959	12781	8447	3
9	6848	6492	4088	5809	1501	3
10	12770	5066	6778	8205	4045	3
11	5373	3660	11154	6729	3927	3
12	5852	13658	4731	8080	4863	3
13	6767	4491	8522	6593	2021	3
14	6185	5744	7599	6509	969	3

Table C-10 – Individual Animal Respiratory Function Data
(Respiratory Rate, Tidal Volume and Minute Volume)

GROUP 1 (SALINE CONTROL) FEMALES

Study Day	Individual Animal Data			Daily Summary Data		
	1CBE	1JKG	1JSG	Mean	S.D.	N
Respiratory Rate (Breaths/Minute)						
1	53.0	32.1	90.4	58.5	29.6	3
2	63.9	23.2	60.4	49.2	22.5	3
3	66.0	41.0	225.5	110.8	100.1	3
4	68.2	207.9	24.8	100.3	95.7	3
5	37.3	45.2	112.2	64.9	41.1	3
6	68.9	63.8	197.3	110.0	75.6	3
7	51.3	50.0	52.7	51.3	1.4	3
8	106.9	50.7	257.3	138.3	106.8	3
9	79.1	42.8	113.5	78.5	35.3	3
10	69.3	30.9	150.7	83.6	61.2	3
11	76.7	36.5	149.2	87.5	57.1	3
12	56.9	43.2	56.8	52.3	7.9	3
13	86.6	43.5	103.9	78.0	31.1	3
14	104.9	26.2	—[a]	65.6	55.7	2
Tidal Volume (mL/Breath)						
1	79	334	100	171	141	3
2	131	166	47	114	61	3
3	92	269	58	140	113	3
4	119	64	108	97	29	3
5	101	212	116	143	60	3
6	171	145	54	123	61	3
7	85	112	129	109	22	3
8	52	309	38	133	152	3
9	202	118	92	138	57	3
10	203	179	73	152	69	3
11	112	272	57	147	112	3
12	176	321	205	234	77	3
13	124	144	96	121	24	3
14	108	248	—[a]	178	99	2
Minute Volume (mL/Minute)						
1	4135	10674	9053	7954	3405	3
2	8337	3838	2830	5001	2932	3
3	6104	11037	13103	10081	3596	3
4	7995	13247	2665	7969	5291	3
5	3747	9554	13001	8767	4677	3
6	11766	9224	10164	10385	1285	3
7	4360	5616	6845	5607	1243	3
8	5520	15616	9886	10341	5063	3
9	15975	5069	10462	10502	5453	3
10	14098	5516	10889	10168	4336	3
11	8611	9951	8458	9007	821	3
12	9982	13851	11527	11787	1947	3
13	10763	6248	10007	9006	2418	3
14	11349	6492	—[a]	8921	3435	2

[a] Data not available due to unstable signal.

Table C-10 – Individual Animal Respiratory Function Data
(Respiratory Rate, Tidal Volume and Minute Volume)

GROUP 2 (VEHICLE CONTROL) MALES

Study Day	Individual Animal Data			Daily Summary Data		
	**2AGF**	**2BSF**	**2FRH**	**Mean**	**S.D.**	**N**
Respiratory Rate (Breaths/Minute)						
1	56.3	55.7	37.3	49.8	10.8	3
2	68.9	68.5	49.1	62.2	11.3	3
3	50.5	99.8	86.6	79.0	25.5	3
4	41.6	111.2	45.6	66.2	39.1	3
5	38.2	58.3	35.0	43.8	12.6	3
6	54.8	80.4	37.7	57.6	21.5	3
7	149.4	60.0	47.8	85.8	55.4	3
8	255.2	70.3	29.4	118.3	120.3	3
9	158.1	90.1	185.5	144.6	49.1	3
10	47.6	128.3	34.5	70.1	50.8	3
11	33.6	74.7	27.8	45.4	25.6	3
12	134.9	33.0	75.6	81.2	51.2	3
13	71.6	45.7	47.1	54.8	14.5	3
14	28.9	84.2	34.1	49.0	30.5	3
Tidal Volume (mL/Breath)						
1	73	167	154	131	51	3
2	113	262	147	174	78	3
3	148	157	224	176	42	3
4	122	147	162	144	20	3
5	222	239	149	203	48	3
6	212	138	256	202	60	3
7	84	492	178	251	214	3
8	55	153	212	140	79	3
9	23	147	26	65	71	3
10	362	79	117	186	153	3
11	150	146	113	136	20	3
12	79	114	133	109	27	3
13	250	145	57	151	97	3
14	178	191	115	161	41	3
Minute Volume (mL/Minute)						
1	4116	9307	5747	6390	2655	3
2	7813	17861	7232	10969	5976	3
3	7367	15604	15341	12771	4682	3
4	5071	16314	7401	9595	5934	3
5	8472	13959	5198	9210	4427	3
6	11624	10749	9628	10667	1000	3
7	12481	29471	8465	16806	11151	3
8	13779	10696	6195	10223	3814	3
9	3730	13243	4731	7235	5228	3
10	17220	9225	4073	10173	6625	3
11	5038	10791	3152	6327	3979	3
12	10589	3741	10019	8116	3800	3
13	17860	6656	2474	8997	7955	3
14	5154	16066	3903	8374	6690	3

Table C-10 – Individual Animal Respiratory Function Data
(Respiratory Rate, Tidal Volume and Minute Volume)

GROUP 2 (VEHICLE CONTROL) FEMALES

Study Day	Individual Animal Data			Daily Summary Data		
	**2AJE**	**2FLE**	**2ZQE**	**Mean**	**S.D.**	**N**
Respiratory Rate (Breaths/Minute)						
1	51.1	37.4	44.8	44.4	6.8	3
2	101.6	73.4	94.2	89.7	14.6	3
3	40.2	52.3	75.3	55.9	17.8	3
4	54.4	82.3	60.9	65.9	14.6	3
5	87.2	55.2	53.3	65.3	19.0	3
6	104.8	42.0	47.5	64.8	34.8	3
7	81.9	81.6	87.8	83.7	3.5	3
8	124.5	40.1	58.7	74.4	44.3	3
9	330.4	55.4	44.5	143.4	162.0	3
10	202.2	54.4	49.0	101.9	86.9	3
11	236.5	153.5	111.4	167.1	63.6	3
12	345.2	58.8	53.8	152.6	166.8	3
13	59.4	47.4	60.7	55.8	7.4	3
14	64.8	55.9	31.4	50.7	17.3	3
Tidal Volume (mL/Breath)						
1	271	226	257	251	23	3
2	147	82	70	100	41	3
3	134	246	195	192	56	3
4	123	98	193	138	49	3
5	79	288	180	182	105	3
6	82	138	120	114	29	3
7	73	45	103	74	29	3
8	72	297	299	223	131	3
9	31	120	336	162	157	3
10	96	137	87	107	27	3
11	24	40	72	46	24	3
12	51	278	102	144	119	3
13	244	308	125	226	93	3
14	264	275	124	221	85	3
Minute Volume (mL/Minute)						
1	13798	8455	11503	11252	2680	3
2	14913	5634	6620	9056	5097	3
3	5366	12876	14705	10982	4949	3
4	6658	8064	11738	8820	2623	3
5	6874	15907	9574	10785	4637	3
6	8567	5820	5683	6690	1627	3
7	5997	3631	8530	6053	2450	3
8	8852	11930	17538	12774	4404	3
9	10129	6635	14943	10569	4172	3
10	19182	7463	4162	10269	7894	3
11	5788	5375	8350	6504	1612	3
12	17792	16131	5352	13092	6754	3
13	14506	14579	7580	12222	4020	3
14	17107	15386	3875	12123	7194	3

Table C-10 – Individual Animal Respiratory Function Data
(Respiratory Rate, Tidal Volume and Minute Volume)

GROUP 3 (APN01; 0.019 mg/L) MALES

Study Day	Individual Animal Data			Daily Summary Data		
	3DWH	3THF	3TYF	Mean	S.D.	N
Respiratory Rate (Breaths/Minute)						
1	44.3	48.4	59.8	50.8	8.0	3
2	46.7	55.5	63.8	55.4	8.5	3
3	19.7	50.6	59.8	43.4	21.0	3
4	72.4	43.7	86.8	67.7	22.0	3
5	61.1	52.3	149.3	87.6	53.6	3
6	85.1	48.1	69.1	67.5	18.6	3
7	73.2	54.5	68.6	65.4	9.7	3
8	193.5	59.7	104.0	119.1	68.1	3
9	156.8	55.3	74.7	95.6	53.9	3
10	29.8	52.5	133.3	71.9	54.4	3
11	38.1	63.2	48.8	50.0	12.6	3
12	85.7	56.6	164.9	102.4	56.0	3
13	33.2	84.5	187.2	101.6	78.4	3
14	96.9	37.8	86.5	73.8	31.6	3
Tidal Volume (mL/Breath)						
1	310	338	235	294	53	3
2	339	383	336	353	27	3
3	204	318	243	255	58	3
4	185	152	111	150	37	3
5	291	394	107	264	146	3
6	184	328	255	256	72	3
7	211	246	155	204	46	3
8	56	290	109	152	122	3
9	66	146	252	155	93	3
10	400	264	234	299	89	3
11	308	192	207	236	63	3
12	190	202	108	166	51	3
13	140	90	83	104	31	3
14	190	315	169	225	79	3
Minute Volume (mL/Minute)						
1	13721	16381	14059	14720	1448	3
2	15835	21254	21384	19491	3167	3
3	4028	16052	14522	11534	6545	3
4	13431	6643	9643	9906	3402	3
5	17777	20628	15370	17925	2632	3
6	15704	15812	17624	16380	1079	3
7	15438	13438	10605	13160	2428	3
8	10852	17321	11436	13203	3578	3
9	10368	7587	18813	12256	5846	3
10	11940	13849	31227	19005	10627	3
11	11734	12083	10119	11312	1048	3
12	16062	11429	17546	15012	3191	3
13	4572	7636	15600	9269	5692	3
14	18390	11898	14651	14980	3258	3

Table C-10 – Individual Animal Respiratory Function Data
(Respiratory Rate, Tidal Volume and Minute Volume)

GROUP 3 (APN01; 0.019 mg/L) FEMALES

Study Day	Individual Animal Data			Daily Summary Data		
	3CVG	3FKE	3ZRE	Mean	S.D.	SEM
Respiratory Rate (Breaths/Minute)						
1	71.4	—[a]	38.0	54.7	23.6	2
2	197.1	43.1	39.3	93.1	90.0	3
3	298.1	34.9	43.4	125.5	149.6	3
4	118.4	52.6	47.6	72.8	39.5	3
5	42.1	92.0	62.2	65.4	25.1	3
6	68.9	48.4	55.8	57.7	10.4	3
7	303.7	57.3	72.8	144.6	138.0	3
8	246.3	43.0	75.8	121.7	109.2	3
9	287.5	54.6	67.2	136.4	131.0	3
10	256.0	33.5	75.4	121.6	118.3	3
11	275.2	40.6	66.6	127.5	128.6	3
12	275.9	29.6	82.6	129.3	129.6	3
13	49.6	65.8	61.0	58.8	8.3	3
14	39.4	20.8	60.1	40.1	19.7	3
Tidal Volume (mL/Breath)						
1	67	—[a]	198	133	93	2
2	38	211	339	196	151	3
3	17	217	208	147	113	3
4	121	142	246	170	67	3
5	290	97	214	200	97	3
6	171	154	185	170	15	3
7	19	325	218	188	155	3
8	33	190	39	87	89	3
9	73	271	311	218	128	3
10	55	203	82	113	79	3
11	54	64	158	92	57	3
12	55	156	182	131	67	3
13	236	136	42	138	97	3
14	314	194	42	183	137	3
Minute Volume (mL/Minute)						
1	4798	—[a]	7527	6163	1930	2
2	7417	9071	13324	9937	3047	3
3	5084	7575	9014	7224	1989	3
4	13903	7461	11691	11019	3273	3
5	12200	8951	13309	11487	2265	3
6	11766	7275	10309	9783	2291	3
7	5881	18602	15901	13461	6703	3
8	8043	8116	2937	6365	2969	3
9	20873	14804	20916	18864	3517	3
10	13956	6787	6196	8980	4320	3
11	14783	2584	10485	9284	6188	3
12	15125	4556	15025	11569	6073	3
13	11695	8977	2542	7738	4701	3
14	12382	4020	2495	6299	5323	3

[a] Data not available due to unstable signal

Table C-10 – Individual Animal Respiratory Function Data
(Respiratory Rate, Tidal Volume and Minute Volume)

GROUP 4 (APN01; 0.038 mg/L) MALES

Study Day	Individual Animal Data			Daily Summary Data		
	4EAH	4JLH	4YJF	Mean	S.D.	N
Respiratory Rate (Breaths/Minute)						
1	61.3	60.7	26.6	49.5	19.9	3
2	74.3	157.1	46.9	92.8	57.4	3
3	270.5	154.0	67.8	164.1	101.7	3
4	157.0	113.3	68.6	113.0	44.2	3
5	123.0	152.1	59.2	111.4	47.5	3
6	100.7	142.4	70.6	104.6	36.1	3
7	278.4	177.3	242.8	232.8	51.3	3
8	280.2	233.0	257.0	256.7	23.6	3
9	301.4	227.2	257.2	261.9	37.3	3
10	125.6	189.1	55.9	123.5	66.6	3
11	349.3	111.3	53.8	171.5	156.7	3
12	63.0	166.2	72.3	100.5	57.1	3
13	304.7	203.3	47.1	185.1	129.8	3
14	198.0	198.0	170.2	188.7	16.1	3
Tidal Volume (mL/Breath)						
1	221	259	350	277	67	3
2	229	60	300	196	123	3
3	34	78	225	112	100	3
4	103	86	162	117	40	3
5	122	86	228	145	74	3
6	131	76	406	204	177	3
7	26	46	67	46	21	3
8	25	41	188	85	90	3
9	47	40	48	45	4	3
10	31	37	229	99	112	3
11	37	115	134	96	52	3
12	85	58	227	123	90	3
13	41	56	18	38	19	3
14	28	54	20	34	18	3
Minute Volume (mL/Minute)						
1	13527	15722	9309	12852	3259	3
2	16992	9353	14101	13482	3857	3
3	9222	11767	15208	12066	3004	3
4	16128	9651	11099	12293	3400	3
5	14980	13114	13498	13864	985	3
6	13229	10709	28586	17508	9676	3
7	7117	8129	16351	10533	5064	3
8	7068	9587	47374	21343	22579	3
9	14232	9176	12347	11918	2555	3
10	3904	7071	12771	7916	4493	3
11	13006	12851	7224	11027	3295	3
12	5361	9652	16277	10430	5499	3
13	12448	11436	834	8239	6433	3
14	5453	10763	3440	6552	3783	3

Table C-10 – Individual Animal Respiratory Function Data
(Respiratory Rate, Tidal Volume and Minute Volume)

GROUP 4 (APN01; 0.038 mg/L) FEMALES

Study Day	Individual Animal Data			Daily Summary Data		
	4ACE	4CAE	4EVE	Mean	S.D.	N
Respiratory Rate (Breaths/Minute)						
1	157.4	49.3	80.0	95.6	55.7	3
2	63.8	326.3	208.7	199.6	131.4	3
3	68.0	170.3	117.0	118.4	51.2	3
4	110.5	229.4	252.4	197.4	76.2	3
5	96.8	148.9	136.3	127.3	27.2	3
6	250.8	136.0	156.6	181.1	61.2	3
7	235.7	59.1	198.7	164.5	93.1	3
8	175.6	129.5	278.8	194.6	76.4	3
9	62.2	90.1	310.8	154.4	136.2	3
10	188.2	100.4	221.8	170.1	62.7	3
11	41.5	158.5	157.0	119.0	67.1	3
12	88.5	183.2	228.5	166.7	71.4	3
13	76.9	72.6	396.9	182.1	186.0	3
14	62.4	175.5	315.0	184.3	126.5	3
Tidal Volume (mL/Breath)						
1	33	325	132	163	148	3
2	201	30	48	93	94	3
3	262	100	94	152	95	3
4	184	70	56	103	70	3
5	149	86	104	113	33	3
6	20	108	91	73	47	3
7	37	269	57	121	129	3
8	25	138	39	67	61	3
9	201	162	53	139	77	3
10	74	102	21	66	41	3
11	223	25	85	111	101	3
12	221	93	73	129	80	3
13	174	93	82	116	50	3
14	236	98	9	114	114	3
Minute Volume (mL/Minute)						
1	5087	15994	9918	10333	5465	3
2	12806	9736	10034	10859	1693	3
3	17822	16903	10956	15227	3727	3
4	20188	16061	14200	16816	3065	3
5	14414	12743	14175	13777	903	3
6	5022	14724	14297	11347	5482	3
7	8762	15915	11312	11996	3626	3
8	4478	17865	10916	11086	6695	3
9	12502	14593	16429	14508	1965	3
10	13857	10282	4668	9603	4632	3
11	9237	4029	13387	8885	4689	3
12	19397	16997	16699	17698	1479	3
13	13366	6767	32371	17501	13293	3
14	14726	17256	2892	11625	7667	3

Table C-10 – Individual Animal Respiratory Function Data
(Respiratory Rate, Tidal Volume and Minute Volume)

GROUP 5 (APN01; 0.075 mg/L) MALES

Study Day	Individual Animal Data			Daily Summary Data		
	5GPH	5ZOF	5ZTF	Mean	S.D.	N
Respiratory Rate (Breaths/Minute)						
1	87.3	43.3	70.5	67.0	22.2	3
2	73.2	54.6	62.5	63.4	9.3	3
3	43.0	74.7	57.8	58.5	15.8	3
4	51.9	40.9	79.4	57.4	19.8	3
5	47.0	40.1	54.4	47.2	7.2	3
6	52.5	49.3	53.0	51.6	2.0	3
7	44.2	40.5	75.1	53.3	19.0	3
8	56.5	70.7	57.8	61.7	7.9	3
9	46.4	72.9	77.5	65.6	16.8	3
10	47.7	49.3	73.4	56.8	14.4	3
11	57.7	92.6	74.3	74.9	17.5	3
12	167.6	43.9	58.1	89.9	67.7	3
13	49.0	25.6	63.5	46.0	19.1	3
14	48.7	66.7	61.7	59.0	9.3	3
Tidal Volume (mL/Breath)						
1	114	256	44	138	108	3
2	59	27	281	122	139	3
3	240	27	96	121	109	3
4	262	155	125	181	72	3
5	269	308	187	255	62	3
6	307	171	200	226	72	3
7	193	96	163	151	50	3
8	256	212	309	259	48	3
9	326	72	206	201	127	3
10	152	325	143	207	103	3
11	165	88	115	123	39	3
12	60	292	237	197	121	3
13	229	221	218	222	6	3
14	326	95	147	189	121	3
Minute Volume (mL/Minute)						
1	9997	11088	3104	8063	4329	3
2	3838	1399	17567	7601	8717	3
3	10224	1998	5568	5930	4125	3
4	13594	6287	9917	9933	3654	3
5	12644	12361	10134	11713	1375	3
6	16117	8446	10582	11715	3959	3
7	8473	3889	12250	8204	4187	3
8	14403	15016	17873	15764	1852	3
9	14635	5218	15991	11948	5868	3
10	7226	16023	10480	11243	4448	3
11	9484	8179	8313	8659	718	3
12	10144	12843	13810	12266	1900	3
13	11180	5641	13818	10213	4174	3
14	15867	6352	9057	10425	4903	3

Table C-10 – Individual Animal Respiratory Function Data
(Respiratory Rate, Tidal Volume and Minute Volume)

GROUP 5 (APN01; 0.075 mg/L) FEMALES

Study Day	Individual Animal Data			Daily Summary Data		
	5AIE	5CGE	5CSG	Mean	S.D.	N
Respiratory Rate (Breaths/Minute)						
1	51.1	53.0	62.1	55.4	5.9	3
2	101.6	92.4	64.1	86.0	19.5	3
3	67.6	36.1	111.0	71.6	37.6	3
4	69.0	43.9	214.9	109.3	92.4	3
5	73.9	29.6	96.4	66.6	34.0	3
6	67.9	39.9	99.8	69.2	30.0	3
7	121.3	32.7	134.4	96.2	55.3	3
8	64.7	76.2	117.6	86.2	27.8	3
9	79.7	60.3	135.9	92.0	39.3	3
10	67.6	50.7	53.4	57.2	9.1	3
11	58.0	67.5	189.4	105.0	73.3	3
12	77.6	65.1	130.8	91.2	34.9	3
13	68.4	86.1	56.2	70.3	15.0	3
14	64.1	40.3	68.1	57.5	15.0	3
Tidal Volume (mL/Breath)						
1	271	79	269	206	110	3
2	147	38	138	108	60	3
3	295	357	137	263	113	3
4	193	418	110	240	159	3
5	211	168	119	166	46	3
6	206	90	104	134	63	3
7	106	313	86	169	126	3
8	283	95	91	156	110	3
9	266	200	138	201	64	3
10	181	246	111	179	68	3
11	318	171	147	212	93	3
12	262	283	190	245	49	3
13	138	72	178	129	54	3
14	195	244	60	166	95	3
Minute Volume (mL/Minute)						
1	13798	4135	14720	10884	5864	3
2	14913	2802	8723	8813	6056	3
3	19944	12859	15207	16003	3609	3
4	13300	18335	22052	17895	4392	3
5	15625	4953	11437	10672	5377	3
6	14001	3605	10231	9279	5263	3
7	12904	10241	11584	11576	1332	3
8	18292	7219	10624	12045	5672	3
9	21199	12068	18689	17319	4717	3
10	12226	12497	5902	10209	3732	3
11	18494	11536	27841	19290	8181	3
12	20332	18441	24895	21222	3318	3
13	9447	6201	10019	8556	2060	3
14	12465	9815	4088	8790	4281	3

Table C-11 – Individual Animal Peripheral Oxygen Saturation Data
(%)

MALES (PRE-TEST)

Dose Group	Animal Number	day -4
G 1 / M Saline	1EZF	95
	1FUH	91
	1UYF	81
	Mean	89
	S.D.	7.2
	N	3
G 2 / M Vehicle	2AGF	79
	2BSF	93
	2FRH	92
	Mean	88
	S.D.	7.8
	N	3
G 3 / M Low 0.019 mg/L	3DWH	90
	3THF	98
	3TYF	87
	Mean	92
	S.D.	5.7
	N	3
G 4 / M Mid 0.038 mg/L	4EAH	87
	4JLH	87
	4YJF	89
	Mean	88
	S.D.	1.2
	N	3
G 5 / M High 0.075 mg/L	5GPH	75
	5ZOF	96
	5ZTF	96
	Mean	89
	S.D.	12.1
	N	3

Table C-11 – Individual Animal Peripheral Oxygen Saturation Data
(%)

MALES (TREATMENT PERIOD)

Dose Group	Animal Number	day 1	day 4	day 7	day 10	day 14
G 1 / M Saline	1EZF	97	81	98	87	73
	1FUH	85	85	92	88	96
	1UYF	79	93	95	90	87
	Mean	87	86	95	88	85
	S.D.	9.2	6.1	3.0	1.5	11.6
	N	3	3	3	3	3
G 2 / M Vehicle	2AGF	77	70	77	80	83
	2BSF	77	79	95	85	86
	2FRH	87	95	100	82	100
	Mean	80	81	91	82	90
	S.D.	5.8	12.7	12.1	2.5	9.1
	N	3	3	3	3	3
G 3 / M Low 0.019 mg/L	3DWH	83	96	99	98	99
	3THF	95	99	94	98	97
	3TYF	99	96	74	93	80
	Mean	92	97	89	96	92
	S.D.	8.3	1.7	13.2	2.9	10.4
	N	3	3	3	3	3
G 4 / M Mid 0.038 mg/L	4EAH	82	94	94	80	93
	4JLH	79	74	95	80	79
	4YJF	98	95	90	97	76
	Mean	86	88	93	86	83
	S.D.	10.2	11.8	2.6	9.8	9.1
	N	3	3	3	3	3
G 5 / M High 0.075 mg/L	5GPH	74	93	76	75	80
	5ZOF	82	81	92	73	76
	5ZTF	92	77	92	77	97
	Mean	83	84	87	75	84
	S.D.	9.0	8.3	9.2	2.0	11.2
	N	3	3	3	3	3

Table C-11 – Individual Animal Peripheral Oxygen Saturation Data

(%)

FEMALES (PRE-TEST)

Dose Group	Animal Number	day -5
G 1 / F Saline	1CBE	96
	1JKG	75
	1JSG	80
	Mean<br>S.D.<br>N	84<br>11.0<br>3
G 2 / F Vehicle	2AJE	94
	2ZQE	94
	2FLE	80
	Mean<br>S.D.<br>N	89<br>8.1<br>3
G 3 / F Low 0.019 mg/L	3CVG	96
	3FKE	81
	3ZRE	98
	Mean<br>S.D.<br>N	92<br>9.3<br>3
G 4 / F Mid 0.038 mg/L	4ACE	84
	4CAE	83
	4EVE	79
	Mean<br>S.D.<br>N	82<br>2.6<br>3
G 5 / F High 0.075 mg/L	5AIE	75
	5CGE	99
	5CSG	74
	Mean<br>S.D.<br>N	83<br>14.2<br>3

Table C-11 – Individual Animal Peripheral Oxygen Saturation Data

(%)

FEMALES (TREATMENT PERIOD)

Dose Group	Animal Number	day 1	day 4	day 7	day 10
G 1 / F Saline	1CBE	97	74	83	75
	1JKG	89	76	82	78
	1JSG	92	94	75	75
	Mean	93	81	80	76
	S.D.	4.0	11.0	4.4	1.7
	N	3	3	3	3
G 2 / F Vehicle	2AJE	71	82	88	94
	2ZQE	80	82	90	97
	2FLE	88	93	93	84
	Mean	80	86	90	92
	S.D.	8.5	6.4	2.5	6.8
	N	3	3	3	3
G 3 / F Low 0.019 mg/L	3CVG	100	91	93	75
	3FKE	97	79	89	91
	3ZRE	97	70	78	91
	Mean	98	80	87	86
	S.D.	1.7	10.5	7.8	9.2
	N	3	3	3	3
G 4 / F Mid 0.038 mg/L	4ACE	91	70	75	99
	4CAE	96	81	99	97
	4EVE	95	95	97	74
	Mean	94	82	90	90
	S.D.	2.6	12.5	13.3	13.9
	N	3	3	3	3
G 5 / F High 0.075 mg/L	5AIE	97	98	98	94
	5CGE	91	95	100	78
	5CSG	76	76	99	76
	Mean	88	90	99	83
	S.D.	10.8	11.9	1.0	9.9
	N	3	3	3	3

NOTE: No data available for females on Day 14.

Table C-12 – Individual Animal Venous Blood Oxygen Saturation Data (mmHg)

MALES (PRE-TEST)

Dose Group	Animal Number	day -4
G 1 / M Saline	1EZF	44.6
	1FUH	47.7
	1UYF	42.5
	Mean	44.9
	S.D.	2.62
	N	3
G 2 / M Vehicle	2AGF	49.3
	2BSF	78.2
	2FRH	41.5
	Mean	56.3
	S.D.	19.33
	N	3
G 3 / M Low 0.019 mg/L	3DWH	40.0
	3THF	58.8
	3TYF	46.9
	Mean	48.6
	S.D.	9.51
	N	3
G 4 / M Mid 0.038 mg/L	4EAH	51.0
	4JLH	46.3
	4YJF	44.6
	Mean	47.3
	S.D.	3.32
	N	3
G 5 / M High 0.075 mg/L	5GPH	53.2
	5ZOF	57.7
	5ZTF	54.5
	Mean	55.1
	S.D.	2.32
	N	3

Table C-12 – Individual Animal Venous Blood Oxygen Saturation Data (mmHg)

MALES (TREATMENT PERIOD)

Dose Group	Animal Number	day 1	day 4	day 7	day 10	day 14
G 1 / M Saline	1EZF	42.2	56.1	59.6	60.9	47.4
	1FUH	54.9	77.4	91.0	80.0	65.9
	1UYF	44.4	53.9	62.8	72.5	46.5
	Mean	47.2	62.5	71.1	71.1	53.3
	S.D.	6.79	12.98	17.28	9.62	10.95
	N	3	3	3	3	3
G 2 / M Vehicle	2AGF	71.4	65.2	58.9	50.5	59.0
	2BSF	44.8	63.3	66.4	49.9	53.8
	2FRH	46.3	65.3	68.9	57.0	65.7
	Mean	54.2	64.6	64.7	52.5	59.5
	S.D.	14.94	1.13	5.20	3.94	5.97
	N	3	3	3	3	3
G 3 / M Low 0.019 mg/L	3DWH	81.2	47.4	46.6	46.7	50.5
	3THF	63.8	44.9	54.4	73.9	49.4
	3TYF	95.1	74.5	42.9	56.6	59.7
	Mean	80.0	55.6	48.0	59.1	53.2
	S.D.	15.68	16.42	5.87	13.77	5.66
	N	3	3	3	3	3
G 4 / M Mid 0.038 mg/L	4EAH	37.5	58.7	76.2	47.9	135.9
	4JLH	54.9	44.6	61.3	68.3	112.6
	4YJF	40.2	44.4	65.4	36.3	147.3
	Mean	44.2	49.2	67.6	50.8	131.9
	S.D.	9.36	8.20	7.70	16.20	17.69
	N	3	3	3	3	3
G 5 / M High 0.075 mg/L	5GPH	50.3	56.7	54.5	59.9	68.2
	5ZOF	41.9	57.3	46.7	62.4	50.3
	5ZTF	49.4	53.7	56.7	71.6	50.5
	Mean	47.2	55.9	52.6	64.6	56.3
	S.D.	4.61	1.93	5.25	6.16	10.28
	N	3	3	3	3	3

Table C-12 – Individual Animal Venous Blood Oxygen Saturation Data (mmHg)

FEMALES (PRE-TEST)

Dose Group	Animal Number	day -5
G 1 / F Saline	1CBE	46.8
	1JKG	49.6
	1JSG	52.5
	Mean	49.6
	S.D.	2.85
	N	3
G 2 / F Vehicle	2AJE	47.0
	2ZQE	49.5
	2FLE	52.8
	Mean	49.8
	S.D.	2.91
	N	3
G 3 / F Low 0.019 mg/L	3CVG	52.0
	3FKE	45.5
	3ZRE	55.2
	Mean	50.9
	S.D.	4.94
	N	3
G 4 / F Mid 0.038 mg/L	4ACE	67.6
	4CAE	44.5
	4EVE	70.2
	Mean	60.8
	S.D.	14.15
	N	3
G 5 / F High 0.075 mg/L	5AIE	42.0
	5CGE	43.3
	5CSG	44.3
	Mean	43.2
	S.D.	1.15
	N	3

Table C-12 – Individual Animal Venous Blood Oxygen Saturation Data (mmHg)

FEMALES (TREATMENT PERIOD)

Dose Group	Animal Number	day 1	day 4	day 7	day 10	day 14
G 1 / F Saline	1CBE	55.2	121.6	138.1	73.5	90.5
	1JKG	51.3	72.8	132.5	55.8	80.4
	1JSG	61.5	100.2	53.0	56.2	82.8
	Mean	56.0	98.2	107.9	61.8	84.6
	S.D.	5.15	24.46	47.60	10.11	5.28
	N	3	3	3	3	3
G 2 / F Vehicle	2AJE	44.0	60.6	105.9	68.5	95.4
	2ZQE	62.0	58.5	60.4	60.4	95.6
	2FLE	137.3	85.4	67.5	50.0	162.6
	Mean	81.1	68.2	77.9	59.6	117.9
	S.D.	49.50	14.96	24.48	9.27	38.74
	N	3	3	3	3	3
G 3 / F Low 0.019 mg/L	3CVG	60.5	69.9	71.1	47.1	64.1
	3FKE	77.5	112.8	100.7	68.8	67.2
	3ZRE	64.8	77.2	95.4	60.1	103.7
	Mean	67.6	86.6	89.1	58.7	78.3
	S.D.	8.84	22.95	15.78	10.92	22.02
	N	3	3	3	3	3
G 4 / F Mid 0.038 mg/L	4ACE	39.6	47.5	38.5	39.6	94.8
	4CAE	54.2	66.8	74.4	50.3	161.2
	4EVE	36.4	48.0	73.4	47.3	153.5
	Mean	43.4	54.1	62.1	45.7	136.5
	S.D.	9.49	11.00	20.44	5.52	36.32
	N	3	3	3	3	3
G 5 / F High 0.075 mg/L	5AIE	65.1	78.9	91.9	96.2	79.4
	5CGE	54.2	59.4	86.3	79.6	71.1
	5CSG	48.6	61.0	59.9	56.0	59.1
	Mean	56.0	66.4	79.4	77.3	69.9
	S.D.	8.39	10.83	17.09	20.20	10.21
	N	3	3	3	3	3

Table C-13 – Individual Animal Blood pH Data

MALES (PRE-TEST)

Dose Group	Animal Number	day -4
G 1 / M Saline	1EZF	7.361
	1FUH	7.409
	1UYF	7.409
	Mean	7.393
	S.D.	0.0277
	N	3
G 2 / M Vehicle	2AGF	7.358
	2BSF	7.364
	2FRH	7.377
	Mean	7.366
	S.D.	0.0097
	N	3
G 3 / M Low 0.019 mg/L	3DWH	7.415
	3THF	7.448
	3TYF	7.374
	Mean	7.412
	S.D.	0.0371
	N	3
G 4 / M Mid 0.038 mg/L	4EAH	7.424
	4JLH	7.329
	4YJF	7.445
	Mean	7.399
	S.D.	0.0618
	N	3
G 5 / M High 0.075 mg/L	5GPH	7.417
	5ZOF	7.422
	5ZTF	7.450
	Mean	7.430
	S.D.	0.0178
	N	3

Table C-13 – Individual Animal Blood pH Data

MALES (TREATMENT PERIOD)

Dose Group	Animal Number	day 1	day 4	day 7	day 10	day 14
G 1 / M Saline	1EZF	7.336	7.378	7.371	7.366	7.368
	1FUH	7.302	7.350	7.363	7.341	7.333
	1UYF	7.372	7.385	7.336	7.387	7.369
	Mean	7.337	7.371	7.357	7.365	7.357
	S.D.	0.0350	0.0185	0.0183	0.0230	0.0205
	N	3	3	3	3	3
G 2 / M Vehicle	2AGF	7.402	7.434	7.399	7.409	7.409
	2BSF	7.347	7.324	7.415	7.409	7.420
	2FRH	7.346	7.365	7.377	7.389	7.463
	Mean	7.365	7.374	7.397	7.402	7.431
	S.D.	0.0320	0.0556	0.0191	0.0115	0.0285
	N	3	3	3	3	3
G 3 / M Low 0.019 mg/L	3DWH	7.299	7.341	7.368	7.293	7.325
	3THF	7.332	7.346	7.347	7.384	7.367
	3TYF	7.324	7.416	7.341	7.361	7.355
	Mean	7.318	7.368	7.352	7.346	7.349
	S.D.	0.0172	0.0419	0.0142	0.0473	0.0216
	N	3	3	3	3	3
G 4 / M Mid 0.038 mg/L	4EAH	7.367	7.377	7.413	7.395	7.340
	4JLH	7.292	7.403	7.401	7.374	7.401
	4YJF	7.344	7.390	7.417	7.379	7.386
	Mean	7.334	7.390	7.410	7.383	7.376
	S.D.	0.0384	0.0130	0.0083	0.0110	0.0318
	N	3	3	3	3	3
G 5 / M High 0.075 mg/L	5GPH	7.362	7.443	7.337	7.404	7.334
	5ZOF	7.342	7.408	7.407	7.395	7.388
	5ZTF	7.349	7.396	7.294	7.342	7.375
	Mean	7.351	7.416	7.346	7.380	7.366
	S.D.	0.0101	0.0244	0.0570	0.0335	0.0282
	N	3	3	3	3	3

Table C-13 – Individual Animal Blood pH Data

FEMALES (PRE-TEST)

Dose Group	Animal Number	day -5
G 1 / F Saline	1CBE	7.414
	1JKG	7.430
	1JSG	7.336
	Mean	7.393
	S.D.	0.0503
	N	3
G 2 / F Vehicle	2AJE	7.397
	2ZQE	7.355
	2FLE	7.353
	Mean	7.368
	S.D.	0.0248
	N	3
G 3 / F Low 0.019 mg/L	3CVG	7.335
	3FKE	7.361
	3ZRE	7.311
	Mean	7.336
	S.D.	0.0250
	N	3
G 4 / F Mid 0.038 mg/L	4ACE	7.406
	4CAE	7.397
	4EVE	7.376
	Mean	7.393
	S.D.	0.0154
	N	3
G 5 / F High 0.075 mg/L	5AIE	7.421
	5CGE	7.380
	5CSG	7.375
	Mean	7.392
	S.D.	0.0252
	N	3

Table C-13 – Individual Animal Blood pH Data

FEMALES (TREATMENT PERIOD)

Dose Group	Animal Number	day 1	day 4	day 7	day 10	day 14
G 1 / F Saline	1CBE	7.394	7.458	7.337	7.357	7.370
	1JKG	7.358	7.356	7.413	7.290	7.264
	1JSG	7.363	7.384	7.263	7.336	7.256
	Mean	7.372	7.399	7.338	7.328	7.297
	S.D.	0.0195	0.0527	0.0750	0.0343	0.0636
	N	3	3	3	3	3
G 2 / F Vehicle	2AJE	7.421	7.354	7.402	7.400	7.239
	2ZQE	7.391	7.370	7.392	7.381	7.272
	2FLE	7.376	7.415	7.343	7.368	7.314
	Mean	7.396	7.380	7.379	7.383	7.275
	S.D.	0.0229	0.0316	0.0316	0.0161	0.0376
	N	3	3	3	3	3
G 3 / F Low 0.019 mg/L	3CVG	7.391	7.440	7.353	7.321	7.307
	3FKE	7.412	7.503	7.334	7.365	7.270
	3ZRE	7.415	7.416	7.332	7.357	7.279
	Mean	7.406	7.453	7.340	7.348	7.285
	S.D.	0.0131	0.0449	0.0116	0.0234	0.0193
	N	3	3	3	3	3
G 4 / F Mid 0.038 mg/L	4ACE	7.442	7.408	7.464	7.375	7.289
	4CAE	7.394	7.415	7.419	7.351	7.266
	4EVE	7.377	7.410	7.411	7.399	7.300
	Mean	7.404	7.411	7.431	7.375	7.285
	S.D.	0.0337	0.0036	0.0286	0.0240	0.0173
	N	3	3	3	3	3
G 5 / F High 0.075 mg/L	5AIE	7.364	7.467	7.416	7.386	7.419
	5CGE	7.364	7.371	7.380	7.385	7.401
	5CSG	7.391	7.394	7.347	7.366	7.339
	Mean	7.373	7.411	7.381	7.379	7.386
	S.D.	0.0156	0.0501	0.0345	0.0113	0.0420
	N	3	3	3	3	3

Table C-14 – Individual Animal Absolute Organ Weight Data

MALES

Dose Group	Animal Number	ADR [g] day 15	BRN [g] day 15	HRT [g] day 15	KIDS [g] day 15	LIV [g] day 15	SPL [g] day 15	TST [g] day 15	THYM [g] day 15	EPI [g] day 15
G 1 / M Saline	1EZF	1.082	81.92	59.03	39.08	231.52	42.183	12.94	9.542	2.674
	1FUH	1.045	71.74	78.42	42.72	294.29	20.038	13.32	5.315	2.375
	1UYF	1.170	80.55	71.18	49.20	282.00	65.979	14.58	7.418	2.646
	Mean	1.099	78.07	69.54	43.67	269.27	42.733	13.61	7.425	2.565
	S.D.	0.0642	5.525	9.798	5.126	33.265	22.9754	0.858	2.1135	0.1651
	N	3	3	3	3	3	3	3	3	3
G 2 / M Vehicle	2AGF	0.884	85.29	68.14	44.70	213.07	47.110	13.32	3.970	3.110
	2BSF	0.894	67.27	69.87	54.18	295.86	82.189	11.61	5.722	2.724
	2FRH	1.303	74.66	76.73	41.56	212.00	49.492	12.23	5.345	2.522
	Mean	1.027	75.74	71.58	46.81	240.31	59.597	12.39	5.012	2.785
	S.D.	0.2391	9.058	4.543	6.570	48.111	19.6015	0.866	0.9222	0.2988
	N	3	3	3	3	3	3	3	3	3
G 3 / M Low 0.019 mg/L	3DWH	0.973	79.14	162.29	48.56	281.22	46.160	16.42	4.092	2.556
	3THF	1.268	71.71	73.33	52.02	259.35	49.213	20.16	3.342	4.087
	3TYF	0.893	73.30	69.35	43.24	278.35	62.479	11.93	5.676	2.534
	Mean	1.045	74.72	101.66	47.94	272.97	52.617	16.17	4.370	3.059
	S.D.	0.1975	3.912	52.548	4.423	11.885	8.6758	4.121	1.1916	0.8903
	N	3	3	3	3	3	3	3	3	3
G 4 / M Mid 0.038 mg/L	4EAH	0.905	82.81	77.23	43.94	281.08	34.249	9.97	3.992	1.806
	4JLH	0.940	79.82	61.23	42.83	255.69	55.782	7.72	10.510	1.489
	4YJF	1.068	76.68	75.96	41.65	253.40	58.250	13.60	6.975	2.410
	Mean	0.971	79.77	71.47	42.81	263.39	49.427	10.43	7.159	1.902
	S.D.	0.0858	3.065	8.894	1.145	15.363	13.2023	2.967	3.2629	0.4679
	N	3	3	3	3	3	3	3	3	3
G 5 / M High 0.075 mg/L	5GPH	1.036	102.03	93.19	41.27	242.56	55.601	13.35	8.156	2.483
	5ZOF	0.788	65.14	69.93	43.52	255.05	35.384	10.96	5.450	2.407
	5ZTF	0.789	64.92	70.42	41.61	216.62	60.686	8.93	9.076	2.631
	Mean	0.871	77.36	77.85	42.13	238.08	50.557	11.08	7.561	2.507
	S.D.	0.1429	21.362	13.290	1.213	19.603	13.3839	2.212	1.8849	0.1139
	N	3	3	3	3	3	3	3	3	3

Table C-14 – Individual Animal Absolute Organ Weight Data

FEMALES

Dose Group	Animal Number	ADR [g] day 15	BRN [g] day 15	HRT [g] day 15	KIDS [g] day 15	LIV [g] day 15	OVA [g] day 15	SPL [g] day 15	THYM [g] day 15	UTER [g] day 15
G 1 / F Saline	1CBE	0.975	77.25	67.11	34.88	186.68	0.902	48.397	5.116	1.06
	1JKG	1.328	77.11	87.49	43.88	267.44	0.948	81.463	6.579	3.19
	1JSG	0.960	73.80	55.35	34.56	213.50	0.461	22.876	11.117	0.94
	Mean	1.088	76.05	69.98	37.77	222.54	0.770	50.912	7.604	1.73
	S.D.	0.2083	1.953	16.262	5.291	41.132	0.2689	29.3744	3.1291	1.266
	N	3	3	3	3	3	3	3	3	3
G 2 / F Vehicle	2AJE	1.059	67.09	61.86	33.54	182.92	0.764	27.383	4.403	2.04
	2ZQE	0.839	60.20	67.03	30.03	205.14	1.701	53.841	6.234	12.74
	2FLE	1.202	74.56	64.86	30.52	215.29	0.617	54.505	6.278	1.87
	Mean	1.033	67.28	64.58	31.36	201.12	1.027	45.243	5.638	5.55
	S.D.	0.1829	7.182	2.596	1.901	16.556	0.5880	15.4708	1.0701	6.227
	N	3	3	3	3	3	3	3	3	3
G 3 / F Low 0.019 mg/L	3CVG	0.878	77.01	65.49	42.67	227.37	0.841	49.982	5.416	5.19
	3FKE	0.909	72.29	55.31	30.91	198.51	0.683	28.480	4.725	2.28
	3ZRE	0.951	65.21	60.86	33.68	189.25	0.637	51.203	3.288	0.87
	Mean	0.913	71.50	60.55	35.75	205.04	0.720	43.222	4.476	2.78
	S.D.	0.0366	5.939	5.097	6.148	19.882	0.1070	12.7812	1.0856	2.203
	N	3	3	3	3	3	3	3	3	3
G 4 / F Mid 0.038 mg/L	4ACE	0.959	74.18	61.90	38.91	218.81	0.672	46.192	6.226	2.42
	4CAE	1.029	68.56	65.89	33.63	174.53	0.667	27.400	4.074	1.97
	4EVE	1.099	69.29	70.03	34.15	215.47	0.727	35.972	8.746	3.08
	Mean	1.029	70.68	65.94	35.56	202.94	0.689	36.521	6.349	2.49
	S.D.	0.0700	3.056	4.065	2.910	24.658	0.0333	9.4080	2.3384	0.558
	N	3	3	3	3	3	3	3	3	3
G 5 / F High 0.075 mg/L	5AIE	0.871	75.78	59.58	31.64	169.06	0.645	62.712	5.171	1.44
	5CGE	0.900	73.01	61.14	30.57	185.07	0.658	24.073	5.474	2.66
	5CSG	1.084	71.17	71.93	44.26	250.63	0.743	54.284	3.405	2.92
	Mean	0.952	73.32	64.22	35.49	201.59	0.682	47.023	4.683	2.34
	S.D.	0.1155	2.321	6.725	7.614	43.221	0.0532	20.3171	1.1174	0.790
	N	3	3	3	3	3	3	3	3	3

Table C-15 – Individual Animal Relative Organ Weight (Organ-to-Body Weight Ratio) Data

MALES

Dose Group	Animal Number	FBW [kg] day 15	ADR [%] day 15	BRN [%] day 15	HRT [%] day 15	KIDS [%] day 15	LIV [%] day 15	SPL [%] day 15	TST [%] day 15	THYM [%] day 15	EPI [%] day 15
G 1 / M Saline	1EZF	8.06	0.013	1.02	0.73	0.48	2.87	0.523	0.16	0.118	0.033
	1FUH	10.00	0.010	0.72	0.78	0.43	2.94	0.200	0.13	0.053	0.024
	1UYF	9.04	0.013	0.89	0.79	0.54	3.12	0.730	0.16	0.082	0.029
	Mean	9.03	0.012	0.87	0.77	0.49	2.98	0.485	0.15	0.085	0.029
	S.D.	0.970	0.0016	0.150	0.031	0.059	0.127	0.2669	0.016	0.0327	0.0047
	N	3	3	3	3	3	3	3	3	3	3
G 2 / M Vehicle	2AGF	8.26	0.011	1.03	0.82	0.54	2.58	0.570	0.16	0.048	0.038
	2BSF	8.76	0.010	0.77	0.80	0.62	3.38	0.938	0.13	0.065	0.031
	2FRH	9.42	0.014	0.79	0.81	0.44	2.25	0.525	0.13	0.057	0.027
	Mean	8.81	0.012	0.86	0.81	0.53	2.74	0.678	0.14	0.057	0.032
	S.D.	0.582	0.0020	0.146	0.014	0.089	0.579	0.2265	0.017	0.0086	0.0055
	N	3	3	3	3	3	3	3	3	3	3
G 3 / M Low 0.019 mg/L	3DWH	8.94	0.011	0.89	1.82	0.54	3.15	0.516	0.18	0.046	0.029
	3THF	8.58	0.015	0.84	0.85	0.61	3.02	0.574	0.23	0.039	0.048
	3TYF	8.60	0.010	0.85	0.81	0.50	3.24	0.726	0.14	0.066	0.029
	Mean	8.71	0.012	0.86	1.16	0.55	3.13	0.605	0.19	0.050	0.035
	S.D.	0.202	0.0024	0.025	0.569	0.052	0.107	0.1087	0.048	0.0141	0.0108
	N	3	3	3	3	3	3	3	3	3	3
G 4 / M Mid 0.038 mg/L	4EAH	8.20	0.011	1.01	0.94	0.54	3.43	0.418	0.12	0.049	0.022
	4JLH	8.64	0.011	0.92	0.71	0.50	2.96	0.646	0.09	0.122	0.017
	4YJF	9.18	0.012	0.84	0.83	0.45	2.76	0.635	0.15	0.076	0.026
	Mean	8.67	0.011	0.92	0.83	0.50	3.05	0.566	0.12	0.082	0.022
	S.D.	0.491	0.0004	0.087	0.117	0.041	0.343	0.1285	0.029	0.0369	0.0045
	N	3	3	3	3	3	3	3	3	3	3
G 5 / M High 0.075 mg/L	5GPH	8.86	0.012	1.15	1.05	0.47	2.74	0.628	0.15	0.092	0.028
	5ZOF	8.92	0.009	0.73	0.78	0.49	2.86	0.397	0.12	0.061	0.027
	5ZTF	8.28	0.010	0.78	0.85	0.50	2.62	0.733	0.11	0.110	0.032
	Mean	8.69	0.010	0.89	0.90	0.49	2.74	0.586	0.13	0.088	0.029
	S.D.	0.353	0.0015	0.229	0.139	0.018	0.122	0.1720	0.022	0.0246	0.0025
	N	3	3	3	3	3	3	3	3	3	3

NOTE: Organ-to-body weight ratio = [absolute organ weight (g) ÷ fasted body weight (g)] × 100

Table C-15 – Individual Animal Relative Organ Weight (Organ-to-Body Weight Ratio) Data

FEMALES

Dose Group	Animal Number	FBW [kg] day 15	ADR [%] day 15	BRN [%] day 15	HRT [%] day 15	KIDS [%] day 15	LIV [%] day 15	OVA [%] day 15	SPL [%] day 15	THYM [%] day 15	UTER [%] day 15
G 1 / F Saline	1CBE	6.22	0.016	1.24	1.08	0.56	3.00	0.015	0.778	0.082	0.02
	1JKG	8.86	0.015	0.87	0.99	0.50	3.02	0.011	0.919	0.074	0.04
	1JSG	7.00	0.014	1.05	0.79	0.49	3.05	0.007	0.327	0.159	0.01
	Mean	7.36	0.015	1.06	0.95	0.52	3.02	0.011	0.675	0.105	0.02
	S.D.	1.356	0.0010	0.186	0.147	0.038	0.025	0.0040	0.3095	0.0467	0.012
	N	3	3	3	3	3	3	3	3	3	3
G 2 / F Vehicle	2AJE	6.74	0.016	1.00	0.92	0.50	2.71	0.011	0.406	0.065	0.03
	2ZQE	7.56	0.011	0.80	0.89	0.40	2.71	0.022	0.712	0.082	0.17
	2FLE	6.66	0.018	1.12	0.97	0.46	3.23	0.009	0.818	0.094	0.03
	Mean	6.99	0.015	0.97	0.93	0.45	2.89	0.014	0.646	0.081	0.08
	S.D.	0.498	0.0035	0.163	0.044	0.051	0.300	0.0071	0.2140	0.0146	0.080
	N	3	3	3	3	3	3	3	3	3	3
G 3 / F Low 0.019 mg/L	3CVG	7.54	0.012	1.02	0.87	0.57	3.02	0.011	0.663	0.072	0.07
	3FKE	6.14	0.015	1.18	0.90	0.50	3.23	0.011	0.464	0.077	0.04
	3ZRE	6.40	0.015	1.02	0.95	0.53	2.96	0.010	0.800	0.051	0.01
	Mean	6.69	0.014	1.07	0.91	0.53	3.07	0.011	0.642	0.067	0.04
	S.D.	0.745	0.0018	0.091	0.042	0.032	0.145	0.0007	0.1690	0.0135	0.028
	N	3	3	3	3	3	3	3	3	3	3
G 4 / F Mid 0.038 mg/L	4ACE	7.24	0.013	1.02	0.85	0.54	3.02	0.009	0.638	0.086	0.03
	4CAE	5.98	0.017	1.15	1.10	0.56	2.92	0.011	0.458	0.068	0.03
	4EVE	6.84	0.016	1.01	1.02	0.50	3.15	0.011	0.526	0.128	0.05
	Mean	6.69	0.016	1.06	0.99	0.53	3.03	0.010	0.541	0.094	0.04
	S.D.	0.644	0.0020	0.074	0.126	0.032	0.116	0.0010	0.0908	0.0307	0.007
	N	3	3	3	3	3	3	3	3	3	3
G 5 / F High 0.075 mg/L	5AIE	7.02	0.012	1.08	0.85	0.45	2.41	0.009	0.893	0.074	0.02
	5CGE	6.46	0.014	1.13	0.95	0.47	2.86	0.010	0.373	0.085	0.04
	5CSG	8.30	0.013	0.86	0.87	0.53	3.02	0.009	0.654	0.041	0.04
	Mean	7.26	0.013	1.02	0.89	0.49	2.76	0.009	0.640	0.066	0.03
	S.D.	0.943	0.0008	0.145	0.052	0.043	0.318	0.0007	0.2606	0.0227	0.011
	N	3	3	3	3	3	3	3	3	3	3

NOTE: Organ-to-body weight ratio = [absolute organ weight (g) ÷ fasted body weight (g)] × 100

***Phase*: Ophthalmic Examinations**	
***Phase Test Site*:**	***Study Director*:**
IIT Research Institute (IITRI)<br>10 West 35th Street<br>Chicago, IL 60616	Jeffrey W. Richig, D.V.M.

Appendix D – Ophthalmic Examination Report

Ophthalmic Examination Report
IITRI Study No. 285700300102

Ophthalmic examinations were performed on all 30 beagle dogs (15 males and 15 females) at pretest and during Week 2. Indirect funduscopic ophthalmic examinations were performed using SOP techniques. All animals were within normal limits for the breed, age, sex and housing conditions at pretest and Week 2, with the exception of dog 2BSF which had a cherry eye O.D.at pretest and Week 2.

Cherry eye is a prolapse of the tear gland of the third eyelid that can occur in genetically predisposed young dogs usually under two years of age.

O.D. = Right eye

Jeffrey W. Richig 2021-03-04
Jeffrey W. Richig, DVM Date
Study Director
Toxicology Division

***Phase*: Analysis of ECG Tracings**	
***Phase Test Site*:**	***Study Director*:**
IIT Research Institute (IITRI)<br>10 West 35th Street<br>Chicago, IL 60616	Jeffrey W. Richig, D.V.M.

Appendix E – Electrocardiographic Evaluation Report

Electrocardiography Evaluation Report
IITRI Study No. 285700300102

Electrocardiographic examinations were performed on all 30 (15 male and 15 female) beagle dogs at pretest and within approximately 2 hours after the first daily exposure during Week 2. Electrocardiographic examinations were performed utilizing a Model Avante True Electrocardiograph 12, DRE Medical Equipment, Louisville, KY. The evaluations included heart rate and rhythm, and duration of the PR interval, QRS complex, and QT interval. All dogs were electrocardiographically within normal limits with the exception of the following:

Dog 3DWH had P-pulmonale at Week 2.
Dog 5ZOF had occasional second-degree atrioventricular block (Mobitz Type II) at Week 2.
Dog 5CGE had tall R waves at Week 2.

P-pulmonale, noted in one Group 3 animal, usually indicates possible right atrial enlargement, which can only be confirmed by additional diagnostics (i.e. chest x-ray, echocardiography) or at necropsy. However, there was no dose response relationship evident and therefore it was not considered treatment-related. Occasional second-degree atrioventricular block (Mobitz Type II), noted in one Group 5 animal, is a normal variant. It is due to increased vagal tone in beagle dogs. Tall R waves, noted in one Group 5 animal, usually indicates possible left ventricular enlargement, which can only be confirmed by additional diagnostics (i.e. chest x-ray, echocardiography) or at necropsy. Therefore, the relationship of this finding to treatment is inconclusive.

Jeffrey W. Richig 2021-03-04
Jeffrey W. Richig, DVM Date
Study Director
Toxicology Division

***Phase*: Analysis of Clinical Pathology Parameters**	
***Phase Test Site*:**	***Study Director*:**
IIT Research Institute (IITRI)<br>10 West 35th Street<br>Chicago, IL 60616	Jeffrey W. Richig, D.V.M.

Appendix F – Clinical Pathology Report

TWO-WEEK AEROSOL TOXICITY STUDY OF APN01 IN DOGS

Appendix F – Clinical Pathology Report

TABLE OF CONTENTS

Page

SIGNATURE PAGE F-2

I. INTRODUCTION F-3

II. METHODS F-3

III. RESULTS F-5

IV. TABLES....F-7

Table F-1a. Summary of Clinical Chemistry Data – Pre-Test (vs Group 1) F-8
Table F-1b. Summary of Clinical Chemistry Data – Pre-Test (vs. Group 2) F-12
Table F-1c. Summary of Clinical Chemistry Data – Day 15 (vs. Group 1) F-16
Table F-1d. Summary of Clinical Chemistry Data – Day 15 (vs. Group 2) F-20

Table F-2a. Summary of Hematology Data – Pre-Test (vs. Group 1)....F-24
Table F-2b. Summary of Hematology Data – Pre-Test (vs. Group 2)....F-28
Table F-2c. Summary of Hematology Data – Day 15 (vs. Group 1) F-32
Table F-2d. Summary of Hematology Data – Day 15 (vs. Group 2) F-36

Table F-3a. Summary of Coagulation Data – Pre-Test (vs. Group 1)....F-40
Table F-3b. Summary of Coagulation Data – Pre-Test (vs. Group 2)....F-41
Table F-3c. Summary of Coagulation Data – Day 15 (vs. Group 1)....F-42
Table F-3d. Summary of Coagulation Data – Day 15 (vs. Group 2)....F-43

Table F-4a. Summary of Select Urinalysis Data – Pre-Test (vs. Group 1) (Refractive Index, SG, pH, Volume) F-44
Table F-4b. Summary of Select Urinalysis Data – Pre-Test (vs. Group 2) (Refractive Index, SG, pH, Volume) F-45
Table F-4c. Summary of Select Urinalysis Data – Day 15 (vs. Group 1) (Refractive Index, SG, pH, Volume) F-46
Table F-4d. Summary of Select Urinalysis Data – Day 15 (vs. Group 2) (Refractive Index, SG, pH, Volume) F-47

Table F-5. Individual Animal Clinical Chemistry Data F-48

Table F-6. Individual Animal Hematology Data....F-60

Table F-7. Individual Animal Coagulation Data....F-76

Table F-8. Individual Animal Urinalysis Data F-80

SIGNATURE PAGE

Report Approval:

Jeffrey W. Richig

Jeffrey W. Richig, D.V.M.
Study Director

2021-04-26
Date

I. INTRODUCTION

This contributing scientist report describes the clinical pathology parameter analysis portion of IITRI Project Number 285700300102. The objective of the study was to provide data to support a comprehensive evaluation of the toxicity of twice daily aerosol administration of APN01 to dogs for fourteen consecutive days. The study design is summarized below:

Group	Number of Dogs (M + F)	Agent	Number and Duration of Daily Exposures	Number of Exposure Days	Target APN01 Concentration in Test Atmosphere (mg/L)
1	3 + 3	Saline (Control)	2 x 60 minutes	14	0
2	3 + 3	Vehicle (Control)	2 x 60 minutes	14	0
3	3 + 3	APN01 – Low	2 x 60 minutes	14	0.019
4	3 + 3	APN01 – Mid	2 x 60 minutes	14	0.038
5	3 + 3	APN01 - High	2 x 60 minutes	14	0.075

Clinical pathology abbreviations and units of measure are listed in a separate appendix (Appendix J), along with a urinalysis key.

II. METHODS

A. Sample Collection: Blood samples for clinical chemistry, hematology and coagulation determinations were collected from the jugular or cephalic vein of each dog during pre-test and prior to terminal necropsy on Study Day 15. Dogs were fasted overnight prior to clinical pathology blood collections.

Urine samples for urinalysis determinations were collected from all dogs during pre-test (on Study Day 1 prior to the first exposure) and on Study Day 15. For pre-test urine collections, dogs were placed in cages overnight, and urine was collected from pans below the cages. For the Study Day 15 urine collections, urine samples were collected directly from the urinary bladder at necropsy.

a. Clinical Chemistry: Clinical chemistry blood samples were collected into tubes, allowed to clot, and centrifuged to obtain serum. The parameters listed below were

evaluated using a Beckman Coulter AU480 Clinical System (Beckman Coulter, Inc.; Brea, CA).

Alanine aminotransferase	Creatinine
Albumin (A)	Creatine kinase
A/G ratio (calculated)	Globulin (G; calculated)
Alkaline phosphatase	Glucose
Aspartate aminotransferase	Phosphorus (inorganic)
Bilirubin (total)	Potassium
Blood urea nitrogen	Protein (total)
Calcium	Sodium
Chloride	Triglycerides
Cholesterol	Urea nitrogen/creatinine ratio (calculated)

b. Hematology: Hematology blood samples (including samples collected for platelet levels) were collected into tubes containing K_2EDTA as the anticoagulant. The parameters listed below were evaluated using an ADVIA 120 Hematology System Analyzer (Siemens Healthcare Diagnostics; Tarrytown, NY).

Differential white blood cell count (absolute and relative)
Erythrocyte count
Erythrocyte morphology
Hematocrit
Hemoglobin
Mean corpuscular hemoglobin
Mean corpuscular hemoglobin concentration
Mean corpuscular volume
Platelet count
Reticulocyte count (absolute and relative)
Total white blood cell count

c. Coagulation: Coagulation blood samples were collected into tubes containing sodium citrate as the anticoagulant. The parameters listed below were evaluated using an STA Compact CT Coagulation Analyzer (Diagnostica Stago; Parsippany, NJ).

Activated partial thromboplastin time
Fibrinogen
Prothrombin time

d. Urinalysis: An analyst assessed the appearance (color and clarity) and volume of each sample and performed a microscopic examination of the sediment (cytology). Refractive index was measured with a refractometer, and a species-specific urine

solids table was used to convert the refractive index to specific gravity. The parameters listed below were evaluated using a cobas u 411 Urine Analyzer (Roche Diagnostics; Indianapolis, IN).

Bilirubin	Occult blood
Glucose	pH
Ketones	Protein
Leukocytes	Urobilinogen
Nitrite	

B. Data Management and Statistics: Clinical pathology data were collected using the ToxData® toxicology data management system (version 3.0; PDS Pathology Data Systems Ltd.; Basel, Switzerland). Descriptive statistics (mean and standard deviation) for clinical chemistry, hematology (excluding erythrocyte morphology), coagulation and select urinalysis parameters [volume, pH, refractive index (RI), specific gravity (SG)] were calculated and analyzed for statistical significance using the ToxData® system.

For all analyses, if the data set was normally distributed and of equal variance, statistical comparisons were conducted using a one-way analysis of variance (ANOVA), with post hoc comparisons made (if necessary) using Dunnett's test. If normality and/or equal variance failed for a data set, statistical comparisons were conducted using nonparametric Kruskal–Wallis ANOVA, with post hoc comparisons made (if necessary) using Dunn's test. A minimum significance level of $p < 0.05$ was used for the statistical comparisons in this study.

III. RESULTS

Clinical chemistry, hematology, coagulation and select urinalysis data are summarized in Tables F-1a/b/c/d, F-2a/b/c/d, F-3a/b/c/d, and F-4a/b/c/d respectively. Summary pre-test data with analysis for statistical significance against Group 1 (Saline Control) are presented in "a" tables; summary pre-test data with analysis for statistical significance against Group 2 (Vehicle Control) are presented in "b" tables; summary Day 15 data with analysis for statistical significance against Group 1 are presented in "c" tables; and summary Day 15 data with analysis for statistical significance against Group 2 are presented in "d" tables. Individual animal data for all parameters are presented in Table F-5 (chemistry), Table F-6 (hematology), Table F-7 (coagulation) and Table F-8 (urinalysis).

No statistically significant differences in comparison to Group 1 (Saline Control) were seen in any statistically analyzed clinical chemistry, hematology, coagulation or urinalysis parameter at Day 15. A statistically significant decrease in cholesterol compared to Group 2 (Vehicle Control) was seen in the Group 3 (0.019 mg/L) females at Day 15. The difference in cholesterol was not considered test article-related due to the lack of a dose-response relationship and only being seen in one sex. No other statistically significant differences in comparison to Group 2 were seen in any statistically analyzed clinical chemistry, hematology, coagulation or urinalysis parameter at Day 15. In addition, a visual examination of the individual animal erythrocyte morphology data and the individual animal urinalysis data (qualitative/scale data sets not conducive to statistical analysis) did not reveal any toxicologically significant differences between the test article-treated groups and Group 1 or Group 2 at Day 15.

IV. TABLES

Table F-1a – Summary of Clinical Chemistry Data – Pre-Test (vs. Group 1)

MALE

		G 1 / M Saline	G 2 / M Vehicle	G 3 / M Low 0.019 mg/L	G 4 / M Mid 0.038 mg/L	G 5 / M High 0.075 mg/L
NA	Mean	131 k	130	140	137	144
[mmol/L]	S.D.	15.9	22.8	6.1	14.6	6.0
day -4	N	3	3	3	3	3
	P-Value	@0.7371				
K	Mean	4.0 a	4.2	4.1	4.1	4.2
[mmol/L]	S.D.	0.20	0.67	0.21	0.15	0.44
day -4	N	3	3	3	3	3
	P-Value	@0.9656				
CL	Mean	100 a	101	107	102	107
[mmol/L]	S.D.	12.2	15.4	5.3	9.0	6.6
day -4	N	3	3	3	3	3
	P-Value	@0.8565				
CALC	Mean	10.8 a	10.9	10.6	11.0	10.7
[mg/dL]	S.D.	0.85	0.25	0.26	0.46	0.38
day -4	N	3	3	3	3	3
	P-Value	@0.8622				
CRE	Mean	0.76 a	0.78	0.70	0.74	0.81
[mg/dL]	S.D.	0.095	0.121	0.032	0.042	0.026
day -4	N	3	3	3	3	3
	P-Value	@0.4993				
PO4	Mean	7.4 k	7.6	6.5	6.8	6.8
[mg/dL]	S.D.	0.12	0.55	0.46	1.08	0.45
day -4	N	3	3	3	3	3
	P-Value	@0.1621				
TP	Mean	5.8 k	6.0	6.1	6.1	6.1
[g/dL]	S.D.	0.67	0.40	0.17	0.36	0.40
day -4	N	3	3	3	3	3
	P-Value	@0.9120				
ALB	Mean	3.1 a	3.2	3.1	3.3	3.1
[g/dL]	S.D.	0.21	0.15	0.21	0.15	0.26
day -4	N	3	3	3	3	3
	P-Value	@0.4333				
GLOB	Mean	2.8 k	2.8	3.0	2.8	3.0
[g/dL]	S.D.	0.47	0.38	0.15	0.25	0.40
day -4	N	3	3	3	3	3
	P-Value	@0.6518				
A/G	Mean	1.1 a	1.2	1.0	1.2	1.0
[-]	S.D.	0.13	0.16	0.11	0.10	0.18
day -4	N	3	3	3	3	3
	P-Value	@0.3725				

k=KRUSKAL-WALLIS; a=ANOVA

Table F-1a – Summary of Clinical Chemistry Data – Pre-Test (vs. Group 1)

MALE

		G 1 / M Saline		G 2 / M Vehicle	G 3 / M Low 0.019 mg/L	G 4 / M Mid 0.038 mg/L	G 5 / M High 0.075 mg/L
BUN	Mean	12	a	15	14	12	14
[mg/dL]	S.D.	3.0		6.4	1.5	1.0	1.5
day -4	N	3		3	3	3	3
	P-Value	@0.7468					
BUN/CRE	Mean	15.69	k	18.43	20.34	16.28	17.71
[Ratio]	S.D.	2.007		5.446	1.253	0.527	1.936
day -4	N	3		3	3	3	3
	P-Value	@0.2772					
ALP	Mean	80	k	77	87	62	87
[IU/L]	S.D.	6.0		29.0	9.8	6.9	24.2
day -4	N	3		3	3	3	3
	P-Value	@0.2829					
ALT	Mean	30	a	34	45	34	38
[IU/L]	S.D.	4.0		6.0	14.2	9.5	6.7
day -4	N	3		3	3	3	3
	P-Value	@0.3639					
AST	Mean	28	a	26	28	28	32
[IU/L]	S.D.	5.5		5.6	7.0	4.4	4.0
day -4	N	3		3	3	3	3
	P-Value	@0.7832					
CK	Mean	194	a	152	180	161	207
[U/L]	S.D.	88.7		12.3	40.0	47.8	35.5
day -4	N	3		3	3	3	3
	P-Value	@0.6744					
TBIL	Mean	0.1	k	0.1	0.1	0.1	0.1
[mg/dL]	S.D.	0.00		0.00	0.00	0.00	0.06
day -4	N	3		3	3	3	3
	P-Value	@0.4060					
CHOL	Mean	136	k	120	113	140	141
[mg/dL]	S.D.	23.0		0.6	11.3	10.1	31.5
day -4	N	3		3	3	3	3
	P-Value	@0.1099					
TG	Mean	40	a	42	28	28	34
[mg/dL]	S.D.	21.0		24.9	5.7	7.2	12.5
day -4	N	3		3	3	3	3
	P-Value	@0.7423					
GLU	Mean	110	k	119	119	113	115
[mg/dL]	S.D.	10.2		13.7	13.1	1.7	3.5
day -4	N	3		3	3	3	3
	P-Value	@0.7469					

a=ANOVA; k=KRUSKAL-WALLIS

Table F-1a – Summary of Clinical Chemistry Data – Pre-Test (vs. Group 1)

FEMALE

		G 1 / F Saline	G 2 / F Vehicle	G 3 / F Low 0.019 mg/L	G 4 / F Mid 0.038 mg/L	G 5 / F High 0.075 mg/L
NA	Mean	135 k	128	135	150	134
[mmol/L]	S.D.	20.8	20.5	1.2	4.0	21.9
day -5	N	3	3	3	3	3
	P-Value	@0.1795				
K	Mean	4.0 k	3.9	4.5	4.1	4.1
[mmol/L]	S.D.	0.60	0.62	0.31	0.17	0.69
day -5	N	3	3	3	3	3
	P-Value	@0.6144				
CL	Mean	99 a	97	104	111	100
[mmol/L]	S.D.	14.8	14.7	3.2	3.8	14.8
day -5	N	3	3	3	3	3
	P-Value	@0.6457				
CALC	Mean	11.2 k	10.8	10.5	10.5	10.9
[mg/dL]	S.D.	0.44	0.60	0.30	0.29	0.30
day -5	N	3	3	3	3	3
	P-Value	@0.2975				
CRE	Mean	0.77 a	0.93	0.75	0.79	0.81
[mg/dL]	S.D.	0.191	0.123	0.221	0.047	0.153
day -5	N	3	3	3	3	3
	P-Value	@0.6546				
PO4	Mean	7.4 k	7.2	7.1	6.1	6.8
[mg/dL]	S.D.	0.93	0.30	0.64	0.53	0.65
day -5	N	3	3	3	3	3
	P-Value	@0.1402				
TP	Mean	6.0 a	6.0	5.5	5.9	5.8
[g/dL]	S.D.	0.45	0.65	0.45	0.32	0.32
day -5	N	3	3	3	3	3
	P-Value	@0.7132				
ALB	Mean	3.2 k	3.0	3.0	3.1	3.1
[g/dL]	S.D.	0.17	0.12	0.06	0.21	0.17
day -5	N	3	3	3	3	3
	P-Value	@0.5026				
GLOB	Mean	2.8 a	2.9	2.6	2.8	2.7
[g/dL]	S.D.	0.32	0.67	0.40	0.30	0.42
day -5	N	3	3	3	3	3
	P-Value	@0.8805				
A/G	Mean	1.1 a	1.1	1.2	1.1	1.2
[-]	S.D.	0.10	0.29	0.16	0.15	0.22
day -5	N	3	3	3	3	3
	P-Value	@0.9725				

k=KRUSKAL-WALLIS; a=ANOVA

Table F-1a – Summary of Clinical Chemistry Data – Pre-Test (vs. Group 1)

FEMALE

		G 1 / F Saline	G 2 / F Vehicle	G 3 / F Low 0.019 mg/L	G 4 / F Mid 0.038 mg/L	G 5 / F High 0.075 mg/L
BUN	Mean	16 k	17	15	15	16
[mg/dL]	S.D.	2.1	1.5	6.6	3.1	0.6
day -5	N	3	3	3	3	3
	P-Value	@0.7849				
BUN/CRE	Mean	22.37 k	18.67	19.52	18.75	20.56
[-]	S.D.	7.674	1.567	2.892	4.578	3.905
day -5	N	3	3	3	3	3
	P-Value	@0.9742				
ALP	Mean	72 a	74	84	100	89
[IU/L]	S.D.	19.9	19.9	27.5	11.8	12.6
day -5	N	3	3	3	3	3
	P-Value	@0.4292				
ALT	Mean	36 a	34	37	35	31
[IU/L]	S.D.	10.4	7.6	7.4	8.4	7.9
day -5	N	3	3	3	3	3
	P-Value	@0.9271				
AST	Mean	36 k	29	34	32	32
[IU/L]	S.D.	11.9	4.2	1.0	5.8	8.3
day -5	N	3	3	3	3	3
	P-Value	@0.7538				
CK	Mean	325 a	203	242	170	161
[U/L]	S.D.	263.0	14.5	76.8	47.5	40.1
day -5	N	3	3	3	3	3
	P-Value	@0.5299				
TBIL	Mean	0.1	0.1	0.1	0.1	0.1
[mg/dL]	S.D.	0.00	0.00	0.00	0.00	0.00
day -5	N	3	3	3	3	3
	P-Value	NC				
CHOL	Mean	120 a	134	109	128	122
[mg/dL]	S.D.	19.5	8.6	15.9	14.6	20.0
day -5	N	3	3	3	3	3
	P-Value	@0.4618				
TG	Mean	29 k	30	40	32	29
[mg/dL]	S.D.	2.1	7.5	15.2	8.2	7.2
day -5	N	3	3	3	3	3
	P-Value	@0.8604				
GLU	Mean	113 a	105	98	101	101
[mg/dL]	S.D.	12.1	10.6	10.5	8.5	11.4
day -5	N	3	3	3	3	3
	P-Value	@0.4872				

k=KRUSKAL-WALLIS; a=ANOVA

Table F-1b – Summary of Clinical Chemistry Data – Pre-Test (vs. Group 2)

MALE

		G 2 / M Vehicle	G 1 / M Saline	G 3 / M Low 0.019 mg/L	G 4 / M Mid 0.038 mg/L	G 5 / M High 0.075 mg/L
NA	Mean	130 k	131	140	137	144
[mmol/L]	S.D.	22.8	15.9	6.1	14.6	6.0
day -4	N	3	3	3	3	3
	P-Value	@0.7371				
K	Mean	4.2 a	4.0	4.1	4.1	4.2
[mmol/L]	S.D.	0.67	0.20	0.21	0.15	0.44
day -4	N	3	3	3	3	3
	P-Value	@0.9656				
CL	Mean	101 a	100	107	102	107
[mmol/L]	S.D.	15.4	12.2	5.3	9.0	6.6
day -4	N	3	3	3	3	3
	P-Value	@0.8565				
CALC	Mean	10.9 a	10.8	10.6	11.0	10.7
[mg/dL]	S.D.	0.25	0.85	0.26	0.46	0.38
day -4	N	3	3	3	3	3
	P-Value	@0.8622				
CRE	Mean	0.78 a	0.76	0.70	0.74	0.81
[mg/dL]	S.D.	0.121	0.095	0.032	0.042	0.026
day -4	N	3	3	3	3	3
	P-Value	@0.4993				
PO4	Mean	7.6 k	7.4	6.5	6.8	6.8
[mg/dL]	S.D.	0.55	0.12	0.46	1.08	0.45
day -4	N	3	3	3	3	3
	P-Value	@0.1621				
TP	Mean	6.0 k	5.8	6.1	6.1	6.1
[g/dL]	S.D.	0.40	0.67	0.17	0.36	0.40
day -4	N	3	3	3	3	3
	P-Value	@0.9120				
ALB	Mean	3.2 a	3.1	3.1	3.3	3.1
[g/dL]	S.D.	0.15	0.21	0.21	0.15	0.26
day -4	N	3	3	3	3	3
	P-Value	@0.4333				
GLOB	Mean	2.8 k	2.8	3.0	2.8	3.0
[g/dL]	S.D.	0.38	0.47	0.15	0.25	0.40
day -4	N	3	3	3	3	3
	P-Value	@0.6518				
A/G	Mean	1.2 a	1.1	1.0	1.2	1.0
[-]	S.D.	0.16	0.13	0.11	0.10	0.18
day -4	N	3	3	3	3	3
	P-Value	@0.3725				

k=KRUSKAL-WALLIS; a=ANOVA

Table F-1b – Summary of Clinical Chemistry Data – Pre-Test (vs. Group 2)

MALE

		G 2 / M Vehicle	G 1 / M Saline	G 3 / M Low 0.019 mg/L	G 4 / M Mid 0.038 mg/L	G 5 / M High 0.075 mg/L
BUN	Mean	15 a	12	14	12	14
[mg/dL]	S.D.	6.4	3.0	1.5	1.0	1.5
day -4	N	3	3	3	3	3
	P-Value	@0.7468				
BUN/CRE	Mean	18.43 k	15.69	20.34	16.28	17.71
[Ratio]	S.D.	5.446	2.007	1.253	0.527	1.936
day -4	N	3	3	3	3	3
	P-Value	@0.2772				
ALP	Mean	77 k	80	87	62	87
[IU/L]	S.D.	29.0	6.0	9.8	6.9	24.2
day -4	N	3	3	3	3	3
	P-Value	@0.2829				
ALT	Mean	34 a	30	45	34	38
[IU/L]	S.D.	6.0	4.0	14.2	9.5	6.7
day -4	N	3	3	3	3	3
	P-Value	@0.3639				
AST	Mean	26 a	28	28	28	32
[IU/L]	S.D.	5.6	5.5	7.0	4.4	4.0
day -4	N	3	3	3	3	3
	P-Value	@0.7832				
CK	Mean	152 a	194	180	161	207
[U/L]	S.D.	12.3	88.7	40.0	47.8	35.5
day -4	N	3	3	3	3	3
	P-Value	@0.6744				
TBIL	Mean	0.1 k	0.1	0.1	0.1	0.1
[mg/dL]	S.D.	0.00	0.00	0.00	0.00	0.06
day -4	N	3	3	3	3	3
	P-Value	@0.4060				
CHOL	Mean	120 k	136	113	140	141
[mg/dL]	S.D.	0.6	23.0	11.3	10.1	31.5
day -4	N	3	3	3	3	3
	P-Value	@0.1099				
TG	Mean	42 a	40	28	28	34
[mg/dL]	S.D.	24.9	21.0	5.7	7.2	12.5
day -4	N	3	3	3	3	3
	P-Value	@0.7423				
GLU	Mean	119 k	110	119	113	115
[mg/dL]	S.D.	13.7	10.2	13.1	1.7	3.5
day -4	N	3	3	3	3	3
	P-Value	@0.7469				

a=ANOVA; k=KRUSKAL-WALLIS

Table F-1b – Summary of Clinical Chemistry Data – Pre-Test (vs. Group 2)

FEMALE

		G 2 / F Vehicle	G 1 / F Saline	G 3 / F Low 0.019 mg/L	G 4 / F Mid 0.038 mg/L	G 5 / F High 0.075 mg/L
NA	Mean	128 k	135	135	150	134
[mmol/L]	S.D.	20.5	20.8	1.2	4.0	21.9
day -5	N	3	3	3	3	3
	P-Value	@0.1795				
K	Mean	3.9 k	4.0	4.5	4.1	4.1
[mmol/L]	S.D.	0.62	0.60	0.31	0.17	0.69
day -5	N	3	3	3	3	3
	P-Value	@0.6144				
CL	Mean	97 a	99	104	111	100
[mmol/L]	S.D.	14.7	14.8	3.2	3.8	14.8
day -5	N	3	3	3	3	3
	P-Value	@0.6457				
CALC	Mean	10.8 k	11.2	10.5	10.5	10.9
[mg/dL]	S.D.	0.60	0.44	0.30	0.29	0.30
day -5	N	3	3	3	3	3
	P-Value	@0.2975				
CRE	Mean	0.93 a	0.77	0.75	0.79	0.81
[mg/dL]	S.D.	0.123	0.191	0.221	0.047	0.153
day -5	N	3	3	3	3	3
	P-Value	@0.6546				
PO4	Mean	7.2 k	7.4	7.1	6.1	6.8
[mg/dL]	S.D.	0.30	0.93	0.64	0.53	0.65
day -5	N	3	3	3	3	3
	P-Value	@0.1402				
TP	Mean	6.0 a	6.0	5.5	5.9	5.8
[g/dL]	S.D.	0.65	0.45	0.45	0.32	0.32
day -5	N	3	3	3	3	3
	P-Value	@0.7132				
ALB	Mean	3.0 k	3.2	3.0	3.1	3.1
[g/dL]	S.D.	0.12	0.17	0.06	0.21	0.17
day -5	N	3	3	3	3	3
	P-Value	@0.5026				
GLOB	Mean	2.9 a	2.8	2.6	2.8	2.7
[g/dL]	S.D.	0.67	0.32	0.40	0.30	0.42
day -5	N	3	3	3	3	3
	P-Value	@0.8805				
A/G	Mean	1.1 a	1.1	1.2	1.1	1.2
[-]	S.D.	0.29	0.10	0.16	0.15	0.22
day -5	N	3	3	3	3	3
	P-Value	@0.9725				

k=KRUSKAL-WALLIS; a=ANOVA

Table F-1b – Summary of Clinical Chemistry Data – Pre-Test (vs. Group 2)

FEMALE

		G 2 / F Vehicle	G 1 / F Saline	G 3 / F Low 0.019 mg/L	G 4 / F Mid 0.038 mg/L	G 5 / F High 0.075 mg/L
BUN	Mean	17 k	16	15	15	16
[mg/dL]	S.D.	1.5	2.1	6.6	3.1	0.6
day -5	N	3	3	3	3	3
	P-Value	@0.7849				
BUN/CRE	Mean	18.67 k	22.37	19.52	18.75	20.56
[-]	S.D.	1.567	7.674	2.892	4.578	3.905
day -5	N	3	3	3	3	3
	P-Value	@0.9742				
ALP	Mean	74 a	72	84	100	89
[IU/L]	S.D.	19.9	19.9	27.5	11.8	12.6
day -5	N	3	3	3	3	3
	P-Value	@0.4292				
ALT	Mean	34 a	36	37	35	31
[IU/L]	S.D.	7.6	10.4	7.4	8.4	7.9
day -5	N	3	3	3	3	3
	P-Value	@0.9271				
AST	Mean	29 k	36	34	32	32
[IU/L]	S.D.	4.2	11.9	1.0	5.8	8.3
day -5	N	3	3	3	3	3
	P-Value	@0.7538				
CK	Mean	203 a	325	242	170	161
[U/L]	S.D.	14.5	263.0	76.8	47.5	40.1
day -5	N	3	3	3	3	3
	P-Value	@0.5299				
TBIL	Mean	0.1	0.1	0.1	0.1	0.1
[mg/dL]	S.D.	0.00	0.00	0.00	0.00	0.00
day -5	N	3	3	3	3	3
	P-Value	NC				
CHOL	Mean	134 a	120	109	128	122
[mg/dL]	S.D.	8.6	19.5	15.9	14.6	20.0
day -5	N	3	3	3	3	3
	P-Value	@0.4618				
TG	Mean	30 k	29	40	32	29
[mg/dL]	S.D.	7.5	2.1	15.2	8.2	7.2
day -5	N	3	3	3	3	3
	P-Value	@0.8604				
GLU	Mean	105 a	113	98	101	101
[mg/dL]	S.D.	10.6	12.1	10.5	8.5	11.4
day -5	N	3	3	3	3	3
	P-Value	@0.4872				

k=KRUSKAL-WALLIS; a=ANOVA

Table F-1c – Summary of Clinical Chemistry Data – Day 15 (vs. Group 1)

MALE

		G 1 / M Saline	G 2 / M Vehicle	G 3 / M Low 0.019 mg/L	G 4 / M Mid 0.038 mg/L	G 5 / M High 0.075 mg/L
NA	Mean	144 k	144	145	141	143
[mmol/L]	S.D.	1.2	1.5	0.0	2.6	2.3
day 15	N	3	3	3	3	3
	P-Value	@0.0992				
K	Mean	4.3 k	4.4	4.4	4.5	4.5
[mmol/L]	S.D.	0.17	0.15	0.25	0.30	0.32
day 15	N	3	3	3	3	3
	P-Value	@0.7511				
CL	Mean	109 k	107	107	105	106
[mmol/L]	S.D.	0.6	1.5	1.0	1.7	1.5
day 15	N	3	3	3	3	3
	P-Value	@0.0616				
CALC	Mean	10.4 k	10.4	10.2	10.2	10.3
[mg/dL]	S.D.	0.10	0.44	0.06	0.15	0.17
day 15	N	3	3	3	3	3
	P-Value	@0.4010				
CRE	Mean	0.68 a	0.63	0.62	0.61	0.67
[mg/dL]	S.D.	0.049	0.131	0.062	0.025	0.050
day 15	N	3	3	3	3	3
	P-Value	@0.6903				
PO4	Mean	6.2 a	6.9	5.9	6.0	6.5
[mg/dL]	S.D.	0.31	1.27	0.65	0.64	0.25
day 15	N	3	3	3	3	3
	P-Value	@0.4654				
TP	Mean	5.5 k	5.9	6.0	5.7	5.9
[g/dL]	S.D.	0.10	0.20	0.06	0.00	0.25
day 15	N	3	3	3	3	3
	P-Value	@0.0661				
ALB	Mean	3.0 k	3.2	3.1	3.1	3.0
[g/dL]	S.D.	0.10	0.12	0.26	0.12	0.12
day 15	N	3	3	3	3	3
	P-Value	@0.3654				
GLOB	Mean	2.5 k	2.7	2.9	2.6	2.8
[g/dL]	S.D.	0.17	0.12	0.32	0.12	0.35
day 15	N	3	3	3	3	3
	P-Value	@0.3213				
A/G	Mean	1.2 k	1.2	1.1	1.2	1.1
[-]	S.D.	0.12	0.05	0.23	0.10	0.17
day 15	N	3	3	3	3	3
	P-Value	@0.5967				

k=KRUSKAL-WALLIS; a=ANOVA

Table F-1c – Summary of Clinical Chemistry Data – Day 15 (vs. Group 1)

MALE

		G 1 / M Saline		G 2 / M Vehicle	G 3 / M Low 0.019 mg/L	G 4 / M Mid 0.038 mg/L	G 5 / M High 0.075 mg/L
BUN	Mean	12	k	11	11	12	13
[mg/dL]	S.D.	1.5		1.7	1.7	2.5	1.5
day 15	N	3		3	3	3	3
	P-Value	@0.5322					
BUN/CRE	Mean	17.04	a	17.54	18.03	19.12	19.97
[Ratio]	S.D.	1.470		1.830	4.352	4.568	3.667
day 15	N	3		3	3	3	3
	P-Value	@0.8308					
ALP	Mean	74	a	74	89	64	70
[IU/L]	S.D.	9.0		15.8	6.8	6.1	12.8
day 15	N	3		3	3	3	3
	P-Value	@0.1329					
ALT	Mean	38	k	44	63	59	51
[IU/L]	S.D.	2.1		0.6	22.9	28.2	21.2
day 15	N	3		3	3	3	3
	P-Value	@0.4569					
AST	Mean	24	a	27	29	32	26
[IU/L]	S.D.	3.0		4.7	11.0	4.7	3.6
day 15	N	3		3	3	3	3
	P-Value	@0.5997					
CK	Mean	182	a	155	279	217	124
[U/L]	S.D.	13.8		9.6	175.5	22.5	4.9
day 15	N	3		3	3	3	3
	P-Value	@0.0642					
TBIL	Mean	0.1	k	0.1	0.1	0.1	0.1
[mg/dL]	S.D.	0.06		0.06	0.00	0.06	0.00
day 15	N	3		3	3	3	3
	P-Value	@0.6747					
CHOL	Mean	131	a	120	125	145	125
[mg/dL]	S.D.	14.1		9.5	2.5	17.5	17.4
day 15	N	3		3	3	3	3
	P-Value	@0.2510					
TG	Mean	32	a	37	37	38	41
[mg/dL]	S.D.	8.5		2.5	7.2	4.7	8.5
day 15	N	3		3	3	3	3
	P-Value	@0.6281					
GLU	Mean	112	k	109	121	119	118
[mg/dL]	S.D.	6.0		11.5	6.4	14.1	10.6
day 15	N	3		3	3	3	3
	P-Value	@0.5101					

k=KRUSKAL-WALLIS; a=ANOVA

Table F-1c – Summary of Clinical Chemistry Data – Day 15 (vs. Group 1)

FEMALE

		G 1 / F Saline	G 2 / F Vehicle	G 3 / F Low 0.019 mg/L	G 4 / F Mid 0.038 mg/L	G 5 / F High 0.075 mg/L
NA	Mean	144 k	144	145	144	145
[mmol/L]	S.D.	0.0	1.0	1.5	0.6	1.2
day 15	N	3	3	3	3	3
	P-Value	@0.5135				
K	Mean	4.6 a	4.7	4.5	4.5	4.5
[mmol/L]	S.D.	0.10	0.10	0.15	0.32	0.26
day 15	N	3	3	3	3	3
	P-Value	@0.6761				
CL	Mean	107 k	108	109	108	108
[mmol/L]	S.D.	0.6	1.7	0.6	1.2	2.3
day 15	N	3	3	3	3	3
	P-Value	@0.3948				
CALC	Mean	10.6 k	10.3	10.2	10.6	10.3
[mg/dL]	S.D.	0.12	0.26	0.10	0.06	0.21
day 15	N	3	3	3	3	3
	P-Value	@0.0912				
CRE	Mean	0.66 k	0.72	0.54	0.55	0.67
[mg/dL]	S.D.	0.081	0.006	0.082	0.059	0.087
day 15	N	3	3	3	3	3
	P-Value	@0.1153				
PO4	Mean	6.2 k	6.0	6.5	6.7	5.6
[mg/dL]	S.D.	0.38	0.70	0.46	0.46	0.42
day 15	N	3	3	3	3	3
	P-Value	@0.1903				
TP	Mean	5.7 k	5.8	5.6	5.8	5.7
[g/dL]	S.D.	0.15	0.32	0.47	0.12	0.15
day 15	N	3	3	3	3	3
	P-Value	@0.7931				
ALB	Mean	3.2 k	3.1	3.0	3.1	3.1
[g/dL]	S.D.	0.15	0.12	0.00	0.23	0.20
day 15	N	3	3	3	3	3
	P-Value	@0.4144				
GLOB	Mean	2.4 a	2.6	2.6	2.7	2.6
[g/dL]	S.D.	0.15	0.38	0.47	0.20	0.35
day 15	N	3	3	3	3	3
	P-Value	@0.8862				
A/G	Mean	1.3 a	1.2	1.2	1.2	1.2
[-]	S.D.	0.13	0.22	0.20	0.17	0.23
day 15	N	3	3	3	3	3
	P-Value	@0.8500				

k=KRUSKAL-WALLIS; a=ANOVA

Table F-1c – Summary of Clinical Chemistry Data – Day 15 (vs. Group 1)

FEMALE

		G 1 / F Saline	G 2 / F Vehicle	G 3 / F Low 0.019 mg/L	G 4 / F Mid 0.038 mg/L	G 5 / F High 0.075 mg/L
BUN	Mean	13 k	14	10	12	13
[mg/dL]	S.D.	0.6	2.6	2.1	2.5	3.5
day 15	N	3	3	3	3	3
	P-Value	@0.3656				
BUN/CRE	Mean	20.53 a	19.37	19.07	21.26	18.69
[-]	S.D.	2.803	3.793	2.066	5.489	3.263
day 15	N	3	3	3	3	3
	P-Value	@0.8990				
ALP	Mean	84 a	89	92	99	97
[IU/L]	S.D.	18.6	26.7	20.3	5.0	32.4
day 15	N	3	3	3	3	3
	P-Value	@0.9229				
ALT	Mean	39 a	49	36	70	39
[IU/L]	S.D.	5.0	14.5	9.8	50.1	8.3
day 15	N	3	3	3	3	3
	P-Value	@0.4586				
AST	Mean	23 k	24	27	29	27
[IU/L]	S.D.	1.7	4.6	5.9	7.0	5.5
day 15	N	3	3	3	3	3
	P-Value	@0.6670				
CK	Mean	132 a	181	146	162	116
[U/L]	S.D.	41.6	9.6	2.5	80.7	17.4
day 15	N	3	3	3	3	3
	P-Value	@0.3952				
TBIL	Mean	0.1 k	0.1	0.1	0.1	0.1
[mg/dL]	S.D.	0.00	0.00	0.06	0.00	0.00
day 15	N	3	3	3	3	3
	P-Value	@0.4060				
CHOL	Mean	112 d	139	94	127	118
[mg/dL]	S.D.	15.6	5.5	10.3	17.0	21.1
day 15	N	3	3	3	3	3
	P-Value	@0.0428	0.1548	0.4429	0.5898	0.9700
TG	Mean	25 k	41	31	32	34
[mg/dL]	S.D.	5.9	11.1	5.5	9.0	5.2
day 15	N	3	3	3	3	3
	P-Value	@0.3185				
GLU	Mean	102 a	101	107	105	105
[mg/dL]	S.D.	7.0	3.6	8.1	6.8	11.4
day 15	N	3	3	3	3	3
	P-Value	@0.8820				

k=KRUSKAL-WALLIS; a=ANOVA; d=ANOVA-DUNNETT

Table F-1d – Summary of Clinical Chemistry Data – Day 15 (vs. Group 2)

MALE

		G 2 / M Vehicle	G 1 / M Saline	G 3 / M Low 0.019 mg/L	G 4 / M Mid 0.038 mg/L	G 5 / M High 0.075 mg/L
NA	Mean	144 k	144	145	141	143
[mmol/L]	S.D.	1.5	1.2	0.0	2.6	2.3
day 15	N	3	3	3	3	3
	P-Value	@0.0992				
K	Mean	4.4 k	4.3	4.4	4.5	4.5
[mmol/L]	S.D.	0.15	0.17	0.25	0.30	0.32
day 15	N	3	3	3	3	3
	P-Value	@0.7511				
CL	Mean	107 k	109	107	105	106
[mmol/L]	S.D.	1.5	0.6	1.0	1.7	1.5
day 15	N	3	3	3	3	3
	P-Value	@0.0616				
CALC	Mean	10.4 k	10.4	10.2	10.2	10.3
[mg/dL]	S.D.	0.44	0.10	0.06	0.15	0.17
day 15	N	3	3	3	3	3
	P-Value	@0.4010				
CRE	Mean	0.63 a	0.68	0.62	0.61	0.67
[mg/dL]	S.D.	0.131	0.049	0.062	0.025	0.050
day 15	N	3	3	3	3	3
	P-Value	@0.6903				
PO4	Mean	6.9 a	6.2	5.9	6.0	6.5
[mg/dL]	S.D.	1.27	0.31	0.65	0.64	0.25
day 15	N	3	3	3	3	3
	P-Value	@0.4654				
TP	Mean	5.9 k	5.5	6.0	5.7	5.9
[g/dL]	S.D.	0.20	0.10	0.06	0.00	0.25
day 15	N	3	3	3	3	3
	P-Value	@0.0661				
ALB	Mean	3.2 k	3.0	3.1	3.1	3.0
[g/dL]	S.D.	0.12	0.10	0.26	0.12	0.12
day 15	N	3	3	3	3	3
	P-Value	@0.3654				
GLOB	Mean	2.7 k	2.5	2.9	2.6	2.8
[g/dL]	S.D.	0.12	0.17	0.32	0.12	0.35
day 15	N	3	3	3	3	3
	P-Value	@0.3213				
A/G	Mean	1.2 k	1.2	1.1	1.2	1.1
[-]	S.D.	0.05	0.12	0.23	0.10	0.17
day 15	N	3	3	3	3	3
	P-Value	@0.5967				

k=KRUSKAL-WALLIS; a=ANOVA

Table F-1d – Summary of Clinical Chemistry Data – Day 15 (vs. Group 2)

MALE

		G 2 / M Vehicle	G 1 / M Saline	G 3 / M Low 0.019 mg/L	G 4 / M Mid 0.038 mg/L	G 5 / M High 0.075 mg/L
BUN	Mean	11 k	12	11	12	13
[mg/dL]	S.D.	1.7	1.5	1.7	2.5	1.5
day 15	N	3	3	3	3	3
	P-Value	@0.5322				
BUN/CRE	Mean	17.54 a	17.04	18.03	19.12	19.97
[Ratio]	S.D.	1.830	1.470	4.352	4.568	3.667
day 15	N	3	3	3	3	3
	P-Value	@0.8308				
ALP	Mean	74 a	74	89	64	70
[IU/L]	S.D.	15.8	9.0	6.8	6.1	12.8
day 15	N	3	3	3	3	3
	P-Value	@0.1329				
ALT	Mean	44 k	38	63	59	51
[IU/L]	S.D.	0.6	2.1	22.9	28.2	21.2
day 15	N	3	3	3	3	3
	P-Value	@0.4569				
AST	Mean	27 a	24	29	32	26
[IU/L]	S.D.	4.7	3.0	11.0	4.7	3.6
day 15	N	3	3	3	3	3
	P-Value	@0.5997				
CK	Mean	155 a	182	279	217	124
[U/L]	S.D.	9.6	13.8	175.5	22.5	4.9
day 15	N	3	3	3	3	3
	P-Value	@0.0642				
TBIL	Mean	0.1 k	0.1	0.1	0.1	0.1
[mg/dL]	S.D.	0.06	0.06	0.00	0.06	0.00
day 15	N	3	3	3	3	3
	P-Value	@0.6747				
CHOL	Mean	120 a	131	125	145	125
[mg/dL]	S.D.	9.5	14.1	2.5	17.5	17.4
day 15	N	3	3	3	3	3
	P-Value	@0.2510				
TG	Mean	37 a	32	37	38	41
[mg/dL]	S.D.	2.5	8.5	7.2	4.7	8.5
day 15	N	3	3	3	3	3
	P-Value	@0.6281				
GLU	Mean	109 k	112	121	119	118
[mg/dL]	S.D.	11.5	6.0	6.4	14.1	10.6
day 15	N	3	3	3	3	3
	P-Value	@0.5101				

k=KRUSKAL-WALLIS; a=ANOVA

Table F-1d – Summary of Clinical Chemistry Data – Day 15 (vs. Group 2)

FEMALE

		G 2 / F Vehicle	G 1 / F Saline	G 3 / F Low 0.019 mg/L	G 4 / F Mid 0.038 mg/L	G 5 / F High 0.075 mg/L
NA	Mean	144 k	144	145	144	145
[mmol/L]	S.D.	1.0	0.0	1.5	0.6	1.2
day 15	N	3	3	3	3	3
	P-Value	@0.5135				
K	Mean	4.7 a	4.6	4.5	4.5	4.5
[mmol/L]	S.D.	0.10	0.10	0.15	0.32	0.26
day 15	N	3	3	3	3	3
	P-Value	@0.6761				
CL	Mean	108 k	107	109	108	108
[mmol/L]	S.D.	1.7	0.6	0.6	1.2	2.3
day 15	N	3	3	3	3	3
	P-Value	@0.3948				
CALC	Mean	10.3 k	10.6	10.2	10.6	10.3
[mg/dL]	S.D.	0.26	0.12	0.10	0.06	0.21
day 15	N	3	3	3	3	3
	P-Value	@0.0912				
CRE	Mean	0.72 k	0.66	0.54	0.55	0.67
[mg/dL]	S.D.	0.006	0.081	0.082	0.059	0.087
day 15	N	3	3	3	3	3
	P-Value	@0.1153				
PO4	Mean	6.0 k	6.2	6.5	6.7	5.6
[mg/dL]	S.D.	0.70	0.38	0.46	0.46	0.42
day 15	N	3	3	3	3	3
	P-Value	@0.1903				
TP	Mean	5.8 k	5.7	5.6	5.8	5.7
[g/dL]	S.D.	0.32	0.15	0.47	0.12	0.15
day 15	N	3	3	3	3	3
	P-Value	@0.7931				
ALB	Mean	3.1 k	3.2	3.0	3.1	3.1
[g/dL]	S.D.	0.12	0.15	0.00	0.23	0.20
day 15	N	3	3	3	3	3
	P-Value	@0.4144				
GLOB	Mean	2.6 a	2.4	2.6	2.7	2.6
[g/dL]	S.D.	0.38	0.15	0.47	0.20	0.35
day 15	N	3	3	3	3	3
	P-Value	@0.8862				
A/G	Mean	1.2 a	1.3	1.2	1.2	1.2
[-]	S.D.	0.22	0.13	0.20	0.17	0.23
day 15	N	3	3	3	3	3
	P-Value	@0.8500				

k=KRUSKAL-WALLIS; a=ANOVA

Table F-1d – Summary of Clinical Chemistry Data – Day 15 (vs. Group 2)

FEMALE

		G 2 / F Vehicle	G 1 / F Saline	G 3 / F Low 0.019 mg/L	G 4 / F Mid 0.038 mg/L	G 5 / F High 0.075 mg/L
BUN	Mean	14 k	13	10	12	13
[mg/dL]	S.D.	2.6	0.6	2.1	2.5	3.5
day 15	N	3	3	3	3	3
	P-Value	@0.3656				
BUN/CRE	Mean	19.37 a	20.53	19.07	21.26	18.69
[-]	S.D.	3.793	2.803	2.066	5.489	3.263
day 15	N	3	3	3	3	3
	P-Value	@0.8990				
ALP	Mean	89 a	84	92	99	97
[IU/L]	S.D.	26.7	18.6	20.3	5.0	32.4
day 15	N	3	3	3	3	3
	P-Value	@0.9229				
ALT	Mean	49 a	39	36	70	39
[IU/L]	S.D.	14.5	5.0	9.8	50.1	8.3
day 15	N	3	3	3	3	3
	P-Value	@0.4586				
AST	Mean	24 k	23	27	29	27
[IU/L]	S.D.	4.6	1.7	5.9	7.0	5.5
day 15	N	3	3	3	3	3
	P-Value	@0.6670				
CK	Mean	181 a	132	146	162	116
[U/L]	S.D.	9.6	41.6	2.5	80.7	17.4
day 15	N	3	3	3	3	3
	P-Value	@0.3952				
TBIL	Mean	0.1 k	0.1	0.1	0.1	0.1
[mg/dL]	S.D.	0.00	0.00	0.06	0.00	0.00
day 15	N	3	3	3	3	3
	P-Value	@0.4060				
CHOL	Mean	139 d	112	94 *	127	118
[mg/dL]	S.D.	5.5	15.6	10.3	17.0	21.1
day 15	N	3	3	3	3	3
	P-Value	@0.0428	0.1548	0.0149	0.7291	0.3081
TG	Mean	41 k	25	31	32	34
[mg/dL]	S.D.	11.1	5.9	5.5	9.0	5.2
day 15	N	3	3	3	3	3
	P-Value	@0.3185				
GLU	Mean	101 a	102	107	105	105
[mg/dL]	S.D.	3.6	7.0	8.1	6.8	11.4
day 15	N	3	3	3	3	3
	P-Value	@0.8820				

k=KRUSKAL-WALLIS; a=ANOVA; d=ANOVA-DUNNETT; * = p < 0.05

Table F-2a– Summary of Hematology Data – Pre-Test (vs. Group 1)

MALE

		G 1 / M Saline		G 2 / M Vehicle	G 3 / M Low 0.019 mg/L	G 4 / M Mid 0.038 mg/L	G 5 / M High 0.075 mg/L
WBC	Mean	10.37	k	10.84	11.65	9.98	11.90
[x10e3/µL]	S.D.	1.787		2.060	2.054	2.396	0.779
day -4	N	3		3	3	3	3
	P-Value	@0.4975					
RBC	Mean	7.14	a	7.36	6.96	7.48	7.22
[x10e6/µL]	S.D.	0.246		0.516	0.618	0.657	0.673
day -4	N	3		3	3	3	3
	P-Value	@0.8216					
HGB	Mean	16.2	k	16.1	15.8	16.8	16.1
[g/dL]	S.D.	0.70		0.72	0.64	1.30	1.00
day -4	N	3		3	3	3	3
	P-Value	@0.6793					
HCT	Mean	47.6	a	48.0	46.2	49.8	46.3
[%]	S.D.	2.07		3.36	2.98	4.19	3.04
day -4	N	3		3	3	3	3
	P-Value	@0.6425					
MCV	Mean	66.7	a	65.3	66.5	66.7	64.2
[fL]	S.D.	0.81		4.20	1.98	2.15	1.85
day -4	N	3		3	3	3	3
	P-Value	@0.6761					
MCH	Mean	22.7	a	22.0	22.8	22.5	22.3
[pg]	S.D.	0.45		1.59	1.17	0.72	0.76
day -4	N	3		3	3	3	3
	P-Value	@0.8429					
MCHC	Mean	34.1	k	33.6	34.3	33.8	34.8
[g/dL]	S.D.	0.40		1.10	0.81	0.29	0.15
day -4	N	3		3	3	3	3
	P-Value	@0.1646					
PLT	Mean	298	a	339	341	343	354
[x10e3/µL]	S.D.	72.5		33.7	33.8	46.8	65.0
day -4	N	3		3	3	3	3
	P-Value	@0.7354					
%NEUT	Mean	52.0	a	57.9	62.7	57.0	62.2
[%]	S.D.	7.65		3.28	2.48	2.76	2.09
day -4	N	3		3	3	3	3
	P-Value	@0.0591					
%LYMPH	Mean	34.7	k	33.2	25.9	34.2	29.5
[%]	S.D.	8.41		5.17	0.72	3.63	2.29
day -4	N	3		3	3	3	3
	P-Value	@0.0691					
%MONO	Mean	7.9	a	6.2	7.3	5.9	5.2
[%]	S.D.	1.28		1.82	1.65	1.55	0.59
day -4	N	3		3	3	3	3
	P-Value	@0.2370					

k=KRUSKAL-WALLIS; a=ANOVA

Table F-2a – Summary of Hematology Data – Pre-Test (vs. Group 1)

MALE

		G 1 / M Saline	G 2 / M Vehicle	G 3 / M Low 0.019 mg/L	G 4 / M Mid 0.038 mg/L	G 5 / M High 0.075 mg/L
%EOS	Mean	4.6 a	2.1	3.6	2.3	2.6
[%]	S.D.	3.56	0.78	2.57	0.26	1.19
day -4	N	3	3	3	3	3
	P-Value	@0.5557				
%BASO	Mean	0.4 k	0.3	0.3	0.3	0.3
[%]	S.D.	0.23	0.10	0.00	0.06	0.10
day -4	N	3	3	3	3	3
	P-Value	@0.8796				
#NEUT	Mean	5.38 a	6.23	7.27	5.65	7.40
[x10e3/µL]	S.D.	1.201	0.867	1.002	1.195	0.390
day -4	N	3	3	3	3	3
	P-Value	@0.0956				
#LYMPH	Mean	3.65 k	3.66	3.02	3.45	3.52
[x10e3/µL]	S.D.	1.298	1.242	0.481	1.054	0.292
day -4	N	3	3	3	3	3
	P-Value	@0.8764				
#MONO	Mean	0.83 a	0.67	0.86	0.58	0.63
[x10e3/µL]	S.D.	0.255	0.197	0.274	0.205	0.116
day -4	N	3	3	3	3	3
	P-Value	@0.4925				
#EOS	Mean	0.43 a	0.22	0.45	0.24	0.31
[x10e3/µL]	S.D.	0.250	0.046	0.388	0.078	0.163
day -4	N	3	3	3	3	3
	P-Value	@0.6002				
#BASO	Mean	0.04 k	0.04	0.04	0.04	0.03
[x10e3/µL]	S.D.	0.015	0.012	0.012	0.015	0.012
day -4	N	3	3	3	3	3
	P-Value	@0.9396				
%RETIC	Mean	0.65 a	0.76	0.90	0.83	0.77
[%]	S.D.	0.090	0.311	0.055	0.469	0.147
day -4	N	3	3	3	3	3
	P-Value	@0.8257				
#RETIC	Mean	46.8 a	57.1	62.8	61.1	55.6
[x10e9/L]	S.D.	6.30	26.90	3.75	34.25	12.26
day -4	N	3	3	3	3	3
	P-Value	@0.8850				
%LUC	Mean	0.3 k	0.2	0.2	0.3	0.2
[%]	S.D.	0.10	0.12	0.06	0.12	0.06
day -4	N	3	3	3	3	3
	P-Value	@0.3340				
#LUC	Mean	0.04 k	0.02	0.02	0.03	0.02
[x10e3/µL]	S.D.	0.012	0.006	0.000	0.017	0.006
day -4	N	3	3	3	3	3
	P-Value	@0.1988				

a=ANOVA; k=KRUSKAL-WALLIS

Table F-2a – Summary of Hematology Data – Pre-Test (vs. Group 1)

FEMALE

		G 1 / F Saline	G 2 / F Vehicle	G 3 / F Low 0.019 mg/L	G 4 / F Mid 0.038 mg/L	G 5 / F High 0.075 mg/L
WBC	Mean	12.59 a	11.47	14.62	12.64	12.65
[x10e3/µL]	S.D.	0.283	2.174	6.567	1.926	2.462
day -5	N	3	3	3	3	3
	P-Value	@0.8472				
RBC	Mean	7.52 k	6.83	7.20	7.29	7.59
[x10e6/µL]	S.D.	0.792	0.441	0.745	0.916	0.954
day -5	N	3	3	3	3	3
	P-Value	@0.7541				
HGB	Mean	16.9 a	15.4	15.8	16.8	17.1
[g/dL]	S.D.	1.36	0.70	0.50	1.71	2.14
day -5	N	3	3	3	3	3
	P-Value	@0.5531				
HCT	Mean	48.8 a	45.1	47.1	48.9	49.7
[%]	S.D.	4.18	1.95	2.08	4.28	5.90
day -5	N	3	3	3	3	3
	P-Value	@0.6472				
MCV	Mean	65.1 k	66.2	65.7	67.2	65.5
[fL]	S.D.	2.60	2.08	3.88	2.65	0.46
day -5	N	3	3	3	3	3
	P-Value	@0.7052				
MCH	Mean	22.4 k	22.6	22.1	23.1	22.5
[pg]	S.D.	0.72	0.70	1.71	0.69	0.00
day -5	N	3	3	3	3	3
	P-Value	@0.7687				
MCHC	Mean	34.5 k	34.2	33.6	34.3	34.3
[g/dL]	S.D.	0.46	0.06	0.70	0.46	0.21
day -5	N	3	3	3	3	3
	P-Value	@0.3749				
PLT	Mean	331 k	346	357	335	318
[x10e3/µL]	S.D.	33.5	15.8	49.7	67.6	85.5
day -5	N	3	3	3	3	3
	P-Value	@0.9383				
%NEUT	Mean	62.7 a	66.0	60.9	59.1	60.6
[%]	S.D.	5.09	14.01	8.97	7.08	6.53
day -5	N	3	3	3	3	3
	P-Value	@0.8946				
%LYMPH	Mean	27.6 a	26.4	28.4	31.2	29.4
[%]	S.D.	4.79	13.97	7.46	5.42	6.72
day -5	N	3	3	3	3	3
	P-Value	@0.9621				
%MONO	Mean	7.0 a	5.9	8.0	7.6	6.6
[%]	S.D.	0.32	0.93	0.51	1.35	1.61
day -5	N	3	3	3	3	3
	P-Value	@0.2265				

a=ANOVA; k=KRUSKAL-WALLIS

Table F-2a – Summary of Hematology Data – Pre-Test (vs. Group 1)

FEMALE

		G 1 / F Saline		G 2 / F Vehicle	G 3 / F Low 0.019 mg/L	G 4 / F Mid 0.038 mg/L	G 5 / F High 0.075 mg/L
%EOS	Mean	2.2	a	1.4	2.3	1.4	2.7
[%]	S.D.	0.35		0.74	1.78	0.65	1.80
day -5	N	3		3	3	3	3
	P-Value	@0.6085					
%BASO	Mean	0.3	k	0.2	0.3	0.3	0.4
[%]	S.D.	0.06		0.06	0.06	0.15	0.21
day -5	N	3		3	3	3	3
	P-Value	@0.1863					
#NEUT	Mean	7.88	a	7.77	9.24	7.53	7.74
[x10e3/µL]	S.D.	0.484		2.963	5.499	1.861	2.268
day -5	N	3		3	3	3	3
	P-Value	@0.9598					
#LYMPH	Mean	3.48	a	2.83	3.85	3.89	3.67
[x10e3/µL]	S.D.	0.672		0.960	0.787	0.457	0.829
day -5	N	3		3	3	3	3
	P-Value	@0.4685					
#MONO	Mean	0.87	a	0.68	1.18	0.95	0.83
[x10e3/µL]	S.D.	0.035		0.191	0.558	0.217	0.241
day -5	N	3		3	3	3	3
	P-Value	@0.4074					
#EOS	Mean	0.28	a	0.16	0.30	0.19	0.32
[x10e3/µL]	S.D.	0.050		0.090	0.215	0.091	0.160
day -5	N	3		3	3	3	3
	P-Value	@0.4965					
#BASO	Mean	0.04	k	0.02	0.04	0.04	0.06
[x10e3/µL]	S.D.	0.006		0.006	0.006	0.017	0.035
day -5	N	3		3	3	3	3
	P-Value	@0.1111					
%RETIC	Mean	0.66	k	0.66	1.20	1.09	0.79
[%]	S.D.	0.079		0.115	1.271	0.117	0.165
day -5	N	3		3	3	3	3
	P-Value	@0.3416					
#RETIC	Mean	49.2	k	45.6	82.2	79.7	60.4
[x10e9/L]	S.D.	3.87		10.46	84.53	15.36	16.95
day -5	N	3		3	3	3	3
	P-Value	@0.3299					
%LUC	Mean	0.2	k	0.1	0.2	0.3	0.3
[%]	S.D.	0.06		0.15	0.06	0.20	0.10
day -5	N	3		3	3	3	3
	P-Value	@0.4046					
#LUC	Mean	0.03	k	0.01	0.02	0.04	0.04
[x10e3/µL]	S.D.	0.010		0.015	0.006	0.030	0.020
day -5	N	3		3	3	3	3
	P-Value	@0.3851					

a=ANOVA; k=KRUSKAL-WALLIS

Table F-2b– Summary of Hematology Data – Pre-Test (vs. Group 2)

MALE

		G 2 / M Vehicle	G 1 / M Saline	G 3 / M Low 0.019 mg/L	G 4 / M Mid 0.038 mg/L	G 5 / M High 0.075 mg/L
WBC	Mean	10.84 k	10.37	11.65	9.98	11.90
[x10e3/µL]	S.D.	2.060	1.787	2.054	2.396	0.779
day -4	N	3	3	3	3	3
	P-Value	@0.4975				
RBC	Mean	7.36 a	7.14	6.96	7.48	7.22
[x10e6/µL]	S.D.	0.516	0.246	0.618	0.657	0.673
day -4	N	3	3	3	3	3
	P-Value	@0.8216				
HGB	Mean	16.1 k	16.2	15.8	16.8	16.1
[g/dL]	S.D.	0.72	0.70	0.64	1.30	1.00
day -4	N	3	3	3	3	3
	P-Value	@0.6793				
HCT	Mean	48.0 a	47.6	46.2	49.8	46.3
[%]	S.D.	3.36	2.07	2.98	4.19	3.04
day -4	N	3	3	3	3	3
	P-Value	@0.6425				
MCV	Mean	65.3 a	66.7	66.5	66.7	64.2
[fL]	S.D.	4.20	0.81	1.98	2.15	1.85
day -4	N	3	3	3	3	3
	P-Value	@0.6761				
MCH	Mean	22.0 a	22.7	22.8	22.5	22.3
[pg]	S.D.	1.59	0.45	1.17	0.72	0.76
day -4	N	3	3	3	3	3
	P-Value	@0.8429				
MCHC	Mean	33.6 k	34.1	34.3	33.8	34.8
[g/dL]	S.D.	1.10	0.40	0.81	0.29	0.15
day -4	N	3	3	3	3	3
	P-Value	@0.1646				
PLT	Mean	339 a	298	341	343	354
[x10e3/µL]	S.D.	33.7	72.5	33.8	46.8	65.0
day -4	N	3	3	3	3	3
	P-Value	@0.7354				
%NEUT	Mean	57.9 a	52.0	62.7	57.0	62.2
[%]	S.D.	3.28	7.65	2.48	2.76	2.09
day -4	N	3	3	3	3	3
	P-Value	@0.0591				
%LYMPH	Mean	33.2 k	34.7	25.9	34.2	29.5
[%]	S.D.	5.17	8.41	0.72	3.63	2.29
day -4	N	3	3	3	3	3
	P-Value	@0.0691				
%MONO	Mean	6.2 a	7.9	7.3	5.9	5.2
[%]	S.D.	1.82	1.28	1.65	1.55	0.59
day -4	N	3	3	3	3	3
	P-Value	@0.2370				

k=KRUSKAL-WALLIS; a=ANOVA

Table F-2b – Summary of Hematology Data – Pre-Test (vs. Group 2)

MALE

		G 2 / M Vehicle	G 1 / M Saline	G 3 / M Low 0.019 mg/L	G 4 / M Mid 0.038 mg/L	G 5 / M High 0.075 mg/L
%EOS	Mean	2.1 a	4.6	3.6	2.3	2.6
[%]	S.D.	0.78	3.56	2.57	0.26	1.19
day -4	N	3	3	3	3	3
	P-Value	@0.5557				
%BASO	Mean	0.3 k	0.4	0.3	0.3	0.3
[%]	S.D.	0.10	0.23	0.00	0.06	0.10
day -4	N	3	3	3	3	3
	P-Value	@0.8796				
#NEUT	Mean	6.23 a	5.38	7.27	5.65	7.40
[x10e3/µL]	S.D.	0.867	1.201	1.002	1.195	0.390
day -4	N	3	3	3	3	3
	P-Value	@0.0956				
#LYMPH	Mean	3.66 k	3.65	3.02	3.45	3.52
[x10e3/µL]	S.D.	1.242	1.298	0.481	1.054	0.292
day -4	N	3	3	3	3	3
	P-Value	@0.8764				
#MONO	Mean	0.67 a	0.83	0.86	0.58	0.63
[x10e3/µL]	S.D.	0.197	0.255	0.274	0.205	0.116
day -4	N	3	3	3	3	3
	P-Value	@0.4925				
#EOS	Mean	0.22 a	0.43	0.45	0.24	0.31
[x10e3/µL]	S.D.	0.046	0.250	0.388	0.078	0.163
day -4	N	3	3	3	3	3
	P-Value	@0.6002				
#BASO	Mean	0.04 k	0.04	0.04	0.04	0.03
[x10e3/µL]	S.D.	0.012	0.015	0.012	0.015	0.012
day -4	N	3	3	3	3	3
	P-Value	@0.9396				
%RETIC	Mean	0.76 a	0.65	0.90	0.83	0.77
[%]	S.D.	0.311	0.090	0.055	0.469	0.147
day -4	N	3	3	3	3	3
	P-Value	@0.8257				
#RETIC	Mean	57.1 a	46.8	62.8	61.1	55.6
[x10e9/L]	S.D.	26.90	6.30	3.75	34.25	12.26
day -4	N	3	3	3	3	3
	P-Value	@0.8850				
%LUC	Mean	0.2 k	0.3	0.2	0.3	0.2
[%]	S.D.	0.12	0.10	0.06	0.12	0.06
day -4	N	3	3	3	3	3
	P-Value	@0.3340				
#LUC	Mean	0.02 k	0.04	0.02	0.03	0.02
[x10e3/µL]	S.D.	0.006	0.012	0.000	0.017	0.006
day -4	N	3	3	3	3	3
	P-Value	@0.1988				

a=ANOVA; k=KRUSKAL-WALLIS

Table F-2b – Summary of Hematology Data – Pre-Test (vs. Group 2)

FEMALE

		G 2 / F Vehicle	G 1 / F Saline	G 3 / F Low 0.019 mg/L	G 4 / F Mid 0.038 mg/L	G 5 / F High 0.075 mg/L
WBC	Mean	11.47 a	12.59	14.62	12.64	12.65
[x10e3/µL]	S.D.	2.174	0.283	6.567	1.926	2.462
day -5	N	3	3	3	3	3
	P-Value	@0.8472				
RBC	Mean	6.83 k	7.52	7.20	7.29	7.59
[x10e6/µL]	S.D.	0.441	0.792	0.745	0.916	0.954
day -5	N	3	3	3	3	3
	P-Value	@0.7541				
HGB	Mean	15.4 a	16.9	15.8	16.8	17.1
[g/dL]	S.D.	0.70	1.36	0.50	1.71	2.14
day -5	N	3	3	3	3	3
	P-Value	@0.5531				
HCT	Mean	45.1 a	48.8	47.1	48.9	49.7
[%]	S.D.	1.95	4.18	2.08	4.28	5.90
day -5	N	3	3	3	3	3
	P-Value	@0.6472				
MCV	Mean	66.2 k	65.1	65.7	67.2	65.5
[fL]	S.D.	2.08	2.60	3.88	2.65	0.46
day -5	N	3	3	3	3	3
	P-Value	@0.7052				
MCH	Mean	22.6 k	22.4	22.1	23.1	22.5
[pg]	S.D.	0.70	0.72	1.71	0.69	0.00
day -5	N	3	3	3	3	3
	P-Value	@0.7687				
MCHC	Mean	34.2 k	34.5	33.6	34.3	34.3
[g/dL]	S.D.	0.06	0.46	0.70	0.46	0.21
day -5	N	3	3	3	3	3
	P-Value	@0.3749				
PLT	Mean	346 k	331	357	335	318
[x10e3/µL]	S.D.	15.8	33.5	49.7	67.6	85.5
day -5	N	3	3	3	3	3
	P-Value	@0.9383				
%NEUT	Mean	66.0 a	62.7	60.9	59.1	60.6
[%]	S.D.	14.01	5.09	8.97	7.08	6.53
day -5	N	3	3	3	3	3
	P-Value	@0.8946				
%LYMPH	Mean	26.4 a	27.6	28.4	31.2	29.4
[%]	S.D.	13.97	4.79	7.46	5.42	6.72
day -5	N	3	3	3	3	3
	P-Value	@0.9621				
%MONO	Mean	5.9 a	7.0	8.0	7.6	6.6
[%]	S.D.	0.93	0.32	0.51	1.35	1.61
day -5	N	3	3	3	3	3
	P-Value	@0.2265				

a=ANOVA; k=KRUSKAL-WALLIS

Table F-2b – Summary of Hematology Data – Pre-Test (vs. Group 2)

FEMALE

		G 2 / F Vehicle	G 1 / F Saline	G 3 / F Low 0.019 mg/L	G 4 / F Mid 0.038 mg/L	G 5 / F High 0.075 mg/L
%EOS	Mean	1.4 a	2.2	2.3	1.4	2.7
[%]	S.D.	0.74	0.35	1.78	0.65	1.80
day -5	N	3	3	3	3	3
	P-Value	@0.6085				
%BASO	Mean	0.2 k	0.3	0.3	0.3	0.4
[%]	S.D.	0.06	0.06	0.06	0.15	0.21
day -5	N	3	3	3	3	3
	P-Value	@0.1863				
#NEUT	Mean	7.77 a	7.88	9.24	7.53	7.74
[x10e3/µL]	S.D.	2.963	0.484	5.499	1.861	2.268
day -5	N	3	3	3	3	3
	P-Value	@0.9598				
#LYMPH	Mean	2.83 a	3.48	3.85	3.89	3.67
[x10e3/µL]	S.D.	0.960	0.672	0.787	0.457	0.829
day -5	N	3	3	3	3	3
	P-Value	@0.4685				
#MONO	Mean	0.68 a	0.87	1.18	0.95	0.83
[x10e3/µL]	S.D.	0.191	0.035	0.558	0.217	0.241
day -5	N	3	3	3	3	3
	P-Value	@0.4074				
#EOS	Mean	0.16 a	0.28	0.30	0.19	0.32
[x10e3/µL]	S.D.	0.090	0.050	0.215	0.091	0.160
day -5	N	3	3	3	3	3
	P-Value	@0.4965				
#BASO	Mean	0.02 k	0.04	0.04	0.04	0.06
[x10e3/µL]	S.D.	0.006	0.006	0.006	0.017	0.035
day -5	N	3	3	3	3	3
	P-Value	@0.1111				
%RETIC	Mean	0.66 k	0.66	1.20	1.09	0.79
[%]	S.D.	0.115	0.079	1.271	0.117	0.165
day -5	N	3	3	3	3	3
	P-Value	@0.3416				
#RETIC	Mean	45.6 k	49.2	82.2	79.7	60.4
[x10e9/L]	S.D.	10.46	3.87	84.53	15.36	16.95
day -5	N	3	3	3	3	3
	P-Value	@0.3299				
%LUC	Mean	0.1 k	0.2	0.2	0.3	0.3
[%]	S.D.	0.15	0.06	0.06	0.20	0.10
day -5	N	3	3	3	3	3
	P-Value	@0.4046				
#LUC	Mean	0.01 k	0.03	0.02	0.04	0.04
[x10e3/µL]	S.D.	0.015	0.010	0.006	0.030	0.020
day -5	N	3	3	3	3	3
	P-Value	@0.3851				

a=ANOVA; k=KRUSKAL-WALLIS

Table F-2c – Summary of Hematology Data – Day 15 (vs. Group 1)

MALE

		G 1 / M Saline	G 2 / M Vehicle	G 3 / M Low 0.019 mg/L	G 4 / M Mid 0.038 mg/L	G 5 / M High 0.075 mg/L
WBC	Mean	7.42 a	7.83	9.41	8.09	9.24
[x10e3/µL]	S.D.	0.616	0.720	2.105	0.495	0.786
day 15	N	3	3	3	3	3
	P-Value	@0.1924				
RBC	Mean	6.53 k	6.98	6.59	6.55	6.81
[x10e6/µL]	S.D.	0.202	0.344	0.386	0.505	0.485
day 15	N	3	3	3	3	3
	P-Value	@0.4923				
HGB	Mean	14.9 k	15.3	14.9	14.8	15.2
[g/dL]	S.D.	0.52	1.27	0.21	1.04	0.95
day 15	N	3	3	3	3	3
	P-Value	@0.8633				
HCT	Mean	45.7 k	47.8	46.2	45.6	46.6
[%]	S.D.	1.45	3.49	1.15	3.00	2.36
day 15	N	3	3	3	3	3
	P-Value	@0.8732				
MCV	Mean	70.0 a	68.5	70.3	69.6	68.5
[fL]	S.D.	1.01	4.50	3.30	1.80	1.49
day 15	N	3	3	3	3	3
	P-Value	@0.8802				
MCH	Mean	22.8 k	21.8	22.6	22.6	22.3
[pg]	S.D.	0.78	1.63	1.25	0.60	0.17
day 15	N	3	3	3	3	3
	P-Value	@0.8168				
MCHC	Mean	32.6 k	31.9	32.2	32.5	31.9
[g/dL]	S.D.	0.64	0.30	0.36	0.23	1.51
day 15	N	3	3	3	3	3
	P-Value	@0.4524				
PLT	Mean	297 a	356	388	374	376
[x10e3/µL]	S.D.	56.0	26.7	22.7	136.2	68.9
day 15	N	3	3	3	3	3
	P-Value	@0.6065				
%NEUT	Mean	54.8 k	51.4	66.0	62.2	63.6
[%]	S.D.	7.01	19.57	7.11	5.23	2.95
day 15	N	3	3	3	3	3
	P-Value	@0.3347				
%LYMPH	Mean	33.6 a	31.2	23.9	30.2	27.8
[%]	S.D.	7.86	1.31	4.55	6.35	3.55
day 15	N	3	3	3	3	3
	P-Value	@0.2786				
%MONO	Mean	8.6 a	5.5	7.8	5.8	5.3
[%]	S.D.	2.20	1.07	2.97	2.06	0.97
day 15	N	3	3	3	3	3
	P-Value	@0.2239				

a=ANOVA; k=KRUSKAL-WALLIS

Table F-2c – Summary of Hematology Data – Day 15 (vs. Group 1)

MALE

		G 1 / M Saline	G 2 / M Vehicle	G 3 / M Low 0.019 mg/L	G 4 / M Mid 0.038 mg/L	G 5 / M High 0.075 mg/L
%EOS	Mean	2.4 a	1.3	1.9	1.4	2.8
[%]	S.D.	1.82	0.32	1.66	0.80	0.60
day 15	N	3	3	3	3	3
	P-Value	@0.5213				
%BASO	Mean	0.2 k	0.2	0.2	0.2	0.2
[%]	S.D.	0.12	0.10	0.00	0.10	0.15
day 15	N	3	3	3	3	3
	P-Value	@0.9593				
#NEUT	Mean	4.06 k	4.81	6.23	5.03	5.89
[x10e3/µL]	S.D.	0.480	0.528	1.605	0.591	0.696
day 15	N	3	3	3	3	3
	P-Value	@0.0812				
#LYMPH	Mean	2.51 a	2.44	2.24	2.43	2.56
[x10e3/µL]	S.D.	0.725	0.197	0.600	0.459	0.345
day 15	N	3	3	3	3	3
	P-Value	@0.9427				
#MONO	Mean	0.65 a	0.43	0.70	0.47	0.48
[x10e3/µL]	S.D.	0.202	0.076	0.153	0.188	0.078
day 15	N	3	3	3	3	3
	P-Value	@0.1879				
#EOS	Mean	0.17 a	0.11	0.20	0.11	0.26
[x10e3/µL]	S.D.	0.114	0.038	0.217	0.071	0.055
day 15	N	3	3	3	3	3
	P-Value	@0.5286				
#BASO	Mean	0.01 k	0.02	0.02	0.02	0.02
[x10e3/µL]	S.D.	0.006	0.006	0.010	0.012	0.010
day 15	N	3	3	3	3	3
	P-Value	@0.8442				
%RETIC	Mean	0.80 a	0.78	1.48	0.93	1.03
[%]	S.D.	0.335	0.386	0.468	0.590	0.263
day 15	N	3	3	3	3	3
	P-Value	@0.3159				
#RETIC	Mean	53.0 a	54.1	96.6	59.7	69.5
[x10e9/L]	S.D.	22.89	25.34	25.31	33.21	14.84
day 15	N	3	3	3	3	3
	P-Value	@0.2597				
%LUC	Mean	0.3 k	0.3	0.3	0.3	0.3
[%]	S.D.	0.06	0.10	0.17	0.06	0.06
day 15	N	3	3	3	3	3
	P-Value	@0.7655				
#LUC	Mean	0.03 k	0.02	0.02	0.03	0.03
[x10e3/µL]	S.D.	0.006	0.006	0.015	0.006	0.006
day 15	N	3	3	3	3	3
	P-Value	@0.9194				

a=ANOVA; k=KRUSKAL-WALLIS

Table F-2c – Summary of Hematology Data – Day 15 (vs. Group 1)

FEMALE

		G 1 / F Saline	G 2 / F Vehicle	G 3 / F Low 0.019 mg/L	G 4 / F Mid 0.038 mg/L	G 5 / F High 0.075 mg/L
WBC	Mean	7.70 a	8.03	11.77	9.31	9.15
[x10e3/µL]	S.D.	0.748	2.559	5.184	1.713	0.218
day 15	N	3	3	3	3	3
	P-Value	@0.4343				
RBC	Mean	7.09 k	6.94	7.00	6.94	7.16
[x10e6/µL]	S.D.	0.087	0.163	0.897	0.942	0.287
day 15	N	3	3	3	3	3
	P-Value	@0.7658				
HGB	Mean	15.9 a	15.7	15.3	15.8	16.1
[g/dL]	S.D.	0.56	0.35	1.11	1.53	0.67
day 15	N	3	3	3	3	3
	P-Value	@0.9004				
HCT	Mean	48.8 a	48.7	48.4	48.9	49.5
[%]	S.D.	1.90	1.23	3.48	5.31	1.70
day 15	N	3	3	3	3	3
	P-Value	@0.9939				
MCV	Mean	68.9 a	70.3	69.4	70.7	69.2
[fL]	S.D.	2.03	0.85	3.76	1.87	0.71
day 15	N	3	3	3	3	3
	P-Value	@0.8136				
MCH	Mean	22.4 k	22.7	22.0	22.9	22.4
[pg]	S.D.	0.60	0.36	1.29	0.87	0.17
day 15	N	3	3	3	3	3
	P-Value	@0.7140				
MCHC	Mean	32.6 k	32.3	31.7	32.4	32.4
[g/dL]	S.D.	0.20	0.15	0.36	0.40	0.53
day 15	N	3	3	3	3	3
	P-Value	@0.1627				
PLT	Mean	345 a	381	354	347	380
[x10e3/µL]	S.D.	70.8	59.8	69.6	73.5	83.5
day 15	N	3	3	3	3	3
	P-Value	@0.9407				
%NEUT	Mean	55.8 a	63.8	56.4	58.5	59.5
[%]	S.D.	4.45	9.90	14.55	5.66	1.63
day 15	N	3	3	3	3	3
	P-Value	@0.7922				
%LYMPH	Mean	35.8 a	28.6	35.1	33.9	31.9
[%]	S.D.	6.00	9.65	12.78	3.65	2.70
day 15	N	3	3	3	3	3
	P-Value	@0.8017				
%MONO	Mean	6.1 a	5.5	6.4	5.5	5.3
[%]	S.D.	1.85	1.37	1.13	1.11	1.04
day 15	N	3	3	3	3	3
	P-Value	@0.8217				

a=ANOVA; k=KRUSKAL-WALLIS

Table F-2c – Summary of Hematology Data – Day 15 (vs. Group 1)

FEMALE

		G 1 / F Saline	G 2 / F Vehicle	G 3 / F Low 0.019 mg/L	G 4 / F Mid 0.038 mg/L	G 5 / F High 0.075 mg/L
%EOS	Mean	1.5 a	1.6	1.6	1.3	2.4
[%]	S.D.	0.51	0.78	1.03	0.79	1.78
day 15	N	3	3	3	3	3
	P-Value	@0.7714				
%BASO	Mean	0.4 k	0.1	0.3	0.3	0.5
[%]	S.D.	0.06	0.06	0.06	0.17	0.17
day 15	N	3	3	3	3	3
	P-Value	@0.0901				
#NEUT	Mean	4.29 a	5.24	7.03	5.49	5.44
[x10e3/µL]	S.D.	0.366	2.357	4.824	1.435	0.279
day 15	N	3	3	3	3	3
	P-Value	@0.7577				
#LYMPH	Mean	2.78 k	2.21	3.75	3.13	2.92
[x10e3/µL]	S.D.	0.711	0.637	1.027	0.505	0.181
day 15	N	3	3	3	3	3
	P-Value	@0.1977				
#MONO	Mean	0.46 a	0.43	0.75	0.50	0.49
[x10e3/µL]	S.D.	0.102	0.081	0.308	0.078	0.104
day 15	N	3	3	3	3	3
	P-Value	@0.1865				
#EOS	Mean	0.11 k	0.11	0.17	0.11	0.22
[x10e3/µL]	S.D.	0.031	0.038	0.108	0.058	0.158
day 15	N	3	3	3	3	3
	P-Value	@0.8299				
#BASO	Mean	0.03 k	0.01	0.03	0.03	0.04
[x10e3/µL]	S.D.	0.006	0.000	0.010	0.015	0.021
day 15	N	3	3	3	3	3
	P-Value	@0.1276				
%RETIC	Mean	0.96 a	1.23	1.20	1.66	1.25
[%]	S.D.	0.320	0.442	0.478	0.463	0.407
day 15	N	3	3	3	3	3
	P-Value	@0.4246				
#RETIC	Mean	67.8 a	85.0	81.7	113.9	89.0
[x10e9/L]	S.D.	23.57	29.57	26.02	27.58	27.45
day 15	N	3	3	3	3	3
	P-Value	@0.3806				
%LUC	Mean	0.4 k	0.3	0.4	0.6	0.5
[%]	S.D.	0.12	0.10	0.12	0.29	0.32
day 15	N	3	3	3	3	3
	P-Value	@0.5484				
#LUC	Mean	0.03 k	0.08	0.04	0.05	0.04
[x10e3/µL]	S.D.	0.010	0.101	0.017	0.026	0.026
day 15	N	3	3	3	3	3
	P-Value	@0.8942				

a=ANOVA; k=KRUSKAL-WALLIS

Table F-2d – Summary of Hematology Data – Day 15 (vs. Group 2)

MALE

		G 2 / M Vehicle		G 1 / M Saline	G 3 / M Low 0.019 mg/L	G 4 / M Mid 0.038 mg/L	G 5 / M High 0.075 mg/L
WBC	Mean	7.83	a	7.42	9.41	8.09	9.24
[x10e3/µL]	S.D.	0.720		0.616	2.105	0.495	0.786
day 15	N	3		3	3	3	3
	P-Value	@0.1924					
RBC	Mean	6.98	k	6.53	6.59	6.55	6.81
[x10e6/µL]	S.D.	0.344		0.202	0.386	0.505	0.485
day 15	N	3		3	3	3	3
	P-Value	@0.4923					
HGB	Mean	15.3	k	14.9	14.9	14.8	15.2
[g/dL]	S.D.	1.27		0.52	0.21	1.04	0.95
day 15	N	3		3	3	3	3
	P-Value	@0.8633					
HCT	Mean	47.8	k	45.7	46.2	45.6	46.6
[%]	S.D.	3.49		1.45	1.15	3.00	2.36
day 15	N	3		3	3	3	3
	P-Value	@0.8732					
MCV	Mean	68.5	a	70.0	70.3	69.6	68.5
[fL]	S.D.	4.50		1.01	3.30	1.80	1.49
day 15	N	3		3	3	3	3
	P-Value	@0.8802					
MCH	Mean	21.8	k	22.8	22.6	22.6	22.3
[pg]	S.D.	1.63		0.78	1.25	0.60	0.17
day 15	N	3		3	3	3	3
	P-Value	@0.8168					
MCHC	Mean	31.9	k	32.6	32.2	32.5	31.9
[g/dL]	S.D.	0.30		0.64	0.36	0.23	1.51
day 15	N	3		3	3	3	3
	P-Value	@0.4524					
PLT	Mean	356	a	297	388	374	376
[x10e3/µL]	S.D.	26.7		56.0	22.7	136.2	68.9
day 15	N	3		3	3	3	3
	P-Value	@0.6065					
%NEUT	Mean	51.4	k	54.8	66.0	62.2	63.6
[%]	S.D.	19.57		7.01	7.11	5.23	2.95
day 15	N	3		3	3	3	3
	P-Value	@0.3347					
%LYMPH	Mean	31.2	a	33.6	23.9	30.2	27.8
[%]	S.D.	1.31		7.86	4.55	6.35	3.55
day 15	N	3		3	3	3	3
	P-Value	@0.2786					
%MONO	Mean	5.5	a	8.6	7.8	5.8	5.3
[%]	S.D.	1.07		2.20	2.97	2.06	0.97
day 15	N	3		3	3	3	3
	P-Value	@0.2239					

a=ANOVA; k=KRUSKAL-WALLIS

Table F-2d – Summary of Hematology Data – Day 15 (vs. Group 2)

MALE

		G 2 / M Vehicle		G 1 / M Saline	G 3 / M Low 0.019 mg/L	G 4 / M Mid 0.038 mg/L	G 5 / M High 0.075 mg/L
%EOS	Mean	1.3	a	2.4	1.9	1.4	2.8
[%]	S.D.	0.32		1.82	1.66	0.80	0.60
day 15	N	3		3	3	3	3
	P-Value	@0.5213					
%BASO	Mean	0.2	k	0.2	0.2	0.2	0.2
[%]	S.D.	0.10		0.12	0.00	0.10	0.15
day 15	N	3		3	3	3	3
	P-Value	@0.9593					
#NEUT	Mean	4.81	k	4.06	6.23	5.03	5.89
[x10e3/µL]	S.D.	0.528		0.480	1.605	0.591	0.696
day 15	N	3		3	3	3	3
	P-Value	@0.0812					
#LYMPH	Mean	2.44	a	2.51	2.24	2.43	2.56
[x10e3/µL]	S.D.	0.197		0.725	0.600	0.459	0.345
day 15	N	3		3	3	3	3
	P-Value	@0.9427					
#MONO	Mean	0.43	a	0.65	0.70	0.47	0.48
[x10e3/µL]	S.D.	0.076		0.202	0.153	0.188	0.078
day 15	N	3		3	3	3	3
	P-Value	@0.1879					
#EOS	Mean	0.11	a	0.17	0.20	0.11	0.26
[x10e3/µL]	S.D.	0.038		0.114	0.217	0.071	0.055
day 15	N	3		3	3	3	3
	P-Value	@0.5286					
#BASO	Mean	0.02	k	0.01	0.02	0.02	0.02
[x10e3/µL]	S.D.	0.006		0.006	0.010	0.012	0.010
day 15	N	3		3	3	3	3
	P-Value	@0.8442					
%RETIC	Mean	0.78	a	0.80	1.48	0.93	1.03
[%]	S.D.	0.386		0.335	0.468	0.590	0.263
day 15	N	3		3	3	3	3
	P-Value	@0.3159					
#RETIC	Mean	54.1	a	53.0	96.6	59.7	69.5
[x10e9/L]	S.D.	25.34		22.89	25.31	33.21	14.84
day 15	N	3		3	3	3	3
	P-Value	@0.2597					
%LUC	Mean	0.3	k	0.3	0.3	0.3	0.3
[%]	S.D.	0.10		0.06	0.17	0.06	0.06
day 15	N	3		3	3	3	3
	P-Value	@0.7655					
#LUC	Mean	0.02	k	0.03	0.02	0.03	0.03
[x10e3/µL]	S.D.	0.006		0.006	0.015	0.006	0.006
day 15	N	3		3	3	3	3
	P-Value	@0.9194					

a=ANOVA; k=KRUSKAL-WALLIS

Table F-2d – Summary of Hematology Data – Day 15 (vs. Group 2)

FEMALE

		G 2 / F Vehicle	G 1 / F Saline	G 3 / F Low 0.019 mg/L	G 4 / F Mid 0.038 mg/L	G 5 / F High 0.075 mg/L
WBC	Mean	8.03 a	7.70	11.77	9.31	9.15
[x10e3/µL]	S.D.	2.559	0.748	5.184	1.713	0.218
day 15	N	3	3	3	3	3
	P-Value	@0.4343				
RBC	Mean	6.94 k	7.09	7.00	6.94	7.16
[x10e6/µL]	S.D.	0.163	0.087	0.897	0.942	0.287
day 15	N	3	3	3	3	3
	P-Value	@0.7658				
HGB	Mean	15.7 a	15.9	15.3	15.8	16.1
[g/dL]	S.D.	0.35	0.56	1.11	1.53	0.67
day 15	N	3	3	3	3	3
	P-Value	@0.9004				
HCT	Mean	48.7 a	48.8	48.4	48.9	49.5
[%]	S.D.	1.23	1.90	3.48	5.31	1.70
day 15	N	3	3	3	3	3
	P-Value	@0.9939				
MCV	Mean	70.3 a	68.9	69.4	70.7	69.2
[fL]	S.D.	0.85	2.03	3.76	1.87	0.71
day 15	N	3	3	3	3	3
	P-Value	@0.8136				
MCH	Mean	22.7 k	22.4	22.0	22.9	22.4
[pg]	S.D.	0.36	0.60	1.29	0.87	0.17
day 15	N	3	3	3	3	3
	P-Value	@0.7140				
MCHC	Mean	32.3 k	32.6	31.7	32.4	32.4
[g/dL]	S.D.	0.15	0.20	0.36	0.40	0.53
day 15	N	3	3	3	3	3
	P-Value	@0.1627				
PLT	Mean	381 a	345	354	347	380
[x10e3/µL]	S.D.	59.8	70.8	69.6	73.5	83.5
day 15	N	3	3	3	3	3
	P-Value	@0.9407				
%NEUT	Mean	63.8 a	55.8	56.4	58.5	59.5
[%]	S.D.	9.90	4.45	14.55	5.66	1.63
day 15	N	3	3	3	3	3
	P-Value	@0.7922				
%LYMPH	Mean	28.6 a	35.8	35.1	33.9	31.9
[%]	S.D.	9.65	6.00	12.78	3.65	2.70
day 15	N	3	3	3	3	3
	P-Value	@0.8017				
%MONO	Mean	5.5 a	6.1	6.4	5.5	5.3
[%]	S.D.	1.37	1.85	1.13	1.11	1.04
day 15	N	3	3	3	3	3
	P-Value	@0.8217				

a=ANOVA; k=KRUSKAL-WALLIS

Table F-2d – Summary of Hematology Data – Day 15 (vs. Group 2)

FEMALE

		G 2 / F Vehicle	G 1 / F Saline	G 3 / F Low 0.019 mg/L	G 4 / F Mid 0.038 mg/L	G 5 / F High 0.075 mg/L
%EOS	Mean	1.6 a	1.5	1.6	1.3	2.4
[%]	S.D.	0.78	0.51	1.03	0.79	1.78
day 15	N	3	3	3	3	3
	P-Value	@0.7714				
%BASO	Mean	0.1 k	0.4	0.3	0.3	0.5
[%]	S.D.	0.06	0.06	0.06	0.17	0.17
day 15	N	3	3	3	3	3
	P-Value	@0.0901				
#NEUT	Mean	5.24 a	4.29	7.03	5.49	5.44
[x10e3/µL]	S.D.	2.357	0.366	4.824	1.435	0.279
day 15	N	3	3	3	3	3
	P-Value	@0.7577				
#LYMPH	Mean	2.21 k	2.78	3.75	3.13	2.92
[x10e3/µL]	S.D.	0.637	0.711	1.027	0.505	0.181
day 15	N	3	3	3	3	3
	P-Value	@0.1977				
#MONO	Mean	0.43 a	0.46	0.75	0.50	0.49
[x10e3/µL]	S.D.	0.081	0.102	0.308	0.078	0.104
day 15	N	3	3	3	3	3
	P-Value	@0.1865				
#EOS	Mean	0.11 k	0.11	0.17	0.11	0.22
[x10e3/µL]	S.D.	0.038	0.031	0.108	0.058	0.158
day 15	N	3	3	3	3	3
	P-Value	@0.8299				
#BASO	Mean	0.01 k	0.03	0.03	0.03	0.04
[x10e3/µL]	S.D.	0.000	0.006	0.010	0.015	0.021
day 15	N	3	3	3	3	3
	P-Value	@0.1276				
%RETIC	Mean	1.23 a	0.96	1.20	1.66	1.25
[%]	S.D.	0.442	0.320	0.478	0.463	0.407
day 15	N	3	3	3	3	3
	P-Value	@0.4246				
#RETIC	Mean	85.0 a	67.8	81.7	113.9	89.0
[x10e9/L]	S.D.	29.57	23.57	26.02	27.58	27.45
day 15	N	3	3	3	3	3
	P-Value	@0.3806				
%LUC	Mean	0.3 k	0.4	0.4	0.6	0.5
[%]	S.D.	0.10	0.12	0.12	0.29	0.32
day 15	N	3	3	3	3	3
	P-Value	@0.5484				
#LUC	Mean	0.08 k	0.03	0.04	0.05	0.04
[x10e3/µL]	S.D.	0.101	0.010	0.017	0.026	0.026
day 15	N	3	3	3	3	3
	P-Value	@0.8942				

a=ANOVA; k=KRUSKAL-WALLIS

Table F-3a – Summary of Coagulation Data – Pre-Test (vs. Group 1)

MALE

		G 1 / M Saline		G 2 / M Vehicle	G 3 / M Low 0.019 mg/L	G 4 / M Mid 0.038 mg/L	G 5 / M High 0.075 mg/L
PT	Mean	8.0	k	8.0	7.9	8.1	8.3
[sec]	S.D.	0.21		0.32	0.15	0.17	0.51
day -4	N	3		3	3	3	3
	P-Value	@0.5536					
APTT	Mean	17.9	k	15.8	15.7	17.3	16.3
[sec]	S.D.	1.18		2.82	0.70	2.00	3.18
day -4	N	3		3	3	3	3
	P-Value	@0.5495					
FIB	Mean	214	a	183	231	287	199
[mg/dL]	S.D.	16.1		33.0	13.0	84.0	21.7
day -4	N	3		3	3	3	3
	P-Value	@0.0976					

k=KRUSKAL-WALLIS; a=ANOVA

FEMALE

		G 1 / F Saline		G 2 / F Vehicle	G 3 / F Low 0.019 mg/L	G 4 / F Mid 0.038 mg/L	G 5 / F High 0.075 mg/L
PT	Mean	8.1	a	9.9	8.6	8.2	8.6
[sec]	S.D.	0.40		3.29	0.38	0.67	0.59
day -5	N	3		3	3	3	3
	P-Value	@0.6556					
APTT	Mean	16.5	a	16.1	16.1	16.7	17.1
[sec]	S.D.	0.85		1.16	1.10	0.10	1.10
day -5	N	3		3	3	3	3
	P-Value	@0.6208					
FIB	Mean	205	a	214	199	231	219
[mg/dL]	S.D.	10.6		18.1	42.5	47.7	66.5
day -5	N	3		3	3	3	3
	P-Value	@0.8971					

a=ANOVA

Table F-3b – Summary of Coagulation Data – Pre-Test (vs. Group 2)

MALE

		G 2 / M Vehicle	G 1 / M Saline	G 3 / M Low 0.019 mg/L	G 4 / M Mid 0.038 mg/L	G 5 / M High 0.075 mg/L
PT	Mean	8.0 k	8.0	7.9	8.1	8.3
[sec]	S.D.	0.32	0.21	0.15	0.17	0.51
day -4	N	3	3	3	3	3
	P-Value	@0.5536				
APTT	Mean	15.8 k	17.9	15.7	17.3	16.3
[sec]	S.D.	2.82	1.18	0.70	2.00	3.18
day -4	N	3	3	3	3	3
	P-Value	@0.5495				
FIB	Mean	183 a	214	231	287	199
[mg/dL]	S.D.	33.0	16.1	13.0	84.0	21.7
day -4	N	3	3	3	3	3
	P-Value	@0.0976				

k=KRUSKAL-WALLIS; a=ANOVA

FEMALE

		G 2 / F Vehicle	G 1 / F Saline	G 3 / F Low 0.019 mg/L	G 4 / F Mid 0.038 mg/L	G 5 / F High 0.075 mg/L
PT	Mean	9.9 a	8.1	8.6	8.2	8.6
[sec]	S.D.	3.29	0.40	0.38	0.67	0.59
day -5	N	3	3	3	3	3
	P-Value	@0.6556				
APTT	Mean	16.1 a	16.5	16.1	16.7	17.1
[sec]	S.D.	1.16	0.85	1.10	0.10	1.10
day -5	N	3	3	3	3	3
	P-Value	@0.6208				
FIB	Mean	214 a	205	199	231	219
[mg/dL]	S.D.	18.1	10.6	42.5	47.7	66.5
day -5	N	3	3	3	3	3
	P-Value	@0.8971				

a=ANOVA

Table F-3c – Summary of Coagulation Data – Day 15 (vs. Group 1)

MALE

		G 1 / M<br>Saline	G 2 / M<br>Vehicle	G 3 / M<br>Low<br>0.019 mg/L	G 4 / M<br>Mid<br>0.038 mg/L	G 5 / M<br>High<br>0.075 mg/L
PT	Mean	8.2 k	8.2	8.0	8.0	8.4
[sec]	S.D.	0.10	0.21	0.23	0.12	0.55
day 15	N	3	3	3	3	3
	P-Value	@0.3232				
APTT	Mean	15.4 a	15.4	14.4	14.4	15.5
[sec]	S.D.	1.23	0.76	0.56	0.44	0.53
day 15	N	3	3	3	3	3
	P-Value	@0.2312				
FIB	Mean	191 k	184	213	256	189
[mg/dL]	S.D.	3.5	37.0	39.6	100.2	27.1
day 15	N	3	3	3	3	3
	P-Value	@0.4387				

k=KRUSKAL-WALLIS; a=ANOVA

FEMALE

		G 1 / F<br>Saline	G 2 / F<br>Vehicle	G 3 / F<br>Low<br>0.019 mg/L	G 4 / F<br>Mid<br>0.038 mg/L	G 5 / F<br>High<br>0.075 mg/L
PT	Mean	8.5 k	9.6	8.7	8.3	8.2
[sec]	S.D.	0.55	2.74	0.59	0.53	0.23
day 15	N	3	3	3	3	3
	P-Value	@0.6298				
APTT	Mean	17.1 a	16.0	15.9	15.0	16.8
[sec]	S.D.	0.95	0.96	0.70	0.87	1.04
day 15	N	3	3	3	3	3
	P-Value	@0.1047				
FIB	Mean	178 a	251	203	164	226
[mg/dL]	S.D.	21.7	34.9	87.1	4.9	81.6
day 15	N	3	3	3	3	3
	P-Value	@0.3804				

k=KRUSKAL-WALLIS; a=ANOVA

Table F-3d – Summary of Coagulation Data – Day 15 (vs. Group 2)

MALE

		G 2 / M Vehicle		G 1 / M Saline	G 3 / M Low 0.019 mg/L	G 4 / M Mid 0.038 mg/L	G 5 / M High 0.075 mg/L
PT	Mean	8.2	k	8.2	8.0	8.0	8.4
[sec]	S.D.	0.21		0.10	0.23	0.12	0.55
day 15	N	3		3	3	3	3
	P-Value	@0.3232					
APTT	Mean	15.4	a	15.4	14.4	14.4	15.5
[sec]	S.D.	0.76		1.23	0.56	0.44	0.53
day 15	N	3		3	3	3	3
	P-Value	@0.2312					
FIB	Mean	184	k	191	213	256	189
[mg/dL]	S.D.	37.0		3.5	39.6	100.2	27.1
day 15	N	3		3	3	3	3
	P-Value	@0.4387					

k=KRUSKAL-WALLIS; a=ANOVA

FEMALE

		G 2 / F Vehicle		G 1 / F Saline	G 3 / F Low 0.019 mg/L	G 4 / F Mid 0.038 mg/L	G 5 / F High 0.075 mg/L
PT	Mean	9.6	k	8.5	8.7	8.3	8.2
[sec]	S.D.	2.74		0.55	0.59	0.53	0.23
day 15	N	3		3	3	3	3
	P-Value	@0.6298					
APTT	Mean	16.0	a	17.1	15.9	15.0	16.8
[sec]	S.D.	0.96		0.95	0.70	0.87	1.04
day 15	N	3		3	3	3	3
	P-Value	@0.1047					
FIB	Mean	251	a	178	203	164	226
[mg/dL]	S.D.	34.9		21.7	87.1	4.9	81.6
day 15	N	3		3	3	3	3
	P-Value	@0.3804					

k=KRUSKAL-WALLIS; a=ANOVA

Table F-4a – Summary of Select Urinalysis Data – Pre-Test (vs. Group 1)
(Refractive Index, SG, pH, Volume)

MALE

		G 1 / M Saline	G 2 / M Vehicle	G 3 / M Low 0.019 mg/L	G 4 / M Mid 0.038 mg/L	G 5 / M High 0.075 mg/L
Refractive Index	Mean	1.3470 k	1.3537	1.3509	1.3388	1.3412
[-]	S.D.	0.01044	0.00058	0.01588	0.00191	0.00838
day 1	N	3	3	3	3	3
	P-Value	@0.1116				
SG	Mean	1.033 k	1.048	1.042	1.014	1.019
[-]	S.D.	0.0252	0.0012	0.0378	0.0047	0.0194
day 1	N	3	3	3	3	3
	P-Value	@0.1116				
pH	Mean	5.7 k	5.3	5.0	6.0	5.5
[-]	S.D.	0.58	0.58	0.00	0.00	0.87
day 1	N	3	3	3	3	3
	P-Value	@0.2688				
Volume	Mean	10.8 k	11.0	11.0	10.8	10.7
[mL]	S.D.	1.26	0.00	0.00	0.29	0.58
day 1	N	3	3	3	3	3
	P-Value	@0.8768				

k=KRUSKAL-WALLIS

FEMALE

		G 1 / F Saline	G 2 / F Vehicle	G 3 / F Low 0.019 mg/L	G 4 / F Mid 0.038 mg/L	G 5 / F High 0.075 mg/L
Refractive Index	Mean	1.3497 a	1.3438	1.3408	1.3457	1.3407
[-]	S.D.	0.00269	0.00434	0.00860	0.00428	0.00221
day 1	N	3	3	3	3	3
	P-Value	@0.2252				
SG	Mean	1.040 a	1.026	1.018	1.030	1.018
[-]	S.D.	0.0062	0.0107	0.0197	0.0096	0.0050
day 1	N	3	3	3	3	3
	P-Value	@0.1892				
pH	Mean	5.7 k	5.3	5.7	6.0	6.0
[-]	S.D.	0.58	0.58	0.58	0.00	0.00
day 1	N	3	3	3	3	3
	P-Value	@0.3480				
Volume	Mean	11.3 k	11.0	10.8	11.0	11.0
[mL]	S.D.	0.58	0.00	0.29	0.00	0.00
day 1	N	3	3	3	3	3
	P-Value	@0.3232				

a=ANOVA; k=KRUSKAL-WALLIS

Table F-4b – Summary of Select Urinalysis Data – Pre-Test (vs. Group 2)
(Refractive Index, SG, pH, Volume)

MALE

		G 2 / M Vehicle	G 1 / M Saline	G 3 / M Low 0.019 mg/L	G 4 / M Mid 0.038 mg/L	G 5 / M High 0.075 mg/L
Refractive Index	Mean	1.3537 k	1.3470	1.3509	1.3388	1.3412
[-]	S.D.	0.00058	0.01044	0.01588	0.00191	0.00838
day 1	N	3	3	3	3	3
	P-Value	@0.1116				
SG	Mean	1.048 k	1.033	1.042	1.014	1.019
[-]	S.D.	0.0012	0.0252	0.0378	0.0047	0.0194
day 1	N	3	3	3	3	3
	P-Value	@0.1116				
pH	Mean	5.3 k	5.7	5.0	6.0	5.5
[-]	S.D.	0.58	0.58	0.00	0.00	0.87
day 1	N	3	3	3	3	3
	P-Value	@0.2688				
Volume	Mean	11.0 k	10.8	11.0	10.8	10.7
[mL]	S.D.	0.00	1.26	0.00	0.29	0.58
day 1	N	3	3	3	3	3
	P-Value	@0.8768				

k=KRUSKAL-WALLIS

FEMALE

		G 2 / F Vehicle	G 1 / F Saline	G 3 / F Low 0.019 mg/L	G 4 / F Mid 0.038 mg/L	G 5 / F High 0.075 mg/L
Refractive Index	Mean	1.3438 a	1.3497	1.3408	1.3457	1.3407
[-]	S.D.	0.00434	0.00269	0.00860	0.00428	0.00221
day 1	N	3	3	3	3	3
	P-Value	@0.2252				
SG	Mean	1.026 a	1.040	1.018	1.030	1.018
[-]	S.D.	0.0107	0.0062	0.0197	0.0096	0.0050
day 1	N	3	3	3	3	3
	P-Value	@0.1892				
pH	Mean	5.3 k	5.7	5.7	6.0	6.0
[-]	S.D.	0.58	0.58	0.58	0.00	0.00
day 1	N	3	3	3	3	3
	P-Value	@0.3480				
Volume	Mean	11.0 k	11.3	10.8	11.0	11.0
[mL]	S.D.	0.00	0.58	0.29	0.00	0.00
day 1	N	3	3	3	3	3
	P-Value	@0.3232				

a=ANOVA; k=KRUSKAL-WALLIS

Table F-4c – Summary of Select Urinalysis Data– Day 15 (vs. Group 1)
(Refractive Index, SG, pH, Volume)

MALE

		G 1 / M Saline	G 2 / M Vehicle	G 3 / M Low 0.019 mg/L	G 4 / M Mid 0.038 mg/L	G 5 / M High 0.075 mg/L
Refractive Index	Mean	1.3519 a	1.3507	1.3491	1.3414	1.3475
[-]	S.D.	0.00110	0.00083	0.00467	0.00624	0.00393
day 15	N	3	3	3	3	3
	P-Value	@0.0610				
SG	Mean	1.045 k	1.042	1.038	1.020	1.034
[-]	S.D.	0.0025	0.0029	0.0107	0.0156	0.0093
day 15	N	3	3	3	3	3
	P-Value	@0.0624				
pH	Mean	6.3 k	6.0	7.0	6.7	6.0
[-]	S.D.	1.53	0.00	1.00	0.58	0.00
day 15	N	3	3	3	3	3
	P-Value	@0.4058				
Volume	Mean	3.7 a	6.2	8.5	7.7	4.7
[mL]	S.D.	2.84	4.37	3.12	2.08	1.61
day 15	N	3	3	3	3	3
	P-Value	@0.3070				

a=ANOVA; k=KRUSKAL-WALLIS

FEMALE

		G 1 / F Saline	G 2 / F Vehicle	G 3 / F Low 0.019 mg/L	G 4 / F Mid 0.038 mg/L	G 5 / F High 0.075 mg/L
Refractive Index	Mean	1.3502 k	1.3460	1.3455	1.3475	1.3496
[-]	S.D.	0.00203	0.00310	0.00383	0.00439	0.00849
day 15	N	3	3	3	3	3
	P-Value	@0.5133				
SG	Mean	1.041 k	1.030	1.029	1.034	1.039
[-]	S.D.	0.0051	0.0075	0.0090	0.0104	0.0202
day 15	N	3	3	3	3	3
	P-Value	@0.5114				
pH	Mean	6.5 k	5.7	6.5	5.8	6.5 X
[-]	S.D.	0.50	0.58	0.50	0.76	0.71
day 15	N	3	3	3	3	2
	P-Value	@0.2321				
Volume	Mean	2.4 k	1.7	4.2	5.0	0.8
[mL]	S.D.	1.94	1.04	3.33	0.87	1.06
day 15	N	3	3	3	3	3
	P-Value	@0.0994				

k=KRUSKAL-WALLIS; X = Group excluded from statistics

NOTE: Group 5 female data for pH at Day 15 were excluded from analysis for statistical significance due to the low number of results (N=2).

Table F-4d – Summary of Select Urinalysis Data– Day 15 (vs. Group 2)
(Refractive Index, SG, pH, Volume)

MALE

		G 2 / M Vehicle	G 1 / M Saline	G 3 / M Low 0.019 mg/L	G 4 / M Mid 0.038 mg/L	G 5 / M High 0.075 mg/L
Refractive Index	Mean	1.3507 a	1.3519	1.3491	1.3414	1.3475
[-]	S.D.	0.00083	0.00110	0.00467	0.00624	0.00393
day 15	N	3	3	3	3	3
	P-Value	@0.0610				
SG	Mean	1.042 k	1.045	1.038	1.020	1.034
[-]	S.D.	0.0029	0.0025	0.0107	0.0156	0.0093
day 15	N	3	3	3	3	3
	P-Value	@0.0624				
pH	Mean	6.0 k	6.3	7.0	6.7	6.0
[-]	S.D.	0.00	1.53	1.00	0.58	0.00
day 15	N	3	3	3	3	3
	P-Value	@0.4058				
Volume	Mean	6.2 a	3.7	8.5	7.7	4.7
[mL]	S.D.	4.37	2.84	3.12	2.08	1.61
day 15	N	3	3	3	3	3
	P-Value	@0.3070				

a=ANOVA; k=KRUSKAL-WALLIS

FEMALE

		G 2 / F Vehicle	G 1 / F Saline	G 3 / F Low 0.019 mg/L	G 4 / F Mid 0.038 mg/L	G 5 / F High 0.075 mg/L
Refractive Index	Mean	1.3460 k	1.3502	1.3455	1.3475	1.3496
[-]	S.D.	0.00310	0.00203	0.00383	0.00439	0.00849
day 15	N	3	3	3	3	3
	P-Value	@0.5133				
SG	Mean	1.030 k	1.041	1.029	1.034	1.039
[-]	S.D.	0.0075	0.0051	0.0090	0.0104	0.0202
day 15	N	3	3	3	3	3
	P-Value	@0.5114				
pH	Mean	5.7 k	6.5	6.5	5.8	6.5 X
[-]	S.D.	0.58	0.50	0.50	0.76	0.71
day 15	N	3	3	3	3	2
	P-Value	@0.2321				
Volume	Mean	1.7 k	2.4	4.2	5.0	0.8
[mL]	S.D.	1.04	1.94	3.33	0.87	1.06
day 15	N	3	3	3	3	3
	P-Value	@0.0994				

k=KRUSKAL-WALLIS; X = Group excluded from statistics

NOTE: Group 5 female data for pH at Day 15 were excluded from analysis for statistical significance due to the low number of results (N=2).

Table F-5 – Individual Animal Clinical Chemistry Data

PRE-TEST (MALE)

Dose Group	Animal Number	NA [mmol/L] day -4	K [mmol/L] day -4	CL [mmol/L] day -4	CALC [mg/dL] day -4	CRE [mg/dL] day -4	PO4 [mg/dL] day -4	TP [g/dL] day -4
G 1 / M Saline	1EZF	140	4.2	106	10.5	0.76	7.3	5.4
	1FUH	113	3.8	86	11.8	0.85	7.3	6.6
	1UYF	141	4.0	108	10.2	0.66	7.5	5.5
	Mean	131	4.0	100	10.8	0.76	7.4	5.8
	S.D.	15.9	0.20	12.2	0.85	0.095	0.12	0.67
	N	3	3	3	3	3	3	3
G 2 / M Vehicle	2AGF	144	4.9	111	10.7	0.89	8.1	6.0
	2BSF	104	3.6	83	11.2	0.65	7.6	6.4
	2FRH	143	4.0	108	10.9	0.79	7.0	5.6
	Mean	130	4.2	101	10.9	0.78	7.6	6.0
	S.D.	22.8	0.67	15.4	0.25	0.121	0.55	0.40
	N	3	3	3	3	3	3	3
G 3 / M Low 0.019 mg/L	3DWH	145	4.0	111	10.3	0.68	6.0	6.2
	3THF	133	3.9	101	10.8	0.69	6.6	6.2
	3TYF	141	4.3	109	10.7	0.74	6.9	5.9
	Mean	140	4.1	107	10.6	0.70	6.5	6.1
	S.D.	6.1	0.21	5.3	0.26	0.032	0.46	0.17
	N	3	3	3	3	3	3	3
G 4 / M Mid 0.038 mg/L	4EAH	143	4.2	107	10.6	0.77	6.0	5.8
	4JLH	120	3.9	92	11.5	0.69	8.0	6.5
	4YJF	147	4.1	108	10.9	0.75	6.3	6.0
	Mean	137	4.1	102	11.0	0.74	6.8	6.1
	S.D.	14.6	0.15	9.0	0.46	0.042	1.08	0.36
	N	3	3	3	3	3	3	3
G 5 / M High 0.075 mg/L	5GPH	138	3.7	100	11.0	0.82	7.2	6.2
	5ZOF	143	4.5	108	10.9	0.83	6.8	6.5
	5ZTF	150	4.4	113	10.3	0.78	6.3	5.7
	Mean	144	4.2	107	10.7	0.81	6.8	6.1
	S.D.	6.0	0.44	6.6	0.38	0.026	0.45	0.40
	N	3	3	3	3	3	3	3

Table F-5 – Individual Animal Clinical Chemistry Data

PRE-TEST (MALE)

Dose Group	Animal Number	ALB [g/dL] day -4	GLOB [g/dL] day -4	A/G [-] day -4	BUN [mg/dL] day -4	BUN/CRE [Ratio] day -4	ALP [IU/L] day -4	ALT [IU/L] day -4
G 1 / M Saline	1EZF	3.0	2.4	1.2	12	15.79	86	34
	1FUH	3.3	3.3	1.0	15	17.65	74	29
	1UYF	2.9	2.6	1.1	9	13.64	81	26
	Mean	3.1	2.8	1.1	12	15.69	80	30
	S.D.	0.21	0.47	0.13	3.0	2.007	6.0	4.0
	N	3	3	3	3	3	3	3
G 2 / M Vehicle	2AGF	3.4	2.6	1.3	22	24.72	105	28
	2BSF	3.2	3.2	1.0	10	15.38	47	35
	2FRH	3.1	2.5	1.2	12	15.19	78	40
	Mean	3.2	2.8	1.2	15	18.43	77	34
	S.D.	0.15	0.38	0.16	6.4	5.446	29.0	6.0
	N	3	3	3	3	3	3	3
G 3 / M Low 0.019 mg/L	3DWH	3.0	3.2	0.9	13	19.12	84	38
	3THF	3.3	2.9	1.1	14	20.29	79	35
	3TYF	2.9	3.0	1.0	16	21.62	98	61
	Mean	3.1	3.0	1.0	14	20.34	87	45
	S.D.	0.21	0.15	0.11	1.5	1.253	9.8	14.2
	N	3	3	3	3	3	3	3
G 4 / M Mid 0.038 mg/L	4EAH	3.3	2.5	1.3	13	16.88	58	45
	4JLH	3.5	3.0	1.2	11	15.94	70	31
	4YJF	3.2	2.8	1.1	12	16.00	58	27
	Mean	3.3	2.8	1.2	12	16.28	62	34
	S.D.	0.15	0.25	0.10	1.0	0.527	6.9	9.5
	N	3	3	3	3	3	3	3
G 5 / M High 0.075 mg/L	5GPH	3.4	2.8	1.2	16	19.51	68	44
	5ZOF	3.0	3.5	0.9	13	15.66	114	40
	5ZTF	2.9	2.8	1.0	14	17.95	78	31
	Mean	3.1	3.0	1.0	14	17.71	87	38
	S.D.	0.26	0.40	0.18	1.5	1.936	24.2	6.7
	N	3	3	3	3	3	3	3

Table F-5 – Individual Animal Clinical Chemistry Data

PRE-TEST (MALE)

Dose Group	Animal Number	AST [IU/L] day -4	CK [U/L] day -4	TBIL [mg/dL] day -4	CHOL [mg/dL] day -4	TG [mg/dL] day -4	GLU [mg/dL] day -4
G 1 / M Saline	1EZF	28	158	0.1	137	41	103
	1FUH	34	295	0.1	159	60	106
	1UYF	23	129	0.1	113	18	122
	Mean	28	194	0.1	136	40	110
	S.D.	5.5	88.7	0.00	23.0	21.0	10.2
	N	3	3	3	3	3	3
G 2 / M Vehicle	2AGF	32	162	0.1	120	70	107
	2BSF	21	155	0.1	120	32	134
	2FRH	25	138	0.1	119	23	117
	Mean	26	152	0.1	120	42	119
	S.D.	5.6	12.3	0.00	0.6	24.9	13.7
	N	3	3	3	3	3	3
G 3 / M Low 0.019 mg/L	3DWH	23	141	0.1	120	26	109
	3THF	25	179	0.1	119	23	134
	3TYF	36	221	0.1	100	34	115
	Mean	28	180	0.1	113	28	119
	S.D.	7.0	40.0	0.00	11.3	5.7	13.1
	N	3	3	3	3	3	3
G 4 / M Mid 0.038 mg/L	4EAH	30	197	0.1	131	24	115
	4JLH	31	180	0.1	151	36	112
	4YJF	23	107	0.1	139	23	112
	Mean	28	161	0.1	140	28	113
	S.D.	4.4	47.8	0.00	10.1	7.2	1.7
	N	3	3	3	3	3	3
G 5 / M High 0.075 mg/L	5GPH	31	209	0.2	124	25	112
	5ZOF	28	171	0.1	177	48	115
	5ZTF	36	242	0.1	121	28	119
	Mean	32	207	0.1	141	34	115
	S.D.	4.0	35.5	0.06	31.5	12.5	3.5
	N	3	3	3	3	3	3

Table F-5 – Individual Animal Clinical Chemistry Data

PRE-TEST (FEMALE)

Dose Group	Animal Number	NA [mmol/L] day -5	K [mmol/L] day -5	CL [mmol/L] day -5	CALC [mg/dL] day -5	CRE [mg/dL] day -5	PO4 [mg/dL] day -5	TP [g/dL] day -5
G 1 / F Saline	1CBE	111	3.4	83	11.5	0.83	6.8	6.5
	1JKG	143	4.0	103	11.4	0.93	8.5	6.0
	1JSG	150	4.6	112	10.7	0.56	7.0	5.6
	Mean	135	4.0	99	11.2	0.77	7.4	6.0
	S.D.	20.8	0.60	14.8	0.44	0.191	0.93	0.45
	N	3	3	3	3	3	3	3
G 2 / F Vehicle	2AJE	139	4.4	105	10.2	1.07	7.2	6.0
	2ZQE	104	3.2	80	11.4	0.90	6.9	6.6
	2FLE	140	4.1	106	10.7	0.83	7.5	5.3
	Mean	128	3.9	97	10.8	0.93	7.2	6.0
	S.D.	20.5	0.62	14.7	0.60	0.123	0.30	0.65
	N	3	3	3	3	3	3	3
G 3 / F Low 0.019 mg/L	3CVG	136	4.8	108	10.5	0.54	6.7	5.1
	3FKE	134	4.2	103	10.8	0.72	6.7	5.5
	3ZRE	134	4.4	102	10.2	0.98	7.8	6.0
	Mean	135	4.5	104	10.5	0.75	7.1	5.5
	S.D.	1.2	0.31	3.2	0.30	0.221	0.64	0.45
	N	3	3	3	3	3	3	3
G 4 / F Mid 0.038 mg/L	4ACE	154	4.0	115	10.3	0.75	5.9	6.0
	4CAE	150	4.3	108	10.8	0.84	5.7	6.1
	4EVE	146	4.0	109	10.3	0.77	6.7	5.5
	Mean	150	4.1	111	10.5	0.79	6.1	5.9
	S.D.	4.0	0.17	3.8	0.29	0.047	0.53	0.32
	N	3	3	3	3	3	3	3
G 5 / F High 0.075 mg/L	5AIE	150	4.5	110	10.9	0.99	6.1	5.7
	5CGE	143	4.5	107	10.6	0.72	6.8	5.6
	5CSG	109	3.3	83	11.2	0.73	7.4	6.2
	Mean	134	4.1	100	10.9	0.81	6.8	5.8
	S.D.	21.9	0.69	14.8	0.30	0.153	0.65	0.32
	N	3	3	3	3	3	3	3

Table F-5 – Individual Animal Clinical Chemistry Data

PRE-TEST (FEMALE)

Dose Group	Animal Number	ALB [g/dL] day -5	GLOB [g/dL] day -5	A/G [-] day -5	BUN [mg/dL] day -5	BUN/CRE [-] day -5	ALP [IU/L] day -5	ALT [IU/L] day -5
G 1 / F Saline	1CBE	3.3	3.2	1.0	18	21.69	58	24
	1JKG	3.3	2.7	1.2	14	15.05	64	43
	1JSG	3.0	2.6	1.2	17	30.36	95	41
	Mean	3.2	2.8	1.1	16	22.37	72	36
	S.D.	0.17	0.32	0.10	2.1	7.674	19.9	10.4
	N	3	3	3	3	3	3	3
G 2 / F Vehicle	2AJE	2.9	3.1	0.9	19	17.76	82	42
	2ZQE	3.1	3.5	0.9	16	17.78	51	32
	2FLE	3.1	2.2	1.4	17	20.48	88	27
	Mean	3.0	2.9	1.1	17	18.67	74	34
	S.D.	0.12	0.67	0.29	1.5	1.567	19.9	7.6
	N	3	3	3	3	3	3	3
G 3 / F Low 0.019 mg/L	3CVG	2.9	2.2	1.3	9	16.67	111	31
	3FKE	3.0	2.5	1.2	14	19.44	85	45
	3ZRE	3.0	3.0	1.0	22	22.45	56	34
	Mean	3.0	2.6	1.2	15	19.52	84	37
	S.D.	0.06	0.40	0.16	6.6	2.892	27.5	7.4
	N	3	3	3	3	3	3	3
G 4 / F Mid 0.038 mg/L	4ACE	2.9	3.1	0.9	18	24.00	106	39
	4CAE	3.3	2.8	1.2	14	16.67	107	40
	4EVE	3.0	2.5	1.2	12	15.58	86	25
	Mean	3.1	2.8	1.1	15	18.75	100	35
	S.D.	0.21	0.30	0.15	3.1	4.578	11.8	8.4
	N	3	3	3	3	3	3	3
G 5 / F High 0.075 mg/L	5AIE	3.3	2.4	1.4	16	16.16	101	37
	5CGE	3.0	2.6	1.2	17	23.61	91	34
	5CSG	3.0	3.2	0.9	16	21.92	76	22
	Mean	3.1	2.7	1.2	16	20.56	89	31
	S.D.	0.17	0.42	0.22	0.6	3.905	12.6	7.9
	N	3	3	3	3	3	3	3

Table F-5 – Individual Animal Clinical Chemistry Data

PRE-TEST (FEMALE)

Dose Group	Animal Number	AST [IU/L] day -5	CK [U/L] day -5	TBIL [mg/dL] day -5	CHOL [mg/dL] day -5	TG [mg/dL] day -5	GLU [mg/dL] day -5
G 1 / F Saline	1CBE	28	118	0.1	120	27	127
	1JKG	50	621	0.1	140	28	109
	1JSG	31	236	0.1	101	31	104
	Mean	36	325	0.1	120	29	113
	S.D.	11.9	263.0	0.00	19.5	2.1	12.1
	N	3	3	3	3	3	3
G 2 / F Vehicle	2AJE	32	217	0.1	142	26	94
	2ZQE	30	204	0.1	136	39	115
	2FLE	24	188	0.1	125	26	107
	Mean	29	203	0.1	134	30	105
	S.D.	4.2	14.5	0.00	8.6	7.5	10.6
	N	3	3	3	3	3	3
G 3 / F Low 0.019 mg/L	3CVG	35	327	0.1	91	56	87
	3FKE	34	223	0.1	119	37	108
	3ZRE	33	177	0.1	118	26	99
	Mean	34	242	0.1	109	40	98
	S.D.	1.0	76.8	0.00	15.9	15.2	10.5
	N	3	3	3	3	3	3
G 4 / F Mid 0.038 mg/L	4ACE	39	211	0.1	114	25	98
	4CAE	29	118	0.1	143	30	95
	4EVE	29	181	0.1	126	41	111
	Mean	32	170	0.1	128	32	101
	S.D.	5.8	47.5	0.00	14.6	8.2	8.5
	N	3	3	3	3	3	3
G 5 / F High 0.075 mg/L	5AIE	39	132	0.1	121	27	88
	5CGE	35	207	0.1	103	23	106
	5CSG	23	145	0.1	143	37	109
	Mean	32	161	0.1	122	29	101
	S.D.	8.3	40.1	0.00	20.0	7.2	11.4
	N	3	3	3	3	3	3

Table F-5 – Individual Animal Clinical Chemistry Data

DAY 15 (MALE)

Dose Group	Animal Number	NA [mmol/L] day 15	K [mmol/L] day 15	CL [mmol/L] day 15	CALC [mg/dL] day 15	CRE [mg/dL] day 15	PO4 [mg/dL] day 15	TP [g/dL] day 15
G 1 / M Saline	1EZF	145	4.1	110	10.4	0.74	6.1	5.4
	1FUH	143	4.4	109	10.5	0.66	5.9	5.6
	1UYF	145	4.4	109	10.3	0.65	6.5	5.5
	Mean	144	4.3	109	10.4	0.68	6.2	5.5
	S.D.	1.2	0.17	0.6	0.10	0.049	0.31	0.10
	N	3	3	3	3	3	3	3
G 2 / M Vehicle	2AGF	145	4.4	107	10.1	0.62	6.1	6.1
	2BSF	144	4.5	108	10.2	0.51	6.3	5.9
	2FRH	142	4.2	105	10.9	0.77	8.4	5.7
	Mean	144	4.4	107	10.4	0.63	6.9	5.9
	S.D.	1.5	0.15	1.5	0.44	0.131	1.27	0.20
	N	3	3	3	3	3	3	3
G 3 / M Low 0.019 mg/L	3DWH	145	4.1	107	10.2	0.69	5.3	6.0
	3THF	145	4.4	106	10.2	0.57	6.6	5.9
	3TYF	145	4.6	108	10.1	0.60	5.9	6.0
	Mean	145	4.4	107	10.2	0.62	5.9	6.0
	S.D.	0.0	0.25	1.0	0.06	0.062	0.65	0.06
	N	3	3	3	3	3	3	3
G 4 / M Mid 0.038 mg/L	4EAH	139	4.8	104	10.1	0.59	5.3	5.7
	4JLH	144	4.5	107	10.2	0.61	6.3	5.7
	4YJF	140	4.2	104	10.4	0.64	6.5	5.7
	Mean	141	4.5	105	10.2	0.61	6.0	5.7
	S.D.	2.6	0.30	1.7	0.15	0.025	0.64	0.00
	N	3	3	3	3	3	3	3
G 5 / M High 0.075 mg/L	5GPH	144	4.6	106	10.5	0.68	6.8	5.6
	5ZOF	140	4.7	104	10.2	0.72	6.3	6.1
	5ZTF	144	4.1	107	10.2	0.62	6.5	5.9
	Mean	143	4.5	106	10.3	0.67	6.5	5.9
	S.D.	2.3	0.32	1.5	0.17	0.050	0.25	0.25
	N	3	3	3	3	3	3	3

Table F-5 – Individual Animal Clinical Chemistry Data

DAY 15 (MALE)

Dose Group	Animal Number	ALB [g/dL] day 15	GLOB [g/dL] day 15	A/G [-] day 15	BUN [mg/dL] day 15	BUN/CRE [Ratio] day 15	ALP [IU/L] day 15	ALT [IU/L] day 15
G 1 / M Saline	1EZF	3.0	2.4	1.2	13	17.57	65	36
	1FUH	2.9	2.7	1.1	12	18.18	83	40
	1UYF	3.1	2.4	1.3	10	15.38	74	37
	Mean	3.0	2.5	1.2	12	17.04	74	38
	S.D.	0.10	0.17	0.12	1.5	1.470	9.0	2.1
	N	3	3	3	3	3	3	3
G 2 / M Vehicle	2AGF	3.3	2.8	1.2	10	16.13	88	45
	2BSF	3.3	2.6	1.3	10	19.61	57	44
	2FRH	3.1	2.6	1.2	13	16.88	78	44
	Mean	3.2	2.7	1.2	11	17.54	74	44
	S.D.	0.12	0.12	0.05	1.7	1.830	15.8	0.6
	N	3	3	3	3	3	3	3
G 3 / M Low 0.019 mg/L	3DWH	3.0	3.0	1.0	9	13.04	87	58
	3THF	3.4	2.5	1.4	12	21.05	84	43
	3TYF	2.9	3.1	0.9	12	20.00	97	88
	Mean	3.1	2.9	1.1	11	18.03	89	63
	S.D.	0.26	0.32	0.23	1.7	4.352	6.8	22.9
	N	3	3	3	3	3	3	3
G 4 / M Mid 0.038 mg/L	4EAH	3.0	2.7	1.1	12	20.34	68	90
	4JLH	3.2	2.5	1.3	14	22.95	67	35
	4YJF	3.2	2.5	1.3	9	14.06	57	52
	Mean	3.1	2.6	1.2	12	19.12	64	59
	S.D.	0.12	0.12	0.10	2.5	4.568	6.1	28.2
	N	3	3	3	3	3	3	3
G 5 / M High 0.075 mg/L	5GPH	3.1	2.5	1.2	12	17.65	67	57
	5ZOF	2.9	3.2	0.9	13	18.06	84	68
	5ZTF	3.1	2.8	1.1	15	24.19	59	27
	Mean	3.0	2.8	1.1	13	19.97	70	51
	S.D.	0.12	0.35	0.17	1.5	3.667	12.8	21.2
	N	3	3	3	3	3	3	3

Table F-5 – Individual Animal Clinical Chemistry Data

DAY 15 (MALE)

Dose Group	Animal Number	AST [IU/L] day 15	CK [U/L] day 15	TBIL [mg/dL] day 15	CHOL [mg/dL] day 15	TG [mg/dL] day 15	GLU [mg/dL] day 15
G 1 / M Saline	1EZF	24	177	0.1	146	31	112
	1FUH	27	198	0.0	129	41	106
	1UYF	21	172	0.1	118	24	118
	Mean	24	182	0.1	131	32	112
	S.D.	3.0	13.8	0.06	14.1	8.5	6.0
	N	3	3	3	3	3	3
G 2 / M Vehicle	2AGF	32	166	0.1	111	40	96
	2BSF	23	148	0.0	120	35	118
	2FRH	25	151	0.1	130	37	113
	Mean	27	155	0.1	120	37	109
	S.D.	4.7	9.6	0.06	9.5	2.5	11.5
	N	3	3	3	3	3	3
G 3 / M Low 0.019 mg/L	3DWH	24	175	0.1	127	35	114
	3THF	22	181	0.1	125	31	125
	3TYF	42	482	0.1	122	45	125
	Mean	29	279	0.1	125	37	121
	S.D.	11.0	175.5	0.00	2.5	7.2	6.4
	N	3	3	3	3	3	3
G 4 / M Mid 0.038 mg/L	4EAH	37	242	0.0	126	42	134
	4JLH	30	209	0.1	160	40	106
	4YJF	28	199	0.1	150	33	117
	Mean	32	217	0.1	145	38	119
	S.D.	4.7	22.5	0.06	17.5	4.7	14.1
	N	3	3	3	3	3	3
G 5 / M High 0.075 mg/L	5GPH	23	122	0.1	105	32	107
	5ZOF	30	121	0.1	138	41	128
	5ZTF	25	130	0.1	131	49	120
	Mean	26	124	0.1	125	41	118
	S.D.	3.6	4.9	0.00	17.4	8.5	10.6
	N	3	3	3	3	3	3

Table F-5 – Individual Animal Clinical Chemistry Data

DAY 15 (FEMALE)

Dose Group	Animal Number	NA [mmol/L] day 15	K [mmol/L] day 15	CL [mmol/L] day 15	CALC [mg/dL] day 15	CRE [mg/dL] day 15	PO4 [mg/dL] day 15	TP [g/dL] day 15
G 1 / F Saline	1CBE	144	4.6	108	10.5	0.60	5.9	5.8
	1JKG	144	4.5	107	10.7	0.75	6.6	5.5
	1JSG	144	4.7	107	10.7	0.62	6.0	5.7
	Mean	144	4.6	107	10.6	0.66	6.2	5.7
	S.D.	0.0	0.10	0.6	0.12	0.081	0.38	0.15
	N	3	3	3	3	3	3	3
G 2 / F Vehicle	2AJE	145	4.7	110	10.2	0.72	6.5	5.9
	2ZQE	143	4.6	107	10.1	0.73	5.2	6.0
	2FLE	144	4.8	107	10.6	0.72	6.3	5.4
	Mean	144	4.7	108	10.3	0.72	6.0	5.8
	S.D.	1.0	0.10	1.7	0.26	0.006	0.70	0.32
	N	3	3	3	3	3	3	3
G 3 / F Low 0.019 mg/L	3CVG	143	4.5	109	10.1	0.47	6.2	5.2
	3FKE	145	4.4	110	10.2	0.63	6.2	5.4
	3ZRE	146	4.7	109	10.3	0.52	7.0	6.1
	Mean	145	4.5	109	10.2	0.54	6.5	5.6
	S.D.	1.5	0.15	0.6	0.10	0.082	0.46	0.47
	N	3	3	3	3	3	3	3
G 4 / F Mid 0.038 mg/L	4ACE	144	4.1	109	10.6	0.51	7.0	5.9
	4CAE	145	4.6	109	10.6	0.53	6.2	5.9
	4EVE	144	4.7	107	10.5	0.62	7.0	5.7
	Mean	144	4.5	108	10.6	0.55	6.7	5.8
	S.D.	0.6	0.32	1.2	0.06	0.059	0.46	0.12
	N	3	3	3	3	3	3	3
G 5 / F High 0.075 mg/L	5AIE	144	4.3	105	10.5	0.72	5.5	5.6
	5CGE	146	4.8	109	10.1	0.72	6.1	5.7
	5CSG	146	4.4	109	10.2	0.57	5.3	5.9
	Mean	145	4.5	108	10.3	0.67	5.6	5.7
	S.D.	1.2	0.26	2.3	0.21	0.087	0.42	0.15
	N	3	3	3	3	3	3	3

Table F-5 – Individual Animal Clinical Chemistry Data

DAY 15 (FEMALE)

Dose Group	Animal Number	ALB [g/dL] day 15	GLOB [g/dL] day 15	A/G [-] day 15	BUN [mg/dL] day 15	BUN/CRE [-] day 15	ALP [IU/L] day 15	ALT [IU/L] day 15
G 1 / F Saline	1CBE	3.2	2.6	1.2	13	21.67	89	44
	1JKG	3.1	2.4	1.3	13	17.33	63	40
	1JSG	3.4	2.3	1.5	14	22.58	99	34
	Mean	3.2	2.4	1.3	13	20.53	84	39
	S.D.	0.15	0.15	0.13	0.6	2.803	18.6	5.0
	N	3	3	3	3	3	3	3
G 2 / F Vehicle	2AJE	3.0	2.9	1.0	16	22.22	114	63
	2ZQE	3.2	2.8	1.1	11	15.07	61	50
	2FLE	3.2	2.2	1.5	15	20.83	93	34
	Mean	3.1	2.6	1.2	14	19.37	89	49
	S.D.	0.12	0.38	0.22	2.6	3.793	26.7	14.5
	N	3	3	3	3	3	3	3
G 3 / F Low 0.019 mg/L	3CVG	3.0	2.2	1.4	8	17.02	110	28
	3FKE	3.0	2.4	1.2	12	19.05	96	47
	3ZRE	3.0	3.1	1.0	11	21.15	70	33
	Mean	3.0	2.6	1.2	10	19.07	92	36
	S.D.	0.00	0.47	0.20	2.1	2.066	20.3	9.8
	N	3	3	3	3	3	3	3
G 4 / F Mid 0.038 mg/L	4ACE	3.0	2.9	1.0	14	27.45	94	48
	4CAE	3.4	2.5	1.4	9	16.98	99	127
	4EVE	3.0	2.7	1.1	12	19.35	104	34
	Mean	3.1	2.7	1.2	12	21.26	99	70
	S.D.	0.23	0.20	0.17	2.5	5.489	5.0	50.1
	N	3	3	3	3	3	3	3
G 5 / F High 0.075 mg/L	5AIE	3.3	2.3	1.4	13	18.06	76	36
	5CGE	3.1	2.6	1.2	16	22.22	80	32
	5CSG	2.9	3.0	1.0	9	15.79	134	48
	Mean	3.1	2.6	1.2	13	18.69	97	39
	S.D.	0.20	0.35	0.23	3.5	3.263	32.4	8.3
	N	3	3	3	3	3	3	3

Table F-5 – Individual Animal Clinical Chemistry Data

DAY 15 (FEMALE)

Dose Group	Animal Number	AST [IU/L] day 15	CK [U/L] day 15	TBIL [mg/dL] day 15	CHOL [mg/dL] day 15	TG [mg/dL] day 15	GLU [mg/dL] day 15
G 1 / F Saline	1CBE	25	97	0.1	102	21	94
	1JKG	22	121	0.1	130	23	105
	1JSG	22	178	0.1	104	32	107
	Mean	23	132	0.1	112	25	102
	S.D.	1.7	41.6	0.00	15.6	5.9	7.0
	N	3	3	3	3	3	3
G 2 / F Vehicle	2AJE	25	190	0.1	136	43	100
	2ZQE	28	171	0.1	145	51	105
	2FLE	19	183	0.1	135	29	98
	Mean	24	181	0.1	139	41	101
	S.D.	4.6	9.6	0.00	5.5	11.1	3.6
	N	3	3	3	3	3	3
G 3 / F Low 0.019 mg/L	3CVG	23	148	0.0	97	37	114
	3FKE	25	146	0.1	103	26	108
	3ZRE	34	143	0.1	83	31	98
	Mean	27	146	0.1	94	31	107
	S.D.	5.9	2.5	0.06	10.3	5.5	8.1
	N	3	3	3	3	3	3
G 4 / F Mid 0.038 mg/L	4ACE	36	134	0.1	109	22	113
	4CAE	22	99	0.1	143	38	103
	4EVE	28	253	0.1	128	37	100
	Mean	29	162	0.1	127	32	105
	S.D.	7.0	80.7	0.00	17.0	9.0	6.8
	N	3	3	3	3	3	3
G 5 / F High 0.075 mg/L	5AIE	33	96	0.1	115	37	92
	5CGE	23	129	0.1	98	28	113
	5CSG	24	122	0.1	140	37	110
	Mean	27	116	0.1	118	34	105
	S.D.	5.5	17.4	0.00	21.1	5.2	11.4
	N	3	3	3	3	3	3

Table F-6 – Individual Animal Hematology Data

PRE-TEST (MALE)

Dose Group	Animal Number	WBC [x10e3/μL] day -4	RBC [x10e6/μL] day -4	HGB [g/dL] day -4	HCT [%] day -4	MCV [fL] day -4	MCH [pg] day -4	MCHC [g/dL] day -4	PLT [x10e3/μL] day -4
G 1 / M Saline	1EZF	11.43	7.27	16.2	48.2	66.3	22.3	33.6	274
	1FUH	8.31	7.30	16.9	49.3	67.6	23.2	34.3	240
	1UYF	11.38	6.86	15.5	45.3	66.1	22.7	34.3	379
	Mean	10.37	7.14	16.2	47.6	66.7	22.7	34.1	298
	S.D.	1.787	0.246	0.70	2.07	0.81	0.45	0.40	72.5
	N	3	3	3	3	3	3	3	3
G 2 / M Vehicle	2AGF	12.91	7.90	16.7	51.5	65.2	21.1	32.3	375
	2BSF	10.83	7.32	15.3	44.8	61.2	21.0	34.2	335
	2FRH	8.79	6.87	16.3	47.8	69.6	23.8	34.2	308
	Mean	10.84	7.36	16.1	48.0	65.3	22.0	33.6	339
	S.D.	2.060	0.516	0.72	3.36	4.20	1.59	1.10	33.7
	N	3	3	3	3	3	3	3	3
G 3 / M Low 0.019 mg/L	3DWH	13.63	7.19	16.2	48.1	66.9	22.6	33.7	302
	3THF	9.53	6.26	15.1	42.8	68.3	24.1	35.2	359
	3TYF	11.80	7.43	16.2	47.8	64.4	21.8	33.9	362
	Mean	11.65	6.96	15.8	46.2	66.5	22.8	34.3	341
	S.D.	2.054	0.618	0.64	2.98	1.98	1.17	0.81	33.8
	N	3	3	3	3	3	3	3	3
G 4 / M Mid 0.038 mg/L	4EAH	7.22	6.76	15.3	45.0	66.5	22.7	34.1	358
	4JLH	11.24	7.62	17.6	52.5	68.9	23.1	33.6	381
	4YJF	11.49	8.05	17.5	52.0	64.6	21.7	33.6	291
	Mean	9.98	7.48	16.8	49.8	66.7	22.5	33.8	343
	S.D.	2.396	0.657	1.30	4.19	2.15	0.72	0.29	46.8
	N	3	3	3	3	3	3	3	3
G 5 / M High 0.075 mg/L	5GPH	11.45	7.71	16.9	48.5	62.8	21.8	34.8	288
	5ZOF	11.45	6.45	15.0	42.8	66.3	23.2	35.0	418
	5ZTF	12.80	7.49	16.5	47.5	63.5	22.0	34.7	355
	Mean	11.90	7.22	16.1	46.3	64.2	22.3	34.8	354
	S.D.	0.779	0.673	1.00	3.04	1.85	0.76	0.15	65.0
	N	3	3	3	3	3	3	3	3

Table F-6 – Individual Animal Hematology Data

PRE-TEST (MALE)

Dose Group	Animal Number	%NEUT [%] day -4	%LYMPH [%] day -4	%MONO [%] day -4	%EOS [%] day -4	%BASO [%] day -4	#NEUT [x10e3/µL] day -4	#LYMPH [x10e3/µL] day -4	#MONO [x10e3/µL] day -4
G 1 / M Saline	1EZF	43.9	44.4	8.2	2.8	0.3	5.02	5.08	0.93
	1FUH	53.0	30.7	6.5	8.7	0.7	4.40	2.55	0.54
	1UYF	59.1	29.1	9.0	2.3	0.3	6.72	3.31	1.02
	Mean	52.0	34.7	7.9	4.6	0.4	5.38	3.65	0.83
	S.D.	7.65	8.41	1.28	3.56	0.23	1.201	1.298	0.255
	N	3	3	3	3	3	3	3	3
G 2 / M Vehicle	2AGF	54.1	39.2	4.6	1.6	0.4	6.98	5.05	0.59
	2BSF	59.4	30.2	8.2	1.7	0.2	6.43	3.27	0.89
	2FRH	60.1	30.3	5.9	3.0	0.3	5.28	2.66	0.52
	Mean	57.9	33.2	6.2	2.1	0.3	6.23	3.66	0.67
	S.D.	3.28	5.17	1.82	0.78	0.10	0.867	1.242	0.197
	N	3	3	3	3	3	3	3	3
G 3 / M Low 0.019 mg/L	3DWH	60.0	25.7	7.2	6.6	0.3	8.17	3.51	0.98
	3THF	64.9	26.7	5.7	2.1	0.3	6.19	2.55	0.55
	3TYF	63.1	25.3	9.0	2.2	0.3	7.45	2.99	1.06
	Mean	62.7	25.9	7.3	3.6	0.3	7.27	3.02	0.86
	S.D.	2.48	0.72	1.65	2.57	0.00	1.002	0.481	0.274
	N	3	3	3	3	3	3	3	3
G 4 / M Mid 0.038 mg/L	4EAH	59.6	31.4	6.4	2.1	0.3	4.30	2.27	0.46
	4JLH	54.1	38.3	4.2	2.6	0.4	6.08	4.30	0.47
	4YJF	57.2	32.9	7.2	2.2	0.3	6.57	3.78	0.82
	Mean	57.0	34.2	5.9	2.3	0.3	5.65	3.45	0.58
	S.D.	2.76	3.63	1.55	0.26	0.06	1.195	1.054	0.205
	N	3	3	3	3	3	3	3	3
G 5 / M High 0.075 mg/L	5GPH	61.2	32.1	4.8	1.6	0.2	7.01	3.68	0.55
	5ZOF	64.6	27.7	5.0	2.2	0.4	7.40	3.18	0.57
	5ZTF	60.8	28.8	5.9	3.9	0.3	7.79	3.69	0.76
	Mean	62.2	29.5	5.2	2.6	0.3	7.40	3.52	0.63
	S.D.	2.09	2.29	0.59	1.19	0.10	0.390	0.292	0.116
	N	3	3	3	3	3	3	3	3

Table F-6 – Individual Animal Hematology Data

PRE-TEST (MALE)

Dose Group	Animal Number	#EOS [x10e3/µL] day -4	#BASO [x10e3/µL] day -4	%RETIC [%] day -4	#RETIC [x10e9/L] day -4	%LUC [%] day -4	#LUC [x10e3/µL] day -4
G 1 / M Saline	1EZF	0.32	0.03	0.74	53.6	0.4	0.05
	1FUH	0.72	0.06	0.56	41.2	0.3	0.03
	1UYF	0.26	0.04	0.66	45.5	0.2	0.03
	Mean	0.43	0.04	0.65	46.8	0.3	0.04
	S.D.	0.250	0.015	0.090	6.30	0.10	0.012
	N	3	3	3	3	3	3
G 2 / M Vehicle	2AGF	0.21	0.05	1.11	87.8	0.1	0.02
	2BSF	0.18	0.03	0.51	37.5	0.3	0.03
	2FRH	0.27	0.03	0.67	46.1	0.3	0.02
	Mean	0.22	0.04	0.76	57.1	0.2	0.02
	S.D.	0.046	0.012	0.311	26.90	0.12	0.006
	N	3	3	3	3	3	3
G 3 / M Low 0.019 mg/L	3DWH	0.90	0.05	0.93	66.6	0.1	0.02
	3THF	0.20	0.03	0.94	59.1	0.2	0.02
	3TYF	0.26	0.03	0.84	62.6	0.2	0.02
	Mean	0.45	0.04	0.90	62.8	0.2	0.02
	S.D.	0.388	0.012	0.055	3.75	0.06	0.000
	N	3	3	3	3	3	3
G 4 / M Mid 0.038 mg/L	4EAH	0.15	0.02	0.94	63.3	0.2	0.02
	4JLH	0.30	0.05	1.24	94.2	0.4	0.05
	4YJF	0.26	0.04	0.32	25.8	0.2	0.02
	Mean	0.24	0.04	0.83	61.1	0.3	0.03
	S.D.	0.078	0.015	0.469	34.25	0.12	0.017
	N	3	3	3	3	3	3
G 5 / M High 0.075 mg/L	5GPH	0.18	0.02	0.64	49.2	0.1	0.02
	5ZOF	0.25	0.04	0.74	47.8	0.2	0.02
	5ZTF	0.49	0.04	0.93	69.7	0.2	0.03
	Mean	0.31	0.03	0.77	55.6	0.2	0.02
	S.D.	0.163	0.012	0.147	12.26	0.06	0.006
	N	3	3	3	3	3	3

Table F-6 – Individual Animal Hematology Data

PRE-TEST (MALE)

Dose Group	Animal Number	ANISO [Scale] day -4	MACRO [Scale] day -4	MICRO [Scale] day -4	HYPO [Scale] day -4	HYPER [Scale] day -4
G 1 / M Saline	1EZF	Norm	Norm	Norm	Norm	Norm
	1FUH	Norm	Norm	Norm	Norm	Norm
	1UYF	Norm	Norm	Norm	Norm	Norm
G 2 / M Vehicle	2AGF	3+	Norm	1+	Norm	Norm
	2BSF	Norm	Norm	Norm	Norm	Norm
	2FRH	Norm	Norm	Norm	Norm	Norm
G 3 / M Low 0.019 mg/L	3DWH	Norm	Norm	Norm	Norm	Norm
	3THF	Norm	Norm	Norm	Norm	Norm
	3TYF	Norm	Norm	Norm	Norm	Norm
G 4 / M Mid 0.038 mg/L	4EAH	Norm	Norm	Norm	Norm	Norm
	4JLH	Norm	Norm	Norm	Norm	Norm
	4YJF	Norm	Norm	Norm	Norm	Norm
G 5 / M High 0.075 mg/L	5GPH	2+	Norm	1+	Norm	Norm
	5ZOF	Norm	Norm	Norm	Norm	Norm
	5ZTF	Norm	Norm	Norm	Norm	Norm

Table F-6 – Individual Animal Hematology Data

PRE-TEST (FEMALE)

Dose Group	Animal Number	WBC [x10e3/µL] day -5	RBC [x10e6/µL] day -5	HGB [g/dL] day -5	HCT [%] day -5	MCV [fL] day -5	MCH [pg] day -5	MCHC [g/dL] day -5	PLT [x10e3/µL] day -5
G 1 / F Saline	1CBE	12.28	7.81	17.7	52.0	66.6	22.6	34.0	351
	1JKG	12.67	8.12	17.6	50.4	62.1	21.6	34.9	292
	1JSG	12.83	6.62	15.3	44.1	66.6	23.0	34.6	349
	Mean	12.59	7.52	16.9	48.8	65.1	22.4	34.5	331
	S.D.	0.283	0.792	1.36	4.18	2.60	0.72	0.46	33.5
	N	3	3	3	3	3	3	3	3
G 2 / F Vehicle	2AJE	13.43	7.20	15.7	46.0	63.8	21.8	34.2	360
	2ZQE	11.84	6.94	15.9	46.5	67.0	22.9	34.2	329
	2FLE	9.13	6.34	14.6	42.9	67.7	23.1	34.1	350
	Mean	11.47	6.83	15.4	45.1	66.2	22.6	34.2	346
	S.D.	2.174	0.441	0.70	1.95	2.08	0.70	0.06	15.8
	N	3	3	3	3	3	3	3	3
G 3 / F Low 0.019 mg/L	3CVG	12.71	8.06	16.3	49.4	61.3	20.2	32.9	300
	3FKE	9.22	6.78	15.3	45.4	67.0	22.6	33.7	386
	3ZRE	21.93	6.76	15.9	46.4	68.7	23.5	34.3	386
	Mean	14.62	7.20	15.8	47.1	65.7	22.1	33.6	357
	S.D.	6.567	0.745	0.50	2.08	3.88	1.71	0.70	49.7
	N	3	3	3	3	3	3	3	3
G 4 / F Mid 0.038 mg/L	4ACE	13.46	7.35	17.3	49.8	67.8	23.5	34.6	400
	4CAE	10.44	8.18	18.2	52.6	64.3	22.3	34.6	265
	4EVE	14.02	6.35	14.9	44.2	69.5	23.5	33.8	339
	Mean	12.64	7.29	16.8	48.9	67.2	23.1	34.3	335
	S.D.	1.926	0.916	1.71	4.28	2.65	0.69	0.46	67.6
	N	3	3	3	3	3	3	3	3
G 5 / F High 0.075 mg/L	5AIE	12.53	8.09	18.2	52.7	65.1	22.5	34.5	316
	5CGE	10.25	6.49	14.6	42.9	66.0	22.5	34.1	405
	5CSG	15.17	8.19	18.4	53.5	65.4	22.5	34.4	234
	Mean	12.65	7.59	17.1	49.7	65.5	22.5	34.3	318
	S.D.	2.462	0.954	2.14	5.90	0.46	0.00	0.21	85.5
	N	3	3	3	3	3	3	3	3

Table F-6 – Individual Animal Hematology Data

PRE-TEST (FEMALE)

Dose Group	Animal Number	%NEUT [%] day -5	%LYMPH [%] day -5	%MONO [%] day -5	%EOS [%] day -5	%BASO [%] day -5	#NEUT [x10e3/µL] day -5	#LYMPH [x10e3/µL] day -5	#MONO [x10e3/µL] day -5
G 1 / F Saline	1CBE	68.4	22.1	7.1	1.9	0.4	8.40	2.71	0.87
	1JKG	58.7	30.9	7.2	2.6	0.3	7.44	3.92	0.91
	1JSG	60.9	29.8	6.6	2.2	0.3	7.81	3.82	0.84
	Mean	62.7	27.6	7.0	2.2	0.3	7.88	3.48	0.87
	S.D.	5.09	4.79	0.32	0.35	0.06	0.484	0.672	0.035
	N	3	3	3	3	3	3	3	3
G 2 / F Vehicle	2AJE	78.4	13.9	6.7	0.8	0.1	10.53	1.87	0.90
	2ZQE	68.8	23.9	4.9	2.2	0.2	8.15	2.83	0.58
	2FLE	50.8	41.5	6.2	1.1	0.2	4.64	3.79	0.56
	Mean	66.0	26.4	5.9	1.4	0.2	7.77	2.83	0.68
	S.D.	14.01	13.97	0.93	0.74	0.06	2.963	0.960	0.191
	N	3	3	3	3	3	3	3	3
G 3 / F Low 0.019 mg/L	3CVG	53.3	33.4	8.4	4.3	0.3	6.78	4.25	1.07
	3FKE	58.6	31.9	7.4	1.7	0.3	5.40	2.94	0.68
	3ZRE	70.8	19.8	8.1	0.9	0.2	15.54	4.35	1.78
	Mean	60.9	28.4	8.0	2.3	0.3	9.24	3.85	1.18
	S.D.	8.97	7.46	0.51	1.78	0.06	5.499	0.787	0.558
	N	3	3	3	3	3	3	3	3
G 4 / F Mid 0.038 mg/L	4ACE	55.4	32.8	8.9	2.1	0.3	7.45	4.41	1.20
	4CAE	54.7	35.7	7.6	1.4	0.5	5.71	3.73	0.79
	4EVE	67.3	25.2	6.2	0.8	0.2	9.43	3.54	0.87
	Mean	59.1	31.2	7.6	1.4	0.3	7.53	3.89	0.95
	S.D.	7.08	5.42	1.35	0.65	0.15	1.861	0.457	0.217
	N	3	3	3	3	3	3	3	3
G 5 / F High 0.075 mg/L	5AIE	55.4	36.4	4.8	2.6	0.5	6.94	4.57	0.60
	5CGE	58.4	28.7	7.9	4.6	0.2	5.98	2.94	0.81
	5CSG	67.9	23.0	7.1	1.0	0.6	10.30	3.49	1.08
	Mean	60.6	29.4	6.6	2.7	0.4	7.74	3.67	0.83
	S.D.	6.53	6.72	1.61	1.80	0.21	2.268	0.829	0.241
	N	3	3	3	3	3	3	3	3

Table F-6 – Individual Animal Hematology Data

PRE-TEST (FEMALE)

Dose Group	Animal Number	#EOS [x10e3/µL] day -5	#BASO [x10e3/µL] day -5	%RETIC [%] day -5	#RETIC [x10e9/L] day -5	%LUC [%] day -5	#LUC [x10e3/µL] day -5
G 1 / F Saline	1CBE	0.23	0.05	0.69	53.6	0.2	0.02
	1JKG	0.33	0.04	0.57	46.3	0.3	0.04
	1JSG	0.28	0.04	0.72	47.7	0.2	0.03
	Mean	0.28	0.04	0.66	49.2	0.2	0.03
	S.D.	0.050	0.006	0.079	3.87	0.06	0.010
	N	3	3	3	3	3	3
G 2 / F Vehicle	2AJE	0.11	0.02	0.73	52.8	0.0	0.00
	2ZQE	0.26	0.02	0.73	50.4	0.1	0.01
	2FLE	0.10	0.01	0.53	33.6	0.3	0.03
	Mean	0.16	0.02	0.66	45.6	0.1	0.01
	S.D.	0.090	0.006	0.115	10.46	0.15	0.015
	N	3	3	3	3	3	3
G 3 / F Low 0.019 mg/L	3CVG	0.55	0.04	0.29	23.4	0.2	0.03
	3FKE	0.16	0.03	0.65	44.2	0.2	0.02
	3ZRE	0.20	0.04	2.65	179.1	0.1	0.02
	Mean	0.30	0.04	1.20	82.2	0.2	0.02
	S.D.	0.215	0.006	1.271	84.53	0.06	0.006
	N	3	3	3	3	3	3
G 4 / F Mid 0.038 mg/L	4ACE	0.29	0.05	0.96	70.8	0.5	0.07
	4CAE	0.15	0.05	1.19	97.4	0.1	0.01
	4EVE	0.12	0.02	1.11	70.8	0.3	0.04
	Mean	0.19	0.04	1.09	79.7	0.3	0.04
	S.D.	0.091	0.017	0.117	15.36	0.20	0.030
	N	3	3	3	3	3	3
G 5 / F High 0.075 mg/L	5AIE	0.33	0.06	0.98	79.2	0.3	0.04
	5CGE	0.48	0.03	0.71	46.3	0.2	0.02
	5CSG	0.16	0.10	0.68	55.7	0.4	0.06
	Mean	0.32	0.06	0.79	60.4	0.3	0.04
	S.D.	0.160	0.035	0.165	16.95	0.10	0.020
	N	3	3	3	3	3	3

Table F-6 – Individual Animal Hematology Data

PRE-TEST (FEMALE)

Dose Group	Animal Number	ANISO [Scale] day -5	MACRO [Scale] day -5	MICRO [Scale] day -5	HYPO [Scale] day -5	HYPER [Scale] day -5
G 1 / F Saline	1CBE	Norm	Norm	Norm	Norm	Norm
	1JKG	Norm	Norm	Norm	Norm	Norm
	1JSG	Norm	Norm	Norm	Norm	Norm
G 2 / F Vehicle	2AJE	1+	Norm	1+	Norm	Norm
	2ZQE	Norm	Norm	Norm	Norm	Norm
	2FLE	Norm	Norm	Norm	Norm	Norm
G 3 / F Low 0.019 mg/L	3CVG	2+	Norm	1+	Norm	Norm
	3FKE	Norm	Norm	Norm	Norm	Norm
	3ZRE	Norm	Norm	Norm	Norm	Norm
G 4 / F Mid 0.038 mg/L	4ACE	Norm	Norm	Norm	Norm	Norm
	4CAE	Norm	Norm	Norm	Norm	Norm
	4EVE	Norm	Norm	Norm	Norm	Norm
G 5 / F High 0.075 mg/L	5AIE	Norm	Norm	Norm	Norm	Norm
	5CGE	Norm	Norm	Norm	Norm	Norm
	5CSG	Norm	Norm	Norm	Norm	Norm

Table F-6 – Individual Animal Hematology Data

DAY 15 (MALE)

Dose Group	Animal Number	WBC [x10e3/µL] day 15	RBC [x10e6/µL] day 15	HGB [g/dL] day 15	HCT [%] day 15	MCV [fL] day 15	MCH [pg] day 15	MCHC [g/dL] day 15	PLT [x10e3/µL] day 15
G 1 / M Saline	1EZF	7.87	6.41	14.3	44.3	69.1	22.3	32.3	285
	1FUH	6.72	6.41	15.2	45.6	71.1	23.7	33.3	248
	1UYF	7.68	6.76	15.2	47.2	69.9	22.4	32.1	358
	Mean	7.42	6.53	14.9	45.7	70.0	22.8	32.6	297
	S.D.	0.616	0.202	0.52	1.45	1.01	0.78	0.64	56.0
	N	3	3	3	3	3	3	3	3
G 2 / M Vehicle	2AGF	8.62	7.38	15.9	49.8	67.4	21.5	31.9	386
	2BSF	7.66	6.77	13.8	43.8	64.6	20.4	31.6	345
	2FRH	7.21	6.80	16.1	49.9	73.4	23.6	32.2	336
	Mean	7.83	6.98	15.3	47.8	68.5	21.8	31.9	356
	S.D.	0.720	0.344	1.27	3.49	4.50	1.63	0.30	26.7
	N	3	3	3	3	3	3	3	3
G 3 / M Low 0.019 mg/L	3DWH	11.81	6.69	15.1	47.4	70.9	22.6	31.9	362
	3THF	8.52	6.16	14.7	45.1	73.2	23.9	32.6	398
	3TYF	7.89	6.91	14.8	46.1	66.7	21.4	32.1	404
	Mean	9.41	6.59	14.9	46.2	70.3	22.6	32.2	388
	S.D.	2.105	0.386	0.21	1.15	3.30	1.25	0.36	22.7
	N	3	3	3	3	3	3	3	3
G 4 / M Mid 0.038 mg/L	4EAH	8.27	6.01	13.6	42.1	70.1	22.6	32.2	530
	4JLH	7.53	6.63	15.4	47.2	71.1	23.2	32.6	279
	4YJF	8.47	7.01	15.4	47.4	67.6	22.0	32.6	313
	Mean	8.09	6.55	14.8	45.6	69.6	22.6	32.5	374
	S.D.	0.495	0.505	1.04	3.00	1.80	0.60	0.23	136.2
	N	3	3	3	3	3	3	3	3
G 5 / M High 0.075 mg/L	5GPH	9.96	6.79	15.2	46.9	69.1	22.4	32.4	316
	5ZOF	9.35	6.34	14.2	44.1	69.6	22.4	30.2	451
	5ZTF	8.40	7.31	16.1	48.8	66.8	22.1	33.1	360
	Mean	9.24	6.81	15.2	46.6	68.5	22.3	31.9	376
	S.D.	0.786	0.485	0.95	2.36	1.49	0.17	1.51	68.9
	N	3	3	3	3	3	3	3	3

Table F-6 – Individual Animal Hematology Data

DAY 15 (MALE)

Dose Group	Animal Number	%NEUT [%] day 15	%LYMPH [%] day 15	%MONO [%] day 15	%EOS [%] day 15	%BASO [%] day 15	#NEUT [x10e3/µL] day 15	#LYMPH [x10e3/µL] day 15	#MONO [x10e3/µL] day 15
G 1 / M Saline	1EZF	46.8	42.5	8.6	1.5	0.1	3.69	3.35	0.68
	1FUH	57.7	30.7	6.4	4.5	0.3	3.88	2.07	0.43
	1UYF	59.9	27.6	10.8	1.2	0.1	4.60	2.12	0.83
	Mean	54.8	33.6	8.6	2.4	0.2	4.06	2.51	0.65
	S.D.	7.01	7.86	2.20	1.82	0.12	0.480	0.725	0.202
	N	3	3	3	3	3	3	3	3
G 2 / M Vehicle	2AGF	62.8	30.2	4.8	1.7	0.3	5.42	2.60	0.41
	2BSF	28.8	32.7	6.7	1.2	0.2	4.50	2.50	0.51
	2FRH	62.6	30.8	4.9	1.1	0.1	4.51	2.22	0.36
	Mean	51.4	31.2	5.5	1.3	0.2	4.81	2.44	0.43
	S.D.	19.57	1.31	1.07	0.32	0.10	0.528	0.197	0.076
	N	3	3	3	3	3	3	3	3
G 3 / M Low 0.019 mg/L	3DWH	66.4	24.0	5.4	3.8	0.2	7.84	2.84	0.64
	3THF	72.9	19.3	6.8	0.7	0.2	6.21	1.64	0.58
	3TYF	58.7	28.4	11.1	1.2	0.2	4.63	2.24	0.87
	Mean	66.0	23.9	7.8	1.9	0.2	6.23	2.24	0.70
	S.D.	7.11	4.55	2.97	1.66	0.00	1.605	0.600	0.153
	N	3	3	3	3	3	3	3	3
G 4 / M Mid 0.038 mg/L	4EAH	68.2	23.0	7.7	0.6	0.1	5.64	1.90	0.64
	4JLH	59.3	35.1	3.6	1.3	0.3	4.46	2.64	0.27
	4YJF	59.0	32.4	6.0	2.2	0.2	4.99	2.74	0.51
	Mean	62.2	30.2	5.8	1.4	0.2	5.03	2.43	0.47
	S.D.	5.23	6.35	2.06	0.80	0.10	0.591	0.459	0.188
	N	3	3	3	3	3	3	3	3
G 5 / M High 0.075 mg/L	5GPH	63.8	29.2	4.2	2.2	0.2	6.36	2.91	0.42
	5ZOF	66.5	23.8	6.1	3.4	0.1	6.22	2.22	0.57
	5ZTF	60.6	30.5	5.5	2.8	0.4	5.09	2.56	0.46
	Mean	63.6	27.8	5.3	2.8	0.2	5.89	2.56	0.48
	S.D.	2.95	3.55	0.97	0.60	0.15	0.696	0.345	0.078
	N	3	3	3	3	3	3	3	3

Table F-6 – Individual Animal Hematology Data

DAY 15 (MALE)

Dose Group	Animal Number	#EOS [x10e3/µL] day 15	#BASO [x10e3/µL] day 15	%RETIC [%] day 15	#RETIC [x10e9/L] day 15	%LUC [%] day 15	#LUC [x10e3/µL] day 15
G 1 / M Saline	1EZF	0.12	0.01	0.95	61.2	0.4	0.03
	1FUH	0.30	0.02	0.42	27.1	0.3	0.02
	1UYF	0.09	0.01	1.04	70.6	0.3	0.03
	Mean	0.17	0.01	0.80	53.0	0.3	0.03
	S.D.	0.114	0.006	0.335	22.89	0.06	0.006
	N	3	3	3	3	3	3
G 2 / M Vehicle	2AGF	0.15	0.02	0.49	36.2	0.2	0.02
	2BSF	0.09	0.02	0.64	43.0	0.4	0.03
	2FRH	0.08	0.01	1.22	83.1	0.3	0.02
	Mean	0.11	0.02	0.78	54.1	0.3	0.02
	S.D.	0.038	0.006	0.386	25.34	0.10	0.006
	N	3	3	3	3	3	3
G 3 / M Low 0.019 mg/L	3DWH	0.45	0.03	1.54	103.0	0.2	0.02
	3THF	0.06	0.02	1.92	118.1	0.2	0.01
	3TYF	0.09	0.01	0.99	68.7	0.5	0.04
	Mean	0.20	0.02	1.48	96.6	0.3	0.02
	S.D.	0.217	0.010	0.468	25.31	0.17	0.015
	N	3	3	3	3	3	3
G 4 / M Mid 0.038 mg/L	4EAH	0.05	0.01	1.60	96.5	0.3	0.03
	4JLH	0.10	0.03	0.48	32.0	0.4	0.03
	4YJF	0.19	0.01	0.72	50.5	0.3	0.02
	Mean	0.11	0.02	0.93	59.7	0.3	0.03
	S.D.	0.071	0.012	0.590	33.21	0.06	0.006
	N	3	3	3	3	3	3
G 5 / M High 0.075 mg/L	5GPH	0.22	0.02	0.79	53.6	0.3	0.03
	5ZOF	0.32	0.01	1.31	83.0	0.2	0.02
	5ZTF	0.23	0.03	0.98	71.8	0.3	0.03
	Mean	0.26	0.02	1.03	69.5	0.3	0.03
	S.D.	0.055	0.010	0.263	14.84	0.06	0.006
	N	3	3	3	3	3	3

Table F-6 – Individual Animal Hematology Data

DAY 15 (MALE)

Dose Group	Animal Number	ANISO [Scale] day 15	MACRO [Scale] day 15	MICRO [Scale] day 15	HYPO [Scale] day 15	HYPER [Scale] day 15
G 1 / M Saline	1EZF	Norm	Norm	Norm	Norm	Norm
	1FUH	Norm	Norm	Norm	Norm	Norm
	1UYF	Norm	Norm	Norm	Norm	Norm
G 2 / M Vehicle	2AGF	2+	Norm	Norm	Norm	Norm
	2BSF	Norm	Norm	Norm	Norm	Norm
	2FRH	Norm	1+	Norm	Norm	Norm
G 3 / M Low 0.019 mg/L	3DWH	Norm	Norm	Norm	Norm	Norm
	3THF	Norm	1+	Norm	Norm	Norm
	3TYF	Norm	Norm	Norm	Norm	Norm
G 4 / M Mid 0.038 mg/L	4EAH	Norm	1+	Norm	Norm	Norm
	4JLH	Norm	Norm	Norm	Norm	Norm
	4YJF	Norm	Norm	Norm	Norm	Norm
G 5 / M High 0.075 mg/L	5GPH	1+	Norm	Norm	Norm	Norm
	5ZOF	Norm	Norm	Norm	Norm	Norm
	5ZTF	Norm	Norm	Norm	Norm	Norm

Table F-6 – Individual Animal Hematology Data

DAY 15 (FEMALE)

Dose Group	Animal Number	WBC [x10e3/μL] day 15	RBC [x10e6/μL] day 15	HGB [g/dL] day 15	HCT [%] day 15	MCV [fL] day 15	MCH [pg] day 15	MCHC [g/dL] day 15	PLT [x10e3/μL] day 15
G 1 / F Saline	1CBE	7.47	7.04	15.8	48.9	69.5	22.5	32.4	422
	1JKG	7.10	7.04	15.4	46.9	66.6	21.8	32.8	283
	1JSG	8.54	7.19	16.5	50.7	70.5	23.0	32.6	329
	Mean	7.70	7.09	15.9	48.8	68.9	22.4	32.6	345
	S.D.	0.748	0.087	0.56	1.90	2.03	0.60	0.20	70.8
	N	3	3	3	3	3	3	3	3
G 2 / F Vehicle	2AJE	6.19	6.88	15.4	47.7	69.4	22.4	32.3	392
	2ZQE	10.95	7.12	16.1	50.1	70.3	22.6	32.2	434
	2FLE	6.94	6.81	15.7	48.4	71.1	23.1	32.5	316
	Mean	8.03	6.94	15.7	48.7	70.3	22.7	32.3	381
	S.D.	2.559	0.163	0.35	1.23	0.85	0.36	0.15	59.8
	N	3	3	3	3	3	3	3	3
G 3 / F Low 0.019 mg/L	3CVG	11.04	8.03	16.5	52.3	65.1	20.6	31.6	343
	3FKE	6.99	6.39	14.3	45.6	71.3	22.4	31.4	290
	3ZRE	17.28	6.58	15.2	47.3	71.9	23.1	32.1	428
	Mean	11.77	7.00	15.3	48.4	69.4	22.0	31.7	354
	S.D.	5.184	0.897	1.11	3.48	3.76	1.29	0.36	69.6
	N	3	3	3	3	3	3	3	3
G 4 / F Mid 0.038 mg/L	4ACE	9.03	6.68	15.5	47.4	71.0	23.1	32.6	344
	4CAE	7.76	7.98	17.5	54.8	68.7	21.9	31.9	275
	4EVE	11.15	6.15	14.5	44.5	72.4	23.6	32.6	422
	Mean	9.31	6.94	15.8	48.9	70.7	22.9	32.4	347
	S.D.	1.713	0.942	1.53	5.31	1.87	0.87	0.40	73.5
	N	3	3	3	3	3	3	3	3
G 5 / F High 0.075 mg/L	5AIE	8.91	7.39	16.5	51.2	69.3	22.3	32.2	284
	5CGE	9.19	6.84	15.3	47.8	69.8	22.3	32.0	424
	5CSG	9.34	7.26	16.4	49.6	68.4	22.6	33.0	433
	Mean	9.15	7.16	16.1	49.5	69.2	22.4	32.4	380
	S.D.	0.218	0.287	0.67	1.70	0.71	0.17	0.53	83.5
	N	3	3	3	3	3	3	3	3

Table F-6 – Individual Animal Hematology Data

DAY 15 (FEMALE)

Dose Group	Animal Number	%NEUT [%] day 15	%LYMPH [%] day 15	%MONO [%] day 15	%EOS [%] day 15	%BASO [%] day 15	#NEUT [x10e3/µL] day 15	#LYMPH [x10e3/µL] day 15	#MONO [x10e3/µL] day 15
G 1 / F Saline	1CBE	60.8	29.9	6.8	1.9	0.3	4.54	2.23	0.50
	1JKG	54.5	35.5	7.5	1.6	0.4	3.87	2.52	0.53
	1JSG	52.2	41.9	4.0	0.9	0.4	4.46	3.58	0.34
	Mean	55.8	35.8	6.1	1.5	0.4	4.29	2.78	0.46
	S.D.	4.45	6.00	1.85	0.51	0.06	0.366	0.711	0.102
	N	3	3	3	3	3	3	3	3
G 2 / F Vehicle	2AJE	65.8	24.4	7.1	2.2	0.1	4.07	1.51	0.44
	2ZQE	72.6	21.7	4.6	0.7	0.1	7.95	2.38	0.50
	2FLE	53.1	39.6	4.9	1.8	0.2	3.69	2.75	0.34
	Mean	63.8	28.6	5.5	1.6	0.1	5.24	2.21	0.43
	S.D.	9.90	9.65	1.37	0.78	0.06	2.357	0.637	0.081
	N	3	3	3	3	3	3	3	3
G 3 / F Low 0.019 mg/L	3CVG	44.8	44.1	7.7	2.7	0.3	4.94	4.87	0.85
	3FKE	51.6	40.8	5.7	1.3	0.3	3.61	2.85	0.40
	3ZRE	72.7	20.5	5.8	0.7	0.2	12.55	3.54	0.99
	Mean	56.4	35.1	6.4	1.6	0.3	7.03	3.75	0.75
	S.D.	14.55	12.78	1.13	1.03	0.06	4.824	1.027	0.308
	N	3	3	3	3	3	3	3	3
G 4 / F Mid 0.038 mg/L	4ACE	52.7	37.9	6.5	1.6	0.4	4.76	3.42	0.59
	4CAE	58.8	32.9	5.7	1.9	0.4	4.56	2.55	0.44
	4EVE	64.0	30.8	4.3	0.4	0.1	7.14	3.43	0.48
	Mean	58.5	33.9	5.5	1.3	0.3	5.49	3.13	0.50
	S.D.	5.66	3.65	1.11	0.79	0.17	1.435	0.505	0.078
	N	3	3	3	3	3	3	3	3
G 5 / F High 0.075 mg/L	5AIE	57.7	35.0	4.1	2.0	0.6	5.14	3.11	0.37
	5CGE	59.8	29.9	5.6	4.3	0.3	5.50	2.75	0.52
	5CSG	60.9	30.9	6.1	0.8	0.6	5.69	2.89	0.57
	Mean	59.5	31.9	5.3	2.4	0.5	5.44	2.92	0.49
	S.D.	1.63	2.70	1.04	1.78	0.17	0.279	0.181	0.104
	N	3	3	3	3	3	3	3	3

Table F-6 – Individual Animal Hematology Data

DAY 15 (FEMALE)

Dose Group	Animal Number	#EOS [x10e3/µL] day 15	#BASO [x10e3/µL] day 15	%RETIC [%] day 15	#RETIC [x10e9/L] day 15	%LUC [%] day 15	#LUC [x10e3/µL] day 15
G 1 / F Saline	1CBE	0.14	0.02	0.95	66.7	0.3	0.02
	1JKG	0.12	0.03	0.64	44.8	0.5	0.03
	1JSG	0.08	0.03	1.28	91.9	0.5	0.04
	Mean	0.11	0.03	0.96	67.8	0.4	0.03
	S.D.	0.031	0.006	0.320	23.57	0.12	0.010
	N	3	3	3	3	3	3
G 2 / F Vehicle	2AJE	0.14	0.01	1.72	118.2	0.3	0.20
	2ZQE	0.07	0.01	0.86	61.5	0.2	0.03
	2FLE	0.13	0.01	1.11	75.3	0.4	0.02
	Mean	0.11	0.01	1.23	85.0	0.3	0.08
	S.D.	0.038	0.000	0.442	29.57	0.10	0.101
	N	3	3	3	3	3	3
G 3 / F Low 0.019 mg/L	3CVG	0.29	0.04	0.70	56.2	0.5	0.05
	3FKE	0.09	0.02	1.26	80.6	0.3	0.02
	3ZRE	0.12	0.03	1.65	108.2	0.3	0.05
	Mean	0.17	0.03	1.20	81.7	0.4	0.04
	S.D.	0.108	0.010	0.478	26.02	0.12	0.017
	N	3	3	3	3	3	3
G 4 / F Mid 0.038 mg/L	4ACE	0.14	0.04	1.23	82.2	0.9	0.08
	4CAE	0.14	0.03	1.60	127.5	0.4	0.03
	4EVE	0.04	0.01	2.15	132.1	0.4	0.04
	Mean	0.11	0.03	1.66	113.9	0.6	0.05
	S.D.	0.058	0.015	0.463	27.58	0.29	0.026
	N	3	3	3	3	3	3
G 5 / F High 0.075 mg/L	5AIE	0.18	0.05	1.38	102.3	0.6	0.05
	5CGE	0.39	0.02	1.57	107.2	0.1	0.01
	5CSG	0.08	0.06	0.79	57.4	0.7	0.06
	Mean	0.22	0.04	1.25	89.0	0.5	0.04
	S.D.	0.158	0.021	0.407	27.45	0.32	0.026
	N	3	3	3	3	3	3

Table F-6 – Individual Animal Hematology Data

DAY 15 (FEMALE)

Dose Group	Animal Number	ANISO [Scale] day 15	MACRO [Scale] day 15	MICRO [Scale] day 15	HYPO [Scale] day 15	HYPER [Scale] day 15
G 1 / F Saline	1CBE	Norm	Norm	Norm	Norm	Norm
	1JKG	Norm	Norm	Norm	Norm	Norm
	1JSG	Norm	Norm	Norm	Norm	Norm
G 2 / F Vehicle	2AJE	2+	1+	Norm	Norm	Norm
	2ZQE	Norm	Norm	Norm	Norm	Norm
	2FLE	Norm	Norm	Norm	Norm	Norm
G 3 / F Low 0.019 mg/L	3CVG	3+	Norm	1+	Norm	Norm
	3FKE	Norm	1+	Norm	Norm	Norm
	3ZRE	Norm	1+	Norm	Norm	Norm
G 4 / F Mid 0.038 mg/L	4ACE	Norm	Norm	Norm	Norm	Norm
	4CAE	Norm	Norm	Norm	Norm	Norm
	4EVE	Norm	1+	Norm	Norm	Norm
G 5 / F High 0.075 mg/L	5AIE	1+	Norm	Norm	Norm	Norm
	5CGE	Norm	Norm	Norm	Norm	Norm
	5CSG	Norm	Norm	Norm	Norm	Norm

Table F-7 – Individual Animal Coagulation Data

PRE-TEST (MALE)

Dose Group	Animal Number	PT [sec] day -4	APTT [sec] day -4	FIB [mg/dL] day -4
G 1 / M Saline	1EZF	8.2	17.2	209
	1FUH	7.8	17.3	201
	1UYF	7.9	19.3	232
	Mean	8.0	17.9	214
	S.D.	0.21	1.18	16.1
	N	3	3	3
G 2 / M Vehicle	2AGF	7.8	14.9	183
	2BSF	8.4	13.6	150
	2FRH	7.9	19.0	216
	Mean	8.0	15.8	183
	S.D.	0.32	2.82	33.0
	N	3	3	3
G 3 / M Low 0.019 mg/L	3DWH	8.1	15.8	244
	3THF	7.8	15.0	230
	3TYF	7.9	16.4	218
	Mean	7.9	15.7	231
	S.D.	0.15	0.70	13.0
	N	3	3	3
G 4 / M Mid 0.038 mg/L	4EAH	8.2	17.2	259
	4JLH	8.2	19.3	381
	4YJF	7.9	15.3	220
	Mean	8.1	17.3	287
	S.D.	0.17	2.00	84.0
	N	3	3	3
G 5 / M High 0.075 mg/L	5GPH	7.9	18.1	191
	5ZOF	8.9	12.6	183
	5ZTF	8.2	18.1	224
	Mean	8.3	16.3	199
	S.D.	0.51	3.18	21.7
	N	3	3	3

Table F-7 – Individual Animal Coagulation Data

PRE-TEST (FEMALE)

Dose Group	Animal Number	PT [sec] day -5	APTT [sec] day -5	FIB [mg/dL] day -5
G 1 / F Saline	1CBE	8.2	16.5	207
	1JKG	7.7	17.4	215
	1JSG	8.5	15.7	194
	Mean	8.1	16.5	205
	S.D.	0.40	0.85	10.6
	N	3	3	3
G 2 / F Vehicle	2AJE	8.1	15.0	235
	2ZQE	13.7	17.3	201
	2FLE	7.9	15.9	207
	Mean	9.9	16.1	214
	S.D.	3.29	1.16	18.1
	N	3	3	3
G 3 / F Low 0.019 mg/L	3CVG	8.9	16.0	163
	3FKE	8.2	17.2	189
	3ZRE	8.8	15.0	246
	Mean	8.6	16.1	199
	S.D.	0.38	1.10	42.5
	N	3	3	3
G 4 / F Mid 0.038 mg/L	4ACE	7.9	16.7	286
	4CAE	9.0	16.6	198
	4EVE	7.8	16.8	210
	Mean	8.2	16.7	231
	S.D.	0.67	0.10	47.7
	N	3	3	3
G 5 / F High 0.075 mg/L	5AIE	8.4	16.6	178
	5CGE	8.2	18.4	184
	5CSG	9.3	16.4	296
	Mean	8.6	17.1	219
	S.D.	0.59	1.10	66.5
	N	3	3	3

Table F-7 – Individual Animal Coagulation Data

DAY 15 (MALE)

Dose Group	Animal Number	PT [sec] day 15	APTT [sec] day 15	FIB [mg/dL] day 15
G 1 / M Saline	1EZF	8.1	14.4	191
	1FUH	8.3	15.1	187
	1UYF	8.2	16.8	194
	Mean	8.2	15.4	191
	S.D.	0.10	1.23	3.5
	N	3	3	3
G 2 / M Vehicle	2AGF	8.1	14.7	174
	2BSF	8.0	15.2	225
	2FRH	8.4	16.2	153
	Mean	8.2	15.4	184
	S.D.	0.21	0.76	37.0
	N	3	3	3
G 3 / M Low 0.019 mg/L	3DWH	8.1	14.9	237
	3THF	8.1	14.5	167
	3TYF	7.7	13.8	234
	Mean	8.0	14.4	213
	S.D.	0.23	0.56	39.6
	N	3	3	3
G 4 / M Mid 0.038 mg/L	4EAH	8.1	14.7	372
	4JLH	7.9	14.6	197
	4YJF	7.9	13.9	200
	Mean	8.0	14.4	256
	S.D.	0.12	0.44	100.2
	N	3	3	3
G 5 / M High 0.075 mg/L	5GPH	8.1	16.1	161
	5ZOF	9.0	15.3	215
	5ZTF	8.0	15.1	191
	Mean	8.4	15.5	189
	S.D.	0.55	0.53	27.1
	N	3	3	3

Table F-7 – Individual Animal Coagulation Data

DAY 15 (FEMALE)

Dose Group	Animal Number	PT [sec] day 15	APTT [sec] day 15	FIB [mg/dL] day 15
G 1 / F Saline	1CBE	8.2	16.4	164
	1JKG	8.1	18.2	203
	1JSG	9.1	16.8	167
	Mean	8.5	17.1	178
	S.D.	0.55	0.95	21.7
	N	3	3	3
G 2 / F Vehicle	2AJE	8.1	15.1	213
	2ZQE	12.8	17.0	282
	2FLE	8.0	15.8	257
	Mean	9.6	16.0	251
	S.D.	2.74	0.96	34.9
	N	3	3	3
G 3 / F Low 0.019 mg/L	3CVG	9.4	16.6	147
	3FKE	8.3	16.0	158
	3ZRE	8.5	15.2	303
	Mean	8.7	15.9	203
	S.D.	0.59	0.70	87.1
	N	3	3	3
G 4 / F Mid 0.038 mg/L	4ACE	8.1	14.0	158
	4CAE	8.9	15.7	167
	4EVE	7.9	15.2	166
	Mean	8.3	15.0	164
	S.D.	0.53	0.87	4.9
	N	3	3	3
G 5 / F High 0.075 mg/L	5AIE	8.3	15.6	165
	5CGE	7.9	17.6	195
	5CSG	8.3	17.1	319
	Mean	8.2	16.8	226
	S.D.	0.23	1.04	81.6
	N	3	3	3

Table F-8 – Individual Animal Urinalysis Data

PRE-TEST (MALE)

Dose Group	Animal Number	Refractive Index [-] day 1	SG [-] day 1	pH [-] day 1	Volume [mL] day 1
G 1 / M Saline	1EZF	1.3420	1.021	6.0	12.0
	1FUH	1.3590	1.062	5.0	11.0
	1UYF	1.3400	1.016	6.0	9.5
	Mean	1.3470	1.033	5.7	10.8
	S.D.	0.01044	0.0252	0.58	1.26
	N	3	3	3	3
G 2 / M Vehicle	2AGF	1.3540	1.049	6.0	11.0
	2BSF	1.3530	1.047	5.0	11.0
	2FRH	1.3540	1.049	5.0	11.0
	Mean	1.3537	1.048	5.3	11.0
	S.D.	0.00058	0.0012	0.58	0.00
	N	3	3	3	3
G 3 / M Low 0.019 mg/L	3DWH	1.3692	1.086	5.0	11.0
	3THF	1.3420	1.021	5.0	11.0
	3TYF	1.3414	1.020	5.0	11.0
	Mean	1.3509	1.042	5.0	11.0
	S.D.	0.01588	0.0378	0.00	0.00
	N	3	3	3	3
G 4 / M Mid 0.038 mg/L	4EAH	1.3410	1.019	6.0	11.0
	4JLH	1.3378	1.012	6.0	10.5
	4YJF	1.3376	1.010	6.0	11.0
	Mean	1.3388	1.014	6.0	10.8
	S.D.	0.00191	0.0047	0.00	0.29
	N	3	3	3	3
G 5 / M High 0.075 mg/L	5GPH	1.3504	1.040	5.0	10.0
	5ZOF	1.3392	1.014	5.0	11.0
	5ZTF	1.3340	1.002	6.5	11.0
	Mean	1.3412	1.019	5.5	10.7
	S.D.	0.00838	0.0194	0.87	0.58
	N	3	3	3	3

NOTE: Pre-test urine samples were collected prior to the first exposure on Day 1.

Table F-8 – Individual Animal Urinalysis Data

PRE-TEST (MALE)

Dose Group	Animal Number	COL [-] day 1	CLA [-] day 1	LEU [-] day 1	NIT [-] day 1	PRO [-] day 1	GLU [-] day 1
G 1 / M Saline	1EZF	Yellow	Clear	2+	negative	neg	neg
	1FUH	Yellow	Clear	2+	positive	2+	neg
	1UYF	Yellow	Clear	1+	negative	neg	neg
G 2 / M Vehicle	2AGF	Yellow	Clear	2+	negative	2+	neg
	2BSF	Yellow	Clear	2+	negative	2+	neg
	2FRH	Yellow	Clear	3+	positive	1+	neg
G 3 / M Low 0.019 mg/L	3DWH	Yellow	Clear	1+	positive	2+	neg
	3THF	Yellow	Clear	3+	negative	neg	neg
	3TYF	Yellow	Clear	3+	negative	1+	neg
G 4 / M Mid 0.038 mg/L	4EAH	Yellow	Clear	neg	negative	neg	neg
	4JLH	Yellow	Clear	1+	negative	neg	neg
	4YJF	Yellow	Clear	1+	positive	neg	neg
G 5 / M High 0.075 mg/L	5GPH	Yellow	Clear	1+	negative	1+	neg
	5ZOF	Yellow	Clear	3+	negative	neg	neg
	5ZTF	Yellow	Clear	3+	negative	neg	neg

Dose Group	Animal Number	KET [-] day 1	UBG [-] day 1	BIL [-] day 1	ERY [-] day 1	Casts [-] day 1	EP Cells [-] day 1
G 1 / M Saline	1EZF	neg	neg	neg	1+	0	0
	1FUH	neg	1+	neg	2+	0	0
	1UYF	neg	neg	neg	neg	0	0
G 2 / M Vehicle	2AGF	neg	1+	neg	1+	0	0
	2BSF	1+	1+	neg	2+	0 - 2	1 - 3
	2FRH	neg	1+	neg	1+	0	0
G 3 / M Low 0.019 mg/L	3DWH	1+	1+	neg	1+	3 - 5	1 - 3
	3THF	neg	neg	neg	2+	0	0
	3TYF	neg	neg	neg	2+	3 - 5	0
G 4 / M Mid 0.038 mg/L	4EAH	neg	neg	neg	2+	0	1 - 3
	4JLH	neg	neg	neg	1+	0	0
	4YJF	neg	neg	neg	1+	0	1 - 3
G 5 / M High 0.075 mg/L	5GPH	neg	neg	neg	1+	0 - 2	0
	5ZOF	neg	neg	neg	1+	0	0
	5ZTF	neg	neg	neg	2+	0	0

Dose Group	Animal Number	PO4 [-] day 1	NMR [-] day 1	MR [-] day 1	RBC [-] day 1	WBC [-] day 1	Sperm [-] day 1
G 1 / M Saline	1EZF	0	3+	0	0 - 2	0 - 3	0
	1FUH	0	2+	0	0 - 2	31 - 50	1+
	1UYF	0	0	0	0 - 2	0 - 3	0
G 2 / M Vehicle	2AGF	0	0	0	0 - 2	0 - 3	2+
	2BSF	0	1+	0	3 - 10	31 - 50	0
	2FRH	0	1+	1+	3 - 10	31 - 50	1+
G 3 / M Low 0.019 mg/L	3DWH	0	3+	0	0 - 2	4 - 12	0
	3THF	0	1+	0	0 - 2	13 - 30	1+
	3TYF		2+	0	0 - 2	31 - 50	1+
G 4 / M Mid 0.038 mg/L	4EAH	0	1+	0	0 - 2	0 - 3	0
	4JLH	0	2+	0	0 - 2	0 - 3	0
	4YJF	0	3+	1+	0 - 2	4 - 12	3+
G 5 / M High 0.075 mg/L	5GPH	0	0	0	0 - 2	4 - 12	1+
	5ZOF	0	2+	1+	0 - 2	13 - 30	0
	5ZTF	0	3+	2+	0 - 2	0 - 3	0

NOTE: Pre-test urine samples were collected prior to the first exposure on Day 1.

Table F-8 – Individual Animal Urinalysis Data

PRE-TEST (FEMALE)

Dose Group	Animal Number	Refractive Index [-] day 1	SG [-] day 1	pH [-] day 1	Volume [mL] day 1
G 1 / F Saline	1CBE	1.3516	1.045	6.0	12.0
	1JKG	1.3508	1.042	5.0	11.0
	1JSG	1.3466	1.033	6.0	11.0
	Mean	1.3497	1.040	5.7	11.3
	S.D.	0.00269	0.0062	0.58	0.58
	N	3	3	3	3
G 2 / F Vehicle	2AJE	1.3488	1.038	5.0	11.0
	2ZQE	1.3416	1.020	5.0	11.0
	2FLE	1.3410	1.019	6.0	11.0
	Mean	1.3438	1.026	5.3	11.0
	S.D.	0.00434	0.0107	0.58	0.00
	N	3	3	3	3
G 3 / F Low 0.019 mg/L	3CVG	1.3504	1.040	5.0	10.5
	3FKE	1.3338	1.002	6.0	11.0
	3ZRE	1.3382	1.012	6.0	11.0
	Mean	1.3408	1.018	5.7	10.8
	S.D.	0.00860	0.0197	0.58	0.29
	N	3	3	3	3
G 4 / F Mid 0.038 mg/L	4ACE	1.3448	1.028	6.0	11.0
	4CAE	1.3504	1.040	6.0	11.0
	4EVE	1.3420	1.021	6.0	11.0
	Mean	1.3457	1.030	6.0	11.0
	S.D.	0.00428	0.0096	0.00	0.00
	N	3	3	3	3
G 5 / F High 0.075 mg/L	5AIE	1.3384	1.013	6.0	11.0
	5CGE	1.3428	1.023	6.0	11.0
	5CSG	1.3410	1.019	6.0	11.0
	Mean	1.3407	1.018	6.0	11.0
	S.D.	0.00221	0.0050	0.00	0.00
	N	3	3	3	3

NOTE: Pre-test urine samples were collected prior to the first exposure on Day 1.

Table F-8 – Individual Animal Urinalysis Data

PRE-TEST (FEMALE)

Dose Group	Animal Number	COL [-] day 1	CLA [-] day 1	LEU [-] day 1	NIT [-] day 1	PRO [-] day 1	GLU [-] day 1
G 1 / F Saline	1CBE	Yellow	Clear	2+	negative	2+	neg
	1JKG	Yellow	Clear	1+	negative	neg	neg
	1JSG	Yellow	Clear	1+	negative	1+	neg
G 2 / F Vehicle	2AJE	Yellow	Clear	1+	negative	1+	neg
	2ZQE	Yellow	Clear	neg	negative	neg	neg
	2FLE	Yellow	Clear	neg	negative	neg	neg
G 3 / F Low 0.019 mg/L	3CVG	Yellow	Clear	3+	negative	1+	neg
	3FKE	Yellow	Clear	3+	negative	neg	neg
	3ZRE	Yellow	Clear	neg	negative	neg	neg
G 4 / F Mid 0.038 mg/L	4ACE	Yellow	Clear	neg	negative	neg	neg
	4CAE	Yellow	Clear	neg	negative	1+	neg
	4EVE	Yellow	Sl Cloudy	2+	negative	1+	neg
G 5 / F High 0.075 mg/L	5AIE	Yellow	Clear	1+	negative	neg	neg
	5CGE	Yellow	Clear	1+	negative	neg	neg
	5CSG	Yellow	Clear	neg	negative	neg	neg

Dose Group	Animal Number	KET [-] day 1	UBG [-] day 1	BIL [-] day 1	ERY [-] day 1	Casts [-] day 1	EP Cells [-] day 1
G 1 / F Saline	1CBE	neg	1+	neg	1+	0	0
	1JKG	neg	neg	neg	1+	0	3 - 6
	1JSG	neg	neg	neg	neg	0	0
G 2 / F Vehicle	2AJE	neg	neg	neg	2+	0	0
	2ZQE	neg	neg	neg	2+	0	0
	2FLE	neg	neg	neg	neg	0	0
G 3 / F Low 0.019 mg/L	3CVG	neg	neg	neg	1+	3 - 5	0
	3FKE	neg	neg	neg	2+	0	0
	3ZRE	neg	neg	neg	neg	0	0
G 4 / F Mid 0.038 mg/L	4ACE	neg	neg	neg	neg	0	0
	4CAE	neg	neg	neg	neg	0 - 2	0
	4EVE	neg	neg	neg	4+	3 - 5	0
G 5 / F High 0.075 mg/L	5AIE	neg	neg	neg	1+	0	0
	5CGE	neg	neg	neg	1+	0 - 2	0
	5CSG	neg	neg	neg	neg	0	0

Dose Group	Animal Number	PO4 [-] day 1	NMR [-] day 1	MR [-] day 1	RBC [-] day 1	WBC [-] day 1
G 1 / F Saline	1CBE	0	1+	0	0 - 2	31 - 50
	1JKG	0	1+	0	0 - 2	4 - 12
	1JSG	1+	1+	0	0 - 2	0 - 3
G 2 / F Vehicle	2AJE	0	2+	0	0 - 2	0 - 3
	2ZQE	0	0	0	0 - 2	0 - 3
	2FLE	0	0	1+	0 - 2	4 - 12
G 3 / F Low 0.019 mg/L	3CVG	0	0	0	0 - 2	13 - 30
	3FKE	0	3+	0	0 - 2	0 - 3
	3ZRE	0	0	0	0 - 2	0 - 3
G 4 / F Mid 0.038 mg/L	4ACE	0	1+	0	0 - 2	0 - 3
	4CAE	0	1+	0	0 - 2	4 - 12
	4EVE	0	4+	0	0 - 2	0 - 3
G 5 / F High 0.075 mg/L	5AIE	0	3+	1+	0 - 2	0 - 3
	5CGE	0	3+	0	0 - 2	4 - 12
	5CSG	0	0	0	0 - 2	0 - 3

NOTE: Pre-test urine samples were collected prior to the first exposure on Day 1.

Table F-8 – Individual Animal Urinalysis Data

DAY 15 (MALE)

Dose Group	Animal Number	Refractive Index [-] day 15	SG [-] day 15	pH [-] day 15	Volume [mL] day 15
G 1 / M Saline	1EZF	1.3518	1.045	8.0	4.5
	1FUH	1.3508	1.042	6.0	6.0
	1UYF	1.3530	1.047	5.0	0.5
	Mean	1.3519	1.045	6.3	3.7
	S.D.	0.00110	0.0025	1.53	2.84
	N	3	3	3	3
G 2 / M Vehicle	2AGF	1.3500	1.040	6.0	2.5
	2BSF	1.3516	1.045	6.0	11.0
	2FRH	1.3504	1.040	6.0	5.0
	Mean	1.3507	1.042	6.0	6.2
	S.D.	0.00083	0.0029	0.00	4.37
	N	3	3	3	3
G 3 / M Low 0.019 mg/L	3DWH	1.3440	1.026	8.0	11.0
	3THF	1.3532	1.047	6.0	9.5
	3TYF	1.3500	1.040	7.0	5.0
	Mean	1.3491	1.038	7.0	8.5
	S.D.	0.00467	0.0107	1.00	3.12
	N	3	3	3	3
G 4 / M Mid 0.038 mg/L	4EAH	1.3376	1.010	7.0	7.0
	4JLH	1.3486	1.038	6.0	6.0
	4YJF	1.3380	1.012	7.0	10.0
	Mean	1.3414	1.020	6.7	7.7
	S.D.	0.00624	0.0156	0.58	2.08
	N	3	3	3	3
G 5 / M High 0.075 mg/L	5GPH	1.3490	1.038	6.0	4.0
	5ZOF	1.3504	1.040	6.0	3.5
	5ZTF	1.3430	1.023	6.0	6.5
	Mean	1.3475	1.034	6.0	4.7
	S.D.	0.00393	0.0093	0.00	1.61
	N	3	3	3	3

Table F-8 – Individual Animal Urinalysis Data

DAY 15 (MALE)

Dose Group	Animal Number	COL [-] day 15	CLA [-] day 15	LEU [-] day 15	NIT [-] day 15	PRO [-] day 15	GLU [-] day 15
G 1 / M Saline	1EZF	Yellow	Clear	1+	negative	2+	neg
	1FUH	Yellow	Clear	1+	negative	2+	neg
	1UYF	Yellow	Clear	1+	positive	2+	neg
G 2 / M Vehicle	2AGF	Yellow	Clear	neg	negative	2+	neg
	2BSF	Yellow	Clear	1+	negative	2+	neg
	2FRH	Yellow	Clear	neg	negative	1+	neg
G 3 / M Low 0.019 mg/L	3DWH	Yellow	Clear	neg	negative	2+	neg
	3THF	Yellow	Clear	1+	negative	2+	neg
	3TYF	Yellow	Clear	1+	negative	2+	neg
G 4 / M Mid 0.038 mg/L	4EAH	Yellow	Clear	neg	negative	neg	neg
	4JLH	Yellow	Clear	neg	negative	2+	neg
	4YJF	Yellow	Clear	neg	negative	neg	neg
G 5 / M High 0.075 mg/L	5GPH	Yellow	Clear	neg	negative	2+	neg
	5ZOF	Yellow	Clear	1+	negative	2+	neg
	5ZTF	Yellow	Clear	1+	negative	2+	neg

Dose Group	Animal Number	KET [-] day 15	UBG [-] day 15	BIL [-] day 15	ERY [-] day 15	Casts [-] day 15	EP Cells [-] day 15
G 1 / M Saline	1EZF	neg	neg	neg	neg	3 - 5	0
	1FUH	neg	2+	1+	1+	0	1 - 3
	1UYF	1+	2+	1+	5+	0	10 - 20
G 2 / M Vehicle	2AGF	neg	2+	2+	4+	0	10 - 20
	2BSF	1+	2+	1+	1+	0	0
	2FRH	neg	1+	neg	1+	0 - 2	6 - 10
G 3 / M Low 0.019 mg/L	3DWH	neg	neg	1+	2+	0 - 2	3 - 6
	3THF	1+	2+	2+	1+	0	3 - 6
	3TYF	neg	neg	neg	neg	0 - 2	1 - 3
G 4 / M Mid 0.038 mg/L	4EAH	neg	neg	neg	neg	0	0
	4JLH	neg	1+	1+	neg	0 - 2	6 - 10
	4YJF	neg	neg	neg	neg	0	0
G 5 / M High 0.075 mg/L	5GPH	neg	2+	1+	1+	0	1 - 3
	5ZOF	1+	2+	1+	3+	3 - 5	10 - 20
	5ZTF	neg	1+	1+	1+	0	3 - 6

Dose Group	Animal Number	PO4 [-] day 15	NMR [-] day 15	MR [-] day 15	RBC [-] day 15	WBC [-] day 15	Sperm [-] day 15
G 1 / M Saline	1EZF	0	0	0	0 - 2	0 - 3	0
	1FUH	0	0	0	0 - 2	0 - 3	1+
	1UYF	0	0	0	11 - 30	0 - 3	0
G 2 / M Vehicle	2AGF	0	0	0	3 - 10	0 - 3	1+
	2BSF	0	0	0	0 - 2	0 - 3	0
	2FRH	0	0	0	0 - 2	0 - 3	0
G 3 / M Low 0.019 mg/L	3DWH	0	0	0	0 - 2	0 - 3	1+
	3THF	0	0	0	0 - 2	0 - 3	1+
	3TYF	0	0	0	0 - 2	0 - 3	1+
G 4 / M Mid 0.038 mg/L	4EAH	0	0	0	0 - 2	0 - 3	0
	4JLH	0	0	0	0 - 2	0 - 3	0
	4YJF	0	0	0	0 - 2	0 - 3	0
G 5 / M High 0.075 mg/L	5GPH	0	0	0	0 - 2	0 - 3	0
	5ZOF	0	0	0	11 - 30	0 - 3	0
	5ZTF	0	0	0	0 - 2	0 - 3	2+

Table F-8 – Individual Animal Urinalysis Data

DAY 15 (FEMALE)

Dose Group	Animal Number	Refractive Index [-] day 15	SG [-] day 15	pH [-] day 15	Volume [mL] day 15
G 1 / F Saline	1CBE	1.3480	1.035	6.5	0.2
	1JKG	1.3506	1.042	7.0	3.0
	1JSG	1.3520	1.045	6.0	4.0
	Mean	1.3502	1.041	6.5	2.4
	S.D.	0.00203	0.0051	0.50	1.94
	N	3	3	3	3
G 2 / F Vehicle	2AJE	1.3492	1.038	5.0	0.5
	2ZQE	1.3430	1.023	6.0	2.5
	2FLE	1.3458	1.030	6.0	2.0
	Mean	1.3460	1.030	5.7	1.7
	S.D.	0.00310	0.0075	0.58	1.04
	N	3	3	3	3
G 3 / F Low 0.019 mg/L	3CVG	1.3460	1.030	6.0	7.0
	3FKE	1.3414	1.020	7.0	5.0
	3ZRE	1.3490	1.038	6.5	0.5
	Mean	1.3455	1.029	6.5	4.2
	S.D.	0.00383	0.0090	0.50	3.33
	N	3	3	3	3
G 4 / F Mid 0.038 mg/L	4ACE	1.3500	1.040	6.5	5.5
	4CAE	1.3500	1.040	5.0	4.0
	4EVE	1.3424	1.022	6.0	5.5
	Mean	1.3475	1.034	5.8	5.0
	S.D.	0.00439	0.0104	0.76	0.87
	N	3	3	3	3
G 5 / F High 0.075 mg/L	5AIE	1.3450	1.028	QN	0.1
	5CGE	1.3594	1.062	7.0	0.2
	5CSG	1.3444	1.026	6.0	2.0
	Mean	1.3496	1.039	6.5	0.8
	S.D.	0.00849	0.0202	0.71	1.06
	N	3	3	2	3

QN = Quantity Not Sufficient

Table F-8 – Individual Animal Urinalysis Data

DAY 15 (FEMALE)

Dose Group	Animal Number	COL [-] day 15	CLA [-] day 15	LEU [-] day 15	NIT [-] day 15	PRO [-] day 15	GLU [-] day 15
G 1 / F Saline	1CBE	Yellow	Clear	1+	negative	neg	neg
	1JKG	Yellow	Clear	1+	negative	2+	neg
	1JSG	Yellow	Clear	neg	negative	2+	neg
G 2 / F Vehicle	2AJE	Yellow	Clear	1+	negative	2+	neg
	2ZQE	Yellow	Clear	neg	negative	neg	neg
	2FLE	Yellow	Clear	neg	negative	1+	neg
G 3 / F Low 0.019 mg/L	3CVG	Yellow	Clear	neg	negative	neg	neg
	3FKE	Yellow	Clear	neg	negative	neg	neg
	3ZRE	Yellow	Clear	1+	negative	1+	neg
G 4 / F Mid 0.038 mg/L	4ACE	Yellow	Clear	1+	negative	2+	neg
	4CAE	Yellow	Clear	neg	negative	1+	neg
	4EVE	Yellow	Clear	neg	negative	1+	neg
G 5 / F High 0.075 mg/L	5AIE	Yellow	Clear	QN	QN	QN	QN
	5CGE	Yellow	Clear	1+	negative	2+	neg
	5CSG	Yellow	Clear	neg	negative	1+	neg

Dose Group	Animal Number	KET [-] day 15	UBG [-] day 15	BIL [-] day 15	ERY [-] day 15	Casts [-] day 15	EP Cells [-] day 15
G 1 / F Saline	1CBE	1+	2+	1+	2+	0	0
	1JKG	neg	1+	neg	1+	0	0
	1JSG	neg	1+	neg	neg	0	0
G 2 / F Vehicle	2AJE	1+	1+	neg	2+	0	0
	2ZQE	neg	neg	neg	neg	0	0
	2FLE	neg	1+	neg	1+	0	1 - 3
G 3 / F Low 0.019 mg/L	3CVG	neg	1+	neg	neg	0	0
	3FKE	neg	neg	neg	neg	0	1 - 3
	3ZRE	1+	1+	neg	5+	0	1 - 3
G 4 / F Mid 0.038 mg/L	4ACE	1+	2+	1+	1+	0	0
	4CAE	neg	1+	neg	neg	0	0
	4EVE	neg	neg	neg	neg	0	1 - 3
G 5 / F High 0.075 mg/L	5AIE	QN	QN	QN	QN	QN	QN
	5CGE	2+	2+	neg	4+	0	1 - 3
	5CSG	neg	1+	neg	1+	0	0

Dose Group	Animal Number	PO4 [-] day 15	NMR [-] day 15	MR [-] day 15	RBC [-] day 15	WBC [-] day 15
G 1 / F Saline	1CBE	1+	0	0	0 - 2	0 - 3
	1JKG	0	0	0	0 - 2	0 - 3
	1JSG	0	0	0	0 - 2	0 - 3
G 2 / F Vehicle	2AJE	0	0	0	0 - 2	0 - 3
	2ZQE	0	0	0	0 - 2	0 - 3
	2FLE	0	0	0	0 - 2	0 - 3
G 3 / F Low 0.019 mg/L	3CVG	0	0	0	0 - 2	0 - 3
	3FKE	0	0	0	0 - 2	0 - 3
	3ZRE	2+	0	0	3 - 10	0 - 3
G 4 / F Mid 0.038 mg/L	4ACE	0	0	0	0 - 2	0 - 3
	4CAE	0	0	0	0 - 2	0 - 3
	4EVE	0	0	0	0 - 2	0 - 3
G 5 / F High 0.075 mg/L	5AIE	QN	QN	QN	QN	QN
	5CGE	0	0	0	11 - 30	0 - 3
	5CSG	0	0	0	0 - 2	0 - 3

QN = Quantity Not Sufficient

***Phase*: Serum Sample Analysis for Drug Level and Analysis of Toxicokinetic Parameters**	
***Phase Test Site*:**	***Contributing Scientist*:**
IIT Research Institute (IITRI)<br>10 West 35th Street<br>Chicago, IL 60616	Miguel Muzzio, Ph.D., M.B.A.<br>Manager, Analytical Chemistry Division

Appendix G – Serum Analysis and Toxicokinetic Report

TABLE OF CONTENTS

Page

CONTRIBUTING SCIENTIST REPORT SIGNATURE PAGE G-3

SUMMARY G-4

I. INTRODUCTION G-5

II. SERUM SAMPLES G-5

III. SERUM ANALYSIS MATERIALS AND METHODS G-6

IV. TOXICOKINETIC MATERIALS AND METHODS G-8

V. SERUM ANALYSIS RESULTS G-9

VI. TOXICOKINETICS RESULTS G-9

VII. TABLES G-11

Table 1. Overview of Serum Analysis Analytical Runs G-12

Table 2. Serum Analysis Instrument Operating Conditions G-13

Table 3a. APN01 Concentration in Dog Serum; Day 1 (Male) G-14

Table 3b. APN01 Concentration in Dog Serum; Day 1 (Female) G-15

Table 4a. APN01 Concentration in Dog Serum; Day 14 (Male) G-16

Table 4b. APN01 Concentration in Dog Serum; Day 14 (Female) G-17

Table 5. Serum Analysis Calibration Curve Parameters G-18

Table 6. Serum Analysis Calibration Standard Results G-18

Table 7. Serum Analysis QC Sample Results G-19

Table 8. Serum Analysis Sample Reanalysis Results G-20

Table 9. TK Abbreviations and Terms G-21

Table 10a. TK Parameter Analysis Results Summary (Male) G-22

Table 10b. TK Parameter Analysis Results Summary (Female) G-22

Table 11a. Individual Animal TK Parameter Analysis Results (Group 3 Males) G-23

Table 11b. Individual Animal TK Parameter Analysis Results (Group 3 Females) G-23

Table 12a. Individual Animal TK Parameter Analysis Results (Group 4 Males) G-24

Table 12b. Individual Animal TK Parameter Analysis Results (Group 4 Females) G-24

Table 13a. Individual Animal TK Parameter Analysis Results (Group 5 Males) G-25

Table 13b. Individual Animal TK Parameter Analysis Results (Group 5 Females) G-25

continued on following page

TABLE OF CONTENTS (cont.)

Page

VIII. FIGURES G-26

Figure 1. Serum Analysis Representative Standard Curve G-27

Figure 2a. APN01 Concentration in Dog Serum; Day 1 (Male) G-28

Figure 2b. APN01 Concentration in Dog Serum; Day 1 (Female) G-28

Figure 3a. APN01 Concentration in Dog Serum; Day 14 (Male) G-29

Figure 3b. APN01 Concentration in Dog Serum; Day 14 (Female) G-29

IX. REFERENCE STANDARD CERTIFICATE OF ANALYSIS G-30

CONTRIBUTING SCIENTIST REPORT SIGNATURE PAGE

Report Approval:

2021-04-20

Miguel Muzzio, PhD, MBA
Contributing Scientist
Manager, Analytical Chemistry Division
IIT Research Institute

Date

SUMMARY

Dog serum samples were analyzed for levels of APN01 using enzyme-linked immunosorbent assay (ELISA) according to a method qualified at IITRI (Project Number 2857003BAMV01, Method No. 201101). Individual animal serum drug concentrations at scheduled (nominal) blood sampling times from animals in Groups 3-5 were used to model TK parameters. Reported TK parameters are T_{max}, C_{max}, and AUC_{6hr}; terminal phase parameters [e.g., $t_{1/2}$] could not be calculated reliably for any of the profiles due to a lack of amenable data in the terminal phase of the profiles. Summary TK results are shown in the table below.

Group	Target Test Atmosphere APN01 Concentration (mg/L)	Estimated Mean Inhaled APN01 Dose (mg/kg)	Mean / *Median* Group TK Parameter			
			n	T_{max} (hr)	C_{max} (ng/mL)	AUC_{6hr} (hr*ng/mL)
**MALES**						
**Day 1**						
3	0.19	0.38	2	*1*	1.26	--
4	0.038	0.53	3	*2*	1.92	16.3
5	0.075	1.34	3	*1*	4.38	21.1
**Day 14**						
3	0.19	0.34	1	*1*	1.04	--
4	0.038	0.69	3	*0.5*	1.44	--
5	0.075	1.54	3	*1*	7.96	38.0
**FEMALES**						
**Day 1**						
3	0.19	0.33	3	*6*	3.63	20.1
4	0.038	0.54	2	*1*	1.64	--
5	0.075	1.49	3	*6*	11.5	54.0
**Day 14**						
3	0.19	0.27	2	*0.25*	3.73	13.5
4	0.038	0.73	2	*0.38*	2.26	9.87
5	0.075	1.63	3	*0.5*	7.50	28.9
NOTE: n = number of animals with measurable serum concentration at one more time points						

Systemic exposure [defined as serum levels of APN01 above the limit of quantitation (LOQ)] was very low in dogs in the low dose and mid dose groups on both Days 1 and 14; in both groups, serum levels of APN01 were below the LOQ (0.5 ng/mL) in most animals at most time points. In the high dose group, serum levels of APN01 on Days 1 and 14 were above the LOQ in 5 of 6 dogs at all time points after 0.5 hr. Although interanimal variability was substantial, mean C_{max} (pooled across both sexes) in the high dose group was approximately 8 ng/mL on both Day 1 and Day 14.

I. INTRODUCTION

APN01 is a recombinant soluble human angiotensin converting enzyme 2 (ACE2) being developed as an inhibitor of SARS-CoV-2 infection and its associated disease, COVID-19. This study (IITRI Project Number 285700300102) was performed to provide a comprehensive evaluation of the toxicity of twice daily inhalation administration of APN01 aerosols to dogs for 14 consecutive days. Goals of this study included characterization of serum levels and toxicokinetics (TK) of inhaled APN01 in dogs; this contributing scientist report describes these portions of the study. The study design is summarized below:

Group	Number of Dogs (M + F)	Agent	Number and Duration of Daily Exposures	Number of Exposure Days	Target APN01 Concentration in Test Atmosphere (mg/L)
1	3 + 3	Saline (Control)	2 x 60 minutes	14	0
2	3 + 3	Vehicle (Control)	2 x 60 minutes	14	0
3	3 + 3	APN01 – Low	2 x 60 minutes	14	0.019
4	3 + 3	APN01 – Mid	2 x 60 minutes	14	0.038
5	3 + 3	APN01 - High	2 x 60 minutes	14	0.075

II. SERUM SAMPLES

On Days 1 and 14, timed blood samples (approximately 3 mL) for serum drug level analysis were collected from each dog exposed to an APN01 aerosol. Blood samples were collected from the cephalic or jugular vein of each dog at the following time points:

- Prior to the first daily exposure (pretest)
- 15 and 30 minutes and 1, 2, 4 and 6 hours (Day 1) or 15 and 30 minutes and 1, 2, and 4-5 hours (Day 14) after the first daily exposure
- 24 hours after the first daily exposure (immediately prior to the first exposure on Day 2 or prior to necropsy on Day 15)

Samples were collected into CAT Serum Sep Clot Activator tubes (Greiner Bio-One North America, Monroe, NC). Following collection, blood was centrifuged and serum was collected and stored frozen at approximately -70°C. The serum samples were transferred from IITRI Toxicology personnel to Analytical Chemistry personnel as follows:

Date Collected	Date Transferred	Day	Number of Samples
2020-11-02 to 2020-11-04	2020-11-19	1	240
2020-11-15 to 2020-11-17	2020-11-19	14	240

Serum samples were analyzed at IITRI within the established stability period (see following section) using a commercial ELISA kit for quantitative detection of human ACE2 (see Section III). For the saline control and vehicle control groups (Groups 1 and 2, respectively), ELISA assays were performed only on serum samples collected at 1 hour post-exposure.

III. SERUM ANALYSIS MATERIALS AND METHODS

Serum samples were analyzed for levels of APN01 according to a method qualified at IITRI. Qualification work was performed under IITRI Project Number 2857003BAMV01, Method No. 201101 ["Analysis of APN01 in Dog Serum by Enzyme-Linked Immunosorbent Assay (ELISA)"]. As part of the qualification work, it was demonstrated that APN01 samples did not show signs of degradation for at least 42 days when stored at -70°C.

A. <u>ELISA Kit and Reference Standard</u>: Serum samples were analyzed for levels of APN01 using a commercial kit (Human ACE2 ELISA Kit PicoKine™; SKU EK0997; batch number 6431639429A1110; Boster Bio, Pleasanton, CA), which was received at IITRI on November 11, 2020, and was stored at 4°C. The kit contained the following components and materials:

- Anti-Human ACE2 pre-coated 96-well strip microplate (capture antibody: monoclonal antibody from mouse)
- Human ACE2 standard (provided in each kit, but not used for analysis)
- Human ACE2 biotinylated antibody (detection antibody: polyclonal antibody from goat)
- Avidin-biotin-peroxidase complex (ABC-HRP)
- Color developing reagent (TMB)
- Stop solution
- Sample diluent
- Antibody diluent
- Avidin-biotin-peroxidase diluent

The APN01 reference standard (ACE20620-B; Polymun Scientific, Klosterneuburg, Austria; retest date of 12/2020; purity via HPLC - 99.8%) was received at IITRI on October 8 and November 3, 2020, from Apeiron Biologics (Vienna, Austria), and was stored at 2-8°C. The APN01 reference standard was used as received for the preparation of calibration standards and quality control (QC) samples for the determination of APN01 in serum. A Certificate of Analysis for the reference standard is presented in Section IX.

B. Serum Sample Preparation: For the determination of APN01 in serum, 75 µL of each sample was added to a 1.5 mL low-binding tube and diluted with 75 µL of sample diluent (from the kit). For samples analyzed in duplicate (specimens collected from Study Groups 3, 4 and 5 at the 1 and 2 hour post-exposure time points), 150 µL of sample was diluted with 150 µL of sample diluent. The tubes were capped and vortexed for 2 minutes at 3000 rpm. Tubes were centrifuged for 5 minutes at 4000 rpm at 4°C to remove any bubbles formed during sample mixing.

If a sample required a 1:2 dilution, a 75 µL aliquot of sample and 75 µL of blank dog serum was diluted with 150 µL of sample diluent. For 1:4 dilution samples, 50 µL of each sample was diluted with 150 µL of blank serum and then 200 µL of sample diluent. Blank dog serum was obtained from BioIVT (Hicksville, NY) and was stored frozen at -20°C.

Blank serum samples were sub-aliquoted from bulk dog serum and processed as described above. Assay calibrators and QC samples were prepared by spiking dog serum with an APN01 working solution. Calibrator and QC samples were prepared in duplicate at the following concentrations for each analytical run and single 96-well strip microplate:

Type	Nominal APN01 Concentration (ng/mL)
Calibrator 1	0.500
Calibrator 2	0.750
Calibrator 3	1.00
Calibrator 4	3.00
Calibrator 5	3.50
Calibrator 6	5.00
Calibrator 7	6.00
QC Low	1.5
QC Mid	2.0
QC High	4.0

For all calibrators, QC samples, and study samples, 100 µL of the diluted serum sample with diluent was added to the appropriate number of strips of the ELISA kit microplate. The plate was covered with the provided plate sealer and incubated at 2-8°C overnight.

The plate was allowed to equilibrate to room temperature the following day. The plate was processed according to the assay protocol provided with each kit.

C. Analytical Equipment and Conditions: An overview of analytical runs is provided in Table 1. Samples were analyzed under instrument conditions as detailed in Table 2.

Calibration curves were calculated from the 4-parameter logistic (4PL) regression of the analyte responses vs. concentrations. Concentrations of analyte in the samples were determined using the optical density reading, the regression parameters of the calibration curves and any dilution factor.

IV. TOXICOKINETIC MATERIALS AND METHODS

Individual animal serum drug concentrations at scheduled (nominal) blood sampling times – with the time zero for the experiment translated to the initiation of the exposure – from animals in Groups 3-5 were used to model TK parameters. Modeling was performed with the noncompartmental model for extravascular administration using validated software (Phoenix WinNonlin, Version 8.1; Certara, Princeton, NJ). Estimated inhaled dose levels were calculated from APN01 test atmosphere concentration, exposure time, and group mean animal weight per gender per Study Day [Bide, R.W., Armour, S.J. and Yee, E. (2000), Allometric respiration/body mass data for animals to be used for estimates of inhalation toxicity to young adult humans. J. Appl. Toxicol., 20: 273-290].

Area under the serum concentration-time curve (AUC) values were calculated by the linear-up/log-down trapezoidal rule. When allowed by the data, elimination rate constant values (λ_z) were calculated by log-linear regression on data points of the terminal phase (using Phoenix WinNonlin's Best Fit Lambda Z Calculation Method option), with elimination half-life ($t_{1/2}$) calculated as $\ln(2)/\lambda_z$. However, terminal phase parameters [e.g., $t_{1/2}$ (half-life)] could not be calculated reliably for any of the profiles due to a lack of amenable data in the terminal phase of the profiles. The reported TK parameters are T_{max} [time of observed maximum (peak) serum concentration post exposure], C_{max} (maximum observed serum concentration), and AUC_{6hr} (area under the serum concentration-time curve from the beginning of the exposure to the 6-hour post exposure time point). TK parameter abbreviations and terms are defined in Table 9.

V. SERUM ANALYSIS RESULTS

A. Serum Analysis: Serum APN01 concentration determinations are presented in Table 3a/Table 3b (Day 1, male/female) and Table 4a/Table 4b (Day 14; male/female). The mean value was reported for samples analyzed in duplicate (Groups 3, 4 and 5 at the 1 and 2 hour time-points) when both measurements were performed above the lowest limit of quantitation (0.5 ng/mL). Sample reanalysis results (including the reason for reanalysis) are reported in Table 8. Serum APN01 concentrations are shown graphically in Figure 2a/Figure 2b (Day 1, male/female) and Figure 3a/Figure 3b (Day 14; male/female).

B. Calibration and QC: Serum samples were analyzed with standard curves and at least two replicates at each QC level. A representative curve is shown in Figure 1. Parameters for the calibration curves are reported in Table 5. Calibrator results are presented in Table 6, and QC results are presented in Table 7.

The acceptable range for the back-calculated concentrations of all non-zero calibrators was 80-120%, except at the lower and upper limits of quantitation, where the acceptable range was 75-125%. The acceptable range for individual QC samples was 80-120% of nominal concentration. Analytical batches were evaluated using the applicable IITRI Standard Operating Procedures (a minimum of 6 non-zero calibration levels and with at least 75% of the calibration levels to be acceptable, and two-thirds of the QC samples from different concentration levels are within the acceptance range).

VI. TOXICOKINETICS RESULTS

Group TK modeling results summary are shown in Table 10a/Table 10b (male/female). Individual animal TK modeling results are shown in Table 11a/Table 11b, Table 12a/Table 12b and Table 13a/Table 13b (male/female) for animals in Study Groups 3, 4 and 5, respectively.

Following a single inhalation exposure (Study Day 1 after the first of two daily exposures) or twenty-seven 60-minute inhalation exposures (on Study Day 14 after the first of two daily exposures) to APN01 testing atmospheres at target concentrations of 0.019 (Group 3, Low), 0.038 (Group 4, Mid) or 0.075 (Group 5, High) mg/L to dogs, overall systemic exposure was low, with either no measurable exposure or sporadic exposure in many animals from the Low and Mid dose groups (mean C_{max} of 1 to 4 ng/mL). As would be expected, the highest

exposure was observed in animals from the High dose group (mean C_{max} of 4 to 12 ng/mL). Inter-animal variability was high, with animals in some groups presenting no measurable exposure (e.g., Group 4 female animal EVE had no measurable systemic exposure on either Day 1 or 14 and Group 5 male animal ZOF had only one measureable time point on Day 14), while others in the same group presented a more complete concentration vs. time profile. Specific TK parameter analysis results were as follows:

- **T_{max}** – The overall median time of observed maximum (peak) serum concentration from the end of the exposure was about 1 hour (across all dose levels and both genders and time points), but varied quite widely for the study animals (0.5 to 2 hours for males; 0.25 to 6 hours for females) and did not appear to change in a consistent manner with the dose level, study day or sex.
- **C_{max}** – Mean systemic exposure using serum peak concentration for both genders and study days was approximately 3 ng/mL (35 pM), 2 ng/mL (22 pM), and 8 ng/mL (89 pM) for the Low, Mid and High dose groups, respectively. C_{max} increased with the dose only for the High dose group and did not appear to change significantly with the study day or sex.
- **AUC_{6hr}** – Mean systemic exposure using AUC_{6hr} was approximately 17, 14 and 37 hr*ng/mL for the Low, Mid and High dose groups, respectively. AUC_{6hr} was larger for the High dose group and did not appear to change significantly with the study day or sex.

VII – TABLES

Table 1 – Overview of Serum Analysis Analytical Runs

Run Number	Analysis Date	Samples Analyzed	Pass/Fail
1	2020-11-25	Days 1 and 14; Groups 3 and 4 Males	Pass
2	2020-11-25	Days 1 and 14; Groups 3 and 4 Females	Pass
3	2020-11-28	Days 1 and 14; Groups 4 and 5 Males	Pass
4	2020-11-28	Days 1 and 14; Groups 4 and 5 Females	Pass
5	2020-12-01	Days 1 and 14; Group 5 Males and Females	Pass
6	2020-12-01	Days 1 and 14; Groups 1 and 2 Males and Females<br>Day 14; Group 5 Females	Fail
7	2020-12-04	Days 1 and 14; Groups 1 and 2 Males and Females<br>Days 1 and 14; Group 5 Females<br>Group 4 confirmatory samples	Pass
8	2020-12-08	Days 1 and 14; Group 5 Females<br>Above Quantitation Limit (AQL) reanalysis	Pass

Table 2 – Serum Analysis Instrument Operating Conditions

**System:**	SpectraMax® M2 Microplate Reader (Molecular Devices; San Jose, CA)
**Read Mode:**	Absorbance
**Read Type:**	Endpoint
**Wavelength 1 (nm):**	450
**Wavelength 2 (nm):**	540
**Temperature:**	Room Temperature
**Data System:**	SoftMax Pro GxP version 7.0.3

Table 3a – APN01 Concentration in Dog Serum; Day 1 (Male)

Group	Target Test Atmosphere APN01 Concentration (mg/L)	Animal ID	Serum Collection Time Point							
			Pre-Exposure	Hours Post-Exposure						
				0.25	0.5	1	2	4	6	24
			APN01 Concentration (ng/mL)							
1	0 (Saline Control)	EZF	x	x	x	BQL	x	x	x	x
		FUH	x	x	x	BQL	x	x	x	x
		UYF	x	x	x	BQL	x	x	x	x
		Average:	**-**	**-**	**-**	**BQL**	**-**	**-**	**-**	**-**
2	0 (Vehicle Control)	AGF	x	x	x	BQL	x	x	x	x
		BSF	x	x	x	BQL	x	x	x	x
		FRH	x	x	x	BQL	x	x	x	x
		Average:	**-**	**-**	**-**	**BQL**	**-**	**-**	**-**	**-**
3	0.019	DWH	BQL	BQL	BQL	BQL	BQL	BQL	BQL	BQL
		THF	BQL	BQL	BQL	BQL	BQL	BQL	BQL	3.20
		TYF	BQL	BQL	BQL	1.26	BQL	BQL	BQL	2.85
		Average:	**BQL**	**BQL**	**BQL**	**1.26**	**BQL**	**BQL**	**BQL**	**3.03**
		STD:	--	--	--	--	--	--	--	0.25
		%RSD:	--	--	--	--	--	--	--	8.2
4	0.038	EAH	BQL	BQL	BQL	BQL	1.05	BQL	BQL	3.42
		JLH	BQL	1.34	1.09	1.07	BQL	BQL	BQL	2.72
		YJF	BQL	BQL	1.71	2.13	2.38	3.05	3.37	6.98
		Average:	**BQL**	**1.34**	**1.40**	**1.60**	**1.72**	**3.05**	**3.37**	**4.37**
		STD:	--	--	0.44	0.75	0.94	--	--	2.3
		%RSD:	--	--	31	47	55	--	--	52
5	0.075	GPH	BQL	2.68	4.47	4.48	2.89	3.18	2.54	5.49
		ZOF	BQL	BQL	BQL	1.10	1.63	2.34	1.79	7.53
		ZTF	BQL	3.44	6.31	4.79	6.31	4.34	6.00	11.9
		Average:	**BQL**	**3.06**	**5.39**	**3.46**	**3.61**	**3.29**	**3.44**	**8.31**
		STD:	--	0.54	1.3	2.0	2.4	1.0	2.2	3.3
		%RSD:	--	18	24	59	67	31	65	39

Notes:

[1] BQL = below quantitation limit (0.5 ng/mL)

[2] x = Analysis not performed at given time point; see Protocol Deviation No. 1 in Appendix A.

Table 3b – APN01 Concentration in Dog Serum; Day 1 (Female)

Group	Target Test Atmosphere APN01 Concentration (mg/L)	Animal ID	Serum Collection Time Point							
			Pre-Exposure	Hours Post-Exposure						
				0.25	0.5	1	2	4	6	24
			APN01 Concentration (ng/mL)							
1	0 (Saline Control)	CBE	x	x	x	BQL	x	x	x	x
		JKG	x	x	x	BQL	x	x	x	x
		JSG	x	x	x	BQL	x	x	x	x
		Average:	**-**	**-**	**-**	**BQL**	**-**	**-**	**-**	**-**
2	0 (Vehicle Control)	AJE	x	x	x	BQL	x	x	x	x
		ZQE	x	x	x	BQL	x	x	x	x
		FLE	x	x	x	BQL	x	x	x	x
		Average:	**-**	**-**	**-**	**BQL**	**-**	**-**	**-**	**-**
3	0.019	CVG	BQL	BQL	BQL	BQL	BQL	BQL	BQL	1.51
		FKE	BQL	BQL	BQL	BQL	BQL	BQL	BQL	2.53
		ZRE	BQL	2.70	3.28	3.21	2.98	3.16	3.63	2.87
		Average:	**BQL**	**2.70**	**3.28**	**3.21**	**2.98**	**3.16**	**3.63**	**2.30**
		STD:	--	--	--	--	--	--	--	0.71
		%RSD:	--	--	--	--	--	--	--	31
4	0.038	ACE	BQL	BQL	BQL	BQL	BQL	BQL	BQL	1.41
		CAE	BQL	1.47	1.51	1.64	1.12	BQL	BQL	BQL
		EVE	BQL	BQL	BQL	BQL	BQL	BQL	BQL	BQL
		Average:	**BQL**	**1.47**	**1.51**	**1.64**	**1.12**	**BQL**	**BQL**	**1.41**
		STD:	--	--	--	--	--	--	--	--
		%RSD:	--	--	--	--	--	--	--	--
5	0.075	AIE	BQL	7.44	9.69	9.46	7.70	4.26	3.66	5.79
		CGE	BQL	4.14	4.10	5.30	6.07	5.76	7.04	39.3
		CSG	4.06	9.40	12.1	11.1	13.7	12.5	17.9	18.3
		Average:	**4.06**	**6.99**	**8.63**	**8.62**	**9.16**	**7.51**	**9.53**	**21.1**
		STD:	--	2.7	4.1	3.0	4.0	4.4	7.4	17
		%RSD:	--	38	48	35	44	58	78	80

Notes:

[1] BQL = below quantitation limit (0.5 ng/mL)

[2] x = Analysis not performed at given time point; see Protocol Deviation No. 1 in Appendix A.

Table 4a – APN01 Concentration in Dog Serum; Day 14 (Male)

Group	Target Test Atmosphere APN01 Concentration (mg/L)	Animal ID	Serum Collection Time Point							
			Pre-Exposure	Hours Post-Exposure						
				0.25	0.5	1	2	4	4-5	24
			APN01 Concentration (ng/mL)							
1	0 (Saline Control)	EZF	x	x	x	BQL	x	x	x	x
		FUH	x	x	x	BQL	x	x	x	x
		UYF	x	x	x	BQL	x	x	x	x
		Average:	**-**	**-**	**-**	**BQL**	**-**	**-**	**-**	**-**
2	0 (Vehicle Control)	AGF	x	x	x	BQL	x	x	x	x
		BSF	x	x	x	BQL	x	x	x	x
		FRH	x	x	x	BQL	x	x	x	x
		Average:	**-**	**-**	**-**	**BQL**	**-**	**-**	**-**	**-**
3	0.019	DWH	BQL	BQL	BQL	BQL	BQL	BQL	BQL	BQL
		THF	BQL	BQL	BQL	1.04	BQL	BQL	BQL	BQL
		TYF	BQL	BQL	BQL	BQL	BQL	BQL	BQL	BQL
		Average:	**BQL**	**BQL**	**BQL**	**1.04**	**BQL**	**BQL**	**BQL**	**BQL**
		STD:	--	--	--	--	--	--	--	--
		%RSD:	--	--	--	--	--	--	--	--
4	0.038	EAH	BQL	1.19	1.54	BQL	BQL	BQL	BQL	BQL
		JLH	BQL	1.17	1.31	1.14	BQL	BQL	BQL	BQL
		YJF	BQL	1.34	1.46	1.35	BQL	BQL	BQL	BQL
		Average:	**BQL**	**1.23**	**1.44**	**1.24**	**BQL**	**BQL**	**BQL**	**BQL**
		STD:	--	0.093	0.12	0.15	--	--	--	--
		%RSD:	--	7.5	8.1	12	--	--	--	--
5	0.075	GPH	BQL	8.33	12.4	11.9	8.23	3.80	3.84	BQL
		ZOF	BQL	BQL	BQL	1.27	BQL	BQL	BQL	BQL
		ZTF	2.24	7.11	8.74	8.32	10.2	5.21	5.39	3.62
		Average:	**2.24**	**7.72**	**10.6**	**7.16**	**9.22**	**4.51**	**4.62**	**3.62**
		STD:	--	0.86	2.6	5.4	1.4	1.0	1.1	--
		%RSD:	--	11	24	76	15	22	24	--

Notes:

[1] BQL = below quantitation limit (0.5 ng/mL)

[2] x = Analysis not performed at given time point; see Protocol Deviation No. 1 in Appendix A.

Table 4b – APN01 Concentration in Dog Serum; Day 14 (Female)

Group	Target Test Atmosphere APN01 Concentration (mg/L)	Animal ID	Serum Collection Time Point							
			Pre-Exposure	Hours Post-Exposure						
				0.25	0.5	1	2	4	4-5	24
			APN01 Concentration (ng/mL)							
1	0 (Saline Control)	CBE	x	x	x	BQL	x	x	x	x
		JKG	x	x	x	BQL	x	x	x	x
		JSG	x	x	x	BQL	x	x	x	x
		Average:	**-**	**-**	**-**	**BQL**	**-**	**-**	**-**	**-**
2	0 (Vehicle Control)	AJE	x	x	x	BQL	x	x	x	x
		ZQE	x	x	x	BQL	x	x	x	x
		FLE	x	x	x	BQL	x	x	x	x
		Average:	**-**	**-**	**-**	**BQL**	**-**	**-**	**-**	**-**
3	0.019	CVG	BQL	BQL	BQL	BQL	BQL	BQL	BQL	BQL
		FKE	BQL	1.65	BQL	1.00	1.33	BQL	BQL	BQL
		ZRE	BQL	5.81	4.83	3.54	2.19	BQL	BQL	BQL
		Average:	**BQL**	**3.73**	**4.83**	**2.27**	**1.76**	**BQL**	**BQL**	**BQL**
		STD:	--	2.9	--	1.8	0.61	--	--	--
		%RSD:	--	79	--	79	35	--	--	--
4	0.038	ACE	BQL	2.07	BQL	BQL	BQL	BQL	BQL	BQL
		CAE	BQL	BQL	2.44	2.11	1.98	1.45	1.46	1.42
		EVE	BQL	BQL	BQL	BQL	BQL	BQL	BQL	BQL
		Average:	**BQL**	**2.07**	**2.44**	**2.11**	**1.98**	**1.45**	**1.46**	**1.42**
		STD:	--	--	--	--	--	--	--	--
		%RSD:	--	--	--	--	--	--	--	--
5	0.075	AIE	BQL	4.74	5.54	5.50	4.80	1.31	2.81	2.40
		CGE	1.69	5.98	7.90	8.53	7.54	4.34	4.68	1.04
		CSG	5.39	8.44	8.37	5.36	5.49	3.51	4.31	4.25
		Average:	**3.54**	**6.39**	**7.27**	**6.46**	**5.94**	**3.05**	**3.93**	**2.56**
		STD:	2.6	1.9	1.5	1.8	1.4	1.6	1.0	1.6
		%RSD:	74	29	21	28	24	51	25	63

Notes:
[1] BQL = below quantitation limit (0.5 ng/mL)
[2] x = Analysis not performed at given time point; see Protocol Deviation No. 1 in Appendix A.

Table 5 – Serum Analysis Calibration Curve Parameters

Run Number	4-Parameter Logistic (D+[(A-D)/1+(x/C)^B])				
	A	B	C	D	R^2
**1**	-0.042	0.945	6.24E+03	2.22E+00	**0.999**
**2**	0.079	1.615	3.29E+03	1.29E+00	**0.988**
**3**	-0.135	0.591	1.15E+12	8.15E+04	**0.996**
**4**	0.005	1.065	3.87E+04	8.386	**0.999**
**5**	0.032	0.913	5.68E+09	2.73E+05	**0.992**
**7**	0.006	1.284	3.19E+03	9.39E-01	**0.996**
**8**	-0.043	0.663	3.22E+11	8.73E+04	**0.999**

Table 6 – Serum Analysis Calibration Standard Results

Run	APN01 Calibrator Concentration (ng/mL)							
	**0.500**	**0.750**	**1.00**	**3.00**	**3.50**	**5.00**	**6.00**	**RESULT**
1	0.517	0.718	1.01	3.09	3.46	4.91	6.12	pass
**Accuracy (%):**	**103**	**96.0**	**101**	**103**	**99.1**	**98.2**	**102**	
2	0.467	0.679	1.13	2.89	3.37	5.97	5.43	pass
**Accuracy (%):**	**93.4**	**90.6**	**113**	**96.5**	**96.3**	**119**	**90.6**	
3	0.502	0.745	0.989	3.15	3.48	4.67	6.25	pass
**Accuracy (%):**	**100**	**99.1**	**99.1**	**105**	**99.8**	**93.4**	**104**	
4	0.539	0.708	0.987	3.01	3.55	4.91	6.04	pass
**Accuracy (%):**	**108**	**94.4**	**98.7**	**100**	**101**	**98.4**	**101**	
5	0.556	0.700	0.932	3.34	3.31	4.76	6.16	pass
**Accuracy (%):**	**111**	**93.3**	**93.2**	**112**	**94.5**	**95.1**	**103**	
7	0.549	0.625	1.08	3.10	3.36	5.14	5.95	pass
**Accuracy (%):**	**110**	**83.3**	**109**	**104**	**95.9**	**103**	**98.9**	
8	0.479	0.751	1.05	2.98	3.52	4.91	6.07	pass
**Accuracy (%):**	**96.0**	**100**	**105**	**99.4**	**100**	**98.4**	**101**	
**Average:**	**103**	**93.8**	**103**	**103**	**98.2**	**101**	**100**	
**St.Dev:**	**6.9**	**5.7**	**6.7**	**4.8**	**2.6**	**8.6**	**4.5**	
**%RSD:**	**6.7**	**6.1**	**6.5**	**4.7**	**2.6**	**8.5**	**4.5**	

Table 7 – Serum Analysis QC Sample Results

**Run Number**	**APN01 QC Concentration**						
	**Low (1.5 ng/mL)**		**Mid (2.0 ng/mL)**		**High (4.0 ng/mL)**		**RESULT**
	**ng/mL**	**% Accuracy**	**ng/mL**	**% Accuracy**	**ng/mL**	**% Accuracy**	
1	1.43	**95.6**	1.55	**77.5**	4.27	**107**	pass
	1.15	**76.6**	1.68	**84.2**	4.40	**110**	
2	1.43	**95.5**	2.28	**114**	4.41	**110**	pass
	1.33	**88.8**	1.85	**92.6**	4.21	**105**	
3	1.84	**122**	1.98	**99.1**	4.20	**105**	pass
	1.63	**109**	2.03	**101**	4.72	**118**	
4	1.80	**120**	2.06	**103**	4.08	**102**	pass
	2.12	**141**	2.41	**120**	4.13	**103**	
5	1.63	**109**	2.74	**137**	3.96	**99.1**	pass
	1.61	**108**	1.95	**97.6**	4.89	**122**	
7	1.52	**101**	1.71	**85.7**	3.71	**92.8**	pass
	1.43	**95.5**	1.91	**95.7**	4.56	**114**	
8	1.64	**110**	2.02	**101**	4.46	**111**	pass
	1.45	**96.8**	1.50	**75.2**	4.75	**119**	
**Average:**	**105**		**98.8**		**108**		
**St.Dev:**	16		17		8.1		
**%RSD:**	15		17		7.5		

Table 8 – Serum Analysis Sample Reanalysis Results

Sample ID	Rationale	Initial Run	Reanalysis Run	Original Result (ng/mL)	Reanalysis Result (ng/mL)	Reported
1CBE D1 1 hr	F	6	7	NA	BQL	Reanalysis
1CBE D14 1 hr	F	6	7	NA	BQL	Reanalysis
1EZF D1 1 hr	F	6	7	NA	BQL	Reanalysis
1EZF D14 1 hr	F	6	7	NA	BQL	Reanalysis
1FUH D1 1 hr	F	6	7	NA	BQL	Reanalysis
1FUH D14 1 hr	F	6	7	NA	BQL	Reanalysis
1JKG D1 1 hr	F	6	7	NA	BQL	Reanalysis
1JKG D14 1 hr	F	6	7	NA	BQL	Reanalysis
1JSG D1 1 hr	F	6	7	NA	BQL	Reanalysis
1JSG D14 1 hr	F	6	7	NA	BQL	Reanalysis
1UYF D1 1 hr	F	6	7	NA	BQL	Reanalysis
1UYF D14 1 hr	F	6	7	NA	BQL	Reanalysis
2AGF D1 1 hr	F	6	7	NA	BQL	Reanalysis
2AGF D14 1 hr	F	6	7	NA	BQL	Reanalysis
2AJE D1 1 hr	F	6	7	NA	BQL	Reanalysis
2AJE D14 1 hr	F	6	7	NA	BQL	Reanalysis
2BSF D1 1 hr	F	6	7	NA	BQL	Reanalysis
2BSF D14 1 hr	F	6	7	NA	BQL	Reanalysis
2FLE D1 1 hr	F	6	7	NA	BQL	Reanalysis
2FLE D14 1 hr	F	6	7	NA	BQL	Reanalysis
2FRH D1 1 hr	F	6	7	NA	BQL	Reanalysis
2FRH D14 1 hr	F	6	7	NA	BQL	Reanalysis
2ZQE D1 1 hr	F	6	7	NA	BQL	Reanalysis
2ZQE D14 1 hr	F	6	7	NA	BQL	Reanalysis
5CSG D14 0 hr (Pre)	F	6	7	NA	5.39	Reanalysis
5CSG D14 0.25 hr (15 min)	F	6	7	NA	8.44	Reanalysis
5CSG D14 1 hr	F	6	7	NA	5.36	Reanalysis
5CSG D14 2 hr	F	6	7	NA	5.49	Reanalysis
5CSG D14 4 hr	F	6	7	NA	3.51	Reanalysis
5CSG D14 6 hr (2nd Exp)	F	6	7	NA	4.31	Reanalysis
5CSG D14 _24 hr	F	6	7	NA	4.25	Reanalysis
4EAH D1 6 hr (2nd Exp)	C	1	7	BQL	1.49	Original
4EAH D1 _24 hr	C	1	7	3.42	3.87	Original
4YJF D1 _24 hr	C	3	7	6.98	7.39	Original
5CGE D1 24 hr	AQL	5	8	19.2	39.3	Reanalysis
5CSG D1 0 hr (Pre)	C	5	7	4.06	4.44	Original
5CSG D1 0.25 hr (15 min)	C; AQL	5	7; 8	9.40	12.6 (AQL) ; 11.8	Original
5CSG D1 0.5 hr (30 min)	C	5	7	12.1	9.83	Original
5CSG D1 1 hr	AQL	5	7	13.2	11.1	Reanalysis
5CSG D1 2 hr	AQL	5	7	13.2	13.7	Reanalysis
5CSG D1 4 hr	AQL	5	7	13.6	12.5	Reanalysis
5CSG D1 6 hr (2nd Exp)	AQL	5	7	16.0	17.9	Reanalysis
5CSG D1 _24 hr	AQL	5	8	22.1	18.3	Reanalysis
5CSG D14 0.5 hr (30 min)	F; AQL	6	7; 8	NA	12.0 (AQL) ; 8.37	Reanalysis
5GPH D14 0.5 hr (30 min)	C	3	7	12.4	15.9	Original
5GPH D14 1 hr	C	3	7	11.6	11.9	Reanalysis
5ZTF D1 24 hr	C and AQL	5	7	11.9	15.0	Original
5ZTF D14 2 hr	C	5	7	10.2	11.5	Original

NOTE: AQL = above quantifiable limits (6 ng/mL); C = confirmatory; F = failed run; NA = not applicable

Table 9 – TK Abbreviations and Terms

**Parameter/Term**	**Definition**
λ_z	Elimination rate constant
AUC_{6hr}	Area under the serum concentration-time curve from the beginning of inhalation exposure to the 6-hour post exposure time point
C_{max}	Maximum (peak) observed serum drug concentration
hr(s)	hour(s)
pM	picomolar
$t_{1/2}$	Elimination half-life, determined by $\ln(2)/\lambda_z$
TK	toxicokinetics
T_{max}	Time of observed maximum (peak) serum concentration post exposure

Table 10a – TK Parameter Analysis Results Summary (Male)

Group	Target Test Atmosphere APN01 Concentration (mg/L)	Estimated Mean Inhaled APN01 Dose (mg/kg)	Mean / *Median* Group TK Parameter			
			n	T_{max} (hr)	C_{max} (ng/mL)	AUC_{6hr} (hr*ng/mL)
**Day 1**						
3	0.19	0.38	2	*1*	1.26	--
4	0.038	0.53	3	*2*	1.92	16.3
5	0.075	1.34	3	*1*	4.38	21.1
**Day 14**						
3	0.19	0.34	1	*1*	1.04	--
4	0.038	0.69	3	*0.5*	1.44	--
5	0.075	1.54	3	*1*	7.96	38.0
NOTE: n = number of animals with measurable serum concentration at one more time points (see Tables 3a and 4a).						

Table 10b – TK Parameter Analysis Results Summary (Female)

Group	Target Test Atmosphere APN01 Concentration (mg/L)	Estimated Mean Inhaled APN01 Dose (mg/kg)	Mean / *Median* Group TK Parameter			
			n	T_{max} (hr)	C_{max} (ng/mL)	AUC_{6hr} (hr*ng/mL)
**Day 1**						
3	0.19	0.33	3	*6*	3.63	20.1
4	0.038	0.54	2	*1*	1.64	--
5	0.075	1.49	3	*6*	11.5	54.0
**Day 14**						
3	0.19	0.27	2	*0.25*	3.73	13.5
4	0.038	0.73	2	*0.38*	2.26	9.87
5	0.075	1.63	3	*0.5*	7.50	28.9
NOTE: n = number of animals with measurable serum concentration at one more time points (see Tables 3b and 4b).						

Table 11a – Individual Animal TK Parameter Analysis Results (Group 3 Males)

Group	Target Test Atmosphere APN01 Concentration (mg/L)	Male Animal ID	Individual Animal TK Parameter			
			Estimated Mean Inhaled APN01 Dose (mg/kg)	T_{max} (hr)	C_{max} (ng/mL)	AUC_{6hr} (hr*ng/mL)
**Day 1**						
3	0.019	DWH	0.38	--	--	--
		THF		--	--	--
		TYF		1	1.26	--
		**Mean/ *Median*:**	--	***1***	**1.26**	--
		**STD:**	--	--	--	--
		**%RSD:**	--	--	--	--
**Day 14**						
3	0.019	DWH	0.34	--	--	--
		THF		1	1.04	--
		TYF		--	--	--
		**Mean/ *Median*:**	--	***1***	**1.04**	**--**
		**STD:**	--	--	--	--
		**%RSD:**	--	--	--	--

Table 11b – Individual Animal TK Parameter Analysis Results (Group 3 Females)

Group	Target Test Atmosphere APN01 Concentration (mg/L)	Female Animal ID	Individual Animal TK Parameter			
			Estimated Mean Inhaled APN01 Dose (mg/kg)	T_{max} (hr)	C_{max} (ng/mL)	AUC_{6hr} (hr*ng/mL)
**Day 1**						
3	0.019	CVG	0.33	--	--	--
		FKE		--	--	--
		ZRE		6	3.63	20.1
		**Mean/ *Median*:**	--	***6***	**3.63**	**20.1**
		**STD:**	--	--	--	--
		**%RSD:**	--	--	--	--
**Day 14**						
3	0.019	CVG	0.27	--	--	--
		FKE		0.25	1.65	--
		ZRE		0.25	5.81	13.5
		**Mean/ *Median*:**	--	***0.25***	**3.73**	**13.5**
		**STD:**	--	--	--	--
		**%RSD:**	--	--	--	--

Table 12a – Individual Animal TK Parameter Analysis Results (Group 4 Males)

Group	Target Test Atmosphere APN01 Concentration (mg/L)	Male Animal ID	Individual Animal TK Parameter			
			Estimated Mean Inhaled APN01 Dose (mg/kg)	T_{max} (hr)	C_{max} (ng/mL)	AUC_{6hr} (hr*ng/mL)
**Day 1**						
4	0.038	EAH	0.53	2	1.05	--
		JLH		0.25	1.34	--
		YJF		6	3.37	16.3
		**Mean/ *Median*:**	--	***2***	**1.92**	**16.3**
		**STD:**	--	--	1.3	--
		**%RSD:**	--	--	66	--
**Day 14**						
4	0.038	EAH	0.69	0.5	1.54	--
		JLH		0.5	1.31	--
		YJF		0.5	1.46	--
		**Mean/ *Median*:**	--	***0.5***	**1.44**	--
		**STD:**	--	--	0.12	--
		**%RSD:**	--	--	8.1	--

Table 12b – Individual Animal TK Parameter Analysis Results (Group 4 Females)

Group	Target Test Atmosphere APN01 Concentration (mg/L)	Female Animal ID	Individual Animal TK Parameter			
			Estimated Mean Inhaled APN01 Dose (mg/kg)	T_{max} (hr)	C_{max} (ng/mL)	AUC_{6hr} (hr*ng/mL)
**Day 1**						
4	0.038	ACE	0.54	--	--	--
		CAE		1	1.64	--
		EVE		--	--	--
		**Mean/ *Median*:**	--	***1***	**1.64**	--
		**STD:**	--	--	--	--
		**%RSD:**	--	--	--	--
**Day 14**						
4	0.038	ACE	0.73	0.25	2.07	--
		CAE		0.5	2.44	9.87
		EVE		--	--	--
		**Mean/ *Median*:**	--	***0.4***	**2.26**	**9.87**
		**STD:**	--	--	0.26	--
		**%RSD:**	--	--	12	--

Table 13a – Individual Animal TK Parameter Analysis Results (Group 5 Males)

Group	Target Test Atmosphere APN01 Concentration (mg/L)	Male Animal ID	Individual Animal TK Parameter			
			Estimated Mean Inhaled APN01 Dose (mg/kg)	T_{max} (hr)	C_{max} (ng/mL)	AUC_{6hr} (hr*ng/mL)
**Day 1**						
5	0.075	GPH	1.34	1	4.48	20.2
		ZOF		4	2.34	10.5
		ZTF		0.5	6.31	32.5
**Mean/ *Median*:**			--	***1***	**4.38**	**21.1**
**STD:**			--	--	2.0	11
**%RSD:**			--	--	45	52
**Day 14**						
5	0.075	GPH	1.54	0.5	12.4	37.2
		ZOF		1	1.27	--
		ZTF		2	10.2	38.9
**Mean/ *Median*:**			--	***1***	**7.96**	**38.0**
**STD:**			--	--	5.9	1.2
**%RSD:**			--	--	74	3.1

Table 13b – Individual Animal TK Parameter Analysis Results (Group 5 Females)

Group	Target Test Atmosphere APN01 Concentration (mg/L)	Female Animal ID	Individual Animal TK Parameter			
			Estimated Mean Inhaled APN01 Dose (mg/kg)	T_{max} (hr)	C_{max} (ng/mL)	AUC_{6hr} (hr*ng/mL)
**Day 1**						
5	0.075	AIE	1.49	0.5	9.69	39.7
		CGE		6	7.04	36.3
		CSG		6	17.9	85.9
**Mean/ *Median*:**			--	***6***	**11.5**	**54.0**
**STD:**			--	--	5.7	28
**%RSD:**			--	--	49	51
**Day 14**						
5	0.075	AIE	1.63	0.5	5.54	19.6
		CGE		1	8.53	34.8
		CSG		0.25	8.44	32.3
**Mean/ *Median*:**			--	***0.5***	**7.50**	**28.9**
**STD:**			--	--	1.7	8.1
**%RSD:**			--	--	23	28

VIII – FIGURES

Figure 1 – Serum Analysis Representative Standard Curve

(Analytical Run 4; Plate 2)

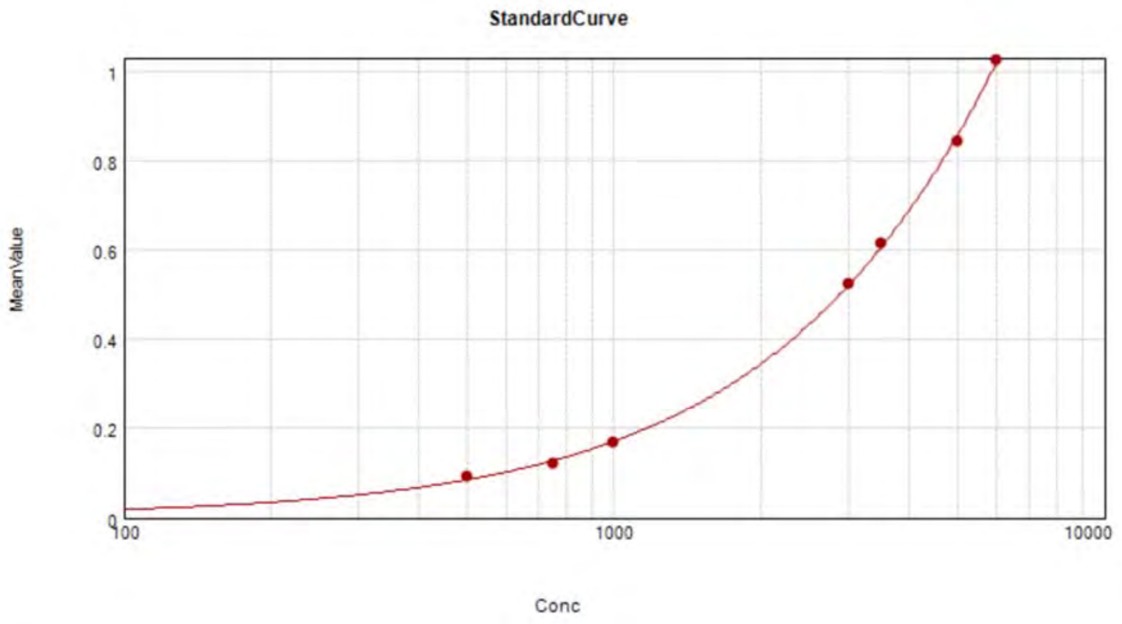

● **Std** (Standards: MeanValue vs Conc) Weighting: Fixed

Curve Fit Results ▲

Curve Fit : 4-Parameter Logistic $y = D + \frac{A - D}{1 + \left(\frac{x}{C}\right)^B}$

	Parameter	Estimated Value	Std. Error	Confidence Interval
**Std** $R^2 = 0.999$ EC50 = 3.87e+4	A	0.005	0.053	[-0.164, 0.173]
	B	1.065	0.327	[0.026, 2.104]
	C	3.87e+4	1.23e+5	[-3.53e+5, 4.31e+5]
	D	8.386	20.95	[-58.29, 75.06]

Figure 2a – APN01 Concentration in Dog Serum; Day 1 (Male)

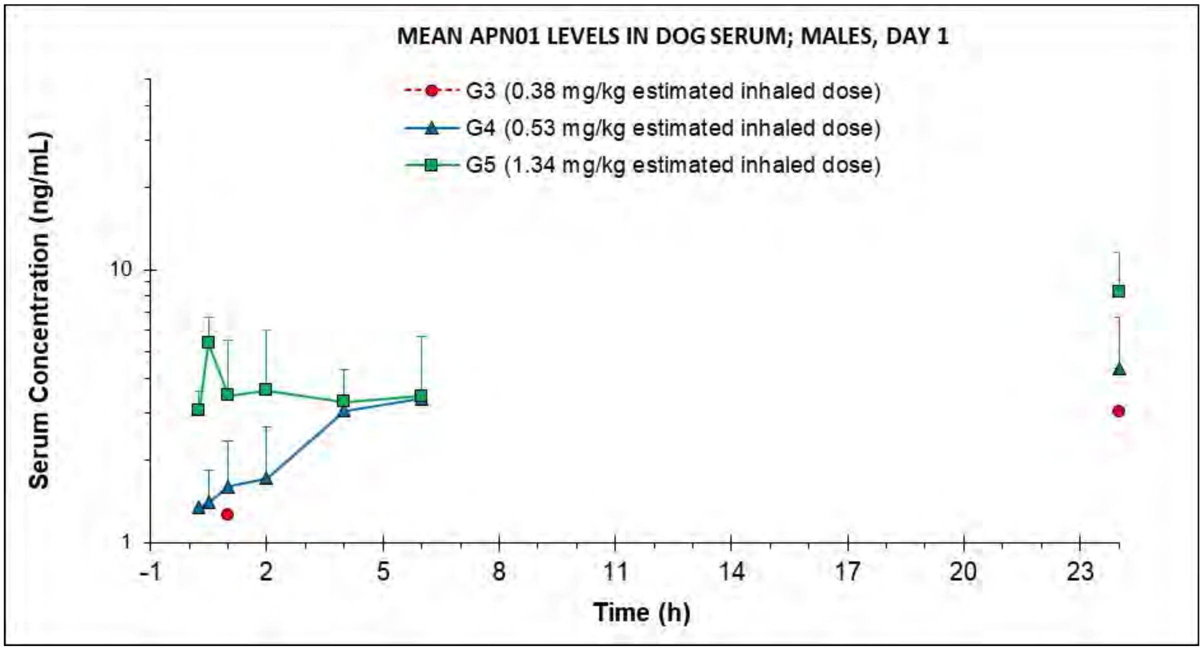

Figure 2b – APN01 Concentration in Dog Serum; Day 1 (Female)

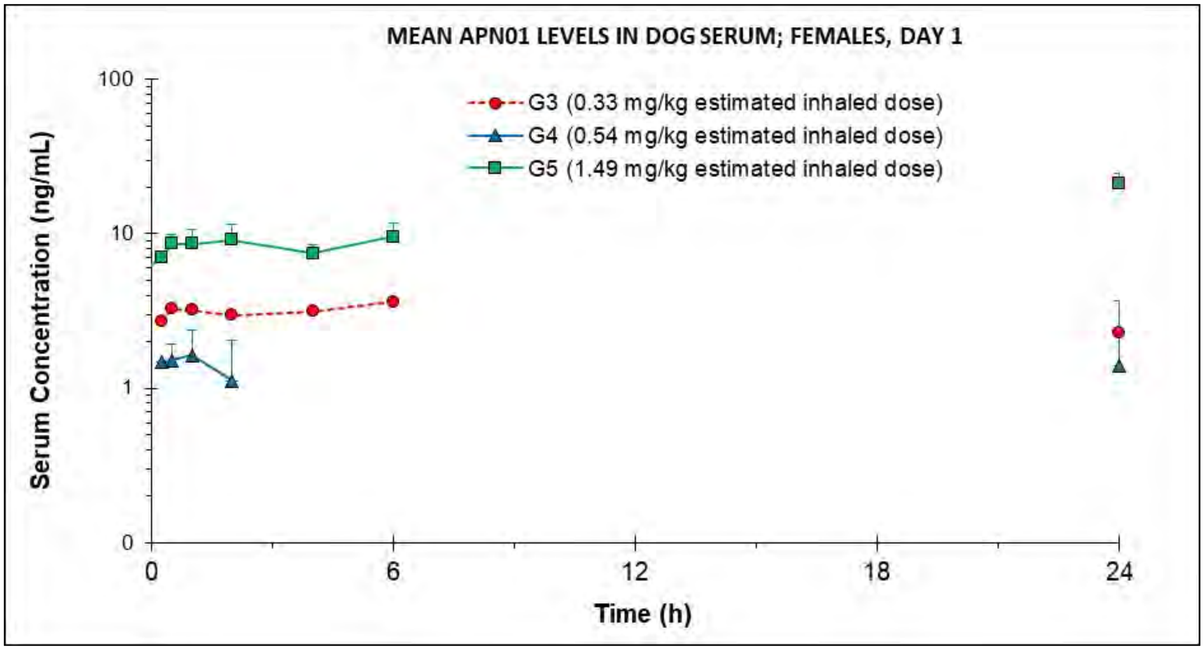

Figure 3a – APN01 Concentration in Dog Serum; Day 14 (Male)

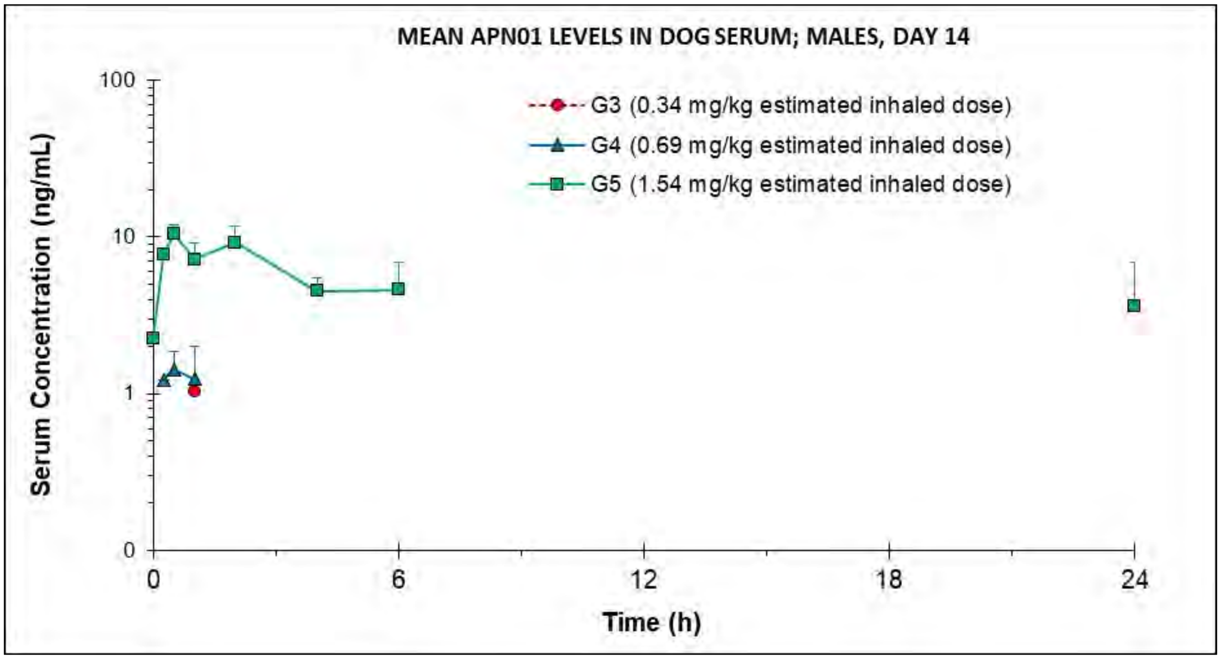

Figure 3b – APN01 Concentration in Dog Serum; Day 14 (Female)

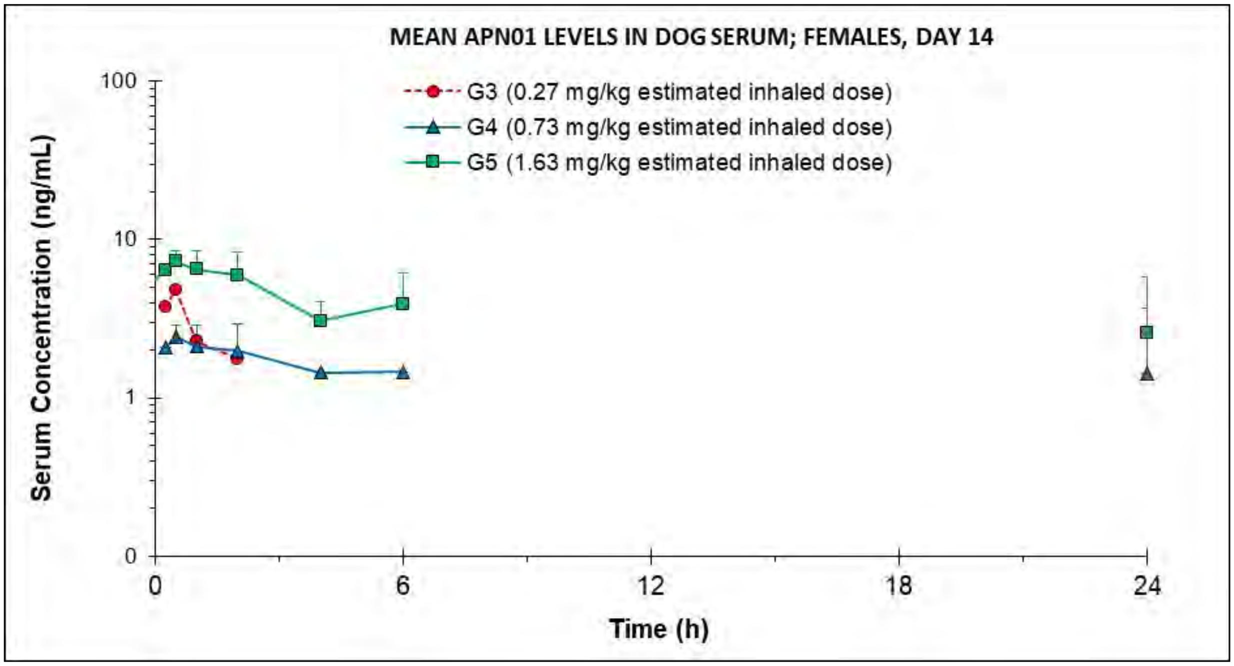

IX – REFERENCE STANDARD CERTIFICATE OF ANALYSIS

Reference Standard (APN01; ACE2 Drug Product) Certificate of Analysis (page 1 of 2)

POLYMUN SCIENTIFIC
Immunbiologische Forschung GmbH

A-3400 Klosterneuburg, Austria
Donaustraße 99
Tel.: +43-2243-25060-300
Fax: +43-2243-25060-399
E.Mail: office@polymun.com
http://www.polymun.com

CERTIFICATE OF ANALYSIS

**Product:** ACE2 Drug Product (5.0 mg/ml)	**Lot Number:** ACE20620-B
**Specification:** ACE2/SPEC/009-01	**Storage Conditions:** 5 ± 3 °C
**Date of Manufacture:** 09.06.2020	**Retest date:** 12/2020
**Site of Manufacture:** Polymun Scientific, Donaustraße 99, A-3400 Klosterneuburg, Austria	

Test Method Reference[1)]	Acceptance criteria	Result	Pass or Fail
**Appearance and Description**			
Visual appearance 224/SOP/011	clear and colourless	Clear, colourless	pass
Visible particles 224/SOP/011	practically free from visible particles	No visible particles	pass
**Identity**			
SDS-PAGE 223/SOP/002, 223/SOP/004	Protein band between 75 kD and 150 kD marker bands banding profile comparable to control standard	conforms	pass
Western Blot 223/SOP/002, 223/SOP/006	Protein band between 75 kD and 150 kD marker bands positive reaction of protein band with specific antibody banding profile comparable to control standard	conforms	pass
Native-PAGE 223/SOP/009, 223/SOP/004	Main band between 242 kD and 480 kD marker bands banding profile comparable to control standard	conforms	pass
Isoelectric Focussing 223/SOP/010, 223/SOP/004	Banding profile comparable to control standard bands between pI 4.2 and 5.3	conforms	pass
**Purity**			
Contents of dimers, di-dimers and polymers, monomers and fragments (SEC-HPLC) ACE2/SOP/007 [2)]	dimers ≥ 95.0 % di-dimers + polymers ≤ 2.0 % monomers ≤ 5.0 % fragments ≤ 2.0 % retention time of main peak comparable to that of control standard	dimers: 99.2 % di-dimers: 0.1 % monomers: 0.5 % fragments: 0.2 % retention time comparable to that of control standard	pass
QSDS-PAGE ACE2/SOP/001	Purity ≥ 95.0 %	98.9 %	pass
RP-HPLC ACE2/SOP/004	Main peak ≥ 90.0 % Retention time of main peak comparable to that of control standard	99.8 % retention time comparable to that of control standard	pass
**Quantity**			
ACE2 content (OD_{280}) 221/SOP/008	5.0 ± 0.5 mg/ml	4.9 mg/ml	pass
**Potency**			
Specific catalytic activity (HPLC) ACE2/SOP/003	0.025 – 0.075 µkat/mg	0.043 µkat/mg	pass

Reference Standard (APN01; ACE2 Drug Product) Certificate of Analysis (page 2 of 2)

POLYMUN SCIENTIFIC
Immunbiologische Forschung GmbH

A-3400 Klosterneuburg, Austria
Donaustraße 99
Tel.: +43-2243-25060-300
Fax: +43-2243-25060-399
E.Mail: office@polymun.com
http://www.polymun.com

Test Method Reference[1]	Acceptance criteria	Result	Pass or Fail
**Contaminants**			
Endotoxin (LAL-assay) 221/SOP/001	≤ 10.42 EU/ml	< 0.15 EU/ml	pass
Sterility [3] USP <71>, Ph.Eur2.6.1	sterile	sterile	pass
**General characteristics**			
pH 224/SOP/016	7.0 ± 0.5	7.0	pass
Osmolality 224/SOP/009	330 ± 50 mOsmol/kg	327 mOsmol/kg	pass
Sialic acid (SIA) content (bound) ACE2/SOP/005	6.0 – 17.0 mol SIA/mol ACE2	10.3 mol SIA/mol ACE2	pass
Particulate Matter 224/SOP/018	Particles ≥ 10 µm: ≤6000 particles / container Particles ≥ 25 µm: ≤600 particles / container	Particles ≥ 10 µm: 26 particles / container Particles ≥ 25 µm: 3 particles / container	pass
Extractable Volume 224/SOP/014	Extractable volume (ml) is not less than the stated nominal volume	4.2 ml	pass

1. Unless specified otherwise, tests were performed at Polymun Scientific, Donaustraße 99, A-3400 Klosterneuburg, Austria.
2. Tested according to version 02 of the referenced SOP.
3. Test was performed at AGES, Institut für Med. Mikrobiologie und Hygiene, Beethovenstraße 6, A-8010 Graz, Austria.

The material was tested in compliance with GMP and conforms to the specification ACE2/SPEC/009-01 and.

Date: 12.10.2020

Quality Assurance / Qualified Person

Version 01

Page 2 of 2

***Phase*: Histopathological Evaluation**	
***Phase Test Site*:**	***Contributing Scientist*:**
Charles River Laboratories, Inc.<br>8025 Lamon Ave., Suite 447<br>Skokie, IL 60077	Carol J. Detrisac, D.V.M., Ph.D.,<br>D.A.C.V.P.

Appendix H – Pathology Report

FINAL PATHOLOGY REPORT

Test Site Reference No. 0020272232

Testing Facility Project No. 285700300102

Sponsor Contract No. 75N91019D00013, Task Order No. 75N91020F00002

Two-Week Aerosol Toxicity Study of APN01 in Dogs

SPONSOR:
Chemopreventive Agent Development Research Group
Division of Cancer Prevention
National Cancer Institute
9609 Medical Center Drive
Rockville MD 20850
United States

TESTING FACILITY:
IIT Research Institute (IITRI)
10 West 35th Street
Chicago, IL 60616
United States

TEST SITE:
Charles River Laboratories, Inc.
8025 Lamon Avenue, Suite 447
Skokie, IL 60077
United States

Page 1 of 58

TABLE OF CONTENTS

LIST OF TABLES.....3

LIST OF APPENDICES.....3

QUALITY ASSURANCE STATEMENT.....4

COMPLIANCE STATEMENT AND REPORT APPROVAL.....5

1. RESPONSIBLE PERSONNEL.....6

2. INTRODUCTION.....6

3. MATERIALS AND METHODS.....6

3.1. Computerized Systems.....8

3.2. Disposition of Study Materials.....9

4. RESULTS AND DISCUSSIONS.....9

4.1. Mortality.....9

4.2. Gross Pathology.....9

4.2.1. Terminal Euthanasia Animals (Day 15).....9

4.3. Histopathology.....9

4.3.1. Terminal Euthanasia (Day 15).....9

5. CONCLUSIONS.....10

LIST OF TABLES

Table 1 Summary of Macroscopic Pathology (Day 15) 11
Table 2 Summary of Microscopic Pathology (Day 15) 13

LIST OF APPENDICES

Appendix 1 Deviations 26
Appendix 2 Individual Macroscopic and Microscopic Pathology...28

QUALITY ASSURANCE STATEMENT

Project Number: 285700300102

This phase has been audited by Quality Assurance in accordance with the applicable Good Laboratory Practice regulations. Reports were submitted in accordance with standard operating procedures as follows:

QA INSPECTION DATES

		Dates Findings Submitted to:			
**Date(s) of Audit**	**Phase(s) Audited**	**Contributing Scientist**	**Contributing Scientist Management**	**Study Director**	**Study Director Management***
08-Jan-2021	Phase Report - Pathology	08-Jan-2021	08-Jan-2021	22-Apr-2021	22-Apr-2021
02-Feb-2021	Data Review - Histology	02-Feb-2021	02-Feb-2021	03-Feb-2021	03-Feb-2021
22-Apr-2021	Final Phase Report - Pathology	22-Apr-2021	22-Apr-2021	22-Apr-2021	22-Apr-2021

Process-based inspections relevant to this study were conducted according to a predetermined schedule. The outcome of each inspection was reported to Management and, where relevant for processes seen as part of a study, the Study Director.

Facilities relevant to this study are included in Charles River's annual facility inspection program. The outcome of each inspection is reported to Management.
*Also date of report to the Lead QAU

DocuSigned by:

Enosha Simmons

Signer Name: Enosha Simmons
Signing Reason: I approve this document
Signing Time: 26-Apr-2021 | 08:41:50 EDT
4CCF1BF93FB74CD4B33DC7AD5C1B296E

Enosha Simmons
Quality Assurance Auditor

COMPLIANCE STATEMENT AND REPORT APPROVAL

The pathology phase of this study conducted in the USA was performed in accordance with the U.S. Department of Health and Human Services, Food and Drug Administration, United States Code of Federal Regulations, Title 21, Part 58: Good Laboratory Practice for Nonclinical Laboratory Studies.

This phase of the study was conducted in accordance with the procedures described herein. The report represents an accurate and complete record of the results obtained for this study phase.

There were no deviations from the above regulations that affected the overall integrity of this study phase or the interpretation of the phase results and conclusions.

DocuSigned by:
Carol J. Detrisac
Signer Name: Carol J. Detrisac
Signing Reason: I approve this document
Signing Time: 26-Apr-2021 | 08:44:50 EDT
7708E4156D194D53B549FE54B200310C

Carol J. Detrisac, DVM, PhD, DACVP
Contributing Scientist, Pathology

1. RESPONSIBLE PERSONNEL

Contributing Scientist, Pathology	Carol J. Detrisac, DVM, PhD, DACVP<br>Charles River Laboratories, Inc., Skokie, Illinois
Test Site Management	James T. Raymond, MS, DVM, DACVP<br>Charles River Laboratories, Inc., Frederick, Maryland

2. INTRODUCTION

This report presents the pathology findings in dogs assigned to Project No. 285700300102. The objective of this study was to provide data to support a comprehensive evaluation of the toxicity of twice daily aerosol administration of APN01 to dogs for fourteen consecutive days.

The study was sponsored by the Chemopreventive Agent Development Research Group of the Division of Cancer Prevention of the National Cancer Institute, Rockville, Maryland, where Robert Shoemaker, PhD, served as the Sponsor Representative. Jeffrey Richig, DVM, served as the Study Director.

3. MATERIALS AND METHODS

Experimental procedures applicable to pathology investigations are summarized in Text Table 1. Deviations to the pathology procedures (if any) performed by Charles River Laboratories, Inc. are listed in Appendix 1.

Text Table 1
Experimental Design

Group	Agent	Route of Administration	Number and Duration of Daily Exposures	Number of Exposure Days	Target APN01 Concentration	Target APN01 Exposure (mg/L)	Number of Dogs	
							Males	Females
1	Saline	Inhalation	2 x 60 minutes	14	0	0	3	3
2	Diluent	Inhalation	2 x 60 minutes	14	0	0	3	3
3	APN01	Inhalation	2 x 60 minutes	14	0.25 MFC	0.01875	3	3
4	APN01	Inhalation	2 x 60 minutes	14	0.5 MFC	0.0375	3	3
5	APN01	Inhalation	2 x 60 minutes	14	MFC	0.075	3	3

MFC = Maximum Feasible Concentration was 0.075 mg/L

All animals were submitted for necropsy on Day 15 (Main Study / Terminal Euthanasia). Necropsies were performed by Charles River Laboratories, Inc., Skokie, Illinois personnel. Tissue samples listed in Text Table 2 were collected and fixed in 10% neutral buffered formalin, unless otherwise noted.

Text Table 2
Tissue Collection and Examination

Provantis Tissue Term	Protocol Tissue Term	Collect	Weigh	Microscopic Evaluation	Comment
-	Animal identification	X	-	-	Ear tattoo
ARTERY, AORTA	Artery, aorta	X	-	X	
BONE, FEMUR	Bone, femur (with epiphyseal plate of head)	X	-	X	
BONE MARROW	Bone marrow, sternum	X	-	X	
BRAIN	Brain	X	X	X	
CERVIX	Cervix	X	-	X	
EPIDIDYMIS	Epididymis (paired)	X	X	X	Fixed in modified Davidson's solution
ESOPHAGUS	Esophagus	X	-	X	
EYE	Eye (paired)	X	-	X	Fixed in Davidson's solution
GALLBLADDER	Gallbladder	X	-	X	
GLAND, ADRENAL	Gland, adrenal (paired)	X	X	X	
GLAND, MAMMARY	Gland, mammary	X	-	X	
GLAND, PARATHYROID	Gland, parathyroid (paired)	X	-	X	
GLAND, PITUITARY	Gland, pituitary	X	-	X	
GLAND, PROSTATE	Gland, prostate	X	-	X	
GLAND, SALIVARY	Gland, salivary	X	-	X	
GLAND, THYROID	Gland, thyroid	X	-	X	
HEART	Heart	X	X	X	
KIDNEY	Kidneys (paired)	X	X	X	
LARGE INTESTINE, CECUM	Large intestine, cecum	X	-	X	
LARGE INTESTINE, COLON	Large intestine, colon	X	-	X	
LARGE INTESTINE, RECTUM	Large intestine, rectum	X	-	X	
LIVER	Liver	X	X	X	
LUNG	Lung	X	-	X	
LYMPH NODE, BRONCHIAL	Lymph node, bronchial	X	-	X	
LYMPH NODE, MANDIBULAR	Lymph node, mandibular	X	-	X	
LYMPH NODE, MESENTERIC	Lymph node, mesenteric	X	-	X	
MUSCLE, SKELETAL	Muscle, skeletal	X	-	X	
NERVE, OPTIC	Nerve, optic (paired)	X	-	X	Fixed in Davidson's solution
NERVE, SCIATIC	Nerve, sciatic	X	-	X	
OVARY	Ovary (paired)	X	X	X	
OVIDUCT	Oviduct (paired)	X	-	X	
PANCREAS	Pancreas	X	-	X	
SKIN	Skin (ventral abdomen)	X	-	X	

Provantis Tissue Term	Protocol Tissue Term	Collect	Weigh	Microscopic Evaluation	Comment
SMALL INTESTINE, DUODENUM	Small intestine, duodenum	X	-	X	
SMALL INTESTINE, ILEUM	Small intestine, ileum	X	-	X	
SMALL INTESTINE, JEJUNUM	Small intestine, jejunum	X	-	X	
SPINAL CORD	Spinal cord, cervical<br>Spinal cord, lumbar<br>Spinal cord, thoracic	X	-	X	
SPLEEN	Spleen	X	X	X	
STOMACH	Stomach	X	-	X	
TESTIS	Testis (paired)	X	X	X	Fixed in modified Davidson's solution
THYMUS	Thymus	X	X	X	
TONGUE	Tongue	X	-	X	
TONSIL	Tonsil (paired)	X	-	X	
TRACHEA	Trachea	X	-	X	
URETER	Ureter (paired)	X	-	X	
URINARY BLADDER	Urinary bladder	X	-	X	
UTERUS	Uterus	X	X	X	
VAGINA	Vagina	X	-	X	
-	Gross lesions	X	-	X	

Tissues required for microscopic evaluation were trimmed, processed routinely, embedded in paraffin, and stained with hematoxylin and eosin by Charles River Laboratories, Inc. Light microscopic evaluation was conducted by the Contributing Scientist, a board-certified veterinary pathologist, on protocol-specified tissues from all animals.

Tissues that were supposed to be microscopically evaluated per protocol but were not available on the slide (and therefore not evaluated) are listed in the Individual Animal Data of the pathology report as not present. These missing tissues did not affect the outcome or interpretation of the pathology portion of the study because the number of tissues examined from each treatment group was sufficient for interpretation.

3.1. Computerized Systems

Critical computerized systems used in the study by the Test Site are listed in Text Table 3.

Text Table 3
Computerized Systems

System Name	Description of Data Collected and/or Analyzed
Provantis®	Histopathology
Share Document Management System	Reporting
DocuSign™	Collection of Part 11 compliant signature

3.2. Disposition of Study Materials

All study-specific raw data, pathology materials, and documentation generated from this study phase were sent to the Testing Facility for archiving. Study materials will be retained for a period of 5 year following issue of the Final Report. Electronic data generated by the Test Site are to be archived as noted above, except that data collected using Provantis and reporting files stored on SDMS are to be archived at the Charles River Laboratories facility location in Wilmington, MA.

4. RESULTS AND DISCUSSIONS

4.1. Mortality

There were no unscheduled deaths during the course of this study.

4.2. Gross Pathology

4.2.1. Terminal Euthanasia Animals (Day 15)

(Table 1 and Appendix 2)

No test article-related gross findings were noted. The gross findings observed were considered incidental, of the nature commonly observed in this strain and age of dog, and/or were of similar incidence in control and treated animals and, therefore, were considered unrelated to administration of APN01.

4.3. Histopathology

4.3.1. Terminal Euthanasia (Day 15)

(Table 2 and Appendix 2)

Possible test article-related microscopic findings are summarized in Text Table 4.

Text Table 4
Summary of Microscopic Findings – Terminal Euthanasia (Day 15)

	Males					Females				
**Group**	**1**	**2**	**3**	**4**	**5**	**1**	**2**	**3**	**4**	**5**
**Target APN01 Concentration (MFC)**	**0**	**0**	**0.25**	**0.5**	**1**	**0**	**0**	**0.25**	**0.5**	**1**
**No. Animals Examined**	**3**	**3**	**3**	**3**	**3**	**3**	**3**	**3**	**3**	**3**
**Lymph node, bronchial (No. Examined)**	**3**	**3**	**3**	**3**	**3**	**3**	**3**	**3**	**3**	**3**
Hyperplasia, germinal center	(0)[a]	(1)	(0)	(0)	(2)	(0)	(2)	(2)	(1)	(2)
Minimal	0	1	0	0	2	0	1	2	1	2
Mild	0	0	0	0	0	0	1	0	0	0

[a] Numbers in parentheses represent the number of animals with the finding.

There was hyperplasia of the germinal centers of the bronchial lymph node, including in some dogs treated with only the diluent for the test article. The diluent and/or test article may have some local immunostimulatory effect; no other lymph nodes had these changes. This finding was considered equivocal.

There were many findings in the lungs, however the findings were not dose-related or consistent across groups. The foreign material was eosinophilic and approximately 25 - 100 µm diameter,

irregularly shaped and present in the foci of mixed cell infiltrates; they may represent particulates of digesta.

Other microscopic findings observed were considered incidental, of the nature commonly observed in this strain and age of dog, and/or were of similar incidence and severity in control and treated animals and, therefore, were considered unrelated to administration of APN01.

5. CONCLUSIONS

Aerosol administration of APN01 for 14 consecutive days at $\leq$ the maximum feasible concentration (0.075 mg/L) resulted in no gross findings or microscopic findings on Day 15.

Table 1
Summary of Macroscopic Pathology (Day 15)

Group	Agent	Target APN01 Concentration	Target APN01 Exposure (mg/L)
1	Saline	0	0
2	Diluent	0	0
3	APN01	0.25 MFC	0.01875
4	APN01	0.5 MFC	0.0375
5	APN01	MFC	0.075

Project No. 285700300102 - Summary of Macroscopic Pathology

Removal Reason(s): TERMINAL EUTHANASIA<br>Summary: Incidence	Male					Female				
	0 MFC Saline Group 1	0 MFC Group 2	0.25 MFC APN01 Group 3	0.5 MFC APN01 Group 4	1 MFC APN01 Group 5	0 MFC Saline Group 1	0 MFC Group 2	0.25 MFC APN01 Group 3	0.5 MFC APN01 Group 4	1 MFC APN01 Group 5
Number of Animals:	3	3	3	3	3	3	3	3	3	3
**KIDNEY**										
Submitted	3	3	3	3	3	3	3	3	3	3
No Visible Lesions	3	3	3	3	3	3	3	3	3	2
Cyst, clear	0	0	0	0	0	0	0	0	0	1
**LUNG**										
Submitted	3	3	3	3	3	3	3	3	3	3
No Visible Lesions	3	3	3	3	3	3	2	3	3	3
Focus; tan, cranial lobe	0	0	0	0	0	0	1	0	0	0
**LYMPH NODE, BRONCHIAL**										
Submitted	3	3	3	3	3	3	3	3	3	3
No Visible Lesions	3	3	3	3	3	3	3	2	3	3
Enlargement	0	0	0	0	0	0	0	1	0	0
**OVARY**										
Submitted	.	.	.	.	.	3	3	3	3	3
No Visible Lesions	.	.	.	.	.	3	2	3	3	3
Enlargement	.	.	.	.	.	0	1	0	0	0
**TONSIL**										
Submitted	3	3	3	3	3	3	3	3	3	3
No Visible Lesions	3	2	2	3	3	3	3	3	3	2
Discoloration; red	0	1	1	0	0	0	0	0	0	1

Table 2
Summary of Microscopic Pathology (Day 15)

Group	Agent	Target APN01 Concentration	Target APN01 Exposure (mg/L)
1	Saline	0	0
2	Diluent	0	0
3	APN01	0.25 MFC	0.01875
4	APN01	0.5 MFC	0.0375
5	APN01	MFC	0.075

Project No. 285700300102 - Summary of Microscopic Pathology

Removal Reason(s): TERMINAL EUTHANASIA<br>Summary: Incidence	Male<br>0 MFC Saline Group 1	0 MFC Group 2	0.25 MFC APN01 Group 3	0.5 MFC APN01 Group 4	1 MFC APN01 Group 5	Female<br>0 MFC Saline Group 1	0 MFC Group 2	0.25 MFC APN01 Group 3	0.5 MFC APN01 Group 4	1 MFC APN01 Group 5
Number of Animals:	3	3	3	3	3	3	3	3	3	3
**ARTERY, AORTA**										
Examined	3	3	3	3	3	3	3	3	3	3
No Visible Lesions	3	3	3	3	3	3	3	2	3	3
Degeneration	0	0	0	0	0	0	0	1	0	0
.... mild	0	0	0	0	0	0	0	1	0	0
Mineralization	0	0	0	0	0	0	0	1	0	0
.... minimal	0	0	0	0	0	0	0	1	0	0
**BONE MARROW**										
Examined	3	3	3	3	3	3	3	3	3	3
No Visible Lesions	3	3	3	3	3	3	3	2	3	3
Myeloid to erythroid ratio, increased	0	0	0	0	0	0	0	1	0	0
.... mild	0	0	0	0	0	0	0	1	0	0
**BONE, FEMUR**										
Examined	3	3	3	3	3	3	3	3	3	3
No Visible Lesions	2	3	3	3	2	2	3	3	3	3
Degeneration; physis	1	0	0	0	1	1	0	0	0	0
.... minimal	1	0	0	0	1	1	0	0	0	0
**BRAIN**										
Examined	3	3	3	3	3	3	3	3	3	3
No Visible Lesions	3	3	3	3	3	3	3	3	3	3

Project No. 285700300102 - Summary of Microscopic Pathology

Removal Reason(s): TERMINAL EUTHANASIA<br>Summary: Incidence	Male					Female				
	0 MFC Saline Group 1	0 MFC Group 2	0.25 MFC APN01 Group 3	0.5 MFC APN01 Group 4	1 MFC APN01 Group 5	0 MFC Saline Group 1	0 MFC Group 2	0.25 MFC APN01 Group 3	0.5 MFC APN01 Group 4	1 MFC APN01 Group 5
Number of Animals:	3	3	3	3	3	3	3	3	3	3
**CERVIX**										
Examined	.	.	.	.	.	3	3	3	3	3
No Visible Lesions	.	.	.	.	.	3	3	2	3	3
Hyperplasia	.	.	.	.	.	0	0	1	0	0
.... minimal	.	.	.	.	.	0	0	1	0	0
**EPIDIDYMIS**										
Examined	3	3	3	3	3	.	.	.	.	.
No Visible Lesions	3	3	2	1	1	.	.	.	.	.
Cellular debris	0	0	1	2	2	.	.	.	.	.
.... minimal	0	0	1	2	1	.	.	.	.	.
.... mild	0	0	0	0	1	.	.	.	.	.
**ESOPHAGUS**										
Examined	3	3	3	3	3	3	3	3	3	3
No Visible Lesions	3	3	3	3	3	2	3	3	3	3
Infiltration, mononuclear cell	0	0	0	0	0	1	0	0	0	0
.... minimal	0	0	0	0	0	1	0	0	0	0
**EYE**										
Examined	3	3	3	3	3	3	3	3	3	3
No Visible Lesions	3	3	3	3	3	3	3	3	3	3
**GALLBLADDER**										
Examined	3	3	3	3	3	3	3	3	3	3

Project No. 285700300102 - Summary of Microscopic Pathology

Removal Reason(s): TERMINAL EUTHANASIA<br>Summary: Incidence	Male					Female				
	0 MFC Saline Group 1	0 MFC Group 2	0.25 MFC APN01 Group 3	0.5 MFC APN01 Group 4	1 MFC APN01 Group 5	0 MFC Saline Group 1	0 MFC Group 2	0.25 MFC APN01 Group 3	0.5 MFC APN01 Group 4	1 MFC APN01 Group 5
Number of Animals:	3	3	3	3	3	3	3	3	3	3
**GALLBLADDER (Continued...)**										
No Visible Lesions	3	3	3	3	3	3	3	3	3	3
**GLAND, ADRENAL**										
Examined	3	3	3	3	3	3	3	3	3	3
No Visible Lesions	3	3	3	3	3	3	3	3	3	3
**GLAND, MAMMARY**										
Examined	3	2	2	3	2	3	3	3	3	3
No Visible Lesions	3	2	2	3	2	3	3	3	3	3
Not Examined: Not Present In Section.	0	1	1	0	1	.	.	.	.	.
**GLAND, PARATHYROID**										
Examined	3	3	3	3	2	2	3	3	3	3
No Visible Lesions	3	3	3	3	2	2	3	3	3	3
Not Examined: Not Present In Section.	0	0	0	0	1	1	0	0	0	0
**GLAND, PITUITARY**										
Examined	3	3	3	3	3	3	3	3	3	3
No Visible Lesions	2	3	2	2	3	3	3	3	3	3
Cyst	1	0	1	1	0	0	0	0	0	0
**GLAND, PROSTATE**										
Examined	3	3	3	3	3	.	.	.	.	.
No Visible Lesions	3	3	3	3	3	.	.	.	.	.

Project No. 285700300102 - Summary of Microscopic Pathology

Removal Reason(s): TERMINAL EUTHANASIA<br>Summary: Incidence	Male					Female				
	0 MFC Saline Group 1	0 MFC Group 2	0.25 MFC APN01 Group 3	0.5 MFC APN01 Group 4	1 MFC APN01 Group 5	0 MFC Saline Group 1	0 MFC Group 2	0.25 MFC APN01 Group 3	0.5 MFC APN01 Group 4	1 MFC APN01 Group 5
Number of Animals:	3	3	3	3	3	3	3	3	3	3
**GLAND, SALIVARY**										
Examined	3	3	3	3	3	3	3	3	3	3
No Visible Lesions	3	3	3	3	3	3	3	3	3	3
**GLAND, THYROID**										
Examined	3	3	3	3	3	3	3	3	3	3
No Visible Lesions	3	3	3	3	3	3	3	3	3	3
**HEART**										
Examined	3	3	3	3	3	3	3	3	3	3
No Visible Lesions	3	3	3	3	3	3	3	3	3	3
**KIDNEY**										
Examined	3	3	3	3	3	3	3	3	3	3
No Visible Lesions	0	0	0	0	1	0	0	0	0	0
Mineralization	3	3	3	3	2	3	2	3	3	3
.... minimal	3	3	3	3	2	3	2	3	3	3
Infiltration, mixed cell; cortical	0	0	0	0	1	0	0	0	0	0
.... mild	0	0	0	0	1	0	0	0	0	0
Cyst; tubular	0	0	0	0	0	0	0	0	0	1
Vacuolation; tubular	0	0	0	0	0	0	1	0	0	0
.... minimal	0	0	0	0	0	0	1	0	0	0
**LARGE INTESTINE, CECUM**										
Examined	3	3	3	3	3	3	3	3	3	3

Project No. 285700300102 - Summary of Microscopic Pathology

Removal Reason(s): TERMINAL EUTHANASIA<br>Summary: Incidence	Male<br>0 MFC Saline Group 1	Male<br>0 MFC Group 2	Male<br>0.25 MFC APN01 Group 3	Male<br>0.5 MFC APN01 Group 4	Male<br>1 MFC APN01 Group 5	Female<br>0 MFC Saline Group 1	Female<br>0 MFC Group 2	Female<br>0.25 MFC APN01 Group 3	Female<br>0.5 MFC APN01 Group 4	Female<br>1 MFC APN01 Group 5
Number of Animals:	3	3	3	3	3	3	3	3	3	3
**LARGE INTESTINE, CECUM (Continued...)**										
No Visible Lesions	3	3	3	3	3	3	3	3	3	3
**LARGE INTESTINE, COLON**										
Examined	3	3	3	3	3	3	3	3	3	3
No Visible Lesions	3	3	3	3	3	3	3	3	3	3
**LARGE INTESTINE, RECTUM**										
Examined	3	3	3	3	3	3	3	3	3	3
No Visible Lesions	3	3	3	3	3	3	3	3	3	3
**LIVER**										
Examined	3	3	3	3	3	3	3	3	3	3
No Visible Lesions	3	3	3	3	3	3	3	3	3	3
**LUNG**										
Examined	3	3	3	3	3	3	3	3	3	3
No Visible Lesions	2	2	1	3	2	1	1	2	1	0
Fibrosis; pleural	0	0	0	0	0	0	0	0	1	1
.... minimal	0	0	0	0	0	0	0	0	0	1
.... mild	0	0	0	0	0	0	0	0	1	0
Fibrosis; septal	0	0	0	0	0	0	1	0	0	0
.... minimal	0	0	0	0	0	0	1	0	0	0
Foreign material; alveolar	0	1	1	0	0	1	1	0	1	0
.... minimal	0	1	1	0	0	1	1	0	1	0

Project No. 285700300102 - Summary of Microscopic Pathology

Removal Reason(s): TERMINAL EUTHANASIA<br>Summary: Incidence	Male					Female				
	0 MFC Saline Group 1	0 MFC Group 2	0.25 MFC APN01 Group 3	0.5 MFC APN01 Group 4	1 MFC APN01 Group 5	0 MFC Saline Group 1	0 MFC Group 2	0.25 MFC APN01 Group 3	0.5 MFC APN01 Group 4	1 MFC APN01 Group 5
Number of Animals:	3	3	3	3	3	3	3	3	3	3
**LUNG (Continued...)**										
Mineralization; submucosal, bronchus	0	0	0	0	0	0	0	0	0	1
.... minimal	0	0	0	0	0	0	0	0	0	1
Infiltration, mononuclear cell; alveolar	0	0	0	0	0	0	1	0	0	0
.... minimal	0	0	0	0	0	0	1	0	0	0
Infiltration, mononuclear cell; perivascular	1	0	0	0	1	0	0	0	0	0
.... minimal	1	0	0	0	1	0	0	0	0	0
Infiltration, mixed cell; alveolar	1	1	1	0	0	2	1	1	1	2
.... minimal	1	1	1	0	0	2	0	0	1	2
.... mild	0	0	0	0	0	0	1	0	0	0
.... moderate	0	0	0	0	0	0	0	1	0	0
Hypertrophy; pneumocyte, type 2	0	0	1	0	0	0	0	0	0	0
.... mild	0	0	1	0	0	0	0	0	0	0
**LYMPH NODE, BRONCHIAL**										
Examined	3	3	3	3	3	3	3	3	3	3
No Visible Lesions	2	2	3	3	1	3	1	1	2	1
Hemorrhage; acute	1	0	0	0	0	0	1	0	0	0
.... minimal	1	0	0	0	0	0	1	0	0	0
Hyperplasia; germinal center	0	1	0	0	2	0	2	2	1	2
.... minimal	0	1	0	0	2	0	1	2	1	2
.... mild	0	0	0	0	0	0	1	0	0	0

Project No. 285700300102 - Summary of Microscopic Pathology

Removal Reason(s): TERMINAL EUTHANASIA<br>Summary: Incidence	Male<br>0 MFC Saline Group 1	Male<br>0 MFC Group 2	Male<br>0.25 MFC APN01 Group 3	Male<br>0.5 MFC APN01 Group 4	Male<br>1 MFC APN01 Group 5	Female<br>0 MFC Saline Group 1	Female<br>0 MFC Group 2	Female<br>0.25 MFC APN01 Group 3	Female<br>0.5 MFC APN01 Group 4	Female<br>1 MFC APN01 Group 5
Number of Animals:	3	3	3	3	3	3	3	3	3	3
**LYMPH NODE, BRONCHIAL (Continued...)**										
Infiltration, mixed cell	0	0	0	0	0	0	0	1	0	0
.... minimal	0	0	0	0	0	0	0	1	0	0
**LYMPH NODE, MANDIBULAR**										
Examined	3	3	3	3	3	3	3	3	3	3
No Visible Lesions	3	3	3	3	3	3	3	3	3	3
**LYMPH NODE, MESENTERIC**										
Examined	3	3	3	3	3	3	3	3	3	3
No Visible Lesions	3	3	2	3	1	3	2	3	3	2
Erythrophagocytosis	0	0	1	0	2	0	1	0	0	1
.... minimal	0	0	0	0	1	0	1	0	0	0
.... mild	0	0	1	0	1	0	0	0	0	1
**MUSCLE, SKELETAL**										
Examined	3	3	3	3	3	3	3	3	3	3
No Visible Lesions	3	3	3	3	3	3	3	3	3	3
**NERVE, OPTIC**										
Examined	3	3	3	3	3	3	3	3	3	3
No Visible Lesions	3	3	3	3	3	3	3	3	3	3
**NERVE, SCIATIC**										
Examined	3	3	3	3	3	3	3	3	3	3
No Visible Lesions	3	3	3	3	3	3	3	3	3	3

Project No. 285700300102 - Summary of Microscopic Pathology

Removal Reason(s): TERMINAL EUTHANASIA<br>Summary: Incidence	Male					Female				
	0 MFC Saline Group 1	0 MFC Group 2	0.25 MFC APN01 Group 3	0.5 MFC APN01 Group 4	1 MFC APN01 Group 5	0 MFC Saline Group 1	0 MFC Group 2	0.25 MFC APN01 Group 3	0.5 MFC APN01 Group 4	1 MFC APN01 Group 5
Number of Animals:	3	3	3	3	3	3	3	3	3	3
**OVARY**										
Examined	.	.	.	.	.	3	3	3	3	3
No Visible Lesions	.	.	.	.	.	0	1	0	0	0
Immaturity	.	.	.	.	.	3	2	3	3	3
**OVIDUCT**										
Examined	.	.	.	.	.	3	3	2	3	3
No Visible Lesions	.	.	.	.	.	3	3	2	3	3
Not Examined: Not Present In Section.	.	.	.	.	.	0	0	1	0	0
**PANCREAS**										
Examined	3	3	3	3	3	3	3	3	3	3
No Visible Lesions	3	3	3	3	3	3	3	3	3	3
**SKIN**										
Examined	3	3	3	3	3	3	3	3	3	3
No Visible Lesions	3	3	3	3	3	3	3	3	2	3
Granuloma	0	0	0	0	0	0	0	0	1	0
.... minimal	0	0	0	0	0	0	0	0	1	0
**SMALL INTESTINE, DUODENUM**										
Examined	3	3	3	3	3	3	3	3	3	3
No Visible Lesions	3	2	3	3	3	3	2	3	3	2
Ectasia; crypt	0	1	0	0	0	0	1	0	0	1
.... minimal	0	0	0	0	0	0	1	0	0	1

Project No. 285700300102 - Summary of Microscopic Pathology

Removal Reason(s): TERMINAL EUTHANASIA<br>Summary: Incidence	Male<br>0 MFC Saline Group 1	0 MFC Group 2	0.25 MFC APN01 Group 3	0.5 MFC APN01 Group 4	1 MFC APN01 Group 5	Female<br>0 MFC Saline Group 1	0 MFC Group 2	0.25 MFC APN01 Group 3	0.5 MFC APN01 Group 4	1 MFC APN01 Group 5
Number of Animals:	3	3	3	3	3	3	3	3	3	3
**SMALL INTESTINE, DUODENUM (Continued...)**										
.... mild	0	1	0	0	0	0	0	0	0	0
**SMALL INTESTINE, ILEUM**										
Examined	3	3	3	3	3	3	3	3	3	3
No Visible Lesions	3	3	3	2	3	3	2	3	2	3
Atrophy; mucosal	0	0	0	1	0	0	1	0	0	0
.... mild	0	0	0	1	0	0	1	0	0	0
Lymphangiectasia; villous	0	0	0	0	0	0	0	0	1	0
.... mild	0	0	0	0	0	0	0	0	1	0
**SMALL INTESTINE, JEJUNUM**										
Examined	3	3	3	3	3	3	3	3	3	3
No Visible Lesions	3	3	3	3	3	3	3	3	3	2
Atrophy; mucosal	0	0	0	0	0	0	0	0	0	1
.... mild	0	0	0	0	0	0	0	0	0	1
**SPINAL CORD**										
Examined	3	3	3	3	3	3	3	3	3	3
No Visible Lesions	3	3	3	3	3	3	3	3	3	3
**SPLEEN**										
Examined	3	3	3	3	3	3	3	3	3	3
No Visible Lesions	3	2	3	3	3	3	3	3	3	3
Siderotic plaque	0	1	0	0	0	0	0	0	0	0

Project No. 285700300102 - Summary of Microscopic Pathology

Removal Reason(s): TERMINAL EUTHANASIA<br>Summary: Incidence	Male					Female				
	0 MFC Saline Group 1	0 MFC Group 2	0.25 MFC APN01 Group 3	0.5 MFC APN01 Group 4	1 MFC APN01 Group 5	0 MFC Saline Group 1	0 MFC Group 2	0.25 MFC APN01 Group 3	0.5 MFC APN01 Group 4	1 MFC APN01 Group 5
Number of Animals:	3	3	3	3	3	3	3	3	3	3
**SPLEEN (Continued...)**										
.... minimal	0	1	0	0	0	0	0	0	0	0
**STOMACH**										
Examined	3	3	3	3	3	3	3	3	3	3
No Visible Lesions	3	3	3	3	2	2	3	3	3	3
Mineralization; mucosal	0	0	0	0	0	1	0	0	0	0
.... mild	0	0	0	0	0	1	0	0	0	0
Mineralization; mucosal, submucosal	0	0	0	0	1	0	0	0	0	0
.... minimal	0	0	0	0	1	0	0	0	0	0
**TESTIS**										
Examined	3	3	3	3	3	.	.	.	.	.
No Visible Lesions	3	3	3	3	2	.	.	.	.	.
Degeneration; seminiferous tubule	0	0	0	0	1	.	.	.	.	.
.... mild	0	0	0	0	1	.	.	.	.	.
**THYMUS**										
Examined	3	3	3	3	3	3	3	3	3	3
No Visible Lesions	3	2	1	2	3	3	2	3	3	2
Cellularity, decreased; cortical	0	1	2	1	0	0	1	0	0	1
.... mild	0	1	1	0	0	0	1	0	0	0
.... moderate	0	0	1	1	0	0	0	0	0	1

Project No. 285700300102 - Summary of Microscopic Pathology

Removal Reason(s): TERMINAL EUTHANASIA<br>Summary: Incidence	Male					Female				
	0 MFC Saline Group 1	0 MFC Group 2	0.25 MFC APN01 Group 3	0.5 MFC APN01 Group 4	1 MFC APN01 Group 5	0 MFC Saline Group 1	0 MFC Group 2	0.25 MFC APN01 Group 3	0.5 MFC APN01 Group 4	1 MFC APN01 Group 5
Number of Animals:	3	3	3	3	3	3	3	3	3	3
**TONGUE**										
Examined	3	3	3	3	3	3	3	3	3	3
No Visible Lesions	3	3	3	3	3	3	3	3	3	3
**TONSIL**										
Examined	3	3	3	3	3	3	3	3	3	3
No Visible Lesions	2	1	3	3	2	2	2	3	3	1
Hemorrhage	0	1	0	0	0	0	0	0	0	0
.... minimal	0	1	0	0	0	0	0	0	0	0
Hemorrhage; acute	1	1	0	0	1	1	1	0	0	2
.... minimal	1	1	0	0	1	1	1	0	0	2
**TRACHEA**										
Examined	3	3	3	3	3	3	3	3	3	3
No Visible Lesions	3	3	3	3	2	3	3	3	3	3
Metaplasia, squamous; carina	0	0	0	0	1	0	0	0	0	0
.... mild	0	0	0	0	1	0	0	0	0	0
**URETER**										
Examined	3	3	3	3	3	3	3	3	3	3
No Visible Lesions	3	3	3	3	3	3	3	3	3	3
**URINARY BLADDER**										
Examined	3	3	3	3	3	3	3	3	3	3
No Visible Lesions	3	3	3	3	3	3	3	3	3	3

Project No. 285700300102 - Summary of Microscopic Pathology

Removal Reason(s): TERMINAL EUTHANASIA	Male					Female				
Summary: Incidence	0 MFC Saline Group 1	0 MFC Group 2	0.25 MFC APN01 Group 3	0.5 MFC APN01 Group 4	1 MFC APN01 Group 5	0 MFC Saline Group 1	0 MFC Group 2	0.25 MFC APN01 Group 3	0.5 MFC APN01 Group 4	1 MFC APN01 Group 5
Number of Animals:	3	3	3	3	3	3	3	3	3	3
**UTERUS**										
Examined	.	.	.	.	.	3	3	3	3	3
No Visible Lesions	.	.	.	.	.	3	3	3	3	3
**VAGINA**										
Examined	.	.	.	.	.	3	3	3	3	3
No Visible Lesions	.	.	.	.	.	3	3	3	3	3

Appendix 1
Deviations

DEVIATIONS

No deviations occurred during this phase of the study.

Appendix 2
Individual Macroscopic and Microscopic Pathology

Group	Agent	Target APN01 Concentration	Target APN01 Exposure (mg/L)
1	Saline	0	0
2	Diluent	0	0
3	APN01	0.25 MFC	0.01875
4	APN01	0.5 MFC	0.0375
5	APN01	MFC	0.075

Project No. 285700300102 - Individual Macroscopic and Microscopic Pathology

Animal:	1EZF	Group:	1	Sex:	Male
Species:	Dog	Strain:	Beagle		
		Dose:	0 MFC Saline		
		Removal Reason:	Terminal Euthanasia		
		Study Day (Week) of Death:	15 (3)		

Gross Pathology Animal Details:

No animal details found

Gross Pathology Observations [Correlation]:

No observations found

Any remaining protocol required tissues, which have been examined, have no visible lesions

Gross Pathology - The following Tissues were Not Examined:

None

Histopathology Animal Details:

No animal details found

Histopathology Observations [Correlation]:

GLAND, PITUITARY : Cyst
KIDNEY : Mineralization; minimal
LUNG : Infiltration, mononuclear cell; perivascular, minimal
LUNG : Infiltration, mixed cell; alveolar, focal, minimal

Histopathology - The following Tissues were Within Normal Limits:

ARTERY, AORTA; BONE MARROW; BONE, FEMUR; BRAIN; EPIDIDYMIS; ESOPHAGUS; EYE; GALLBLADDER; GLAND, ADRENAL; GLAND, MAMMARY; GLAND, PARATHYROID; GLAND, PROSTATE; GLAND, SALIVARY; GLAND, THYROID; HEART; LARGE INTESTINE, CECUM; LARGE INTESTINE, COLON; LARGE INTESTINE, RECTUM; LIVER; LYMPH NODE, BRONCHIAL; LYMPH NODE, MANDIBULAR; LYMPH NODE, MESENTERIC; MUSCLE, SKELETAL; NERVE, OPTIC; NERVE, SCIATIC; PANCREAS; SKIN; SMALL INTESTINE, DUODENUM; SMALL INTESTINE, ILEUM; SMALL INTESTINE, JEJUNUM; SPINAL CORD; SPLEEN; STOMACH; TESTIS; THYMUS; TONGUE; TONSIL; TRACHEA; URETER; URINARY BLADDER

Histopathology - The following Tissues were Not Examined:

None

Project No. 285700300102 - Individual Macroscopic and Microscopic Pathology

Animal:	1FUH	Group:	1	Sex:	Male
Species:	Dog	Strain:	Beagle		
		Dose:	0 MFC Saline		
		Removal Reason:	Terminal Euthanasia		
		Study Day (Week) of Death:	15 (3)		

Gross Pathology Animal Details:

No animal details found

Gross Pathology Observations [Correlation]:

No observations found

Any remaining protocol required tissues, which have been examined, have no visible lesions

Gross Pathology - The following Tissues were Not Examined:

None

Histopathology Animal Details:

No animal details found

Histopathology Observations [Correlation]:

KIDNEY : Mineralization; minimal
LYMPH NODE, BRONCHIAL : Hemorrhage; acute, minimal

Histopathology - The following Tissues were Within Normal Limits:

ARTERY, AORTA; BONE MARROW; BONE, FEMUR; BRAIN; EPIDIDYMIS; ESOPHAGUS; EYE; GALLBLADDER; GLAND, ADRENAL; GLAND, MAMMARY; GLAND, PARATHYROID; GLAND, PITUITARY; GLAND, PROSTATE; GLAND, SALIVARY; GLAND, THYROID; HEART; LARGE INTESTINE, CECUM; LARGE INTESTINE, COLON; LARGE INTESTINE, RECTUM; LIVER; LUNG; LYMPH NODE, MANDIBULAR; LYMPH NODE, MESENTERIC; MUSCLE, SKELETAL; NERVE, OPTIC; NERVE, SCIATIC; PANCREAS; SKIN; SMALL INTESTINE, DUODENUM; SMALL INTESTINE, ILEUM; SMALL INTESTINE, JEJUNUM; SPINAL CORD; SPLEEN; STOMACH; TESTIS; THYMUS; TONGUE; TONSIL; TRACHEA; URETER; URINARY BLADDER

Histopathology - The following Tissues were Not Examined:

None

Project No. 285700300102 - Individual Macroscopic and Microscopic Pathology

Animal:	1UYF	Group:	1	Sex:	Male
Species:	Dog	Strain:	Beagle		
		Dose:	0 MFC Saline		
		Removal Reason:	Terminal Euthanasia		
		Study Day (Week) of Death:	15 (3)		

Gross Pathology Animal Details:

No animal details found

Gross Pathology Observations [Correlation]:

No observations found

Any remaining protocol required tissues, which have been examined, have no visible lesions

Gross Pathology - The following Tissues were Not Examined:

None

Histopathology Animal Details:

No animal details found

Histopathology Observations [Correlation]:

BONE, FEMUR : Degeneration; minimal, physis
GLAND, PARATHYROID : (Note) One Of A Pair Available For Evaluation.
KIDNEY : Mineralization; minimal
TONSIL : Hemorrhage; acute, minimal

Histopathology - The following Tissues were Within Normal Limits:

ARTERY, AORTA; BONE MARROW; BRAIN; EPIDIDYMIS; ESOPHAGUS; EYE; GALLBLADDER; GLAND, ADRENAL; GLAND, MAMMARY; GLAND, PARATHYROID; GLAND, PITUITARY; GLAND, PROSTATE; GLAND, SALIVARY; GLAND, THYROID; HEART; LARGE INTESTINE, CECUM; LARGE INTESTINE, COLON; LARGE INTESTINE, RECTUM; LIVER; LUNG; LYMPH NODE, BRONCHIAL; LYMPH NODE, MANDIBULAR; LYMPH NODE, MESENTERIC; MUSCLE, SKELETAL; NERVE, OPTIC; NERVE, SCIATIC; PANCREAS; SKIN; SMALL INTESTINE, DUODENUM; SMALL INTESTINE, ILEUM; SMALL INTESTINE, JEJUNUM; SPINAL CORD; SPLEEN; STOMACH; TESTIS; THYMUS; TONGUE; TRACHEA; URETER; URINARY BLADDER

Histopathology - The following Tissues were Not Examined:

None

Project No. 285700300102 - Individual Macroscopic and Microscopic Pathology

Animal:	1CBE	Group:	1	Sex:	Female
Species:	Dog	Strain:	Beagle		
		Dose:	0 MFC Saline		
		Removal Reason:	Terminal Euthanasia		
		Study Day (Week) of Death:	15 (3)		

Gross Pathology Animal Details:

No animal details found

Gross Pathology Observations [Correlation]:

No observations found

Any remaining protocol required tissues, which have been examined, have no visible lesions

Gross Pathology - The following Tissues were Not Examined:

None

Histopathology Animal Details:

No animal details found

Histopathology Observations [Correlation]:

KIDNEY : Mineralization; minimal
LUNG : Foreign material; alveolar, focal, minimal
LUNG : Infiltration, mixed cell; alveolar, focal, minimal
OVARY : Immaturity

Histopathology - The following Tissues were Within Normal Limits:

ARTERY, AORTA; BONE MARROW; BONE, FEMUR; BRAIN; CERVIX; ESOPHAGUS; EYE; GALLBLADDER; GLAND, ADRENAL; GLAND, MAMMARY; GLAND, PARATHYROID; GLAND, PITUITARY; GLAND, SALIVARY; GLAND, THYROID; HEART; LARGE INTESTINE, CECUM; LARGE INTESTINE, COLON; LARGE INTESTINE, RECTUM; LIVER; LYMPH NODE, BRONCHIAL; LYMPH NODE, MANDIBULAR; LYMPH NODE, MESENTERIC; MUSCLE, SKELETAL; NERVE, OPTIC; NERVE, SCIATIC; OVIDUCT; PANCREAS; SKIN; SMALL INTESTINE, DUODENUM; SMALL INTESTINE, ILEUM; SMALL INTESTINE, JEJUNUM; SPINAL CORD; SPLEEN; STOMACH; THYMUS; TONGUE; TONSIL; TRACHEA; URETER; URINARY BLADDER; UTERUS; VAGINA

Histopathology - The following Tissues were Not Examined:

None

Project No. 285700300102 - Individual Macroscopic and Microscopic Pathology

Animal:	1JKG	Group:	1	Sex:	Female
Species:	Dog	Strain:	Beagle		
		Dose:	0 MFC Saline		
		Removal Reason:	Terminal Euthanasia		
		Study Day (Week) of Death:	15 (3)		

Gross Pathology Animal Details:

No animal details found

Gross Pathology Observations [Correlation]:

No observations found

Any remaining protocol required tissues, which have been examined, have no visible lesions

Gross Pathology - The following Tissues were Not Examined:

None

Histopathology Animal Details:

No animal details found

Histopathology Observations [Correlation]:

ESOPHAGUS : Infiltration, mononuclear cell; minimal
KIDNEY : Mineralization; minimal
LUNG : Infiltration, mixed cell; alveolar, focal, minimal
OVARY : Immaturity

Histopathology - The following Tissues were Within Normal Limits:

ARTERY, AORTA; BONE MARROW; BONE, FEMUR; BRAIN; CERVIX; EYE; GALLBLADDER; GLAND, ADRENAL; GLAND, MAMMARY; GLAND, PARATHYROID; GLAND, PITUITARY; GLAND, SALIVARY; GLAND, THYROID; HEART; LARGE INTESTINE, CECUM; LARGE INTESTINE, COLON; LARGE INTESTINE, RECTUM; LIVER; LYMPH NODE, BRONCHIAL; LYMPH NODE, MANDIBULAR; LYMPH NODE, MESENTERIC; MUSCLE, SKELETAL; NERVE, OPTIC; NERVE, SCIATIC; OVIDUCT; PANCREAS; SKIN; SMALL INTESTINE, DUODENUM; SMALL INTESTINE, ILEUM; SMALL INTESTINE, JEJUNUM; SPINAL CORD; SPLEEN; STOMACH; THYMUS; TONGUE; TONSIL; TRACHEA; URETER; URINARY BLADDER; UTERUS; VAGINA

Histopathology - The following Tissues were Not Examined:

None

Project No. 285700300102 - Individual Macroscopic and Microscopic Pathology

Animal:	1JSG	Group:	1	Sex:	Female
Species:	Dog	Strain:	Beagle		
		Dose:	0 MFC Saline		
		Removal Reason:	Terminal Euthanasia		
		Study Day (Week) of Death:	15 (3)		

Gross Pathology Animal Details:

No animal details found

Gross Pathology Observations [Correlation]:

No observations found

Any remaining protocol required tissues, which have been examined, have no visible lesions

Gross Pathology - The following Tissues were Not Examined:

None

Histopathology Animal Details:

No animal details found

Histopathology Observations [Correlation]:

BONE, FEMUR : Degeneration; minimal, physis
KIDNEY : Mineralization; minimal
OVARY : Immaturity
STOMACH : Mineralization; mucosal, mild
TONSIL : Hemorrhage; acute, minimal

Histopathology - The following Tissues were Within Normal Limits:

ARTERY, AORTA; BONE MARROW; BRAIN; CERVIX; ESOPHAGUS; EYE; GALLBLADDER; GLAND, ADRENAL; GLAND, MAMMARY; GLAND, PITUITARY; GLAND, SALIVARY; GLAND, THYROID; HEART; LARGE INTESTINE, CECUM; LARGE INTESTINE, COLON; LARGE INTESTINE, RECTUM; LIVER; LUNG; LYMPH NODE, BRONCHIAL; LYMPH NODE, MANDIBULAR; LYMPH NODE, MESENTERIC; MUSCLE, SKELETAL; NERVE, OPTIC; NERVE, SCIATIC; OVIDUCT; PANCREAS; SKIN; SMALL INTESTINE, DUODENUM; SMALL INTESTINE, ILEUM; SMALL INTESTINE, JEJUNUM; SPINAL CORD; SPLEEN; THYMUS; TONGUE; TRACHEA; URETER; URINARY BLADDER; UTERUS; VAGINA

Histopathology - The following Tissues were Not Examined:

GLAND, PARATHYROID - Not Present In Section.

Project No. 285700300102 - Individual Macroscopic and Microscopic Pathology

Animal:	2BSF	Group:	2	Sex:	Male
Species:	Dog	Strain:	Beagle		
		Dose:	0 MFC		
		Removal Reason:	Terminal Euthanasia		
		Study Day (Week) of Death:	15 (3)		

Gross Pathology Animal Details:

No animal details found

Gross Pathology Observations [Correlation]:

No observations found

Any remaining protocol required tissues, which have been examined, have no visible lesions

Gross Pathology - The following Tissues were Not Examined:

None

Histopathology Animal Details:

No animal details found

Histopathology Observations [Correlation]:

KIDNEY : Mineralization; minimal
LUNG : Foreign material; alveolar, focal, minimal
LUNG : Infiltration, mixed cell; alveolar, focal, minimal
LYMPH NODE, BRONCHIAL : Hyperplasia; minimal, germinal center
TRACHEA : (Comment) Carina unavailable for evaluation.

Histopathology - The following Tissues were Within Normal Limits:

ARTERY, AORTA; BONE MARROW; BONE, FEMUR; BRAIN; EPIDIDYMIS; ESOPHAGUS; EYE; GALLBLADDER; GLAND, ADRENAL; GLAND, MAMMARY; GLAND, PARATHYROID; GLAND, PITUITARY; GLAND, PROSTATE; GLAND, SALIVARY; GLAND, THYROID; HEART; LARGE INTESTINE, CECUM; LARGE INTESTINE, COLON; LARGE INTESTINE, RECTUM; LIVER; LYMPH NODE, MANDIBULAR; LYMPH NODE, MESENTERIC; MUSCLE, SKELETAL; NERVE, OPTIC; NERVE, SCIATIC; PANCREAS; SKIN; SMALL INTESTINE, DUODENUM; SMALL INTESTINE, ILEUM; SMALL INTESTINE, JEJUNUM; SPINAL CORD; SPLEEN; STOMACH; TESTIS; THYMUS; TONGUE; TONSIL; TRACHEA; URETER; URINARY BLADDER

Histopathology - The following Tissues were Not Examined:

None

Project No. 285700300102 - Individual Macroscopic and Microscopic Pathology

Animal:	2FRH	Group:	2	Sex:	Male
Species:	Dog	Strain:	Beagle		
		Dose:	0 MFC		
		Removal Reason:	Terminal Euthanasia		
		Study Day (Week) of Death:	15 (3)		

Gross Pathology Animal Details:

No animal details found

Gross Pathology Observations [Correlation]:

No observations found

Any remaining protocol required tissues, which have been examined, have no visible lesions

Gross Pathology - The following Tissues were Not Examined:

None

Histopathology Animal Details:

No animal details found

Histopathology Observations [Correlation]:

GLAND, PARATHYROID : (Note) One Of A Pair Available For Evaluation.
KIDNEY : Mineralization; minimal
TONSIL : Hemorrhage; acute, minimal

Histopathology - The following Tissues were Within Normal Limits:

ARTERY, AORTA; BONE MARROW; BONE, FEMUR; BRAIN; EPIDIDYMIS; ESOPHAGUS; EYE; GALLBLADDER; GLAND, ADRENAL; GLAND, MAMMARY; GLAND, PARATHYROID; GLAND, PITUITARY; GLAND, PROSTATE; GLAND, SALIVARY; GLAND, THYROID; HEART; LARGE INTESTINE, CECUM; LARGE INTESTINE, COLON; LARGE INTESTINE, RECTUM; LIVER; LUNG; LYMPH NODE, BRONCHIAL; LYMPH NODE, MANDIBULAR; LYMPH NODE, MESENTERIC; MUSCLE, SKELETAL; NERVE, OPTIC; NERVE, SCIATIC; PANCREAS; SKIN; SMALL INTESTINE, DUODENUM; SMALL INTESTINE, ILEUM; SMALL INTESTINE, JEJUNUM; SPINAL CORD; SPLEEN; STOMACH; TESTIS; THYMUS; TONGUE; TRACHEA; URETER; URINARY BLADDER

Histopathology - The following Tissues were Not Examined:

None

Project No. 285700300102 - Individual Macroscopic and Microscopic Pathology

Animal:	2AGF	Group:	2	Sex:	Male
Species:	Dog	Strain:	Beagle		
		Dose:	0 MFC		
		Removal Reason:	Terminal Euthanasia		
		Study Day (Week) of Death:	15 (3)		

Gross Pathology Animal Details:

No animal details found

Gross Pathology Observations [Correlation]:

TONSIL : Discoloration; red, bilateral (TGL)

Any remaining protocol required tissues, which have been examined, have no visible lesions

Gross Pathology - The following Tissues were Not Examined:

None

Histopathology Animal Details:

No animal details found

Histopathology Observations [Correlation]:

KIDNEY : Mineralization; minimal
SMALL INTESTINE, DUODENUM : Ectasia; mild, crypt
SPLEEN : Siderotic plaque; minimal
THYMUS : Cellularity, decreased; cortical, mild
TONSIL : Hemorrhage; minimal [TONSIL : Discoloration; red, bilateral (G)]

Histopathology - The following Tissues were Within Normal Limits:

ARTERY, AORTA; BONE MARROW; BONE, FEMUR; BRAIN; EPIDIDYMIS; ESOPHAGUS; EYE; GALLBLADDER; GLAND, ADRENAL; GLAND, PARATHYROID; GLAND, PITUITARY; GLAND, PROSTATE; GLAND, SALIVARY; GLAND, THYROID; HEART; LARGE INTESTINE, CECUM; LARGE INTESTINE, COLON; LARGE INTESTINE, RECTUM; LIVER; LUNG; LYMPH NODE, BRONCHIAL; LYMPH NODE, MANDIBULAR; LYMPH NODE, MESENTERIC; MUSCLE, SKELETAL; NERVE, OPTIC; NERVE, SCIATIC; PANCREAS; SKIN; SMALL INTESTINE, ILEUM; SMALL INTESTINE, JEJUNUM; SPINAL CORD; STOMACH; TESTIS; TONGUE; TRACHEA; URETER; URINARY BLADDER

Histopathology - The following Tissues were Not Examined:

GLAND, MAMMARY - Not Present In Section.

Project No. 285700300102 - Individual Macroscopic and Microscopic Pathology

Animal:	2AJE	Group:	2	Sex:	Female
Species:	Dog	Strain:	Beagle		
		Dose:	0 MFC		
		Removal Reason:	Terminal Euthanasia		
		Study Day (Week) of Death:	15 (3)		

Gross Pathology Animal Details:

No animal details found

Gross Pathology Observations [Correlation]:

LUNG : Focus; tan, right, cranial lobe : (Comment) 8x8 mm (TGL)

Any remaining protocol required tissues, which have been examined, have no visible lesions

Gross Pathology - The following Tissues were Not Examined:

None

Histopathology Animal Details:

No animal details found

Histopathology Observations [Correlation]:

KIDNEY : Mineralization; minimal
LUNG : Fibrosis; septal, focal, minimal [LUNG : Focus; tan, right, cranial lobe : (Comment) 8x8 mm (G)]
LUNG : Infiltration, mononuclear cell; alveolar, focal, minimal
OVARY : Immaturity
SMALL INTESTINE, DUODENUM : Ectasia; minimal, crypt

Histopathology - The following Tissues were Within Normal Limits:

ARTERY, AORTA; BONE MARROW; BONE, FEMUR; BRAIN; CERVIX; ESOPHAGUS; EYE; GALLBLADDER; GLAND, ADRENAL; GLAND, MAMMARY; GLAND, PARATHYROID; GLAND, PITUITARY; GLAND, SALIVARY; GLAND, THYROID; HEART; LARGE INTESTINE, CECUM; LARGE INTESTINE, COLON; LARGE INTESTINE, RECTUM; LIVER; LYMPH NODE, BRONCHIAL; LYMPH NODE, MANDIBULAR; LYMPH NODE, MESENTERIC; MUSCLE, SKELETAL; NERVE, OPTIC; NERVE, SCIATIC; OVIDUCT; PANCREAS; SKIN; SMALL INTESTINE, ILEUM; SMALL INTESTINE, JEJUNUM; SPINAL CORD; SPLEEN; STOMACH; THYMUS; TONGUE; TONSIL; TRACHEA; URETER; URINARY BLADDER; UTERUS; VAGINA

Histopathology - The following Tissues were Not Examined:

None

Project No. 285700300102 - Individual Macroscopic and Microscopic Pathology

Animal:	2FLE	Group:	2	Sex:	Female
Species:	Dog	Strain:	Beagle		
		Dose:	0 MFC		
		Removal Reason:	Terminal Euthanasia		
		Study Day (Week) of Death:	15 (3)		

Gross Pathology Animal Details:

No animal details found

Gross Pathology Observations [Correlation]:

No observations found

Any remaining protocol required tissues, which have been examined, have no visible lesions

Gross Pathology - The following Tissues were Not Examined:

None

Histopathology Animal Details:

No animal details found

Histopathology Observations [Correlation]:

KIDNEY : Mineralization; minimal
LYMPH NODE, BRONCHIAL : Hyperplasia; mild, germinal center
OVARY : Immaturity
SMALL INTESTINE, ILEUM : Atrophy; mucosal, mild

Histopathology - The following Tissues were Within Normal Limits:

ARTERY, AORTA; BONE MARROW; BONE, FEMUR; BRAIN; CERVIX; ESOPHAGUS; EYE; GALLBLADDER; GLAND, ADRENAL; GLAND, MAMMARY; GLAND, PARATHYROID; GLAND, PITUITARY; GLAND, SALIVARY; GLAND, THYROID; HEART; LARGE INTESTINE, CECUM; LARGE INTESTINE, COLON; LARGE INTESTINE, RECTUM; LIVER; LUNG; LYMPH NODE, MANDIBULAR; LYMPH NODE, MESENTERIC; MUSCLE, SKELETAL; NERVE, OPTIC; NERVE, SCIATIC; OVIDUCT; PANCREAS; SKIN; SMALL INTESTINE, DUODENUM; SMALL INTESTINE, JEJUNUM; SPINAL CORD; SPLEEN; STOMACH; THYMUS; TONGUE; TONSIL; TRACHEA; URETER; URINARY BLADDER; UTERUS; VAGINA

Histopathology - The following Tissues were Not Examined:

None

Project No. 285700300102 - Individual Macroscopic and Microscopic Pathology

Animal:	2ZQE	Group:	2	Sex:	Female
Species:	Dog	Strain:	Beagle		
		Dose:	0 MFC		
		Removal Reason:	Terminal Euthanasia		
		Study Day (Week) of Death:	15 (3)		

Gross Pathology Animal Details:

No animal details found

Gross Pathology Observations [Correlation]:

OVARY : Enlargement; left (TGL)

Any remaining protocol required tissues, which have been examined, have no visible lesions

Gross Pathology - The following Tissues were Not Examined:

None

Histopathology Animal Details:

No animal details found

Histopathology Observations [Correlation]:

KIDNEY : Vacuolation; tubular, minimal
LUNG : Foreign material; alveolar, focal, minimal
LUNG : Infiltration, mixed cell; alveolar, focal, mild
LYMPH NODE, BRONCHIAL : Hemorrhage; acute, minimal
LYMPH NODE, BRONCHIAL : Hyperplasia; minimal, germinal center
LYMPH NODE, MESENTERIC : Erythrophagocytosis; minimal
OVARY : (Comment) Normal cystic structures caused enlargement.
THYMUS : Cellularity, decreased; cortical, mild
TONSIL : Hemorrhage; acute, minimal
NO CORRELATE : No correlating lesion [OVARY : Enlargement; left (G)]

Histopathology - The following Tissues were Within Normal Limits:

ARTERY, AORTA; BONE MARROW; BONE, FEMUR; BRAIN; CERVIX; ESOPHAGUS; EYE; GALLBLADDER; GLAND, ADRENAL; GLAND, MAMMARY; GLAND, PARATHYROID; GLAND, PITUITARY; GLAND, SALIVARY; GLAND, THYROID; HEART; LARGE INTESTINE, CECUM; LARGE INTESTINE, COLON; LARGE INTESTINE, RECTUM; LIVER; LYMPH NODE, MANDIBULAR; MUSCLE, SKELETAL; NERVE, OPTIC; NERVE, SCIATIC; OVARY; OVIDUCT; PANCREAS; SKIN; SMALL INTESTINE, DUODENUM; SMALL INTESTINE, ILEUM; SMALL INTESTINE, JEJUNUM; SPINAL CORD; SPLEEN; STOMACH; TONGUE; TRACHEA; URETER; URINARY BLADDER; UTERUS; VAGINA

Histopathology - The following Tissues were Not Examined:

None

Project No. 285700300102 - Individual Macroscopic and Microscopic Pathology

Animal:	3DWH	Group:	3	Sex:	Male
Species:	Dog	Strain:	Beagle		
		Dose:	0.25 MFC APN01		
		Removal Reason:	Terminal Euthanasia		
		Study Day (Week) of Death:	15 (3)		

Gross Pathology Animal Details:

No animal details found

Gross Pathology Observations [Correlation]:

No observations found

Any remaining protocol required tissues, which have been examined, have no visible lesions

Gross Pathology - The following Tissues were Not Examined:

None

Histopathology Animal Details:

No animal details found

Histopathology Observations [Correlation]:

EPIDIDYMIS : Cellular debris; minimal
GLAND, PARATHYROID : (Note) One Of A Pair Available For Evaluation.
GLAND, PITUITARY : Cyst
KIDNEY : Mineralization; minimal
LUNG : Foreign material; alveolar, focal, minimal
LUNG : Infiltration, mixed cell; alveolar, focal, minimal
THYMUS : Cellularity, decreased; cortical, mild

Histopathology - The following Tissues were Within Normal Limits:

ARTERY, AORTA; BONE MARROW; BONE, FEMUR; BRAIN; ESOPHAGUS; EYE; GALLBLADDER; GLAND, ADRENAL; GLAND, PARATHYROID; GLAND, PROSTATE; GLAND, SALIVARY; GLAND, THYROID; HEART; LARGE INTESTINE, CECUM; LARGE INTESTINE, COLON; LARGE INTESTINE, RECTUM; LIVER; LYMPH NODE, BRONCHIAL; LYMPH NODE, MANDIBULAR; LYMPH NODE, MESENTERIC; MUSCLE, SKELETAL; NERVE, OPTIC; NERVE, SCIATIC; PANCREAS; SKIN; SMALL INTESTINE, DUODENUM; SMALL INTESTINE, ILEUM; SMALL INTESTINE, JEJUNUM; SPINAL CORD; SPLEEN; STOMACH; TESTIS; TONGUE; TONSIL; TRACHEA; URETER; URINARY BLADDER

Histopathology - The following Tissues were Not Examined:

GLAND, MAMMARY - Not Present In Section.

Project No. 285700300102 - Individual Macroscopic and Microscopic Pathology

Animal:	3THF	Group:	3	Sex:	Male
Species:	Dog	Strain:	Beagle		
		Dose:	0.25 MFC APN01		
		Removal Reason:	Terminal Euthanasia		
		Study Day (Week) of Death:	15 (3)		

Gross Pathology Animal Details:

No animal details found

Gross Pathology Observations [Correlation]:

TONSIL : Discoloration; red, bilateral (TGL)

Any remaining protocol required tissues, which have been examined, have no visible lesions

Gross Pathology - The following Tissues were Not Examined:

None

Histopathology Animal Details:

No animal details found

Histopathology Observations [Correlation]:

GLAND, PARATHYROID : (Note) One Of A Pair Available For Evaluation.
KIDNEY : Mineralization; minimal
THYMUS : Cellularity, decreased; cortical, moderate
NO CORRELATE : No correlating lesion [TONSIL : Discoloration; red, bilateral (G)]

Histopathology - The following Tissues were Within Normal Limits:

ARTERY, AORTA; BONE MARROW; BONE, FEMUR; BRAIN; EPIDIDYMIS; ESOPHAGUS; EYE; GALLBLADDER; GLAND, ADRENAL; GLAND, MAMMARY; GLAND, PARATHYROID; GLAND, PITUITARY; GLAND, PROSTATE; GLAND, SALIVARY; GLAND, THYROID; HEART; LARGE INTESTINE, CECUM; LARGE INTESTINE, COLON; LARGE INTESTINE, RECTUM; LIVER; LUNG; LYMPH NODE, BRONCHIAL; LYMPH NODE, MANDIBULAR; LYMPH NODE, MESENTERIC; MUSCLE, SKELETAL; NERVE, OPTIC; NERVE, SCIATIC; PANCREAS; SKIN; SMALL INTESTINE, DUODENUM; SMALL INTESTINE, ILEUM; SMALL INTESTINE, JEJUNUM; SPINAL CORD; SPLEEN; STOMACH; TESTIS; TONGUE; TONSIL; TRACHEA; URETER; URINARY BLADDER

Histopathology - The following Tissues were Not Examined:

None

Project No. 285700300102 - Individual Macroscopic and Microscopic Pathology

Animal:	3TYF	Group:	3	Sex:	Male
Species:	Dog	Strain:	Beagle		
		Dose:	0.25 MFC APN01		
		Removal Reason:	Terminal Euthanasia		
		Study Day (Week) of Death:	15 (3)		

Gross Pathology Animal Details:

No animal details found

Gross Pathology Observations [Correlation]:

No observations found

Any remaining protocol required tissues, which have been examined, have no visible lesions

Gross Pathology - The following Tissues were Not Examined:

None

Histopathology Animal Details:

No animal details found

Histopathology Observations [Correlation]:

KIDNEY : Mineralization; minimal
LUNG : Hypertrophy; focal, mild, pneumocyte, type 2
LYMPH NODE, MESENTERIC : Erythrophagocytosis; mild

Histopathology - The following Tissues were Within Normal Limits:

ARTERY, AORTA; BONE MARROW; BONE, FEMUR; BRAIN; EPIDIDYMIS; ESOPHAGUS; EYE; GALLBLADDER; GLAND, ADRENAL; GLAND, MAMMARY; GLAND, PARATHYROID; GLAND, PITUITARY; GLAND, PROSTATE; GLAND, SALIVARY; GLAND, THYROID; HEART; LARGE INTESTINE, CECUM; LARGE INTESTINE, COLON; LARGE INTESTINE, RECTUM; LIVER; LYMPH NODE, BRONCHIAL; LYMPH NODE, MANDIBULAR; MUSCLE, SKELETAL; NERVE, OPTIC; NERVE, SCIATIC; PANCREAS; SKIN; SMALL INTESTINE, DUODENUM; SMALL INTESTINE, ILEUM; SMALL INTESTINE, JEJUNUM; SPINAL CORD; SPLEEN; STOMACH; TESTIS; THYMUS; TONGUE; TONSIL; TRACHEA; URETER; URINARY BLADDER

Histopathology - The following Tissues were Not Examined:

None

Project No. 285700300102 - Individual Macroscopic and Microscopic Pathology

Animal:	3CVG	Group:	3	Sex:	Female
Species:	Dog	Strain:	Beagle		
		Dose:	0.25 MFC APN01		
		Removal Reason:	Terminal Euthanasia		
		Study Day (Week) of Death:	15 (3)		

Gross Pathology Animal Details:

No animal details found

Gross Pathology Observations [Correlation]:

No observations found

Any remaining protocol required tissues, which have been examined, have no visible lesions

Gross Pathology - The following Tissues were Not Examined:

None

Histopathology Animal Details:

No animal details found

Histopathology Observations [Correlation]:

ARTERY, AORTA : Degeneration; mild : (Comment) outflow tract on heart section
ARTERY, AORTA : Mineralization; minimal : (Comment) outflow tract on heart section.
KIDNEY : Mineralization; minimal
OVARY : Immaturity

Histopathology - The following Tissues were Within Normal Limits:

BONE MARROW; BONE, FEMUR; BRAIN; CERVIX; ESOPHAGUS; EYE; GALLBLADDER; GLAND, ADRENAL; GLAND, MAMMARY; GLAND, PARATHYROID; GLAND, PITUITARY; GLAND, SALIVARY; GLAND, THYROID; HEART; LARGE INTESTINE, CECUM; LARGE INTESTINE, COLON; LARGE INTESTINE, RECTUM; LIVER; LUNG; LYMPH NODE, BRONCHIAL; LYMPH NODE, MANDIBULAR; LYMPH NODE, MESENTERIC; MUSCLE, SKELETAL; NERVE, OPTIC; NERVE, SCIATIC; OVIDUCT; PANCREAS; SKIN; SMALL INTESTINE, DUODENUM; SMALL INTESTINE, ILEUM; SMALL INTESTINE, JEJUNUM; SPINAL CORD; SPLEEN; STOMACH; THYMUS; TONGUE; TONSIL; TRACHEA; URETER; URINARY BLADDER; UTERUS; VAGINA

Histopathology - The following Tissues were Not Examined:

None

Project No. 285700300102 - Individual Macroscopic and Microscopic Pathology

Animal:	3FKE	Group:	3	Sex:	Female
Species:	Dog	Strain:	Beagle		
		Dose:	0.25 MFC APN01		
		Removal Reason:	Terminal Euthanasia		
		Study Day (Week) of Death:	15 (3)		

Gross Pathology Animal Details:

No animal details found

Gross Pathology Observations [Correlation]:

No observations found

Any remaining protocol required tissues, which have been examined, have no visible lesions

Gross Pathology - The following Tissues were Not Examined:

None

Histopathology Animal Details:

No animal details found

Histopathology Observations [Correlation]:

CERVIX : Hyperplasia; minimal
KIDNEY : Mineralization; minimal
LYMPH NODE, BRONCHIAL : Hyperplasia; minimal, germinal center
OVARY : Immaturity

Histopathology - The following Tissues were Within Normal Limits:

ARTERY, AORTA; BONE MARROW; BONE, FEMUR; BRAIN; ESOPHAGUS; EYE; GALLBLADDER; GLAND, ADRENAL; GLAND, MAMMARY; GLAND, PARATHYROID; GLAND, PITUITARY; GLAND, SALIVARY; GLAND, THYROID; HEART; LARGE INTESTINE, CECUM; LARGE INTESTINE, COLON; LARGE INTESTINE, RECTUM; LIVER; LUNG; LYMPH NODE, MANDIBULAR; LYMPH NODE, MESENTERIC; MUSCLE, SKELETAL; NERVE, OPTIC; NERVE, SCIATIC; OVIDUCT; PANCREAS; SKIN; SMALL INTESTINE, DUODENUM; SMALL INTESTINE, ILEUM; SMALL INTESTINE, JEJUNUM; SPINAL CORD; SPLEEN; STOMACH; THYMUS; TONGUE; TONSIL; TRACHEA; URETER; URINARY BLADDER; UTERUS; VAGINA

Histopathology - The following Tissues were Not Examined:

None

Project No. 285700300102 - Individual Macroscopic and Microscopic Pathology

Animal:	3ZRE	Group:	3	Sex:	Female
Species:	Dog	Strain:	Beagle		
		Dose:	0.25 MFC APN01		
		Removal Reason:	Terminal Euthanasia		
		Study Day (Week) of Death:	15 (3)		

Gross Pathology Animal Details:

No animal details found

Gross Pathology Observations [Correlation]:

LYMPH NODE, BRONCHIAL : Enlargement (TGL)

Any remaining protocol required tissues, which have been examined, have no visible lesions

Gross Pathology - The following Tissues were Not Examined:

None

Histopathology Animal Details:

No animal details found

Histopathology Observations [Correlation]:

BONE MARROW : Myeloid to erythroid ratio, increased; mild
KIDNEY : Mineralization; minimal
LUNG : Infiltration, mixed cell; alveolar, diffuse, moderate
LYMPH NODE, BRONCHIAL : Hyperplasia; minimal, germinal center [LYMPH NODE, BRONCHIAL : Enlargement (G)]
LYMPH NODE, BRONCHIAL : Infiltration, mixed cell; minimal
OVARY : Immaturity

Histopathology - The following Tissues were Within Normal Limits:

ARTERY, AORTA; BONE, FEMUR; BRAIN; CERVIX; ESOPHAGUS; EYE; GALLBLADDER; GLAND, ADRENAL; GLAND, MAMMARY; GLAND, PARATHYROID; GLAND, PITUITARY; GLAND, SALIVARY; GLAND, THYROID; HEART; LARGE INTESTINE, CECUM; LARGE INTESTINE, COLON; LARGE INTESTINE, RECTUM; LIVER; LYMPH NODE, MANDIBULAR; LYMPH NODE, MESENTERIC; MUSCLE, SKELETAL; NERVE, OPTIC; NERVE, SCIATIC; PANCREAS; SKIN; SMALL INTESTINE, DUODENUM; SMALL INTESTINE, ILEUM; SMALL INTESTINE, JEJUNUM; SPINAL CORD; SPLEEN; STOMACH; THYMUS; TONGUE; TONSIL; TRACHEA; URETER; URINARY BLADDER; UTERUS; VAGINA

Histopathology - The following Tissues were Not Examined:

OVIDUCT - Not Present In Section.

Project No. 285700300102 - Individual Macroscopic and Microscopic Pathology

Animal:	4EAH	Group:	4	Sex:	Male
Species:	Dog	Strain:	Beagle		
		Dose:	0.5 MFC APN01		
		Removal Reason:	Terminal Euthanasia		
		Study Day (Week) of Death:	15 (3)		

Gross Pathology Animal Details:

No animal details found

Gross Pathology Observations [Correlation]:

No observations found

Any remaining protocol required tissues, which have been examined, have no visible lesions

Gross Pathology - The following Tissues were Not Examined:

None

Histopathology Animal Details:

No animal details found

Histopathology Observations [Correlation]:

EPIDIDYMIS : Cellular debris; minimal
GLAND, PITUITARY : Cyst
KIDNEY : Mineralization; minimal
SMALL INTESTINE, ILEUM : Atrophy; mucosal, mild
THYMUS : Cellularity, decreased; cortical, moderate

Histopathology - The following Tissues were Within Normal Limits:

ARTERY, AORTA; BONE MARROW; BONE, FEMUR; BRAIN; ESOPHAGUS; EYE; GALLBLADDER; GLAND, ADRENAL; GLAND, MAMMARY; GLAND, PARATHYROID; GLAND, PROSTATE; GLAND, SALIVARY; GLAND, THYROID; HEART; LARGE INTESTINE, CECUM; LARGE INTESTINE, COLON; LARGE INTESTINE, RECTUM; LIVER; LUNG; LYMPH NODE, BRONCHIAL; LYMPH NODE, MANDIBULAR; LYMPH NODE, MESENTERIC; MUSCLE, SKELETAL; NERVE, OPTIC; NERVE, SCIATIC; PANCREAS; SKIN; SMALL INTESTINE, DUODENUM; SMALL INTESTINE, JEJUNUM; SPINAL CORD; SPLEEN; STOMACH; TESTIS; TONGUE; TONSIL; TRACHEA; URETER; URINARY BLADDER

Histopathology - The following Tissues were Not Examined:

None

Project No. 285700300102 - Individual Macroscopic and Microscopic Pathology

Animal:	4JLH	Group:	4	Sex:	Male
Species:	Dog	Strain:	Beagle		
		Dose:	0.5 MFC APN01		
		Removal Reason:	Terminal Euthanasia		
		Study Day (Week) of Death:	15 (3)		

Gross Pathology Animal Details:

No animal details found

Gross Pathology Observations [Correlation]:

No observations found

Any remaining protocol required tissues, which have been examined, have no visible lesions

Gross Pathology - The following Tissues were Not Examined:

None

Histopathology Animal Details:

No animal details found

Histopathology Observations [Correlation]:

EPIDIDYMIS : Cellular debris; minimal
KIDNEY : Mineralization; minimal

Histopathology - The following Tissues were Within Normal Limits:

ARTERY, AORTA; BONE MARROW; BONE, FEMUR; BRAIN; ESOPHAGUS; EYE; GALLBLADDER; GLAND, ADRENAL; GLAND, MAMMARY; GLAND, PARATHYROID; GLAND, PITUITARY; GLAND, PROSTATE; GLAND, SALIVARY; GLAND, THYROID; HEART; LARGE INTESTINE, CECUM; LARGE INTESTINE, COLON; LARGE INTESTINE, RECTUM; LIVER; LUNG; LYMPH NODE, BRONCHIAL; LYMPH NODE, MANDIBULAR; LYMPH NODE, MESENTERIC; MUSCLE, SKELETAL; NERVE, OPTIC; NERVE, SCIATIC; PANCREAS; SKIN; SMALL INTESTINE, DUODENUM; SMALL INTESTINE, ILEUM; SMALL INTESTINE, JEJUNUM; SPINAL CORD; SPLEEN; STOMACH; TESTIS; THYMUS; TONGUE; TONSIL; TRACHEA; URETER; URINARY BLADDER

Histopathology - The following Tissues were Not Examined:

None

Project No. 285700300102 - Individual Macroscopic and Microscopic Pathology

Animal:	4YJF	Group:	4	Sex:	Male
Species:	Dog	Strain:	Beagle		
		Dose:	0.5 MFC APN01		
		Removal Reason:	Terminal Euthanasia		
		Study Day (Week) of Death:	15 (3)		

Gross Pathology Animal Details:

No animal details found

Gross Pathology Observations [Correlation]:

No observations found

Any remaining protocol required tissues, which have been examined, have no visible lesions

Gross Pathology - The following Tissues were Not Examined:

None

Histopathology Animal Details:

No animal details found

Histopathology Observations [Correlation]:

KIDNEY : Mineralization; minimal

Histopathology - The following Tissues were Within Normal Limits:

ARTERY, AORTA; BONE MARROW; BONE, FEMUR; BRAIN; EPIDIDYMIS; ESOPHAGUS; EYE; GALLBLADDER; GLAND, ADRENAL; GLAND, MAMMARY; GLAND, PARATHYROID; GLAND, PITUITARY; GLAND, PROSTATE; GLAND, SALIVARY; GLAND, THYROID; HEART; LARGE INTESTINE, CECUM; LARGE INTESTINE, COLON; LARGE INTESTINE, RECTUM; LIVER; LUNG; LYMPH NODE, BRONCHIAL; LYMPH NODE, MANDIBULAR; LYMPH NODE, MESENTERIC; MUSCLE, SKELETAL; NERVE, OPTIC; NERVE, SCIATIC; PANCREAS; SKIN; SMALL INTESTINE, DUODENUM; SMALL INTESTINE, ILEUM; SMALL INTESTINE, JEJUNUM; SPINAL CORD; SPLEEN; STOMACH; TESTIS; THYMUS; TONGUE; TONSIL; TRACHEA; URETER; URINARY BLADDER

Histopathology - The following Tissues were Not Examined:

None

Project No. 285700300102 - Individual Macroscopic and Microscopic Pathology

Animal:	4ACE	Group:	4	Sex:	Female
Species:	Dog	Strain:	Beagle		
		Dose:	0.5 MFC APN01		
		Removal Reason:	Terminal Euthanasia		
		Study Day (Week) of Death:	15 (3)		

Gross Pathology Animal Details:

No animal details found

Gross Pathology Observations [Correlation]:

No observations found

Any remaining protocol required tissues, which have been examined, have no visible lesions

Gross Pathology - The following Tissues were Not Examined:

None

Histopathology Animal Details:

No animal details found

Histopathology Observations [Correlation]:

KIDNEY : Mineralization; minimal
OVARY : Immaturity
OVIDUCT : (Note) One Of A Pair Available For Evaluation.
SKIN : Granuloma; minimal

Histopathology - The following Tissues were Within Normal Limits:

ARTERY, AORTA; BONE MARROW; BONE, FEMUR; BRAIN; CERVIX; ESOPHAGUS; EYE; GALLBLADDER; GLAND, ADRENAL; GLAND, MAMMARY; GLAND, PARATHYROID; GLAND, PITUITARY; GLAND, SALIVARY; GLAND, THYROID; HEART; LARGE INTESTINE, CECUM; LARGE INTESTINE, COLON; LARGE INTESTINE, RECTUM; LIVER; LUNG; LYMPH NODE, BRONCHIAL; LYMPH NODE, MANDIBULAR; LYMPH NODE, MESENTERIC; MUSCLE, SKELETAL; NERVE, OPTIC; NERVE, SCIATIC; OVIDUCT; PANCREAS; SMALL INTESTINE, DUODENUM; SMALL INTESTINE, ILEUM; SMALL INTESTINE, JEJUNUM; SPINAL CORD; SPLEEN; STOMACH; THYMUS; TONGUE; TONSIL; TRACHEA; URETER; URINARY BLADDER; UTERUS; VAGINA

Histopathology - The following Tissues were Not Examined:

None

Project No. 285700300102 - Individual Macroscopic and Microscopic Pathology

Animal:	4CAE	Group:	4	Sex:	Female
Species:	Dog	Strain:	Beagle		
		Dose:	0.5 MFC APN01		
		Removal Reason:	Terminal Euthanasia		
		Study Day (Week) of Death:	15 (3)		

Gross Pathology Animal Details:

No animal details found

Gross Pathology Observations [Correlation]:

No observations found

Any remaining protocol required tissues, which have been examined, have no visible lesions

Gross Pathology - The following Tissues were Not Examined:

None

Histopathology Animal Details:

No animal details found

Histopathology Observations [Correlation]:

KIDNEY : Mineralization; minimal
LUNG : Foreign material; alveolar, focal, minimal
LUNG : Infiltration, mixed cell; alveolar, focal, minimal
LYMPH NODE, BRONCHIAL : Hyperplasia; minimal, germinal center
OVARY : Immaturity

Histopathology - The following Tissues were Within Normal Limits:

ARTERY, AORTA; BONE MARROW; BONE, FEMUR; BRAIN; CERVIX; ESOPHAGUS; EYE; GALLBLADDER; GLAND, ADRENAL; GLAND, MAMMARY; GLAND, PARATHYROID; GLAND, PITUITARY; GLAND, SALIVARY; GLAND, THYROID; HEART; LARGE INTESTINE, CECUM; LARGE INTESTINE, COLON; LARGE INTESTINE, RECTUM; LIVER; LYMPH NODE, MANDIBULAR; LYMPH NODE, MESENTERIC; MUSCLE, SKELETAL; NERVE, OPTIC; NERVE, SCIATIC; OVIDUCT; PANCREAS; SKIN; SMALL INTESTINE, DUODENUM; SMALL INTESTINE, ILEUM; SMALL INTESTINE, JEJUNUM; SPINAL CORD; SPLEEN; STOMACH; THYMUS; TONGUE; TONSIL; TRACHEA; URETER; URINARY BLADDER; UTERUS; VAGINA

Histopathology - The following Tissues were Not Examined:

None

Project No. 285700300102 - Individual Macroscopic and Microscopic Pathology

Animal:	4EVE	Group:	4	Sex:	Female
Species:	Dog	Strain:	Beagle		
		Dose:	0.5 MFC APN01		
		Removal Reason:	Terminal Euthanasia		
		Study Day (Week) of Death:	15 (3)		

Gross Pathology Animal Details:

No animal details found

Gross Pathology Observations [Correlation]:

No observations found

Any remaining protocol required tissues, which have been examined, have no visible lesions

Gross Pathology - The following Tissues were Not Examined:

None

Histopathology Animal Details:

No animal details found

Histopathology Observations [Correlation]:

GLAND, PARATHYROID : (Note) One Of A Pair Available For Evaluation.
KIDNEY : Mineralization; minimal
LUNG : Fibrosis; pleural, mild
OVARY : Immaturity
SMALL INTESTINE, ILEUM : Lymphangiectasia; villous, mild

Histopathology - The following Tissues were Within Normal Limits:

ARTERY, AORTA; BONE MARROW; BONE, FEMUR; BRAIN; CERVIX; ESOPHAGUS; EYE; GALLBLADDER; GLAND, ADRENAL; GLAND, MAMMARY; GLAND, PARATHYROID; GLAND, PITUITARY; GLAND, SALIVARY; GLAND, THYROID; HEART; LARGE INTESTINE, CECUM; LARGE INTESTINE, COLON; LARGE INTESTINE, RECTUM; LIVER; LYMPH NODE, BRONCHIAL; LYMPH NODE, MANDIBULAR; LYMPH NODE, MESENTERIC; MUSCLE, SKELETAL; NERVE, OPTIC; NERVE, SCIATIC; OVIDUCT; PANCREAS; SKIN; SMALL INTESTINE, DUODENUM; SMALL INTESTINE, JEJUNUM; SPINAL CORD; SPLEEN; STOMACH; THYMUS; TONGUE; TONSIL; TRACHEA; URETER; URINARY BLADDER; UTERUS; VAGINA

Histopathology - The following Tissues were Not Examined:

None

Project No. 285700300102 - Individual Macroscopic and Microscopic Pathology

Animal:	5GPH	Group:	5	Sex:	Male
Species:	Dog	Strain:	Beagle		
		Dose:	1 MFC APN01		
		Removal Reason:	Terminal Euthanasia		
		Study Day (Week) of Death:	15 (3)		

Gross Pathology Animal Details:

No animal details found

Gross Pathology Observations [Correlation]:

No observations found

Any remaining protocol required tissues, which have been examined, have no visible lesions

Gross Pathology - The following Tissues were Not Examined:

None

Histopathology Animal Details:

No animal details found

Histopathology Observations [Correlation]:

BONE, FEMUR : Degeneration; minimal, physis
KIDNEY : Mineralization; minimal
STOMACH : Mineralization; mucosal, submucosal, minimal
TONSIL : Hemorrhage; acute, minimal

Histopathology - The following Tissues were Within Normal Limits:

ARTERY, AORTA; BONE MARROW; BRAIN; EPIDIDYMIS; ESOPHAGUS; EYE; GALLBLADDER; GLAND, ADRENAL; GLAND, PARATHYROID; GLAND, PITUITARY; GLAND, PROSTATE; GLAND, SALIVARY; GLAND, THYROID; HEART; LARGE INTESTINE, CECUM; LARGE INTESTINE, COLON; LARGE INTESTINE, RECTUM; LIVER; LUNG; LYMPH NODE, BRONCHIAL; LYMPH NODE, MANDIBULAR; LYMPH NODE, MESENTERIC; MUSCLE, SKELETAL; NERVE, OPTIC; NERVE, SCIATIC; PANCREAS; SKIN; SMALL INTESTINE, DUODENUM; SMALL INTESTINE, ILEUM; SMALL INTESTINE, JEJUNUM; SPINAL CORD; SPLEEN; TESTIS; THYMUS; TONGUE; TRACHEA; URETER; URINARY BLADDER

Histopathology - The following Tissues were Not Examined:

GLAND, MAMMARY - Not Present In Section.

Project No. 285700300102 - Individual Macroscopic and Microscopic Pathology

Animal:	5ZOF	Group:	5	Sex:	Male
Species:	Dog	Strain:	Beagle		
		Dose:	1 MFC APN01		
		Removal Reason:	Terminal Euthanasia		
		Study Day (Week) of Death:	15 (3)		

Gross Pathology Animal Details:

No animal details found

Gross Pathology Observations [Correlation]:

No observations found

Any remaining protocol required tissues, which have been examined, have no visible lesions

Gross Pathology - The following Tissues were Not Examined:

None

Histopathology Animal Details:

No animal details found

Histopathology Observations [Correlation]:

EPIDIDYMIS : Cellular debris; minimal
LUNG : Infiltration, mononuclear cell; perivascular, minimal
LYMPH NODE, BRONCHIAL : Hyperplasia; minimal, germinal center
LYMPH NODE, MESENTERIC : Erythrophagocytosis; mild

Histopathology - The following Tissues were Within Normal Limits:

ARTERY, AORTA; BONE MARROW; BONE, FEMUR; BRAIN; ESOPHAGUS; EYE; GALLBLADDER; GLAND, ADRENAL; GLAND, MAMMARY; GLAND, PITUITARY; GLAND, PROSTATE; GLAND, SALIVARY; GLAND, THYROID; HEART; KIDNEY; LARGE INTESTINE, CECUM; LARGE INTESTINE, COLON; LARGE INTESTINE, RECTUM; LIVER; LYMPH NODE, MANDIBULAR; MUSCLE, SKELETAL; NERVE, OPTIC; NERVE, SCIATIC; PANCREAS; SKIN; SMALL INTESTINE, DUODENUM; SMALL INTESTINE, ILEUM; SMALL INTESTINE, JEJUNUM; SPINAL CORD; SPLEEN; STOMACH; TESTIS; THYMUS; TONGUE; TONSIL; TRACHEA; URETER; URINARY BLADDER

Histopathology - The following Tissues were Not Examined:

GLAND, PARATHYROID - Not Present In Section.

Project No. 285700300102 - Individual Macroscopic and Microscopic Pathology

Animal:	5ZTF	Group:	5	Sex:	Male
Species:	Dog	Strain:	Beagle		
		Dose:	1 MFC APN01		
		Removal Reason:	Terminal Euthanasia		
		Study Day (Week) of Death:	15 (3)		

Gross Pathology Animal Details:

No animal details found

Gross Pathology Observations [Correlation]:

No observations found

Any remaining protocol required tissues, which have been examined, have no visible lesions

Gross Pathology - The following Tissues were Not Examined:

None

Histopathology Animal Details:

No animal details found

Histopathology Observations [Correlation]:

EPIDIDYMIS : Cellular debris; mild
KIDNEY : Mineralization; minimal
KIDNEY : Infiltration, mixed cell; cortical, mild
LYMPH NODE, BRONCHIAL : Hyperplasia; minimal, germinal center
LYMPH NODE, MESENTERIC : Erythrophagocytosis; minimal
TESTIS : Degeneration; mild, seminiferous tubule
TRACHEA : Metaplasia, squamous; mild, carina

Histopathology - The following Tissues were Within Normal Limits:

ARTERY, AORTA; BONE MARROW; BONE, FEMUR; BRAIN; ESOPHAGUS; EYE; GALLBLADDER; GLAND, ADRENAL; GLAND, MAMMARY; GLAND, PARATHYROID; GLAND, PITUITARY; GLAND, PROSTATE; GLAND, SALIVARY; GLAND, THYROID; HEART; LARGE INTESTINE, CECUM; LARGE INTESTINE, COLON; LARGE INTESTINE, RECTUM; LIVER; LUNG; LYMPH NODE, MANDIBULAR; MUSCLE, SKELETAL; NERVE, OPTIC; NERVE, SCIATIC; PANCREAS; SKIN; SMALL INTESTINE, DUODENUM; SMALL INTESTINE, ILEUM; SMALL INTESTINE, JEJUNUM; SPINAL CORD; SPLEEN; STOMACH; THYMUS; TONGUE; TONSIL; URETER; URINARY BLADDER

Histopathology - The following Tissues were Not Examined:

None

Project No. 285700300102 - Individual Macroscopic and Microscopic Pathology

Animal:	5AIE	Group:	5	Sex:	Female
Species:	Dog	Strain:	Beagle		
		Dose:	1 MFC APN01		
		Removal Reason:	Terminal Euthanasia		
		Study Day (Week) of Death:	15 (3)		

Gross Pathology Animal Details:

No animal details found

Gross Pathology Observations [Correlation]:

TONSIL : Discoloration; red, bilateral (TGL)

Any remaining protocol required tissues, which have been examined, have no visible lesions

Gross Pathology - The following Tissues were Not Examined:

None

Histopathology Animal Details:

No animal details found

Histopathology Observations [Correlation]:

KIDNEY : Mineralization; minimal
LUNG : Fibrosis; pleural, minimal
LYMPH NODE, BRONCHIAL : Hyperplasia; minimal, germinal center
OVARY : Immaturity
SMALL INTESTINE, DUODENUM : Ectasia; minimal, crypt
TONSIL : Hemorrhage; acute, minimal [TONSIL : Discoloration; red, bilateral (G)]

Histopathology - The following Tissues were Within Normal Limits:

ARTERY, AORTA; BONE MARROW; BONE, FEMUR; BRAIN; CERVIX; ESOPHAGUS; EYE; GALLBLADDER; GLAND, ADRENAL; GLAND, MAMMARY; GLAND, PARATHYROID; GLAND, PITUITARY; GLAND, SALIVARY; GLAND, THYROID; HEART; LARGE INTESTINE, CECUM; LARGE INTESTINE, COLON; LARGE INTESTINE, RECTUM; LIVER; LYMPH NODE, MANDIBULAR; LYMPH NODE, MESENTERIC; MUSCLE, SKELETAL; NERVE, OPTIC; NERVE, SCIATIC; OVIDUCT; PANCREAS; SKIN; SMALL INTESTINE, ILEUM; SMALL INTESTINE, JEJUNUM; SPINAL CORD; SPLEEN; STOMACH; THYMUS; TONGUE; TRACHEA; URETER; URINARY BLADDER; UTERUS; VAGINA

Histopathology - The following Tissues were Not Examined:

None

Project No. 285700300102 - Individual Macroscopic and Microscopic Pathology

Animal:	5CGE	Group:	5	Sex:	Female
Species:	Dog	Strain:	Beagle		
		Dose:	1 MFC APN01		
		Removal Reason:	Terminal Euthanasia		
		Study Day (Week) of Death:	15 (3)		

Gross Pathology Animal Details:

No animal details found

Gross Pathology Observations [Correlation]:

No observations found

Any remaining protocol required tissues, which have been examined, have no visible lesions

Gross Pathology - The following Tissues were Not Examined:

None

Histopathology Animal Details:

No animal details found

Histopathology Observations [Correlation]:

GLAND, PARATHYROID : (Note) One Of A Pair Available For Evaluation.
KIDNEY : Mineralization; minimal
LUNG : Mineralization; submucosal, minimal, bronchus
LUNG : Infiltration, mixed cell; alveolar, focal, minimal
LYMPH NODE, BRONCHIAL : Hyperplasia; minimal, germinal center
LYMPH NODE, MESENTERIC : Erythrophagocytosis; mild
OVARY : Immaturity
OVIDUCT : (Note) One Of A Pair Available For Evaluation.
SMALL INTESTINE, JEJUNUM : Atrophy; mucosal, mild

Histopathology - The following Tissues were Within Normal Limits:

ARTERY, AORTA; BONE MARROW; BONE, FEMUR; BRAIN; CERVIX; ESOPHAGUS; EYE; GALLBLADDER; GLAND, ADRENAL; GLAND, MAMMARY; GLAND, PARATHYROID; GLAND, PITUITARY; GLAND, SALIVARY; GLAND, THYROID; HEART; LARGE INTESTINE, CECUM; LARGE INTESTINE, COLON; LARGE INTESTINE, RECTUM; LIVER; LYMPH NODE, MANDIBULAR; MUSCLE, SKELETAL; NERVE, OPTIC; NERVE, SCIATIC; OVIDUCT; PANCREAS; SKIN; SMALL INTESTINE, DUODENUM; SMALL INTESTINE, ILEUM; SPINAL CORD; SPLEEN; STOMACH; THYMUS; TONGUE; TONSIL; TRACHEA; URETER; URINARY BLADDER; UTERUS; VAGINA

Histopathology - The following Tissues were Not Examined:

None

Project No. 285700300102 - Individual Macroscopic and Microscopic Pathology

Animal:	5CSG	Group:	5	Sex:	Female
Species:	Dog	Strain:	Beagle		
		Dose:	1 MFC APN01		
		Removal Reason:	Terminal Euthanasia		
		Study Day (Week) of Death:	15 (3)		

Gross Pathology Animal Details:

No animal details found

Gross Pathology Observations [Correlation]:

KIDNEY : Cyst, clear; left : (Comment) 2x2x2 mm (TGL)

Any remaining protocol required tissues, which have been examined, have no visible lesions

Gross Pathology - The following Tissues were Not Examined:

None

Histopathology Animal Details:

No animal details found

Histopathology Observations [Correlation]:

KIDNEY : Mineralization; minimal
KIDNEY : Cyst; tubular [KIDNEY : Cyst, clear; left : (Comment) 2x2x2 mm (G)]
LUNG : Infiltration, mixed cell; alveolar, focal, minimal
OVARY : Immaturity
OVIDUCT : (Note) One Of A Pair Available For Evaluation.
THYMUS : Cellularity, decreased; cortical, moderate
TONSIL : Hemorrhage; acute, minimal

Histopathology - The following Tissues were Within Normal Limits:

ARTERY, AORTA; BONE MARROW; BONE, FEMUR; BRAIN; CERVIX; ESOPHAGUS; EYE; GALLBLADDER; GLAND, ADRENAL; GLAND, MAMMARY; GLAND, PARATHYROID; GLAND, PITUITARY; GLAND, SALIVARY; GLAND, THYROID; HEART; LARGE INTESTINE, CECUM; LARGE INTESTINE, COLON; LARGE INTESTINE, RECTUM; LIVER; LYMPH NODE, BRONCHIAL; LYMPH NODE, MANDIBULAR; LYMPH NODE, MESENTERIC; MUSCLE, SKELETAL; NERVE, OPTIC; NERVE, SCIATIC; OVIDUCT; PANCREAS; SKIN; SMALL INTESTINE, DUODENUM; SMALL INTESTINE, ILEUM; SMALL INTESTINE, JEJUNUM; SPINAL CORD; SPLEEN; STOMACH; TONGUE; TRACHEA; URETER; URINARY BLADDER; UTERUS; VAGINA

Histopathology - The following Tissues were Not Examined:

None

Appendix I – Functional Observational Battery Data

TABLE OF CONTENTS

Page

Exhibit I-1. Functional Observational Battery Key I-2

Summary and Individual Animal Body Temperature Data:

Table I-1a. Males I-4
Table I-1b. Females I-5

Individual Animal Functional Observational Battery Observations:

Table I-2a. Males, Pre-Test I-6
Table I-2b. Females, Pre-Test I-7

Table I-3a. Males, Week 2 .... I-8
Table I-3b. Females, Week 2 .... I-9

Exhibit I-1: Functional Observational Battery Key

HOME CAGE OBSERVATIONS

Mental Status:

1. Normal – bright and alert, aware of its surroundings.
2. Hypoactive – dull, lethargic, but will move when stimulated.
3. Unresponsive – unwilling to move even when stimulated.
4. Comatose – totally unaware of its surroundings.
5. Hyperactive – abnormally active, anxious about its surroundings.
6. Aggressive – angry.

OBSERVATIONS MADE WHILE HANDLING THE ANIMAL

Gait and Posture:

1. Normal strength and coordination.
2. Stands and supports weight, but reluctant to move.
3. Stands and supports weight, but stumbles and falls.
4. Unable to stand or support weight.
5. Absence of any purposeful movement.

Wheelbarrow Test:

1. Normal – symmetrical movement of both thoracic limbs with the head extended.
2. Abnormal – asymmetrical movement of thoracic limbs. Head and neck may be flexed toward the floor to provide support, and paws may knuckle over.

Hopping Test (forelimb):

1. Normal – symmetrical movement.
2. Abnormal – asymmetrical, uneven movement.

Hopping Test (hind limb):

1. Normal – symmetrical movement.
2. Abnormal – asymmetrical, uneven movement.

Placing Response:

1. Normal.
2. Abnormal – could not complete the test.

Patellar Reflex:

1. Normal.
2. Hyperreflexic – exaggerated movement.
3. Hyporeflexic – very little movement
4. Areflexic – no movement

Perineal Reflex:

1. Normal.
2. Abnormal – no reflex observed

Hindlimb and forelimb Flexor Reflexes:

1. Normal.
2. Abnormal – no reflex observed.

Menace Reflex:

1. Normal.
2. Abnormal – no reflex observed.

Pupillary Reflex:

1. Normal.
2. Abnormal – no reflex observed.

Righting Reflex:

1. Normal.
2. Abnormal – cannot right itself.

Table I-1a: Summary and Individual Animal Body Temperature Data – Males

Group (Treatment)	Animal Number		Body Temperature (ºF)	
			Pre-Test	Week 2
**G 1 / M (Saline Control)**	1EZF		102.5	101.9
	1FUH		102.0	102.3
	1UYF		102.1	102.0
		**Mean:**	**102.2**	**102.1**
		SD:	0.26	0.21
		N:	3	3
		p value ‡:	*0.657*	*0.382*
**G 2 / M (Vehicle Control)**	2AGF		102.5	102.0
	2BSF		102.6	102.0
	2FRH		102.3	102.0
		**Mean:**	**102.5**	**102.0**
		SD:	0.15	0.00
		N:	3	3
**G 3 / M (APN01; 0.019 mg/L)**	3DWH		102.8	100.9
	3THF		103.9	101.9
	3TYF		102.8	101.8
		**Mean:**	**103.2**	**101.5**
		SD:	0.64	0.55
		N:	3	3
**G 4 / M (APN01; 0.038 mg/L)**	4EAH		104.0	101.3
	4JLH		101.6	101.2
	4YJF		100.7	101.6
		**Mean:**	**102.1**	**101.4**
		SD:	1.71	0.21
		N:	3	3
**G 5 / M (APN01; 0.075 mg/L)**	5GPH		103.7	100.7
	5ZOF		101.7	102.1
	5ZTF		102.7	102.3
		**Mean:**	**102.7**	**101.7**
		SD:	1.00	0.87
		N:	3	3

‡ Parameter statistically analyzed using ANOVA; no statistically significant differences in comparison to Group 1 or Group 2 were identified ($p < 0.05$).

Table I-1b: Summary and Individual Animal Body Temperature Data – Females

Group (Treatment)	Animal Number		Body Temperature (ºF) Pre-Test	Body Temperature (ºF) Week 2
**G 1 / M (Saline Control)**	1CBE		102.1	102.4
	1JKG		101.8	101.6
	1JSG		101.7	101.6
		**Mean:**	**101.9**	**101.9**
		SD:	0.21	0.46
		N:	3	3
		p value ‡:	*0.721*	*0.344*
**G 2 / M (Vehicle Control)**	2AJE		103.2	102.3
	2ZQE		102.1	101.6
	2FLE		101.4	101.3
		**Mean:**	**102.2**	**101.7**
		SD:	0.91	0.51
		N:	3	3
**G 3 / M (APN01; 0.019 mg/L)**	3CVG		102.5	102.3
	3FKE		101.8	101.9
	3ZRE		101.8	102.2
		**Mean:**	**102.0**	**102.1**
		SD:	0.40	0.21
		N:	3	3
**G 4 / M (APN01; 0.038 mg/L)**	4ACE		101.8	101.3
	4CAE		100.9	101.0
	4EVE		102.1	101.9
		**Mean:**	**101.6**	**101.4**
		SD:	0.62	0.46
		N:	3	3
**G 5 / M (APN01; 0.075 mg/L)**	5AIE		101.9	101.1
	5CGE		102.3	101.6
	5CSG		101.6	102.0
		**Mean:**	**101.9**	**101.6**
		SD:	0.35	0.45
		N:	3	3

‡ Parameter statistically analyzed using ANOVA; no statistically significant differences in comparison to Group 1 or Group 2 were identified ($p < 0.05$).

Table I-2a: Individual Animal Functional Observation Battery Observations – Males, Pre-Test

Animal Number	Mental Status	Gait and Posture	Wheelbarrow Test	Hopping Test		Placing Response	Patellar Reflex	Perineal Reflex	Flexor[a] Reflexes	Menace Reflex	Pupillary Reflex	Righting Reflex
				Forelimb	Hindlimb							
**GROUP 1 / M (Saline Control)**												
1EZF	1	1	1	1	1	1	1	1	1	1	1	1
1FUH	1	1	1	1	1	1	1	1	1	1	1	1
1UYF	1	1	1	1	1	1	1	1	1	1	1	1
**GROUP 2 / M (Vehicle Control)**												
2AGF	1	1	1	1	1	1	1	1	1	1	1	1
2BSF	1	1	1	1	1	1	1	1	1	1	1	1
2FRH	1	1	1	1	1	1	1	1	1	1	1	1
**GROUP 3 / M (APN01; 0.019 mg/L)**												
3DWH	1	1	1	1	1	1	1	1	1	1	1	1
3THF	1	1	1	1	1	1	1	1	1	1	1	1
3TYF	1	1	1	1	1	1	1	1	1	1	1	1
**GROUP 4 / M (APN01; 0.038 mg/L)**												
4EAH	1	1	1	1	1	1	1	1	1	1	1	1
4JLH	1	1	1	1	1	1	1	1	1	1	1	1
4YJF	1	1	1	1	1	1	1	1	1	1	1	1
**GROUP 5 / M (APN01; 0.075 mg/L)**												
5GPH	1	1	1	1	1	1	1	1	1	1	1	1
5ZOF	1	1	1	1	1	1	1	1	1	1	1	1
5ZTF	1	1	1	1	1	1	1	2	1	1	1	1

[a] Hindlimb and forelimb flexor reflexes

Table I-2b: Individual Animal Functional Observation Battery Observations – Females, Pre-Test

Animal Number	Mental Status	Gait and Posture	Wheelbarrow Test	Hopping Test		Placing Response	Patellar Reflex	Perineal Reflex	Flexor[a] Reflexes	Menace Reflex	Pupillary Reflex	Righting Reflex
				Forelimb	Hindlimb							
**GROUP 1 / F (Saline Control)**												
1CBE	1	1	1	1	1	1	1	1	1	1	1	1
1JKG	1	1	1	1	1	1	1	1	1	1	1	1
1JSG	1	1	1	1	1	1	1	1	1	1	1	1
**GROUP 2 / F (Vehicle Control)**												
2AJE	1	1	1	1	1	1	1	1	1	1	1	1
2ZQE	1	1	1	1	1	1	1	1	1	1	1	1
2FLE	1	1	1	1	1	1	1	1	1	1	1	1
**GROUP 3 / F (APN01; 0.019 mg/L)**												
3CVG	1	1	1	1	1	1	1	1	1	1	1	1
3FKE	1	1	1	1	1	1	1	1	1	1	1	1
3ZRE	1	1	1	1	1	1	1	1	1	1	1	1
**GROUP 4 / F (APN01; 0.038 mg/L)**												
4ACE	1	1	1	1	1	1	1	1	1	1	1	1
4CAE	1	1	1	1	1	1	1	1	1	1	1	1
4EVE	1	1	1	1	1	1	1	1	1	1	1	1
**GROUP 5 / F (APN01; 0.075 mg/L)**												
5AIE	1	1	1	1	1	1	1	1	1	1	1	1
5CGE	1	1	1	1	1	1	1	1	1	1	1	1
5CSG	1	1	1	1	1	1	1	1	1	1	1	1

[a] Hindlimb and forelimb flexor reflexes

Table I-3a: Individual Animal Functional Observation Battery Observations – Males, Week 2

Animal Number	Mental Status	Gait and Posture	Wheelbarrow Test	Hopping Test		Placing Response	Patellar Reflex	Perineal Reflex	Flexor[a] Reflexes	Menace Reflex	Pupillary Reflex	Righting Reflex
				Forelimb	Hindlimb							
**GROUP 1 / M (Saline Control)**												
1EZF	1	1	1	1	1	1	1	1	1	1	1	1
1FUH	1	1	1	1	1	1	1	1	1	1	1	1
1UYF	1	1	1	1	1	1	1	1	1	1	1	1
**GROUP 2 / M (Vehicle Control)**												
2AGF	1	1	1	1	1	1	1	1	1	1	1	1
2BSF	1	1	1	1	1	1	1	1	1	1	1	1
2FRH	1	1	1	1	1	1	1	1	1	1	1	1
**GROUP 3 / M (APN01; 0.019 mg/L)**												
3DWH	1	1	1	1	1	1	1	1	1	1	1	1
3THF	1	1	1	1	1	1	1	1	1	1	1	1
3TYF	1	1	1	1	1	1	1	1	1	1	1	1
**GROUP 4 / M (APN01; 0.038 mg/L)**												
4EAH	1	1	1	1	1	1	1	1	1	1	1	1
4JLH	1	1	1	1	1	1	1	1	1	1	1	1
4YJF	1	1	1	1	1	1	1	1	1	1	1	1
**GROUP 5 / M (APN01; 0.075 mg/L)**												
5GPH	1	1	1	1	1	1	1	1	1	1	1	1
5ZOF	1	1	1	1	1	1	1	1	1	1	1	1
5ZTF	1	1	1	1	1	1	1	1	1	1	1	1

[a] Hindlimb and forelimb flexor reflexes

Table I-3b: Individual Animal Functional Observation Battery Observations – Females, Week 2

Animal Number	Mental Status	Gait and Posture	Wheelbarrow Test	Hopping Test		Placing Response	Patellar Reflex	Perineal Reflex	Flexor[a] Reflexes	Menace Reflex	Pupillary Reflex	Righting Reflex
				Forelimb	Hindlimb							
**GROUP 1 / F (Saline Control)**												
1CBE	1	1	1	1	1	1	1	1	1	1	1	1
1JKG	1	1	1	1	1	1	1	1	1	1	1	1
1JSG	1	1	1	1	1	1	1	1	1	1	1	1
**GROUP 2 / F (Vehicle Control)**												
2AJE	1	1	1	1	1	1	1	1	1	1	1	1
2ZQE	1	1	1	1	1	1	1	1	1	1	1	1
2FLE	1	1	1	1	1	1	1	1	1	1	1	1
**GROUP 3 / F (APN01; 0.019 mg/L)**												
3CVG	1	1	1	1	1	1	1	1	1	1	1	1
3FKE	1	1	1	1	1	1	1	1	1	1	1	1
3ZRE	1	1	1	1	1	1	1	1	1	1	1	1
**GROUP 4 / F (APN01; 0.038 mg/L)**												
4ACE	1	1	1	1	1	1	1	1	1	1	1	1
4CAE	1	1	1	1	1	1	1	1	1	1	1	1
4EVE	1	1	1	1	1	1	1	1	1	1	1	1
**GROUP 5 / F (APN01; 0.075 mg/L)**												
5AIE	1	1	1	1	1	1	1	1	1	1	1	1
5CGE	1	1	1	1	1	1	1	1	1	1	1	1
5CSG	1	1	1	1	1	1	1	1	1	1	1	1

[a] Hindlimb and forelimb flexor reflexes

Appendix J – Abbreviations and Units of Measure; Urinalysis Key

ABBREVIATIONS AND UNITS OF MEASURE

**Abbreviation**	**Explanation**
**%**	percent
**#**	number
**°**	degree
**µL or uL**	microliter (10^{-6} L)
**ADR**	adrenal
**A/G**	albumin/globulin ratio
**ALB**	albumin (g/dL serum)
**ALP**	alkaline phosphatase (IU/L serum)
**ALT**	alanine aminotransferase (IU/L serum)
**ANOVA**	analysis of variance
**APTT**	activated partial thromboplastin time (second)
**AST**	aspartate aminotransferase (IU/L serum)
**BASO**	basophils (10^3 cells/µL blood; %)
**BRN**	brain
**BUN**	blood urea nitrogen (mg nitrogen/dL serum)
**C**	Celsius; centigrade
**CALC**	calcium (mg/dL serum)
**CHOL**	cholesterol (mg/dL serum)
**CK**	creatine kinase (U/L serum)
**CL**	chloride (mmol/L serum)
**CRE**	creatinine (mg/dL serum)
**dL or dl**	deciliter (10^{-1} L)
**DP**	drug product
**DS**	drug substance
**ECG**	electrocardiography or electrocardiogram
**EDTA**	ethylenediaminetetraacetic acid
**EOS**	eosinophils (10^3 cells/µL blood; %)
**EPI**	epididymis
**F**	female
**FBW**	fasted body weight
**FIB**	fibrinogen (mg/dL)
**fL**	femtoliter (10^{-15} liter)
**FOB**	functional observational battery
**g**	gram
**G**	group
**GLOB**	globulin (g/dL serum)
**GLU**	glucose (mg/dL serum)
**GSD**	geometric standard deviation
**HCT**	hematocrit (%)
**HGB**	hemoglobin (g/dL blood)
**HPLC**	high performance liquid chromatography
**HRT**	heart
**IU**	international units
**K**	potassium (mmol/L serum)
**KIDS**	kidneys
**kg or Kg**	kilogram (10^3 g)
**L or l**	liter
**LIV**	liver

ABBREVIATIONS AND UNITS OF MEASURE

Abbreviation	Explanation
**LPM**	liters per minute
**LUC**	large unstained cell count (10^3 cells/µL blood; %)
**LYMPH**	lymphocytes (10^3 cells/µL blood; %)
**M**	male
**MCH**	mean corpuscular hemoglobin (pg)
**MCHC**	mean corpuscular hemoglobin concentration (g/dL blood)
**MCV**	mean corpuscular volume (fL)
**MFC**	maximum feasible concentration
**mg**	milligram (10^{-3} grams)
**min. or min**	minute
**ml or mL**	milliliter (10^{-3} l)
**MMAD**	mass median aerodynamic diameter (µm)
**mmol**	millimole (1/1000th of a mole)
**MONO**	monocytes (10^3 cells/µL blood; %)
**N or No.**	number
**NA**	sodium (mmol/L serum)
**NEUT**	neutrophils (10^3 cells/µL blood; %)
**ng**	nanograms (10^{-9} grams)
**NS**	no more scheduled
**OVA**	ovaries
**pg**	picograms
**PO4**	inorganic phosphorus (mg/dL serum)
**PLT**	platelet count (10^3/µL blood)
**PT**	prothrombin time (second)
**QCM**	quartz crystal microbalance
**RBC**	erythrocyte count (10^6 cells/µL blood)
**RETIC**	reticulocyte count (10^9 cells/L blood; %)
**RH**	relative humidity (%)
**SD, S.D., or STD**	standard deviation
**SPL**	spleen
**SpO_2**	peripheral oxygen saturation (%)
**SvO_2**	venous blood oxygen saturation (mmHg)
**TBIL**	total bilirubin (mg/dL serum)
**TG**	triglycerides (mg/dL serum)
**THYM**	thymus
**TK**	toxicokinetic
**TP**	total protein (g/dL serum)
**TST**	testes
**UTER**	uterus
**vs.**	versus
**w or w/**	with
**WBC**	total leukocyte count (10^3 cells/µL blood)
**Wt**	weight
**x10e3**	thousand
**x10e6**	million
**x10e9**	billion

NOTE: Toxicokinetic-related abbreviations are presented in Appendix G (Serum Analysis and Toxicokinetic Report).

URINALYSIS KEY

**Bilirubin (BIL)**	**Glucose (GLU)**	**Ketones (KET)**	**Leukocytes (LEU)**
neg	Neg	neg	neg
1+ = 1 mg/dl	1+ = 50 mg/dl	1+ = 5 mg/dl	1+ = 25/µl
2+ = 3 mg/dl	2+ = 100 mg/dl	2+ = 15 mg/dl	2+ = 100/µl
3+ = 6 mg/dl	3+ = 250 mg/dl	3+ = 50 mg/dl	3+ = 500/µl
	4+ = 1000 mg/dl	4+ = 150 mg/dl	

**Erythrocytes (ERY)**	**Protein (PRO)**	**Urobilinogen (UBG)**	**Nitrite (NIT)**
neg	neg	neg	negative
1+ = 10/µl	1+ = 15 mg/dl	1+ = 1 mg/dl	positive
2+ = 25/µl	2+ = 30 mg/dl	2+ = 4 mg/dl	
3+ = 50/µl	3+ = 100 mg/dl	3+ = 8 mg/dl	
4+ = 150/µl	4+ = 500 mg/dl	4+ = 12 mg/dl	
5+ = 250/µl			

**Abbreviation/Term**	**Explanation/Measurement Criteria**
**Casts**	#/LPF
**CLA**	clarity
**COL**	color
**EP Cells**	Epithelial cells (#/HPF)
**HPF**	high power field; 400x or 40x10
**LPF**	low power field (100x or 10x10)
**MR**	motile rod-shaped bacteria; scale/HPF
**neg**	negative/normal
**NMR**	non-motile rod-shaped bacteria; scale/HPF
**PO4**	phosphate crystals, triple; scale/HPF
**RBC**	red blood cells; #/HPF
**RI**	refractive index
**SG**	specific gravity
**Sperm**	scale/LPF
**WBC**	white blood cells; #/HPF

Microscopic Analysis Scale:
0 = none seen
1 = occasionally noted
2 = noted in every field
3 = large amounts in every field
4 = full fields

Appendix K – Test Material Documentation

APN01 (ACE2 Drug Product) Certificate of Analysis (page 1 of 2)

POLYMUN SCIENTIFIC
Immunbiologische Forschung GmbH

A-3400 Klosterneuburg, Austria
Donaustraße 99
Tel.: +43-2243-25060-300
Fax: +43-2243-25060-399
E.Mail: office@polymun.com
http://www.polymun.com

CERTIFICATE OF ANALYSIS

**Product:** ACE2 Drug Product (5.0 mg/ml)	**Lot Number:** ACE20620-B
**Specification:** ACE2/SPEC/009-01	**Storage Conditions:** 5 ± 3 °C
**Date of Manufacture:** 09.06.2020	**Retest date:** 12/2020
**Site of Manufacture:** Polymun Scientific, Donaustraße 99, A-3400 Klosterneuburg, Austria	

Test Method Reference[1)]	Acceptance criteria	Result	Pass or Fail
**Appearance and Description**			
Visual appearance 224/SOP/011	clear and colourless	Clear, colourless	pass
Visible particles 224/SOP/011	practically free from visible particles	No visible particles	pass
**Identity**			
SDS-PAGE 223/SOP/002, 223/SOP/004	Protein band between 75 kD and 150 kD marker bands banding profile comparable to control standard	conforms	pass
Western Blot 223/SOP/002, 223/SOP/006	Protein band between 75 kD and 150 kD marker bands positive reaction of protein band with specific antibody banding profile comparable to control standard	conforms	pass
Native-PAGE 223/SOP/009, 223/SOP/004	Main band between 242 kD and 480 kD marker bands banding profile comparable to control standard	conforms	pass
Isoelectric Focussing 223/SOP/010, 223/SOP/004	Banding profile comparable to control standard bands between pI 4.2 and 5.3	conforms	pass
**Purity**			
Contents of dimers, di-dimers and polymers, monomers and fragments (SEC-HPLC) ACE2/SOP/007 [2)]	dimers ≥ 95.0 % di-dimers + polymers ≤ 2.0 % monomers ≤ 5.0 % fragments ≤ 2.0 % retention time of main peak comparable to that of control standard	dimers: 99.2 % di-dimers: 0.1 % monomers: 0.5 % fragments: 0.2 % retention time comparable to that of control standard	pass
QSDS-PAGE ACE2/SOP/001	Purity ≥ 95.0 %	98.9 %	pass
RP-HPLC ACE2/SOP/004	Main peak ≥ 90.0 % Retention time of main peak comparable to that of control standard	99.8 % retention time comparable to that of control standard	pass
**Quantity**			
ACE2 content (OD_{280}) 221/SOP/008	5.0 ± 0.5 mg/ml	4.9 mg/ml	pass
**Potency**			
Specific catalytic activity (HPLC) ACE2/SOP/003	0.025 – 0.075 µkat/mg	0.043 µkat/mg	pass

Version 01 Page 1 of 2

APN01 (ACE2 Drug Product) Certificate of Analysis (page 2 of 2)

POLYMUN SCIENTIFIC
Immunbiologische Forschung GmbH

A-3400 Klosterneuburg, Austria
Donaustraße 99
Tel.: +43-2243-25060-300
Fax: +43-2243-25060-399
E.Mail: office@polymun.com
http://www.polymun.com

Test Method Reference[1]	Acceptance criteria	Result	Pass or Fail
**Contaminants**			
Endotoxin (LAL-assay) 221/SOP/001	≤ 10.42 EU/ml	< 0.15 EU/ml	pass
Sterility [3] USP <71>, Ph.Eur2.6.1	sterile	sterile	pass
**General characteristics**			
pH 224/SOP/016	7.0 ± 0.5	7.0	pass
Osmolality 224/SOP/009	330 ± 50 mOsmol/kg	327 mOsmol/kg	pass
Sialic acid (SIA) content (bound) ACE2/SOP/005	6.0 – 17.0 mol SIA/mol ACE2	10.3 mol SIA/mol ACE2	pass
Particulate Matter 224/SOP/018	Particles ≥ 10 µm: ≤6000 particles / container Particles ≥ 25 µm: ≤600 particles / container	Particles ≥ 10 µm: 26 particles / container Particles ≥ 25 µm: 3 particles / container	pass
Extractable Volume 224/SOP/014	Extractable volume (ml) is not less than the stated nominal volume	4.2 ml	pass

1. Unless specified otherwise, tests were performed at Polymun Scientific, Donaustraße 99, A-3400 Klosterneuburg, Austria.
2. Tested according to version 02 of the referenced SOP.
3. Test was performed at AGES, Institut für Med. Mikrobiologie und Hygiene, Beethovenstraße 6, A-8010 Graz, Austria.

The material was tested in compliance with GMP and conforms to the specification ACE2/SPEC/009-01 and.

Date: 12.10.2020

Quality Assurance / Qualified Person

Version 01 Page 2 of 2

APN01 PS80 Concentrate (Lot 15145-29-2)

285700300102

COPY

MRIGlobal

The science you expect.
The people you know.

MRIGlobal
425 Volker Boulevard
Kansas City, Missouri 64110
Telephone (816) 753-7600
Telefax (816) 753-5519

STATEMENT OF PRODUCT COMPOSITION

Project No. 111069

Product Name:	APN01 PS80 Concentrate
Lot No.:	15145-29-2
Manufacture Date:	October 7, 2020
Manufacturer:	MRIGlobal
Storage Conditions:	5°C

Formulation: 10 mM Sodium Phosphate, 210 mM Sucrose, 25 mM Arginine HCl, 10 mM Sodium Chloride, 50 µM Zinc Chloride, 0.154% (w/w) Polysorbate 80, in Water.

Approved:

Jonathan White, Ph.D.
Principal Investigator
Pharmaceutical Sciences

December 17, 2020

MRIGlobal-PDRM\111069_CoA14522-77-3

APN01 PS80 Concentrate (Lot 15241-4-3)

285700300102

COPY

MRIGlobal

The science you expect.
The people you know.

MRIGlobal
425 Volker Boulevard
Kansas City, Missouri 64110
Telephone (816) 753-7600
Telefax (816) 753-5519

STATEMENT OF PRODUCT COMPOSITION

Project No. 111069

Product Name:	APN01 PS80 Concentrate
Lot No.:	15241-4-3
Manufacture Date:	October 30, 2020
Manufacturer:	MRIGlobal
Storage Conditions:	5°C

Formulation: 10 mM Sodium Phosphate, 210 mM Sucrose, 25 mM Arginine HCl, 10 mM Sodium Chloride, 50 µM Zinc Chloride, 0.154% (w/w) Polysorbate 80, in Water.

Approved:

Jonathan White, Ph.D.
Principal Investigator
Pharmaceutical Sciences

November 2, 2020

MRIGlobal-PDRM111069_CoA14622-77-3

Saline (Lot 87-116-4B); page 1 of 3

285700300102

COPY

Hospira

Highway 301 North

Rocky Mount, North Carolina 27801

Certificate of Analysis for BQ and CQ Product Lot

List Number: **7138 0439**

Product Name: **0.9% SODIUM CHLORIDE IRRIGATION, USP**

Lot Number: **87-116-4B**

Expiration Date: **March 01, 2021**

Test	Specification	Results
Bacterial Endotoxin / B0610	<=0.50 EU/ml	Less Than 0.06 EU/ml
Identification Chloride / C0003	RESPONDS TO THE TEST	RESPONDS TO THE TEST
pH Reported / C0021	4.5 - 7.0	5.8
Sodium Chloride / C0042	95.0 - 105.0 %	100.2 %
Iron / C0869	NMT 2 ppm (0.0002%)	NMT 2 ppm (0.0002%)
Identification Sodium / C1648	RESPONDS TO THE TEST	RESPONDS TO THE TEST
AG / C2377	NMT 5 ng/mL	NMT 5 ng/mL
AG REPORTED / C2377		LT 2.50 ng/ml
AS / C2377	NMT 7.5 ng/mL	NMT 7.5 ng/mL
AS REPORTED / C2377		ND
AU / C2377	NMT 50 ng/mL	NMT 50 ng/mL
AU REPORTED / C2377		LT 25.0 ng/ml
BA / C2377	NMT 350 ng/mL	NMT 350 ng/mL
BA REPORTED / C2377		ND
CD / C2377	NMT 1.0 ng/mL	NMT 1.0 ng/mL
CD REPORTED / C2377		LT 0.50 ng/ml
CO / C2377	NMT 2.5 ng/mL	NMT 2.5 ng/mL
CO REPORTED / C2377		LT 1.25 ng/ml
CR / C2377	NMT 550 ng/mL	NMT 550 ng/mL
CR REPORTED / C2377		LT 25.0 ng/ml
CU / C2377	NMT 65 ng/mL	NMT 65 ng/mL

Page 1/3

Saline (Lot 87-116-4B); page 2 of 3

285700300102

COPY

List Number: 7138 0439

Product Name: 0.9% SODIUM CHLORIDE IRRIGATION, USP

Lot Number: 87-116-4B

Expiration Date: March 01, 2021

Test	Specification	Results
CU REPORTED / C2377		LT 25.0 ng/ml
FE / C2377	NMT 650 ng/mL	NMT 650 ng/mL
FE REPORTED / C2377		ND
HG / C2377	NMT 0.75 ng/mL	NMT 0.75 ng/mL
HG REPORTED / C2377		LT 0.38 ng/ml
IR / C2377	NMT 5 ng/mL	NMT 5 ng/mL
IR REPORTED / C2377		ND
LI / C2377	NMT 125 ng/mL	NMT 125 ng/mL
LI REPORTED / C2377		LT 25.0 ng/ml
MN / C2377	NMT 125 ng/mL	NMT 125 ng/mL
MN REPORTED / C2377		LT 25.0 ng/ml
MO / C2377	NMT 45 ng/mL	NMT 45 ng/mL
MO REPORTED / C2377		LT 20.0 ng/ml
NI / C2377	NMT 10 ng/mL	NMT 10 ng/mL
NI REPORTED / C2377		ND
OS / C2377	NMT 5 ng/mL	NMT 5 ng/mL
OS REPORTED / C2377		LT 2.50 ng/ml
PB / C2377	NMT 2.5 ng/mL	NMT 2.5 ng/mL
PB REPORTED / C2377		LT 1.25 ng/ml
PD / C2377	NMT 5 ng/mL	NMT 5 ng/mL
PD REPORTED / C2377		LT 2.50 ng/ml
PT / C2377	NMT 5 ng/mL	NMT 5 ng/mL
PT REPORTED / C2377		LT 2.50 ng/ml
RH / C2377	NMT 5 ng/mL	NMT 5 ng/mL
RH REPORTED / C2377		ND
RU / C2377	NMT 5 ng/mL	NMT 5 ng/mL
RU REPORTED / C2377		LT 2.50 ng/ml
SB / C2377	NMT 45 ng/mL	NMT 45 ng/mL
SB REPORTED / C2377		LT 20.0 ng/ml
SE / C2377	NMT 40 ng/mL	NMT 40 ng/mL
SE REPORTED / C2377		ND
SN / C2377	NMT 300 ng/mL	NMT 300 ng/mL

Page 2/3

Saline (Lot 87-116-4B); page 3 of 3

285700300102

COPY

List Number: 7138 0439

Product Name: 0.9% SODIUM CHLORIDE IRRIGATION, USP

Lot Number: 87-116-4B

Expiration Date: March 01, 2021

Test	Specification	Results
SN REPORTED / C2377		LT 25.0 ng/ml
TL / C2377	NMT 4 ng/mL	NMT 4 ng/mL
TL REPORTED / C2377		LT 2.00 ng/ml
V / C2377	NMT 5 ng/mL	NMT 5 ng/mL
V REPORTED / C2377		ND
ZN / C2377	NMT 650 ng/mL	NMT 650 ng/mL
ZN REPORTED / C2377		ND

This is to Certify that Quality Assurance of the above mentioned production lot including testing, investigation, quality control and general standards of workmanship has been audited by the Quality Assurance Auditor(s) and have found that 0.9% SODIUM CHLORIDE IRRIGATION, USP to be in COMPLIANCE with the requirements per procedures and/or recognized standards.

Authorized By: Ira Moss Authorized Date: April 16, 2018

Printed By: [signature] Print Date: 16 APR 2018

Confidential

Page 3/3

Saline (Batch 1902033)

LABORATORIOS
BIOGALENIC S.A. De C.V.
BOULEVARD DEL EJERCITO NACIONAL Km. 5 ½,
SOYAPANGO
SAN SALVADOR, EL SALVADOR, C.A.
TELEFONO: 2227-4133 (PBX) FAX: 2227-4466
APARTADO POSTAL 1639
E-MAIL: biogal@biogalenic.com.sv

COPY

QUALITY CONTROL DEPARTMENT
FINISHED PRODUCT ANALYSIS CERTIFICATE/
DEPARTAMENTO DE CONTROL DE CALIDAD
CERTIFICADO DE ANÁLISIS DE PRODUCTO TERMINADO

Product Name (Nombre del Producto):
VETERINARY 0.9% SODIUM CHLORIDE INJECTION
(SOLUCIÓN INYECTABLE VETERINARIA DE CLORURO DE SODIO AL 0.9 %)

BATCH<br>LOTE	CATALOG<br>CATÁLOGO	PRESENTATION<br>PRESENTACIÓN	MANUFACTURE DATE<br>FECHA FABRICACIÓN	EXPIRATION DATE<br>FECHA EXPIRACIÓN	UNITS<br>UNIDADES
**1902033**	**V0080**	**250 mL**	**02/2019**	**02/2022**	**45,360**

TESTS /DETERMINACIONES	RESULTS /RESULTADOS	LIMITS /LIMITES
**Solution Appearance/** Aspecto de la solución	**The sample is transparent colorless and free of visible particles/** La muestra es incolora transparente y libre de partículas visibles	**The sample must be transparent colorless and free of visible particles/** La muestra debe ser incolora transparente y libre de partículas visibles
**CHLORIDE Identity/** CLORUROS Identidad	**Positive** /Positiva	**Positive** /Positiva
**SODIUM Identity** /SODIO Identidad	**Positive** /Positiva	**Positive** /Positiva
**pH**	5.4	**4.5 – 7.0**
**Iron** /Hierro	< 2 ppm	**No more than 2 ppm/** No más de 2 ppm
**Volume in container/** Volumen en envase	254 mL	**The volume is not less than the labeled volume/** El volumen no es menor que el volumen etiquetado
**Paticulate matter in injections** /Partículas en inyectables	Partículas ≥10 μm: 0.12/mL<br>Partículas ≥25 μm: 0.00/mL	**Particles ≥10 μm: does not exceed 25/mL**<br>**Particles ≥25 μm: does not exceed 3/mL**<br>/Partículas ≥10 μm: no excede 25/mL<br>Partículas ≥25 μm: no excede 3/mL
**Bacterial endotoxins** / Endotoxinas Bacterianas	< 0.2 EU/Ml	No more than 0.5 EU / mL<br>**No más de 0.5 EU / mL**
**Assay**/Ensayo	0.885 g/100 mL	0.855 – 0.945 g/100 mL
**Sterility** /Esterilidad	**The sample not show any growth at 14 days /** La muestra no presenta crecimiento a los 14 días	**The sample must not show any growth at 14 days /** La muestra no debe presentar crecimiento a los 14 días

METHOD (MÉTODO): **USP 40**
RESULT (RESULTADOS): **APPROVED** (APROBADO)

285700300102

LIC. OSCAR [illegible] RODRIGUEZ PONCE
QUALITY CONTROL MANAGER/ GERENTE DE CONTROL DE CALIDAD

SAN SALVADOR, FEBRUARY 28, 2019
Fs-Ps-CC-240-1 (05/14)

APN01 Diluent (Lot 15145-29-1)

285700300102

COPY

MRIGlobal

The science you expect.
The people you know.

MRIGlobal
425 Volker Boulevard
Kansas City, Missouri 64110
Telephone (816) 753-7600
Telefax (816) 753-5519

STATEMENT OF PRODUCT COMPOSITION

Project No. 111069

Product Name:	APN01 Diluent
Lot No.:	15145-29-1
Manufacture Date:	October 7, 2020
Manufacturer:	MRIGlobal
Storage Conditions:	5°C

Formulation: 10 mM Sodium Phosphate, 210 mM Sucrose, 25 mM Arginine HCl, 10 mM Sodium Chloride, 50 µM Zinc Chloride, 0.02% (w/w) Polysorbate 80, in Water.

Approved:

Jonathan White, Ph.D.
Principal Investigator
Pharmaceutical Sciences

December 17, 2020

MRIGlobal-PDRM\111069_CoA14622-77-3

APN01 Diluent (Lot 15241-4-2)

285700300102

MRIGlobal
The science you expect.
The people you know.

COPY

MRIGlobal
425 Volker Boulevard
Kansas City, Missouri 64110
Telephone (816) 753-7600
Telefax (816) 753-5519

STATEMENT OF PRODUCT COMPOSITION

Project No. 111069

Product Name:	APN01 Diluent
Lot No.:	15241-4-2
Manufacture Date:	October 30, 2020
Manufacturer:	MRIGlobal
Storage Conditions:	5°C

Formulation: 10 mM Sodium Phosphate, 210 mM Sucrose, 25 mM Arginine HCl, 10 mM Sodium Chloride, 50 µM Zinc Chloride, 0.02% (w/w) Polysorbate 80, in Water.

Approved:

Jonathan White, Ph.D.
Principal Investigator
Pharmaceutical Sciences

November 2, 2020

MRIGlobal-PDRM\111069_CoA14622-77-3
